# Pharmacological interventions for the prevention of bleeding in people undergoing elective hip or knee surgery: a systematic review and network meta‐analysis

**DOI:** 10.1002/14651858.CD013295.pub2

**Published:** 2024-01-16

**Authors:** Victoria N Gibbs, Rita Champaneria, Josie Sandercock, Nicky J Welton, Louise J Geneen, Susan J Brunskill, Carolyn Dorée, Catherine Kimber, Antony JR Palmer, Lise J Estcourt

**Affiliations:** Systematic Review InitiativeNHS Blood and TransplantOxfordUK; Nuffield Department of Clinical Laboratory SciencesUniversity of OxfordOxfordUK; Bristol Medical SchoolUniversity of BristolBristolUK; Nuffield Department of Orthopaedics, Rheumatology and Musculoskeletal SciencesUniversity of OxfordOxfordUK; Haematology/Transfusion MedicineNHS Blood and TransplantOxfordUK

**Keywords:** Adult, Female, Humans, Male, Aminocaproic Acid, Aminocaproic Acid/therapeutic use, Aprotinin, Aprotinin/therapeutic use, Deamino Arginine Vasopressin, Fibrin, Hemorrhage, Hemorrhage/etiology, Orthopedic Procedures, Orthopedic Procedures/adverse effects, Stroke, Stroke/drug therapy, Tranexamic Acid, Tranexamic Acid/therapeutic use

## Abstract

**Background:**

Hip and knee replacement surgery is a well‐established means of improving quality of life, but is associated with a significant risk of bleeding. One‐third of people are estimated to be anaemic before hip or knee replacement surgery; coupled with the blood lost during surgery, up to 90% of individuals are anaemic postoperatively. As a result, people undergoing orthopaedic surgery receive 3.9% of all packed red blood cell transfusions in the UK. Bleeding and the need for allogeneic blood transfusions has been shown to increase the risk of surgical site infection and mortality, and is associated with an increased duration of hospital stay and costs associated with surgery.

Reducing blood loss during surgery may reduce the risk of allogeneic blood transfusion, reduce costs and improve outcomes following surgery. Several pharmacological interventions are available and currently employed as part of routine clinical care.

**Objectives:**

To determine the relative efficacy of pharmacological interventions for preventing blood loss in elective primary or revision hip or knee replacement, and to identify optimal administration of interventions regarding timing, dose and route, using network meta‐analysis (NMA) methodology.

**Search methods:**

We searched the following databases for randomised controlled trials (RCTs) and systematic reviews, from inception to 18 October 2022: CENTRAL (the Cochrane Library), MEDLINE (Ovid), Embase (Ovid), CINAHL (EBSCO*host*), Transfusion Evidence Library (Evidentia), ClinicalTrials.gov and WHO International Clinical Trials Registry Platform (ICTRP).

**Selection criteria:**

We included RCTs of people undergoing elective hip or knee surgery only.

We excluded non‐elective or emergency procedures, and studies published since 2010 that had not been prospectively registered (Cochrane Injuries policy). There were no restrictions on gender, ethnicity or age (adults only). We excluded studies that used standard of care as the comparator.

Eligible interventions included: antifibrinolytics (tranexamic acid (TXA), aprotinin, epsilon‐aminocaproic acid (EACA)), desmopressin, factor VIIa and XIII, fibrinogen, fibrin sealants and non‐fibrin sealants.

**Data collection and analysis:**

We performed the review according to standard Cochrane methodology. Two authors independently assessed trial eligibility and risk of bias, and extracted data. We assessed the certainty of the evidence using CINeMA. We presented direct (pairwise) results using RevMan Web and performed the NMA using BUGSnet.

We were interested in the following primary outcomes: need for allogenic blood transfusion (up to 30 days) and all‐cause mortality (deaths occurring up to 30 days after the operation), and the following secondary outcomes: mean number of transfusion episodes per person (up to 30 days), re‐operation due to bleeding (within seven days), length of hospital stay and adverse events related to the intervention received.

**Main results:**

We included a total of 102 studies. Twelve studies did not report the number of included participants; the other 90 studies included 8418 participants. Trials included more women (64%) than men (36%).

In the NMA for allogeneic blood transfusion, we included 47 studies (4398 participants). Most studies examined TXA (58 arms, 56%). We found that TXA, given intra‐articularly and orally at a total dose of greater than 3 g pre‐incision, intraoperatively and postoperatively, ranked the highest, with an anticipated absolute effect of 147 fewer blood transfusions per 1000 people (150 fewer to 104 fewer) (53% chance of ranking 1st) within the NMA (risk ratio (RR) 0.02, 95% credible interval (CrI) 0 to 0.31; moderate‐certainty evidence). This was followed by TXA given orally at a total dose of 3 g pre‐incision and postoperatively (RR 0.06, 95% CrI 0.00 to 1.34; low‐certainty evidence) and TXA given intravenously and orally at a total dose of greater than 3 g intraoperatively and postoperatively (RR 0.10, 95% CrI 0.02 to 0.55; low‐certainty evidence).

Aprotinin (RR 0.59, 95% CrI 0.36 to 0.96; low‐certainty evidence), topical fibrin (RR 0.86, CrI 0.25 to 2.93; very low‐certainty evidence) and EACA (RR 0.60, 95% CrI 0.29 to 1.27; very low‐certainty evidence) were not shown to be as effective compared with TXA at reducing the risk of blood transfusion.

We were unable to perform an NMA for our primary outcome all‐cause mortality within 30 days of surgery due to the large number of studies with zero events, or because the outcome was not reported.

In the NMA for deep vein thrombosis (DVT), we included 19 studies (2395 participants). Most studies examined TXA (27 arms, 64%). No studies assessed desmopressin, EACA or topical fibrin. We found that TXA given intravenously and orally at a total dose of greater than 3 g intraoperatively and postoperatively ranked the highest, with an anticipated absolute effect of 67 fewer DVTs per 1000 people (67 fewer to 34 more) (26% chance of ranking first) within the NMA (RR 0.16, 95% CrI 0.02 to 1.43; low‐certainty evidence). This was followed by TXA given intravenously and intra‐articularly at a total dose of 2 g pre‐incision and intraoperatively (RR 0.21, 95% CrI 0.00 to 9.12; low‐certainty evidence) and TXA given intravenously and intra‐articularly, total dose greater than 3 g pre‐incision, intraoperatively and postoperatively (RR 0.13, 95% CrI 0.01 to 3.11; low‐certainty evidence). Aprotinin was not shown to be as effective compared with TXA (RR 0.67, 95% CrI 0.28 to 1.62; very low‐certainty evidence).

We were unable to perform an NMA for our secondary outcomes pulmonary embolism, myocardial infarction and CVA (stroke) within 30 days, mean number of transfusion episodes per person (up to 30 days), re‐operation due to bleeding (within seven days), or length of hospital stay, due to the large number of studies with zero events, or because the outcome was not reported by enough studies to build a network.

There are 30 ongoing trials planning to recruit 3776 participants, the majority examining TXA (26 trials).

**Authors' conclusions:**

We found that of all the interventions studied, TXA is probably the most effective intervention for preventing bleeding in people undergoing hip or knee replacement surgery. Aprotinin and EACA may not be as effective as TXA at preventing the need for allogeneic blood transfusion. We were not able to draw strong conclusions on the optimal dose, route and timing of administration of TXA. We found that TXA given at higher doses tended to rank higher in the treatment hierarchy, and we also found that it may be more beneficial to use a mixed route of administration (oral and intra‐articular, oral and intravenous, or intravenous and intra‐articular). Oral administration may be as effective as intravenous administration of TXA. We found little to no evidence of harm associated with higher doses of tranexamic acid in the risk of DVT. However, we are not able to definitively draw these conclusions based on the trials included within this review.

## Plain language summary

**What is the best medication to stop bleeding in those having non‐emergency hip or knee surgery?**

**Key messages**

• Tranexamic acid may be an effective medicine to help blood to clot during hip or knee replacement surgery, which reduces bleeding and the need for a blood transfusion (replacing lost blood with donated blood).

• A high dose of this medicine and administering it in more than one way (for example, as a tablet and injected into the joint at the end of surgery) appears to work best.

• Tranexamic acid is potentially as effective when given as a tablet as when injected into a vein.

• There is little to no evidence to indicate that higher doses of tranexamic acid increase the risk of blood clots in the leg or other harms.

**Background**

***Why is it important to stop bleeding during hip or knee surgery?***

Controlling bleeding during surgery reduces the likelihood of the person developing anaemia and requiring a blood transfusion, which carries the risk of complications. Anaemia occurs when the number of red blood cells (haemoglobin) available to carry oxygen is lower than normal. This causes symptoms such as fatigue, weakness, dizziness, shortness of breath and, in severe cases, can be life‐threatening. Preventing blood loss during surgery improves patient outcomes, decreases healthcare costs and preserves the limited supplies of donated blood.

***Is there a medication that helps control bleeding?***

Many research studies have investigated whether certain medication, such as tranexamic acid, can help minimise blood loss during surgery. Most of the studies test different doses of medications, different methods of administration and different times of use, either before, during or after an operation.

**What did we want to find out?**

We wanted to find out whether medication can reduce blood loss and the need for blood transfusions in people having hip or knee replacement surgery. We also wanted to know the most effective way of giving this medication to patients.

**What did we do?**

We searched for studies comparing different medications that could help reduce bleeding in adults undergoing planned hip or knee replacement surgery. We also searched for studies comparing medication with a placebo. A placebo is a 'dummy' medication that looks or tastes identical to the medication being tested.

**What did we find?**

We found 102 studies. Twelve studies did not report the number of included participants; in the other 90 studies, there were 8418 included participants. On average, people were between 50 and 77 years of age and 64% were women. The smallest study included 16 people and the largest involved 300. Studies took place around the globe, with the highest number in Europe and Asia. Of those studies that reported a source of funding, seven were fully funded and five partly funded by pharmaceutical (medication) companies. All studies investigated medications that helped the blood to clot. However, most studies assessed the effectiveness and safety of tranexamic acid administered intravenously (injected into a vein), orally (swallowing a tablet or liquid), injected into the joint during surgery, or with a combination of these methods.

**Main results**

Our review of the studies showed the following:

• Tranexamic acid was the most effective at controlling bleeding compared to other medications.

• Adults who underwent hip or knee replacement surgery required fewer blood transfusions if they were given tranexamic acid.

• Tranexamic acid given in higher doses using multiple methods of administration, such as orally and injected into the joint during surgery, may be more likely to prevent bleeding.

• Taking tranexamic acid orally is probably as effective at preventing a blood transfusion as injecting the medication into a vein.

We were unable to decide on the optimal dose, which combination of methods are best to administer tranexamic acid and when it is most beneficial to use it (before, during and after surgery). We did not find evidence to suggest that higher doses of tranexamic acid increase the risk of developing a blood clot, or any other harms.

**What are the limitations of the evidence?**

We are not able to definitively draw conclusions based on the trials included within this review. We have little confidence in the evidence for some outcomes, and are not confident about the evidence for others. This is because it is possible that people in the studies were aware of which treatment they were getting. Also, the studies were small and did not all provide data about everything in which we were interested.

**Ongoing studies and future updates**

Thirty studies with a planned total of 3776 participants are currently ongoing. These studies should be completed and published within the next few years. Once the authors publish their data, we may update our analyses and provide stronger answers than we can at present.

**How up‐to‐date is this evidence?**

The evidence is current to 18 October 2022.

## Summary of findings

**Summary of findings 1:** Summary of findings: Risk of a blood transfusion up to 30 days post‐surgery

**Estimates of effects, credible intervals and certainty of the evidence for the prevention of bleeding in hip and knee replacement patients**							
**Patient or population**: individuals undergoing planned hip or knee replacement surgery **Interventions**: antifibrinolytics (tranexamic acid, aprotinin or epsilon‐aminocaproic acid), fibrin sealants **Comparator (reference)**: placebo **Outcome**: risk of requiring a blood transfusion within 30 days of surgery **Setting**: elective orthopaedic surgery (See Figure 1)							
**Total studies:** 47 **Total participants:** 4398	Relative effect* (95% CrI)	Anticipated absolute effect**			**Certainty of evidence** **(**Table 2)	Median nodal ranking (95% CrI)***	Probability of ranking 1st (%)****
		***Without* intervention**	***With* intervention**	**Difference**			
TXA given orally and intra‐articularly at a total dose of greater than 3 g pre‐incision, intraoperatively and postoperatively	0.02 (0 0.31)	150 per 1000	3 per 1000	147 fewer per 1000 (150 fewer to 104 fewer per 1000)	⊕⊕⊕⊝ Moderate (due to reporting bias)	1 (1 to 13)	53%
TXA given orally at a total dose of 3 g pre‐incision and postoperatively	0.06 (0 to 1.34)	150 per 1000	9 per 1000	141 fewer per 1000 (150 fewer to 51 more per 1000)	⊕⊕⊝⊝ Low (due to imprecision)	5 (1 to 28)	18%
TXA given intravenously and orally at a total dose of greater than 3 g intraoperatively and postoperatively	0.1 (0.02 to 0.55)	150 per 1000	15 per 1000	135 fewer per 1000 (147 fewer to 68 fewer per 1000)	⊕⊕⊝⊝ Low (due to within‐study bias, heterogeneity)	6 (1 to 21)	5%
TXA given intravenously at a total dose of 2 g pre‐incision	0.09 (0.02 to 0.56)	150 per 1000	14 per 1000	136 fewer per 1000 (147 fewer to 66 fewer per 1000)	⊕⊕⊕⊝ Moderate (due to within‐study bias)	6 (1 to 21)	5%
TXA given intravenously and intra‐articularly at a total dose of 2 g intraoperatively	0.09 (0.03 to 0.3)	150 per 1000	14 per 1000	136 fewer per 1000 (146 fewer to 105 fewer per 1000)	⊕⊕⊕⊝ Moderate (due to within‐study bias)	5 (1 to 14)	4%
TXA given intravenously and orally at a total dose of greater than 3 g pre‐incision and postoperatively	0.21 (0.02 to 2.08)	150 per 1000	32 per 1000	118 fewer per 1000 (147 fewer to 162 more per 1000)	⊕⊕⊝⊝ Low (due to imprecision)	11 (1 to 29)	3%
TXA given intravenously at a total dose of 1 g pre‐incision and postoperatively	0.18 (0.03 to 1.11)	150 per 1000	27 per 1000	123 fewer per 1000 (146 fewer to 17 more per 1000)	⊕⊕⊝⊝ Low (due to within‐study bias and imprecision)	11 (2 to 29)	2%
TXA given intravenously and intra‐articularly at a total dose of greater than 3 g pre‐incision, intraoperatively and postoperatively	0.18 (0.03 to 1.17)	150 per 1000	27 per 1000	123 fewer per 1000 (146 fewer to 26 more per 1000)	⊕⊕⊝⊝ Low (due to imprecision)	11 (2 to 28)	2%
TXA given intra‐articularly at a total dose of 2 g intraoperatively	0.17 (0.02 to 1.47)	150 per 1000	26 per 1000	124 fewer per 1000 (147 fewer to 71 more per 1000)	⊕⊕⊝⊝ Low (due to imprecision)	10 (2 to 28)	2%
TXA given intravenously at a total dose of 1 g intraoperatively and postoperatively	0.15 (0.03 to 0.74)	150 per 1000	23 per 1000	127 fewer per 1000 (146 fewer to 39 fewer per 1000)	⊕⊕⊝⊝ Low (due to within‐study bias and imprecision)	8 (2 to 24)	2%
TXA given orally at a total dose of greater than 3 g pre‐incision and postoperatively	0.16 (0.03 to 0.84)	150 per 1000	24 per 1000	126 fewer per 1000 (146 fewer to 24 fewer per 1000)	⊕⊝⊝⊝ Very low (due to within‐study bias and imprecision)	9 (2 to 25)	1%
TXA given orally at a total dose of 2 g pre‐incision	0.33 (0.05 to 2.12)	150 per 1000	50 per 1000	100 fewer per 1000 (143 fewer to 168 more per 1000)	⊕⊕⊝⊝ Low (due to imprecision)	15 (2 to 29)	1%
TXA given intra‐articularly at a total dose of 1 g intraoperatively	0.16 (0.04 to 0.58)	150 per 1000	24 per 1000	126 fewer per 1000 (144 fewer to 63 fewer per 1000)	Low (due to within‐study bias)	8 (2 to 23)	0%
TXA given intravenously at a total dose of greater than 3 g intraoperatively and postoperatively	0.34 (0.1 to 1.19)	150 per 1000	51 per 1000	99 fewer per 1000 (135 fewer to 29 more per 1000)	⊕⊕⊕⊝ Moderate (due to imprecision and heterogeneity)	15 (4 to 28)	0%
TXA given intravenously and intra‐articularly at a total dose of 2 g pre‐incision and intraoperatively	0.36 (0.1 to 1.26)	150 per 1000	54 per 1000	96 fewer per 1000 (135 fewer to 39 more per 1000)	⊕⊝⊝⊝ Very low (due to within‐study bias and imprecision)	17 (5 to 28)	0%
TXA given orally at a total dose of 2 g pre‐incision and postoperatively	0.29 (0.1 to 0.84)	150 per 1000	44 per 1000	106 fewer per 1000 (135 fewer to 24 fewer per 1000)	⊕⊕⊝⊝ Low (due to within‐study bias, imprecision and heterogeneity)	14 (5 to 25)	0%
TXA given intravenously at a total dose of 2 g pre‐incision and intraoperatively	0.42 (0.12 to 1.43)	150 per 1000	63 per 1000	87 fewer per 1000 (132 fewer to 65 more per 1000)	⊕⊝⊝⊝ Very low (due to within‐study bias and imprecision)	19 (6 to 28)	0%
TXA given intravenously at a total dose of 1 g pre‐incision and intraoperatively	0.29 (0.11 to 0.78)	150 per 1000	44 per 1000	106 fewer per 1000 (134 fewer to 33 fewer per 1000)	⊕⊕⊕⊝ Moderate (due to within‐study bias and heterogeneity)	14 (5 to 24)	0%
Aprotinin given intravenously	0.59 (0.36 to 0.96)	150 per 1000	89 per 1000	61 fewer per 1000 (96 fewer to 6 fewer per 1000)	⊕⊕⊝⊝ Low (due to within‐study bias and heterogeneity)	23 (15 to 27)	0%
Desmopressin given intravenously	1.41 (0.23 to 8.53)	150 per 1000	212 per 1000	62 more per 1000 (116 fewer to 1130 more per 1000)	⊕⊕⊝⊝ Low (due to imprecision)	28 (12 to 29)	0%
EACA given intravenously	0.6 (0.29 to 1.27)	150 per 1000	90 per 1000	60 fewer per 1000 (107 fewer to 41 more per 1000)	⊕⊝⊝⊝ Very low (due to within‐study bias and imprecision)	23 (12 to 28)	0%
Fibrin (topical)	0.86 (0.25 to 2.93)	150 per 1000	129 per 1000	21 fewer per 1000 (113 fewer to 290 more per 1000)	⊕⊝⊝⊝ Very low (due to within‐study bias and imprecision)	26 (12 to 29)	0%
TXA given intravenously at a total dose of 1 g intraoperatively	0.37 (0.19 to 0.73)	150 per 1000	56 per 1000	94 fewer per 1000 (122 fewer to 41 fewer per 1000)	⊕⊕⊝⊝ Low (due to within‐study bias, imprecision and incoherence)	17 (9 to 24)	0%
TXA given intravenously at a total dose of 1 g pre‐incision	0.47 (0.31 to 0.73)	150 per 1000	71 per 1000	79 fewer per 1000 (104 fewer to 41 fewer per 1000)	⊕⊕⊕⊝ Moderate (due to within‐study bias and heterogeneity)	20 (14 to 25)	0%
TXA given intravenously at a total dose of 1 g pre‐incision, intraoperatively and postoperatively	0.7 (0.26 to 1.87)	150 per 1000	105 per 1000	45 fewer per 1000 (111 fewer to 131 more per 1000)	⊕⊕⊝⊝ Low (due to within‐study bias and imprecision)	25 (12 to 29)	0%
TXA given intravenously at a total dose of 2 g intraoperatively and postoperatively	0.32 (0.17 to 0.61)	150 per 1000	48 per 1000	102 fewer per 1000 (125 fewer to 59 fewer per 1000)	⊕⊕⊝⊝ Low (due to within‐study bias)	15 (7 to 24)	0%
TXA given intravenously at a total dose of 2 g pre‐incision and postoperatively	0.39 (0.19 to 0.77)	150 per 1000	59 per 1000	91 fewer per 1000 (122 fewer to 35 fewer per 1000)	⊕⊕⊕⊝ Moderate (due to within‐study bias and heterogeneity)	17 (9 to 25)	0%
TXA given intravenously at a total dose of 3 g intraoperatively and postoperatively	0.4 (0.17 to 0.91)	150 per 1000	60 per 1000	90 fewer per 1000 (125 fewer to 14 fewer per 1000)	⊕⊕⊕⊝ Moderate (due to within‐study bias and heterogeneity)	18 (8 to 26)	0%

CrI: credible interval; EACA: epsilon‐aminocaproic acid; TXA: tranexamic acid *Results are expressed as risk ratios with credible intervals as opposed to confidence intervals, since a Bayesian analysis has been conducted. **Anticipated absolute effect. The anticipated absolute effect compares two risks by calculating the difference between the risk in the intervention group and the risk in the control group. ***Median rank with empirical 95% confidence interval, based on SUCRA scores. The SUCRA score for rank n is the probability that the treatment ranks at least nth. ****Probability of treatment ranking first.

**1:**
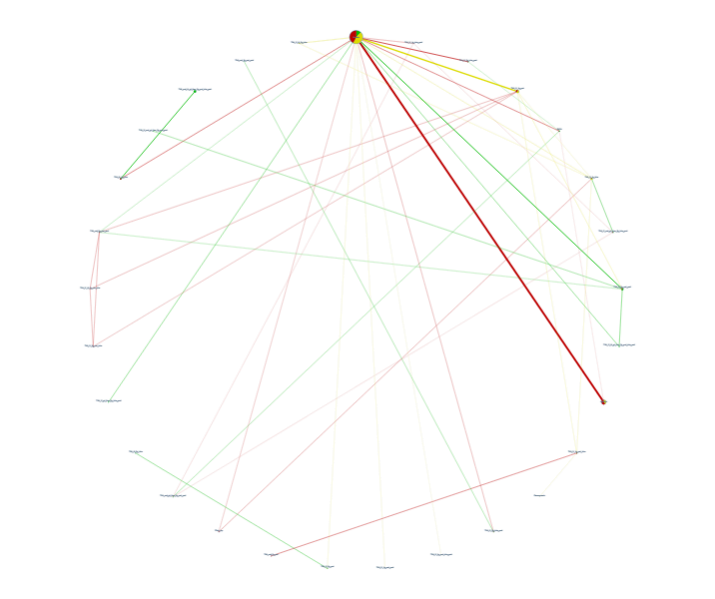
Network plot allogeneic blood transfusion

**1:** CINeMA grading for comparisons of intervention vs placebo (risk of allogeneic blood transfusion)

**Comparison**	**Number of studies**	**Within‐study bias**	**Reporting bias**	**Indirectness**	**Imprecision**	**Heterogeneity**	**Incoherence**	**Confidence rating**	**Reason(s) for downgrading**
**Mixed evidence**									
Aprotinin vs placebo	5	Some concerns	Some concerns	No concerns	No concerns	Major concerns	No concerns	Low	Within‐study bias (1 point), heterogeneity (1 point)
EACA vs placebo	3	Major concerns	Some concerns	No concerns	Major concerns	No concerns	No concerns	Very low	Within‐study bias (1 point), imprecision (2 points)
Fibrin topical vs placebo	1	Major concerns	Some concerns	No concerns	Major concerns	No concerns	No concerns	Very low	Within‐study bias (1 point), imprecision (2 points)
TXA_IA_1g_intra vs placebo	2	Major concerns	Some concerns	No concerns	No concerns	No concerns	No concerns	Low	Within‐study bias (2 points)
TXA_IV_1g_intra vs placebo	2	Some concerns	Some concerns	No concerns	No concerns	Some concerns	Major concerns	Low	Within‐study bias and heterogeneity (1 point), incoherence (1 point)
TXA_IV_1g_intra_post vs placebo	1	Major concerns	Some concerns	No concerns	Some concerns	Some concerns	No concerns	Low	Within‐study bias (1 point), imprecision (1 point)
TXA_IV_1g_preI vs placebo	7	Some concerns	Some concerns	No concerns	No concerns	Some concerns	No concerns	Moderate	Within‐study bias and heterogeneity (1 point)
TXA_IV_1g_preI_intra_post vs placebo	1	Some concerns	Some concerns	No concerns	Major concerns	No concerns	No concerns	Low	Within‐study bias (1 point), imprecision (1 point)
TXA_IV_1g_preI_post vs placebo	1	Some concerns	Some concerns	No concerns	Major concerns	No concerns	No concerns	Low	Within‐study bias (1 point), imprecision (1 point)
TXA_IV_2g_intra_post vs placebo	4	Major concerns	Some concerns	No concerns	No concerns	No concerns	No concerns	Low	Within‐study bias (2 points)
TXA_IV_2g_preI vs placebo	1	Some concerns	Some concerns	No concerns	No concerns	No concerns	No concerns	Moderate	Within‐study bias (1 point)
TXA_IV_2g_preI_post vs placebo	4	Some concerns	Some concerns	No concerns	No concerns	Some concerns	No concerns	Moderate	Within‐study bias and heterogeneity (1 point)
TXA_IV_3g_intra_post vs placebo	2	Some concerns	Some concerns	No concerns	No concerns	Some concerns	No concerns	Moderate	Within‐study bias and heterogeneity (1 point)
TXA_IV_IA_2g_intra vs placebo	1	Some concerns	Some concerns	No concerns	No concerns	No concerns	No concerns	Moderate	Within‐study bias (1 point)
TXA_IV_IA_grt_than_3g_preI_intra_post vs placebo	1	No concerns	Some concerns	No concerns	Major concerns	No concerns	No concerns	Low	Imprecision (2 points)
TXA_IV_grt_than_3g_intra_post vs placebo	1	No concerns	Some concerns	No concerns	Some concerns	Some concerns	No concerns	Moderate	Imprecision and heterogeneity (1 point)
TXA_oral_2g_preI_post vs placebo	1	Major concerns	Some concerns	No concerns	Some concerns	Some concerns	No concerns	Low	Within‐study bias (1 point), imprecision and heterogeneity (1 point)
**Indirect evidence**									
Desmopressin vs placebo	0	Some concerns	Some concerns	No concerns	Major concerns	No concerns	No concerns	Low	Imprecision (2 points)
TXA_IA_2g_intra vs placebo	0	No concerns	Some concerns	No concerns	Major concerns	No concerns	No concerns	Low	Imprecision (2 points)
TXA_IV_1g_preI_intra vs placebo	0	Some concerns	Some concerns	No concerns	No concerns	Some concerns	No concerns	Moderate	Within‐study bias and heterogeneity (1 point)
TXA_IV_2g_preI_intra vs placebo	0	Major concerns	Some concerns	No concerns	Major concerns	No concerns	No concerns	Very low	Within‐study bias (1 point), imprecision (2 points)
TXA_IV_IA_2g_preI_intra vs placebo	0	Major concerns	Some concerns	No concerns	Major concerns	No concerns	No concerns	Very low	Within‐study bias (1 point), imprecision (2 points)
TXA_IV_oral_grt_than_3g_intra_post vs placebo	0	Major concerns	Some concerns	No concerns	Some concerns	Some concerns	No concerns	Low	Within‐study bias (1 point), heterogeneity (1 point)
TXA_IV_oral_grt_than_3g_preI_post vs placebo	0	No concerns	Some concerns	No concerns	Major concerns	No concerns	No concerns	Low	Imprecision (2 points)
TXA_oral_2g_preI vs placebo	0	Some concerns	Some concerns	No concerns	Major concerns	No concerns	No concerns	Low	Within‐study bias (1 point), imprecision (1 point)
TXA_oral_3g_preI_post vs placebo	0	No concerns	Some concerns	No concerns	Major concerns	No concerns	No concerns	Low	Imprecision (2 points)
TXA_oral_IA_grt_than_3g_preI_intra_post vs placebo	0	No concerns	Some concerns	No concerns	No concerns	No concerns	No concerns	Moderate	Reporting bias (1 point)
TXA_oral_grt_than_3g_preI_post vs placebo	0	Major concerns	Some concerns	No concerns	Major concerns	No concerns	No concerns	Very low	Within‐study bias (1 point), imprecision (2 points)

EACA: epsilon aminocaproic acid; grt_than_3g: greater than 3 g; IA: intra‐articular; intra: intraoperative dose; IV: intravenous; post: postoperative dose; preI: pre‐incision dose; top: topical; TXA: tranexamic acid

**Summary of findings 2:** Summary of findings: Risk of deep vein thrombosis (DVT) up to 90 days post‐surgery

**Estimates of effects, credible intervals and certainty of the evidence for the prevention of bleeding in hip and knee replacement patients**							
**Patient or population**: individuals undergoing planned hip or knee replacement surgery **Interventions**: antifibrinolytics (tranexamic acid, aprotinin) **Comparator (reference)**: placebo **Outcome**: risk of deep vein thrombosis within 90 days of surgery **Setting**: elective orthopaedic surgery (See Figure 2)							
**Total studies:** 19 **Total participants:** 2395	Relative effect* (95% CrI)	Anticipated absolute effect**			**Certainty of evidence** **(**Table 4)	Median nodal ranking (95% CrI)***	**Probability of ranking 1st (%)**
		***Without* intervention**	***With* intervention**	**Difference**			
TXA given intravenously and orally at a total dose of greater than 3 g intraoperatively and postoperatively	0.16 (0.02 to 1.43)	80 per 1000	13 per 1000	67 fewer per 1000 (78 fewer to 34 more per 1000)	⊕⊕⊝⊝ Low (due to imprecision)	3 (1 to 16)	26%
TXA given intravenously and intra‐articularly at a total dose of 2 g pre‐incision and intraoperatively	0.21 (0 to 9.12)	80 per 1000	17 per 1000	63 fewer per 1000 (80 fewer to 650 more per 1000)	⊕⊕⊝⊝ Low (due to imprecision)	5 (1 to 18)	17%
TXA given intravenously and intra‐articularly at a total dose of greater than 3 g pre‐incision, intraoperatively and postoperatively	0.13 (0.01 to 3.11)	80 per 1000	10 per 1000	70 fewer per 1000 (79 fewer to 169 more per 1000)	⊕⊕⊝⊝ Low (due to imprecision)	4 (1 to 17)	15%
TXA given intravenously at a total dose of greater than 3 g intraoperatively and postoperatively	0.29 (0.01 to 5.47)	80 per 1000	23 per 1000	57 fewer per 1000 (79 fewer to 358 more per 1000)	⊕⊕⊝⊝ Low (due to imprecision)	6 (1 to 18)	15%
TXA given intravenously and orally at a total dose of greater than 3 g pre‐incision and postoperatively	0.27 (0.01 to 6.44)	80 per 1000	22 per 1000	58 fewer per 1000 (79 fewer to 435 more per 1000)	⊕⊕⊝⊝ Low (due to imprecision)	7 (1 to 18)	6%
TXA given intravenously at a total dose of 3 g intraoperatively and postoperatively	0.56 (0.07 to 4.73)	80 per 1000	45 per 1000	35 fewer per 1000 (74 fewer to 298 more per 1000)	⊕⊕⊝⊝ Low (due to imprecision)	9 (1 to 18)	5%
TXA given intra‐articularly at a total dose of 2 g intraoperatively	0.35 (0.09 to 1.45)	80 per 1000	28 per 1000	52 fewer per 1000 (73 fewer to 36 more per 1000)	⊕⊕⊝⊝ Low (due to imprecision)	6 (1 to 16)	5%
TXA given orally and intra‐articularly at a total dose of greater than 3 g pre‐incision, intraoperatively and postoperatively	0.9 (0.05, 15.45)	80 per 1000	72 per 1000	8 fewer per 1000 (76 fewer to 920 more per 1000)	⊕⊕⊝⊝ Low (due to imprecision)	11 (1 to 18)	4%
TXA given intravenously at a total dose of 2 g pre‐incision and postoperatively	0.19 (0.01 to 2.91)	80 per 1000	15 per 1000	65 fewer per 1000 (79 fewer to 153 more per 1000)	⊕⊕⊝⊝ Low (due to imprecision)	6 (1 to 17)	3%
TXA given intravenously at a total dose of 1 g postoperatively	0.75 (0.13 to 4.47)	80 per 1000	60 per 1000	20 fewer per 1000 (70 fewer to 278 more per 1000)	⊕⊕⊝⊝ Low (due to imprecision)	11 (2 to 18)	2%
TXA given intravenously at a total dose of 2 g postoperatively	1.02 (0.2 to 5.22)	80 per 1000	82 per 1000	2 more per 1000 (64 fewer to 338 more per 1000)	⊕⊕⊝⊝ Low (due to imprecision)	13 (3 to 18)	1%
TXA given intravenously at a total dose of 2 g intraoperatively and postoperatively	0.77 (0.27 to 2.16)	80 per 1000	62 per 1000	18 fewer per 1000 (58 fewer to 93 more per 1000)	⊕⊝⊝⊝ Very low (due to imprecision and within‐study bias)	11 (3 to 18)	0%
Aprotinin given intravenously	0.67 (0.28 to 1.62)	80 per 1000	54 per 1000	26 fewer per 1000 (58 fewer to 50 more per 1000)	⊕⊝⊝⊝ Very low (due to imprecision and within‐study bias)	10 (3 to 17)	0%
TXA given intra‐articularly at a total dose of 1 g intraoperatively	0.77 (0.09 to 6.48)	80 per 1000	62 per 1000	18 fewer per 1000 (73 fewer to 438 more per 1000)	⊕⊕⊝⊝ Low (due to imprecision)	11 (2 to 17)	0%
TXA given intravenously at a total dose of 1 g pre‐incision	0.73 (0.3 to 1.76)	80 per 1000	58 per 1000	22 fewer per 1000 (56 fewer to 61 more per 1000)	⊕⊕⊝⊝ Low (due to imprecision)	11 (5 to 17)	0%
TXA given intravenously at a total dose of 1 g pre‐incision and intraoperatively	0.83 (0.35 to 1.97)	80 per 1000	66 per 1000	14 fewer per 1000 (52 fewer to 78 more per 1000)	⊕⊕⊝⊝ Low (due to imprecision)	12 (5 to 18)	0%
TXA given intravenously at a total dose of 1 g intraoperatively	0.76 (0.32 to 1.79)	80 per 1000	61 per 1000	19 fewer per 1000 (54 fewer to 63 more per 1000)	⊕⊕⊝⊝ Low (due to imprecision)	11 (4 to 17)	0%

CrI: credible interval; TXA: tranexamic acid *Results are expressed as risk ratios with credible intervals as opposed to confidence intervals, since a Bayesian analysis has been conducted. **Anticipated absolute effect. The anticipated absolute effect compares two risks by calculating the difference between the risk in the intervention group and the risk in the control group. ***Median rank with empirical 95% confidence interval, based on SUCRA scores. The SUCRA score for rank n is the probability that the treatment ranks at least nth. ****Probability of treatment ranking first.

**2:**
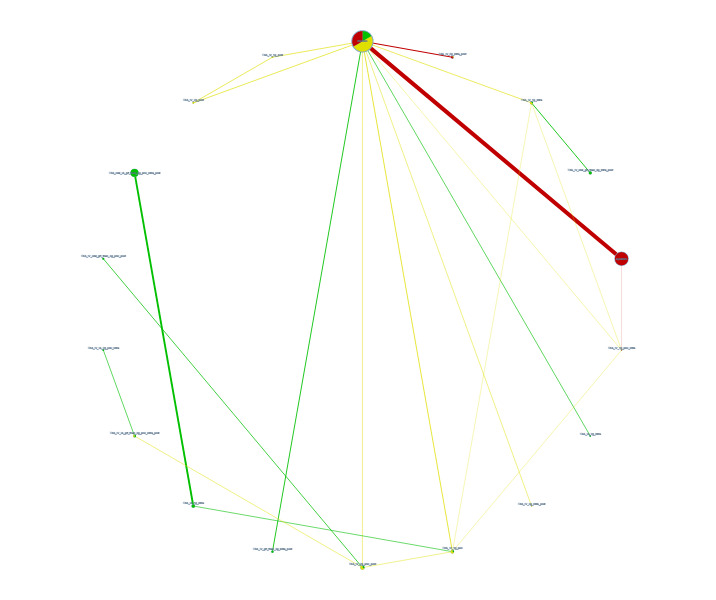
Network plot deep vein thrombosis

**2:** CINeMA grading for comparisons of intervention vs placebo (risk of deep vein thrombosis)

**Comparison**	**Number of studies**	**Within‐study bias**	**Reporting bias**	**Indirectness**	**Imprecision**	**Heterogeneity**	**Incoherence**	**Confidence rating**	**Reason(s) for downgrading**
**Mixed evidence**									
Aprotinin vs placebo	2	Major concerns	Some concerns	Low risk	Major concerns	No concerns	No concerns	Very low	Within‐study bias (1 point), imprecision (2 points)
TXA_IA_2g_intra vs placebo	1	No concerns	Some concerns	Low risk	Major concerns	No concerns	No concerns	Low	Imprecision (2 points)
TXA_IV_1g_intra vs placebo	2	Some concerns	Some concerns	Low risk	Major concerns	No concerns	No concerns	Low	Imprecision (2 points)
TXA_IV_1g_post vs placebo	1	Some concerns	Some concerns	Low risk	Major concerns	No concerns	No concerns	Low	Imprecision (2 points)
TXA_IV_1g_preI vs placebo	2	Some concerns	Some concerns	Low risk	Major concerns	No concerns	No concerns	Low	Imprecision (2 points)
TXA_IV_1g_preI_intra vs placebo	1	Some concerns	Some concerns	Low risk	Major concerns	No concerns	No concerns	Low	Imprecision (2 points)
TXA_IV_2g_intra_post vs placebo	3	Major concerns	Some concerns	Low risk	Major concerns	No concerns	No concerns	Very low	Within‐study bias (1 point), imprecision (2 points)
TXA_IV_2g_post vs placebo	1	Some concerns	Some concerns	Low risk	Major concerns	No concerns	No concerns	Low	Imprecision (2 points)
TXA_IV_2g_preI_post vs placebo	1	Some concerns	Some concerns	Low risk	Major concerns	No concerns	No concerns	Low	Imprecision (2 points)
TXA_IV_3g_intra_post vs placebo	1	Some concerns	Some concerns	Low risk	Major concerns	No concerns	No concerns	Low	Imprecision (2 points)
TXA_IV_grt_than_3g_intra_post vs placebo	1	No concerns	Some concerns	Low risk	Major concerns	No concerns	No concerns	Low	Imprecision (2 points)
**Indirect evidence**									
TXA_IA_1g_intra vs placebo	0	Some concerns	Some concerns	Low risk	Major concerns	No concerns	No concerns	Low	Imprecision (2 points)
TXA_IV_IA_2g_preI_intra vs placebo	0	Some concerns	Some concerns	Low risk	Major concerns	No concerns	No concerns	Low	Imprecision (2 points)
TXA_IV_IA_grt_than_3g_preI_intra_post vs placebo	0	Some concerns	Some concerns	Low risk	Major concerns	No concerns	No concerns	Low	Imprecision (2 points)
TXA_IV_oral_grt_than_3g_intra_post vs placebo	0	Some concerns	Some concerns	Low risk	Major concerns	No concerns	No concerns	Low	Imprecision (2 points)
TXA_IV_oral_grt_than_3g_preI_post vs placebo	0	Some concerns	Some concerns	Low risk	Major concerns	No concerns	No concerns	Low	Imprecision (2 points)
TXA_oral_IA_grt_than_3g_preI_intra_post vs placebo	0	No concerns	Some concerns	Low risk	Major concerns	No concerns	No concerns	Low	Imprecision (2 points)

grt_than_3g: greater than 3 g; IA: intra‐articular; intra: intraoperative dose; IV: intravenous; post: postoperative dose; preI: pre‐incision dose; top: topical; TXA: tranexamic acid

## Background

### Description of the condition

Musculoskeletal conditions such as osteoarthritis represent a major international public health challenge. Osteoarthritis affecting the hip or knee was reported as being the 11th highest contributor to global disability in the Global Burden of Disease Study (Cross 2014).

Hip or knee replacement surgery is a well‐established means of improving quality of life and offers effective pain relief, as well as restoration of function in people suffering from hip or knee disease. Data from the National Joint Registry in the UK demonstrate that 85.6% of people having hip replacement surgery and 70.8% of people having knee replacement surgery report being ‘much better’ following their surgery (NJR 2017a; NJR 2017b).

Internationally, the number of total hip replacements is increasing. In a study across 20 OECD (Organisation for Economic Co‐operation and Development) countries, the annual growth rate of hip replacement surgery is projected to rise from 1.8 million hip replacements per year in 2015 to 2.8 million per year in 2050. The mean incidence of hip replacement is expected to increase from 184 per 100,000 population to 275 per 100,000 population (Pabinger 2018). In 2015, the incidence of knee replacements was 150 per 100,000 population; it is anticipated that this figure will increase four‐fold by the year 2030 (Pabinger 2015).

Despite the benefits, hip or knee replacement surgery is associated with significant risk. In the UK, mortality from primary hip replacement within 90 days of surgery ranges from 0.2% in younger people, to 3.1% in older people, with even higher risk following revision surgery (NJR 2018). It is estimated that one‐third of people undergoing primary joint replacement are anaemic preoperatively (Munoz 2017). Hip or knee surgery can result in significant blood loss and up to 90% of patients are anaemic following surgery (Lasocki 2015; Park 2013). For revision surgery, the prevalence of preoperative anaemia and the average blood loss may be even greater (Palmer 2020). The increased prevalence of preoperative anaemia amongst people undergoing revision surgery is probably because the people who require revision surgery are older, and so more likely to suffer with chronic diseases and to be malnourished, all of which are factors that contribute to anaemia (Clevenger 2015).

As a consequence, people undergoing orthopaedic surgery receive 3.9% of all packed red blood cell transfusions in the UK and, of those, hip or knee replacement surgery uses 77% (Tinegate 2016). Bleeding and the need for allogeneic blood transfusions (donated blood from other people) has been shown to increase the risk of surgical site infection and mortality (Kim 2017). In addition, it is associated with an increased duration of hospital stay, and increased costs associated with surgery (Monsef 2014; Stokes 2011).

Prevention of bleeding during surgery offers the opportunity to reduce the risk of allogeneic blood transfusion, reduce cost and improve patients' outcomes following surgery. Several interventions are available and are currently employed as part of routine clinical care. These interventions include pharmacological therapies that have been proven to reduce blood loss from surgery (Li 2016; Schulman 2012).

### Description of the intervention

There are many pharmacological interventions that can be administered to reduce bleeding during surgery (Schulman 2012). This review focuses on several interventions including antifibrinolytics, desmopressin, factor VIIa and factor XIII, fibrinogen and sealants. Antifibrinolytics include tranexamic acid, aprotinin and epsilon‐aminocaproic acid. Tranexamic acid and epsilon‐aminocaproic acid are synthetic derivatives of the amino acid lysine and aprotinin is a non‐specific serine protease inhibitor derived from bovine lung. Antifibrinolytics are widely used in cardiac surgery to prevent bleeding (Henry 2011). Sealants can be grouped into fibrin containing sealants and non‐fibrin containing sealants. Fibrin sealants are composed of blood clotting agents and are applied to the wound to reduce blood loss; they have been found to be most effective when used in orthopaedic surgery (Carless 2003). Non‐fibrin sealants tend to function through mechanical expansion and prevent bleeding in a similar way to the application of pressure to a wound (Baird 2015). These interventions provide an advantage over blood transfusion through a reduction in the risk of the infective and compatibility complications associated with blood transfusion. In addition, there is a greater availability of pharmacological interventions than of blood transfusions. Finally, pharmacological interventions are versatile; they can be administered in a variety of different ways, including intravenously, orally, topically and nasally (see Appendix 1).

### How the intervention might work

When blood loss from hip or knee surgery results in a haemoglobin level below a certain threshold and the onset of associated symptoms, patients are often transfused with red blood cells, even though this procedure is associated with significant risk. All of the interventions described above aim to reduce bleeding and minimise blood loss. Each intervention and its mode of action, along with any limitations or potential risks, is described below.

#### Antifibrinolytics (tranexamic acid, aprotinin and epsilon‐aminocaproic acid)

During surgery, the clotting mechanism is activated. Antifibrinolytic drugs block the process of blood clot breakdown (fibrinolysis), therefore increasing clot strength and stability, which prevents excessive bleeding (Okamoto 1997). The most commonly used antifibrinolytic agents include tranexamic acid, aprotinin and epsilon‐aminocaproic acid (Henry 2011). In the UK, tranexamic acid is used in 42% of planned surgical cases (NCABT 2017). These medicines may be given orally, intravenous or topically (BNF 2022). Most have few side effects, however there is a theoretical increased risk of venous thromboembolism with their use (Levy 2018; Myers 2019).

#### Desmopressin

Desmopressin functions as a vasopressin analogue that increases the levels of von Willebrand factor and factor VIII (Pearson 2016). Von Willebrand factor and factor VIII enable platelets to adhere to wound sites and form clots to prevent bleeding. Desmopressin may be administered intravenously, subcutaneously or intranasally (BNF 2022). Side effects include facial flushing and possibly low blood sodium levels, especially with repeated doses (Desborough 2017a; Desborough 2017b).

#### Recombinant factor VIIa and factor XIII

Recombinant factor VIIa (rFVIIa) is an intervention licensed for use in people with haemophilia, congenital factor VII deficiency and inhibitory alloantibodies. However, it has also been used off‐license to prevent bleeding in surgery where the potential for blood loss is expected to be high (Simpson 2012). Despite its use, the efficacy of the drug in people without haemophilia remains uncertain. Factor XIII protects a developing clot from fibrinolysis and improves clot strength. Recombinant factor XIII (rFXIII) has been shown to mediate clot formation in a dose‐dependent manner, and it has been suggested that maintaining higher levels of rFXIII levels may prevent bleeding (Aleman 2014).

#### Fibrinogen

Fibrinogen concentrate is a blood component that is administered intravenously. Fibrinogen is converted to fibrin by thrombin and forms the structural basis of a clot. As it is derived from blood, there is a small risk of viral infection with its use, however due to its manufacturing process this is unlikely to result in infection (Franchini 2012).

#### Fibrin sealants

Fibrin sealants are derived from plasma and may be applied to actively bleeding bony surfaces or the wound. They usually consist of fibrinogen, thrombin, factor XIII, an antifibrinolytic agent and calcium chloride. However, some sealants do not contain an antifibrinolytic agent (Fischer 2011). Allergy is a rare complication (Aguilera 2013). Although fibrin sealants are derived from blood plasma, they have a lower risk of transmitting infections than allogeneic blood transfusions (Carless 2003).

#### Non‐fibrin sealants

Non‐fibrin sealants tend to be low‐viscosity liquids that polymerise to form a film that enables platelet activation and aggregation. This allows a clot to form, but relies on the patient’s own fibrin to create the clot. Other forms of non‐fibrin sealants include dressings, powders or bandages. Non‐fibrin sealants may enable clot formation where the use of a tourniquet is impractical. Adverse events that have been reported with their use are either associated with expansion of the sealant, e.g. nerve compression, or are the result of allergy (Baird 2015).

### Why it is important to do this review

A key objective for global health agencies such as the World Health Organization (WHO) is to ensure that every country is able to provide universal access to safe and adequate blood supplies to help save lives (WHO Factsheet 2017). Undertaking unnecessary transfusions and using unsafe transfusion practices can expose people to transfusion‐transmitted infections and serious adverse transfusion reactions, as well as consuming blood products that could be better used in those who are in need (WHO Factsheet 2017). This review will focus on the question of which pharmacological bleeding prevention treatment is most effective at preventing blood transfusion and blood loss. Bleeding and the need for blood transfusion may lead to costly adverse events such as infections and increased length of hospital stay (Monsef 2014; Stokes 2011). Reducing the number of blood transfusions is important to reduce these risks and to help preserve an already limited resource. Saving blood by reducing bleeding during surgery through pharmacological interventions may offer a lower‐risk option and will be cheaper than transfusing blood. For example, an ampoule of tranexamic acid or desmopressin costs approximately GBP 1.50 (BNF 2022), whereas one unit of red blood cells costs GBP 153.30, an increase of GBP 24.30 since 2019 (NHSBT).

To date, audits in orthopaedic hip or knee surgery suggest that there is still limited use of alternatives to allogeneic blood transfusion (NCABT 2017). In addition, there is some concern around using pharmacological interventions such as tranexamic acid due to a theoretical risk of unwanted blood clots, such as deep vein thrombosis or pulmonary embolism (blood clots in the lungs, which can affect breathing). In other populations, the timing of the dose has been shown to be of importance when considering adverse events. In the CRASH‐2 trial (a large multicentre international trial of tranexamic acid versus placebo), patients with significant bleeding from trauma had an increased risk of mortality if tranexamic acid was given after three hours (Roberts 2013). The dose of the intervention is also important from a cost perspective, as well as minimising the side‐effect profile of the agent. Safety concerns in people at increased risk of stroke or myocardial infarction have led to limited use of alternative interventions (Danninger 2015). In addition, topical alternatives may aid haemostasis while reducing systemic exposure to the treatment (Xu 2019a).

At protocol stage (Gibbs 2023), we anticipated that it would be unlikely that we would identify any trials that compared timing, dose and route of all these interventions directly, and that this would lead to uncertainty for decision‐makers. Therefore, in order to lessen this uncertainty and provide the highest level of evidence for treatment decisions in those undergoing orthopaedic surgery, we planned a network meta‐analysis to synthesise direct and indirect evidence to enable the evaluation of different treatment strategies for the prevention of bleeding in hip or knee surgery.

#### Description of network meta‐analysis

We carried out a network meta‐analysis (NMA) to allow the comparison of more than two treatments (Lu 2004). The evidence for each comparison is represented within a network map where each treatment is represented by a node (vertex), with lines connecting treatments to be compared (Jansen 2011). We have used solid lines to represent ‘direct’ comparisons where the treatments in question have been compared in clinical trials. We have used absent lines to represent ‘indirect’ comparisons, and indicate there are no clinical trials that made that comparison (Bucher 1997; Jansen 2011).

We used the data from the ‘direct’ comparisons to infer and estimate the effects of the missing comparisons ‘indirectly’ (Caldwell 2005; Jansen 2011; Jansen 2013; Song 2003). By doing this, we were able to bridge gaps in the evidence by combining data from direct comparisons in clinical trials with missing comparison information in the network structure, enabling more precise estimates to be obtained by using data from across the network (Krahn 2013; Salanti 2014). We only included data in the network that was similar enough in terms of effect modifiers across all direct comparisons to draw robust conclusions (Jansen 2013).

We presented results in a tabular format specifying treatment and outcome, to enable clinical decision‐making (Hoaglin 2011; Jansen 2011; Sutton 2008; van der Valk 2009).

## Objectives

To determine the relative efficacy of pharmacological interventions for preventing blood loss in elective primary or revision hip or knee replacement, and to identify the optimal administration of interventions regarding timing, dose and route, using network meta‐analysis methodology.

## Methods

### Criteria for considering studies for this review

#### Types of studies

We included randomised controlled trials (RCTs). If the process of randomisation was unclear, we contacted the trial authors to obtain further information. If we were unable to contact the authors, we included the trial in the review and considered it to be at unclear risk of bias. To be eligible, trials had to compare at least one of the active interventions of interest versus placebo or versus another active treatment. We used both abstracts and full‐text publications if they reported adequate information about study design, participant characteristics and interventions.

We did not include quasi‐randomised trials (assigned to a treatment, procedure or intervention by methods that are not random) due to lack of proper randomisation.

We only included trials that had been prospectively registered, unless the final trial report was published before 2010. The decision to exclude unregistered (or retrospectively registered) trials was taken due to the evidence highlighting issues surrounding false data (Carlisle 2021; Roberts 2015) and has now become the policy of Cochrane Injuries (Broughton 2021; Cochrane policy). Prospective registration reduces the chance of publication bias, and has been compulsory for randomised controlled trials since 2005, suggesting that those that have not been registered (or were registered retrospectively) since then are less likely to be of high quality (Roberts 2015). We have used a cut‐off of 2010 as this allowed studies that commenced before the introduction of compulsory registration in 2005 to complete and publish.

#### Types of participants

We included any person who had undergone an elective hip or knee replacement or revision surgery. We included people who had total knee replacements, partial or unicondylar knee replacements, hip replacements, and revision hip or knee surgery. We excluded people with known bleeding disorders such as haemophilia. We placed no restrictions on ethnicity or gender.

If an eligible trial contained a mixed population of people, then we only used data contributed from our population of interest. If no subgroup data were given, and we were unable to contact the corresponding author to provide this information, at least 80% of the sample size had to be from our population of interest for the trial to be eligible for inclusion.

#### Types of interventions

We included trials that compared one or more of the following interventions:

- antifibrinolytics:
  - tranexamic acid;
  - aprotinin;
  - epsilon‐aminocaproic acid;
- desmopressin;
- factor VIIa and factor XIII;
- fibrinogen;
- fibrin sealants/glue (not including surface dressings);
- non‐fibrin sealants (not including surface dressings).

Drugs and treatments that are not listed above were not used in the NMA. Acceptable comparators included placebo or one of the active interventions listed above. We excluded trials that used standard of care as the comparator.

We considered interventions given at a range of threshold doses, and as single or multiple doses via intravenous, subcutaneous, intranasal, oral or topical routes of administration. We also considered the timing of the interventions.

#### Types of outcome measures

We assessed the relative hierarchy ranking of the interventions using the following outcome measures.

##### Primary outcomes

- Risk of an allogeneic blood transfusion (up to 30 days)
- All‐cause mortality (deaths occurring up to 30 days after the operation)

##### Secondary outcomes

- Mean number of transfusion episodes per person (up to 30 days)
- Re‐operation due to bleeding (within seven days)
- Length of hospital stay
- Adverse events:
  - Risk of thromboembolism (deep vein thrombosis, pulmonary embolism, myocardial infarction, stroke): within 30 days
  - Risk of transfusion reactions (acute): within 24 hours
  - Risk of suspected serious drug reactions: within 30 days

We collected quality of life and cost data reported in the included studies. We did not perform an analysis of quality of life data or a formal economic evaluation with the collected information.

### Search methods for identification of studies

The Systematic Review Initiative (SRI, Oxford, UK) Information Specialist (CD) formulated the search strategies in collaboration with Cochrane Injuries.

#### Electronic searches

##### Bibliographic databases

We developed a thorough and sensitive search strategy to search for RCTs and systematic reviews from database inception to 18 October 2022, in the following databases:

- the Cochrane Central Register of Controlled Trials (CENTRAL 2022, Issue 10), in the Cochrane Library;
- MEDLINE (Ovid; 1946 to 18 October 2022);
- Embase (Ovid; 1974 to 18 October 2022);
- CINAHL (EBSCO*host;* 1937 to 18 October 2022);
- Transfusion Evidence Library (Evidentia Publishing, 1950 to 18 October 2022) (www.transfusionevidencelibrary.com);
- ClinicalTrials.gov (www.clinicaltrials.gov);
- World Health Organization International Clinical Trials Registry Platform (ICTRP) (apps.who.int/trialsearch).

Search strategies developed specifically for this review consisted of index terms, text words and word variations for the concepts of population (hip and knee surgery) and intervention/comparator (pharmacological interventions for the prevention of bleeding). We combined our searches in MEDLINE, Embase and CINAHL with adaptations of the recommended Cochrane RCT filter (Lefebvre 2011), and of the SIGN systematic review filters (www.sign.ac.uk/search-filters.html). We did not limit searches by language, year of publication or publication type. Search strategies for all databases are presented in Appendix 2.

#### Searching other resources

To complement the database searches, we handsearched the reference lists of recent systematic reviews to identify additional trials potentially missed by the electronic searches, but also to ensure that we collected as much of the available evidence as possible. We contacted the corresponding authors of the reviews to determine whether they were aware of any further trials in this area. In addition, we contacted authors of ongoing trials to obtain any unpublished data. We also examined any relevant retraction statements and errata for the included studies.

### Data collection and analysis

We performed the review according to the methods stated in Chapter 7 of the *Cochrane Handbook for Systematic Reviews of Interventions* (Higgins 2011a). We summarised the direct (pairwise) evidence using Review Manager Web (RevMan Web) (Review Manager Web), and performed the network meta‐analysis using BUGSnet (BUGSnet).

#### Selection of studies

Independently, two review authors (VNG, RC) screened all titles and abstracts identified by the electronic searches for eligibility and they excluded any citations deemed irrelevant. Independently, these review authors (VNG, RC) screened the full texts of all potentially relevant trials for eligibility against the criteria set out in the protocol. We resolved disagreements through discussion or, if required, through consultation with a third review author (LJE). We requested information from trial authors when there was insufficient information from trial reports to make a decision about eligibility. We kept the records of the selection process, as well as details of our reasons for exclusion at the full‐text stage. These were used to populate a PRISMA flowchart to demonstrate the selection of studies (Moher 2009; Page 2021). We used colleagues or Cochrane resources such as Task Exchange for translation of articles written in languages that the review authors cannot read and we thank and acknowledge these colleagues.

#### Data extraction and management

Independently, VNG and RC undertook data extraction of the included trials, using standardised, piloted forms designed according to the methods described in the *Cochrane Handbook for Systematic Reviews of Interventions* (Higgins 2011a). The review authors were not blinded to institutions, authors or outcomes of the trials. Colleagues who provided translation of studies written in languages other than English also extracted data from these studies. We piloted the data extraction forms on a random sample of 10 included trials (split equally between the review authors) and made adjustments. If a trial was identified as relevant by one author but not by the other, the authors discussed the rationale behind their assessments. If a consensus was not reached between the two authors, LJE served as the arbitrator. We contacted the corresponding authors of included trials up to three times to request additional trial data. If no response was received within four weeks, we deemed the data unobtainable. If there was conflict over data sources, we gave preference to published data over unpublished, as published data have been through a peer review process.

There were a large number of possible combinations for each intervention and data synthesis was difficult to determine prior to data extraction. Taxonomy of interventions took place prior to outcome data extraction, with help from an external expert panel to create clinically meaningful groups ready for data analysis. The external panel consisted of two haematologists (blood specialist doctors), two orthopaedic surgeons (bone and joint specialist doctors) and two anaesthetists. The panel were blinded to the outcome data and were given information on the study design, types of studies included and intervention information. The panel recommended nodes consisting of intervention name, mode of administration, total dose of intervention and timing. For tranexamic acid, the panel recommended doses were grouped into 1 g, 2 g, 3 g and > 3 g, and for the interventions EACA, aprotinin and desmopressin they recommended grouping all the studies together, as they are likely to be weight‐adjusted doses. The panel recommended timing be subdivided into pre‐incision (prior to making a surgical incision), intraoperative, postoperative within six hours of surgery, and postoperative administered within 6 to 24 hours. Where a study reported weighted doses of the interventions (e.g. mg/kg), we converted these doses to a uniform dose. We used the average weight of the patient population of the country in which the study was conducted according to published literature (Walpole 2012).

We extracted the following information.

- **General information:** name of review author carrying out data extraction; date of when data extraction was done; study ID (and any other unique trial identifiers); surname and contact address of first author of included trial; citation of included trial; language of trial and details of any duplicate publications.
- **Trial information:** trial design ‐ type of RCT; location of trial; setting; sample size; duration of trial; power calculation; treatment arms; randomisation; inclusion and exclusion criteria; comparability of groups and length of follow‐up.
- **Characteristics of participants:** age; sex; ethnicity; breakdown of total numbers for those recruited, randomised and analysed; type of surgery; numbers lost to follow‐up; dropouts (percentage in each arm) with reasons; protocol violations and co‐morbidities.
- **Characteristics of interventions:** number of treatment arms; description of experimental arm(s); description of control arm; timing, dose and route of administration of intervention; and other differences between intervention arms.
- **Outcomes:** need for blood transfusion within 30 days postoperatively; number of units of red blood cells transfused; mortality due to any cause within 30 days postoperatively; proportion of participants requiring each type of transfusion; and adverse effects (transfusion reactions, thromboembolism and drug reactions), re‐operation due to bleeding and length of hospital stay. (We extracted exactly how ‘adverse effects’ and ‘serious adverse effects’ were defined in each study.)
- **Quality assessment:** allocation concealment; blinding (participants, personnel, outcome assessors); incomplete outcome data; selective outcome reporting; other sources of bias. (Blinding was not possible for some comparisons.)

We utilised arm‐level data rather than study‐level data from both abstracts and full‐text papers. We obtained maximal data by extracting data from all publications available but used one data extraction form per trial. We contacted the primary or corresponding author of a trial, study groups or companies for additional data, if insufficient information was provided in the trial reports.

We also collected and have presented data on costs reported in the included studies. Although this does not constitute a formal economic evaluation, it provides useful additional information that may be of value in a decision‐making context.

Three review authors (VNG, RC, CK) entered the data into RevMan 5 and cross‐checked entries for accuracy.

##### Data on potential risk modifiers

From every included trial we extracted data on the following characteristics, which may act as treatment risk modifiers.

- **Type of surgery(primary hip or knee replacement or hip or knee revision):** surgery may have an impact on allogeneic transfusion and mortality, as often revision joint surgery results in more blood loss than primary joint replacement (Kasivisvanathan 2016). This is likely due to revision surgery taking longer and being more complex than primary joint replacement.
- **Reason for surgery:** the indication for surgery may also affect blood loss during surgery as, although most primary replacements are performed for arthritis, people who have replacements performed for other reasons such as bony cancer, may bleed more due to the tumour being more vascular than normal bone (Kumar 2014).
- **Duration of surgery:** longer surgery is likely to result in more bleeding.
- **Incidence of preoperative anaemia:** people with anaemia have a higher risk of blood transfusion following surgery (Kasivisvanathan 2016; Park 2013).
- **Type of anaesthetic used (general or spinal):** general anaesthesia has been associated with increased risk of blood transfusion, which may be due to loss of maintenance of venous pressure when the anaesthetic agents are administered (Basques 2015).
- **Use of tourniquet (in knee replacement surgery):** tourniquet use may reduce intraoperative blood loss, however some studies suggest that this may not affect total blood loss (Zhang 2014).
- **Use of anticoagulation:** participants on anticoagulants are likely to bleed more.

#### Assessment of risk of bias in included studies

We performed quality assessment on all the included trials using the methods described in Chapter 8 of the *Cochrane Handbook for Systematic Reviews of Interventions* (Higgins 2011b). We used the Cochrane risk of bias tool (RoB tool) (Higgins 2016). We tested the RoB tool in a small, random sample of trials. Three review authors (VNG, RC, CK) independently assessed risk of bias for each trial to assign each a classification of high, low or unclear risk. We created a Characteristics of included studies table and outlined the judgement process. We compared the review authors’ statements and reached a consensus on the classification of risk of bias. If necessary, a third author (LJE) was consulted.

Using this information, we explored statistical heterogeneity in each study and performed a sensitivity analysis. We followed the Cochrane methods for assessing risk of bias and addressed the following domains:

- selection bias (random sequence generation and allocation concealment);
- reporting bias (selective reporting);
- attrition bias (incomplete outcome data);
- performance bias (blinding of participants, personnel and outcome assessors);
- detection bias (blinding of outcome assessment);
- other forms of bias.

We assigned each of the domains listed above a classification of risk:

- **low risk** ‐ if the criterion has been adequately fulfilled in the study;
- **high risk** ‐ if the criterion has not been fulfilled in the study;
- **unclear risk** ‐ if the study report does not provide enough information with which to reach a clear decision.

We resolved any conflicts through discussion between the review authors (VNG, RC, CK) or by involving another author (LJE).

If a publication stated that participants were randomised but the method of randomisation used was not described, we contacted the trial authors. If this information was unobtainable, then we included the trial and considered it to be at an 'unclear' risk of bias as per the Cochrane risk of bias tool (Higgins 2011b).

We included both abstracts and full‐text publications.

#### Measures of treatment effect

When extracting data for dichotomous outcomes (number of participants with at least one bleeding episode, number of participants with at least one severe or life‐threatening bleeding episode, mortality, proportion of participants needing an allogeneic blood transfusion, adverse events), we documented the number of events and number of participants in the intervention and control arms.

For continuous outcomes (number of units of allogeneic blood transfused per participant, length of hospital stay), we documented the mean, standard deviation and total number of participants in both the intervention and control arms. If only study‐level data were available we recorded the reported effect size and the associated standard error.

We presented direct treatment comparisons and grouped the comparisons by treatment nodes; we compared and produced the forest plots using RevMan (Review Manager Web). We produced these to provide transparency on all outcome data collected for all studies.

#### Unit of analysis issues

In pairwise meta‐analyses, we treated trials with multiple treatment group comparisons as individual, independent two‐arm studies. The placebo group acted as a node in the NMA, which helped with indirect analyses and formation of a hierarchy of interventions. In the NMA, we included all comparisons where there were sufficient data to do so. These trials were analysed appropriately to take into account the respective treatment effects. The NMA method accounted correctly for correlations in relative effects from trials with more than two arms. We performed our analyses using the participant as the unit of analysis.

In future updates, in the event that we include one or more cluster‐RCTs, we will follow the guidance in Chapter 23 of the *Cochrane Handbook for Systematic Reviews of Interventions* (Higgins 2022), using a method of generic inverse variance in RevMan. We will also carefully consider the potential risk of bias associated with the method of randomisation described.

#### Dealing with missing data

Where there were missing data from any study, we contacted a corresponding author, by email, to obtain missing data. We attempted to contact the authors, by email, up to a maximum of three times to obtain the information. If we were still unable to obtain the information, and where missing data were thought to introduce serious bias, we performed a sensitivity analysis to evaluate the impact of missing outcome data.

We recorded the number of participants lost to follow‐up in each trial. In trials that also included other populations, such as those undergoing non‐elective hip or knee replacement, we extracted data for the elective hip or knee subgroup.

Continuous outcomes are often reported as a median and a measure of spread, such as a range or interquartile range (IQR) when the distribution is skewed. We considered using an assumption of log‐normality to obtain estimates of mean and SD from medians and range/IQR. However, neither of our continuous outcomes (units of blood product transfused and length of hospital stay) are granular enough for this approach to be reasonable, and small sample sizes posed an additional problem where range was the only available measure of spread.

#### Assessment of heterogeneity

##### Assessment of clinical and methodological heterogeneity within treatment comparisons

If we deemed the data to be homogenous, we combined them and performed a meta‐analysis. We assessed whether clinical and methodological heterogeneity were present within each comparison by looking at trial and participant characteristics across all included trials within the nodes. If significant clinical and methodological heterogeneity were found within a particular comparison, which meant that a meta‐analysis could not be performed, or that the summary statistic could not be reported, we provided a descriptive summary.

**Network meta‐analysis**

An assumption underlying NMA is that effect modifiers are similarly distributed across comparisons in the network. That means that an effect modifier should be similar in AB and BC trials in order to obtain a valid AC estimate. Equivalent formulations of the transitivity assumption are presented in Salanti 2012. In order to verify this assumption, for each comparison we compiled a table of important trial and patient characteristics and visually inspected the similarity of factors we considered likely to modify treatment effect. We also assessed the inclusion and exclusion criteria of every trial in the network to ensure that patients, trial protocols, etc. were similar in those aspects that might modify the treatment effect.

In the NMA we assumed a common estimate for heterogeneity across our comparisons and estimated a total I^2^ value for the network. We assessed statistical heterogeneity in the entire network based on the magnitude of the heterogeneity variance parameter (Tau^2^), which was estimated from the NMA models. We performed a likelihood ratio test for the null hypothesis of no heterogeneity versus presence of heterogeneity. For pairwise meta‐analyses, we estimated different heterogeneity variances for each pairwise comparison. We calculated the heterogeneity within each pair using the I^2^ statistic and 95% CI (I^2^ > 50% indicating moderate heterogeneity), which describes the variability that cannot be due to random error. We planned to explore heterogeneity by performing subgroup meta‐regression, but this was not possible.

#### Assessment of reporting biases

At the protocol stage, we planned to explore the existence of small‐study effects in our pairwise meta‐analyses (when there were more than 10 studies) by producing funnel plots, and by using meta‐regression in our NMA. We deemed a P value below the threshold of 0.10 to be statistically significant. The association between study effect size and funnel plot asymmetry is affected by several factors. We assumed that a lack of studies in areas of non‐significance would be indicative of publication bias. In the event, we were unable to action these plans, due to insufficient numbers of studies in the pairwise analyses.

#### Data synthesis

##### Relative treatment effects

We performed a Bayesian NMA using the BUGSnet package in R (v1.1.0) with default priors, producing estimated treatment effects for each comparison along with 95% credible intervals (CrIs). For the network meta‐analysis, we grouped interventions into clinically meaningful groups during the first stage of the data extraction, and treated each group as a single node within the network analysis. The large number of tranexamic acid regimens were grouped by dose, route and timing. Two review authors (VNG and RC) entered the data into the software and cross‐checked for accuracy.

For NMA of binary outcomes, we excluded direct comparisons with zero events, or zero non‐events, in one or both arms. Zero event studies are highly likely to lead to numerical instability and lack of convergence as this can affect connectivity (Dias 2010; Dias 2018).

For both continuous and binary outcomes, we used a random‐effects consistency model with 1000 adaptations, 50,000 burn‐ins and 100,000 iterations to ensure good convergence, and compared this with the equivalent inconsistency model to check that the model assumptions were reasonable.

##### Relative treatment ranking

We performed a Bayesian NMA using the BUGSnet package in R (v1.1.0) with default priors, producing rankings based on Surface Under the Cumulative RAnking curve (SUCRA). Two review authors (VNG and RC) entered the data into the software and cross‐checked for accuracy.

#### Subgroup analysis and investigation of heterogeneity

##### Subgroup analysis

We were unable to perform any subgroup analyses due to a lack of data and the number of interventions (23) included within the NMA compared with the number of studies (43).

We had planned to perform subgroup analyses and network meta‐regression for each of the following variables in order to explain heterogeneity, inconsistency, or both.

- Participants with preoperative anaemia
- Type of surgery (hip or knee primary replacement or hip or knee revision)
- Type of anaesthesia (general or spinal)
- Duration of surgery
- Use of tourniquet in knee replacement surgery
- Reason for surgery
- Use of anticoagulation

##### Investigation of heterogeneity

We estimated heterogeneity within direct comparisons grouped into broadly clinically consistent groups. We assessed statistical heterogeneity using Tau^2^, Cochran's Q and the I^2^ statistic.

##### Assessment of transitivity and inconsistency

We reported event rates or means for each node in the networks for each outcome and compared the model fit of a random‐effects consistency model to a random‐effects inconsistency model to check for potential problems with the transitivity assumption.

#### Sensitivity analysis

If trials contained mixed populations (e.g. included those requiring trauma surgery), then we used data only from the elective hip and knee subgroups, if available. If no subgroup data were presented and the corresponding author was not contactable for the information, we specified that at least 80% of the sample size had to be from our population of interest for the trial to be included.

We planned to assess the strength of the overall results by performing sensitivity analyses excluding trials deemed to be at high risk of bias. In the event, the network was too fragile to allow sensitivity analysis; we considered risk of bias in our interpretation of results.

#### Summary of findings and assessment of the certainty of the evidence

We did not specify any information related to the summary of findings table in our protocol. We produced an NMA summary of findings table as recommended by Yepes‐Nuez et al (Yepes‐Nuñez 2019).

We used the CINeMA framework (Confidence in Network Meta‐Analysis) to evaluate the confidence of the evidence for the summary of findings table (Nikolakopoulou 2020). We used the CINeMA framework rather than GRADEpro (Schünemann 2022) to develop a summary of findings table as the CINeMA framework has been created specifically to assess confidence in the results of a network meta‐analysis where there are a large number of interventions.

We used the online CINeMA tool to assess confidence for each comparison within the network based on: within‐study bias, across‐studies bias, indirectness, imprecision, heterogeneity and incoherence. We provide justifications in the summary of findings table for any decisions made to downgrade the certainty of the evidence to aid the reader's understanding of the review. We also included the relative effect with 95% credible intervals, the anticipated absolute effect with 95% credible intervals, the median nodal ranking point with 95% credible intervals and the probability of intervention ranking first (%).

## Results

### Description of studies

See Characteristics of included studies; Characteristics of excluded studies; Characteristics of studies awaiting classification; Characteristics of ongoing studies.

#### Results of the search

Our search, conducted on 18 October 2022, retrieved 6889 records. After removing all duplicates, we screened 3493 records based on their titles and abstracts.

We excluded 2870 records that did not meet the prespecified inclusion criteria at title and abstract stage and following full‐text screening we excluded a further 241 studies (from 251 publications). We identified 102 eligible completed trials (from 158 publications), 30 ongoing trials (from 31 publications) and 166 trials (from 183 publications) awaiting assessment. For further details, see Characteristics of included studies; Characteristics of studies awaiting classification; Characteristics of ongoing studies.

The study flow diagram Figure 3 illustrates the study selection process according to PRISMA guidelines (Moher 2009).

**3:**
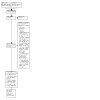
PRISMA diagram

#### Included studies

See Characteristics of included studies for full details of each trial.

#### Study selection

We included 102 RCTs in the review. For an overview of the studies included, see Table 5 and Table 6. TXA was the most common drug studied. The trials were mostly conducted in primary hip and knee replacement surgery. The most common route of administration studied was intravenous alone, followed by intra‐articular and then combined (intravenous and intra‐articular or oral and intra‐articular).

**3:** Overview of characteristics of included studies

**Study**	**Number of male participants**	**Number of female participants**	**Total number of participants**	**Outcomes reported**							
				**All‐cause mortality**	**Blood trans**	**Mean units**	**CVA**	**DVT**	**MI**	**PE**	**LOHS**
Alvarez 2008	17	78	95	N	Y	Y*	N	Y	N	Y	N
Alvarez 2019 hip	7	15	22	Y	Y	N	Y	Y	Y	Y	N
Alvarez 2019 knee	5	17	22	Y	Y	N	Y	Y	Y	Y	N
Benoni 1996	23	63	86	N	Y	Y*	N	Y	N	Y	N
Benoni 2000	17	22	39	N	Y	N	N	Y	N	Y	N
Benoni 2001	19	19	38	N	Y	Y*	Y	Y	Y	Y	N
Boese 2017	54	140	194	N	Y	Y*	Y	Y*	N	Y	Y
Bradley 2019 hip	42	48	90	N	Y	N	Y	Y	Y	Y	Y
Bradley 2019 knee	53	92	145	N	Y	N	Y	Y	Y	Y	Y
Camarasa 2006	25	102	127	N	Y	Y	Y	Y	Y	Y	N
Cao 2018	69	83	152	N	Y	Y*	Y	Y	Y	Y	N
Chang 2022	12	129	141	N	Y	N	N	Y	N	Y	N
Chin 2020	NR	NR	NR	N	Y	Y*	N	N	Y	Y	Y*
Claeys 2007	12	28	40	N	Y	Y*	N	Y	N	N	N
Clave 2019	98	131	229	N	Y	N	N	Y	Y	Y	Y
Colwell 2007	172	180	352	Y	Y	Y	Y	Y	Y	Y	N
Compostella 1997	NR	NR	NR	N	N	Y*	N	N	N	N	N
Cui 2019	35	37	72	N	Y	N	N	Y	N	Y	N
D'Ambrosio 1999	14	16	30	N	Y	Y*	N	N	N	N	N
Dorji 2021	20	11	31	N	Y	N	N	N	N	N	N
Ekback 2000	20	20	40	N	Y	Y*	N	Y	Y	N	N
Ellis 2001	11	29	40	N	Y	Y*	N	Y	N	Y	Y
Engel 2001	7	17	24	N	Y	Y*	N	Y	N	N	N
Flordal 1992	24	26	50	N	N	Y	N	N	N	N	N
Garcia Enguita 1998	NR	NR	NR	N	N	Y	N	N	N	N	N
Garneti 2004	NR	NR	NR	N	Y	Y	N	Y	N	Y	N
Georgiadis 2013	31	70	101	N	Y	Y*	N	Y	N	Y	Y
Gill 2009	3	7	10	N	Y	Y	N	Y	N	Y	Y*
Gomez Barrena 2014	27	51	78	Y	Y	Y*	N	Y	N	Y	Y
Gonzalez Osuna 2021	4	20	24	N	Y	Y	Y	Y	Y	Y	N
Good 2003	15	36	51	N	Y	Y*	N	Y	N	N	N
Goyal 2017	78	90	168	N	Y	Y*	N	Y	N	N	Y
Harley 2002	21	34	55	N	Y	Y*	N	Y	N	Y	N
Hayes 1996	15	27	42	N	N	Y	N	Y	N	N	N
Hiippala 1995	5	23	28	N	Y	Y	N	Y	Y	N	N
Hiippala 1997	12	65	77	Y	Y	Y	N	Y	Y	Y	N
Husted 2003	6	14	20	N	Y	Y*	N	Y	N	Y	N
Jansen 1999	8	34	42	N	Y	Y	N	Y	N	N	N
Janssens 1994	16	24	40	N	Y*	Y	N	Y	N	Y	Y
Jeserschek 2003	7	9	16	N	Y	Y	N	Y	N	N	N
Johansson 2005	53	47	100	N	Y	Y*	N	Y	N	Y	N
Jules‐Elysee 2019	31	32	63	Y	N	N	N	N	N	N	Y*
Kakar 2009 Bilateral TKR	14	36	50	N	N	Y*	N	Y	N	N	N
Kakar 2009 Unilateral TKR	14	36	50	N	N	Y*	N	Y	N	N	N
Kang 2021a	10	87	97	N	Y	Y*	N	Y	Y	Y	N
Kang 2021b	56	244	300	N	Y	Y*	N	Y	N	Y	N
Karnezis 1994 knee	16	20	36	Y	N	N	N	Y	Y	N	Y
Karnezis 1994 hip	26	30	56	Y	N	N	N	Y	Y	N	Y
Kayupov 2017a	24	47	71	N	Y	Y	N	Y	N	Y	Y
Kayupov 2017b	42	41	83	N	Y	N	N	Y	N	Y	Y
King 2019	26	27	53	N	Y	N	N	Y	N	N	Y*
Langdown 2000	NR	NR	NR	N	N	N	N	N	N	N	N
Lei 2017	27	132	159	N	Y	Y*	Y	Y	Y	Y	Y
Lei 2018	61	89	150	N	Y	Y*	Y	Y	Y	Y	Y
Lei 2020	26	124	150	N	Y	N	Y	Y	Y	Y	Y*
Lemay 2004	25	14	39	N	Y	N	N	Y	N	Y	N
Levine 2014	15	25	40	Y	Y	Y*	N	Y	N	Y	N
Llau 1998	NR	NR	NR	N	Y	Y*	N	Y	N	N	N
Lopez Picado 2017	57	51	108	N	Y	Y	N	Y	N	N	Y
Luo 2022	48	52	100	N	Y	N	Y	Y	Y	Y	Y
Molloy 2007	NR	NR	NR	Y	Y	Y	N	Y	N	Y	Y
Morales‐Avalos 2021	50	52	102	Y	Y	N	Y	Y	Y	Y	N
Murkin 1995	20	33	53	N	Y	Y	Y	Y	N	N	Y
Murkin 2000	139	141	280	Y	Y	N	N	Y	Y	N	N
NCT02922582	6	9	15	Y	Y	N	Y	N	N	N	N
Niskanen 2005	13	26	39	N	Y	Y*	N	Y	N	Y	Y*
North 2016	77	61	138	N	Y	N	N	Y	Y	Y	N
Orpen 2006	11	18	29	N	Y	N	N	Y	N	Y	N
Painter 2018	65	75	140	Y	Y	N	Y	Y	Y	Y	Y
Peng 2021	13	80	93	N	Y	N	Y	Y	Y	Y	N
Petsatodis 2006	NR	NR	NR	N	Y	Y	N	Y	N	N	N
Ray 2005	NR	NR	NR	N	Y	N	N	Y	N	Y	N
Schott 1995	35	44	79	N	N	Y	N	N	Y	Y	N
Sershon 2020	86	89	175	N	Y	N	Y	Y	N	Y	Y*
Staniforth 2017	NR	NR	NR	N	N	N	N	N	N	N	N
Stowers 2017	59	75	134	N	Y	Y*	N	Y	N	Y	Y
Tanaka 2001	31	68	99	N	Y	Y	N	Y	N	Y	N
Tsukada 2019	16	61	77	N	Y	N	N	Y	N	Y	N
Tsukada 2020	23	77	100	N	Y	Y*	Y	Y	Y	Y	N
Utada 1997	3	18	21	N	N	Y	N	N	N	N	N
Veien 2002	5	25	30	N	Y	Y*	N	Y	N	Y	N
Veien 2005	14	17	31	N	Y	Y*	N	Y	N	Y	N
Vles 2020	NR	NR	NR	N	N	N	N	N	N	N	N
Wang 2018	61	139	200	N	Y	N	Y	Y	Y	Y	Y
Wang 2019b	59	141	200	Y	Y	N	Y	Y	N	Y	Y*
Wang 2019c	112	188	300	Y	Y	N	Y	Y	Y	Y	Y*
Wang 2019a	26	92	118	Y	Y	N	N	Y	N	N	Y*
Wu 2018	59	41	100	N	Y	Y*	N	Y	N	Y	Y
Xie 2016	41	110	151	N	Y	Y*	Y	Y	Y	Y	Y
Xie 2017	60	90	150	N	Y	N	Y	Y	Y	Y	Y
Xu 2023	23	109	162**	Y	Y	Y	Y	Y	Y	Y	N
Xue 2021	38	118	156	N	Y	Y	Y	Y	Y	Y	N
Yamasaki 2004	37	3	40	N	Y	Y*	N	Y	N	Y	N
Yang 2020	24	70	94	N	Y	N	N	Y	N	Y	N
Yasli 2019	40	20	60	N	N	N	N	Y	N	Y	Y
Yen 2017	23	70	93	Y	Y	N	N	Y	N	Y	Y
Yen 2021	15	88	103	Y	Y	Y*	Y	Y	N	Y	Y*
Zeng 2017	60	40	100	N	Y	Y*	N	Y	N	Y	Y
Zeng 2018	23	37	60	N	Y	Y*	N	Y	N	Y	Y
Zhang 2007	NR	NR	NR	N	Y	Y	N	Y	N	N	N
Zhao 2018	70	50	120	N	Y	Y*	N	Y	N	Y	Y
Zohar 2004	18	42	60	N	Y	Y	N	Y	N	Y	Y

Y*: Represents where data were not included in the analysis due to being either incomplete or unusable (for example, due to differing units or inability to transform data to mean and SD). **: The numbers of males and females in Xu 2023 do not add up to the total number of patients. The author has been emailed for clarification but no response as yet (17 January 2023). CVA: cerebrovascular accident; DVT: deep vein thrombosis; LOHS: length of hospital stay; MI: myocardial infarction; N: no; NR: not reported; PE: pulmonary embolism; trans: transfusion; Y: yes

**4:** Baseline characteristics for included studies

***Study population***	***Frequency***	***%***
Primary THA	46	45
Primary TKA	43	42
Mixed primary THA and TKA	1	1
Revision THA	4	4
Mixed revision THA and TKA	2	2
Bilateral TKA	1	1
Mixed primary and bilateral TKA	1	1
Mixed primary and revision THA and TKA	4	4
***RCT origin***	***Number of studies***	
Europe	35	34
Asia	37	36
North America	21	21
Australia	6	6
South America	1	1
New Zealand	2	2
***Routes of interventions***	***Study arms***	
IV	170	71
IA	23	10
IV and IA	17	7
Oral and IA	12	5
Oral	11	5
IV and oral	5	2
IV, IA and oral	1	<1
***Drug type***	***Study arms***	
Tranexamic acid	150	83
Aprotinin	17	10
EACA	7	4
Desmopressin	5	3
Fibrin	2	1

EACA: epsilon‐aminocaproic acid; IA: intra‐articular; IV: intravenous; RCT: randomised controlled trial; THA: total hip arthroplasty; TKA: total knee arthroplasty

#### Design

All included trials were RCTs. There were 12 multi‐arm studies included in the primary outcome NMA (risk of an allogeneic blood transfusion) (Stowers 2017, Camarasa 2006; Clave 2019; Lopez Picado 2017; Ray 2005; Sershon 2020; Tanaka 2001; Wang 2019c; Xu 2023; Xue 2021; Yen 2021; Zhao 2018) and 36 two‐arm studies.

#### Setting

The included trials were published between 1992 and 2022. Ten included studies were multicentre studies (Benoni 2001; Clave 2019; Colwell 2007; Johansson 2005; Lopez Picado 2017; Murkin 2000; NCT02922582; Painter 2018; Sershon 2020; Stowers 2017), 30 studies did not report this information (Alvarez 2019 hip; Alvarez 2019 knee; Benoni 1996; Benoni 2000; Claeys 2007; Compostella 1997; Ekback 2000; Engel 2001; Flordal 1992; Good 2003; Hayes 1996; Hiippala 1997; Husted 2003; Jansen 1999; Jeserschek 2003; Kakar 2009 Bilateral TKR; Kakar 2009 Unilateral TKR; Langdown 2000; Lemay 2004; Luo 2022; Molloy 2007; Murkin 1995; Petsatodis 2006; Schott 1995; Veien 2002; Veien 2005; Vles 2020; Yasli 2019; Zhang 2007; Zohar 2004), and the remaining 62 were single‐centre studies. One study was conducted across two countries (America and Canada) (Colwell 2007). We found a global spread of trials, with the highest number of trials being conducted in Europe and Asia (Table 6). Four studies included were translated into English for the review (Cui 2019; Utada 1997; Veien 2005; Zhang 2007).

#### Trial size

The number of participants enrolled in the included studies ranged from 16 (NCT02922582) to 300 (Wang 2019c). Power calculations were included in 47 studies (Alvarez 2019 hip; Benoni 2000; Benoni 2001; Camarasa 2006; Cao 2018; Chang 2022; Clave 2019; Dorji 2021; Gill 2009; Gomez Barrena 2014; Good 2003; Goyal 2017; Harley 2002; Husted 2003; Jeserschek 2003; Johansson 2005; Jules‐Elysee 2019; Kayupov 2017a; Kayupov 2017b; King 2019; Lei 2017; Lei 2018; Lei 2020; Lopez Picado 2017; Luo 2022; Molloy 2007; Morales‐Avalos 2021; Niskanen 2005; Painter 2018; Peng 2021; Sershon 2020; Stowers 2017; Tsukada 2019; Tsukada 2020; Wang 2018; Wang 2019a; Wang 2019b; Wang 2019c; Wu 2018; Xie 2016; Xie 2017; Xu 2023; Xue 2021; Yasli 2019; Yen 2017; Yen 2021; Zohar 2004). In one study it was unclear whether the investigators had achieved their target sample size (Hiippala 1995), and in 30 studies a sample size was not reported (Claeys 2007; Compostella 1997; D'Ambrosio 1999; Ekback 2000; Ellis 2001; Flordal 1992; Garcia Enguita 1998; Garneti 2004; Georgiadis 2013; Gonzalez Osuna 2021; Hayes 1996; Hiippala 1997; Jansen 1999; Janssens 1994; Kakar 2009 Bilateral TKR; Kakar 2009 Unilateral TKR; Karnezis 1994 hip; Karnezis 1994 knee; Langdown 2000; Llau 1998; Petsatodis 2006; Staniforth 2017; Tanaka 2001; Utada 1997; Veien 2005; Vles 2020; Yamasaki 2004; Zeng 2017; Zeng 2018; Zhao 2018). The remaining 24 studies did not achieve their target sample size.

#### Characteristics of participants

We summarised the characteristics of the participants in each trial in the Characteristics of included studies table and provided an overview of the characteristics of the included studies in Table 5. The mean age of participants in the included trials ranged from 50 to 77 years of age (Lei 2018; Tsukada 2019, respectively). Trials included more women (5388 (64%)) than men (3030 (36%)). (There is a discrepancy in the number of males and females in Xu 2023; the authors were contacted for clarification). Five studies reported ethnicity, and of those the majority of included participants were white (Caucasian) (Table 5).

#### Characteristics of outcomes reported

Twenty studies reported the primary outcome mortality within 30 days and 86 studies reported the primary outcome of risk of requiring a blood transfusion within 30 days. Sixty‐four studies reported the secondary outcome mean number of units transfused within 30 days. For adverse events, 28 studies reported on the outcome cerebrovascular event, 89 reported on the outcome deep vein thrombosis, 32 reported on the outcome myocardial infarction and 70 reported on the adverse outcome pulmonary embolism. There were 42 studies reporting on the length of hospital stay (Table 5).

#### Characteristics of interventions

The majority of the included studies examined tranexamic acid (150 arms, 83%). Aprotinin was the next most studied intervention (17 arms, 9%), followed by epsilon‐aminocaproic acid (EACA) (seven arms, 4%), desmopressin (five arms, 3%) and fibrin (two arms, 1%) (see Table 6).

#### Sources of support

In total, 81 studies declared a funding source and of those seven were funded by pharmaceutical companies (Clave 2019; Colwell 2007; Molloy 2007; Murkin 2000; NCT02922582; Niskanen 2005; Schott 1995), and five were reported as being partly funded via pharmaceutical companies and partly non‐pharmaceutical (Benoni 2001; Ekback 2000; Gomez Barrena 2014; Hayes 1996; Jansen 1999).

Twenty‐one studies did not report a funding source (Chang 2022; Claeys 2007; Compostella 1997; Ellis 2001; Engel 2001; Georgiadis 2013; Harley 2002; Hiippala 1997; Husted 2003; Janssens 1994; Kakar 2009 Bilateral TKR; Kakar 2009 Unilateral TKR; Lei 2017; Lei 2018; Llau 1998; Orpen 2006; Veien 2002; Wang 2019a; Xue 2021; Zhang 2007; Zohar 2004).

#### Ongoing studies

We identified 30 ongoing studies (from 31 publications):

- 11 studies exploring TXA in elective knee surgery (ChiCTR1800016960; ChiCTR1800017038; ChiCTR1800018751; ChiCTR1800019261; ChiCTR2000035271; ChiCTR2000039368; ChiCTR‐INR‐16008762; ChiCTR‐IPC‐14005579; EUCTR‐2016‐000071‐24‐ES; NCT05099276; Yang 2021);
- 11 studies exploring TXA in elective hip surgery (ChiCTR1800017094; ChiCTR1800017095; ChiCTR1800018100; ChiCTR1900020498; ChiCTR2100045474; CTRI/2022/03/041001; EUCTR‐2020‐003321‐32‐DK; EUCTR‐2020‐004167‐29‐BE; NCT03623789; NCT03897621; NCT04691362);
- four studies exploring TXA in elective hip or knee surgery (ChiCTR‐INR‐16010030; ChiCTR‐INR‐17013711; EUCTR‐2018‐003537‐15; UMIN000047607);
- one study exploring bone wax in elective knee surgery (NCT04992052);
- one study exploring topical haemostatic drugs in elective knee surgery (ChiCTR‐IPR‐16008176);
- one study exploring topical haemostatic drugs in elective hip surgery (ChiCTR‐IPR‐16008175);
- one study exploring fibrinogen in elective hip surgery (EUCTR‐2008‐007110‐29‐FR).

#### Studies awaiting assessment

We identified 166 studies (from 183 publications) for which a decision on eligibility could not be made. Full details are provided in Characteristics of studies awaiting classification.

- Unable to find a trial registration to determine whether the trial was prospectively or retrospectively registered = 131 (Abdallah 2020; Adravanti 2018; Aggarwal 2016; Alipour 2013; Almeida 2018; Amin 2020; Anon 2016; Antinolfi 2014; Arora 2018; Arslan 2018; Bae 2014; Balasubramanian 2016; Bao 2019; Bidolegui 2014; Borisov 2011; Bowman 2018; Bradshaw 2012; Canata 2012; Cankaya 2017; Carvalho 2015; Castro‐Menendez 2016; Cavusoglu 2015; Chai 2015; Chen 2016a; Chen 2018; DiFrancesco 2013; Digas 2015; Falez 2013; Fleischmann 2011; Gautam 2011; Gautam 2013; Gomez Barbero 2019; Gulabi 2019; Guzel 2016; Hongshun 2019; Hou 2015; Hsu 2015; Hu 2018; Huang 2014; Imai 2012; Jain 2016; Jaszczyk 2015; Jia 2019; Karaaslan 2015; Kazemi 2010; Keyhani 2016; Kim 2014; Kundu 2015; Kusuma 2013; Lacko 2017; Lee 2013; Lee 2013a; Lee 2017; Lee 2017a; Li‐Qing 2018; Liebelt 2013; Lin 2015; Liu 2018; Liu 2021; Lopez‐Hualda 2018; Luo 2018; Ma 2014; MacGillivray 2011; Malhotra 2011; Maniar 2012; May 2016; McDonald 2017; McDonald 2022; Mehta 2019; Min 2015; Moo 2017; Na 2016; Nambiar 2019; Ni 2016; Notarnicola 2012; Obaidur 2014; Oztas 2015; Pan 2019; Patel 2014; Patni 2012; Piolanti 2018; Prabhu 2015; Prakash 2018; Raviraj 2012; Rizzo 2020; Sarzaeem 2014; Seo 2013; Shen 2015; Shinde 2015; Singh Sidhu 2021; Soni 2014; Specchiulli 2011; Sun 2016; Sun 2017; Taheriazam 2018; Taheriazam 2019; Tang 2019; Triyudanto 2016; Tzatzairis 2019; Ugurlu 2017; Vandesande 2015; Volquind 2016; Wang 2015; Wang 2015a; Wang 2015b; Wang 2016; Wang 2017; Wei 2014; Wu 2016a; Wu 2018a; Wu 2019; Wu 2019a; Wu 2020; Wu 2020a; Xiaofei 2020; Xie 2016a; Xu 2016; Xu 2019b; Yang 2015; Ye 2019; Yue 2014; Zeng 2016; Zhang 2015; Zhang 2016; Zhang 2018; Zhang 2019; Zhang 2019a; Zhang 2020; Zhang 2020a; Zhang 2020b; Zhaohui 2014).
- Status on trial registry:
  - complete = 10 (ChiCTR‐IPR‐17012265; ChiCTR1800015834; CTRI/2018/05/013588; CTRI/2018/08/015421; CTRI/2019/09/021302; EUCTR‐2013‐003169‐33‐DK; NCT01391182; NCT02056444; NCT02117128; NCT03386656);
  - “not yet recruiting” = 12 (ChiCTR‐INR‐16010188; ChiCTR‐INR‐17010951; ChiCTR‐IOR‐15007198; ChiCTR‐IPR‐17011848; ChiCTR1900026092; ChiCTR1900027416; ChiCTR2000032271; CTRI/2018/02/012030; CTRI/2019/01/017105; CTRI/2021/09/036855; Lei 2020a; NCT03822793);
  - paper in submission = 1 (ChiCTR‐INR‐16010270);
  - recruitment complete = 2 (IRCT20120910010800N3; UMIN000029797);
  - unknown = 10 (NCT00983112; NCT01260818; NCT01527968; NCT02286973; NCT02438566; NCT02587845; NCT02938962; NCT03109652; NCT03310060; NCT0393407).

#### Excluded studies

After full‐text screening, we excluded 241 studies (within 251 publications) from the review. Full details are provided in the Characteristics of excluded studies with a summary of the reasons for exclusion below:

- 154 were retrospectively registered (see Characteristics of excluded studies);
- 29 were unregistered trials as confirmed by the author or stated in the text (Ahmed 2018; Bouali 2011; DeNapoli 2016; Drosos 2016; Hourlier 2014; Hourlier 2015; Karampinas 2019; Kyriakopoulos 2019; Laoruengthana 2019a; Leino 2010; Li 2020; Lo 2020; Lostak 2020; Lostak 2020a; Maniar 2017; Martin 2014; Mehta 2019a; Melo 2017; Morales Santias 2020; Oremus 2014; Palija 2021; Pavao 2019; Sa‐Ngasoongsong 2011; Sahin 2019; Shihab 2021; Tandogan 2021; Tavares Sanchez‐Monge 2018; Tripathy 2020; Vela 2012);
- 28 had an ineligible study design only confirmed at full‐text screening (study design unclear or not reported in abstract but could be an RCT) (ACTRN12617000617369; Aguilera 2012; Akgul 2016; Bali 2011; Benoni 1997; Cao 2015; Chen 2014; Cornell 2017; Dong 2017; Drosos 2016; Fernandez‐Collins 2017; Haas 1984; Hegde 2013; Iseki 2018; Ishida 2011; Ketterl 1982; Kim 2018; Lee 2017b; Lin 2011; Luo 2012; Mercuriali 2004; Munoz Gomez 2013; Ollivier 2016; Pachauri 2014; Perez‐Jimeno 2018; Prakash 2016; Seol 2016; Stutz 2004);
- 15 had an ineligible comparator, which was not discovered until full‐text screening (ChiCTR1800015839; Choufani 2015; Cvachovec 2011; Freick 1983; Kluba 2012; Kraft 1999; Mena 2002; NCT01410240; NTR6464 Netherlands Trials Register; Ruiz‐Moyano 1997; Suarez 2014; Thorpe 1994; Wang 2001; Wang 2003; Wollinsky 1991);
- seven had an ineligible patient population, which was not discovered until full‐text screening (Amar 2003; Capdevila 1998; ChiCTR‐TRC‐14004379; Lassen 2006; Samama 2002; Thipparampall 2017; Xu 2015);
- seven were withdrawn (ACTRN12613001043729; DRKS00007564; EUCTR‐2009‐012043‐42‐UK; NCT00375440; NCT00440921; NCT02553122; NCT02644473);
- one had an ineligible intervention, which was not discovered until full‐text screening (Rajesparan 2009).

### Risk of bias in included studies

For a visual representation of the assessments of risk of bias across all trials, see Figure 4 and Figure 5. For further information regarding bias detected in individual trials, see Characteristics of included studies.

**4:**
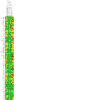
Risk of bias summary: review authors' judgements about each risk of bias item for each included study.

**5:**
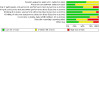


#### Random sequence generation (selection bias)

We considered no trial to be at high risk of bias and 39 trials to be at unclear risk of bias, as they did not provide sufficient information on sequence generation (Benoni 1996; Benoni 2000; Benoni 2001; Cao 2018; Claeys 2007; Compostella 1997; D'Ambrosio 1999; Ekback 2000; Engel 2001; Flordal 1992; Garcia Enguita 1998; Gomez Barrena 2014; Harley 2002; Hayes 1996; Hiippala 1995; Hiippala 1997; Janssens 1994; Jeserschek 2003; Kakar 2009 Bilateral TKR; Kakar 2009 Unilateral TKR; Lei 2017; Lei 2020; Levine 2014; Llau 1998; Murkin 2000; Niskanen 2005; North 2016; Orpen 2006; Petsatodis 2006; Ray 2005; Schott 1995; Staniforth 2017; Stowers 2017; Tanaka 2001; Utada 1997; Xie 2016; Xie 2017; Yang 2020; Zhang 2007).

We judged 63 trials to be at low risk of bias as they provided clear and detailed information on sequence generation (Alvarez 2008; Alvarez 2019 hip; Alvarez 2019 knee; Boese 2017; Bradley 2019 hip; Bradley 2019 knee; Camarasa 2006; Chang 2022; Chin 2020; Clave 2019; Colwell 2007; Cui 2019; Dorji 2021; Ellis 2001; Garneti 2004; Georgiadis 2013; Gill 2009; Gonzalez Osuna 2021; Good 2003; Goyal 2017; Husted 2003; Jansen 1999; Johansson 2005; Jules‐Elysee 2019; Kang 2021a; Kang 2021b; Karnezis 1994 hip; Karnezis 1994 knee; Kayupov 2017a; Kayupov 2017b; King 2019; Langdown 2000; Lei 2018; Lemay 2004; Lopez Picado 2017; Luo 2022; Molloy 2007; Morales‐Avalos 2021; Murkin 1995; NCT02922582; Painter 2018; Peng 2021; Sershon 2020; Tsukada 2019; Tsukada 2020; Veien 2002; Veien 2005; Vles 2020; Wang 2018; Wang 2019a; Wang 2019b; Wang 2019c; Wu 2018; Xu 2023; Xue 2021; Yamasaki 2004; Yasli 2019; Yen 2017; Yen 2021; Zeng 2017; Zeng 2018; Zhao 2018; Zohar 2004).

#### Allocation concealment (selection bias)

We considered no trial to be at high risk of bias and 74 trials to have unclear risk of bias due to lack of information on allocation concealment (Alvarez 2008; Alvarez 2019 hip; Alvarez 2019 knee; Benoni 1996; Benoni 2001; Bradley 2019 hip; Bradley 2019 knee; Camarasa 2006; Cao 2018; Claeys 2007; Compostella 1997; Cui 2019; D'Ambrosio 1999; Dorji 2021; Ekback 2000; Ellis 2001; Engel 2001; Flordal 1992; Garcia Enguita 1998; Goyal 2017; Harley 2002; Hayes 1996; Hiippala 1995; Hiippala 1997; Husted 2003; Jansen 1999; Janssens 1994; Jeserschek 2003; Jules‐Elysee 2019; Kakar 2009 Bilateral TKR; Kakar 2009 Unilateral TKR; Kang 2021b; Karnezis 1994 hip; Karnezis 1994 knee; Kayupov 2017a; Kayupov 2017b; King 2019; Langdown 2000; Lei 2017; Lei 2018; Lei 2020; Levine 2014; Llau 1998; Luo 2022; Molloy 2007; Morales‐Avalos 2021; Murkin 1995; NCT02922582; Niskanen 2005; Orpen 2006; Peng 2021; Petsatodis 2006; Ray 2005; Schott 1995; Sershon 2020; Staniforth 2017; Stowers 2017; Tanaka 2001; Tsukada 2019; Utada 1997; Veien 2002; Veien 2005; Vles 2020; Wu 2018; Xie 2016; Xie 2017; Yamasaki 2004; Yasli 2019; Zeng 2017; Zeng 2018; Zhang 2007; Zhao 2018; Zohar 2004).

We judged 28 trials to be at low risk of bias as they provided clear and detailed information on allocation concealment (Benoni 2000; Boese 2017; Chang 2022; Chin 2020; Clave 2019; Colwell 2007; Georgiadis 2013; Gill 2009; Gomez Barrena 2014; Gonzalez Osuna 2021; Good 2003; Johansson 2005; Kang 2021a; Levine 2014; Lopez Picado 2017; Murkin 2000; North 2016; Painter 2018; Tsukada 2020; Wang 2018; Wang 2019a; Wang 2019b; Wang 2019c; Xu 2023; Xue 2021; Yang 2020; Yen 2017; Yen 2021).

#### Blinding

To assess bias due to a lack of blinding, we separately assessed the risk for objective and subjective outcomes.

We considered objective outcomes to include: mortality, incidence of myocardial infarction (MI), cerebrovascular accident (CVA) or stroke, and pulmonary embolism (PE) due to the clear diagnostic criteria in wide use.

We deemed the remaining outcomes subjective: need for allogeneic blood transfusion, length of hospital stay, incidence of serious drug reactions and the incidence of deep vein thrombosis (DVT), due to the more subjective nature of a DVT diagnosis.

#### Blinding of participants and personnel (performance bias)

##### Subjective outcomes

We judged 18 studies to be at high risk of bias due to inadequate blinding (Alvarez 2008; Compostella 1997; Dorji 2021; Engel 2001; Gonzalez Osuna 2021; Goyal 2017; Kang 2021a; King 2019; Lei 2017; Molloy 2007; Murkin 2000; NCT02922582; Sershon 2020; Veien 2005; Xie 2017; Yang 2020; Yen 2021; Zohar 2004), 31 studies were judged to be at unclear risk of bias (Benoni 2000; Bradley 2019 hip; Bradley 2019 knee; Chang 2022; Claeys 2007; Cui 2019; D'Ambrosio 1999; Ekback 2000; Ellis 2001; Flordal 1992; Garcia Enguita 1998; Good 2003; Hayes 1996; Hiippala 1995; Husted 2003; Janssens 1994; Kang 2021b; Llau 1998; Luo 2022; Niskanen 2005; Petsatodis 2006; Ray 2005; Schott 1995; Utada 1997; Xu 2023; Xue 2021; Yamasaki 2004; Yasli 2019; Zeng 2017; Zeng 2018; Zhang 2007), and the remaining 53 studies are low risk of bias (Alvarez 2019 hip; Alvarez 2019 knee; Benoni 1996; Benoni 2001; Boese 2017; Camarasa 2006; Cao 2018; Chin 2020; Clave 2019; Colwell 2007; Garneti 2004; Georgiadis 2013; Gill 2009; Gomez Barrena 2014; Harley 2002; Hiippala 1997; Jansen 1999; Jeserschek 2003; Johansson 2005; Jules‐Elysee 2019; Kakar 2009 Bilateral TKR; Kakar 2009 Unilateral TKR; Karnezis 1994 hip; Karnezis 1994 knee; Kayupov 2017a; Kayupov 2017b; Langdown 2000; Lei 2018; Lei 2020; Lemay 2004; Levine 2014; Lopez Picado 2017; Morales‐Avalos 2021; Murkin 1995; North 2016; Orpen 2006; Painter 2018; Peng 2021; Staniforth 2017; Stowers 2017; Tanaka 2001; Tsukada 2019; Tsukada 2020; Veien 2002; Vles 2020; Wang 2018; Wang 2019a; Wang 2019b; Wang 2019c; Wu 2018; Xie 2016; Yen 2017; Zhao 2018).

##### Objective outcomes

We judged all trials to be at low risk of bias as we believed that the blinding would not affect the objective outcomes stated in this review.

#### Blinding of outcome assessment (detection bias)

##### Subjective outcomes

We judged eight studies to be at high risk of bias (Bradley 2019 hip; Bradley 2019 knee; Dorji 2021; Engel 2001; King 2019; NCT02922582; Sershon 2020; Veien 2002).

We assessed 43 studies as being at unclear risk of bias (Benoni 2000; Chang 2022; Chin 2020; Claeys 2007; Colwell 2007; Compostella 1997; Cui 2019; D'Ambrosio 1999; Ekback 2000; Flordal 1992; Garcia Enguita 1998; Garneti 2004; Gonzalez Osuna 2021; Good 2003; Hayes 1996; Hiippala 1995; Hiippala 1997; Husted 2003; Jansen 1999; Janssens 1994; Jeserschek 2003; Kang 2021b; Langdown 2000; Llau 1998; Luo 2022; Murkin 1995; Murkin 2000; Niskanen 2005; Petsatodis 2006; Ray 2005; Schott 1995; Tanaka 2001; Tsukada 2019; Tsukada 2020; Utada 1997; Veien 2005; Xue 2021; Yamasaki 2004; Yasli 2019; Zeng 2017; Zeng 2018; Zhang 2007; Zohar 2004).

We judged the remaining 51 studies to be at low risk of bias (Alvarez 2008; Alvarez 2019 hip; Alvarez 2019 knee; Benoni 1996; Benoni 2001; Boese 2017; Camarasa 2006; Cao 2018; Clave 2019; Ellis 2001; Georgiadis 2013; Gill 2009; Gomez Barrena 2014; Goyal 2017; Harley 2002; Johansson 2005; Jules‐Elysee 2019; Kakar 2009 Bilateral TKR; Kakar 2009 Unilateral TKR; Kang 2021a; Karnezis 1994 hip; Karnezis 1994 knee; Kayupov 2017a; Kayupov 2017b; Lei 2017; Lei 2018; Lei 2020; Lemay 2004; Levine 2014; Lopez Picado 2017; Molloy 2007; Morales‐Avalos 2021; North 2016; Orpen 2006; Painter 2018; Peng 2021; Staniforth 2017; Stowers 2017; Vles 2020; Wang 2018; Wang 2019a; Wang 2019b; Wang 2019c; Wu 2018; Xie 2016; Xie 2017; Xu 2023; Yang 2020; Yen 2017; Yen 2021; Zhao 2018).

##### Objective outcomes

We judged all trials to be at low risk of bias as we believed that the blinding would not affect the objective outcomes stated in this review.

#### Incomplete outcome data

We judged three trials to be at high risk of bias (Camarasa 2006; Chin 2020; Johansson 2005).

We judged 25 trials to be at unclear risk of bias (Benoni 2000; Boese 2017; Bradley 2019 hip; Bradley 2019 knee; Colwell 2007; Compostella 1997; Cui 2019; D'Ambrosio 1999; Ekback 2000; Ellis 2001; Garcia Enguita 1998; Garneti 2004; Goyal 2017; Langdown 2000; Lei 2020; Llau 1998; Lopez Picado 2017; Molloy 2007; Murkin 2000; Ray 2005; Staniforth 2017; Tanaka 2001; Xu 2023Yasli 2019; Yen 2021).

We considered the remaining 74 studies to be at low risk of bias (Alvarez 2008; Alvarez 2019 hip; Alvarez 2019 knee; Benoni 1996; Benoni 2001; Cao 2018; Chang 2022; Claeys 2007; Clave 2019; Dorji 2021; Engel 2001; Flordal 1992; Georgiadis 2013; Gill 2009; Gomez Barrena 2014; Gonzalez Osuna 2021; Good 2003; Harley 2002; Hayes 1996; Hiippala 1995; Hiippala 1997; Husted 2003; Jansen 1999; Janssens 1994; Jeserschek 2003; Jules‐Elysee 2019; Kakar 2009 Bilateral TKR; Kakar 2009 Unilateral TKR; Kang 2021a; Kang 2021b; Karnezis 1994 hip; Karnezis 1994 knee; Kayupov 2017a; Kayupov 2017b; King 2019; Lei 2017; Lei 2018; Lemay 2004; Levine 2014; Luo 2022; Morales‐Avalos 2021; Murkin 1995; NCT02922582; Niskanen 2005; North 2016; Orpen 2006; Painter 2018; Peng 2021; Petsatodis 2006; Schott 1995; Sershon 2020; Stowers 2017; Tsukada 2019; Tsukada 2020; Utada 1997; Veien 2002; Veien 2005; Vles 2020; Wang 2018; Wang 2019a; Wang 2019b; Wang 2019c; Wu 2018; Xie 2016; Xie 2017; Xue 2021; Yamasaki 2004; Yang 2020; Yen 2017; Zeng 2017; Zeng 2018; Zhang 2007; Zhao 2018; Zohar 2004).

#### Selective reporting

We assessed 35 trials as being at high risk of bias (Benoni 2000; Boese 2017; Clave 2019; Colwell 2007; Cui 2019; Ellis 2001; Garcia Enguita 1998; Goyal 2017; Hiippala 1995; Hiippala 1997; Jansen 1999; Janssens 1994; Kang 2021b; Karnezis 1994 hip; Karnezis 1994 knee; Kayupov 2017a; Kayupov 2017b; Langdown 2000; Lei 2017; Lei 2018; Lemay 2004; Llau 1998; Lopez Picado 2017; Murkin 2000; Niskanen 2005; Peng 2021; Ray 2005; Sershon 2020; Staniforth 2017; Stowers 2017; Vles 2020; Xie 2017; Zeng 2018; Zhao 2018; Zohar 2004).

We assessed 36 studies as at unclear risk of bias (Alvarez 2008; Benoni 1996; Benoni 2001; Camarasa 2006; Cao 2018; Claeys 2007; Compostella 1997; D'Ambrosio 1999; Ekback 2000; Engel 2001; Flordal 1992; Garneti 2004; Gill 2009; Good 2003; Harley 2002; Hayes 1996; Husted 2003; Jeserschek 2003; Johansson 2005; Kakar 2009 Bilateral TKR; Kakar 2009 Unilateral TKR; Levine 2014; Molloy 2007; Murkin 1995; North 2016; Orpen 2006; Petsatodis 2006; Schott 1995; Tanaka 2001; Tsukada 2019; Utada 1997; Veien 2002; Veien 2005; Xue 2021; Yamasaki 2004; Zhang 2007).

We judged the remaining 31 trials to be at low risk of bias (Alvarez 2019 hip; Alvarez 2019 knee; Bradley 2019 hip; Bradley 2019 knee; Chang 2022; Chin 2020; Dorji 2021; Georgiadis 2013; Gomez Barrena 2014; Gonzalez Osuna 2021; Jules‐Elysee 2019; Kang 2021a; King 2019; Lei 2020; Luo 2022; Morales‐Avalos 2021; NCT02922582; Painter 2018; Tsukada 2020; Wang 2018; Wang 2019a; Wang 2019b; Wang 2019c; Wu 2018; Xie 2016; Xu 2023; Yang 2020; Yasli 2019; Yen 2017; Yen 2021; Zeng 2017).

#### Other potential sources of bias

Other potential biases that we considered included: baseline imbalances, block randomisation in an unblinded trial, and funding and conflict reporting. We also noted where data were being drawn from a non‐peer‐reviewed publication, and any other potential risks.

We judged 17 studies to be at high risk of bias: Benoni 1996 (non‐adherence to protocol, additional TXA given by personal depending on need); Benoni 2000 (baseline imbalances); Boese 2017 (trial stopped early due to data‐dependent process); Colwell 2007 (per protocol analysis); Dorji 2021 (baseline imbalances); Gill 2009 (power calculation re‐done); Harley 2002 (per protocol analysis); Kayupov 2017a (per protocol analysis); Kayupov 2017b (per protocol analysis); Lei 2018 (mismatch between interventions in protocol and in published paper); Molloy 2007 (no demographics reported); Murkin 2000 (other interventions used (EACA and desmopressin) and not reported); NCT02922582 (study terminated early); Niskanen 2005 (per protocol analysis); Ray 2005 (study terminated early and baseline imbalances); Stowers 2017 (conflicts of interest); Veien 2005 (per protocol analysis).

We assessed 46 studies as at unclear risk of bias (Alvarez 2008; Benoni 2001; Bradley 2019 hip; Bradley 2019 knee; Chin 2020; Claeys 2007; Clave 2019; Compostella 1997; Cui 2019; D'Ambrosio 1999; Ellis 2001; Engel 2001; Flordal 1992; Garcia Enguita 1998; Garneti 2004; Good 2003; Goyal 2017; Hayes 1996; Hiippala 1995; Hiippala 1997; Husted 2003; Jansen 1999; Janssens 1994; Jeserschek 2003; Johansson 2005; Kakar 2009 Bilateral TKR; Kakar 2009 Unilateral TKR; Langdown 2000; Lei 2020; Lemay 2004; Llau 1998; Lopez Picado 2017; Luo 2022; Murkin 1995; Orpen 2006; Petsatodis 2006; Staniforth 2017; Tanaka 2001; Utada 1997; Veien 2002; Vles 2020; Yamasaki 2004; Yasli 2019; Zhang 2007; Zhao 2018; Zohar 2004).

We considered the remaining 39 trials at low risk of bias (Alvarez 2019 hip; Alvarez 2019 knee; Camarasa 2006; Cao 2018; Chang 2022; Ekback 2000; Georgiadis 2013; Gomez Barrena 2014; Gonzalez Osuna 2021; Jules‐Elysee 2019; Kang 2021a; Kang 2021b; Karnezis 1994 hip; Karnezis 1994 knee; King 2019; Lei 2017; Levine 2014; Morales‐Avalos 2021; North 2016; Painter 2018; Peng 2021; Schott 1995; Sershon 2020; Tsukada 2019; Tsukada 2020; Wang 2018; Wang 2019a; Wang 2019b; Wang 2019c; Wu 2018; Xie 2016; Xie 2017; Xu 2023; Xue 2021; Yang 2020; Yen 2017; Yen 2021; Zeng 2017; Zeng 2018).

### Effects of interventions

See: Table 1; Table 3

Results are presented primarily for NMA, which we conducted for four outcomes with a reasonably coherent network available for analysis. Direct comparisons included in the NMAs are summarised in Figure 6; Figure 7; Figure 8; and Figure 9. For completeness, the pairwise results for all trials and outcomes are also shown in forest plots grouped by broadly similar treatment nodes and comparisons. The data in the forest plots are treatment nodes and have not been split for multi‐arm trials; totals are not included in the plots.

**6:**
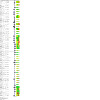


**7:**
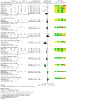


**8:**
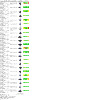


**9:**
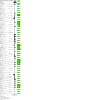


The results of each NMA are reported in full in Appendix 3. This includes a summary of each network; a comparison of model fit for random‐effects consistency and inconsistency models; trace plots and convergence diagnostics; results for all comparisons in the network with forest plots for comparisons with placebo; SUCRA curves and rankings based on SUCRA score.

#### Primary outcomes

##### Risk of allogeneic blood transfusion

###### Network meta‐analysis

See Appendix 3 (section 1), Table 1 and Table 7.

**5:** Summary of participant characteristics by treatment node (NMA only): risk of a blood transfusion up to 30 days post‐surgery

**Node**	**Description of node**	**Participants (number of studies)**	**Characteristic or potential treatment effect modifier** Participants (%) **Fraction of studies with participants that have the potential treatment effect modifier (x/y)**					
			**Surgery type**			**Use of tourniquet (proportion of TKR studies)**	**Transfusion strategy described (proportion of studies)**	**Proportion receiving allogeneic blood transfusion**
			**Primary total hip replacement**	**Primary total knee replacement**	**Mixed including revision/bilateral procedures**			
Placebo	Equivalent volume of normal saline (0.9% NaCl)	1240 (33)^a^	767 (18)	367 (11)	106 (4)	11/11	29/33	0.31
Aprotinin	Aprotinin given intravenously	439 (5)^b^	402 (3)	0 (0)	0 (0)	0/1	3/5	0.13
Desmopressin	Desmopressin given intravenously	20 (1)^c^	0 (0)	20 (1)	0 (0)	1/1	1/1	0.55
EACA (epsilon‐aminocaproic acid)	EACA given intravenously	120 (4)^d^	88 (3)	32 (1)	0 (0)	1/1	3/4	0.13
Fibrin_top	Fibrin spray given intra‐articularly	84 (2)^e^	0 (0)	84 (2)	0 (0)	2/2	2/2	0.12
TXA_IA_1g_intra	TXA given topically (intra‐articularly) at a total dose of 1 g intraoperatively	167 (3)^f^	60 (1)	107 (2)	0 (0)	2/2	3/3	0.04
TXA_IA_2g_intra	TXA given topically (intra‐articularly) at a total dose of 2 g intraoperatively	69 (1)^g^	69 (1)	0 (0)	0 (0)	0/0	1/1	0.17
TXA_IV_1g_intra	TXA given intravenously at a total dose of 1 g intraoperatively	192 (6)^h^	0 (0)	192 (6)	0 (0)	6/6	6/6	0.19
TXA_IV_1g_intra_post	TXA given intravenously at a total dose of 1 g, intraoperatively and postoperatively	66 (2)^i^	0 (0)	66 (2)	0 (0)	2/2	1/2	0.06
TXA_IV_1g_preI	TXA given intravenously at a total dose of 1 g pre‐incision	304 (9)^j^	280 (8)	24 (1)	0 (0)	1/1	8/9	0.23
TXA_IV_1g_preI_intra	TXA given intravenously at a total dose of 1 g pre‐incision and intraoperatively	127 (4)^k^	43 (1)	84 (3)	0 (0)	3/3	4/4	0.15
TXA_IV_1g_preI_intra_post	TXA given intravenously at a total dose of 1 g, pre‐incision, intraoperatively and postoperatively	15 (1)^l^	0 (0)	15 (1)	0 (0)	1/1	1/1	0.67
TXA_IV_1g_preI_post	TXA given intravenously at a total dose of 1 g, pre‐incision and postoperatively	20 (1)^m^	20 (1)	0 (0)	0 (0)	0/0	1/1	0.10
TXA_IV_2g_intra_post	TXA given intravenously at a total dose of 2 g intraoperatively and postoperatively	118 (4)^n^	40 (2)	78 (2)	0 (0)	2/2	4/4	0.16
TXA_IV_2g_preI	TXA given intravenously at a total dose of 2 g pre‐incision	91 (2)^o^	70 (1)	21 (1)	0 (0)	1/1	2/2	0.11
TXA_IV_2g_preI_intra	TXA given intravenously at a total dose of 2 g pre‐incision and intraoperatively	40 (1)^p^	40 (1)	0 (0)	0 (0)	0/0	1/1	0.20
TXA_IV_2g_preI_post	TXA given intravenously at a total dose of 2 g pre‐incision and postoperatively	227 (5)^q^	169 (4)	58 (1)	0 (0)	1/1	5/5	0.07
TXA_IV_3g_intra_post	TXA given intravenously at a total dose of 3 g intraoperatively and postoperatively	94 (3)^r^	50 (1)	39 (1)	5 (1)	1/1	3/3	0.21
TXA_IV_grt_than_3g_intra_post	TXA given intravenously at a total dose of greater than 3 g intraoperatively and postoperatively	71 (1)^s^	0 (0)	0 (0)	71 (1)	1/1	1/1	0.08
TXA_IV_IA_2g_intra	TXA given intravenously and topically (intra‐articularly) at a total dose of 2 g intraoperatively	80 (2)^t^	50 (1)	30 (1)	0 (0)	1/1	2/2	0.06
TXA_IV_IA_2g_preI_intra	TXA given intravenously and topically (intra‐articularly) at a total dose of 2 g pre‐incision and intraoperatively	46 (1)^u^	0 (0)	0 (0)	46 (1)	0/0	1/1	0.17
TXA_IV_IA_grt_than_3g_preI_intra_post	TXA given intravenously and topically (intra‐articularly) at a total dose of greater than 3 g pre‐incision, intraoperatively and postoperatively	74 (1)^v^	74 (1)	0 (0)	0 (0)	0/0	1/1	0.03
TXA_IV_oral_grt_than_3g_intra_post	TXA given intravenously and orally at a total dose of greater than 3 g intraoperatively and postoperatively	113 (2)^w^	0 (0)	113 (2)	0 (0)	2/2	2/2	0.04
TXA_IV_oral_grt_than_3g_preI_post	TXA given intravenously and orally at a total dose of greater than 3 g pre‐incision and postoperatively	60 (1)^x^	0 (0)	60 (1)	0 (0)	0/1	1/1	0.03
TXA_oral_2g_preI	TXA given orally at a total dose of 2 g, pre‐incision	74 (2)^y^	40 (1)	34 (1)	0 (0)	1/1	2/2	0.05
TXA_oral_2g_preI_post	TXA given orally at a total dose of 2 g, pre‐incision and postoperatively	86 (2)^z^	40 (1)	0 (0)	46 (1)	0/0	2/2	0.10
TXA_oral_3g_preI_post	TXA given orally at a total dose of 3 g, pre‐incision and postoperatively	50 (1)^aa^	50 (1)	0 (0)	0 (0)	0/0	1/1	0.02
TXA_oral_grt_than_3g_preI_post	TXA given orally at a total dose of greater than 3 g, pre‐incision and postoperatively	71 (2)^bb^	51 (1)	20 (1)	0 (0)	1/1	2/2	0.07
TXA_oral_IA_grt_than_3g_preI_intra_post	TXA given orally and topically (intra‐articularly) at a total dose of greater than 3 g, pre‐incision, intraoperatively and postoperatively	240 (1)^cc^	240 (1)	0 (0)	0 (0)	0/0	1/1	0.01

DVT: deep vein thrombosis; EACA: epsilon aminocaproic acid; grt_than_3g: greater than 3 g; IA: intra‐articular; intra: intraoperative dose; IV: intravenous; post: postoperative dose; preI: pre‐incision dose; TKR: total knee replacement; top: topical; TXA: tranexamic acid. ^a^Ekback 2000; Ray 2005; Orpen 2006; Gill 2009; Clave 2019; Claeys 2007; Alvarez 2008; Harley 2002; Husted 2003; Jeserschek 2003; Niskanen 2005; Zhao 2018; Benoni 2000; Chin 2020; Murkin 1995; Hiippala 1997; Jansen 1999; Yang 2020; Garneti 2004; Good 2003; Lopez Picado 2017; Painter 2018; Benoni 2001; Murkin 2000; Zeng 2017; Camarasa 2006; Johansson 2005; Benoni 1996; Petsatodis 2006; Tanaka 2001; Hiippala 1995; Colwell 2007; Zhang 2007. ^b^Colwell 2007; Jeserschek 2003; Murkin 1995; Murkin 2000; Ray 2005. ^c^Ellis 2001. ^d^Camarasa 2006; Harley 2002; Morales‐Avalos 2021; Ray 2005. ^e^Molloy 2007; Yen 2021. ^f^Stowers 2017; Wang 2019c; Yang 2020. ^g^North 2016. ^h^Chang 2022; Good 2003; Molloy 2007; Orpen 2006; Tanaka 2001; Zeng 2018. ^i^Alvarez 2008; Zohar 2004. ^j^Benoni 2001; Chin 2020; Claeys 2007; Garneti 2004; Johansson 2005; Lopez Picado 2017; Luo 2022; Sershon 2020; Tanaka 2001. ^k^Ellis 2001; Kayupov 2017a; Kayupov 2017b; Tanaka 2001. ^l^Hiippala 1995. ^m^Husted 2003. ^n^Benoni 1996; Benoni 2000; Camarasa 2006; Ekback 2000. ^o^Jansen 1999; North 2016. ^p^Sershon 2020. ^q^Clave 2019; Lopez Picado 2017; Niskanen 2005; Wang 2019c; Zhao 2018. ^r^Gill 2009; Hiippala 1997; Wu 2018. ^s^Painter 2018. ^t^Zeng 2017; Zeng 2018. ^u^Sershon 2020. ^v^Clave 2019. ^w^Chang 2022; Zohar 2004. ^x^Wang 2019a ‐ the study stated that tourniquet was not used in any patient. ^y^Kayupov 2017a; Kayupov 2017b. ^z^Sershon 2020; Zhao 2018. ^aa^Wu 2018. ^bb^Morales‐Avalos 2021; Zohar 2004. ^cc^Wang 2019c.

All included participants had either a primary total hip or knee replacement, unicompartmental knee replacement, bilateral replacements or a revision of a hip or knee replacement.

The NMA included a total of 47 RCTs, involving 4398 participants. There were 406 possible pairwise comparisons and 44 comparisons with direct data and a total of 765 blood transfusions. Trials excluded from the network are summarised in section 1.1.2 of Appendix 3. The direct results for each included treatment node are summarised in Figure 6.

There were considerable differences between nodes in the risk of allogeneic blood transfusion, ranging from 1% to 67%, reflecting increasingly restrictive transfusion policies over time (Appendix 3, sections 1.1.3 and 1.1.4). This may be responsible for some moderate heterogeneity seen within groups of trials making similar comparisons (Figure 6). Garneti 2004, for example, commented: *"Perhaps this was because of the different transfusion strategies of the anesthetists, one of whom transfused most patients unless they were young and healthy. We established no defined criteria for administering blood transfusion in this trial, and this could be a source of bias."*

There is no evidence that these differences have invalidated the transitivity assumption of our NMA model, with little difference between the consistency and inconsistency models (Appendix 3, section 1.2.2).

Appendix 3, section 1.2.3 shows the forest plot of all interventions included within the network compared to placebo with risk ratios (RRs) and 95% credible intervals (CrIs). There is some evidence of benefit for all but one of the included interventions (desmopressin) but the credible intervals are wide and certainty of the evidence is low. The network is sparsely populated, with not many more trials than there are treatments to compare, with all the trials being small or very small.

###### Treatment ranking

The SUCRA plot in section 1.2.5 of Appendix 3 plots the cumulative ranking probabilities with treatment nodes involving tranexamic acid identified by line thickness to indicate dose, line colour to indicate route(s) of administration and line style to indicate timing. While the results for individual regimens should be treated with caution due to the limited amount of direct evidence in the network, the SUCRA plot does suggest that higher doses appear more effective, regimens including oral administration perform well and combined routes may be the most effective strategy, although this observation is somewhat confounded by dose.

###### Pairwise analyses

Data for all studies that reported the primary outcome risk of requiring allogeneic blood transfusion within 30 days are presented in forest plots: TXA intravenous (IV) versus placebo (Analysis 2.1); TXA oral versus placebo (Analysis 3.1); TXA topical versus placebo (Analysis 4.1); TXA IV + TXA topical versus placebo (Analysis 5.1); TXA IV lower dose versus TXA IV higher dose (Analysis 6.1); TXA IV versus TXA oral (Analysis 7.1); TXA IV versus TXA topical (Analysis 8.1); TXA oral lower dose versus TXA oral higher dose (Analysis 9.1); TXA topical lower dose versus TXA topical higher dose (Analysis 10.1); TXA versus aprotinin (Analysis 11.1); TXA IV versus EACA (Analysis 12.1); TXA oral versus EACA oral (Analysis 13.1); TXA IV versus desmopressin (Analysis 14.1); TXA IV versus fibrin topical (Analysis 15.1); TXA topical versus fibrin topical (Analysis 16.1); aprotinin versus placebo (Analysis 17.1); EACA versus placebo (Analysis 18.1); EACA versus aprotinin (Analysis 19.1); fibrin topical versus placebo (Analysis 21.1); TXA IV + TXA oral versus TXA IV (Analysis 22.1); TXA IV + TXA topical versus TXA IV (Analysis 23.1); TXA IV + TXA topical versus TXA oral (Analysis 24.1); TXA IV + TXA topical versus TXA topical (Analysis 25.1); TXA topical versus TXA oral + TXA topical (Analysis 26.1); TXA oral versus TXA combined topical + IV + oral (Analysis 27.1); TXA IV + topical lower dose versus TXA IV + topical higher dose (Analysis 28.1); TXA oral + topical lower dose versus TXA oral + topical higher dose (Analysis 29.1).

##### All‐cause mortality

We did not have enough data to present an NMA for our primary outcome of mortality within 30 days of surgery.

We presented the available data in pairwise meta‐analyses: TXA IV versus placebo (Analysis 2.2); TXA oral versus placebo (Analysis 3.2); TXA topical versus placebo (Analysis 4.2); TXA IV lower dose versus TXA IV higher dose (Analysis 6.2); TXA IV versus TXA topical (Analysis 8.2); TXA oral lower dose versus TXA oral higher dose (Analysis 9.2); TXA topical lower dose versus TXA topical higher dose (Analysis 10.2); TXA oral versus EACA oral (Analysis 13.2); TXA IV versus fibrin topical (Analysis 15.2); TXA topical versus fibrin topical (Analysis 16.2); aprotinin versus placebo (Analysis 17.2); desmopressin versus placebo (Analysis 20.1); fibrin topical versus placebo (Analysis 21.2); TXA IV + TXA oral versus TXA IV (Analysis 22.2); TXA topical versus TXA oral + TXA topical (Analysis 26.2); TXA oral + topical lower dose versus TXA oral + topical higher dose (Analysis 29.2).

#### Secondary outcomes

##### Mean number of red cell units transfused per person (up to 30 days)

We had planned to report the number of transfusion episodes but no studies reported this outcome; instead we reported the number of red cell units transfused per participant.

###### Network meta‐analysis

See Appendix 3 (section 2).

We were able to conduct an NMA for this outcome. We analysed the number of units per person randomised, using reported means and standard deviations. We included 16 studies and nine interventions in the NMA. There were a total of 1223 participants within the network, with a total of 36 pairwise comparisons, with 10 comparisons containing direct data. The direct results for each included treatment node are summarised in Figure 7.

The mean number of units transfused within each node varied from 0.19 to 1.65, reflecting increasingly restrictive transfusion policies over time. This may be responsible for some moderate heterogeneity seen within two of the groups of trials making similar comparisons (Figure 7). Garneti 2004, for example, commented: *"Perhaps this was because of the different transfusion strategies of the anesthetists, one of whom transfused most patients unless they were young and healthy. We established no defined criteria for administering blood transfusion in this trial, and this could be a source of bias."*

There is no evidence that these differences have invalidated the transitivity assumption of our NMA model, with little difference between the consistency and inconsistency models (Appendix 3, section 2.2.2).

There is some evidence of reduced volume of blood transfusion in favour of aprotinin and some TXA regimens, with no evidence of benefit for desmopressin or EACA, although all the confidence intervals are wide (Appendix 3, section 2.2.3). The network is sparsely populated, with not many more trials than there are treatments to compare, with all the trials being small or very small.

###### Treatment ranking

The SUCRA plot in section 2.2.5 of Appendix 3 plots the cumulative ranking probabilities with treatment nodes involving tranexamic acid identified by line thickness to indicate dose, line colour to indicate route(s) of administration and line style to indicate timing. All of the TXA regimens included in this network were IV only. The results for individual regimens should be treated with caution due to the limited amount of direct evidence in the network.

###### Pairwise analyses

Data for all studies that reported the outcome mean number of red cell units transfused per person up to 30 days are presented in pairwise meta‐analyses: TXA IV versus placebo (Analysis 2.3); TXA IV lower dose versus TXA IV higher dose (Analysis 6.3); TXA IV versus EACA (Analysis 12.2); aprotinin versus placebo (Analysis 17.3); EACA versus placebo (Analysis 18.2); desmopressin versus placebo (Analysis 20.2).

##### Re‐operation due to bleeding (within seven days)

We did not have enough data to present an NMA for the secondary outcome re‐operation due to bleeding.

Data for all studies that reported the outcome are presented in pairwise meta‐analyses: TXA IV versus placebo (Analysis 2.4); TXA topical versus placebo (Analysis 4.3); TXA IV lower dose versus TXA IV higher dose (Analysis 6.4); TXA IV versus TXA topical (Analysis 8.3); TXA topical lower dose versus TXA topical higher dose (Analysis 10.3); TXA IV versus EACA (Analysis 12.3); TXA oral versus EACA oral (Analysis 13.3); TXA topical versus fibrin topical (Analysis 16.3); aprotinin versus placebo (Analysis 17.4); EACA versus placebo (Analysis 18.3); EACA versus aprotinin (Analysis 19.2); fibrin topical versus placebo (Analysis 21.3); TXA IV + TXA oral versus TXA IV (Analysis 22.3).

##### Length of hospital stay

###### Network meta‐analysis

See Appendix 3 (section 4).

We were able to conduct an NMA for this outcome. We included 28 studies and 30 interventions in the NMA. There were a total of 3205 participants within the network with a total of 435 pairwise comparisons, with direct data available for 44 comparisons. The direct results for each included treatment node are summarised in Figure 9.

The mean length of hospital stay varied from 1.97 to 13.9 days, with the single trial of aprotinin reporting a much longer stay than the other trials in the network. There is little heterogeneity within direct comparisons for this outcome, primarily because there were few direct comparisons made by more than one trial.

There is little difference between the consistency and inconsistency models for this outcome (section 4.2.2, Appendix 3).

There is limited evidence of reduced hospital stay for any treatment regimen, although all the confidence intervals are wide (section 4.2.3, Appendix 3). The network is sparsely populated, with not many more trials than there are treatments to compare, with all the trials being small or very small (Appendix 3).

###### Treatment ranking

The SUCRA plot (section 4.2.5, Appendix 3) shows the cumulative ranking probabilities with treatment nodes involving tranexamic acid identified by line thickness to indicate dose, line colour to indicate route(s) of administration, and line style to indicate timing. The results for individual regimens should be treated with caution due to the limited amount of direct evidence in the network. There is little evidence from the SUCRA plot that dose, route or timing of treatment has any consistent effect on length of hospital stay (Appendix 3).

###### Pairwise analyses

Data for all studies that reported the outcome are presented in pairwise meta‐analyses: TXA IV versus placebo (Analysis 2.5); TXA oral versus placebo (Analysis 3.3); TXA topical versus placebo (Analysis 4.4); TXA IV + TXA topical versus placebo (Analysis 5.2); TXA IV lower dose versus TXA IV higher dose (Analysis 6.5); TXA IV versus TXA oral (Analysis 7.3); TXA IV versus TXA topical (Analysis 8.4); TXA IV versus EACA (Analysis 12.4); TXA IV versus desmopressin (Analysis 14.2); TXA topical versus fibrin topical (Analysis 16.4); aprotinin versus placebo (Analysis 17.5); fibrin topical versus placebo (Analysis 21.4); TXA IV + TXA oral versus TXA IV (Analysis 22.4); TXA IV + TXA topical versus TXA IV (Analysis 23.2); TXA IV + TXA topical versus TXA oral (Analysis 24.2); TXA topical versus TXA oral + TXA topical (Analysis 26.3); TXA oral versus TXA combined topical + IV + oral (Analysis 27.2); TXA IV + topical lower dose versus TXA IV + topical higher dose (Analysis 28.2); TXA oral + topical lower dose versus TXA oral + topical higher dose (Analysis 29.3).

We could not separate hip and knee data for one study comparing desmopressin versus placebo (Karnezis 1994 hip; Karnezis 1994 knee). The combined result for both hip and knee populations was mean difference (MD) 0.50 (95% confidence interval (CI) ‐1.57 to 0.57, 92 participants).

##### Adverse events

###### Deep vein thrombosis

####### Network meta‐analysis

See Appendix 3 (section 3), Table 3 and Table 8.

**6:** Summary of participant characteristics by treatment node (NMA only): risk of deep vein thrombosis (DVT) up to 90 days post‐surgery

**Node**	**Description of node**	**Participants (number of studies)**	**Characteristics or potential treatment effect modifier** Participants (%) **Fraction of studies with participants that have the potential treatment effect modifier (x/y)**					
			**Surgery type**			**Use of tourniquet (proportion of TKR studies)**	**Transfusion strategy described (proportion of studies)**	**Proportion experiencing DVT**
			**Primary total hip replacement**	**Primary total knee replacement**	**Mixed including revision/bilateral procedures**			
Placebo	Equivalent volume of normal saline (0.9% NaCl)	630 (12)^a^	326 (5)	235 (6)	69 (1)	7/7	12/12	0.05
Aprotinin	Aprotinin given intravenously	408 (3)^b^	396 (2)	12 (1)	0 (0)	1/1	3/3	0.08
TXA_IA_1g_intra	TXA given topically (intra‐articularly) at a total dose of 1 g intraoperatively	106 (2)^c^	60 (1)	46 (1)	0 (0)	1/1	2/2	0.03
TXA_IA_2g_intra	TXA given topically (intra‐articularly) at a total dose of 2 g intraoperatively	50 (1)^d^	0 (0)	50 (1)	0 (0)	1/1	2/2	0.08
TXA_IV_1g_intra	TXA given intravenously at a total dose of 1 g intraoperatively	96 (3)^e^	0 (0)	96 (3)	0 (0)	3/3	3/3	0.16
TXA_IV_1g_post	TXA given intravenously at a total dose of 1 g postoperatively	50 (1)^f^	0 (0)	50 (1)	0 (0)	1/1	1/1	0.06
TXA_IV_1g_preI	TXA given intravenously at a total dose of 1 g pre‐incision	106 (3)^g^	35 (1)	71 (2)	0 (0)	2/2	3/3	0.14
TXA_IV_1g_preI_intra	TXA given intravenously at a total dose of 1 g pre‐incision and intraoperatively	39 (2)^h^	0 (0)	39 (2)	0 (0)	2/2	2/2	0.38
TXA_IV_2g_intra_post	TXA given intravenously at a total dose of 2 g intraoperatively and postoperatively	83 (3)^i^	40 (2)	43 (1)	0 (0)	1/1	3/3	0.10
TXA_IV_2g_post	TXA given intravenously at a total dose of 2 g postoperatively	53 (1)^j^	0 (0)	53 (1)	0 (0)	1/1	1/1	0.08
TXA_IV_2g_preI_post	TXA given intravenously at a total dose of 2 g pre‐incision and postoperatively	128 (3)^k^	36 (1)	58 (1)	34 (1)	0/2	3/3	0.06
TXA_IV_3g_intra_post	TXA given intravenously at a total dose of 3 g intraoperatively and postoperatively	39 (1)^l^	0 (0)	39 (1)	0 (0)	1/1	1/1	0.05
TXA_IV_grt_than_3g_intra_post	TXA given intravenously at a total dose of greater than 3 g intraoperatively and postoperatively	71 (1)^m^	0 (0)	0 (0)	71 (1)	1/1	1/1	0.01
TXA_IV_IA_2g_preI_intra	TXA given intravenously and topically (intra‐articularly) at a total dose of 2 g pre‐incision and intraoperatively	54 (1)^n^	0 (0)	54 (1)	0 (0)	0/1	1/1	0.04
TXA_IV_IA_grt_than_3g_preI_intra_post	TXA given intravenously at a total dose of greater than 3 g pre‐incision and postoperatively	89 (2)^o^	0 (0)	46 (1)	43 (1)	0/2	2/2	0.07
TXA_IV_oral_grt_than_3g_intra_post	TXA given intravenously and orally at a total dose of greater than 3 g intraoperatively and postoperatively	93 (1)^p^	0 (0)	93 (1)	0 (0)	1/1	1/1	0.02
TXA_IV_oral_grt_than_3g_preI_post	TXA given intravenously and orally at a total dose of greater than 3 g pre‐incision and postoperatively	60 (1)^q^	0 (0)	60 (1)	0 (0)	0/1	1/1	0.08
TXA_oral_IA_grt_than_3g_preI_intra_post	TXA given intravenously and orally at a total dose of greater than 3 g, pre‐incision, intraoperatively and postoperatively	240 (1)^r^	240 (1)	0 (0)	0 (0)	0/0	1/1	0.03

DVT: deep vein thrombosis; grt_than_3g: greater than 3 g; IA: intra‐articular; intra: intraoperative dose; IV: intravenous; post: postoperative dose; preI: pre‐incision dose; TKR: total knee replacement; top: topical; TXA: tranexamic acid. ^a^Benoni 1996; Benoni 2000; Colwell 2007; Ekback 2000; Georgiadis 2013; Good 2003; Hiippala 1997; Lopez Picado 2017; Murkin 2000; Painter 2018; Tanaka 2001; Xue 2021. ^b^Colwell 2007; Engel 2001; Murkin 2000. ^c^Peng 2021; Wang 2019c. ^d^Georgiadis 2013. ^e^Chang 2022; Good 2003; Tanaka 2001. ^f^Xue 2021. ^g^Lopez Picado 2017; Peng 2021; Tanaka 2001. ^h^Engel 2001; Tanaka 2001. ^i^Benoni 1996; Benoni 2000; Ekback 2000. ^j^Xue 2021. ^k^Lopez Picado 2017; Tsukada 2019 ‐ the study stated that tourniquet was not used in any patient; Wang 2019a ‐ the study stated that tourniquet was not used in any patient. ^l^Hiippala 1997. ^m^Painter 2018. ^n^Tsukada 2020 ‐ the study stated that tourniquet was not used in any patient. ^o^Tsukada 2019; Tsukada 2020 ‐ the studies stated that tourniquet was not used in any patient. ^p^Chang 2022. ^q^Wang 2019a ‐ the study stated that tourniquet was not used in any patient. ^r^Wang 2019c.

We were able to conduct an NMA for the secondary outcome deep vein thrombosis (DVT). We included 19 studies in the network with a total of 2395 participants. There were 153 possible pairwise comparisons, with direct data available for 23 comparisons. There were 168 events in the network. Trials excluded from the network are summarised in section 3.1.2 of Appendix 3. The direct results for each included treatment node are summarised in Figure 8.

There were considerable differences between nodes in the risk of DVT, ranging from 1% to 38%, which probably reflects the subjectivity of this outcome (Appendix 3, sections 3.1.3 and 3.1.4). There was little evidence of heterogeneity within direct comparisons, primarily because few comparisons included more than one trial.

There is no evidence that the different rates of diagnosis of DVT have affected the transitivity assumption of our NMA model, with little difference between the consistency and inconsistency models (Appendix 3, section 3.2.2)

Appendix 3, section 3.2.3 shows the forest plot of all interventions included within the network compared to placebo with risk ratios (RRs) and 95% credible intervals (CrIs). There is no evidence of harm with respect to the risk of DVT and some evidence overall of a potential protective effect, but the credible intervals are wide and the certainty of the evidence is low.

####### Pairwise analyses

Data for all studies that reported the outcome are presented in pairwise meta‐analyses: TXA IV versus placebo (Analysis 2.6); TXA oral versus placebo (Analysis 3.4); TXA topical versus placebo (Analysis 4.5); TXA IV + TXA topical versus placebo (Analysis 5.3); TXA IV lower dose versus TXA IV higher dose (Analysis 6.6); TXA IV versus TXA oral (Analysis 7.4); TXA IV versus TXA topical (Analysis 8.5); TXA oral lower dose versus TXA oral higher dose (Analysis 9.3); TXA versus aprotinin (Analysis 11.2); TXA IV versus EACA (Analysis 12.5); TXA oral versus EACA oral (Analysis 13.4); TXA IV versus desmopressin (Analysis 14.3); TXA IV versus fibrin topical (Analysis 15.5); TXA topical versus fibrin topical (Analysis 16.5); aprotinin versus placebo (Analysis 17.6); EACA versus placebo (Analysis 18.4); EACA versus aprotinin (Analysis 19.3); desmopressin versus placebo (Analysis 20.3); fibrin topical versus placebo (Analysis 21.5); TXA IV + TXA oral versus TXA IV (Analysis 22.5); TXA IV + TXA topical versus TXA IV (Analysis 23.3); TXA IV + TXA topical versus TXA oral (Analysis 24.3); TXA topical versus TXA oral + TXA topical (Analysis 26.4); TXA oral versus TXA combined topical + IV + oral (Analysis 27.3); TXA IV + topical lower dose versus TXA IV + topical higher dose (Analysis 28.3); TXA oral + topical lower dose versus TXA oral + topical higher dose (Analysis 29.4).

###### Pulmonary embolism, myocardial infarction and cerebrovascular event (CVA or stroke)

We did not have enough data to present NMAs for the secondary outcomes pulmonary embolism, myocardial infarction and CVA (stroke) within 30 days.

####### Pulmonary embolism pairwise analyses

Data for all studies that reported the outcome are presented in pairwise meta‐analyses: TXA IV versus placebo (Analysis 2.7); TXA oral versus placebo (Analysis 3.5); TXA topical versus placebo (Analysis 4.6); TXA IV + TXA topical versus placebo (Analysis 5.4); TXA IV lower dose versus TXA IV higher dose (Analysis 6.7); TXA IV versus TXA oral (Analysis 7.5); TXA IV versus TXA topical (Analysis 8.6); TXA oral lower dose versus TXA oral higher dose (Analysis 9.4); TXA IV versus EACA (Analysis 12.6); TXA oral versus EACA oral (Analysis 13.5); TXA IV versus desmopressin (Analysis 14.4); TXA IV versus fibrin topical (Analysis 15.6); TXA topical versus fibrin topical (Analysis 16.6); aprotinin versus placebo (Analysis 17.7); EACA versus placebo (Analysis 18.5); EACA versus aprotinin (Analysis 19.4); desmopressin versus placebo (Analysis 20.4); fibrin topical versus placebo (Analysis 21.6); TXA IV + TXA topical versus TXA IV (Analysis 23.4); TXA IV + TXA topical versus TXA oral (Analysis 24.4); TXA topical versus TXA oral + TXA topical (Analysis 26.5); TXA IV + topical lower dose versus TXA IV + topical higher dose (Analysis 28.4); TXA oral + topical lower dose versus TXA oral + topical higher dose (Analysis 29.5).

####### Myocardial infarction pairwise analyses

Data for all studies that reported the outcome are presented in pairwise meta‐analyses: TXA IV versus placebo (Analysis 2.8); TXA oral versus placebo (Analysis 3.6); TXA IV lower dose versus TXA IV higher dose (Analysis 6.8); TXA IV versus TXA topical (Analysis 8.7); TXA oral lower dose versus TXA oral higher dose (Analysis 9.5); TXA IV versus EACA (Analysis 12.7); TXA oral versus EACA oral (Analysis 13.6); aprotinin versus placebo (Analysis 17.8); EACA versus placebo (Analysis 18.6); desmopressin versus placebo (Analysis 20.5); TXA topical versus TXA oral + TXA topical (Analysis 26.6); TXA IV + topical lower dose versus TXA IV + topical higher dose (Analysis 28.5); TXA oral + topical lower dose versus TXA oral + topical higher dose (Analysis 29.6).

####### Cerebrovascular event (CVA or stroke) pairwise analyses

Data for all studies that reported the outcome are presented in pairwise meta‐analyses: TXA IV versus placebo (Analysis 2.9); TXA oral versus placebo (Analysis 3.7); TXA topical versus placebo (Analysis 4.7); TXA IV lower dose versus TXA IV higher dose (Analysis 6.9); TXA IV versus TXA oral (Analysis 7.6); TXA IV versus TXA topical (Analysis 8.8); TXA oral lower dose versus TXA oral higher dose (Analysis 9.6); TXA topical lower dose versus TXA topical higher dose (Analysis 10.4); TXA IV versus EACA (Analysis 12.8); TXA oral versus EACA oral (Analysis 13.7); TXA topical versus fibrin topical (Analysis 16.7); aprotinin versus placebo (Analysis 17.9); EACA versus placebo (Analysis 18.7); fibrin topical versus placebo (Analysis 21.7); TXA IV + TXA topical versus TXA IV (Analysis 23.5); TXA IV + TXA topical versus TXA oral (Analysis 24.5); TXA topical versus TXA oral + TXA topical (Analysis 26.7); TXA IV + topical lower dose versus TXA IV + topical higher dose (Analysis 28.6); TXA oral + topical lower dose versus TXA oral + topical higher dose (Analysis 29.7).

####### Transfusion reactions within 24 hours

Only one study reported on transfusion reactions within 24 hours (Yang 2020) and one pairwise analysis was conducted (Analysis 4.8).

####### Suspected serious drug reactions: within 30 days

Few studies reported the secondary outcome suspected serious drug reaction within 30 days and there was not enough data to conduct an NMA.

Data for all studies that reported the outcome are presented in pairwise meta‐analyses: TXA IV versus placebo (Analysis 2.10); TXA IV lower dose versus TXA IV higher dose (Analysis 6.10); TXA IV versus TXA topical (Analysis 8.9); TXA topical lower dose versus TXA topical higher dose (Analysis 10.5); TXA oral versus EACA oral (Analysis 13.8); aprotinin versus placebo (Analysis 17.10); desmopressin versus placebo (Analysis 20.6).

####### Cost and quality of life data

We collected information on the cost of interventions and quality of life measures where they were reported. Thirty‐one studies reported information on cost and five studies reported any quality of life data. We summarise the information in Table 9 and Table 10, respectively.

**7:** Table of descriptive cost information

**Study**	**Cost information**
None of the included studies reported quantitative cost data. However, some studies have reported descriptive information. Where a direct quote has been taken from the study, we have indicated by the use of speech marks (" ").	
**Alvarez 2008**	"In contrast, results of this study also question the use of presurgical donation of autologous blood in patients undergoing total knee arthroplasty in our institution, because of 11 patients in which this procedure was used, only 3 received blood transfusion. Therefore, the use of presurgically donated units is far from the 70 percent recommended for an adequate cost effectiveness ratio".
**Benoni 1996**	"At our hospital, one Sagman unit of blood costs 512 SEK (51 GBP). The price of one ampoule of Cyklokapron, containing one gram of tranexamic acid, is 42 SEK (4 GBP). The total cost of blood transfusions plus tranexamic acid was 9756 SEK (976 GBP) in the whole prophylactic group against 21 110 SEK (2111 GBP) in the whole placebo group".
**Benoni 2001**	"The price of one ampoule of tranexamic acid (1 gram) in Sweden is 5 Euro. In our department, 1 unit of leukocyte‐depleted erythrocyte concentrate costs 77 Euro. The total cost of tranexamic acid and blood transfusions in the TA group was 475 Euro versus 1100 Euro in the placebo group".
**Boese 2017**	"Antifibrinolytics were added to the blood management program for TKA in June 2012. At that time, TXA was not on the formulary, and its acquisition cost was much higher than that of EACA ($43/g for TXA compared with $0.20/g for EACA). Despite its higher cost, our surgeons preferred administrating TXA over EACA because of the paucity of data on the use of EACA in TKA. However, EACA was administered when TXA was unavailable, with no apparent differences in efficacy or drug‐related adverse events".
**Bradley 2019 hip**	"At the investigating hospital, TXA costs $465 per patient while EACA costs $60 per patient for the dosages used in this study. Due to the low rate of transfusion and no statistical difference in LOS, there appears to be a difference of about $400 (pharmacy cost) between these two agents. Unfortunately, there have been problems with the availability of EACA: currently, it is not available due to a national shortage".
**Chin 2020**	"When one considers the financial cost of such treatment, the total drug cost of tranexamic acid (NZ$58) is less than the production cost of a unit of allogenic blood (NZ$158) [12]. However, using the number needed to treat of 67, this study effectively spent $3886 on TXA to save 1 unit of blood. 'is cost was not retrieved in a significantly shorter duration of stay as shown by the time to discharge".
**Claeys 2007**	"The reduction in the risk associated with the transfusion of allogenic blood, as well as the cost‐effectiveness are obvious (3 amp TA €5, 1 unit of packed cells €67 ; total cost TA group :€100 vs placebo group:€871)".
**Colwell 2007**	"We did not examine the costs of using aprotinin. Realizing costs and charges vary, the approximate direct cost of aprotinin we used was $450. This does not, however, take into account the staff time in preparation in the pharmacy and administration in the OR".
**Ekback 2000**	"As both IAT and PAD are costly and time‐consuming procedures, it seems reasonable to refrain from using one or both of them if TA is to be used, although this was not examined in the present study".
**Georgiadis 2013**	"We observed a trend towards decreased blood transfusion in the TNA group vs. the placebo group (8% and 0% respectively), but our results were not significant and therefore no “number needed to treat” analysis could be undertaken. Models that reflect the real world costs of blood utilization in the United States estimate that a single unit of allogenic leukoreduced red blood cells costs $950[1]. At our institution one 2 g dose of tranexamic acid can be compounded for $60 USD, and it is readily available internationally for $6 USD per dose. A cost–benefit analysis would be beneficial in determining the realized benefit of TXA administration in preventing allogenic transfusion, as pecuniary considerations become increasingly important and regulated in orthopaedic surgery".
**Gill 2009**	"The cost of allogenic blood transfusions was reduced by approximately $800 per patient (P<0.03) in the tranexamic acid group. Moreover, only one patient in the tranexamic acid group received a transfusion, whereas four patients received transfusions in the placebo group. This translated into a significantly lower cost in blood products administered in the tranexamic acid group with even taking into account the cost of tranexamic acid".
**Gomez Barrena 2014**	"Retrospective clinical and economical evaluations have indicated an estimated $1500 savings per primary total knee replacement performed with use of topical TXA, with significant decreases in length of stay, blood bank costs, and total direct costs to the hospital for the total knee replacement. We confirmed that the length of stay was short and blood bank costs were reduced to a minimum when TXA was used in the present study. Indirect cost savings would also result from the avoidance of transfusions that result in complications requiring additional treatment and an increased length of stay".
**Good 2003**	"In our hospital the dose of tranexamic acid given would cost less than £7, compared with £46 for a unit of banked blood. Thus, the immediate saving in the patients given tranexamic acid would have been about £1100. To our knowledge, giving tranexamic acid is the only blood saving method that is cheaper, per saved unit, than banked blood in this type of surgery. This estimate does not include potential adverse effects from banked blood such as immediate transfusion reactions, transmission of infectious agents and disturbances of the immune system".
**Harley 2002**	"The cost of the preparation and administration of EACA as described in this study is Can$80 per patient, so this agent represents one of the most cost‐effective modalities currently under investigation".
**Husted 2003**	"1 blood transfusion costs 93 Euro; 4 ampoules (2 grams) of tranexamic acid cost 18 Euro. The total costs of maintaining or restoring levels of haemoglobin thus amounts to 1092 Euro in the tranexamic group and 2325 Euro in the placebo group".
**Janssens 1994**	"Although Aprotinin is expensive (in Belgium, about $235 for 3.5 x 10 (6) kIU) the economic benefit of reducing the requirement for blood transfusion may justify the cost".
**Jeserschek 2003**	"At the current price of aprotinin, approximately £75 (120 euros) per patient was spent on each operation. The price of 1.8 fewer units of blood (approximately £120 (190 euros)), led to a mean saving of £45 (70 euros) per patient".
**Johansson 2005**	"In this study, 4/5 patients weighed more than 67 kg and would have needed 2 ampoules of TA, since 1 ampoule contains 1,000 mg. If all patients had received TA 15 mg/kg, the average cost would have been (180 ampoules × EUR 5)/ 100 patients = EUR 9 per patient. At our hospitals, the cost for 1 unit of blood is EUR 78. The cost saving for transfusions when tranexamic acid is used would be: EUR 78/unit × reduction in average transfusion (1.08 – 0.36 units) = EUR 56. Thus, if the results from this study were generalized, the cost saving would be EUR 56 – EUR 9 = EUR 47 per patient".
**Kakar 2009 Bilateral TKR****;****Kakar 2009 Unilateral TKR**	"Patients in the tranexamic acid group were given 4 units of blood in total, compared with 26 units in the control group. In our hospital the dose of tranexamic acid given would cost Rs. 166, compared with Rs. 6000 for a unit of leucodepleted banked blood. Thus, the immediate saving in the patients given tranexamic acid would have been about Rs. 5000. •Cost of 1 unit leuco depleted PRBC = Rs. 6000 •Total cost of blood in Control patients ( 26 units) = Rs 1,56,000 •Total cost of blood in TAX patients ( 4 units) = Rs 24, 000 •Cost of 1 ampoule of TXA = Rs 166 •Cost of TXA ( 25 patients) = Rs 8,300 •Cost of blood saved by giving TXA= Rs 1,23,700 •Cost saved per patient = Rs 4958 •Potential savings per year ( 500 patients) = Rs 25,00,000".
**Kayupov 2017a**	"In the present study, the oral TXA dosage cost $14 compared with $47‐$108 depending on the availability of the generic IV formulation. Given the aging population, the utilization of primary total knee replacements will only grow from the current rate of approximately 700,000 per year in the United States. As a result, the transition to oral TXA could yield total cost savings of between $23 million and $67 million dollars per year for our health care system".
**Molloy 2007**	"At the time of our study, the cost of the pharmaceutical intervention involved in the topical fibrin group was £380 per patient whereas in the tranexamic acid group it was less than £4".
**Murkin 1995**	"Currently, the cost of aprotinin in Canada averages $450 ($590 Cdn) for this dosage of 3.8 m KIU. The average reduction in transfusion requirements of 0.9 U PRBC shown here, may not be sufficient to justify this expenditure. If the trend to reduction in DVT, as demonstrated in both recent studies in this high‐risk population, can be confirmed, however, the resultant decrease in morbidity and associated length of stay could render this therapy cost effective".
**Murkin 2000**	"Notably, the direct cost of one unit of allogeneic blood (approximately $150 [United States dollars] per unit) is comparable with that of the starting dose of aprotinin, with a current hospital acquisition cost for use in a hip replacement of approximately $162 for a low dose of 100 millilitres of aprotinin to $486 for a high dose of 300 millilitres. Moreover, on the basis of the total incremental hospital costs of hip arthroplasty, allogeneic blood transfusion may be associated with $1000 to $1500 per unit in additional costs compared with the cost of no transfusion or of transfusion of one to five units of autologous blood. Thus, use of aprotinin may be of particular clinical and economic benefit in patients at high risk of receiving allogeneic blood, such as those who have not predonated blood or perhaps those with a low baseline haemoglobin level".
**Niskanen 2005**	"One unit of red cells costs EUR 90, and 6 ampoules of tranexamic acid used for one patient cost EUR 13. Thus, the total cost per patient amounts to EUR 58 in the tranexamic acid group and EUR 81 in the placebo group. If we use only 2 ampoules of tranexamic acid preoperatively and drain only in the placebo group, the costs would amount to EUR 50 and EUR 100. According to the Finnish arthroplasty registry, about 2500 hip operations per year in Finland might be suitable for this kind of policy (Nevalainen et al. 2003). It means a saving of about EUR 32,500–125,000. If we take uncemented and revision cases into account, the saving will increase many fold".
**North 2016**	"cost analysis using IV TXA demonstrated a savings of $314 USD per patient".
**Ray 2005**	"The cost of these doses of aprotinin and EACA is Aus $401 and $71 respectively, i.e. aprotinin is more than ﬁve times the cost, bears a risk of anaphylactic reaction and has similar effect in reducing bleeding".
**Veien 2002**	"The blood‐sparing effect of TXA has a high cost‐beneﬁt ratio. The cost of short‐term TXA therapy is significantly less than the cost of autologous and allogenic blood transfusions".
**Wang 2018**	"An appropriate oral dose can save between $33 and $94 compared with an equivalent intravenous or intra‐articular dose, depending on the formulations of TXA. We came to the same conclusion about the costs. Although several authors have conﬁrmed the enhanced efficacy of higher or additional intravenous administration of TXA in arthroplasty, to our knowledge there have been no prior RCTs determining the optimum regimen for oral TXA, which is associated with great cost savings, ease of administration, and equivalent clinical blood‐conserving efficacy".
**Wu 2018**	"The total TXA cost in the oral TXA group was signiﬁcantly less compared to that in the IV TXA group (¥600 and ¥ 3150, P < 0.01)".
**Zeng 2018**	"The mean hospital charge in the extension, and controlled group was 7070$, and 7140$, respectively, without significant intergroup differences".
**Zhao 2018**	"The cost associated with oral TXA (546 RMB total patients) was significantly lower than that of intravenous TXA (4573.2 RMB total patients; p = 0.001; Table 2). The oral TXA dosage cost 6.83 RMB per dose. The cost of 1g of IV TXA was 76.30 RMB, the cost of oral form of TXA is cheaper than the intravenous form, and beside its relatively low cost, the advantage of oral TXA is simple application avoiding IV access, which is requirement for expensive nursing care for IV application. The transfusion cost per two U red blood cells was estimated to be 930 RMB at our hospital. Costs of TXA and transfusions were significantly lower in the oral group than the intravenous group (p < 0.05). Similarly, the cost of transfusion was significantly lower in the oral group (929.65 RMB total transfusion) than in the intravenous group (1859.3 RMB total transfusion) and control group (8366.8 RMB, total transfusion; p = 0.004; Table 2)".

DVT: deep vein thrombosis; EACA: epsilon‐aminocaproic acid; IAT: intraoperative autotransfusion; LOS: length of stay; IV: intravenous; OR: operating room; PAD: preoperative autologous blood donation; RCT: randomised controlled trial; TKA: total knee arthroplasty; TXA, TA, TNA: tranexamic acid

**8:** Table of descriptive HRQoL information

**Study**	**Intervention**	**HRQoL information**
None of the included studies reported health‐related quality of life data. However, some studies have reported descriptive information.		
Chin 2020	TXA, IV, 1 g	There was no significant difference between TXA and placebo groups in the improvement of functional scales, comparing the preoperative to the 1‐year postoperative scores. The Oxford Hip Score showed a mean improvement of 25.9 points in those patients who received TXA, compared with 26.7 points in those who received placebo (P = 0.679). The WOMAC scores were improved by 49.9 points in the TXA group, compared with 50.7 points in the placebo group (P = 0.864). The mean improvement in the HAAS was 7.5 points in the TXA group, compared with 8.2 points in the placebo group (P = 0.278).
	Placebo	
Morales‐Avalos 2021	TXA, oral	VAS score, Harris hip score. VAS preop 7.88 ± 1.54. HHS (points) 48.10 ± 8.48. VAS postop 30 days 1.38 ± 0.95. HHS postop 30 days 84.99 ± 12.92.
	EACA, oral	VAS score, Harris hip score. VAS preop 8.01 ± 1.22. HHS 49.56 ± 9.01. VAS postop 30 days 1.59 ± 1.02. HHS postop 30 days 83.13 ±14.69.
Painter 2018	Placebo	EQ‐5D indexed, median (interquartile range (IQR)) preoperative: 0.38 (0.22 to 0.60), Week 3: 0.64 (0.54 to 0.74), Week 6: 0.67 (0.59 to 0.84), Month 3: 0.73 (0.64 to 0.84), Month 6: 0.77 (0.59 to 0.91). Quality of recovery score, median (IQR) Day 3: 102 (84 to 123), Week 3: 120 (107 to 138), Week 6: 124 (102 to 140) WOMAC® Index, median (IQR) preoperative: 64 (51 to 72), Week 3: 30 (21 to 48), Week 6: 28 (16 to 38), Month 3: 21 (10 to 37), Month 6: 18 (10 to 33). Oxford score (hip or knee), median (IQR) preoperative: 44 (38 to 50), Week 3: 35 (26 to 41), Week 6: 28 (21 to 35), Month 3: 24 (17 to 30), Month 6: 21 (15 to 29).
	TXA	EQ‐5D indexed, median (interquartile range (IQR)) preoperative: 0.42 (0.19 to 0.58), Week 3: 0.65 (0.52 to 0.74), Week 6: 0.73 (0.59 to 0.84), Month 3: 0.74 (0.64 to 0.84), Month 6: 0.77 (0.66 to 1.00). Quality of recovery score, median (IQR) Day 3: 106 (88 to 122), Week 3: 119 (104 to 127), Week 6: 129 (116 to 139). WOMAC® Index, median (IQR) preoperative: 61 (54 to 71), Week 3: 35 (25 to 45), Week 6: 28 (16 to 37), Month 3: 19 (12 to 31), Month 6: 17 (8 to 32). Oxford score (hip or knee), median (IQR) preoperative: 43 (39 to 50), Week 3: 32 (28 to 37), Week 6: 26 (21 to 32), Month 3: 23 (19 to 28), Month 6: 23 (17 to 28).
Xie 2017	TXA, IV pre‐op + placebo, IV, postop, repeated dose	VAS score pre‐op day 1: 3.1, VAS score postop day 1: 2.7, VAS score postop day 2: 2.4, VAS score postop day 3: 2.2
	TXA, IV pre‐op + TXA, IV, postop + placebo, IV, postop	VAS score pre‐op day 1: 2.9, VAS score postop day 1: 2.5, VAS score postop day 2: 2.2, VAS score postop day 3: 2.2
	TXA, IV pre‐op + TXA, IV, postop, repeated dose	VAS score pre‐op day 1: 3.3, VAS score postop day 1: 1.8, VAS score postop day 2: 2.0, VAS score postop day 3: 1.9
Yen 2017	Placebo	Average VAS mean score (1 day postop): 3.89 ± 0.83
	TXA, IV	Average VAS mean score (1 day postop): 3.84 ± 0.74
	TXA, IA	Average VAS mean score (1 day postop): 3.93 ± 0.84

EACA: epsilon aminocaproic acid; HAAS: High Activity Arthroplasty Score; HHS: Harris Hip Score; HRQoL: health‐related quality of life; IA: intra‐articular; IQR: interquartile range; IV: intravenous; TXA: tranexamic acid; VAS: visual analogue scale; WOMAC: Western Ontario and McMaster Universities Arthritis Index

###### Subgroup analysis

We were unable to perform any of the subgroup analyses detailed in our protocol (Gibbs 2023), due to the very limited networks remaining after the data were split.

###### Sensitivity analysis

No included study reported a dropout rate of more than 20%; therefore, we did not perform any further sensitivity analyses. Sensitivity analyses by risk of bias could not be performed as planned. Exclusion of studies with high risk of bias resulted in loss of connectivity.

## Discussion

### Summary of main results

We aimed to determine the relative efficacy of pharmacological interventions for preventing blood loss in elective primary or revision hip or knee replacement, and to identify optimal administration of interventions regarding timing, dose and route. We identified 102 eligible RCTs including participants undergoing hip or knee replacement surgery. Our primary outcomes were the proportion of participants requiring an allogeneic blood transfusion and all‐cause mortality; secondary outcomes included the mean number of units transfused per participant (up to 30 days), reoperation due to bleeding (within seven days), length of hospital stay and adverse events including DVT, pulmonary embolism, myocardial infarction and stroke, transfusion reactions (acute): within 24 hours and serious suspected serious drug reactions: within 30 days. We also collected cost data and quality of life data, where they were reported in the included studies.

There are relatively few data to support the large number of treatment regimens identified. There is low‐certainty evidence that TXA given at higher doses, intra‐articularly and orally, is likely to be the most effective approach for reducing the need for blood transfusion in people undergoing hip or knee replacement surgery (Table 1; Table 7). The ranking of individual treatments should be interpreted with caution given the limited amount of evidence contributing to each comparison.

Tranexamic acid interventions consistently ranked higher than other treatments such as aprotinin, EACA and topical fibrin sealants compared with placebo. We noted that mixed routes of administration (oral and intra‐articular, intravenous and intra‐articular) appear to be more effective than single routes of administration and higher doses of tranexamic acid feature higher up the treatment ranking hierarchy. Oral tranexamic acid appears to perform well, which is an important finding as oral tranexamic acid is cheaper and easier to administer than intravenous tranexamic acid (GBP 6.01 for sixty 500 mg tablets (30 g) versus GBP 15.47 for five 1 g ampoules (5 g), BNF 2022).

Mortality was not reported by many trials, which is likely due to the low risk of death in people undergoing hip or knee replacement surgery.

We found that there was little to no evidence of harm associated with any of the interventions compared with placebo. In particular, the number of thromboembolic events was low in all arms and there is no evidence that higher doses of tranexamic acid increased this risk. In fact, while the estimates are imprecise, what evidence there is suggests that the risk may actually be reduced (Table 3; Table 8). This may be due to the anti‐inflammatory effects of TXA (TXA reduces the levels of inflammatory proteins such as C‐reactive protein and interleukin‐6) within people undergoing orthopaedic procedures who receive TXA compared to those receiving no or lower doses of TXA (Okholm 2022).

### Overall completeness and applicability of evidence

We excluded all studies published after 2010 that were unregistered, or retrospectively registered, as per our protocol and in line with Cochrane Injuries Editorial Policy (Broughton 2021; Cochrane policy; Roberts 2015). This may have excluded some relevant and useful studies from the review.

Given the ability of studies to compare all the various combinations of drug, route, dose and timing, we conducted an NMA to enable the combination of direct and indirect evidence and to rank different treatment interventions in a methodologically robust way. Our review includes 102 trials assessing a variety of drug regimens for the prevention of bleeding in people undergoing hip or knee surgery. The review includes all pre‐registered trials identifiable through bibliographic databases and trial registries, with no date restrictions.

Our review has limitations. The trials included in this review were small, with a large number of interventions tested, resulting in a sparsely populated network, wide credible intervals and low certainty in the evidence for any specific treatment. Evidence for some of the interventions studied was informed by a single trial, which led to imprecision and low certainty of evidence. The included population was quite homogeneous, as it was limited to people who had undergone a hip or knee replacement. However, some variation in the use of topical tranexamic acid or fibrin sealants in these populations could affect the transitivity assumption as they may have been administered in different ways (e.g. through bathing the joint during surgery or injected within the tissues). Transitivity may have also been affected by transfusion thresholds. The variations in the criteria to trigger a transfusion could have a significant influence on the pooled studies for analysis, especially if in the presence of a network with fewer connections.

Whilst 102 eligible trials were identified, we could only include 47 in the NMA for our primary outcome. Many studies could not be included due to observing zero events in one or more arms, and some did not connect within the network (Appendix 3).

Although we were able to undertake an NMA for our primary outcome (risk of needing an allogeneic blood transfusion), we did not have enough data to conduct an NMA for all‐cause mortality. Only 19 studies reported the outcome all‐cause mortality and many studies reported zero events. Similarly, we did not have enough data to conduct an NMA for reoperation due to bleeding within seven days or adverse events, except for DVT. The rate of adverse events, including reoperation for bleeding, was low and concerns over increased risk of thromboembolic events are not borne out by the evidence we identified.

Our current protocol does not include plans for regular updating (Gibbs 2023); however, we identified 30 ongoing trials planning to recruit 3776 participants, which may allow firmer conclusions to be drawn in future.

### Quality of the evidence

The overall degree of certainty of the evidence evaluated ranged from very low to moderate based on grading using the CINeMA assessment. However, in general there was not enough good evidence to draw definitive conclusions. The degree of certainty of the evidence of our top‐ranking TXA treatments was assessed as low, except for TXA given orally and intra‐articularly at a total dose of greater than 3 g pre‐incision, intraoperatively and postoperatively. The main reason for downgrading the certainty of evidence was imprecision (wide credible and/or confidence intervals) and within‐study bias. Many comparisons yielded low‐certainty evidence due to these concerns (Table 2; Table 4). This means that we are not able to draw any firm conclusions on the optimal dose, route and timing of administration of TXA.

### Potential biases in the review process

We have attempted to minimise bias in the review process. We conducted a comprehensive search: we searched multiple data sources (including multiple databases and clinical trial registries) to ensure that all relevant studies would be captured. There were no restrictions on the language in which reports were originally published. We assessed the relevance of each publication carefully and performed all screening and data extraction in duplicate. We prespecified all outcomes and subgroups prior to analysis.

We excluded trials that did not prospectively register their protocol (for publications since 2010) to minimise the potential for bias from the included data, although we accept that this may have excluded some relevant and useful studies (Gibbs 2023). However, the decision to exclude unregistered (or retrospectively registered) trials was taken due to the evidence highlighting issues surrounding false data, including the possibility of 'zombie' trials, where a trial did not even take place (Carlisle 2021; Roberts 2015). Prospective registration reduces the chance of publication bias, and has been compulsory for randomised controlled trials since 2005, thus suggesting that those that have not been registered (or registered retrospectively) since then are less likely to be at low risk of bias (Roberts 2015).

We planned subgroup analyses by type of surgery (primary hip or knee replacement or hip or knee revision), reason for surgery, duration of surgery, incidence of preoperative anaemia, type of anaesthetic used (general or spinal), use of tourniquet and use of anticoagulation. However, the data were too limited to allow informative subgroup analyses. Similarly, our sensitivity analyses by risk of bias could not be performed as planned. Exclusion of studies with high risk of bias resulted in loss of connectivity.

There were a large number of interventions tested in a relatively small number of trials, all with small sample sizes. We grouped the interventions according to total dose, route and timing to provide a manageable set for analysis and inevitably some detail is lost, especially for postoperative infusion strategies. There are not sufficient data to establish the most effective regimen of those tested in these trials, only some broad general trends.

### Agreements and disagreements with other studies or reviews

Our findings have demonstrated greater efficacy of tranexamic acid compared to placebo and other pharmacological agents studied. TXA has been shown to be effective for preventing bleeding in people undergoing hip or knee replacement surgery in other reviews. Fillingham et al performed a network meta‐analysis of randomised trials using tranexamic acid in people having a primary hip replacement (Fillingham 2018). These authors included 34 studies in their review. They similarly concluded that there was strong evidence to support the use of TXA to reduce blood loss and risk of transfusion; however, they were not able to clearly identify superior routes of administration, dosage, dosing regimen or timing of administration. They found that oral TXA may not have been as effective as other routes of intervention, a finding that this review did not conclude. As with our review, the authors found that many treatments relied on a limited number of studies connecting the nodes and therefore relied more heavily on indirect comparisons.

The same group of authors also conducted an NMA of randomised trials in people undergoing primary knee replacement (Fillingham 2018a). They included 67 studies in their review. They found that there was strong evidence to support the use of TXA to reduce blood loss and the risk of transfusion in people undergoing a primary knee replacement. However, they were not able to conclude a superior route or dose of administration. They did, however, find moderate evidence to support the use of TXA pre‐incision.

Another recent NMA looking at tranexamic acid use in people undergoing both hip and knee replacement found that TXA given intravenously and intra‐articularly provided the best efficacy to prevent transfusion (Xu 2019a). We did not draw this conclusion. Our review found that interventions including intra‐articular administration and oral TXA regimens may be more beneficial in reducing the need for blood transfusion. Importantly, all three reviews studying tranexamic acid report no increased risk of adverse events compared with placebo.

In some countries, EACA is cheaper than TXA and has been preferred for use in people undergoing hip or knee replacement surgery. A meta‐analysis study conducted by Riaz et al focused on the efficacy of EACA compared with TXA in reducing the need for blood transfusion (Riaz 2019). They found three studies comparing TXA and EACA and concluded that TXA was not superior to EACA, and both antifibrinolytic therapies demonstrated similar efficacy in terms of transfusion requirements and blood loss. Our review found that TXA was superior to EACA in terms of reducing the need for blood transfusion.

In this review, we have focused exclusively on people undergoing elective (planned) surgery, excluding those studies that had a mixed population where we could not separate the relevant data. Our sister review focused on non‐elective surgery only (Gibbs 2023).

## Authors' conclusions

**Implications for practice**

Tranexamic acid (TXA) probably reduces the need for blood transfusion in people undergoing hip or knee replacement surgery. Other antifibrinolytics (aprotinin and epsilon‐aminocaproic acid) are not as effective at reducing the need for allogeneic blood transfusion as tranexamic acid. We are not able to draw strong conclusions about the optimal dose, route and timing of administration. We found that tranexamic acid given at higher doses tended to rank higher in the treatment hierarchy, and we also found that it may be more beneficial to use a mixed route of administration (oral and intra‐articular, or intravenous and intra‐articular). Oral administration may be as effective as intravenous administration of tranexamic acid at reducing the risk of allogeneic blood transfusion. Although cost‐effectiveness was not directly assessed in this review, oral tranexamic acid is widely known to be cheaper than intravenous and this may provide a cheaper alternative to intravenous tranexamic acid with similar efficacy. We found little to no evidence of harm associated with higher doses of tranexamic acid in the risk of deep vein thrombosis (DVT). However, we are not able to definitively draw these conclusions based on the trials included within this review.

**Implications for research**

The majority of trials included in this review had a small number of participants, which affected the quality of the network meta‐analysis. Larger, adequately powered randomised controlled trials, conducted in a way that reduces bias, need to be carried out in order for us to ascertain the optimal dose, route and timing of administration of tranexamic acid. Studies including people undergoing revision hip and knee replacement, for whom blood loss is higher, are also needed to evaluate the optimal dose, route and timing of tranexamic acid. Currently, there are no ongoing trials identified that are studying people undergoing revision hip or knee replacement surgery.

## History

Protocol first published: Issue 3, 2019

## Appendices

### Appendix 1. Table of intervention variables

This table highlights the scope of our question by demonstrating all possible variable combinations

**Table:**

	**TXA**	**Aprotinin**	**Epsilon‐aminocaproic acid**	**Desmopressin**	**Factor VIIa**	**Factor XIII**	**Fibrinogen**	**Fibrin sealants/glue**	**Non‐fibrin sealants**
**Timing**									
**Pre‐operative**	✓	✓	✓	✓	✓	✓	✓	X	X
**Intraoperative**	✓	✓	✓	✓	✓	✓	✓	✓	✓
**Postoperative**	✓	X	X	✓	✓	✓	✓	X	X
**Route**									
**IV (injection, infusion)**	✓	✓	✓	✓	✓	✓	✓	X	X
**Topical**	✓	X	X	X	X	X	X	✓	✓
**Intranasal**	X	X	X	✓	X	X	X	X	X
**Subcutaneous injection**	X	X	X	✓	X	X	X	X	X
**IV + topical**	✓	X	X	X	X	X	X	X	X
**Oral**	✓	X	✓	X	X	X	X	X	X
**IV + oral**	✓	X	X	X	X	X	X	X	X
**Topical + oral**	✓	X	X	X	X	X	X	X	X
**Dose**									
**Single**	✓	X	✓	✓	✓	✓	✓	✓	✓
**Multiple**	✓	✓	X	✓	✓	✓	✓	X	X
**Variable units/kg**	✓	X	✓	X	✓	✓	✓	X	X
**Variable trial set dose**	✓	✓	X	✓	✓	✓	✓	✓	✓
**Abbreviations** IV: intravenous TXA: tranexamic acid **Notes** This table is for illustrative purposes only and is limited to transfusion‐related indications. Ticks indicate which intervention and timing or route or dose combinations are clinically possible. Crosses indicate which intervention and timing or route or dose combinations are not clinically possible.									

### Appendix 2. Search strategies

#### CENTRAL (*The Cochrane Library*)

#1 MeSH descriptor: [Arthroplasty, Replacement, Hip] this term only #2 MeSH descriptor: [Hip] this term only and with qualifier(s): [surgery ‐ SU] #3 MeSH descriptor: [Osteoarthritis, Hip] explode all trees and with qualifier(s): [surgery ‐ SU] #4 MeSH descriptor: [Arthroplasty, Replacement, Knee] this term only #5 MeSH descriptor: [Knee Injuries] explode all trees and with qualifier(s): [surgery ‐ SU] #6 MeSH descriptor: [Knee] explode all trees and with qualifier(s): [surgery ‐ SU] #7 MeSH descriptor: [Osteoarthritis, Knee] explode all trees and with qualifier(s): [surgery ‐ SU] #8 ((hip* or knee* or femur or femoral) near/10 (replac* or operat* or surg* or arthroplast* or reconstruct* or repair* or osteotom* or alloplast* or plast* or resurfac* or implant* or prosthes*)) #9 (acetabuloplast* or acetabulum arthroplast*) #10 (THA or THR or TKA or TKR or UKA or UKR) #11 #1 or #2 or #3 or #4 or #5 or #6 or #7 or #8 or #9 or #10 #12 MeSH descriptor: [Antifibrinolytic Agents] this term only #13 MeSH descriptor: [Tranexamic Acid] this term only #14 MeSH descriptor: [Aminocaproic Acid] explode all trees #15 antifibrinolytic* or anti‐fibrinolytic* or antifibrinolysin* or antiplasmin* or plasmin inhibitor* or tranexamic or tranhexamic or cyclohexanecarboxylic acid* or amcha or t‐amcha or amca or transamin or amchafibrin or anvitoff or spotof or cyklokapron or femstrual or ugurol #16 AMCHA or amchafibrin or amikapron or amstat or antivoff or caprilon or cl65336 or cyclocapron or cyclokapron or cyklocapron or cyklokapron or exacyl or frenolyse or fibrinon or hemostan or hexacapron #17 hexakapron or kalnex or lysteda or rikaparin or ronex or theranex or tranexam or tranexanic or tranexic or "trans achma" or transexamic or trenaxin or TXA #18 (fibrinolysis near/2 inhibitor*) #19 (Agretax or Bio‐Stat or Capiloc or Capitrax or Clip Inj or Clot‐XL or Clotawin‐T or Coastat or Cuti or Cymin or Dubatran or Espercil or Examic or Existat or Extam or Fibran or Gynae‐Pil or Hemstate or Kapron or Menogia or Monitex or Nestran or Nexamic or Nexi‐500 or Nexmeff or Nicolda or Nixa‐500 or Pause or Rheonex or Sylstep TX or Synostat or T‐nex or T Stat or T Stat or Tanmic or Temsyl‐T or Texakind or Texanis or Texapar or Texid or Thams or Tonopan or Traklot or Tramic or Tramix or Tranarest or Trance Inj or Tranecid or Tranee or Tranemic or Tranex or Tranexa or Tranfib or Tranlok or Transtat or Transys or Transcam or Tranxi or Trapic or Traxage or Traxamic or Traxyl or Trenaxa or Trexamic or Trim Inj or Tx‐1000 or Tx 500 or Wistran or X‐Tran or Xamic) #20 ecapron or ekaprol or epsamon or epsicaprom or epsicapron or epsilcapramin or epsilon amino caproate or epsilon aminocaproate or epsilonaminocaproic or epsilonaminocapronsav or ethaaminocaproic or ethaaminocaproich or emocaprol or hepin or ipsilon or neocaprol or resplamin or tachostyptan #21 lederle or acikaprin or afibrin or amicar or caprocid or capracid or capramol or caprogel or caprolest or caprolisin* or caprolysin* or capromol or epsikapron or hemocaprol or caproamin or EACA or caprolest or capralense or hexalense or hamostat or hemocid #22 aminohexanoic or aminocaproic or aminohexanoic or amino caproic or amino‐caproic or amino‐n‐hexanoic #23 #12 or #13 or #14 or #15 or #16 or #17 or #18 or #19 or #20 or #21 or #22 #24 MeSH descriptor: [Aprotinin] this term only #25 (antagosan or antilysin* or aprotimbin or apronitin* or aprotinin* or bayer a128 or contrical or contrycal or contrykal or dilmintal or frey inhibitor or kontrycal or Kunitz inhibitor or gordox or haemoprot or kallikrein‐trypsin inactivator or iniprol or kontrikal or kontrykal or kunitz pancreatic trypsin inhibitor or midran or pulmin or tracylol or trascolan or trasilol or tra?ylol or traskolan or zymofren or pancreas antitrypsin or protinin or riker 52g or Rivilina zymofren) #26 #24 or #25 #27 MeSH descriptor: [Factor VIIa] this term only #28 (factor viia or factor 7a or rfviia or fviia or novoseven* or novo seven* or aryoseven or acset or eptacog* or proconvertin) #29 (activated near/1 (factor seven or factor vii or rfvii or fvii)) #30 (factor seven or factor vii or factor 7):ti #31 #27 or #28 or #29 or #30 #32 MeSH descriptor: [Fibrinogen] this term only #33 ("fibrinogen concentrate" or "factor I" or Haemocomplettan* or Riastap* or Fibryga* or Fibryna*) #34 #32 or #33 #35 MeSH descriptor: [Deamino Arginine Vasopressin] this term only #36 (desmopressin* or vasopressin deamino or D amino D arginine vasopressin or deamino 8 d arginine vasopressin or vasopressin desamino 8 arginine or desmotabs or DDAVP or desmogalen or adin or adiuretin or concentraid or d void or dav ritter or deamino 8 dextro arginine vasopressin or deamino 8d arginine vasopressin or deamino dextro arginine vasopressin or deaminovasopressin or defirin or defirin melt or desmirin pr desmomelt or desmopresina or desmospray or desmotab* or desurin or emosint or enupresol or minirin or minirinette or minirinmelt or minrin or minurin or miram or nictur or noctisson or nocturin or nocutil or nordurine or novidin or nucotil or octim or octostim or presinex or stimate or wetirin) #37 #35 or #36 #38 MeSH descriptor: [Factor XIII] explode all trees #39 (factor xiii or fxiii or fibrin stabili?ing factor* or Tretten* or Catridecacog) #40 #38 or #39 #41 MeSH descriptor: [Tissue Adhesives] explode all trees #42 MeSH descriptor: [Collagen] explode all trees and with qualifier(s): [therapeutic use ‐ TU] #43 MeSH descriptor: [Thrombin] explode all trees and with qualifier(s): [therapeutic use ‐ TU] #44 MeSH descriptor: [Gelatin] explode all trees and with qualifier(s): [therapeutic use ‐ TU] #45 MeSH descriptor: [Gelatin Sponge, Absorbable] this term only #46 ((fibrin* or collagen or cellulose or gelatin or gel or thrombin* or albumin or hemostatic* or haemostatic*) next (glu* or seal* or adhesive* or topical* or local* or matrix or matrices or spong* or fleece* or foam* or scaffold* or patch* or sheet* or bandag* or aerosol* or dressing* or paste or powder*)) #47 ((nonfibrin* or non‐fibrin* or synthetic* or non‐biological* or nonbiological* or biological*) near/3 (glue* or seal* or adhesive*)) #48 (surgical* near/3 (glue* or sealant* or adhesive*)) #49 ((fibrin* or collagen or cellulose or gelatin or thrombin) near/3 (hemosta* or haemosta*)) #50 (8Y or Aafact or Actif‐VIII or Advate or Artiss or Bioglue or Biocol or Collaseal or Omrixil or Transglutine or Raplixa or Evarrest or Aleviate or Alphanate or Amofil or Beriate or Beriplast or Biostate or Bolheal or Cluvot or Conco‐Eight‐HT or Crosseel or Crosseal or Crosseight or Emoclot or Evarrest or Evicel or Factane or Fanhdi or Fibrogammin P or Green VIII or Green VIII Factor or Greengene or Greenmono or Greenplast or Haemate or Haemate P or Haemate P or Haemate P500 or Haemate‐P or Haemoctin or Haemoctin SDH or Haemoctin‐SDH or Hemaseel or Hemaseal or Hemofil M or Hemoraas or Humaclot or Humafactor‐8 or Humate‐P or Immunate or Innovate or Koate or Koate‐DVI or Kogenate Bayer or Kogenate FS or Monoclate‐P or NovoThirteen or Octafil or Octanate or Octanate or Optivate or Quixil or Talate or Tisseel or Tisseal or Tissel or Tissucol or Tricos or Vivostat or Voncento or Wilate or Wilnativ or Wilstart or Xyntha) #51 (Glubran or Gluetiss or Ifabond or Indermil or LiquiBand or TissuGlu or Evithrom or Floseal or Hemopatch or Gel‐Flow or Gelfoam or Gelfilm or Recothrom or Surgifoam or Surgiflo* or "rh Thrombin" or Thrombi‐Gel or Thrombi‐Pad or Thrombin‐JMI or Thrombinar or Thrombogen or Thrombostat) #52 (porcine gelatin or bovine collagen or bovine gelatin or nu‐knit or arista or hemostase or vita sure or thrombin‐jmi or thrombinjmi or avicel or vivagel or lyostypt or tabotamp or arterx or omnex or veriset) #53 (polysaccharide next (sphere* or hemostatic powder)) #54 MeSH descriptor: [Chitosan] this term only #55 MeSH descriptor: [Polyethylene Glycols] this term only and with qualifier(s): [therapeutic use ‐ TU] #56 MeSH descriptor: [Hydrogel, Polyethylene Glycol Dimethacrylate] explode all trees and with qualifier(s): [therapeutic use ‐ TU] #57 MeSH descriptor: [Polyurethanes] explode all trees and with qualifier(s): [pharmacology ‐ PD, adverse effects ‐ AE, toxicity ‐ TO, administration & dosage ‐ AD, therapeutic use ‐ TU] #58 ((polymer‐derived elastic* or polymer tissue adhesive* or elastic hydrogel* or glutaraldehyde or PEG‐based or polyurethane‐based tissue or polyethylene glycol* or polyvinyl alcohol‐based tissue or PVA‐based tissue or natural biopolymer* or polypeptide‐based or protein‐based or polysaccharide‐based or chitosan or poliglusam or cyanoacrylic or cyanoacrylate or cyacrin or dextran‐based or chondroitin sulfate‐based or mussel‐inspired elastic* or glycol hydrogel or polymer‐based) next (glu* or seal* or adhesive* or topical* or local* or matrix or matrices or spong* or fleece* or foam* or scaffold* or patch* or sheet* or bandag* or aerosol* or dressing* or paste* or powder*)) #59 (chitosan* or mucoadhesive or muco‐adhesive or Celox Gauze or ChitoGauze or Combat Gauze) #60 MeSH descriptor: [Cellulose, Oxidized] this term only #61 (absorbable cellulose or resorbable cellulose or oxidi?ed cellulose or carboxycellulose or oxycellulose or cellulosic acid or oxycel or oxidi?ed regenerated cellulose) #62 (BioGlue or Progel or Duraseal or Coseal or FocalSeal or ADAL‐1 or AdvaSeal or Pleuraseal or Angio‐Seal or Avitene or Instat or Helitene or Helistat or TDM‐621 or Dermabond or Tissueseal or PolyStat or Raplixa or Spongostan or Surgicel or Surgilux or Tachosil or Traumstem) #63 (collagen‐thrombin or thrombin‐collagen or gelatin‐fibrinogen or fibrinogen‐gelatin or gelatin‐thrombin or thrombin‐gelatin or fibrinogen‐thrombin or thrombin‐fibrinogen or collagen‐fibrinogen or fibrinogen‐collagen or microfibrillar collagen or CoStasis or "GRF Glue" or GR‐Dial or Algosterile or TraumaStat or HemCon or ChitoFlex or Celox or QuikClot or WoundStat or Vitagel or TachSeal or TachoComb or Cryoseal) #64 #41 or #42 or #43 or #44 or #45 or #46 or #47 or #48 or #49 or #50 or #51 or #52 or #53 or #54 or #55 or #56 or #57 or #58 or #59 or #60 or #61 or #62 or #63 #65 MeSH descriptor: [Waxes] explode all trees #66 (bonewax* or bone wax* or bone putty or hemasorb or ostene) #67 #65 or #66 #68 MeSH descriptor: [Hemostatics] this term only and with qualifier(s): [administration & dosage ‐ AD, therapeutic use ‐ TU] #69 (((haemosta* or hemosta* or antihaemorrhag* or antihemorrhag* or anti haemorrhag* or anti‐hemorrhag*) next (drug* or agent* or treat* or therap* or dressing*)) or ((coagulat* or clotting) next factor*)) #70 #23 or #26 or #31 or #34 or #37 or #40 or #64 or #67 or #68 or #69 #71 #11 and #70

#### MEDLINE (Ovid)

1. Arthroplasty, Replacement, Hip/ 2. Hip/su 3. Osteoarthritis, Hip/su 4. Arthroplasty, Replacement, Knee/ 5. exp Knee Injuries/su 6. Knee/su 7. Osteoarthritis, Knee/su 8. ((hip* or knee* or femur or femoral) adj10 (replac* or operat* or surg* or arthroplast* or reconstruct* or repair* or osteotom* or alloplast* or plast* or resurfac* or implant* or prosthes*)).tw,kf. 9. (acetabuloplast* or acetabulum arthroplast*).tw,kf. 10. (THA or THR or TKA or TKR or UKA or UKR).tw. 11. or/1‐10 12. Antifibrinolytic Agents/ 13. Tranexamic Acid/ 14. Aminocaproic Acid/ 15. (antifibrinolytic* or anti‐fibrinolytic* or antifibrinolysin* or antiplasmin* or plasmin inhibitor* or tranexamic or tranhexamic or cyclohexanecarboxylic acid* or amcha or trans‐4‐aminomethyl‐cyclohexanecarboxylic acid* or t‐amcha or amca or "kabi 2161" or transamin or amchafibrin or anvitoff or spotof or cyklokapron or cyclo‐F or femstrual or ugurol or aminomethylcyclohexanecarbonic acid or aminomethylcyclohexanecarboxylic acid or AMCHA or amchafibrin or amikapron or aminomethyl cyclohexane carboxylic acid or aminomethyl cyclohexanecarboxylic acid or aminomethylcyclohexane carbonic acid or aminomethylcyclohexane carboxylic acid or aminomethylcyclohexanecarbonic acid or aminomethylcyclohexanecarboxylic acid or aminomethylcyclohexanocarboxylic acid or aminomethylcyclohexanoic acid or amstat or antivoff or caprilon or cl?65336 or cl65336 or cyclocapron or cyclokapron or cyklocapron or cyklokapron or exacyl or frenolyse or fibrinon or hemostan or hexacapron or hexakapron or kalnex or lysteda or rikaparin or ronex or theranex or tranexam or tranexanic or tranexic or trans achma or transexamic or trenaxin or TXA or (fibrinolysis adj2 inhibitor*)).tw,kf. 16. (Agretax or Bio‐Stat or Capiloc or Capitrax or Clip Inj or Clot‐XL or Clotawin‐T or Coastat or Cuti or Cymin or Dubatran or Espercil or Examic or Existat or Extam or Fibran or Gynae‐Pil or Hemstate or Kapron or Menogia or Monitex or Nestran or Nexamic or Nexi‐500 or Nexmeff or Nicolda or Nixa‐500 or Pause or Rheonex or Sylstep TX or Synostat or T‐nex or T Stat or T Stat or Tanmic or Temsyl‐T or Texakind or Texanis or Texapar or Texid or Thams or Tonopan or Traklot or Tramic or Tramix or Tranarest or Trance Inj or Tranecid or Tranee or Tranemic or Tranex or Tranexa or Tranfib or Tranlok or Transtat or Transys or Transcam or Tranxi or Trapic or Traxage or Traxamic or Traxyl or Trenaxa or Trexamic or Trim Inj or Tx‐1000 or Tx 500 or Wistran or X‐Tran or Xamic).tw,kf. 17. (6‐aminohexanoic or amino?caproic or amino?hexanoic or amino caproic or amino‐caproic or amino‐n‐hexanoic or cy‐116 or cy116 or lederle or acikaprin or afibrin or amicar or caprocid or capracid or capramol or caprogel or caprolest or caprolisin* or caprolysin* or capromol or epsikapron or hemocaprol or caproamin or EACA or caprolest or capralense or hexalense or hamostat or hemocid or cl 10304 or cl10304 or ecapron or ekaprol or epsamon or epsicaprom or epsicapron or epsilcapramin or epsilon amino caproate or epsilon aminocaproate or epsilonaminocaproic or epsilonaminocapronsav or etha?aminocaproic or ethaaminocaproich or emocaprol or hepin or ipsilon or jd?177or neocaprol or nsc?26154 or resplamin or tachostyptan).tw,kf. 18. or/12‐17 19. Aprotinin/ 20. (antagosan or antilysin* or aprotimbin or apronitin* or aprotinin* or bayer a128 or contrical or contrycal or contrykal or dilmintal or frey inhibitor or kontrycal or Kunitz inhibitor or gordox or haemoprot or kallikrein‐trypsin inactivator).tw,kf. 21. (iniprol or kontrikal or kontrykal or kunitz pancreatic trypsin inhibitor or midran or pulmin or tracylol or trascolan or trasilol or tra?ylol or traskolan or zymofren or pancreas antitrypsin or protinin or riker 52g or Rivilina zymofren).tw,kf. 22. or/19‐21 23. Factor VIIa/ 24. (factor viia or factor 7a or rfviia or fviia or novoseven* or novo seven* or aryoseven or acset or eptacog* or proconvertin).tw,kf. 25. ((activated adj2 factor seven) or (activated adj2 factor vii) or (activated adj3 rfvii) or (activated adj2 fvii)).tw,kf. 26. (factor seven or factor vii or factor 7).ti. 27. 23 or 24 or 25 or 26 28. Fibrinogen/ad, ae, de, sd, tu, th, to 29. *Fibrinogen/ 30. (fibrinogen concentrate* or factor I or Haemocomplettan* or Riastap* or Fibryga* or Fibryna*).tw,kf. 31. 28 or 29 or 30 32. Deamino Arginine Vasopressin/ 33. (desmopressin* or vasopressin deamino or D‐amino D‐arginine vasopressin or deamino‐8‐d‐arginine vasopressin or vasopressin 1‐desamino‐8‐arginine or desmotabs or DDAVP or desmogalen or adin or adiuretin or concentraid or d‐void or dav ritter or deamino 8 dextro arginine vasopressin or deamino 8d arginine vasopressin or deamino dextro arginine vasopressin or deaminovasopressin or defirin or defirin melt or desmirin or desmomelt or desmopresina or desmospray or desmotab* or desurin or emosint or enupresol or minirin or minirinette or minirinmelt or minrin or minurin or miram or nictur or noctisson or nocturin or nocutil or nordurine or novidin or nucotil or octim or octostim or presinex or stimate or wetirin).tw,kf. 34. 32 or 33 35. exp Factor XIII/ 36. (factor XIII or fXIII or fibrin stabili?ing factor* or Tretten* or Catridecacog).tw,kf. 37. 35 or 36 38. exp Tissue Adhesives/ 39. *Adhesives/ 40. Collagen/tu 41. Thrombin/tu 42. Gelatin/tu 43. Gelatin Sponge, Absorbable/ 44. ((fibrin* or collagen or cellulose or gelatin or gel or thrombin* or albumin or hemostatic* or haemostatic*) adj3 (glu* or seal* or adhesive* or topical* or local* or matrix or matrices or spong* or fleece* or foam* or scaffold* or patch* or sheet* or bandag* or aerosol* or dressing* or paste or powder*)).tw,kf. 45. ((nonfibrin* or non‐fibrin* or synthetic* or non‐biological* or nonbiological* or biological*) adj3 (glue* or seal* or adhesive*)).tw,kf. 46. (surgical* adj3 (glue* or sealant* or adhesive*)).tw,kf. 47. ((fibrin* or collagen or cellulose or gelatin or thrombin) adj3 (hemosta* or haemosta*)).tw,kf. 48. (8Y or Aafact or Actif‐VIII or Advate or Artiss or Bioglue or Biocol or Collaseal or Omrixil or Transglutine or Raplixa or Evarrest or Aleviate or Alphanate or Amofil or Beriate or Beriplast or Biostate or Bolheal or Cluvot or Conco‐Eight‐HT or Crosseel or Crosseal or Crosseight or Emoclot or Evarrest or Evicel or Factane or Fanhdi or Fibrogammin P or Green VIII or Green VIII Factor or Greengene or Greenmono or Greenplast or Haemate or Haemate P or Haemate P or Haemate P500 or Haemate‐P or Haemoctin or Haemoctin SDH or Haemoctin‐SDH or Hemaseel or Hemaseal or Hemofil M or Hemoraas or Humaclot or Humafactor‐8 or Humate‐P or Immunate or Innovate or Koate or Koate‐DVI or Kogenate Bayer or Kogenate FS or Monoclate‐P or NovoThirteen or Octafil or Octanate or Octanate or Optivate or Quixil or Talate or Tisseel or Tisseal or Tissel or Tissucol or Tricos or Vivostat or Voncento or Wilate or Wilnativ or Wilstart or Xyntha).tw,kf. 49. (Glubran or Gluetiss or Ifabond or Indermil or LiquiBand or TissuGlu).tw,kf. 50. (Evithrom or Floseal or Hemopatch or Gel‐Flow or Gelfoam or Gelfilm or Recothrom or Surgifoam or Surgiflo* or "rh Thrombin" or Thrombi‐Gel or Thrombi‐Pad or Thrombin‐JMI or Thrombinar or Thrombogen or Thrombostat).tw,kf. 51. (porcine gelatin or bovine collagen or bovine gelatin or nu‐knit or arista or hemostase or vita sure or thrombin‐jmi or thrombinjmi or avicel or vivagel or lyostypt or tabotamp or arterx or omnex or veriset).tw,kf. 52. (polysaccharide adj (sphere* or hemostatic powder)).tw,kf. 53. *Chitosan/ or (chitosan* or mucoadhesive or muco‐adhesive or Celox Gauze or ChitoGauze or Combat Gauze).tw,kf. 54. *Polyethylene Glycols/ 55. *Hydrogel, Polyethylene Glycol Dimethacrylate/ 56. Polyurethanes/ad, ae, pd, tu, to 57. ((polymer‐derived elastic* or polymer tissue adhesive* or elastic hydrogel* or glutaraldehyde or PEG‐based or polyurethane‐based tissue or polyethylene glycol* or polyvinyl alcohol‐based tissue or PVA‐based tissue or natural biopolymer* or polypeptide‐based or protein‐based or polysaccharide‐based or chitosan or poliglusam or cyanoacrylic or cyanoacrylate or cyacrin or dextran‐based or chondroitin sulfate‐based or mussel‐inspired elastic* or glycol hydrogel or polymer‐based) adj3 (glu* or seal* or adhesive* or topical* or local* or matrix or matrices or spong* or fleece* or foam* or scaffold* or patch* or sheet* or bandag* or aerosol* or dressing* or paste* or powder*)).tw,kf. 58. Cellulose, Oxidized/ 59. (absorbable cellulose or resorbable cellulose or oxidi?ed cellulose or carboxycellulose or oxycellulose or cellulosic acid or oxycel or oxidi?ed regenerated cellulose).tw,kf. 60. (BioGlue or Progel or Duraseal or Coseal or FocalSeal or ADAL‐1 or AdvaSeal or Pleuraseal or Angio‐Seal or Avitene or Instat or Helitene or Helistat or TDM‐621 or Dermabond or Tissueseal or PolyStat or Raplixa or Spongostan or Surgicel or Surgilux or Tachosil or Traumstem).tw,kf. 61. (collagen‐thrombin or thrombin‐collagen or gelatin‐fibrinogen or fibrinogen‐gelatin or gelatin‐thrombin or thrombin‐gelatin or fibrinogen‐thrombin or thrombin‐fibrinogen or collagen‐fibrinogen or fibrinogen‐collagen or microfibrillar collagen or CoStasis or "GRF Glue" or GR‐Dial or Algosterile or TraumaStat or HemCon or ChitoFlex or Celox or QuikClot or WoundStat or Vitagel or TachSeal or TachoComb or Cryoseal).tw,kf. 62. or/38‐61 63. exp Waxes/ 64. (bonewax* or bone wax* or bone putty or hemasorb or ostene).tw,kf. 65. 63 or 64 66. Hemostatics/ad, th, tu 67. (((haemosta* or hemosta* or antihaemorrhag* or antihemorrhag* or anti haemorrhag* or anti‐hemorrhag*) adj5 (drug* or agent* or treat* or therap* or dressing*)) or ((coagulat* or clotting) adj factor*)).tw,kf. 68. 18 or 22 or 27 or 31 or 34 or 37 or 62 or 65 or 66 or 67 69. 11 and 68 70. Meta‐Analysis.pt. 71. ((meta analy* or metaanaly*) and (trials or studies)).ab. 72. (meta analy* or metaanaly* or evidence‐based).ti. 73. ((systematic* or evidence‐based) adj2 (review* or overview*)).tw,kf. 74. (evidence synthes* or cochrane or medline or pubmed or embase or cinahl or cinhal or lilacs or "web of science" or science citation index or scopus or search terms or literature search or electronic search* or comprehensive search* or systematic search* or published articles or search strateg* or reference list* or bibliograph* or handsearch* or hand search* or manual* search*).ab. 75. Cochrane Database of systematic reviews.jn. 76. (additional adj (papers or articles or sources)).ab. 77. ((electronic* or online) adj (sources or resources or databases)).ab. 78. (relevant adj (journals or articles)).ab. 79. or/70‐78 80. Review.pt. 81. exp Randomized Controlled Trials as Topic/ 82. selection criteria.ab. or critical appraisal.tw. 83. (data adj (abstract* or extract* or analys*)).ab. 84. exp Randomized Controlled Trial/ 85. or/81‐84 86. 80 and 85 87. 79 or 86 88. Randomized Controlled Trial.pt. 89. Controlled Clinical Trial.pt. 90. (placebo or randomly or groups).ab. 91. (randomi* or trial).tw,kf. 92. exp Clinical Trial as Topic/ 93. 87 or 88 or 89 or 90 or 91 or 92 94. exp animals/ not humans/ 95. 93 not 94 96. 69 and 95

#### Embase (Ovid)

1. exp Hip Surgery/ 2. exp Hip Disease/su 3. exp Knee Surgery/ 4. exp Knee Disease/su 5. ((hip* or knee* or femur or femoral) adj10 (replac* or operat* or surg* or arthroplast* or reconstruct* or repair* or osteotom* or alloplast* or plast* or resurfac* or implant* or prosthes*)).tw. 6. (acetabuloplast* or acetabulum arthroplast*).tw. 7. or/1‐6 8. Antifibrinolytic Agent/ 9. Tranexamic Acid/ 10. Aminocaproic Acid/ 11. (antifibrinolytic* or anti‐fibrinolytic* or antifibrinolysin* or antiplasmin* or plasmin inhibitor* or tranexamic or tranhexamic or cyclohexanecarboxylic acid* or amcha or trans‐4‐aminomethyl‐cyclohexanecarboxylic acid* or t‐amcha or amca or "kabi 2161" or transamin or amchafibrin or anvitoff or spotof or cyklokapron or cyclo‐F or femstrual or ugurol or aminomethylcyclohexanecarbonic acid or aminomethylcyclohexanecarboxylic acid or AMCHA or amchafibrin or amikapron or aminomethyl cyclohexane carboxylic acid or aminomethyl cyclohexanecarboxylic acid or aminomethylcyclohexane carbonic acid or aminomethylcyclohexane carboxylic acid or aminomethylcyclohexanecarbonic acid or aminomethylcyclohexanecarboxylic acid or aminomethylcyclohexanocarboxylic acid or aminomethylcyclohexanoic acid or amstat or antivoff or caprilon or cl?65336 or cl65336 or cyclocapron or cyclokapron or cyklocapron or cyklokapron or exacyl or frenolyse or fibrinon or hemostan or hexacapron or hexakapron or kalnex or lysteda or rikaparin or ronex or theranex or tranexam or tranexanic or tranexic or trans achma or transexamic or trenaxin or TXA or (fibrinolysis adj2 inhibitor*)).tw. 12. (Agretax or Bio‐Stat or Capiloc or Capitrax or Clip Inj or Clot‐XL or Clotawin‐T or Coastat or Cuti or Cymin or Dubatran or Espercil or Examic or Existat or Extam or Fibran or Gynae‐Pil or Hemstate or Kapron or Menogia or Monitex or Nestran or Nexamic or Nexi‐500 or Nexmeff or Nicolda or Nixa‐500 or Pause or Rheonex or Sylstep TX or Synostat or T‐nex or T Stat or T Stat or Tanmic or Temsyl‐T or Texakind or Texanis or Texapar or Texid or Thams or Tonopan or Traklot or Tramic or Tramix or Tranarest or Trance Inj or Tranecid or Tranee or Tranemic or Tranex or Tranexa or Tranfib or Tranlok or Transtat or Transys or Transcam or Tranxi or Trapic or Traxage or Traxamic or Traxyl or Trenaxa or Trexamic or Trim Inj or Tx‐1000 or Tx 500 or Wistran or X‐Tran or Xamic).tw. 13. (6‐aminohexanoic or amino?caproic or amino?hexanoic or amino caproic or amino‐caproic or amino‐n‐hexanoic or cy‐116 or cy116 or lederle or acikaprin or afibrin or amicar or caprocid or capracid or capramol or caprogel or caprolest or caprolisin* or caprolysin* or capromol or epsikapron or hemocaprol or caproamin or EACA or caprolest or capralense or hexalense or hamostat or hemocid or cl 10304 or cl10304 or ecapron or ekaprol or epsamon or epsicaprom or epsicapron or epsilcapramin or epsilon amino caproate or epsilon aminocaproate or epsilonaminocaproic or epsilonaminocapronsav or etha?aminocaproic or ethaaminocaproich or emocaprol or hepin or ipsilon or jd?177or neocaprol or nsc?26154 or resplamin or tachostyptan).tw. 14. or/8‐13 15. Aprotinin/ 16. (antagosan or antilysin* or aprotimbin or apronitin* or aprotinin* or bayer a128 or contrical or contrycal or contrykal or dilmintal or frey inhibitor or kontrycal or Kunitz inhibitor or gordox or haemoprot or kallikrein‐trypsin inactivator).tw. 17. (iniprol or kontrikal or kontrykal or kunitz pancreatic trypsin inhibitor or midran or pulmin or tracylol or trascolan or trasilol or tra?ylol or traskolan or zymofren or pancreas antitrypsin or protinin or riker 52g or Rivilina zymofren).tw. 18. or/15‐17 19. Blood Clotting Factor 7a/ 20. (factor viia or factor 7a or rfviia or fviia or novoseven* or novo seven* or aryoseven or acset or eptacog* or proconvertin).tw. 21. ((activated adj2 factor seven) or (activated adj2 factor vii) or (activated adj3 rfvii) or (activated adj2 fvii)).tw. 22. (factor seven or factor vii or factor 7).ti. 23. 19 or 20 or 21 or 22 24. Fibrinogen Concentrate/ 25. (fibrinogen concentrate* or factor I or Haemocomplettan* or Riastap* or Fibryga* or Fibryna*).tw. 26. 24 or 25 27. Desmopressin/ 28. (desmopressin* or vasopressin deamino or D‐amino D‐arginine vasopressin or deamino‐8‐d‐arginine vasopressin or vasopressin 1‐desamino‐8‐arginine or desmotabs or DDAVP or desmogalen or adin or adiuretin or concentraid or d‐void or dav ritter or deamino 8 dextro arginine vasopressin or deamino 8d arginine vasopressin or deamino dextro arginine vasopressin or deaminovasopressin or defirin or defirin melt or desmirin or desmomelt or desmopresina or desmospray or desmotab* or desurin or emosint or enupresol or minirin or minirinette or minirinmelt or minrin or minurin or miram or nictur or noctisson or nocturin or nocutil or nordurine or novidin or nucotil or octim or octostim or presinex or stimate or wetirin).tw. 29. 27 or 28 30. Blood Clotting Factor 13/ 31. (factor xiii or fxiii or fibrin stabili?ing factor* or Tretten* or Catridecacog).tw. 32. 30 or 31 33. exp Tissue Adhesive/ 34. *Adhesive Agent/ 35. *Hemostatic Agent/ 36. ((fibrin* or collagen or cellulose or gelatin or gel or thrombin* or albumin or hemostatic* or haemostatic*) adj3 (glu* or seal* or adhesive* or topical* or local* or matrix or matrices or spong* or fleece* or foam* or scaffold* or patch* or sheet* or bandag* or aerosol* or dressing* or paste or powder*)).tw. 37. ((nonfibrin* or non‐fibrin* or synthetic* or non‐biological* or nonbiological* or biological*) adj3 (glue* or seal* or adhesive*)).tw. 38. (surgical* adj3 (glue* or sealant* or adhesive*)).tw. 39. ((fibrin* or collagen or cellulose or gelatin or thrombin) adj3 (hemosta* or haemosta*)).tw. 40. (8Y or Aafact or Actif‐VIII or Advate or Artiss or Raplixa or Evarrest or Aleviate or Alphanate or Amofil or Beriate or Beriplast or Biostate or Bolheal or Cluvot or Conco‐Eight‐HT or Crosseel or Crosseal or Crosseight or Emoclot or Evarrest or Evicel or Factane or Fanhdi or Fibrogammin P or Green VIII or Green VIII Factor or Greengene or Greenmono or Greenplast or Haemate or Haemate P or Haemate P or Haemate P500 or Haemate‐P or Haemoctin or Haemoctin SDH or Haemoctin‐SDH or Hemaseel or Hemaseal or Hemofil M or Hemoraas or Humaclot or Humafactor‐8 or Humate‐P or Immunate or Innovate or Koate or Koate‐DVI or Kogenate Bayer or Kogenate FS or Monoclate‐P or NovoThirteen or Octafil or Octanate or Octanate or Optivate or Quixil or Talate or Tisseel or Tisseal or Tissel or Tissucol or Tricos or Vivostat or Voncento or Wilate or Wilnativ or Wilstart or Xyntha).tw. 41. (Glubran or Gluetiss or Ifabond or Indermil or LiquiBand or TissuGlu).tw. 42. Collagen Sponge/ or Collagen Dressing/ 43. Gelatin Sponge/ or Gelfoam/ 44. (Evithrom or Floseal or Hemopatch or Gel‐Flow or Gelfoam or Gelfilm or Recothrom or Surgifoam or Surgiflo* or "rh Thrombin" or Thrombi‐Gel or Thrombi‐Pad or Thrombin‐JMI or Thrombinar or Thrombogen or Thrombostat).tw. 45. *Chitosan/ or (chitosan* or mucoadhesive or muco‐adhesive or Celox Gauze or ChitoGauze or Combat Gauze).tw. 46. Hydrogel Dressing/ 47. Fibrinogen plus Thrombin/ 48. Polyvinyl Alcohol Sponge/ 49. (porcine gelatin or bovine collagen or bovine gelatin or nu‐knit or arista or hemostase or vita sure or thrombin‐jmi or thrombinjmi or avicel or vivagel or lyostypt or tabotamp or arterx or omnex or veriset).tw. 50. (polysaccharide adj (sphere* or hemostatic powder)).tw. 51. ((polymer‐derived elastic* or polymer tissue adhesive* or elastic hydrogel* or glutaraldehyde or PEG‐based or polyurethane‐based tissue or polyethylene glycol* or polyvinyl alcohol‐based tissue or PVA‐based tissue or natural biopolymer* or polypeptide‐based or protein‐based or polysaccharide‐based or chitosan or poliglusam or cyanoacrylic or cyanoacrylate or cyacrin or dextran‐based or chondroitin sulfate‐based or mussel‐inspired elastic* or glycol hydrogel or polymer‐based) adj3 (glu* or seal* or adhesive* or topical* or local* or matrix or matrices or spong* or fleece* or foam* or scaffold* or patch* or sheet* or bandag* or aerosol* or dressing* or paste* or powder*)).tw. 52. Oxidized Cellulose/ 53. Oxidized Regenerated Cellulose/ 54. Recombinant Thrombin/ 55. Tachocomb/ 56. (absorbable cellulose or resorbable cellulose or oxidi?ed cellulose or carboxycellulose or oxycellulose or cellulosic acid or oxycel or oxidi?ed regenerated cellulose).tw. 57. (BioGlue or Progel or Duraseal or Coseal or FocalSeal or ADAL‐1 or AdvaSeal or Pleuraseal or Angio‐Seal or Avitene or Instat or Helitene or Helistat or TDM‐621 or Dermabond or Tissueseal or PolyStat or Raplixa or Spongostan or Surgicel).tw. 58. (Tachosil or Traumstem or CoStasis or "GRF Glue" or GR‐Dial or Algosterile or TraumaStat or HemCon or ChitoFlex or Celox or QuikClot or WoundStat or Vitagel or TachSeal or TachoComb or Cryoseal).tw. 59. (collagen‐thrombin or thrombin‐collagen or gelatin‐fibrinogen or fibrinogen‐gelatin or gelatin‐thrombin or thrombin‐gelatin or fibrinogen‐thrombin or thrombin‐fibrinogen or collagen‐fibrinogen or fibrinogen‐collagen or microfibrillar collagen).tw. 60. or/33‐59 61. Bone Wax/ 62. (bonewax* or bone wax* or bone putty or hemasorb or ostene).tw. 63. or/61‐62 64. Hemostatic Agent/ 65. (((haemosta* or hemosta* or antihaemorrhag* or antihemorrhag* or anti haemorrhag* or anti‐hemorrhag*) adj5 (drug* or agent* or treat* or therap* or dressing*)) or ((coagulat* or clotting) adj factor*)).tw. 66. 14 or 18 or 23 or 26 or 29 or 32 or 60 or 63 or 64 or 65 67. 7 and 66 68. Meta Analysis/ 69. (meta analy* or metaanaly* or evidence‐based).ti. 70. ((meta analy* or metaanaly*) and (trials or studies)).ab. 71. Systematic Review/ 72. ((systematic* or evidence‐based) adj2 (review* or overview*)).tw. 73. (evidence synthes* or cochrane or medline or pubmed or embase or cinahl or cinhal or lilacs or "web of science" or science citation index or scopus or search terms or literature search or electronic search* or comprehensive search* or systematic search* or published articles or search strateg* or reference list* or bibliograph* or handsearch* or hand search* or manual* search*).ab. 74. ((electronic* or online) adj (sources or resources or databases)).ab. 75. ((additional adj (papers or articles or sources)) or (relevant adj (journals or articles))).ab. 76. or/68‐75 77. Review.pt. 78. (data extraction or selection criteria).ab. 79. 77 and 78 80. 76 or 79 81. Editorial.pt. 82. 80 not 81 83. crossover‐procedure/ or double‐blind procedure/ or randomized controlled trial/ or single‐blind procedure/ 84. (random* or factorial* or crossover* or cross over* or cross‐over* or placebo* or doubl* blind* or singl* blind* or assign* or allocat* or volunteer*).mp. 85. 83 or 84 86. 82 or 85 87. 67 and 86

#### CINAHL (EBSCO*host*)

S1 (MH "Arthroplasty, Replacement, Hip") S2 (MH "Osteoarthritis, Hip") S3 (MH "Arthroplasty, Replacement, Knee+") S4 (MH "Knee Injuries+/SU") S5 (MH "Knee/SU") OR (MH "Hip/SU") S6 (MH "Osteoarthritis, Knee/SU") S7 TI ( ((hip* or knee* or femur or femoral) N10 (replac* or operat* or surg* or arthroplast* or reconstruct* or repair* or osteotom* or alloplast* or plast* or resurfac* or implant* or prosthes*)) ) OR AB ( ((hip* or knee* or femur or femoral) N10 (replac* or operat* or surg* or arthroplast* or reconstruct* or repair* or osteotom* or alloplast* or plast* or resurfac* or implant* or prosthes*)) ) S8 TI ( (acetabuloplast* or acetabulum arthroplast*) ) OR AB ( (acetabuloplast* or acetabulum arthroplast*) ) OR TI ( (THA or THR or TKA or TKR or UKA or UKR) ) OR AB ( (THA or THR or TKA or TKR or UKA or UKR) ) S9 S1 OR S2 OR S3 OR S4 OR S5 OR S6 OR S7 OR S8 S10 (MH "Antifibrinolytic Agents") OR (MH "Aminocaproic Acids") OR (MH "Tranexamic Acid") S11 TI ( (antifibrinolytic* or anti‐fibrinolytic* or antifibrinolysin* or antiplasmin* or plasmin inhibitor* or tranexamic or tranhexamic or cyclohexanecarboxylic acid* or amcha or trans‐4‐aminomethyl‐cyclohexanecarboxylic acid* or t‐amcha or amca or "kabi 2161" or transamin or amchafibrin or anvitoff or spotof or cyklokapron or cyclo‐F or femstrual or ugurol or aminomethylcyclohexanecarbonic acid or aminomethylcyclohexanecarboxylic acid or AMCHA or amchafibrin or amikapron or aminomethyl cyclohexane carboxylic acid or aminomethyl cyclohexanecarboxylic acid or aminomethylcyclohexane carbonic acid or aminomethylcyclohexane carboxylic acid or aminomethylcyclohexanecarbonic acid or aminomethylcyclohexanecarboxylic acid or aminomethylcyclohexanocarboxylic acid or aminomethylcyclohexanoic acid or amstat or antivoff or caprilon or cl?65336 or cl65336 or cyclocapron or cyclokapron or cyklocapron or cyklokapron or exacyl or frenolyse or fibrinon or hemostan or hexacapron or hexakapron or kalnex or lysteda or rikaparin or ronex or theranex or tranexam or tranexanic or tranexic or trans achma or transexamic or trenaxin or TXA or (fibrinolysis N2 inhibitor*)) ) OR AB ( (antifibrinolytic* or anti‐fibrinolytic* or antifibrinolysin* or antiplasmin* or plasmin inhibitor* or tranexamic or tranhexamic or cyclohexanecarboxylic acid* or amcha or trans‐4‐aminomethyl‐cyclohexanecarboxylic acid* or t‐amcha or amca or "kabi 2161" or transamin or amchafibrin or anvitoff or spotof or cyklokapron or cyclo‐F or femstrual or ugurol or aminomethylcyclohexanecarbonic acid or aminomethylcyclohexanecarboxylic acid or AMCHA or amchafibrin or amikapron or aminomethyl cyclohexane carboxylic acid or aminomethyl cyclohexanecarboxylic acid or aminomethylcyclohexane carbonic acid or aminomethylcyclohexane carboxylic acid or aminomethylcyclohexanecarbonic acid or aminomethylcyclohexanecarboxylic acid or aminomethylcyclohexanocarboxylic acid or aminomethylcyclohexanoic acid or amstat or antivoff or caprilon or cl?65336 or cl65336 or cyclocapron or cyclokapron or cyklocapron or cyklokapron or exacyl or frenolyse or fibrinon or hemostan or hexacapron or hexakapron or kalnex or lysteda or rikaparin or ronex or theranex or tranexam or tranexanic or tranexic or trans achma or transexamic or trenaxin or TXA or (fibrinolysis N2 inhibitor*)) ) S12 TI ( (6‐aminohexanoic or amino?caproic or amino?hexanoic or amino caproic or amino‐caproic or amino‐n‐hexanoic or cy‐116 or cy116 or lederle or acikaprin or afibrin or amicar or caprocid or capracid or capramol or caprogel or caprolest or caprolisin* or caprolysin* or capromol or epsikapron or hemocaprol or caproamin or EACA or caprolest or capralense or hexalense or hamostat or hemocid or cl 10304 or cl10304 or ecapron or ekaprol or epsamon or epsicaprom or epsicapron or epsilcapramin or epsilon amino caproate or epsilon aminocaproate or epsilonaminocaproic or epsilonaminocapronsav or etha?aminocaproic or ethaaminocaproich or emocaprol or hepin or ipsilon or jd?177or neocaprol or nsc?26154 or resplamin or tachostyptan) ) OR AB ( (6‐aminohexanoic or amino?caproic or amino?hexanoic or amino caproic or amino‐caproic or amino‐n‐hexanoic or cy‐116 or cy116 or lederle or acikaprin or afibrin or amicar or caprocid or capracid or capramol or caprogel or caprolest or caprolisin* or caprolysin* or capromol or epsikapron or hemocaprol or caproamin or EACA or caprolest or capralense or hexalense or hamostat or hemocid or cl 10304 or cl10304 or ecapron or ekaprol or epsamon or epsicaprom or epsicapron or epsilcapramin or epsilon amino caproate or epsilon aminocaproate or epsilonaminocaproic or epsilonaminocapronsav or etha?aminocaproic or ethaaminocaproich or emocaprol or hepin or ipsilon or jd?177or neocaprol or nsc?26154 or resplamin or tachostyptan) ) S13 S10 OR S11 OR S12 S14 (MH "Aprotinin") S15 TI ( (antagosan or antilysin* or aprotimbin or apronitin* or aprotinin* or bayer a128 or contrical or contrycal or contrykal or dilmintal or frey inhibitor or kontrycal or Kunitz inhibitor or gordox or haemoprot or kallikrein‐trypsin inactivator) ) OR AB ( (antagosan or antilysin* or aprotimbin or apronitin* or aprotinin* or bayer a128 or contrical or contrycal or contrykal or dilmintal or frey inhibitor or kontrycal or Kunitz inhibitor or gordox or haemoprot or kallikrein‐trypsin inactivator) ) S16 TI ( (iniprol or kontrikal or kontrykal or kunitz pancreatic trypsin inhibitor or midran or pulmin or tracylol or trascolan or trasilol or tra?ylol or traskolan or zymofren or pancreas antitrypsin or protinin or riker 52g or Rivilina zymofren) ) OR AB ( (iniprol or kontrikal or kontrykal or kunitz pancreatic trypsin inhibitor or midran or pulmin or tracylol or trascolan or trasilol or tra?ylol or traskolan or zymofren or pancreas antitrypsin or protinin or riker 52g or Rivilina zymofren) ) S17 S14 OR S15 OR S16 S18 TX ( (factor viia or factor 7a or rfviia or fviia or novoseven* or novo seven* or aryoseven or acset or eptacog* or proconvertin) ) OR TX ( ((activated N2 factor seven) or (activated N2 factor vii) or (activated N3 rfvii) or (activated N2 fvii)) ) S19 TX (factor seven or factor vii or factor 7) S20 S18 OR S19 S21 (MH "Fibrinogen") S22 TX (fibrinogen concentrate* or factor I or Haemocomplettan* or Riastap* or Fibryga* or Fibryna*) S23 S21 OR S22 S24 (MH "Desmopressin") S25 TI ( (desmopressin* or vasopressin deamino or D‐amino D‐arginine vasopressin or deamino‐8‐d‐arginine vasopressin or vasopressin 1‐desamino‐8‐arginine or desmotabs or DDAVP or desmogalen or adin or adiuretin or concentraid or d‐void or dav ritter or deamino 8 dextro arginine vasopressin or deamino 8d arginine vasopressin or deamino dextro arginine vasopressin or deaminovasopressin or defirin or defirin melt or desmirin or desmomelt or desmopresina or desmospray or desmotab* or desurin or emosint or enupresol or minirin or minirinette or minirinmelt or minrin or minurin or miram or nictur or noctisson or nocturin or nocutil or nordurine or novidin or nucotil or octim or octostim or presinex or stimate or wetirin) ) OR AB ( (desmopressin* or vasopressin deamino or D‐amino D‐arginine vasopressin or deamino‐8‐d‐arginine vasopressin or vasopressin 1‐desamino‐8‐arginine or desmotabs or DDAVP or desmogalen or adin or adiuretin or concentraid or d‐void or dav ritter or deamino 8 dextro arginine vasopressin or deamino 8d arginine vasopressin or deamino dextro arginine vasopressin or deaminovasopressin or defirin or defirin melt or desmirin or desmomelt or desmopresina or desmospray or desmotab* or desurin or emosint or enupresol or minirin or minirinette or minirinmelt or minrin or minurin or miram or nictur or noctisson or nocturin or nocutil or nordurine or novidin or nucotil or octim or octostim or presinex or stimate or wetirin) ) S26 S24 OR S25 S27 TX (factor XIII or fXIII or fibrin stabili?ing factor* or Tretten* or Catridecacog) S28 (MH "Tissue Adhesives") S29 (MH "Fibrin Tissue Adhesive") S30 (MH "Collagen/TU") S31 (MH "Thrombin/TU") S32 (MH "Surgical Sponges") S33 TI ( ((fibrin* or collagen or cellulose or gelatin or gel or thrombin* or albumin or hemostatic* or haemostatic*) N3 (glu* or seal* or adhesive* or topical* or local* or matrix or matrices or spong* or fleece* or foam* or scaffold* or patch* or sheet* or bandag* or aerosol* or dressing* or paste or powder*)) ) OR AB ( ((fibrin* or collagen or cellulose or gelatin or gel or thrombin* or albumin or hemostatic* or haemostatic*) N3 (glu* or seal* or adhesive* or topical* or local* or matrix or matrices or spong* or fleece* or foam* or scaffold* or patch* or sheet* or bandag* or aerosol* or dressing* or paste or powder*)) ) S34 TI ( ((nonfibrin* or non‐fibrin* or synthetic* or non‐biological* or nonbiological* or biological*) N3 (glue* or seal* or adhesive*)) ) OR AB ( ((nonfibrin* or non‐fibrin* or synthetic* or non‐biological* or nonbiological* or biological*) N3 (glue* or seal* or adhesive*)) ) S35 TI ( (surgical* N3 (glue* or sealant* or adhesive*)) ) OR AB ( (surgical* N3 (glue* or sealant* or adhesive*)) ) S36 TI ( ((fibrin* or collagen or cellulose or gelatin or thrombin) N3 (hemosta* or haemosta*)) ) OR AB ( ((fibrin* or collagen or cellulose or gelatin or thrombin) N3 (hemosta* or haemosta*)) ) S37 TI ( (8Y or Aafact or Actif‐VIII or Advate or Artiss or Bioglue or Biocol or Collaseal or Omrixil or Transglutine or Raplixa or Evarrest or Aleviate or Alphanate or Amofil or Beriate or Beriplast or Biostate or Bolheal or Cluvot or Conco‐Eight‐HT or Crosseel or Crosseal or Crosseight or Emoclot or Evarrest or Evicel or Factane or Fanhdi or Fibrogammin P or Green VIII or Green VIII Factor or Greengene or Greenmono or Greenplast or Haemate or Haemate P or Haemate P or Haemate P500 or Haemate‐P or Haemoctin or Haemoctin SDH or Haemoctin‐SDH or Hemaseel or Hemaseal or Hemofil M or Hemoraas or Humaclot or Humafactor‐8 or Humate‐P or Immunate or Innovate or Koate or Koate‐DVI or Kogenate Bayer or Kogenate FS or Monoclate‐P or NovoThirteen or Octafil or Octanate or Octanate or Optivate or Quixil or Talate or Tisseel or Tisseal or Tissel or Tissucol or Tricos or Vivostat or Voncento or Wilate or Wilnativ or Wilstart or Xyntha) ) OR AB ( (8Y or Aafact or Actif‐VIII or Advate or Artiss or Bioglue or Biocol or Collaseal or Omrixil or Transglutine or Raplixa or Evarrest or Aleviate or Alphanate or Amofil or Beriate or Beriplast or Biostate or Bolheal or Cluvot or Conco‐Eight‐HT or Crosseel or Crosseal or Crosseight or Emoclot or Evarrest or Evicel or Factane or Fanhdi or Fibrogammin P or Green VIII or Green VIII Factor or Greengene or Greenmono or Greenplast or Haemate or Haemate P or Haemate P or Haemate P500 or Haemate‐P or Haemoctin or Haemoctin SDH or Haemoctin‐SDH or Hemaseel or Hemaseal or Hemofil M or Hemoraas or Humaclot or Humafactor‐8 or Humate‐P or Immunate or Innovate or Koate or Koate‐DVI or Kogenate Bayer or Kogenate FS or Monoclate‐P or NovoThirteen or Octafil or Octanate or Octanate or Optivate or Quixil or Talate or Tisseel or Tisseal or Tissel or Tissucol or Tricos or Vivostat or Voncento or Wilate or Wilnativ or Wilstart or Xyntha) ) S38 TI ( (Glubran or Gluetiss or Ifabond or Indermil or LiquiBand or TissuGlu) ) OR AB ( (Glubran or Gluetiss or Ifabond or Indermil or LiquiBand or TissuGlu) ) S39 TI ( (Evithrom or Floseal or Hemopatch or Gel‐Flow or Gelfoam or Gelfilm or Recothrom or Surgifoam or Surgiflo* or "rh Thrombin" or Thrombi‐Gel or Thrombi‐Pad or Thrombin‐JMI or Thrombinar or Thrombogen or Thrombostat) ) OR AB ( (Evithrom or Floseal or Hemopatch or Gel‐Flow or Gelfoam or Gelfilm or Recothrom or Surgifoam or Surgiflo* or "rh Thrombin" or Thrombi‐Gel or Thrombi‐Pad or Thrombin‐JMI or Thrombinar or Thrombogen or Thrombostat) ) S40 TI ( (porcine gelatin or bovine collagen or bovine gelatin or nu‐knit or arista or hemostase or vita sure or thrombin‐jmi or thrombinjmi or avicel or vivagel or lyostypt or tabotamp or arterx or omnex or veriset) ) OR AB ( (porcine gelatin or bovine collagen or bovine gelatin or nu‐knit or arista or hemostase or vita sure or thrombin‐jmi or thrombinjmi or avicel or vivagel or lyostypt or tabotamp or arterx or omnex or veriset) ) S41 TX (polysaccharide NEXT (sphere* or hemostatic powder)) S42 (MM "Polyethylene Glycols") S43 (MH "Hydrogel Dressings") S44 (MH "Polyurethanes/AD/AE/TU/ST/DE") S45 TI ( ((polymer‐derived elastic* or polymer tissue adhesive* or elastic hydrogel* or glutaraldehyde or PEG‐based or polyurethane‐based tissue or polyethylene glycol* or polyvinyl alcohol‐based tissue or PVA‐based tissue or natural biopolymer* or polypeptide‐based or protein‐based or polysaccharide‐based or chitosan or poliglusam or cyanoacrylic or cyanoacrylate or cyacrin or dextran‐based or chondroitin sulfate‐based or mussel‐inspired elastic* or glycol hydrogel or polymer‐based) N3 (glu* or seal* or adhesive* or topical* or local* or matrix or matrices or spong* or fleece* or foam* or scaffold* or patch* or sheet* or bandag* or aerosol* or dressing* or paste* or powder*)) ) OR AB ( ((polymer‐derived elastic* or polymer tissue adhesive* or elastic hydrogel* or glutaraldehyde or PEG‐based or polyurethane‐based tissue or polyethylene glycol* or polyvinyl alcohol‐based tissue or PVA‐based tissue or natural biopolymer* or polypeptide‐based or protein‐based or polysaccharide‐based or chitosan or poliglusam or cyanoacrylic or cyanoacrylate or cyacrin or dextran‐based or chondroitin sulfate‐based or mussel‐inspired elastic* or glycol hydrogel or polymer‐based) N3 (glu* or seal* or adhesive* or topical* or local* or matrix or matrices or spong* or fleece* or foam* or scaffold* or patch* or sheet* or bandag* or aerosol* or dressing* or paste* or powder*)) ) S46 (MH "Cellulose/TU") S47 TI ( (absorbable cellulose or resorbable cellulose or oxidi?ed cellulose or carboxycellulose or oxycellulose or cellulosic acid or oxycel or oxidi?ed regenerated cellulose) ) OR AB ( (absorbable cellulose or resorbable cellulose or oxidi?ed cellulose or carboxycellulose or oxycellulose or cellulosic acid or oxycel or oxidi?ed regenerated cellulose) ) S48 TI ( (BioGlue or Progel or Duraseal or Coseal or FocalSeal or ADAL‐1 or AdvaSeal or Pleuraseal or Angio‐Seal or Avitene or Instat or Helitene or Helistat or TDM‐621 or Dermabond or Tissueseal or PolyStat or Raplixa or Spongostan or Surgicel or Surgilux or Tachosil or Traumstem) ) OR AB ( (BioGlue or Progel or Duraseal or Coseal or FocalSeal or ADAL‐1 or AdvaSeal or Pleuraseal or Angio‐Seal or Avitene or Instat or Helitene or Helistat or TDM‐621 or Dermabond or Tissueseal or PolyStat or Raplixa or Spongostan or Surgicel or Surgilux or Tachosil or Traumstem) ) S49 TI ( (collagen‐thrombin or thrombin‐collagen or gelatin‐fibrinogen or fibrinogen‐gelatin or gelatin‐thrombin or thrombin‐gelatin or fibrinogen‐thrombin or thrombin‐fibrinogen or collagen‐fibrinogen or fibrinogen‐collagen or microfibrillar collagen or CoStasis or "GRF Glue" or GR‐Dial or Algosterile or TraumaStat or HemCon or ChitoFlex or Celox or QuikClot or WoundStat or Vitagel or TachSeal or TachoComb or Cryoseal) ) OR AB ( (collagen‐thrombin or thrombin‐collagen or gelatin‐fibrinogen or fibrinogen‐gelatin or gelatin‐thrombin or thrombin‐gelatin or fibrinogen‐thrombin or thrombin‐fibrinogen or collagen‐fibrinogen or fibrinogen‐collagen or microfibrillar collagen or CoStasis or "GRF Glue" or GR‐Dial or Algosterile or TraumaStat or HemCon or ChitoFlex or Celox or QuikClot or WoundStat or Vitagel or TachSeal or TachoComb or Cryoseal) ) S50 S27 OR S28 OR S29 OR S30 OR S31 OR S32 OR S33 OR S34 OR S35 OR S36 OR S37 OR S38 OR S39 OR S40 OR S41 OR S42 OR S43 OR S44 OR S45 OR S46 OR S47 OR S48 OR S49 S51 (MH "Waxes/TU") S52 TI ( (bonewax* or bone wax* or bone putty or hemasorb or ostene) ) OR AB ( (bonewax* or bone wax* or bone putty or hemasorb or ostene) ) S53 S51 OR S52 S54 TI ( (((haemosta* or hemosta* or antihaemorrhag* or antihemorrhag* or anti haemorrhag* or anti‐hemorrhag*) N5 (drug* or agent* or treat* or therap* or dressing*)) or ((coagulat* or clotting) NEXT factor*)) ) OR AB ( (((haemosta* or hemosta* or antihaemorrhag* or antihemorrhag* or anti haemorrhag* or anti‐hemorrhag*) N5 (drug* or agent* or treat* or therap* or dressing*)) or ((coagulat* or clotting) NEXT factor*)) ) S55 S13 OR S17 OR S20 OR S23 OR S26 OR S50 OR S53 OR S54 S56 S9 AND S55 S57 (MH Clinical Trials+) S58 PT Clinical Trial S59 TI ((controlled trial*) or (clinical trial*)) OR AB ((controlled trial*) or (clinical trial*)) S60 TI ((singl* blind*) OR (doubl* blind*) OR (trebl* blind*) OR (tripl* blind*) OR (singl* mask*) OR (doubl* mask*) OR (tripl* mask*)) OR AB ((singl* blind*) OR (doubl* blind*) OR (trebl* blind*) OR (tripl* blind*) OR (singl* mask*) OR (doubl* mask*) OR (tripl* mask*)) S61 TI randomi* OR AB randomi* S62 MH RANDOM ASSIGNMENT S63 TI ((phase three) or (phase III) or (phase three)) or AB ((phase three) or (phase III) or (phase three)) S64 ( TI (random* N2 (assign* or allocat*)) ) OR ( AB (random* N2 (assign* or allocat*)) ) S65 MH PLACEBOS S66 MH META ANALYSIS S67 MH SYSTEMATIC REVIEW S68 TI ("meta analys*" OR metaanalys* OR "systematic review" OR "systematic overview" OR "systematic search*") OR AB ("meta analys*" OR metaanalys* OR "systematic review" OR "systematic overview" OR "systematic search*") S69 TI ("literature review" OR "literature overview" OR "literature search*") OR AB ("literature review" OR "literature overview" OR "literature search*") S70 TI (cochrane OR embase OR cinahl OR cinhal OR lilacs OR BIDS OR science AND citation AND index OR cancerlit) OR AB (cochrane OR embase OR cinahl OR cinhal OR lilacs OR BIDS OR science AND citation AND index OR cancerlit) S71 TI placebo* OR AB placebo* S72 MH QUANTITATIVE STUDIES S73 S57 or S58 or S59 or S60 or S61 or S62 or S63 or S64 or S65 or S66 or S67 or S68 or S69 or S70 or S71 or S72 S74 S56 AND S73

#### Transfusion Evidence Library

Clinical Specialty: Orthopaedic Surgery AND Subject Areas: Alternatives to Blood/Antifibrinolytics OR Alternatives to Blood/Fractionated Blood Products OR Alternatives to Blood/Recombinant Coagulation Factors

#### ClinicalTrials.gov

1. Other Terms: (hip surgery OR knee surgery OR arthroplasty OR hip replacement OR femoral replacement OR knee replacement OR knee injury OR osteoarthritis OR THA OR TKA) AND (randomized or randomised OR randomly OR random) AND Intervention: hemostatic OR antifibrinolytic OR tranexamic OR EACA OR aminocaproic OR aprotinin OR desmopressin OR DDAVP OR factor viia OR novoseven OR aryoseven OR fibrinogen OR haemocomplettan OR Riastap OR Fibryna OR Fibryga OR factor XIII OR Tretten 2. Other Terms: (hip surgery OR knee surgery OR arthroplasty OR hip replacement OR femoral replacement OR knee replacement OR knee injury OR osteoarthritis OR THA OR TKA) AND (randomized or randomised OR randomly OR random) AND Intervention: sealant OR adhesive OR collagen OR cellulose OR gelatin OR glue OR matrix OR sponge OR fleece OR foam OR scaffold OR patch OR sheet OR gelfoam OR chitosan OR hydrogel OR polyethylene glycol OR tachocomb OR BioGlue OR Surgicel OR Veriset 3. Other Terms: (hip surgery OR knee surgery OR arthroplasty OR hip replacement OR femoral replacement OR knee replacement OR knee injury OR osteoarthritis OR THA OR TKA) AND (randomized or randomised OR randomly OR random) AND Intervention: Evithrom OR Floseal OR Tachosil OR Cryoseal OR Hemopatch OR Progel OR Duraseal OR Coseal OR FocalSeal OR Algosterile OR TraumaStat OR HemCon OR ChitoFlex OR Celox OR QuikClot OR WoundStat OR Vitagel OR TachSeal OR bonewax OR hemasorb OR ostene 4. Other Terms: (hip surgery OR knee surgery OR arthroplasty OR hip replacement OR femoral replacement OR knee replacement OR knee injury OR osteoarthritis OR THA OR TKA) AND (randomized or randomised OR randomly OR random) AND Intervention: iniprol or kontrikal OR CloSys OR Glubran OR Gluetiss OR Ifabond OR Indermil OR LiquiBand OR Octafil OR Octanate OR Optivate OR Quixil OR Tisseel OR Tissucol OR TissuGlu OR Thrombi‐Gel OR Vivostat OR Voncento OR Wilate OR Wilnativ OR Wilstart 5. Other Terms: (hip surgery OR knee surgery OR arthroplasty OR hip replacement OR femoral replacement OR knee replacement OR knee injury OR osteoarthritis OR THA OR TKA) AND (randomized or randomised OR randomly OR random) AND Title: hemostasis OR hemostatic OR antifibrinolytic OR factor OR fibrinogen OR thrombin OR collagen OR gelatin OR cellulose 6. Other Terms: (hip surgery OR knee surgery OR arthroplasty OR hip replacement OR femoral replacement OR knee replacement OR knee injury OR osteoarthritis OR THA OR TKA) AND (randomized or randomised OR randomly OR random) AND Condition: bleeding OR hemorrhage OR blood loss OR bloodloss 7. 1 OR 2 OR 3 OR 4 OR 5 OR 6 [N.B. combined and de‐duplicated in EndNote]

#### WHO ICTRP

1. Title OR Condition: hip surgery OR knee surgery OR arthroplasty OR hip replacement OR femoral replacement OR knee replacement OR knee injury OR osteoarthritis OR TKA OR THA Intervention: hemostatic OR antifibrinolytic OR tranexamic OR EACA OR aminocaproic OR aprotinin OR desmopressin OR DDAVP OR factor viia OR novoseven OR aryoseven OR fibrinogen OR haemocomplettan OR Riastap OR Fibryna OR Fibryga OR factor XIII OR Tretten

2. Title OR Condition: hip surgery OR knee surgery OR arthroplasty OR hip replacement OR femoral replacement OR knee replacement OR knee injury OR osteoarthritis OR TKA OR THA Intervention: sealant OR adhesive OR collagen OR cellulose OR gelatin OR glue OR matrix OR sponge OR fleece OR foam OR scaffold OR patch OR sheet OR gelfoam OR chitosan OR hydrogel OR polyethylene glycol OR tachocomb OR BioGlue OR Surgicel OR Veriset

3. Title OR Condition: hip surgery OR knee surgery OR arthroplasty OR hip replacement OR femoral replacement OR knee replacement OR knee injury OR osteoarthritis OR TKA OR THA Intervention: Evithrom OR Floseal OR Tachosil OR Cryoseal OR Hemopatch OR Progel OR Duraseal OR Coseal OR FocalSeal OR Algosterile OR TraumaStat OR HemCon OR ChitoFlex OR Celox OR QuikClot OR WoundStat OR Vitagel OR TachSeal OR bonewax OR hemasorb OR ostene

4. Title OR Condition: hip surgery OR knee surgery OR arthroplasty OR hip replacement OR femoral replacement OR knee replacement OR knee injury OR osteoarthritis OR TKA OR THA Intervention: iniprol or kontrikal OR CloSys OR Glubran OR Gluetiss OR Ifabond OR Indermil OR LiquiBand OR Octafil OR Octanate OR Optivate OR Quixil OR Tisseel OR Tissucol OR TissuGlu OR Thrombi‐Gel OR Vivostat OR Voncento OR Wilate OR Wilnativ OR Wilstart

5. Title OR Condition: hip surgery OR knee surgery OR arthroplasty OR hip replacement OR femoral replacement OR knee replacement OR knee injury OR osteoarthritis OR TKA OR THA Intervention OR Title: hemostasis OR hemostatic OR antifibrinolytic OR factor OR fibrinogen OR thrombin OR collagen OR gelatin OR cellulose

6. 1 OR 2 OR 3 OR 4 OR 5 [N.B. combined and de‐duplicated in EndNote]

### Appendix 3. Results of network meta‐analysis

https://ora.ox.ac.uk/objects/uuid:4d1c666f-22a0-447d-8b8b-c9b30e291d81

### Appendix 4. Interventions of interest by ATC code

**Table:**

**Drug name**	**ATC code**	**Notes**
Epsilon‐aminocaproic acid	B02AA01	Code only available for aminocaproic acid
Tranexamic acid	B02AA02	
Aprotinin	B02AB01	
Fibrinogen	B02BB01	
Fibrin sealants or glue	B02BC	Fibrin sealants providing haemostasis at the site of application
Factor XIII	B02BD07	
Factor VIIa	B02BD08	
Desmopressin	H01BA02	

**Abbreviation** ATC: anatomical therapeutic chemical

## Data and analyses

**Comparison 1:** Results included in the network meta‐analyses

Outcome or subgroup title	No. of studies	No. of participants	Statistical method	Effect size
1.1 Need for allogeneic blood transfusion (only trials included in the NMA)	47		Risk Ratio (M‐H, Random, 95% CI)	Subtotals only
1.1.1 Aprotinin vs placebo	5	731	Risk Ratio (M‐H, Random, 95% CI)	0.66 [0.46, 0.96]
1.1.2 EACA vs placebo	3	168	Risk Ratio (M‐H, Random, 95% CI)	0.59 [0.27, 1.28]
1.1.3 Fibrin topical vs placebo	1	69	Risk Ratio (M‐H, Random, 95% CI)	3.09 [0.34, 28.25]
1.1.4 TXA topical 1 g intraoperatively vs placebo	2	177	Risk Ratio (M‐H, Random, 95% CI)	0.22 [0.08, 0.64]
1.1.5 TXA IV 1 g intraoperatively vs placebo	2	80	Risk Ratio (M‐H, Random, 95% CI)	0.21 [0.08, 0.57]
1.1.6 TXA IV total dose 1 g, intraoperatively and postoperatively vs placebo	1	95	Risk Ratio (M‐H, Random, 95% CI)	0.18 [0.02, 1.42]
1.1.7 TXA IV 1 g pre‐incision vs placebo	7	481	Risk Ratio (M‐H, Random, 95% CI)	0.60 [0.37, 0.98]
1.1.8 TXA IV total dose 1 g, pre‐incision, intraoperatively and postoperatively vs placebo	1	28	Risk Ratio (M‐H, Random, 95% CI)	0.72 [0.49, 1.07]
1.1.9 TXA IV total dose 1 g, pre‐incision and postoperatively vs placebo	1	40	Risk Ratio (M‐H, Random, 95% CI)	0.29 [0.07, 1.21]
1.1.10 TXA IV total dose 2 g, intraoperatively and postoperatively vs placebo	4	260	Risk Ratio (M‐H, Random, 95% CI)	0.37 [0.18, 0.78]
1.1.11 TXA IV 2 g pre‐incision vs placebo	1	42	Risk Ratio (M‐H, Random, 95% CI)	0.15 [0.04, 0.60]
1.1.12 TXA IV total dose 2 g, pre‐incision and postoperatively vs placebo	4	336	Risk Ratio (M‐H, Random, 95% CI)	0.46 [0.26, 0.81]
1.1.13 TXA IV total dose 3 g, intraoperatively and postoperatively vs placebo	2	87	Risk Ratio (M‐H, Random, 95% CI)	0.47 [0.33, 0.68]
1.1.14 TXA IV total dose > 3 g, intraoperatively and postoperatively vs placebo	1	140	Risk Ratio (M‐H, Random, 95% CI)	0.39 [0.16, 0.94]
1.1.15 TXA IV + TXA topical total dose 2 g, intraoperatively vs placebo	1	100	Risk Ratio (M‐H, Random, 95% CI)	0.12 [0.03, 0.48]
1.1.16 TXA IV + TXA topical total dose > 3 g, pre‐incision, intraoperatively and postoperatively vs placebo	1	144	Risk Ratio (M‐H, Random, 95% CI)	0.38 [0.08, 1.89]
1.1.17 TXA oral total dose 2 g, pre‐incision and postoperatively vs placebo	1	80	Risk Ratio (M‐H, Random, 95% CI)	0.13 [0.02, 0.95]
1.1.18 Aprotinin vs EACA	1	30	Risk Ratio (M‐H, Random, 95% CI)	0.50 [0.11, 2.33]
1.1.19 Desmopressin vs TXA IV total dose 1 g, pre‐incision and intraoperatively	1	40	Risk Ratio (M‐H, Random, 95% CI)	3.67 [1.20, 11.19]
1.1.20 EACA vs TXA IV total dose 2 g, intraoperatively and postoperatively	1	67	Risk Ratio (M‐H, Random, 95% CI)	4.38 [0.52, 37.12]
1.1.21 EACA vs TXA oral total dose > 3 g, pre‐incision and postoperatively	1	102	Risk Ratio (M‐H, Random, 95% CI)	3.00 [0.32, 27.89]
1.1.22 Fibrin topical vs TXA IV 1 g intraoperatively	1	100	Risk Ratio (M‐H, Random, 95% CI)	1.40 [0.48, 4.12]
1.1.23 TXA topical 1 g, intraoperatively vs TXA oral + TXA topical total dose > 3 g, pre‐incision, intraoperatively and postoperatively	1	300	Risk Ratio (M‐H, Random, 95% CI)	4.00 [0.58, 27.82]
1.1.24 TXA topical 2 g intraoperatively vs TXA IV 2 g pre‐incision	1	139	Risk Ratio (M‐H, Random, 95% CI)	1.52 [0.66, 3.49]
1.1.25 TXA IV 1 g intraoperatively vs TXA IV 1 g pre‐incision	1	46	Risk Ratio (M‐H, Random, 95% CI)	1.16 [0.81, 1.67]
1.1.26 TXA IV 1 g intraoperatively vs TXA IV total dose 1 g, pre‐incision and intraoperatively	1	49	Risk Ratio (M‐H, Random, 95% CI)	1.49 [0.97, 2.29]
1.1.27 TXA IV 1 g intraoperatively vs TXA IV + TXA topical total dose 2 g, intraoperatively	1	60	Risk Ratio (M‐H, Random, 95% CI)	3.33 [1.02, 10.92]
1.1.28 TXA_IV_1g_intra vs TXA_IV_oral_grt_than_3g_intra_post	1	141	Risk Ratio (M‐H, Random, 95% CI)	0.97 [0.09, 10.42]
1.1.29 TXA_IV_1g_intra_post vs TXA_IV_oral_grt_than_3g_intra_post	1	40	Risk Ratio (M‐H, Random, 95% CI)	1.50 [0.28, 8.04]
1.1.30 TXA_IV_1g_intra_post vs TXA_oral_grt_than_3g_preI_post	1	40	Risk Ratio (M‐H, Random, 95% CI)	0.75 [0.19, 2.93]
1.1.31 TXA_IV_1g_preI vs TXA_IV_1g_preI_intra	1	51	Risk Ratio (M‐H, Random, 95% CI)	1.29 [0.81, 2.04]
1.1.32 TXA_IV_1g_preI vs TXA_IV_2g_preI_intra	1	83	Risk Ratio (M‐H, Random, 95% CI)	0.81 [0.32, 2.04]
1.1.33 TXA_IV_1g_preI vs TXA_IV_2g_preI_post	1	71	Risk Ratio (M‐H, Random, 95% CI)	2.06 [0.68, 6.22]
1.1.34 TXA_IV_1g_preI vs TXA_IV_IA_2g_preI_intra	1	89	Risk Ratio (M‐H, Random, 95% CI)	0.94 [0.37, 2.36]
1.1.35 TXA_IV_1g_preI vs TXA_oral_2g_preI_post	1	89	Risk Ratio (M‐H, Random, 95% CI)	0.94 [0.37, 2.36]
1.1.36 TXA_IV_1g_preI_intra vs TXA_oral_2g_preI	2	154	Risk Ratio (M‐H, Random, 95% CI)	0.48 [0.09, 2.68]
1.1.37 TXA_IV_2g_preI_intra vs TXA_IV_IA_2g_preI_intra	1	86	Risk Ratio (M‐H, Random, 95% CI)	1.15 [0.48, 2.78]
1.1.38 TXA_IV_2g_preI_intra vs TXA_oral_2g_preI_post	1	86	Risk Ratio (M‐H, Random, 95% CI)	1.15 [0.48, 2.78]
1.1.39 TXA_IV_2g_preI_post vs TXA_IV_IA_grt_than_3g_preI_intra_post	1	148	Risk Ratio (M‐H, Random, 95% CI)	2.00 [0.38, 10.59]
1.1.40 TXA_IV_2g_preI_post vs TXA_IV_oral_grt_than_3g_preI_post	1	118	Risk Ratio (M‐H, Random, 95% CI)	1.03 [0.15, 7.10]
1.1.41 TXA_IV_2g_preI_post vs TXA_oral_2g_preI_post	1	80	Risk Ratio (M‐H, Random, 95% CI)	2.00 [0.19, 21.18]
1.1.42 TXA_IV_3g_intra_post vs TXA_oral_3g_preI_post	1	100	Risk Ratio (M‐H, Random, 95% CI)	2.00 [0.19, 21.36]
1.1.43 TXA_IV_IA_2g_preI_intra vs TXA_oral_2g_preI_post	1	92	Risk Ratio (M‐H, Random, 95% CI)	1.00 [0.41, 2.44]
1.1.44 TXA_IV_oral_grt_than_3g_intra_post vs TXA_oral_grt_than_3g_preI_post	1	40	Risk Ratio (M‐H, Random, 95% CI)	0.50 [0.10, 2.43]
1.2 Units of red blood cells transfused (only trials included in the NMA)	16		Mean Difference (IV, Random, 95% CI)	Subtotals only
1.2.1 Aprotinin vs placebo	7	549	Mean Difference (IV, Random, 95% CI)	‐0.96 [‐1.71, ‐0.21]
1.2.2 Desmopressin vs placebo	2	129	Mean Difference (IV, Random, 95% CI)	‐0.15 [‐0.64, 0.33]
1.2.3 EACA vs placebo	1	92	Mean Difference (IV, Random, 95% CI)	‐0.39 [‐0.66, ‐0.12]
1.2.4 TXA_IV_1g_preI vs placebo	2	122	Mean Difference (IV, Random, 95% CI)	0.16 [‐0.97, 1.30]
1.2.5 TXA_IV_1g_preI_intra_post vs placebo	1	28	Mean Difference (IV, Random, 95% CI)	‐1.80 [‐2.98, ‐0.62]
1.2.6 TXA_IV_2g_intra_post vs placebo	3	250	Mean Difference (IV, Random, 95% CI)	‐0.74 [‐1.03, ‐0.46]
1.2.7 TXA_IV_2g_preI_post vs placebo	1	73	Mean Difference (IV, Random, 95% CI)	‐0.60 [‐1.05, ‐0.15]
1.2.8 TXA_IV_3g_intra_post vs placebo	1	77	Mean Difference (IV, Random, 95% CI)	‐2.10 [‐2.73, ‐1.47]
1.2.9 EACA vs TXA_IV_2g_intra_post	1	67	Mean Difference (IV, Random, 95% CI)	0.16 [‐0.03, 0.35]
1.2.10 TXA_IV_1g_preI vs TXA_IV_2g_preI_post	1	71	Mean Difference (IV, Random, 95% CI)	0.20 [‐0.15, 0.55]
1.3 Risk of experiencing DVT (only trials included in the NMA)	19		Risk Ratio (M‐H, Random, 95% CI)	Subtotals only
1.3.1 Aprotinin vs placebo	2	646	Risk Ratio (M‐H, Random, 95% CI)	0.78 [0.36, 1.66]
1.3.2 TXA_IA_2g_intra vs placebo	1	101	Risk Ratio (M‐H, Random, 95% CI)	0.45 [0.15, 1.38]
1.3.3 TXA_IV_1g_intra vs placebo	2	99	Risk Ratio (M‐H, Random, 95% CI)	0.98 [0.54, 1.75]
1.3.4 TXA_IV_1g_post vs placebo	1	103	Risk Ratio (M‐H, Random, 95% CI)	1.06 [0.22, 5.01]
1.3.5 TXA_IV_1g_preI vs placebo	2	122	Risk Ratio (M‐H, Random, 95% CI)	0.96 [0.53, 1.71]
1.3.6 TXA_IV_1g_preI_intra vs placebo	1	53	Risk Ratio (M‐H, Random, 95% CI)	1.04 [0.59, 1.85]
1.3.7 TXA_IV_2g_intra_post vs placebo	3	165	Risk Ratio (M‐H, Random, 95% CI)	1.11 [0.43, 2.91]
1.3.8 TXA_IV_2g_post vs placebo	1	106	Risk Ratio (M‐H, Random, 95% CI)	1.33 [0.31, 5.67]
1.3.9 TXA_IV_2g_preI_post vs placebo	1	73	Risk Ratio (M‐H, Random, 95% CI)	0.51 [0.05, 5.42]
1.3.10 TXA_IV_3g_intra_post vs placebo	1	77	Risk Ratio (M‐H, Random, 95% CI)	0.97 [0.14, 6.57]
1.3.11 TXA_IV_grt_than_3g_intra_post vs placebo	1	140	Risk Ratio (M‐H, Random, 95% CI)	0.97 [0.06, 15.23]
1.3.12 Aprotinin vs TXA_IV_1g_preI_intra	1	24	Risk Ratio (M‐H, Random, 95% CI)	0.50 [0.05, 4.81]
1.3.13 TXA_IA_1g_intra vs TXA_IV_1g_preI	1	93	Risk Ratio (M‐H, Random, 95% CI)	0.68 [0.12, 3.89]
1.3.14 TXA_IA_1g_intra vs TXA_oral_IA_grt_than_3g_preI_intra_post	1	300	Risk Ratio (M‐H, Random, 95% CI)	0.57 [0.07, 4.56]
1.3.15 TXA_IV_1g_intra vs TXA_IV_1g_preI	1	46	Risk Ratio (M‐H, Random, 95% CI)	0.99 [0.53, 1.86]
1.3.16 TXA_IV_1g_intra vs TXA_IV_1g_preI_intra	1	49	Risk Ratio (M‐H, Random, 95% CI)	0.94 [0.52, 1.72]
1.3.17 TXA_IV_1g_intra vs TXA_IV_oral_grt_than_3g_intra_post	1	140	Risk Ratio (M‐H, Random, 95% CI)	2.97 [0.51, 17.16]
1.3.18 TXA_IV_1g_post vs TXA_IV_2g_post	1	103	Risk Ratio (M‐H, Random, 95% CI)	0.80 [0.19, 3.38]
1.3.19 TXA_IV_1g_preI vs TXA_IV_1g_preI_intra	1	51	Risk Ratio (M‐H, Random, 95% CI)	0.95 [0.53, 1.71]
1.3.20 TXA_IV_1g_preI vs TXA_IV_2g_preI_post	1	71	Risk Ratio (M‐H, Random, 95% CI)	1.03 [0.07, 15.81]
1.3.21 TXA_IV_2g_preI_post vs TXA_IV_IA_grt_than_3g_preI_intra_post	1	77	Risk Ratio (M‐H, Random, 95% CI)	1.26 [0.34, 4.69]
1.3.22 TXA_IV_2g_preI_post vs TXA_IV_oral_grt_than_3g_preI_post	1	118	Risk Ratio (M‐H, Random, 95% CI)	0.62 [0.16, 2.48]
1.3.23 TXA_IV_IA_2g_preI_intra vs TXA_IV_IA_grt_than_3g_preI_intra_post	1	100	Risk Ratio (M‐H, Random, 95% CI)	0.85 [0.12, 5.81]
1.4 Length of hospital stay (only trials included in the NMA)	28		Mean Difference (IV, Random, 95% CI)	Subtotals only
1.4.1 Aprotinin vs placebo	1	40	Mean Difference (IV, Random, 95% CI)	0.70 [‐1.71, 3.11]
1.4.2 Desmopressin vs placebo	2	92	Mean Difference (IV, Random, 95% CI)	‐0.50 [‐1.57, 0.57]
1.4.3 Fibrin_top vs placebo	1	69	Mean Difference (IV, Random, 95% CI)	0.07 [‐0.15, 0.29]
1.4.4 TXA_IA_2g_intra vs placebo	1	101	Mean Difference (IV, Random, 95% CI)	‐0.10 [‐0.45, 0.25]
1.4.5 TXA_IA_3g_intra vs placebo	1	69	Mean Difference (IV, Random, 95% CI)	0.07 [‐0.15, 0.29]
1.4.6 TXA_IV_1g_preI vs placebo	2	172	Mean Difference (IV, Random, 95% CI)	‐0.19 [‐1.03, 0.65]
1.4.7 TXA_IV_2g_preI_post vs placebo	3	304	Mean Difference (IV, Random, 95% CI)	‐0.04 [‐0.40, 0.31]
1.4.8 TXA_IV_grt_than_3g_intra_post vs placebo	1	140	Mean Difference (IV, Random, 95% CI)	‐0.80 [‐1.96, 0.36]
1.4.9 TXA_IV_IA_2g_intra vs placebo	1	100	Mean Difference (IV, Random, 95% CI)	‐0.60 [‐1.33, 0.13]
1.4.10 TXA_IV_IA_grt_than_3g_preI_intra_post vs placebo	1	156	Mean Difference (IV, Random, 95% CI)	0.00 [‐0.65, 0.65]
1.4.11 TXA_oral_2g_preI_post vs placebo	1	80	Mean Difference (IV, Random, 95% CI)	‐0.10 [‐0.50, 0.30]
1.4.12 Desmopressin vs TXA_IV_1g_preI_intra	1	40	Mean Difference (IV, Random, 95% CI)	1.00 [‐0.24, 2.24]
1.4.13 EACA vs TXA_IV_2g_preI_intra	1	194	Mean Difference (IV, Random, 95% CI)	0.11 [‐0.17, 0.39]
1.4.14 Fibrin_top vs TXA_IA_3g_intra	1	68	Mean Difference (IV, Random, 95% CI)	0.00 [‐0.24, 0.24]
1.4.15 TXA_IA_3g_intra vs TXA_IV_1g_preI_intra	1	78	Mean Difference (IV, Random, 95% CI)	‐0.40 [‐0.98, 0.18]
1.4.16 TXA_IA_3g_intra vs TXA_IV_2g_intra	1	120	Mean Difference (IV, Random, 95% CI)	0.20 [‐0.82, 1.22]
1.4.17 TXA_IA_3g_intra vs TXA_IV_3g_preI_intra	1	168	Mean Difference (IV, Random, 95% CI)	0.20 [‐0.22, 0.62]
1.4.18 TXA_IV_1g_intra vs TXA_IV_IA_2g_intra	1	60	Mean Difference (IV, Random, 95% CI)	0.03 [‐0.50, 0.56]
1.4.19 TXA_IV_1g_intra_post vs TXA_IV_oral_grt_than_3g_intra_post	1	40	Mean Difference (IV, Random, 95% CI)	0.00 [‐1.24, 1.24]
1.4.20 TXA_IV_1g_intra_post vs TXA_oral_grt_than_3g_preI_post	1	40	Mean Difference (IV, Random, 95% CI)	0.00 [‐1.24, 1.24]
1.4.21 TXA_IV_1g_preI vs TXA_IV_2g_preI_intra	1	83	Mean Difference (IV, Random, 95% CI)	‐0.80 [‐1.76, 0.16]
1.4.22 TXA_IV_1g_preI vs TXA_IV_2g_preI_post	1	71	Mean Difference (IV, Random, 95% CI)	0.00 [‐1.14, 1.14]
1.4.23 TXA_IV_1g_preI vs TXA_IV_IA_2g_preI_intra	1	89	Mean Difference (IV, Random, 95% CI)	‐0.60 [‐1.69, 0.49]
1.4.24 TXA_IV_1g_preI vs TXA_oral_2g_preI_post	1	89	Mean Difference (IV, Random, 95% CI)	‐0.20 [‐1.02, 0.62]
1.4.25 TXA_IV_1g_preI_intra vs TXA_oral_2g_preI	2	154	Mean Difference (IV, Random, 95% CI)	0.00 [‐0.32, 0.32]
1.4.26 TXA_IV_2g_preI_intra vs TXA_IV_IA_2g_preI_intra	1	86	Mean Difference (IV, Random, 95% CI)	0.20 [‐1.09, 1.49]
1.4.27 TXA_IV_2g_preI_intra vs TXA_oral_2g_preI_post	1	86	Mean Difference (IV, Random, 95% CI)	0.60 [‐0.47, 1.67]
1.4.28 TXA_IV_2g_preI_post vs TXA_IV_3g_preI_intra	1	110	Mean Difference (IV, Random, 95% CI)	1.00 [0.57, 1.43]
1.4.29 TXA_IV_2g_preI_post vs TXA_IV_grt_than_3g_preI_post	1	102	Mean Difference (IV, Random, 95% CI)	0.50 [0.05, 0.95]
1.4.30 TXA_IV_2g_preI_post vs TXA_IV_IA_grt_than_3g_preI_intra_post	1	154	Mean Difference (IV, Random, 95% CI)	0.40 [‐0.39, 1.19]
1.4.31 TXA_IV_2g_preI_post vs TXA_IV_oral_grt_than_3g_preI_post	1	118	Mean Difference (IV, Random, 95% CI)	0.30 [‐0.08, 0.68]
1.4.32 TXA_IV_2g_preI_post vs TXA_oral_2g_preI_post	1	80	Mean Difference (IV, Random, 95% CI)	0.00 [‐0.20, 0.20]
1.4.33 TXA_IV_3g_intra_post vs TXA_oral_3g_preI_post	1	100	Mean Difference (IV, Random, 95% CI)	‐0.20 [‐0.57, 0.17]
1.4.34 TXA_IV_3g_preI_intra vs TXA_IV_grt_than_3g_preI_post	1	106	Mean Difference (IV, Random, 95% CI)	‐0.50 [‐0.83, ‐0.17]
1.4.35 TXA_IV_grt_than_3g_preI_post vs TXA_IV_IA_3g_preI_intra_post	1	101	Mean Difference (IV, Random, 95% CI)	‐1.30 [‐1.89, ‐0.71]
1.4.36 TXA_IV_grt_than_3g_preI_post vs TXA_IV_oral_grt_than_3g_preI_post	1	101	Mean Difference (IV, Random, 95% CI)	‐0.90 [‐1.41, ‐0.39]
1.4.37 TXA_IV_IA_2g_preI_intra vs TXA_oral_2g_preI_post	1	92	Mean Difference (IV, Random, 95% CI)	0.40 [‐0.79, 1.59]
1.4.38 TXA_IV_IA_3g_preI_intra vs TXA_IV_IA_grt_than_3g_preI_intra_post	1	150	Mean Difference (IV, Random, 95% CI)	0.72 [0.12, 1.33]
1.4.39 TXA_IV_IA_3g_preI_intra_post vs TXA_IV_oral_grt_than_3g_preI_post	1	100	Mean Difference (IV, Random, 95% CI)	0.40 [‐0.15, 0.95]
1.4.40 TXA_IV_IA_oral_grt_than_3g_intra_post vs TXA_oral_3g_preI_post	1	53	Mean Difference (IV, Random, 95% CI)	0.50 [‐0.20, 1.20]
1.4.41 TXA_IV_oral_grt_than_3g_intra_post vs TXA_oral_grt_than_3g_preI_post	1	40	Mean Difference (IV, Random, 95% CI)	0.00 [‐1.24, 1.24]
1.4.42 TXA_oral_2g_preI vs TXA_oral_3g_preI_post	1	100	Mean Difference (IV, Random, 95% CI)	‐0.12 [‐0.44, 0.20]
1.4.43 TXA_oral_2g_preI vs TXA_oral_grt_than_3g_preI_post	1	150	Mean Difference (IV, Random, 95% CI)	‐0.32 [‐0.60, ‐0.04]
1.4.44 TXA_oral_3g_preI_post vs TXA_oral_grt_than_3g_preI_post	1	150	Mean Difference (IV, Random, 95% CI)	‐0.20 [‐0.49, 0.09]

**Comparison 2:** TXA IV vs placebo

Outcome or subgroup title	No. of studies	No. of participants	Statistical method	Effect size
2.1 Need for allogeneic blood transfusion	33		Risk Ratio (M‐H, Random, 95% CI)	Subtotals only
2.1.1 TXA, IV 1 g	20	1133	Risk Ratio (M‐H, Random, 95% CI)	0.46 [0.33, 0.64]
2.1.2 TXA, IV 2 g	10	707	Risk Ratio (M‐H, Random, 95% CI)	0.46 [0.22, 0.94]
2.1.3 TXA, IV 3 g	1	140	Risk Ratio (M‐H, Random, 95% CI)	0.39 [0.16, 0.94]
2.1.4 TXA, IV more than 3 g	4	296	Risk Ratio (M‐H, Random, 95% CI)	0.23 [0.09, 0.57]
2.2 All‐cause mortality	5		Peto Odds Ratio (Peto, Fixed, 95% CI)	Subtotals only
2.2.1 TXA, IV 1 g	3	105	Peto Odds Ratio (Peto, Fixed, 95% CI)	Not estimable
2.2.2 TXA, IV 2 g	1	77	Peto Odds Ratio (Peto, Fixed, 95% CI)	0.13 [0.00, 6.65]
2.2.3 TXA, IV 3 g	1	140	Peto Odds Ratio (Peto, Fixed, 95% CI)	0.13 [0.00, 6.63]
2.3 Units of red blood cells transfused	7		Mean Difference (IV, Random, 95% CI)	Subtotals only
2.3.1 TXA, IV 1 g	5	326	Mean Difference (IV, Random, 95% CI)	‐0.31 [‐0.83, 0.21]
2.3.2 TXA, IV 2 g	3	252	Mean Difference (IV, Random, 95% CI)	‐1.14 [‐1.78, ‐0.50]
2.4 Reoperation	3		Peto Odds Ratio (Peto, Fixed, 95% CI)	Subtotals only
2.4.1 TXA, IV 2 g	1	151	Peto Odds Ratio (Peto, Fixed, 95% CI)	Not estimable
2.4.2 TXA, IV 3 g	1	140	Peto Odds Ratio (Peto, Fixed, 95% CI)	Not estimable
2.4.3 TXA, IV more than 3 g	2	253	Peto Odds Ratio (Peto, Fixed, 95% CI)	7.20 [0.45, 116.28]
2.5 Length of hospital stay	6		Mean Difference (IV, Random, 95% CI)	Subtotals only
2.5.1 TXA, IV 1 g	3	253	Mean Difference (IV, Random, 95% CI)	‐0.34 [‐0.82, 0.15]
2.5.2 TXA, IV 2 g	3	304	Mean Difference (IV, Random, 95% CI)	‐0.04 [‐0.40, 0.31]
2.5.3 TXA, IV 3 g	1	140	Mean Difference (IV, Random, 95% CI)	‐0.80 [‐1.96, 0.36]
2.5.4 TXA, IV more than 3 g	1	153	Mean Difference (IV, Random, 95% CI)	‐0.50 [‐1.10, 0.10]
2.6 Risk of experiencing DVT	35		Peto Odds Ratio (Peto, Fixed, 95% CI)	Subtotals only
2.6.1 TXA, IV 1 g	21	1097	Peto Odds Ratio (Peto, Fixed, 95% CI)	1.22 [0.62, 2.40]
2.6.2 TXA, IV 2 g	10	714	Peto Odds Ratio (Peto, Fixed, 95% CI)	0.85 [0.30, 2.42]
2.6.3 TXA, IV 3 g	2	200	Peto Odds Ratio (Peto, Fixed, 95% CI)	0.97 [0.06, 15.69]
2.6.4 TXA, IV more than 3 g	4	305	Peto Odds Ratio (Peto, Fixed, 95% CI)	0.56 [0.11, 2.85]
2.7 Risk of experiencing PE	27		Peto Odds Ratio (Peto, Fixed, 95% CI)	Subtotals only
2.7.1 TXA, IV 1 g	16	942	Peto Odds Ratio (Peto, Fixed, 95% CI)	1.26 [0.33, 4.86]
2.7.2 TXA, IV 2 g	7	499	Peto Odds Ratio (Peto, Fixed, 95% CI)	0.66 [0.04, 11.65]
2.7.3 TXA, IV 3 g	2	200	Peto Odds Ratio (Peto, Fixed, 95% CI)	0.13 [0.00, 6.63]
2.7.4 TXA, IV more than 3 g	3	263	Peto Odds Ratio (Peto, Fixed, 95% CI)	Not estimable
2.8 Risk of experiencing MI	12		Peto Odds Ratio (Peto, Fixed, 95% CI)	Subtotals only
2.8.1 TXA, IV 1 g	7	386	Peto Odds Ratio (Peto, Fixed, 95% CI)	0.90 [0.06, 14.40]
2.8.2 TXA, IV 2 g	3	268	Peto Odds Ratio (Peto, Fixed, 95% CI)	0.13 [0.00, 6.65]
2.8.3 TXA, IV 3 g	1	140	Peto Odds Ratio (Peto, Fixed, 95% CI)	0.97 [0.06, 15.69]
2.8.4 More than 3 g	2	253	Peto Odds Ratio (Peto, Fixed, 95% CI)	7.11 [0.14, 358.60]
2.9 Risk of experiencing CVA	5		Peto Odds Ratio (Peto, Fixed, 95% CI)	Subtotals only
2.9.1 TXA, IV 1 g	3	233	Peto Odds Ratio (Peto, Fixed, 95% CI)	Not estimable
2.9.2 TXA, IV 3 g	1	140	Peto Odds Ratio (Peto, Fixed, 95% CI)	0.13 [0.00, 6.63]
2.9.3 TXA, IV more than 3 g	1	100	Peto Odds Ratio (Peto, Fixed, 95% CI)	Not estimable
2.10 Risk of having suspected serious drug reactions	4		Peto Odds Ratio (Peto, Fixed, 95% CI)	Subtotals only
2.10.1 TXA, IV 1 g	2	133	Peto Odds Ratio (Peto, Fixed, 95% CI)	Not estimable
2.10.2 TXA, IV 2 g	1	39	Peto Odds Ratio (Peto, Fixed, 95% CI)	Not estimable
2.10.3 TXA, IV more than 3 g	1	10	Peto Odds Ratio (Peto, Fixed, 95% CI)	Not estimable

**Comparison 3:** TXA oral vs placebo

Outcome or subgroup title	No. of studies	No. of participants	Statistical method	Effect size
3.1 Need for allogeneic blood transfusion	2		Peto Odds Ratio (Peto, Fixed, 95% CI)	Subtotals only
3.1.1 TXA, oral 1 g	1	100	Peto Odds Ratio (Peto, Fixed, 95% CI)	0.14 [0.00, 6.82]
3.1.2 TXA, oral 2 g	2	180	Peto Odds Ratio (Peto, Fixed, 95% CI)	0.17 [0.05, 0.63]
3.1.3 TXA, oral 3 g	1	100	Peto Odds Ratio (Peto, Fixed, 95% CI)	0.14 [0.00, 6.82]
3.2 All‐cause mortality	1		Peto Odds Ratio (Peto, Fixed, 95% CI)	Subtotals only
3.2.1 TXA, oral 1 g	1	100	Peto Odds Ratio (Peto, Fixed, 95% CI)	Not estimable
3.2.2 TXA, oral 2 g	1	100	Peto Odds Ratio (Peto, Fixed, 95% CI)	Not estimable
3.2.3 TXA, oral 3 g	1	100	Peto Odds Ratio (Peto, Fixed, 95% CI)	Not estimable
3.3 Length of hospital stay	1		Mean Difference (IV, Random, 95% CI)	Subtotals only
3.3.1 TXA, oral 2 g	1	80	Mean Difference (IV, Random, 95% CI)	‐0.10 [‐0.50, 0.30]
3.4 Risk of experiencing DVT	3		Peto Odds Ratio (Peto, Fixed, 95% CI)	Subtotals only
3.4.1 TXA, oral 1 g	1	100	Peto Odds Ratio (Peto, Fixed, 95% CI)	Not estimable
3.4.2 TXA, oral 2 g	2	180	Peto Odds Ratio (Peto, Fixed, 95% CI)	Not estimable
3.4.3 TXA, oral 3 g	1	100	Peto Odds Ratio (Peto, Fixed, 95% CI)	Not estimable
3.4.4 TXA, oral more than 3 g	1	102	Peto Odds Ratio (Peto, Fixed, 95% CI)	0.14 [0.00, 6.82]
3.5 Risk of experiencing PE	3		Peto Odds Ratio (Peto, Fixed, 95% CI)	Subtotals only
3.5.1 TXA, oral 1 g	1	100	Peto Odds Ratio (Peto, Fixed, 95% CI)	Not estimable
3.5.2 TXA, oral 2 g	2	180	Peto Odds Ratio (Peto, Fixed, 95% CI)	Not estimable
3.5.3 TXA, oral 3 g	1	100	Peto Odds Ratio (Peto, Fixed, 95% CI)	Not estimable
3.5.4 TXA, oral more than 3 g	1	102	Peto Odds Ratio (Peto, Fixed, 95% CI)	Not estimable
3.6 Risk of experiencing MI	1		Peto Odds Ratio (Peto, Fixed, 95% CI)	Subtotals only
3.6.1 TXA, oral more than 3 g	1	102	Peto Odds Ratio (Peto, Fixed, 95% CI)	Not estimable
3.7 Risk of experiencing CVA	2		Peto Odds Ratio (Peto, Fixed, 95% CI)	Subtotals only
3.7.1 TXA, oral 1 g	1	100	Peto Odds Ratio (Peto, Fixed, 95% CI)	Not estimable
3.7.2 TXA, oral 2 g	1	100	Peto Odds Ratio (Peto, Fixed, 95% CI)	Not estimable
3.7.3 TXA, oral 3 g	1	100	Peto Odds Ratio (Peto, Fixed, 95% CI)	Not estimable
3.7.4 TXA, oral more than 3 g	1	102	Peto Odds Ratio (Peto, Fixed, 95% CI)	Not estimable

**Comparison 4:** TXA topical vs placebo

Outcome or subgroup title	No. of studies	No. of participants	Statistical method	Effect size
4.1 Risk of allogeneic blood transfusion	6		Risk Ratio (M‐H, Random, 95% CI)	Subtotals only
4.1.1 TXA, topical 1 g	1	94	Risk Ratio (M‐H, Random, 95% CI)	0.23 [0.07, 0.76]
4.1.2 TXA, topical 2 g	2	184	Risk Ratio (M‐H, Random, 95% CI)	0.16 [0.03, 0.97]
4.1.3 TXA, topical 3 g	2	131	Risk Ratio (M‐H, Random, 95% CI)	0.25 [0.03, 2.20]
4.1.4 TXA, topical more than 3 g	1	77	Risk Ratio (M‐H, Random, 95% CI)	Not estimable
4.2 All‐cause mortality	2		Risk Ratio (M‐H, Random, 95% CI)	Totals not selected
4.2.1 TXA, topical 3 g	2		Risk Ratio (M‐H, Random, 95% CI)	Totals not selected
4.3 Reoperation	1		Risk Ratio (M‐H, Random, 95% CI)	Subtotals only
4.3.1 TXA, topical 3 g	1	69	Risk Ratio (M‐H, Random, 95% CI)	Not estimable
4.4 Length of hospital stay	1		Mean Difference (IV, Random, 95% CI)	Subtotals only
4.4.1 TXA, topical 2 g	1	101	Mean Difference (IV, Random, 95% CI)	‐0.10 [‐0.45, 0.25]
4.5 Risk of experiencing DVT	6		Risk Ratio (M‐H, Random, 95% CI)	Subtotals only
4.5.1 TXA, topical 1 g	1	94	Risk Ratio (M‐H, Random, 95% CI)	Not estimable
4.5.2 TXA, Topical 2g	2	184	Risk Ratio (M‐H, Random, 95% CI)	0.45 [0.15, 1.38]
4.5.3 TXA, Topical 3g	2	130	Risk Ratio (M‐H, Random, 95% CI)	Not estimable
4.5.4 TXA, Topical More than 3g	1	77	Risk Ratio (M‐H, Random, 95% CI)	0.79 [0.21, 2.93]
4.6 Risk of experiencing PE	6		Peto Odds Ratio (Peto, Fixed, 95% CI)	Subtotals only
4.6.1 TXA, topical 1 g	1	94	Peto Odds Ratio (Peto, Fixed, 95% CI)	Not estimable
4.6.2 TXA, topical 2 g	2	184	Peto Odds Ratio (Peto, Fixed, 95% CI)	1.06 [0.17, 6.71]
4.6.3 TXA, topical 3 g	2	130	Peto Odds Ratio (Peto, Fixed, 95% CI)	Not estimable
4.6.4 TXA, topical more than 3 g	1	77	Peto Odds Ratio (Peto, Fixed, 95% CI)	Not estimable
4.7 Risk of experiencing CVA	1		Risk Ratio (M‐H, Random, 95% CI)	Subtotals only
4.7.1 TXA, topical 3 g	1	69	Risk Ratio (M‐H, Random, 95% CI)	Not estimable
4.8 Risk of transfusion reactions	1		Peto Odds Ratio (Peto, Fixed, 95% CI)	Totals not selected
4.8.1 TXA, topical 1 g	1		Peto Odds Ratio (Peto, Fixed, 95% CI)	Totals not selected

**Comparison 5:** TXA IV + TXA topical vs placebo

Outcome or subgroup title	No. of studies	No. of participants	Statistical method	Effect size
5.1 Risk of allogeneic blood transfusion	1		Peto Odds Ratio (Peto, Fixed, 95% CI)	Totals not selected
5.1.1 TXA, IV + TXA, topical 2 g	1		Peto Odds Ratio (Peto, Fixed, 95% CI)	Totals not selected
5.2 Length of hospital stay	1		Mean Difference (IV, Random, 95% CI)	Totals not selected
5.3 Risk of experiencing DVT	1		Peto Odds Ratio (Peto, Fixed, 95% CI)	Totals not selected
5.3.1 TXA, IV + TXA, topical 2 g	1		Peto Odds Ratio (Peto, Fixed, 95% CI)	Totals not selected
5.4 Risk of experiencing PE	1		Risk Ratio (M‐H, Random, 95% CI)	Subtotals only
5.4.1 TXA, IV + TXA, topical 2 g	1	100	Risk Ratio (M‐H, Random, 95% CI)	Not estimable

**Comparison 6:** TXA IV lower dose vs TXA IV higher dose

Outcome or subgroup title	No. of studies	No. of participants	Statistical method	Effect size
6.1 Risk of allogeneic blood transfusion	13		Risk Ratio (M‐H, Random, 95% CI)	Subtotals only
6.1.1 TXA, IV 1 g vs TXA, IV 2 g	5	297	Risk Ratio (M‐H, Random, 95% CI)	1.12 [0.53, 2.36]
6.1.2 TXA, IV 1g vs TXA, IV 3g	1	97	Risk Ratio (M‐H, Random, 95% CI)	Not estimable
6.1.3 TXA, IV 2g vs TXA, IV More than 3g	3	359	Risk Ratio (M‐H, Random, 95% CI)	2.00 [0.38, 10.59]
6.1.4 TXA, IV 2g vs TXA, IV 3g	2	202	Risk Ratio (M‐H, Random, 95% CI)	Not estimable
6.1.5 TXA, IV 3g vs TXA, IV More than 3g	2	207	Risk Ratio (M‐H, Random, 95% CI)	Not estimable
6.1.6 TXA, IV 3g vs TXA, IV 4g	1	100	Risk Ratio (M‐H, Random, 95% CI)	Not estimable
6.1.7 TXA, IV 3g vs TXA, IV 5g	1	100	Risk Ratio (M‐H, Random, 95% CI)	Not estimable
6.1.8 TXA, IV 4g vs TXA, IV 5g	2	200	Risk Ratio (M‐H, Random, 95% CI)	Not estimable
6.1.9 TXA, IV 5g vs TXA, IV More than 5g	2	262	Risk Ratio (M‐H, Random, 95% CI)	Not estimable
6.1.10 TXA, IV 4g vs TXA, IV More than 5g	1	100	Risk Ratio (M‐H, Random, 95% CI)	Not estimable
6.1.11 TXA, IV 3.5g vs TXA, IV 5.5g	1	200	Risk Ratio (M‐H, Random, 95% CI)	Not estimable
6.1.12 TXA, IV 5.5g vs TXA, IV 6.5g	1	200	Risk Ratio (M‐H, Random, 95% CI)	Not estimable
6.1.13 TXA, IV 3.5g vs TXA, IV 6.5g	1	200	Risk Ratio (M‐H, Random, 95% CI)	Not estimable
6.2 All‐cause mortality	2		Risk Ratio (M‐H, Random, 95% CI)	Subtotals only
6.2.1 TXA, IV 1 g vs TXA, IV 2 g	1	40	Risk Ratio (M‐H, Random, 95% CI)	Not estimable
6.2.2 TXA, IV 5g vs TXA, IV more than 5 g	1	162	Risk Ratio (M‐H, Random, 95% CI)	Not estimable
6.3 Units of red blood cells transfused	2		Mean Difference (IV, Random, 95% CI)	Subtotals only
6.3.1 TXA, IV 1 g vs TXA, IV 2 g	1	71	Mean Difference (IV, Random, 95% CI)	0.20 [‐0.15, 0.55]
6.3.2 TXA, IV 1 g vs TXA, IV 3 g	1	40	Mean Difference (IV, Random, 95% CI)	‐0.05 [‐0.36, 0.26]
6.4 Reoperation	3		Risk Ratio (M‐H, Random, 95% CI)	Totals not selected
6.4.1 TXA, IV 1 g vs TXA, IV 3 g	1		Risk Ratio (M‐H, Random, 95% CI)	Totals not selected
6.4.2 TXA, IV 2 g vs TXA, IV more than 3 g	1		Risk Ratio (M‐H, Random, 95% CI)	Totals not selected
6.4.3 TXA, IV 5 g vs TXA, IV more than 5 g	1		Risk Ratio (M‐H, Random, 95% CI)	Totals not selected
6.5 Length of hospital stay	9		Mean Difference (IV, Random, 95% CI)	Subtotals only
6.5.1 TXA, IV 1 g vs TXA, IV 2 g	2	154	Mean Difference (IV, Random, 95% CI)	‐0.46 [‐1.24, 0.31]
6.5.2 TXA, IV 1 g vs TXA, IV 3 g	1	40	Mean Difference (IV, Random, 95% CI)	0.00 [‐1.24, 1.24]
6.5.3 TXA, IV 2 g vs TXA, IV more than 3 g	3	365	Mean Difference (IV, Random, 95% CI)	0.97 [0.54, 1.39]
6.5.4 TXA, IV 2 g vs TXA, IV 3 g	2	202	Mean Difference (IV, Random, 95% CI)	0.46 [0.11, 0.81]
6.5.5 TXA, IV 3 g vs TXA, IV more than 3 g	2	207	Mean Difference (IV, Random, 95% CI)	0.65 [0.27, 1.03]
6.5.6 TXA, IV 3 g vs TXA, IV 4 g	1	100	Mean Difference (IV, Random, 95% CI)	‐0.10 [‐0.69, 0.49]
6.5.7 TXA, IV 3 g vs TXA, IV 5 g	1	100	Mean Difference (IV, Random, 95% CI)	0.30 [‐0.20, 0.80]
6.5.8 TXA, IV 4 g vs TXA, IV 5 g	2	200	Mean Difference (IV, Random, 95% CI)	0.18 [‐0.08, 0.44]
6.5.9 TXA, IV 5 g vs TXA, IV more than 5 g	2	262	Mean Difference (IV, Random, 95% CI)	0.03 [‐0.13, 0.20]
6.5.10 TXA, IV 4 g vs TXA, IV more than 5 g	1	100	Mean Difference (IV, Random, 95% CI)	0.30 [0.02, 0.58]
6.6 Risk of experiencing DVT	14		Peto Odds Ratio (Peto, Fixed, 95% CI)	Subtotals only
6.6.1 TXA, IV 1 g vs TXA, IV 2 g	5	297	Peto Odds Ratio (Peto, Fixed, 95% CI)	0.52 [0.05, 5.05]
6.6.2 TXA, IV 1 g vs TXA, IV 3 g	2	137	Peto Odds Ratio (Peto, Fixed, 95% CI)	Not estimable
6.6.3 TXA, IV 2 g vs TXA, IV more than 3 g	3	365	Peto Odds Ratio (Peto, Fixed, 95% CI)	Not estimable
6.6.4 TXA, IV 2 g vs TXA, IV 3 g	2	202	Peto Odds Ratio (Peto, Fixed, 95% CI)	Not estimable
6.6.5 TXA, IV 3 g vs TXA, IV more than 3 g	2	207	Peto Odds Ratio (Peto, Fixed, 95% CI)	Not estimable
6.6.6 TXA, IV 3 g vs TXA, IV 4 g	1	100	Peto Odds Ratio (Peto, Fixed, 95% CI)	Not estimable
6.6.7 TXA, IV 3 g vs TXA, IV 5 g	1	100	Peto Odds Ratio (Peto, Fixed, 95% CI)	Not estimable
6.6.8 TXA, IV 4 g vs TXA, IV 5 g	2	200	Peto Odds Ratio (Peto, Fixed, 95% CI)	Not estimable
6.6.9 TXA, IV 5 g vs TXA, IV more than 5 g	2	262	Peto Odds Ratio (Peto, Fixed, 95% CI)	0.98 [0.06, 15.73]
6.6.10 TXA, IV 4 g vs TXA, IV more than 5 g	1	100	Peto Odds Ratio (Peto, Fixed, 95% CI)	Not estimable
6.6.11 TXA, IV 3.5 g vs TXA, IV 5.5 g	1	200	Peto Odds Ratio (Peto, Fixed, 95% CI)	Not estimable
6.6.12 TXA, IV 5.5 g vs TXA, IV 6.5 g	1	200	Peto Odds Ratio (Peto, Fixed, 95% CI)	Not estimable
6.6.13 TXA, IV 3.5 g vs TXA, IV 6.5 g	1	200	Peto Odds Ratio (Peto, Fixed, 95% CI)	Not estimable
6.7 Risk of experiencing PE	13		Peto Odds Ratio (Peto, Fixed, 95% CI)	Subtotals only
6.7.1 TXA, IV 1 g vs TXA, IV 2 g	4	226	Peto Odds Ratio (Peto, Fixed, 95% CI)	7.39 [0.15, 372.38]
6.7.2 TXA, IV 1 g vs TXA, IV 3 g	2	137	Peto Odds Ratio (Peto, Fixed, 95% CI)	Not estimable
6.7.3 TXA, IV 2 g vs TXA, IV more than 3 g	3	365	Peto Odds Ratio (Peto, Fixed, 95% CI)	Not estimable
6.7.4 TXA, IV 2 g vs TXA, IV 3 g	2	202	Peto Odds Ratio (Peto, Fixed, 95% CI)	Not estimable
6.7.5 TXA, IV 3 g vs TXA, IV more than 3 g	2	207	Peto Odds Ratio (Peto, Fixed, 95% CI)	Not estimable
6.7.6 TXA, IV 3 g vs TXA, IV 4 g	1	100	Peto Odds Ratio (Peto, Fixed, 95% CI)	Not estimable
6.7.7 TXA, IV 3 g vs TXA, IV 5 g	1	100	Peto Odds Ratio (Peto, Fixed, 95% CI)	Not estimable
6.7.8 TXA, IV 4 g vs TXA, IV 5 g	2	200	Peto Odds Ratio (Peto, Fixed, 95% CI)	Not estimable
6.7.9 TXA, IV 5 g vs TXA, IV more than 5 g	2	262	Peto Odds Ratio (Peto, Fixed, 95% CI)	Not estimable
6.7.10 TXA, IV 4 g vs TXA, IV more than 5 g	1	100	Peto Odds Ratio (Peto, Fixed, 95% CI)	Not estimable
6.7.11 TXA, IV 3.5 g vs TXA, IV 5.5 g	1	200	Peto Odds Ratio (Peto, Fixed, 95% CI)	Not estimable
6.7.12 TXA, IV 3.5 g vs TXA, IV 6.5 g	1	200	Peto Odds Ratio (Peto, Fixed, 95% CI)	Not estimable
6.7.13 TXA, IV 5.5 g vs TXA, IV 6.5 g	1	200	Peto Odds Ratio (Peto, Fixed, 95% CI)	Not estimable
6.8 Risk of experiencing MI	7		Peto Odds Ratio (Peto, Fixed, 95% CI)	Subtotals only
6.8.1 TXA, IV 1 g vs TXA, IV 3 g	1	97	Peto Odds Ratio (Peto, Fixed, 95% CI)	Not estimable
6.8.2 TXA, IV 2 g vs TXA, IV more than 3 g	3	365	Peto Odds Ratio (Peto, Fixed, 95% CI)	0.14 [0.00, 7.00]
6.8.3 TXA, IV 2 g vs TXA, IV 3 g	2	202	Peto Odds Ratio (Peto, Fixed, 95% CI)	Not estimable
6.8.4 TXA, IV 3 g vs TXA, IV more than 3 g	2	207	Peto Odds Ratio (Peto, Fixed, 95% CI)	Not estimable
6.8.5 TXA, IV 3 g vs TXA, IV 4 g	1	100	Peto Odds Ratio (Peto, Fixed, 95% CI)	Not estimable
6.8.6 TXA, IV 3 g vs TXA, IV 5 g	1	100	Peto Odds Ratio (Peto, Fixed, 95% CI)	Not estimable
6.8.7 TXA, IV 4 g vs TXA, IV 5 g	2	200	Peto Odds Ratio (Peto, Fixed, 95% CI)	Not estimable
6.8.8 TXA, IV 5 g vs TXA, IV more than 5 g	2	262	Peto Odds Ratio (Peto, Fixed, 95% CI)	Not estimable
6.8.9 TXA, IV 4 g vs TXA, IV more than 5 g	1	100	Peto Odds Ratio (Peto, Fixed, 95% CI)	Not estimable
6.9 Risk of experiencing CVA	6		Peto Odds Ratio (Peto, Fixed, 95% CI)	Subtotals only
6.9.1 TXA, IV 1 g vs TXA, IV 2 g	1	83	Peto Odds Ratio (Peto, Fixed, 95% CI)	Not estimable
6.9.2 TXA, IV 2 g vs TXA, IV 3 g	2	202	Peto Odds Ratio (Peto, Fixed, 95% CI)	Not estimable
6.9.3 TXA, IV 2 g vs TXA, IV more than 3 g	2	211	Peto Odds Ratio (Peto, Fixed, 95% CI)	Not estimable
6.9.4 TXA, IV 3 g vs TXA, IV more than 3 g	2	207	Peto Odds Ratio (Peto, Fixed, 95% CI)	Not estimable
6.9.5 TXA, IV 3 g vs TXA, IV 4 g	1	100	Peto Odds Ratio (Peto, Fixed, 95% CI)	Not estimable
6.9.6 TXA, IV 3 g vs TXA, IV 5 g	1	100	Peto Odds Ratio (Peto, Fixed, 95% CI)	Not estimable
6.9.7 TXA, IV 4 g vs TXA, IV 5 g	2	200	Peto Odds Ratio (Peto, Fixed, 95% CI)	Not estimable
6.9.8 TXA, IV 5 g vs TXA, IV more than 5 g	2	262	Peto Odds Ratio (Peto, Fixed, 95% CI)	Not estimable
6.9.9 TXA, IV 4 g vs TXA, IV more than 5 g	1	100	Peto Odds Ratio (Peto, Fixed, 95% CI)	Not estimable
6.10 Risk of suspected serious drug reactions	1		Risk Ratio (M‐H, Random, 95% CI)	Subtotals only
6.10.1 TXA, IV 1 g vs TXA, IV 2 g	1	40	Risk Ratio (M‐H, Random, 95% CI)	Not estimable

**Comparison 7:** TXA IV vs TXA oral

Outcome or subgroup title	No. of studies	No. of participants	Statistical method	Effect size
7.1 Risk of allogeneic blood transfusion	6		Risk Ratio (M‐H, Random, 95% CI)	Subtotals only
7.1.1 TXA, IV 1g	4	283	Risk Ratio (M‐H, Random, 95% CI)	0.79 [0.39, 1.59]
7.1.2 TXA, IV 2 g	2	166	Risk Ratio (M‐H, Random, 95% CI)	1.23 [0.54, 2.82]
7.1.3 TXA, IV 3 g	1	100	Risk Ratio (M‐H, Random, 95% CI)	2.00 [0.19, 21.36]
7.2 Units of red blood cells transfused	2		Mean Difference (IV, Random, 95% CI)	Subtotals only
7.2.1 TXA, IV 1 g	2	123	Mean Difference (IV, Random, 95% CI)	‐0.06 [‐0.14, 0.03]
7.3 Length of hospital stay	6		Mean Difference (IV, Random, 95% CI)	Subtotals only
7.3.1 TXA, IV 1 g	4	283	Mean Difference (IV, Random, 95% CI)	‐0.02 [‐0.31, 0.26]
7.3.2 TXA, IV 2 g	2	166	Mean Difference (IV, Random, 95% CI)	0.06 [‐0.29, 0.42]
7.3.3 TXA, IV 3 g	1	100	Mean Difference (IV, Random, 95% CI)	‐0.20 [‐0.57, 0.17]
7.4 Risk of experiencing DVT	6		Peto Odds Ratio (Peto, Fixed, 95% CI)	Subtotals only
7.4.1 TXA, IV 1 g	4	283	Peto Odds Ratio (Peto, Fixed, 95% CI)	0.14 [0.00, 7.30]
7.4.2 TXA, IV 2 g	2	166	Peto Odds Ratio (Peto, Fixed, 95% CI)	0.15 [0.00, 7.85]
7.4.3 TXA, IV 3 g	1	100	Peto Odds Ratio (Peto, Fixed, 95% CI)	Not estimable
7.5 Risk of experiencing PE	6		Risk Ratio (M‐H, Random, 95% CI)	Subtotals only
7.5.1 TXA, IV 1 g	4	283	Risk Ratio (M‐H, Random, 95% CI)	Not estimable
7.5.2 TXA, IV 2 g	2	166	Risk Ratio (M‐H, Random, 95% CI)	Not estimable
7.5.3 TXA, IV 3 g	1	100	Risk Ratio (M‐H, Random, 95% CI)	Not estimable
7.6 Risk of experiencing CVA	1		Risk Ratio (M‐H, Random, 95% CI)	Subtotals only
7.6.1 TXA, IV 1 g	1	89	Risk Ratio (M‐H, Random, 95% CI)	Not estimable
7.6.2 TXA, IV 2 g	1	86	Risk Ratio (M‐H, Random, 95% CI)	Not estimable

**Comparison 8:** TXA IV vs TXA topical

Outcome or subgroup title	No. of studies	No. of participants	Statistical method	Effect size
8.1 Risk of allogeneic blood transfusion	9		Risk Ratio (M‐H, Random, 95% CI)	Subtotals only
8.1.1 TXA, IV vs TXA, topical 400 mg	1	8	Risk Ratio (M‐H, Random, 95% CI)	Not estimable
8.1.2 TXA, IV vs TXA, topical 800 mg	1	7	Risk Ratio (M‐H, Random, 95% CI)	Not estimable
8.1.3 TXA, IV vs TXA, topical 1 g	1	93	Risk Ratio (M‐H, Random, 95% CI)	Not estimable
8.1.4 TXA, IV vs TXA, topical 1200 mg	1	8	Risk Ratio (M‐H, Random, 95% CI)	Not estimable
8.1.5 TXA, IV vs TXA, topical 2 g	4	352	Risk Ratio (M‐H, Random, 95% CI)	0.64 [0.28, 1.42]
8.1.6 TXA, IV vs TXA, topical 3 g	3	351	Risk Ratio (M‐H, Random, 95% CI)	5.00 [0.25, 102.00]
8.2 All‐cause mortality	4		Peto Odds Ratio (Peto, Fixed, 95% CI)	Subtotals only
8.2.1 TXA, IV vs TXA, topical 400 mg	1	8	Peto Odds Ratio (Peto, Fixed, 95% CI)	Not estimable
8.2.2 TXA, IV vs TXA, topical 800 mg	1	7	Peto Odds Ratio (Peto, Fixed, 95% CI)	Not estimable
8.2.3 TXA, IV vs TXA, topical 1200 mg	1	8	Peto Odds Ratio (Peto, Fixed, 95% CI)	Not estimable
8.2.4 TXA, IV vs TXA, topical 2 g	1	78	Peto Odds Ratio (Peto, Fixed, 95% CI)	Not estimable
8.2.5 TXA, IV vs TXA, topical 3 g	2	126	Peto Odds Ratio (Peto, Fixed, 95% CI)	0.14 [0.00, 7.04]
8.3 Reoperation	2		Risk Ratio (M‐H, Random, 95% CI)	Subtotals only
8.3.1 TXA, IV vs TXA, topical 400 mg	1	8	Risk Ratio (M‐H, Random, 95% CI)	Not estimable
8.3.2 TXA, IV vs TXA, topical 800 mg	1	7	Risk Ratio (M‐H, Random, 95% CI)	Not estimable
8.3.3 TXA, IV vs TXA, topical 1 g	1	93	Risk Ratio (M‐H, Random, 95% CI)	Not estimable
8.3.4 TXA, IV vs TXA, topical 1200 mg	1	8	Risk Ratio (M‐H, Random, 95% CI)	Not estimable
8.4 Length of hospital stay	3		Mean Difference (IV, Random, 95% CI)	Subtotals only
8.4.1 TXA, IV vs TXA, topical 3 g	2	288	Mean Difference (IV, Random, 95% CI)	‐0.20 [‐0.59, 0.19]
8.4.2 TXA, IV vs TXA, topical 2 g	1	78	Mean Difference (IV, Random, 95% CI)	0.40 [‐0.18, 0.98]
8.5 Risk of experiencing DVT	8		Peto Odds Ratio (Peto, Fixed, 95% CI)	Subtotals only
8.5.1 TXA, IV vs TXA, topical 1 g	1	93	Peto Odds Ratio (Peto, Fixed, 95% CI)	1.49 [0.25, 8.92]
8.5.2 TXA, IV vs TXA, topical 2 g	4	353	Peto Odds Ratio (Peto, Fixed, 95% CI)	0.14 [0.00, 6.82]
8.5.3 TXA, IV vs TXA, topical 3 g	3	350	Peto Odds Ratio (Peto, Fixed, 95% CI)	0.98 [0.19, 4.94]
8.6 Risk of experiencing PE	7		Peto Odds Ratio (Peto, Fixed, 95% CI)	Subtotals only
8.6.1 TXA, IV vs TXA, topical 1 g	1	93	Peto Odds Ratio (Peto, Fixed, 95% CI)	Not estimable
8.6.2 TXA, IV vs TXA, topical 2 g	4	353	Peto Odds Ratio (Peto, Fixed, 95% CI)	1.13 [0.16, 8.15]
8.6.3 TXA, IV vs TXA, topical 3 g	2	182	Peto Odds Ratio (Peto, Fixed, 95% CI)	Not estimable
8.7 Risk of experiencing MI	3		Peto Odds Ratio (Peto, Fixed, 95% CI)	Subtotals only
8.7.1 TXA, IV vs TXA, topical 2 g	2	164	Peto Odds Ratio (Peto, Fixed, 95% CI)	0.50 [0.05, 4.90]
8.7.2 TXA, IV vs TXA, topical 1 g	1	93	Peto Odds Ratio (Peto, Fixed, 95% CI)	Not estimable
8.8 Risk of experiencing CVA	3		Risk Ratio (M‐H, Random, 95% CI)	Subtotals only
8.8.1 TXA, IV vs TXA, topical 400 mg	1	7	Risk Ratio (M‐H, Random, 95% CI)	Not estimable
8.8.2 TXA, IV vs TXA, topical 800 mg	1	6	Risk Ratio (M‐H, Random, 95% CI)	Not estimable
8.8.3 TXA, IV vs TXA, topical 1200 mg	1	7	Risk Ratio (M‐H, Random, 95% CI)	Not estimable
8.8.4 TXA, IV vs TXA, topical 1 g	1	93	Risk Ratio (M‐H, Random, 95% CI)	Not estimable
8.8.5 TXA, IV vs TXA, topical 2 g	1	25	Risk Ratio (M‐H, Random, 95% CI)	Not estimable
8.9 Risk of suspected serious drug reactions	3		Risk Ratio (M‐H, Random, 95% CI)	Subtotals only
8.9.1 TXA, IV vs TXA, topical 400 mg	1	8	Risk Ratio (M‐H, Random, 95% CI)	Not estimable
8.9.2 TXA, IV vs TXA, topical 800 mg	1	7	Risk Ratio (M‐H, Random, 95% CI)	Not estimable
8.9.3 TXA, IV vs TXA, topical 1200 mg	1	8	Risk Ratio (M‐H, Random, 95% CI)	Not estimable
8.9.4 TXA, IV vs TXA, topical 2 g	1	25	Risk Ratio (M‐H, Random, 95% CI)	Not estimable
8.9.5 TXA, IV vs TXA, topical 3 g	1	63	Risk Ratio (M‐H, Random, 95% CI)	Not estimable

**Comparison 9:** TXA oral lower dose vs TXA oral higher dose

Outcome or subgroup title	No. of studies	No. of participants	Statistical method	Effect size
9.1 Risk of allogeneic blood transfusion	2		Risk Ratio (M‐H, Random, 95% CI)	Subtotals only
9.1.1 TXA, oral 2 g vs TXA, oral 4 g	1	102	Risk Ratio (M‐H, Random, 95% CI)	Not estimable
9.1.2 TXA, oral 2 g vs TXA, oral more than 4 g	1	101	Risk Ratio (M‐H, Random, 95% CI)	Not estimable
9.1.3 TXA, oral 4 g vs TXA, oral more than 4 g	1	101	Risk Ratio (M‐H, Random, 95% CI)	Not estimable
9.1.4 TXA, oral 1 g vs TXA, oral 2 g	1	100	Risk Ratio (M‐H, Random, 95% CI)	Not estimable
9.1.5 TXA, oral 1 g vs TXA, oral 3 g	1	100	Risk Ratio (M‐H, Random, 95% CI)	Not estimable
9.1.6 TXA, oral 2 g vs TXA, oral 3 g	1	100	Risk Ratio (M‐H, Random, 95% CI)	Not estimable
9.2 All‐cause mortality	1		Risk Ratio (M‐H, Random, 95% CI)	Subtotals only
9.2.1 TXA, oral 1 g vs TXA, oral 2 g	1	100	Risk Ratio (M‐H, Random, 95% CI)	Not estimable
9.2.2 TXA, Oral 1g vs TXA, Oral 3g	1	100	Risk Ratio (M‐H, Random, 95% CI)	Not estimable
9.2.3 TXA, Oral 2g vs TXA, Oral 3g	1	100	Risk Ratio (M‐H, Random, 95% CI)	Not estimable
9.3 Risk of experiencing DVT	2		Peto Odds Ratio (Peto, Fixed, 95% CI)	Subtotals only
9.3.1 TXA, oral 2 g vs TXA, oral 4 g	1	102	Peto Odds Ratio (Peto, Fixed, 95% CI)	7.39 [0.15, 372.38]
9.3.2 TXA, oral 2 g vs TXA, oral more than 4 g	1	101	Peto Odds Ratio (Peto, Fixed, 95% CI)	0.98 [0.06, 15.89]
9.3.3 TXA, oral 4 g vs TXA, oral more than 4 g	1	101	Peto Odds Ratio (Peto, Fixed, 95% CI)	0.13 [0.00, 6.69]
9.3.4 TXA, oral 1 g vs TXA, oral 2 g	1	100	Peto Odds Ratio (Peto, Fixed, 95% CI)	Not estimable
9.3.5 TXA, oral 1 g vs TXA, oral 3 g	1	100	Peto Odds Ratio (Peto, Fixed, 95% CI)	Not estimable
9.3.6 TXA, oral 2 g vs TXA, oral 3 g	1	100	Peto Odds Ratio (Peto, Fixed, 95% CI)	Not estimable
9.4 Risk of experiencing PE	2		Risk Ratio (M‐H, Random, 95% CI)	Subtotals only
9.4.1 TXA, oral 2 g vs TXA, oral 4 g	1	102	Risk Ratio (M‐H, Random, 95% CI)	Not estimable
9.4.2 TXA, oral 2 g vs TXA, oral more than 4 g	1	101	Risk Ratio (M‐H, Random, 95% CI)	Not estimable
9.4.3 TXA, oral 4 g vs TXA, oral, more than 4 g	1	101	Risk Ratio (M‐H, Random, 95% CI)	Not estimable
9.4.4 TXA, oral 1 g vs TXA, oral 2 g	1	100	Risk Ratio (M‐H, Random, 95% CI)	Not estimable
9.4.5 TXA, oral 1 g vs TXA, oral 3 g	1	100	Risk Ratio (M‐H, Random, 95% CI)	Not estimable
9.4.6 TXA, oral 2 g vs TXA, oral 3 g	1	100	Risk Ratio (M‐H, Random, 95% CI)	Not estimable
9.5 Risk of experiencing MI	1		Risk Ratio (M‐H, Random, 95% CI)	Subtotals only
9.5.1 TXA, oral 2 g vs TXA, oral 4 g	1	102	Risk Ratio (M‐H, Random, 95% CI)	Not estimable
9.5.2 TXA, oral 2 g vs TXA, oral more than 4 g	1	101	Risk Ratio (M‐H, Random, 95% CI)	Not estimable
9.5.3 TXA, oral 4 g vs TXA, oral more than 4 g	1	101	Risk Ratio (M‐H, Random, 95% CI)	Not estimable
9.6 Risk of experiencing CVA	2		Risk Ratio (M‐H, Random, 95% CI)	Subtotals only
9.6.1 TXA, oral 2 g vs TXA, oral 4 g	1	102	Risk Ratio (M‐H, Random, 95% CI)	Not estimable
9.6.2 TXA, oral 2 g vs TXA, oral more than 4 g	1	101	Risk Ratio (M‐H, Random, 95% CI)	Not estimable
9.6.3 TXA, oral 4 g vs TXA, oral more than 4 g	1	101	Risk Ratio (M‐H, Random, 95% CI)	Not estimable
9.6.4 TXA, oral 1 g vs TXA, oral 2 g	1	100	Risk Ratio (M‐H, Random, 95% CI)	Not estimable
9.6.5 TXA, oral 1 g vs TXA, oral 3 g	1	100	Risk Ratio (M‐H, Random, 95% CI)	Not estimable
9.6.6 TXA, oral 2 g vs TXA, oral 3 g	1	100	Risk Ratio (M‐H, Random, 95% CI)	Not estimable

**Comparison 10:** TXA topical lower dose vs TXA topical higher dose

Outcome or subgroup title	No. of studies	No. of participants	Statistical method	Effect size
10.1 Risk of allogeneic blood transfusion	1		Risk Ratio (M‐H, Random, 95% CI)	Subtotals only
10.1.1 TXA, topical 400 mg vs TXA, topical 800 mg	1	7	Risk Ratio (M‐H, Random, 95% CI)	Not estimable
10.1.2 TXA, topical 400 mg vs TXA, topical 1200 mg	1	8	Risk Ratio (M‐H, Random, 95% CI)	Not estimable
10.1.3 TXA, topical 800 mg vs TXA, topical 1200 mg	1	7	Risk Ratio (M‐H, Random, 95% CI)	Not estimable
10.2 All‐cause mortality	1		Risk Ratio (M‐H, Random, 95% CI)	Subtotals only
10.2.1 TXA, topical 400 mg vs TXA, topical 800 mg	1	7	Risk Ratio (M‐H, Random, 95% CI)	Not estimable
10.2.2 TXA, topical 400 mg vs TXA, topical 1200 mg	1	8	Risk Ratio (M‐H, Random, 95% CI)	Not estimable
10.2.3 TXA, topical 800 mg vs TXA, topical 1200 mg	1	7	Risk Ratio (M‐H, Random, 95% CI)	Not estimable
10.3 Reoperation	1		Risk Ratio (M‐H, Random, 95% CI)	Subtotals only
10.3.1 TXA, topical 400 mg vs TXA, topical 800 mg	1	7	Risk Ratio (M‐H, Random, 95% CI)	Not estimable
10.3.2 TXA, topical 400 mg vs TXA, topical 1200 mg	1	8	Risk Ratio (M‐H, Random, 95% CI)	Not estimable
10.3.3 TXA, topical 800 mg vs TXA, topical 1200 mg	1	7	Risk Ratio (M‐H, Random, 95% CI)	Not estimable
10.4 Risk of experiencing CVA	1		Risk Ratio (M‐H, Random, 95% CI)	Subtotals only
10.4.1 TXA, topical 400 mg vs TXA, topical 800 mg	1	7	Risk Ratio (M‐H, Random, 95% CI)	Not estimable
10.4.2 TXA, topical 400 mg vs TXA, topical 1200 mg	1	8	Risk Ratio (M‐H, Random, 95% CI)	Not estimable
10.4.3 TXA, topical 800 mg vs TXA, topical 1200 mg	1	7	Risk Ratio (M‐H, Random, 95% CI)	Not estimable
10.5 Risk of suspected serious drug reactions	1		Risk Ratio (M‐H, Random, 95% CI)	Subtotals only
10.5.1 TXA, topical 400 mg vs TXA, topical 800 mg	1	7	Risk Ratio (M‐H, Random, 95% CI)	Not estimable
10.5.2 TXA, topical 400 mg vs TXA, topical 1200 mg	1	8	Risk Ratio (M‐H, Random, 95% CI)	Not estimable
10.5.3 TXA, topical 800 mg vs TXA, topical 1200 mg	1	7	Risk Ratio (M‐H, Random, 95% CI)	Not estimable

**Comparison 11:** TXA IV vs aprotinin

Outcome or subgroup title	No. of studies	No. of participants	Statistical method	Effect size
11.1 Risk of allogeneic blood transfusion	1		Risk Ratio (M‐H, Random, 95% CI)	Subtotals only
11.1.1 TXA, IV 2 g	1	24	Risk Ratio (M‐H, Random, 95% CI)	0.09 [0.01, 1.48]
11.2 Risk of experiencing DVT	1		Risk Ratio (M‐H, Random, 95% CI)	Subtotals only
11.2.1 TXA, IV 2 g	1	24	Risk Ratio (M‐H, Random, 95% CI)	2.00 [0.21, 19.23]

**Comparison 12:** TXA IV vs EACA

Outcome or subgroup title	No. of studies	No. of participants	Statistical method	Effect size
12.1 Risk of allogeneic blood transfusion	4		Peto Odds Ratio (Peto, Fixed, 95% CI)	Subtotals only
12.1.1 TXA, IV 2 g	3	430	Peto Odds Ratio (Peto, Fixed, 95% CI)	0.13 [0.00, 6.73]
12.1.2 TXA, IV 1 g	1	67	Peto Odds Ratio (Peto, Fixed, 95% CI)	0.25 [0.04, 1.54]
12.2 Units of red blood cells transfused	1		Mean Difference (IV, Random, 95% CI)	Subtotals only
12.2.1 TXA, IV 1 g	1	67	Mean Difference (IV, Random, 95% CI)	‐0.16 [‐0.35, 0.03]
12.3 Reoperation	1		Risk Ratio (M‐H, Random, 95% CI)	Subtotals only
12.3.1 TXA, IV 2 g	1	91	Risk Ratio (M‐H, Random, 95% CI)	Not estimable
12.4 Length of hospital stay	1		Mean Difference (IV, Random, 95% CI)	Subtotals only
12.4.1 TXA, IV 2 g	1	194	Mean Difference (IV, Random, 95% CI)	‐0.11 [‐0.39, 0.17]
12.5 Risk of experiencing DVT	4		Peto Odds Ratio (Peto, Fixed, 95% CI)	Subtotals only
12.5.1 TXA, IV 2 g	3	429	Peto Odds Ratio (Peto, Fixed, 95% CI)	0.13 [0.00, 6.68]
12.5.2 TXA, IV 1 g	1	67	Peto Odds Ratio (Peto, Fixed, 95% CI)	Not estimable
12.6 Risk of experiencing PE	4		Peto Odds Ratio (Peto, Fixed, 95% CI)	Subtotals only
12.6.1 TXA, IV 1 g	1	67	Peto Odds Ratio (Peto, Fixed, 95% CI)	Not estimable
12.6.2 TXA, IV 2 g	3	429	Peto Odds Ratio (Peto, Fixed, 95% CI)	0.98 [0.20, 4.91]
12.7 Risk of experiencing MI	3		Peto Odds Ratio (Peto, Fixed, 95% CI)	Subtotals only
12.7.1 TXA, IV 1 g	1	67	Peto Odds Ratio (Peto, Fixed, 95% CI)	Not estimable
12.7.2 TXA, IV 2 g	2	235	Peto Odds Ratio (Peto, Fixed, 95% CI)	7.29 [0.14, 367.35]
12.8 Risk of experiencing CVA	4		Peto Odds Ratio (Peto, Fixed, 95% CI)	Subtotals only
12.8.1 TXA, IV 1 g	1	67	Peto Odds Ratio (Peto, Fixed, 95% CI)	Not estimable
12.8.2 TXA, IV 2 g	3	429	Peto Odds Ratio (Peto, Fixed, 95% CI)	0.13 [0.00, 6.68]

**Comparison 13:** TXA oral vs EACA oral

Outcome or subgroup title	No. of studies	No. of participants	Statistical method	Effect size
13.1 Risk of allogeneic blood transfusion	1		Risk Ratio (M‐H, Random, 95% CI)	Subtotals only
13.2 All‐cause mortality	1		Risk Ratio (M‐H, Random, 95% CI)	Subtotals only
13.3 Reoperation	1		Risk Ratio (M‐H, Random, 95% CI)	Subtotals only
13.4 Risk of experiencing DVT	1		Risk Ratio (M‐H, Random, 95% CI)	Subtotals only
13.5 Risk of experiencing PE	1		Risk Ratio (M‐H, Random, 95% CI)	Subtotals only
13.6 Risk of experiencing MI	1		Risk Ratio (M‐H, Random, 95% CI)	Subtotals only
13.7 Risk of experiencing CVA	1		Risk Ratio (M‐H, Random, 95% CI)	Subtotals only
13.8 Risk of suspected serious drug reactions	1		Risk Ratio (M‐H, Random, 95% CI)	Subtotals only

**Comparison 14:** TXA IV vs desmopressin

Outcome or subgroup title	No. of studies	No. of participants	Statistical method	Effect size
14.1 Risk of allogeneic blood transfusion	1		Risk Ratio (M‐H, Random, 95% CI)	Subtotals only
14.1.1 TXA, IV 1 g	1	40	Risk Ratio (M‐H, Random, 95% CI)	0.27 [0.09, 0.83]
14.2 Length of hospital stay	1		Mean Difference (IV, Random, 95% CI)	Subtotals only
14.2.1 TXA, IV 1 g	1	40	Mean Difference (IV, Random, 95% CI)	‐1.00 [‐2.24, 0.24]
14.3 Risk of experiencing DVT	1		Risk Ratio (M‐H, Random, 95% CI)	Subtotals only
14.3.1 TXA, IV 1 g	1	40	Risk Ratio (M‐H, Random, 95% CI)	Not estimable
14.4 Risk of experiencing PE	1		Risk Ratio (M‐H, Random, 95% CI)	Subtotals only
14.4.1 TXA, IV 1 g	1	40	Risk Ratio (M‐H, Random, 95% CI)	Not estimable

**Comparison 15:** TXA IV vs fibrin topical

Outcome or subgroup title	No. of studies	No. of participants	Statistical method	Effect size
15.1 Risk of allogeneic blood transfusion	1		Risk Ratio (M‐H, Random, 95% CI)	Subtotals only
15.1.1 TXA, IV 1 g	1	100	Risk Ratio (M‐H, Random, 95% CI)	0.71 [0.24, 2.10]
15.2 All‐cause mortality	1		Risk Ratio (M‐H, Random, 95% CI)	Subtotals only
15.2.1 TXA, IV 1 g	1	100	Risk Ratio (M‐H, Random, 95% CI)	Not estimable
15.3 Units of red blood cells transfused	1		Mean Difference (IV, Random, 95% CI)	Subtotals only
15.3.1 TXA, IV 1 g	1	100	Mean Difference (IV, Random, 95% CI)	‐0.06 [‐0.28, 0.16]
15.5 Risk of experiencing DVT	1		Risk Ratio (M‐H, Random, 95% CI)	Subtotals only
15.5.1 TXA, IV 1 g	1	100	Risk Ratio (M‐H, Random, 95% CI)	0.33 [0.01, 7.99]
15.6 Risk of experiencing PE	1		Risk Ratio (M‐H, Random, 95% CI)	Subtotals only
15.6.1 TXA, IV 1 g	1	100	Risk Ratio (M‐H, Random, 95% CI)	0.33 [0.01, 7.99]

**Comparison 16:** TXA topical vs fibrin topical

Outcome or subgroup title	No. of studies	No. of participants	Statistical method	Effect size
16.1 Risk of allogeneic blood transfusion	1		Risk Ratio (M‐H, Random, 95% CI)	Subtotals only
16.2 All‐cause mortality	1		Risk Ratio (M‐H, Random, 95% CI)	Subtotals only
16.3 Reoperation	1		Risk Ratio (M‐H, Random, 95% CI)	Subtotals only
16.4 Length of hospital stay	1		Mean Difference (IV, Random, 95% CI)	Subtotals only
16.5 Risk of experiencing DVT	1		Risk Ratio (M‐H, Random, 95% CI)	Subtotals only
16.6 Risk of experiencing PE	1	68	Risk Ratio (M‐H, Random, 95% CI)	Not estimable
16.7 Risk of experiencing CVA	1	68	Risk Ratio (M‐H, Random, 95% CI)	Not estimable

**Comparison 17:** Aprotinin vs placebo

Outcome or subgroup title	No. of studies	No. of participants	Statistical method	Effect size
17.1 Risk of allogeneic blood transfusion	9	727	Risk Ratio (M‐H, Random, 95% CI)	0.70 [0.50, 0.97]
17.2 All‐cause mortality	2	500	Peto Odds Ratio (Peto, Fixed, 95% CI)	0.13 [0.01, 2.15]
17.3 Units of red blood cells transfused	8	602	Mean Difference (IV, Random, 95% CI)	‐0.94 [‐1.58, ‐0.29]
17.4 Reoperation	1	30	Peto Odds Ratio (Peto, Fixed, 95% CI)	0.14 [0.00, 6.82]
17.5 Length of hospital stay	1	40	Mean Difference (IV, Random, 95% CI)	0.70 [‐1.71, 3.11]
17.6 Risk of experiencing DVT	9	745	Peto Odds Ratio (Peto, Fixed, 95% CI)	0.60 [0.26, 1.37]
17.7 Risk of experiencing PE	3	422	Peto Odds Ratio (Peto, Fixed, 95% CI)	0.52 [0.05, 5.01]
17.8 Risk of experiencing MI	2	500	Peto Odds Ratio (Peto, Fixed, 95% CI)	0.51 [0.05, 4.93]
17.9 Risk of experiencing CVA	2	405	Peto Odds Ratio (Peto, Fixed, 95% CI)	0.11 [0.00, 5.64]
17.10 Risk of suspected serious drug reactions	3	228	Risk Ratio (M‐H, Random, 95% CI)	1.04 [0.71, 1.52]

**Comparison 18:** EACA vs placebo

Outcome or subgroup title	No. of studies	No. of participants	Statistical method	Effect size
18.1 Risk of allogeneic blood transfusion	3	168	Risk Ratio (M‐H, Random, 95% CI)	0.59 [0.27, 1.28]
18.2 Units of red blood cells transfused	1	92	Mean Difference (IV, Random, 95% CI)	‐0.39 [‐0.66, ‐0.12]
18.3 Reoperation	1	30	Peto Odds Ratio (Peto, Fixed, 95% CI)	0.14 [0.00, 6.82]
18.4 Risk of experiencing DVT	3		Risk Ratio (M‐H, Random, 95% CI)	Subtotals only
18.5 Risk of experiencing PE	3		Risk Ratio (M‐H, Random, 95% CI)	Subtotals only
18.6 Risk of experiencing MI	1		Risk Ratio (M‐H, Random, 95% CI)	Subtotals only
18.7 Risk of experiencing CVA	1		Risk Ratio (M‐H, Random, 95% CI)	Subtotals only

**Comparison 19:** EACA vs aprotinin

Outcome or subgroup title	No. of studies	No. of participants	Statistical method	Effect size
19.1 Risk of allogeneic blood transfusion	1	30	Risk Ratio (M‐H, Random, 95% CI)	2.00 [0.43, 9.32]
19.2 Reoperation	1		Risk Ratio (M‐H, Random, 95% CI)	Subtotals only
19.3 Risk of experiencing DVT	1		Risk Ratio (M‐H, Random, 95% CI)	Subtotals only
19.4 Risk of experiencing PE	1		Risk Ratio (M‐H, Random, 95% CI)	Subtotals only

**Comparison 20:** Desmopressin vs placebo

Outcome or subgroup title	No. of studies	No. of participants	Statistical method	Effect size
20.1 All‐cause mortality	2		Risk Ratio (M‐H, Random, 95% CI)	Subtotals only
20.2 Units of red blood cells transfused	2	129	Mean Difference (IV, Random, 95% CI)	‐0.15 [‐0.64, 0.33]
20.3 Risk of experiencing DVT	2		Risk Ratio (M‐H, Random, 95% CI)	Subtotals only
20.4 Risk of experiencing PE	1	79	Risk Ratio (M‐H, Random, 95% CI)	3.08 [0.13, 73.27]
20.5 Risk of experiencing MI	3		Risk Ratio (M‐H, Random, 95% CI)	Subtotals only
20.6 Risk of suspected serious drug reactions	2		Risk Ratio (M‐H, Random, 95% CI)	Subtotals only
20.7 Length of hospital stay	2		Mean Difference (IV, Random, 95% CI)	Subtotals only

**Comparison 21:** Fibrin topical vs placebo

Outcome or subgroup title	No. of studies	No. of participants	Statistical method	Effect size
21.1 Risk of allogeneic blood transfusion	1	69	Risk Ratio (M‐H, Random, 95% CI)	3.09 [0.34, 28.25]
21.2 All‐cause mortality	1		Risk Ratio (M‐H, Random, 95% CI)	Subtotals only
21.3 Reoperation	1		Risk Ratio (M‐H, Random, 95% CI)	Subtotals only
21.4 Length of hospital stay	1	69	Mean Difference (IV, Random, 95% CI)	0.07 [‐0.15, 0.29]
21.5 Risk of experiencing DVT	1		Risk Ratio (M‐H, Random, 95% CI)	Subtotals only
21.6 Risk of experiencing PE	1		Risk Ratio (M‐H, Random, 95% CI)	Subtotals only
21.7 Risk of experiencing CVA	1		Risk Ratio (M‐H, Random, 95% CI)	Subtotals only

**Comparison 22:** TXA IV + TXA oral vs TXA IV

Outcome or subgroup title	No. of studies	No. of participants	Statistical method	Effect size
22.1 Risk of allogeneic blood transfusion	2		Peto Odds Ratio (Peto, Fixed, 95% CI)	Subtotals only
22.1.1 TXA, IV + TXA oral days 1 to 2 vs TXA, IV	1	94	Peto Odds Ratio (Peto, Fixed, 95% CI)	1.04 [0.06, 16.95]
22.1.2 TXA, IV + TXA oral days 1 to 5 vs TXA, IV	1	95	Peto Odds Ratio (Peto, Fixed, 95% CI)	1.02 [0.06, 16.58]
22.1.3 TXA IV + TXA oral days 1 to 14 vs TXA IV	1	118	Peto Odds Ratio (Peto, Fixed, 95% CI)	Not estimable
22.2 All‐cause mortality	1		Peto Odds Ratio (Peto, Fixed, 95% CI)	Subtotals only
22.3 Reoperation	1		Risk Ratio (M‐H, Random, 95% CI)	Subtotals only
22.4 Length of hospital stay	1		Mean Difference (IV, Random, 95% CI)	Totals not selected
22.5 Risk of experiencing DVT	2		Risk Ratio (M‐H, Random, 95% CI)	Subtotals only
22.5.1 TXA, IV + TXA oral days 1 to 2 vs TXA, IV	1	93	Risk Ratio (M‐H, Random, 95% CI)	0.15 [0.01, 2.75]
22.5.2 TXA, IV + TXA oral days 1 to 5 vs TXA, IV	1	94	Risk Ratio (M‐H, Random, 95% CI)	0.67 [0.12, 3.81]
22.5.3 TXA IV + TXA oral days 1 to 14 vs TXA IV	1	118	Risk Ratio (M‐H, Random, 95% CI)	1.61 [0.40, 6.44]
22.6 Risk of experiencing PE	1		Peto Odds Ratio (Peto, Fixed, 95% CI)	Subtotals only
22.6.1 TXA, IV + TXA oral days 1 to 2 vs TXA, IV	1	94	Peto Odds Ratio (Peto, Fixed, 95% CI)	0.14 [0.00, 7.12]
22.6.2 TXA, IV + TXA oral days 1 to 5 vs TXA, IV	1	95	Peto Odds Ratio (Peto, Fixed, 95% CI)	0.14 [0.00, 6.97]

**Comparison 23:** TXA IV + TXA topical vs TXA IV

Outcome or subgroup title	No. of studies	No. of participants	Statistical method	Effect size
23.1 Risk of allogeneic blood transfusion	2		Risk Ratio (M‐H, Random, 95% CI)	Subtotals only
23.1.1 TXA, IV 1 g	2	149	Risk Ratio (M‐H, Random, 95% CI)	0.60 [0.17, 2.08]
23.1.2 TXA, IV 2 g	1	86	Risk Ratio (M‐H, Random, 95% CI)	0.87 [0.36, 2.10]
23.2 Length of hospital stay	2		Mean Difference (IV, Random, 95% CI)	Subtotals only
23.2.1 TXA, IV 1 g	2	149	Mean Difference (IV, Random, 95% CI)	0.10 [‐0.40, 0.59]
23.2.2 TXA, IV 2 g	1	86	Mean Difference (IV, Random, 95% CI)	‐0.20 [‐1.49, 1.09]
23.3 Risk of experiencing DVT	2		Risk Ratio (M‐H, Random, 95% CI)	Subtotals only
23.3.1 TXA, IV 1 g	2	149	Risk Ratio (M‐H, Random, 95% CI)	Not estimable
23.3.2 TXA, IV 2 g	1	86	Risk Ratio (M‐H, Random, 95% CI)	Not estimable
23.4 Risk of experiencing PE	2		Risk Ratio (M‐H, Random, 95% CI)	Subtotals only
23.4.1 TXA, IV 1 g	2	149	Risk Ratio (M‐H, Random, 95% CI)	Not estimable
23.4.2 TXA, IV 2 g	1	86	Risk Ratio (M‐H, Random, 95% CI)	Not estimable
23.5 Risk of experiencing CVA	1		Risk Ratio (M‐H, Random, 95% CI)	Subtotals only
23.5.1 TXA, IV 1 g	1	89	Risk Ratio (M‐H, Random, 95% CI)	Not estimable
23.5.2 TXA, IV 2 g	1	86	Risk Ratio (M‐H, Random, 95% CI)	Not estimable

**Comparison 24:** TXA IV + TXA topical vs TXA oral

Outcome or subgroup title	No. of studies	No. of participants	Statistical method	Effect size
24.1 Risk of allogeneic blood transfusion	1		Risk Ratio (M‐H, Random, 95% CI)	Subtotals only
24.2 Length of hospital stay	1		Mean Difference (IV, Random, 95% CI)	Totals not selected
24.3 Risk of experiencing DVT	1		Peto Odds Ratio (Peto, Fixed, 95% CI)	Subtotals only
24.4 Risk of experiencing PE	1		Risk Ratio (M‐H, Random, 95% CI)	Subtotals only
24.5 Risk of experiencing CVA	1		Risk Ratio (M‐H, Random, 95% CI)	Subtotals only

**Comparison 25:** TXA IV + TXA topical vs TXA topical

Outcome or subgroup title	No. of studies	No. of participants	Statistical method	Effect size
25.1 Risk of allogeneic blood transfusion	1		Peto Odds Ratio (Peto, Fixed, 95% CI)	Subtotals only

**Comparison 26:** TXA topical vs TXA oral + TXA topical

Outcome or subgroup title	No. of studies	No. of participants	Statistical method	Effect size
26.1 Risk of allogeneic blood transfusion	1		Peto Odds Ratio (Peto, Fixed, 95% CI)	Subtotals only
26.1.1 TXA, topical vs TXA, oral + TXA, topical 4 g	1	120	Peto Odds Ratio (Peto, Fixed, 95% CI)	1.97 [0.20, 19.31]
26.1.2 TXA, topical vs TXA, oral + TXA, topical 5 g	1	120	Peto Odds Ratio (Peto, Fixed, 95% CI)	1.97 [0.20, 19.31]
26.1.3 TXA, topical vs TXA, oral + TXA, topical 6 g	1	120	Peto Odds Ratio (Peto, Fixed, 95% CI)	7.52 [0.46, 121.57]
26.1.4 TXA, topical vs TXA, oral + TXA, topical 7 g	1	120	Peto Odds Ratio (Peto, Fixed, 95% CI)	7.52 [0.46, 121.57]
26.2 All‐cause mortality	1		Risk Ratio (M‐H, Random, 95% CI)	Subtotals only
26.2.1 TXA, topical vs TXA, oral + TXA, topical 4 g	1	120	Risk Ratio (M‐H, Random, 95% CI)	Not estimable
26.2.2 TXA, topical vs TXA, oral + TXA, topical 5 g	1	120	Risk Ratio (M‐H, Random, 95% CI)	Not estimable
26.2.3 TXA, topical vs TXA, oral + TXA, topical 6 g	1	120	Risk Ratio (M‐H, Random, 95% CI)	Not estimable
26.2.4 TXA, topical vs TXA, oral + TXA, topical 7 g	1	120	Risk Ratio (M‐H, Random, 95% CI)	Not estimable
26.3 Length of hospital stay	1		Mean Difference (IV, Random, 95% CI)	Subtotals only
26.3.1 TXA, topical vs TXA, oral + TXA, topical 4 g	1	120	Mean Difference (IV, Random, 95% CI)	0.10 [‐0.22, 0.42]
26.3.2 TXA, topical vs TXA, oral + TXA, topical 5 g	1	120	Mean Difference (IV, Random, 95% CI)	0.30 [‐0.06, 0.66]
26.3.3 TXA, topical vs TXA, oral + TXA, topical 6 g	1	120	Mean Difference (IV, Random, 95% CI)	0.30 [‐0.02, 0.62]
26.3.4 TXA, topical vs TXA, oral + TXA, topical 7 g	1	120	Mean Difference (IV, Random, 95% CI)	0.30 [‐0.04, 0.64]
26.4 Risk of experiencing DVT	1		Peto Odds Ratio (Peto, Fixed, 95% CI)	Subtotals only
26.4.1 TXA, topical vs TXA, oral + TXA, topical 4 g	1	120	Peto Odds Ratio (Peto, Fixed, 95% CI)	0.51 [0.05, 4.97]
26.4.2 TXA, topical vs TXA, oral + TXA, topical 5 g	1	120	Peto Odds Ratio (Peto, Fixed, 95% CI)	1.00 [0.06, 16.18]
26.4.3 TXA, topical vs TXA, oral + TXA, topical 6 g	1	120	Peto Odds Ratio (Peto, Fixed, 95% CI)	0.51 [0.05, 4.97]
26.4.4 TXA, topical vs TXA, oral + TXA, topical 7 g	1	120	Peto Odds Ratio (Peto, Fixed, 95% CI)	0.51 [0.05, 4.97]
26.5 Risk of experiencing PE	1		Risk Ratio (M‐H, Random, 95% CI)	Subtotals only
26.5.1 TXA, topical vs TXA, oral + TXA, topical 4 g	1	120	Risk Ratio (M‐H, Random, 95% CI)	Not estimable
26.5.2 TXA, topical vs TXA, oral + TXA, topical 5 g	1	120	Risk Ratio (M‐H, Random, 95% CI)	Not estimable
26.5.3 TXA, topical vs TXA, oral + TXA, topical 6 g	1	120	Risk Ratio (M‐H, Random, 95% CI)	Not estimable
26.5.4 TXA, topical vs TXA, oral + TXA, topical 7 g	1	120	Risk Ratio (M‐H, Random, 95% CI)	Not estimable
26.6 Risk of experiencing MI	1		Risk Ratio (M‐H, Random, 95% CI)	Subtotals only
26.6.1 TXA, topical vs TXA, oral + TXA, topical 4 g	1	120	Risk Ratio (M‐H, Random, 95% CI)	Not estimable
26.6.2 TXA, topical vs TXA, oral + TXA, topical 5 g	1	120	Risk Ratio (M‐H, Random, 95% CI)	Not estimable
26.6.3 TXA, topical vs TXA, oral + TXA, topical 6 g	1	120	Risk Ratio (M‐H, Random, 95% CI)	Not estimable
26.6.4 TXA, topical vs TXA, oral + TXA, topical 7 g	1	120	Risk Ratio (M‐H, Random, 95% CI)	Not estimable
26.7 Risk of experiencing CVA	1		Risk Ratio (M‐H, Random, 95% CI)	Subtotals only
26.7.1 TXA, topical vs TXA, oral + TXA, topical 4 g	1	120	Risk Ratio (M‐H, Random, 95% CI)	Not estimable
26.7.2 TXA, topical vs TXA, oral + TXA, topical 5 g	1	120	Risk Ratio (M‐H, Random, 95% CI)	Not estimable
26.7.3 TXA, topical vs TXA, oral + TXA, topical 6 g	1	120	Risk Ratio (M‐H, Random, 95% CI)	Not estimable
26.7.4 TXA, topical vs TXA, oral + TXA, topical 7 g	1	120	Risk Ratio (M‐H, Random, 95% CI)	Not estimable

**Comparison 27:** TXA oral vs TXA combined topical + IV + oral

Outcome or subgroup title	No. of studies	No. of participants	Statistical method	Effect size
27.1 Risk of allogeneic blood transfusion	1		Peto Odds Ratio (Peto, Fixed, 95% CI)	Subtotals only
27.2 Length of hospital stay	1		Mean Difference (IV, Random, 95% CI)	Totals not selected
27.3 Risk of experiencing DVT	1		Risk Ratio (M‐H, Random, 95% CI)	Subtotals only

**Comparison 28:** TXA IV + topical lower dose vs TXA IV + topical higher dose

Outcome or subgroup title	No. of studies	No. of participants	Statistical method	Effect size
28.1 Risk of allogeneic blood transfusion	1		Peto Odds Ratio (Peto, Fixed, 95% CI)	Subtotals only
28.1.1 TXA, IV + TXA, IA 3 g vs TXA, IV + TXA, IA more than 3 g	1	100	Peto Odds Ratio (Peto, Fixed, 95% CI)	7.39 [0.15, 372.38]
28.1.2 TXA, IV + TXA, IA 3 g vs TXA, IV + TXA, IA more than 4 g	1	100	Peto Odds Ratio (Peto, Fixed, 95% CI)	7.39 [0.15, 372.38]
28.1.3 TXA, IV + TXA, IA more than 3 g vs TXA, IV + TXA, IA more than 4 g	1	100	Peto Odds Ratio (Peto, Fixed, 95% CI)	Not estimable
28.2 Length of hospital stay	1		Mean Difference (IV, Random, 95% CI)	Subtotals only
28.2.1 TXA, IV + TXA, IA 3 g vs TXA, IV + TXA, IA more than 3 g	1	100	Mean Difference (IV, Random, 95% CI)	0.54 [‐0.17, 1.25]
28.2.2 TXA, IV + TXA, IA 3 g vs TXA, IV + TXA, IA more than 4 g	1	100	Mean Difference (IV, Random, 95% CI)	0.91 [0.27, 1.55]
28.2.3 TXA, IV + TXA, IA more than 3 g vs TXA, IV + TXA, IA more than 4 g	1	100	Mean Difference (IV, Random, 95% CI)	0.37 [‐0.26, 1.00]
28.3 Risk of experiencing DVT	1		Risk Ratio (M‐H, Random, 95% CI)	Subtotals only
28.3.1 TXA, IV + TXA, IA 3 g vs TXA, IV + TXA, IA more than 3 g	1	100	Risk Ratio (M‐H, Random, 95% CI)	Not estimable
28.3.2 TXA, IV + TXA, IA 3 g vs TXA, IV + TXA, IA more than 4 g	1	100	Risk Ratio (M‐H, Random, 95% CI)	Not estimable
28.3.3 TXA, IV + TXA, IA more than 3 g vs TXA, IV + TXA, IA more than 4 g	1	100	Risk Ratio (M‐H, Random, 95% CI)	Not estimable
28.4 Risk of experiencing PE	1		Risk Ratio (M‐H, Random, 95% CI)	Subtotals only
28.4.1 TXA, IV + TXA, IA 3 g vs TXA, IV + TXA, IA more than 3 g	1	100	Risk Ratio (M‐H, Random, 95% CI)	Not estimable
28.4.2 TXA, IV + TXA, IA 3 g vs TXA, IV + TXA, IA more than 4 g	1	100	Risk Ratio (M‐H, Random, 95% CI)	Not estimable
28.4.3 TXA, IV + TXA, IA more than 3 g vs TXA, IV + TXA, IA more than 4 g	1	100	Risk Ratio (M‐H, Random, 95% CI)	Not estimable
28.5 Risk of experiencing MI	1		Risk Ratio (M‐H, Random, 95% CI)	Subtotals only
28.5.1 TXA, IV + TXA, IA 3 g vs TXA, IV + TXA, IA more than 3 g	1	100	Risk Ratio (M‐H, Random, 95% CI)	Not estimable
28.5.2 TXA, IV + TXA, IA 3g vs TXA, IV + TXA, IA More than 4g	1	100	Risk Ratio (M‐H, Random, 95% CI)	Not estimable
28.5.3 TXA, IV + TXA, IA More than 3g vs TXA, IV + TXA, IA More than 4g	1	100	Risk Ratio (M‐H, Random, 95% CI)	Not estimable
28.6 Risk of experiencing CVA	1		Risk Ratio (M‐H, Random, 95% CI)	Subtotals only
28.6.1 TXA, IV + TXA, IA 3 g vs TXA, IV + TXA, IA more than 3 g	1	100	Risk Ratio (M‐H, Random, 95% CI)	Not estimable
28.6.2 TXA, IV + TXA, IA 3 g vs TXA, IV + TXA, IA more than 4 g	1	100	Risk Ratio (M‐H, Random, 95% CI)	Not estimable
28.6.3 TXA, IV + TXA, IA more than 3 g vs TXA, IV + TXA, IA more than 4 g	1	100	Risk Ratio (M‐H, Random, 95% CI)	Not estimable

**Comparison 29:** TXA oral + topical lower dose vs TXA oral + topical higher dose

Outcome or subgroup title	No. of studies	No. of participants	Statistical method	Effect size
29.1 Risk of allogeneic blood transfusion	2		Peto Odds Ratio (Peto, Fixed, 95% CI)	Subtotals only
29.1.1 TXA, oral + TXA, topical 4 g vs TXA, oral + TXA, topical 5 g	2	220	Peto Odds Ratio (Peto, Fixed, 95% CI)	1.96 [0.20, 18.92]
29.1.2 TXA, oral + TXA, topical 4 g vs TXA, oral + TXA, topical 6 g	2	220	Peto Odds Ratio (Peto, Fixed, 95% CI)	7.39 [0.46, 118.13]
29.1.3 TXA, oral + TXA, topical 4 g vs TXA, oral + TXA, topical 7 g	2	220	Peto Odds Ratio (Peto, Fixed, 95% CI)	7.39 [0.46, 118.13]
29.1.4 TXA, oral + TXA, topical 5 g vs TXA, oral + TXA, topical 6 g	2	220	Peto Odds Ratio (Peto, Fixed, 95% CI)	7.39 [0.15, 372.38]
29.1.5 TXA, oral + TXA, topical 5 g vs TXA, oral + TXA, topical 7 g	2	220	Peto Odds Ratio (Peto, Fixed, 95% CI)	7.39 [0.15, 372.38]
29.1.6 TXA, oral + TXA, topical 6 g vs TXA, oral + TXA, topical 7 g	2	220	Peto Odds Ratio (Peto, Fixed, 95% CI)	Not estimable
29.2 All‐cause mortality	1		Risk Ratio (M‐H, Random, 95% CI)	Subtotals only
29.2.1 TXA, oral + TXA, topical 4 g vs TXA, oral + TXA, topical 5 g	1	120	Risk Ratio (M‐H, Random, 95% CI)	Not estimable
29.2.2 TXA, oral + TXA, topical 4 g vs TXA, oral + TXA, topical 6 g	1	120	Risk Ratio (M‐H, Random, 95% CI)	Not estimable
29.2.3 TXA, oral + TXA, topical 4 g vs TXA, oral + TXA, topical 7 g	1	120	Risk Ratio (M‐H, Random, 95% CI)	Not estimable
29.2.4 TXA, oral + TXA, topical 5 g vs TXA, oral + TXA, topical 6 g	1	120	Risk Ratio (M‐H, Random, 95% CI)	Not estimable
29.2.5 TXA, oral + TXA, topical 5 g vs TXA, oral + TXA, topical 7 g	1	120	Risk Ratio (M‐H, Random, 95% CI)	Not estimable
29.2.6 TXA, oral + TXA, topical 6 g vs TXA, oral + TXA, topical 7 g	1	120	Risk Ratio (M‐H, Random, 95% CI)	Not estimable
29.3 Length of hospital stay	2		Mean Difference (IV, Random, 95% CI)	Subtotals only
29.3.1 TXA, oral + TXA, tpical 4 g vs TXA, oral + TXA, topical 5 g	2	220	Mean Difference (IV, Random, 95% CI)	0.03 [‐0.28, 0.34]
29.3.2 TXA, oral + TXA, topical 4 g vs TXA, oral + TXA, topical 6 g	2	220	Mean Difference (IV, Random, 95% CI)	‐0.10 [‐0.69, 0.49]
29.3.3 TXA, oral + TXA, topical 4 g vs TXA, oral + TXA, topical 7 g	2	220	Mean Difference (IV, Random, 95% CI)	‐0.02 [‐0.45, 0.41]
29.3.4 TXA, oral + TXA, topical 5 g vs TXA, oral + TXA, topical 6 g	2	220	Mean Difference (IV, Random, 95% CI)	‐0.15 [‐0.43, 0.12]
29.3.5 TXA, oral + TXA, topical 5 g vs TXA, oral + TXA, topical 7 g	2	220	Mean Difference (IV, Random, 95% CI)	‐0.06 [‐0.32, 0.20]
29.3.6 TXA, oral + TXA, topical 6 g vs TXA, oral + TXA, topical 7 g	2	220	Mean Difference (IV, Random, 95% CI)	0.08 [‐0.16, 0.32]
29.4 Risk of experiencing DVT	2		Peto Odds Ratio (Peto, Fixed, 95% CI)	Subtotals only
29.4.1 TXA, oral + TXA, topical 4 g vs TXA, oral, + TXA, topical 5 g	2	220	Peto Odds Ratio (Peto, Fixed, 95% CI)	1.97 [0.20, 19.31]
29.4.2 TXA, oral + TXA, topical 4 g vs TXA, oral + TXA, topical 6 g	2	220	Peto Odds Ratio (Peto, Fixed, 95% CI)	1.00 [0.14, 7.28]
29.4.3 TXA, oral + TXA, topical 4 g vs TXA, oral + TXA, topical 7 g	2	220	Peto Odds Ratio (Peto, Fixed, 95% CI)	1.00 [0.14, 7.28]
29.4.4 TXA, oral + TXA, topical 5 g vs TXA, oral + TXA, topical 6 g	2	220	Peto Odds Ratio (Peto, Fixed, 95% CI)	0.51 [0.05, 4.97]
29.4.5 TXA, oral + TXA, topical 5 g vs TXA, oral + TXA, topical 7 g	2	220	Peto Odds Ratio (Peto, Fixed, 95% CI)	0.51 [0.05, 4.97]
29.4.6 TXA, oral + TXA, topical 6 g vs TXA, oral + TXA, topical 7 g	2	220	Peto Odds Ratio (Peto, Fixed, 95% CI)	1.00 [0.14, 7.28]
29.5 Risk of experiencing PE	2		Risk Ratio (M‐H, Random, 95% CI)	Subtotals only
29.5.1 TXA, oral + TXA, topical 4 g vs TXA, oral + TXA, topical 5 g	2	220	Risk Ratio (M‐H, Random, 95% CI)	Not estimable
29.5.2 TXA, oral + TXA, topical 4 g vs TXA, oral + TXA, topical 6 g	2	220	Risk Ratio (M‐H, Random, 95% CI)	Not estimable
29.5.3 TXA, oral + TXA, topical 4 g vs TXA, oral + TXA, topical 7 g	2	220	Risk Ratio (M‐H, Random, 95% CI)	Not estimable
29.5.4 TXA, oral + TXA, topical 5 g vs TXA, oral + TXA, topical 6 g	2	220	Risk Ratio (M‐H, Random, 95% CI)	Not estimable
29.5.5 TXA, oral + TXA, topical 5 g vs TXA, oral + TXA, topical 7 g	2	220	Risk Ratio (M‐H, Random, 95% CI)	Not estimable
29.5.6 TXA, oral + TXA, topical 6 g vs TXA, oral + TXA, topical 7 g	2	220	Risk Ratio (M‐H, Random, 95% CI)	Not estimable
29.6 Risk of experiencing MI	2		Risk Ratio (M‐H, Random, 95% CI)	Subtotals only
29.6.1 TXA, oral + TXA, topical 4 g vs TXA, oral + TXA, topical 5 g	2	220	Risk Ratio (M‐H, Random, 95% CI)	Not estimable
29.6.2 TXA, oral + TXA, topical 4 g vs TXA, oral + TXA, topical 6 g	2	220	Risk Ratio (M‐H, Random, 95% CI)	Not estimable
29.6.3 TXA, oral + TXA, topical 4 g vs TXA, oral + TXA, topical 7 g	2	220	Risk Ratio (M‐H, Random, 95% CI)	Not estimable
29.6.4 TXA, oral + TXA, topical 5 g vs TXA, oral + TXA, topical 6 g	2	220	Risk Ratio (M‐H, Random, 95% CI)	Not estimable
29.6.5 TXA, oral + TXA, topical 5 g vs TXA, oral + TXA, topical 7 g	2	220	Risk Ratio (M‐H, Random, 95% CI)	Not estimable
29.6.6 TXA, oral + TXA, topical 6 g vs TXA, oral + TXA, topical 7 g	2	220	Risk Ratio (M‐H, Random, 95% CI)	Not estimable
29.7 Risk of experiencing CVA	2		Risk Ratio (M‐H, Random, 95% CI)	Subtotals only
29.7.1 TXA, oral + TXA, topical 4 g vs TXA, oral + TXA, topical 5 g	2	220	Risk Ratio (M‐H, Random, 95% CI)	Not estimable
29.7.2 TXA, oral + TXA, topical 4 g vs TXA, oral + TXA, topical 6 g	2	220	Risk Ratio (M‐H, Random, 95% CI)	Not estimable
29.7.3 TXA, oral + TXA, topical 4 g vs TXA, oral + TXA, topical 7g	2	220	Risk Ratio (M‐H, Random, 95% CI)	Not estimable
29.7.4 TXA, oral + TXA, topical 5 g vs TXA, oral + TXA, topical 6 g	2	220	Risk Ratio (M‐H, Random, 95% CI)	Not estimable
29.7.5 TXA, oral + TXA, topical 5 g vs TXA, oral + TXA, topical 7 g	2	220	Risk Ratio (M‐H, Random, 95% CI)	Not estimable
29.7.6 TXA, oral + TXA, topical 6 g vs TXA, oral + TXA, topical 7 g	2	220	Risk Ratio (M‐H, Random, 95% CI)	Not estimable

**Comparison 30:** TXA IV + TXA, oral days 1 to 2 vs TXA IV + TXA, oral days 1 to 5

Outcome or subgroup title	No. of studies	No. of participants	Statistical method	Effect size
30.1 Risk of allogeneic blood transfusion	1		Peto Odds Ratio (Peto, Fixed, 95% CI)	Subtotals only
30.2 Risk of experiencing DVT	1		Peto Odds Ratio (Peto, Fixed, 95% CI)	Subtotals only
30.3 Risk of experiencing PE	1		Risk Ratio (M‐H, Random, 95% CI)	Subtotals only

**Comparison 31:** TXA, IV + TXA, IV vs TXA, IV + placebo

Outcome or subgroup title	No. of studies	No. of participants	Statistical method	Effect size
31.1 Risk of allogeneic blood transfusion	1		Risk Ratio (M‐H, Random, 95% CI)	Subtotals only
31.2 Risk of experiencing MI	1		Risk Ratio (M‐H, Random, 95% CI)	Subtotals only
31.3 Risk of experiencing CVA	1		Risk Ratio (M‐H, Random, 95% CI)	Subtotals only
31.4 Risk of experiencing DVT	1		Risk Ratio (M‐H, Random, 95% CI)	Subtotals only
31.5 Risk of experiencing PE	1		Risk Ratio (M‐H, Random, 95% CI)	Subtotals only

**Comparison 32:** TXA, IV + TXA, IV vs placebo

Outcome or subgroup title	No. of studies	No. of participants	Statistical method	Effect size
32.1 Risk of allogeneic blood transfusion	1		Risk Ratio (M‐H, Random, 95% CI)	Subtotals only
32.2 Risk of experiencing MI	1		Risk Ratio (M‐H, Random, 95% CI)	Subtotals only
32.3 Risk of experiencing CVA	1		Risk Ratio (M‐H, Random, 95% CI)	Subtotals only
32.4 Risk of experiencing DVT	1		Risk Ratio (M‐H, Random, 95% CI)	Subtotals only
32.5 Risk of experiencing PE	1		Risk Ratio (M‐H, Random, 95% CI)	Subtotals only

**Comparison 33:** TXA IV + placebo vs placebo

Outcome or subgroup title	No. of studies	No. of participants	Statistical method	Effect size
33.1 Risk of allogeneic blood transfusion	1		Risk Ratio (M‐H, Random, 95% CI)	Subtotals only
33.2 Risk of experiencing MI	1		Risk Ratio (M‐H, Random, 95% CI)	Subtotals only
33.3 Risk of experiencing CVA	1		Risk Ratio (M‐H, Random, 95% CI)	Subtotals only
33.4 Risk of experiencing DVT	1		Risk Ratio (M‐H, Random, 95% CI)	Subtotals only
33.5 Risk of experiencing PE	1		Risk Ratio (M‐H, Random, 95% CI)	Subtotals only

## Characteristics of studies

### Characteristics of included studies [ordered by study ID]

**Table:** Alvarez 2008

***Study characteristics***		
Methods	**Study design:** single‐centre, 2‐arm, RCT **Intention‐to‐treat analysis**: yes **Duration of study:** 13 months **Power calculation reached:** no **Transfusion strategy:** not reported **Was the trial stopped early:** no	
Participants	**Baseline characteristics** Placebo arm *Age (years) (mean SD)*: 72 (7) *Ethnicity*: not reported *Gender (males, females)*: 10/49 M (20%), 39/49 F (80%) *Length of surgery (minutes) (mean SD)*: not reported *Proportion of participants on anticoagulants prior to surgery (n/N, %)*: 0/55, 0% *Incidence of preoperative anaemia (n/N, %):* not reported *Co‐morbidities (n/N, %)*: not reported *ASA 1 (n/N, %)*: 3/49, 6.12% *ASA 2 (n/N, %)*: 39/49, 79.59% *ASA 3 (n/N, %)*: 7/49, 14.29% *ASA 4 (n/N, %)*: not reported *Number of participants randomised*: 55 *Number of participants receiving treatment*: 50 *Number of participants analysed*: 49 *Dropout rate*: 6/55 (10.91%) TXA arm *Age (years) (mean SD)*: 71 (9) *Ethnicity*: not reported *Gender (males, females)*: 7/46 M (15%), 39/46 F (85%) *Length of surgery (minutes) (mean SD)*: not reported *Proportion of participants on anticoagulants prior to surgery (n/N, %)*: 0/55, 0% *Incidence of preoperative anaemia (n/N, %)*: not reported *Co‐morbidities (n/N, %)*: not reported *ASA 1 (n/N, %)*: 1/46, 2.17% *ASA 2 (n/N, %)*: 39/46, 84.78% *ASA 3 (n/N, %)*: 5/46, 10.87% *ASA 4 (n/N, %)*: not reported *Number of participants randomised*: 55 *Number of participants receiving treatment*: 46 *Number of participants analysed*: 46 *Drop out rate*: 9/55 (16.36%) Overall *Age (years) (mean SD)*: not reported *Ethnicity*: not reported *Gender (male, female)*: not reported *Length of surgery (minutes) (mean SD)*: not reported *Proportion of participants on anticoagulants prior to surgery (n/N, %)*: not reported *Incidence of preoperative anaemia (n/N, %)*: not reported *Co‐morbidities (n/N, %)*: not reported *ASA 1 (n/N, %)*: not reported *ASA 2 (n/N, %)*: not reported *ASA 3 (n/N, %)*: not reported *ASA 4 (n/N, %)*: not reported *Number of participants randomised*: 110 *Number of participants receiving treatment*: 96 *Number of participants analysed*: 95 *Drop out rate (%)*: 15/110 (13.6%) **Inclusion criteria:** 1) patients scheduled for total knee replacement surgery, 2) diagnosed with osteoarthrosis **Exclusion criteria:** 1) known allergy to tranexamic acid, 2) ASA‐IV physical status or higher, 3) severe ischaemia and/or heart valve disease, 4) history of thromboembolic episodes, 5) known coagulopathy, 6) renal dysfunction (serum creatinine concentration > 1.5 mg/dL) **If TKR, is tourniquet used:** yes **Indication for surgery:** osteoarthrosis **Type of anaesthetic:** spinal **Type of surgery:** primary TKR	
Interventions	**Intervention characteristics** Placebo arm Placebo bolus (physiologic saline), administered by the research anaesthetist 30 minutes before deflation of the tourniquet followed by an infusion of 1 mg per kg per hour starting at the end of the operation and continuing during the first 6 postoperative hour Placebo, IV, 10 mg/kg, preop + IV, 1 mg/kg/hr, postop (repeated dose) TXA arm TXA bolus of 10 mg per kg tranexamic acid, administered by the research anaesthetist 30 minutes before deflation of the tourniquet followed by an infusion of 1 mg per kg per hour starting at the end of the operation and continuing during the first 6 postoperative hours TXA, IV, 10 mg/kg, intraop (30 mins before tourniquet deflation) + 1 mg/kg/hr postop infusion for 6 hours	
Outcomes	*Primary outcome* Transfusion rate *Secondary outcomes* Postoperative blood loss	
Notes	**Sponsorship source:** not reported **Country**: Spain **Setting**: not reported **Comments**: none **Author's name:** J.C. Alvarez **Institution:** University Hospital of Mar‐Esperança **Email:** 94188@imas.imim.es **Address:** Department of Anesthesiology, University Hospital of Mar‐Esperança, Passeig Marítim 25, 08003 Barcelona, Spain **Native language of paper:** English **Reference type:** full text (1) **Trial registration number:** not applicable, but when checked, trial registration number not found **Was it translated for this review:** no	
***Risk of bias***		
**Bias**	**Authors' judgement**	**Support for judgement**
Random sequence generation (selection bias)	Low risk	Quote: "Patients were assigned to tranexamic acid group or control group with software that provided a series of random numbers." Judgement comment: cmputer‐generated randomisation
Allocation concealment (selection bias)	Unclear risk	Quote: "The randomized assignment was sealed in a numbered envelope." Judgement comment: the envelopes were not opaque or sequential.
Blinding of participants and personnel (performance bias) Subjective outcomes	High risk	Quote: "An independent anesthetist who was not otherwise engaged in the study opened the sealed randomization envelope and was responsible for preparing the medication, a bolus of 10 mg per kg tranexamic acid" Judgement comment: key personnel were not blinded, which may introduce bias. The research anesthetist administered the intervention.
Blinding of participants and personnel (performance bias) Objective outcomes	Low risk	Quote: Not applicable Judgement comment: objective outcome for personnel, so low risk of bias regardless of quality of blinding. Objective outcome for personnel, so low risk of bias regardless of quality of blinding.
Blinding of outcome assessment (detection bias) Subjective outcomes	Low risk	Quote: "An independent anesthetist who was not otherwise engaged in the study opened the sealed randomization envelope and was responsible for preparing the medication, a bolus of 10 mg per kg tranexamic acid" Judgement comment: key personnel were not blinded, which may introduce bias. The research anaesthetist administered the intervention.
Blinding of outcome assessment (detection bias) Objective outcomes	Low risk	Quote: Not applicable Judgement comment: objective outcome for assessors, so low risk of bias regardless of quality of blinding
Incomplete outcome data (attrition bias) All outcomes	Low risk	Quote: Not applicable Judgement comment: reason for missing outcome data unlikely to affect outcome.
Selective reporting (reporting bias)	Unclear risk	Quote: not applicable Judgement comment: no protocol or trial registration available to know pre‐specified outcomes.
Other bias	Unclear risk	Quote: not applicable Judgement comment: sponsorship and funding source not declared.

**Table:** Alvarez 2019 hip

***Study characteristics***		
Methods	**Study design:** RCT **Intention‐to‐treat analysis:** yes **Duration of study:** recruitment period not stated, but follow‐up was 1 yr **Power calculation reached:** yes **Transfusion strategy:** if haemoglobin (Hb) levels fell to below 8 g/dL or the patient showed signs of anaemia, the protocol permitted the transfusion of red blood cells **Was the trial stopped early:** no	
Participants	**Baseline characteristics** Placebo, IV *Age (years) (mean SD)*: 70 (12.4) *Ethnicity*: not reported *Gender (male, female)*: 4/11 M (36.4%); 7/11 F (63.6%) L*ength of surgery (minutes) (mean SD)*: 59 (30) *Proportion of participants on anticoagulants prior to surgery (n/N, %)*: not reported *Incidence of preoperative anaemia (n/N, %)*: not reported *Co‐morbidities (n/N, %)*: BMI 30.2 (6.2) *ASA 1 (n/N, %)*: not reported *ASA 2 (n/N, %)*: 8/11, 72.7% *ASA 3 (n/N, %)*: 3/11, 27.3% *ASA 4 (n/N, %)*: 0/11, 0% *Number of participants randomised*: 11 *Number of participants receiving treatment*: 11 *Number of participants analysed*: 11 *Dropout rate (%)*: 0/11, 0% TXA, IV *Age (years) (mean SD)*: 68.4 (9.4) *Ethnicity*: not reported *Gender (male, female)*: 3/11 M (27.3%); 8/11 F (72.7%) *Length of surgery (minutes) (mean SD)*: 71 (30) *Proportion of participants on anticoagulants prior to surgery (n/N, %)*: not reported *Incidence of preoperative anaemia (n/N, %)*: not reported *Co‐morbidities (n/N, %)*: not reported *ASA 1 (n/N, %)*: not reported *ASA 2 (n/N, %)*: 8/11, 72.7% *ASA 3 (n/N, %)*: 3/11, 27.3% *ASA 4 (n/N, %)*: 0/11, 0% *Number of participants randomised*: 11 *Number of participants receiving treatment*: 11 *Number of participants analysed*: 11 *Dropout rate (%)*: 0/11, 0% Overall *Age (years) (mean SD)*: not reported *Ethnicity*: not reported *Gender (male, female)*: not reported *Length of surgery (minutes) (mean SD)*: not reported *Proportion of participants on anticoagulants prior to surgery (n/N, %)*: not reported *Incidence of preoperative anaemia (n/N, %)*: not reported *Co‐morbidities (n/N, %)*: not reported *ASA 1 (n/N, %)*: not reported *ASA 2 (n/N, %)*: not reported *ASA 3 (n/N, %)*: not reported *ASA 4 (n/N, %)*: not reported *Number of participants randomised*: 22 *Number of participants receiving treatment*: 22 *Number of participants analysed*: 22 *Dropout rate (%)*: 0/22, 0% **Inclusion criteria:** patients scheduled for TKA and THA were considered eligible for the study **Exclusion criteria:** exclusion criteria were: allergy to TXA or radiological contrast media, history of thromboembolism (to avoid exposing patients with previously undetected thrombophilia to TXA), coagulopathy and kidney failure with glomerular filtration < 60 mL/min/m^2^ **If TKR, is tourniquet used:** tourniquet was used for the TKA group in this study (see Alvarez 2019 knee for details) **Indication for surgery:** not reported **Type of anaesthetic:** the anaesthesia protocol prioritised the use of spinal anaesthesia, but general anaesthesia was also permitted **Type of surgery:** primary TKA	
Interventions	**Intervention characteristics** Placebo, IV An independent anaesthetist prepared 2 vials containing 10 mg/kg TXA (Amchafibrin, Agenzia‐Ecopharma Laboratories) or placebo (saline solution). Following the double‐blind protocol, the researcher administered the medication in 2 infusions: 30 min before and 3 h after the incision. Placebo, IV, 10 mg/kg, pre‐op + 10 mg/kg, postop TXA, IV An independent anaesthetist prepared 2 vials containing 10 mg/kg TXA (Amchafibrin, Agenzia‐Ecopharma Laboratories) or placebo (saline solution). Following the double‐blind protocol, the researcher administered the medication in 2 infusions: 30 min before and 3 h after the incision. TXA, IV, 10 mg/kg, pre‐op + 10 mg/kg, postop	
Outcomes	*Primary outcome:* Percentage of patients requiring transfusion *Secondary outcomes:* Postoperative bleeding Incidence of DVT and PE Clotting and fibrinolysis parameters	
Notes	**Sponsorship source:** non‐pharmaceutical (this study was funded by an FIS grant from the Carlos III Institute, Madrid (FIS 2006/1227)) **Country:** Spain **Setting:** single‐centre **Comments**: 1) This paper gives data for both knees and hips, which we have extracted separately: Alvarez 2019 hip for the THA group and Alvarez 2019 knee for the TKA group; 2. Red blood cells divided by TXA/placebo not by operation as well. **Author's name:** J. Alvarez **Institution:** Institut Hospital del Mar d’Investigacions Mèdiques (IMIM), Universitat Autònoma de Barcelona (UAB) **Email:** J. Alvarez: 94188@hospitaldelmar.cat **Address:** Serviciode Anestesia Reanimación y Terapia del Dolor, Parc de Salut MAR, Institut Hospital del Mar d’Investigacions Mèdiques (IMIM), Universitat Autònoma de Barcelona (UAB), Barcelona, Spain **Native language of paper:** English **Reference type:** full text (1) **Trial registration number:** EudraCT2007‐000193‐22 **Was it translated for this review:** no	
***Risk of bias***		
**Bias**	**Authors' judgement**	**Support for judgement**
Random sequence generation (selection bias)	Low risk	Quote: "After signing the informed consent form, patients were assigned a sequential identification number. Using randomisation software, each number was randomised to receive TXA or placebo" Judgement comment: adequate method of sequence generation with computer‐generated code.
Allocation concealment (selection bias)	Unclear risk	Quote: "each number was randomised to receive TXA or placebo, and placed in a sealed envelope." Judgement comment: unclear if envelopes were opaque.
Blinding of participants and personnel (performance bias) Subjective outcomes	Low risk	Quote: "independent anaesthetist prepared 2 vials containing 10 mg/kg TXA (Amchafibrin, Agenzia‐Ecopharma Laboratories) or placebo (saline solution)." Judgement comment: not applicable.
Blinding of participants and personnel (performance bias) Objective outcomes	Low risk	Quote: not applicable Judgement comment: objective outcome for personnel, so low risk of bias regardless of quality of blinding.
Blinding of outcome assessment (detection bias) Subjective outcomes	Low risk	Quote: not applicable Judgement comment: independent anaesthetist prepared the vials
Blinding of outcome assessment (detection bias) Objective outcomes	Low risk	Quote: not applicable Judgement comment: objective outcome for personnel, so low risk of bias regardless of quality of blinding.
Incomplete outcome data (attrition bias) All outcomes	Low risk	Quote: not applicable Judgement comment: no obvious outcome data missing
Selective reporting (reporting bias)	Low risk	Quote: not applicable Judgement comment: all outcomes planned in the protocol or prospective trial registration are reported.
Other bias	Low risk	Quote: not applicable Judgement comment: no other concerns such as early stopping or imbalanced study arms.

**Table:** Alvarez 2019 knee

***Study characteristics***		
Methods	**Study design:** RCT **Intention‐to‐treat analysis**: yes **Duration of study:** recruitment period not stated, but follow‐up was 1 yr **Power calculation reached:** yes **Transfusion strategy:** if haemoglobin (Hb) levels fell to below 8 g/dL or the patient showed signs of anaemia, the protocol permitted the transfusion of red blood cells **Was the trial stopped early:** no	
Participants	**Baseline characteristics** Placebo, IV *Age (years) (mean SD)*: 72.4 (7.1) *Ethnicity*: not reported *Gender (male, female)*: 2/11 M (18.2%); 9/11 F (81.8%) *Length of surgery (minutes) (mean SD)*: 84 (14) *Proportion of participants on anticoagulants prior to surgery (n/N, %)*: not reported *Incidence of preoperative anaemia (n/N, %)*: not reported *Co‐morbidities (n/N, %)*: BMI: 34.6 (8.9) *ASA 1 (n/N, %)*: not reported *ASA 2 (n/N, %)*: 5/11, 45.5% *ASA 3 (n/N, %)*: 6/11, 54.5% *ASA 4 (n/N, %)*: 0/11, 0% *Number of participants randomised*: 11 *Number of participants receiving treatment*: 11 *Number of participants analysed*: 11 *Dropout rate (%)*: 0/11, 0% TXA, IV *Age (years) (mean SD)*: 70 (7.5) *Ethnicity*: not reported *Gender (male, female)*: 3/11 M (27.3%); 8/11 F (72.7%) *Length of surgery (minutes) (mean SD)*: 85 (22) *Proportion of participants on anticoagulants prior to surgery (n/N, %)*: not reported *Incidence of preoperative anaemia (n/N, %)*: not reported *Co‐morbidities (n/N, %)*: BMI 34.1 (7) *ASA 1 (n/N, %)*: not reported *ASA 2 (n/N, %)*: 6/11, 54.5% *ASA 3 (n/N, %)*: 4/11, 36.4% *ASA 4 (n/N, %)*: 1/11, 9.1% *Number of participants randomised*: 11 *Number of participants receiving treatment*: 11 *Number of participants analysed*: 11 *Dropout rate (%)*: 0/11, 0% Overall *Age (years) (mean SD)*: not reported *Ethnicity*: not reported *Gender (male, female)*: not reported *Length of surgery (minutes) (mean SD)*: not reported *Proportion of participants on anticoagulants prior to surgery (n/N, %)*: not reported *Incidence of preoperative anaemia (n/N, %)*: not reported *Co‐morbidities (n/N, %)*: not reported *ASA 1 (n/N, %)*: not reported *ASA 2 (n/N, %)*: not reported *ASA 3 (n/N, %)*: not reported *ASA 4 (n/N, %)*: not reported *Number of participants randomised*: 22 *Number of participants receiving treatment*: 22 *Number of participants analysed*: 22 *Dropout rate (%)*: 0/22, 0% **Inclusion criteria:** patients scheduled for TKA and THA were considered eligible for the study **Exclusion criteria:** exclusion criteria were: allergy to TXA or radiological contrast media, history of thromboembolism (to avoid exposing patients with previously undetected thrombophilia to TXA), coagulopathy and kidney failure with glomerular filtration < 60 mL/min/m^2^ **If TKR, is tourniquet used: y**es (tourniquet was inflated to 350 mmHg during surgery) **Indication for surgery:** not reported **Type of anaesthetic:** the anaesthesia protocol prioritised the use of spinal anaesthesia, but general anaesthesia was also permitted **Type of surgery:** primary TKA	
Interventions	**Intervention characteristics** Placebo, IV An independent anaesthetist prepared 2 vials containing 10 mg/kg TXA (Amchafibrin, Agenzia‐Ecopharma Laboratories) or placebo (saline solution). Following the double‐blind protocol, the researcher administered the medication in 2 infusions: 30 min before and 3 h after the incision. Placebo, IV, 10 mg/kg, pre‐op + 10 mg/kg, postop TXA, IV An independent anaesthetist prepared 2 vials containing 10 mg/kg TXA (Amchafibrin, Agenzia‐Ecopharma Laboratories) or placebo (saline solution). Following the double‐blind protocol, the researcher administered the medication in 2 infusions: 30 min before and 3 h after the incision. TXA, IV, 10 mg/kg, pre‐op + 10 mg/kg, postop	
Outcomes	*Primary outcome:* Percentage of patients requiring transfusion *Secondary outcomes:* Postoperative bleeding Incidence of DVT and PE Clotting and fibrinolysis parameters	
Notes	**Sponsorship source:** non‐pharmaceutical (this study was funded by an FIS grant from the Carlos III Institute, Madrid (FIS 2006/1227)) **Country:** Spain **Setting:** single‐centre **Comments**: 1) This paper gives data for both knees and hips, which we have extracted separately: Alvarez 2019 hip for the THA group and Alvarez 2019 knee for the TKA group; 2) Red blood cells divided by TXA/placebo not by operation as well. **Author's name:** J. Alvarez **Institution:** Institut Hospital del Mar d’Investigacions Mèdiques (IMIM), Universitat Autònoma de Barcelona (UAB) **Email:** J. Alvarez: 94188@hospitaldelmar.cat **Address:** Serviciode Anestesia Reanimación y Terapia del Dolor, Parc de Salut MAR, Institut Hospital del Mar d’Investigacions Mèdiques (IMIM), Universitat Autònoma de Barcelona (UAB), Barcelona, Spain **Native language of paper:** English **Reference type:** full text (1) **Trial registration number:** EudraCT2007‐000193‐22 **Was it translated for this review:** no	
***Risk of bias***		
**Bias**	**Authors' judgement**	**Support for judgement**
Random sequence generation (selection bias)	Low risk	Quote: "After signing the informed consent form, patients were assigned a sequential identification number. Using randomisation software, each number was randomised to receive TXA or placebo" Judgement comment: adequate method of sequence generation with computer‐generated code.
Allocation concealment (selection bias)	Unclear risk	Quote: "each number was randomised to receive TXA or placebo, and placed in a sealed envelope." Judgement comment: unclear if envelopes were opaque.
Blinding of participants and personnel (performance bias) Subjective outcomes	Low risk	Quote: "independent anaesthetist prepared 2 vials containing 10 mg/kg TXA (Amchafibrin, Agenzia‐Ecopharma Laboratories) or placebo (saline solution)." Judgement comment: none
Blinding of participants and personnel (performance bias) Objective outcomes	Low risk	Quote: not applicable Judgement comment: objective outcome for personnel, so low risk of bias regardless of quality of blinding.
Blinding of outcome assessment (detection bias) Subjective outcomes	Low risk	Quote: not applicable Judgement comment: independent anaesthetist prepared the vials.
Blinding of outcome assessment (detection bias) Objective outcomes	Low risk	Quote: not applicable Judgement comment: objective outcome for personnel, so low risk of bias regardless of quality of blinding.
Incomplete outcome data (attrition bias) All outcomes	Low risk	Quote: none Judgement comment: no obvious outcome data missing.
Selective reporting (reporting bias)	Low risk	Quote: not applicable Judgement comment: all outcomes planned in the protocol or prospective trial registration are reported.
Other bias	Low risk	Quote: not applicable Judgement comment: no other concerns such as early stopping or imbalanced study arms.

**Table:** Benoni 1996

***Study characteristics***		
Methods	**Study design:** 2‐arm, RCT **Intention‐to‐treat analysis:** no **Duration of study:** not reported **Power calculation reached:** no **Transfusion strategy:** no specific value for Hb concentration or haematocrit levels; each transfusion prescribed with regard to the patient’s cardiovascular history, present status, fall in Hb level, rate of blood loss and age. As a rule of thumb, they considered blood transfusion indicated at a Hb concentration of below 85 to 100 g/L. **Was the trial stopped early:** no	
Participants	**Baseline characteristics** Placebo *Age (years) (mean SD)*: 74 (7) *Ethnicity*: not reported *Gender (males, females)*: 10/43 M (23%); 33/43 F (77%) *Length of surgery (minutes) (mean SD)*: 96 (18) *Proportion of participants on anticoagulants prior to surgery (n/N, %)*: not reported *Incidence of preoperative anaemia (n/N, %)*: not reported *Co‐morbidities (n/N, %)*: not reported *ASA 1 (n/N, %)*: not reported *ASA 2 (n/N, %)*: not reported *ASA 3 (n/N, %)*: not reported *ASA 4 (n/N, %)*: not reported *Number of participants randomised*: 48 *Number of participants receiving treatment*: not reported *Number of participants analysed*: 43 *Dropout rate*: 5/43 (11.6%) TXA *Age (years) (mean SD)*: 76 (7) *Ethnicity*: not reported *Gender (males, females)*: 13, 30 *Length of surgery (minutes) (mean SD)*: 96 (18) *Proportion of participants on anticoagulants prior to surgery (n/N, %) (within one week)*: not reported *Incidence of preoperative anaemia*: not reported *Co‐morbidities (n/N, %)*: not reported *ASA 1 (n/N, %)*: not reported *ASA 2 (n/N, %)*: not reported *ASA 3 (n/N, %)*: not reported *ASA 4 (n/N, %)*: not reported *Number of participants randomised*: 48 *Number of participants receiving treatment*: not reported *Number of participants analysed:* 43 *Dropout rate*: 5/43 (11.6%) Overall *Age (years) (mean SD)*: not reported *Ethnicity*: not reported *Gender (male, female)*: not reported *Length of surgery (minutes) (mean SD)*: not reported *Proportion of participants on anticoagulants prior to surgery (n/N, %)*: not reported *Incidence of preoperative anaemia (n/N, %)*: not reported *Co‐morbidities (n/N, %)*: not reported *ASA 1 (n/N, %)*: not reported *ASA 2 (n/N, %)*: not reported *ASA 3 (n/N, %)*: not reported *ASA 4 (n/N, %)*: not reported *Number of participants randomised*: not reported *Number of participants receiving treatment*: not reported *Number of participants analysed*: not reported *Dropout rate (%)*: not reported **Inclusion criteria:** 1) no history of bleeding disorders or warfarin medication, 2) diagnosis of osteoarthritis or aseptic bone necrosis, 3) primary, unilateral, bicompartmental knee arthroplasty, 4) either both or no components cemented, 5) continuous epidural anaesthesia, 6) use of only balanced electrolyte solutions and/or albumin for plasma volume restitution **Exclusion criteria:** rheumatoid arthritis **If TKR, is tourniquet used:** yes **Indication for surgery:** osteoarthritis or aseptic bone necrosis **Type of anaesthetic:** epidural **Type of surgery:** primary TKR	
Interventions	**Intervention characteristics** Placebo Placebo given as a slow intravenous injection towards the end of the operation at a median time of 12 minutes (1 to 40) before deflation of the tourniquet. This dose was repeated after 3 hours from the other ampoule of the pair provided in an envelope. Placebo, IV, intraop 12 mins before deflation of tourniquet + placebo, IV, 3 hours postop (if severe bleeding given additional TXA) TXA Tranexamic acid of 10 mg/kg body weight, maximum 1 g = 10 mL was given as a slow intravenous injection towards the end of the operation at a median time of 12 minutes (1 to 40) before deflation of the tourniquet. This dose was repeated after 3 hours from the other ampoule of the pair provided in an envelope. TXA, IV, 10 mg/kg, intraop 12 mins before deflation of tourniquet + TXA, IV, 10 mg/kg, 3 hours postop (if severe bleeding given additional TXA)	
Outcomes	*Primary outcome:* Blood loss *Secondary outcomes:* Need for blood transfusion Knee circumference Wound complications Range of motion Thromboembolic complications	
Notes	**Sponsorship source:** non‐pharmaceutical **Country**: Sweden **Setting**: not reported **Comments**: none **Author's name:** G. Benoni **Institution:** Malmö University Hospital **Email:** not reported **Address:** Malmö University Hospital, S‐205 02 Malmö, Sweden **Native language of paper:** English **Reference type:** full text (1) **Trial registration number:** not applicable, but when checked, trial registration number not found **Was it translated for this review:** no	
***Risk of bias***		
**Bias**	**Authors' judgement**	**Support for judgement**
Random sequence generation (selection bias)	Unclear risk	Quote: "Randomisation into blocks of 12 was done by an independent pharmacologist" Judgement comment: not enough information to permit judgement.
Allocation concealment (selection bias)	Unclear risk	Quote: "Pairs of ampoules, each containing 10 ml of either the active substance (Cyklokapron 100 mg/ml; Pharmacia, Stockholm, Sweden) or the placebo (physiological saline) were numbered and packed in envelopes which were opened by the anaesthetist before administration. These ampoules could be identified only by their numbers, and the randomisation code was known only to the independent pharmacologist. The code was not broken until the end of the study and until all data had been corrected and included in the database." Judgement comment: unclear whether envelopes were consecutively numbered and opaque.
Blinding of participants and personnel (performance bias) Subjective outcomes	Low risk	Quote: "These ampoules could be identified only by their numbers, and the randomisation code was known only to the independent pharmacologist." Judgement comment: unlikely blinding could have been broken.
Blinding of participants and personnel (performance bias) Objective outcomes	Low risk	Quote: none. Judgement comment: objective outcome for personnel, so low risk of bias regardless of quality of blinding.
Blinding of outcome assessment (detection bias) Subjective outcomes	Low risk	Quote: "The code was not broken until the end of the study and until all data had been corrected and included in the database." Judgement comment: blinding of outcome assessors ensured.
Blinding of outcome assessment (detection bias) Objective outcomes	Low risk	Quote: not applicable Judgement comment: objective outcome for outcome assessor, so low risk of bias regardless of quality of blinding.
Incomplete outcome data (attrition bias) All outcomes	Low risk	Quote: not applicable Judgement comment: no missing data.
Selective reporting (reporting bias)	Unclear risk	Quote: not applicable Judgement comment: no available protocol to check pre‐specified outcomes reported.
Other bias	High risk	Quote: "For patients with severe postoperative bleeding, an extra dose of tranexamic acid was given, without breaking the randomisation code." Judgement comment: potential source of bias related to study design. In addition, discrepancy between how many patients were randomised and how many received treatment.

**Table:** Benoni 2000

***Study characteristics***		
Methods	**Study design:** 2‐arm, RCT **Intention‐to‐treat analysis**: not reported **Duration of study:** not reported **Power calculation reached:** yes **Transfusion strategy:** according to patients’ clinical need, with individual regard to age, cardiovascular status and present clinical condition **Was the trial stopped early:** no	
Participants	**Baseline characteristics** Placebo *Age (years) (mean SD)*: 68 (10) *Ethnicity*: not reported *Gender (males, females)*: 11/19 M (58%); 8/19 F (42%) *Length of surgery (minutes, mean SD)*: 105 (17) *Proportion of participants on anticoagulants prior to surgery (n/N, %)*: not reported *Incidence of preoperative anaemia (n/N, %)*: not reported *Co‐morbidities (n/N, %)*: not reported *ASA 1 (n/N, %)*: not reported *ASA 2 (n/N, %)*: not reported *ASA 3 (n/N, %)*: not reported *ASA 4 (n/N, %)*: not reported *Number of participants randomised*: 20 *Number of participants receiving treatment*: 19 *Number of participants analysed*: 19 *Dropout rate*: 1/20 (5%) TXA *Age (years) (mean SD)*: 69.5 (10) *Ethnicity*: not reported *Gender (males, females)*: 6/20 M (30%); 14/20 F (70%) *Length of surgery (minutes, mean SD)*: 107 (25) *Proportion of participants on anticoagulants prior to surgery (n/N, %)*: not reported *Incidence of preoperative anaemia (n/N, %)*: not reported *Co‐morbidities (n/N, %)*: not reported *ASA 1 (n/N, %)*: not reported *ASA 2 (n/N, %)*: not reported *ASA 3 (n/N, %)*: not reported *ASA 4 (n/N, %)*: not reported *Number of participants randomised*: 20 *Number of participants receiving treatment*: 20 *Number of participants analysed*: 20 *Dropout rate*: 0/20 (0%) Overall *Age (years) (mean SD)*: not reported *Ethnicity*: not reported *Gender (male, female)*: not reported *Length of surgery (minutes) (mean SD)*: not reported *Proportion of participants on anticoagulants prior to surgery (n/N, %)*: not reported *Incidence of preoperative anaemia (n/N, %)*: not reported *Co‐morbidities (n/N, %)*: not reported *ASA 1 (n/N, %)*: not reported *ASA 2 (n/N, %)*: not reported *ASA 3 (n/N, %)*: not reported *ASA 4 (n/N, %)*: not reported *Number of participants randomised*: not reported *Number of participants receiving treatment*: not reported *Number of participants analysed*: not reported *Dropout rate (%)*: not reported **Inclusion criteria:** 1) osteoarthrosis, 2) idiopathic femoral head necrosis undergoing THR **Exclusion criteria:** previously operated hip **If TKR, is tourniquet used:** not applicable **Indication for surgery:** osteoarthrosis or idiopathic femoral head necrosis **Type of anaesthetic:** mixed (continuous epidural, spinal, spinal/epidural, general, general and epidural) **Type of surgery:** primary THR	
Interventions	**Intervention characteristics** Placebo Placebo given intravenously at the end of the operation and the administration was repeated 3 hours later Placebo, IV, intraop + 3 hours postop TXA Tranexamic acid 10 mg/kg body weight intravenously at the end of the operation and the administration was repeated 3 hours later Tranexamic acid, IV, 15 mg/kg, intraop + 3 hours postop	
Outcomes	*Primary outcomes:* Postoperative blood loss Need for blood transfusion Deep venous thrombosis	
Notes	**Sponsorship source:** non‐pharmaceutical **Country**: Sweden **Setting**: not reported **Comments**: none **Author's name:** G. Benoni **Institution:** Malmö University Hospital, Sweden **Email:** not reported **Address:** Departments of Orthopedics, Malmö University Hospital, SE‐205 02 Malmö, Sweden **Native language of paper:** English **Reference type:** full paper (1) **Trial registration number:** not applicable, but when checked trial registration number not found **Was it translated for this review:** no	
***Risk of bias***		
**Bias**	**Authors' judgement**	**Support for judgement**
Random sequence generation (selection bias)	Unclear risk	Quote: not applicable Judgement comment: method of sequence generation for randomisation not described.
Allocation concealment (selection bias)	Low risk	Quote: not applicable Judgement comment: the drug was given from numbered ampoules containing either active substance or placebo and the randomisation was done by a pharmacist not otherwise engaged in the study. The code was not broken until completion of the study when all clinical data had been filed in the statistics program.
Blinding of participants and personnel (performance bias) Subjective outcomes	Unclear risk	Quote: not applicable Judgement comment: blinding of outcome assessor not described.
Blinding of participants and personnel (performance bias) Objective outcomes	Low risk	Quote: not applicable Judgement comment: objective outcome for participants and personnel, so low risk despite quality of blinding.
Blinding of outcome assessment (detection bias) Subjective outcomes	Unclear risk	Quote: not applicable Judgement comment: insufficient information to permit judgement.
Blinding of outcome assessment (detection bias) Objective outcomes	Low risk	Quote: not applicable Judgement comment: no description of outcome assessor blinding.
Incomplete outcome data (attrition bias) All outcomes	Unclear risk	Quote: not applicable Judgement comment: insufficient information to permit judgement.
Selective reporting (reporting bias)	High risk	Quote: not applicable Judgement comment: one or more outcomes of interest are reported incompletely so that they cannot be entered in a meta‐analysis.
Other bias	High risk	Quote: not applicable Judgement comment: obvious baseline imbalances. Uneven number of males to females.

**Table:** Benoni 2001

***Study characteristics***		
Methods	**Study design:** multi‐centre, 2‐arm, RCT **Intention‐to‐treat analysis**: yes **Duration of study:** not reported **Power calculation reached:** yes **Transfusion strategy:** transfusions were given on a case‐by‐case basis with regard to age, cardiovascular status, haemoglobin concentration and blood loss. Most patients who had blood transfusions received these at a haemoglobin concentration between 80 and 100 g/L. **Was the trial stopped early:** no	
Participants	**Baseline characteristics** Placebo *Age (years) (mean SD)*: 68 (9.4) *Ethnicity*: not reported *Gender (males, females)*: 10/20 M (50%); 10/20 F (50%) *Length of surgery (minutes) (mean SD)*: not reported *Proportion of participants on anticoagulants prior to surgery (n/N, %)*: not reported *Incidence of preoperative anaemia (n/N, %)*: not reported *Co‐morbidities (n/N, %)*: not reported *ASA 1 (n/N, %)*: not reported *ASA 2 (n/N, %)*: not reported *ASA 3 (n/N, %)*: not reported *ASA 4 (n/N, %)*: not reported *Number of participants randomised*: 20 *Number of participants receiving treatment*: 20 *Number of participants analysed*: 20 *Dropout rate*: 0/20, (0%) TXA *Age (years) (mean SD)*: 66 (9.5) *Ethnicity*: not reported *Gender (males, females)*: 9/18 M (50%); 9/18 F (50%) *Length of surgery (minutes) (mean SD)*: not reported *Proportion of participants on anticoagulants prior to surgery (n/N, %)*: not reported *Incidence of preoperative anaemia (n/N, %)*: not reported *Co‐morbidities (n/N, %)*: not reported *ASA 1 (n/N, %)*: not reported *ASA 2 (n/N, %)*: not reported *ASA 3 (n/N, %)*: not reported *ASA 4 (n/N, %)*: not reported *Number of participants randomised*: 20 *Number of participants receiving treatment*: 20 *Number of participants analysed*: 18 *Dropout rate*: 2/20 (10%) Overall *Age (years) (mean SD)*: not reported *Ethnicity*: not reported *Gender (male, female)*: not reported *Length of surgery (minutes) (mean SD)*: not reported *Proportion of participants on anticoagulants prior to surgery (n/N, %)*: not reported *Incidence of preoperative anaemia (n/N, %)*: not reported *Co‐morbidities (n/N, %)*: not reported *ASA 1 (n/N, %)*: not reported *ASA 2 (n/N, %)*: not reported *ASA 3 (n/N, %)*: not reported *ASA 4 (n/N, %)*: not reported *Number of participants randomised*: not reported *Number of participants receiving treatment*: not reported *Number of participants analysed*: not reported *Dropout rate (%)*: not reported **Inclusion criteria:** 1) patients scheduled for a unilateral, primary total hip replacement for osteoarthrosis or osteonecrosis **Exclusion criteria:** 1) rheumatoid arthritis, 2) patients who were to undergo bone grafting, 3) bleeding disorders, 4) signs of renal insufficiency **If TKR, is tourniquet used:** not applicable **Indication for surgery:** osteoarthrosis or osteonecrosis **Type of anaesthetic:** mixed (general, spinal and epidural) **Type of surgery:** primary THR	
Interventions	**Intervention characteristics** Placebo The patients received tranexamic acid 100 mg/mL (Cyklokapron, Pharmacia & Upjohn, Sweden), 10 mg/kg body weight (maximum 1 g), in a slow (5 to 10 minutes) intravenous injection or a similar volume of placebo (saline) immediately before the operation started, contained in specially prepared ampoules with 10 mL of the substance, identified by their numbers only Saline was given as a slow (5 to 10 minutes) intravenous injection immediately before the operation started TXA The patients received tranexamic acid 100 mg/mL (Cyklokapron, Pharmacia & Upjohn, Sweden), 10 mg/kg body weight (maximum 1 g), in a slow (5 to 10 minutes) intravenous injection or a similar volume of placebo (saline) immediately before the operation started, contained in specially prepared ampoules with 10 mL of the substance Tranexamic acid 100 mg/mL, 10 mg/kg body weight (maximum 1 g), in a slow (5 to 10 minutes) intravenous injection immediately before the operation started	
Outcomes	*Primary outcomes:* Blood loss Need for transfusion Haemoglobin concentration Clinical events	
Notes	**Sponsorship source:** mixed pharmaceutical and non‐pharmaceutical **Country**: Sweden **Setting**: not reported **Comments**: none **Author's name:** G. Benoni **Institution:** Malmö University Hospital **Email:** goran.benoni@skane.se **Address:** Departments of Orthopaedics and Diagnostic Radiology, Malmö University Hospital, SE‐205 02 Malmö, Sweden **Native language of paper:** English **Reference type:** full text (1) **Trial registration number:** not applicable, but when checked, trial registration number not found **Was it translated for this review:** no	
***Risk of bias***		
**Bias**	**Authors' judgement**	**Support for judgement**
Random sequence generation (selection bias)	Unclear risk	Quote: "the study comprised 20 patients of each sex, randomized separately." Judgement comment: insufficient evidence to permit judgement.
Allocation concealment (selection bias)	Unclear risk	Quote: not applicable Judgement comment: insufficient information to permit judgement.
Blinding of participants and personnel (performance bias) Subjective outcomes	Low risk	Quote: "The hospital’s chief pharmacist, who was not otherwise involved in the study, kept the code, which was broken only after the study." Judgement comment: likely low, as central allocation of intervention by pharmacist only.
Blinding of participants and personnel (performance bias) Objective outcomes	Low risk	Quote: not applicable Judgement comment: objective outcome for personnel, so low risk of bias regardless of quality of blinding
Blinding of outcome assessment (detection bias) Subjective outcomes	Low risk	Quote: not applicable Judgement comment: likely low, as central allocation of interventions and identical intervention regimen.
Blinding of outcome assessment (detection bias) Objective outcomes	Low risk	Quote: not applicable Judgement comment: objective outcome for outcome assessor, so low risk of bias regardless of quality of blinding.
Incomplete outcome data (attrition bias) All outcomes	Low risk	Quote: not applicable Judgement comment: reason for missing outcome data unlikely to be related to true outcome.
Selective reporting (reporting bias)	Unclear risk	Quote: not applicable Judgement comment: no published protocol to compare whether pre‐specified outcomes reported.
Other bias	Unclear risk	Quote: not applicable Judgement comment: insufficient evidence to permit judgement.

**Table:** Boese 2017

***Study characteristics***		
Methods	**Study design:** single‐centre, 2‐arm RCT **Intention‐to‐treat analysis:** per protocol **Duration of study:** 23 months **Power calculation reached:** no **Transfusion strategy:** standard transfusion protocol was utilised, dictating that a transfusion be performed only when the Hb level dropped below 7 g/dL, or below 10 g/dL if there were clinical symptoms related to anaemia **Was the trial stopped early:** yes	
Participants	**Baseline characteristics** TXA *Age (years) (mean SD)*: 64.97 (9.59) *Ethnicity*: not reported *Gender (males, females)*: 29/98 M (30%); 69/98 F (70%) *Length of surgery (minutes) (mean SD)*: 76.67 (25.27) *Proportion of participants on anticoagulants prior to surgery (n/N, %)*: 98/98, (100%) *Incidence of preoperative anaemia (n/N, %)*: not reported *Co‐morbidities (n/N, %)*: not reported *ASA 1 (n/N, %)*: 1/98, (1%) *ASA 2 (n/N, %)*: 37/98, (37.8%) *ASA 3 (n/N, %)*: 55/98, (56.1%) *ASA 4 (n/N, %)*: 5/98, (5.1%) *Number of participants randomised*: 98 *Number of participants receiving treatment*: not reported *Number of participants analysed*: not reported *Dropout rate*: not reported EACA *Age (years) (mean SD)*: 66.12 (9.73) *Ethnicity*: not reported *Gender (males, females)*: 25/96 M (26%), 71/96 F (74%) *Length of surgery (minutes) (mean SD)*: 73.78 (12.71) *Proportion of participants on anticoagulants prior to surgery (n/N, %)*: 96/96, (100%) *Incidence of preoperative anaemia (n/N, %)*: not reported *Co‐morbidities (n/N, %)*: not reported *ASA 1 (n/N, %)*: 2/96, (2.1%) *ASA 2 (n/N, %)*: 38/96, (39.6%) *ASA 3 (n/N, %)*: 53/96, (55.2%) *ASA 4 (n/N, %)*: 3/96, (3.1%) *Number of participants randomised*: 96 *Number of participants receiving treatment*: not reported *Number of participants analysed*: not reported *Dropout rate*: not reported Overall *Age (years) (mean SD)*: not reported *Ethnicity*: not reported *Gender (males, females)*: not reported *Length of surgery (minutes) (mean SD)*: not reported *Proportion of participants on anticoagulants prior to surgery (n/N, %)*: not reported *Incidence of preoperative anaemia (n/N, %)*: not reported *Co‐morbidities (n/N, %)*: not reported *ASA 1 (n/N, %)*: not reported *ASA 2 (n/N, %)*: not reported *ASA 3 (n/N, %)*: not reported *ASA 4 (n/N, %)*: not reported *Number of participants randomised*: not reported *Number of participants receiving treatment*: not reported *Number of participants analysed*: not reported *Drop out rate*: not reported **Inclusion criteria:** 1) patients between the ages of 18 and 90 years who were scheduled to be treated with a unilateral total knee replacement by 1 of 3 surgeons from the same orthopaedic practice were eligible for inclusion **Exclusion criteria:** 1) history of coagulopathy, 2) history of deep vein thrombosis or pulmonary embolism 1 year prior to surgery, 3) treatment with a coronary stent 1 year prior to surgery, 4) preoperative autologous blood donation, 5) being unwilling or unable to take an anticoagulant for prophylaxis against deep vein thrombosis, 6) nonsteroidal anti‐inflammatory drug (NSAID) or platelet antiaggregant treatment 5 days prior to surgery, 7) a recorded preoperative creatinine level of > 1.5 mg/dL, 8) an inability to speak English. **If TKR, is tourniquet used:** yes **Indication for surgery:** not reported **Type of anaesthetic:** mixed (general, spinal and epidural) **Type of surgery:** primary TKR	
Interventions	**Intervention characteristics** TXA Tranexamic acid was given 1 g in 250 mL of saline solution over 30 minutes prior to inflating the tourniquet and making the initial incision. A second 1 g in 250mL of saline solution was infused over 30 minutes beginning at the time of wound closure TXA, IV, 1 g, intraop, 30 mins infusion prior to tourniquet AND TXA, IV, 1 g, intraop, 30 mins infusion at beginning of wound closure EACA EACA was given 7 g in 250 mL of saline solution over 30 minutes just prior to inflating the tourniquet and making the initial incision. A second 7 g of EACA in 250 mL of saline solution was infused over 30 minutes beginning at the time of wound closure EACA, IV, 7 g, intraop, 30 mins infusion prior to tourniquet AND EACA, IV, 7 g, intraop, 30 mins infusion at beginning of wound closure	
Outcomes	*Primary outcomes:* Transfusions Estimated blood loss Drop in the haemoglobin (Hb) level *Secondary outcomes:* Change in the serum creatinine level Postoperative complications Length of hospital stay	
Notes	**Sponsorship source:** non‐pharmaceutical **Country:** USA **Setting**: not reported **Comments**: none **Author's name:** RW Walters **Institution:** Creighton University **Email:** ryanwalters@creighton.edu **Address:** Department of Medicine, Creighton University, Omaha, Nebraska **Native language of paper:** English **Reference type:** full text (1), trial registration (1) **Trial registration number:** NCT01873768 **Was it translated for this review:** no	
***Risk of bias***		
**Bias**	**Authors' judgement**	**Support for judgement**
Random sequence generation (selection bias)	Low risk	Quote: "... study nurses placed patient names onto a permuted‐block randomization table. This table was organized by blocks holding 4 names with numbers that corresponded to the patient's treatment assignment. If a study procedure was cancelled, the next patient on the list was assigned to that cancelled subject's treatment assignment. A separate randomization schedule was created for each surgeon." Judgement comment: use of random number table.
Allocation concealment (selection bias)	Low risk	Quote: "The pharmacist marked each drug as “antifibrinolytic,” and the drug was sent to the operating room with the patient’s name on it. The anesthesiologist then infused the assigned drug per protocol." Judgement comment: centralised allocation.
Blinding of participants and personnel (performance bias) Subjective outcomes	Low risk	Quote: "The pharmacist marked each drug as “antifibrinolytic,” and the drug was sent to the operating room with the patient’s name on it. The anesthesiologist then infused the assigned drug per protocol. The patient, operating surgeon, physician’s assistant, internist, and anesthesiologist were blinded to the administered study drug." Judgement comment: blinding of participants and personnel ensured and unlikely that blinding could have been broken.
Blinding of participants and personnel (performance bias) Objective outcomes	Low risk	Quote: not applicable Judgement comment: objective outcome for personnel, so low risk of bias regardless of quality of blinding.
Blinding of outcome assessment (detection bias) Subjective outcomes	Low risk	Quote: not applicable Judgement comment: blinding of outcome assessors likely ensured.
Blinding of outcome assessment (detection bias) Objective outcomes	Low risk	Quote: not applicable Judgement comment: objective outcome for outcome assessor, so low risk of bias regardless of quality of blinding.
Incomplete outcome data (attrition bias) All outcomes	Unclear risk	Quote: not applicable Judgement comment: no information given on reasons for missing data. Study population reports 96 patients in EACA and 98 patients in TXA, however in the table 2 main analysis, EACA n = 92 and for TXA n = 95. Unclear number of patients in table 3 (reported complications).
Selective reporting (reporting bias)	High risk	Quote: not applicable Judgement comment: one or more outcomes of interest have been reported incompletely such that they cannot be entered into a meta‐analysis.
Other bias	High risk	Quote: "The results of the 2 interim analyses (n = 140 and n = 194) were nearly identical for all primary and secondary outcomes. Specifically, this study was powered to detect the a priori MCID in Hgb level of 10%, but the largest observed difference was consistently near 3%, which we considered to be clinically irrelevant as no transfusions were required and the length of hospital stay was similar between the treatment groups. The consistency of this result across both interim analyses justified stopping the trial at 194 patients; only results from the second interim analysis are presented." Judgement comment: trial stopped early due to data‐dependent process. Per protocol analysis performed.

**Table:** Bradley 2019 hip

***Study characteristics***		
Methods	**Study design:** RCT **Intention‐to‐treat analysis**: yes **Duration of study:** 34 months + 3 months follow‐up **Power calculation reached:** no (study enrolment stopped early therefore unable to reach target sample size) **Transfusion strategy:** patients received a transfusion based on the institutional protocol of Hb < 7.0 g/dL or Hb < 10.0 g/dL with associated symptoms **Was the trial stopped early:** no (enrolment stopped early ‐ but not trial; enrolment onto study terminated early due to national lack of EACA)	
Participants	**Baseline characteristics** TXA *Age (years) (median IQR)*: 61.3 (55.8 to 69.3) *Ethnicity (N, %)*: Caucasian 38 (82.6), African American 7 (15.2), Other 1 (2.2) *Gender (males, females) (N, %)*: 19/46 M (41.3%); 27/46 F (58.7%) *Length of surgery (minutes) (mean SD)*: not reported *Proportion of participants on anticoagulants prior to surgery (n/N, %)*: not reported *Incidence of preoperative anaemia (n/N, %)*: not reported *Co‐morbidities (n/N, %)*: not reported *ASA 1 (n/N, %) or ASA 2 (n/N, %)*: 30 (65.2%) *ASA 2 (n/N, %)*: not reported *ASA 3 (n/N, %)*: not reported *ASA 4 (n/N, %)*: not reported *Number of participants randomised*: not reported *Number of participants receiving treatment*: 46 *Number of participants analysed*: 46 *Dropout rate*: not reported EACA *Age (years) (median IQR)*: 59.2 (50.2 to 68.0) *Ethnicity (N, %)*: Caucasian 36 (81.8), African American 6 (13.6), Other 2 (4.6) *Gender (males, females) (N, %)*: 23/44 M (52.3%); 21/44 F (47.7%) *Length of surgery (minutes) (mean SD)*: not reported *Proportion of participants on anticoagulants prior to surgery (n/N, %)*: not reported *Incidence of preoperative anaemia (n/N, %)*: not reported *Co‐morbidities (n/N, %)*: not reported *ASA 1 (n/N, %) or ASA 2 (n/N, %)*: 24 (54.6) *ASA 2 (n/N, %)*: not reported *ASA 3 (n/N, %)*: not reported *ASA 4 (n/N, %)*: not reported *Number of participants randomised*: not reported *Number of participants receiving treatment*: 44 *Number of participants analysed*: 44 *Dropout rate*: not reported Overall *Age (years) (median IQR)*: not reported *Ethnicity (N, %)*: not reported *Gender (females) (N, %)*: 48/90 *Length of surgery (minutes) (mean IQR)*: 98 minutes (86 to 110) *Proportion of participants on anticoagulants prior to surgery (n/N, %)*: not reported *Incidence of preoperative anaemia (n/N, %)*: not reported *Co‐morbidities (n/N, %)*: not reported *ASA 1 (n/N, %) or ASA 2 (n/N, %)*: not reported *ASA 2 (n/N, %)*: not reported *ASA 3 (n/N, %)*: not reported *ASA 4 (n/N, %)*: not reported *Number of participants randomised*: 94 *Number of participants receiving treatment*: 90 *Number of participants analysed*: 90 *Dropout rate*: 4/90, 4.4% **Inclusion criteria:** all patients aged 18 years and over who were to undergo primary THA or TKA were considered for inclusion in the study **Exclusion criteria:** patients were excluded if they had a history of cardiac stents, myocardial infarction (MI), cerebrovascular accident or stroke, deep venous thrombosis (DVT), pulmonary embolism (PE), late‐onset colour blindness or any known hypercoagulable state **If TKR, is tourniquet used:** not applicable **Indication for surgery:** not reported **Type of anaesthetic:** spinal **Type of surgery:** THR	
Interventions	**Intervention characteristics** TXA TXA was given as 1 g intravenously in 2 doses. The dosage was determined on the recommendation of the cardiac anaesthesiology team at our hospital, noting that TXA binds to plasminogen 6 to 10 times more than EACA TXA IV 1 g pre‐incision, 1 g wound closure EACA Antifibrinolytics were given twice perioperatively. The first dose was given immediately before making the incision and a second dose was given during wound closure. EACA was given as a 5 g dose in 250 mL of normal saline. EACA IV 5 g pre incision, 5 g wound closure	
Outcomes	*Primary outcomes:* Change in haemoglobin level and blood volume Postoperative drainage Rate of transfusion *Secondary outcomes:* Postoperative complications Cost Length of stay (LOS)	
Notes	**Sponsorship source:** hospital funded **Country:** USA **Setting**: single‐centre **Comments**: none **Author's name:** KE Bradley **Institution:** Duke University Medical Center **Email:** kendall.bradley@duke.edu **Address:** Duke University Medical Center, Durham North Carolina, United States **Native language of paper:** English **Reference type:** full text (1), trial registration (1) **Trial registration number:** NCT02030821 **Was it translated for this review:** no	
***Risk of bias***		
**Bias**	**Authors' judgement**	**Support for judgement**
Random sequence generation (selection bias)	Low risk	Quote: "patients were randomized using a random number table generated by the institutional Department of Biostatistics and Bioinformatics" Judgement comment: use of random number table.
Allocation concealment (selection bias)	Unclear risk	Quote: not applicable Judgement comment: insufficient information to permit judgement.
Blinding of participants and personnel (performance bias) Subjective outcomes	Unclear risk	Quote: not applicable Judgement comment: blinding of patients and personnel not mentioned.
Blinding of participants and personnel (performance bias) Objective outcomes	Low risk	Quote: not applicable Judgement comment: objective outcome for personnel, so low risk of bias regardless of quality of blinding.
Blinding of outcome assessment (detection bias) Subjective outcomes	High risk	Quote: not applicable Judgement comment: subjective outcome for personnel and high risk of bias due to inadequate personnel blinding.
Blinding of outcome assessment (detection bias) Objective outcomes	Low risk	Quote: not applicable Judgement comment: objective outcome for outcome assessors, so low risk of bias regardless of quality of blinding
Incomplete outcome data (attrition bias) All outcomes	Unclear risk	Quote: not applicable Judgement comment: unclear initial number randomised.
Selective reporting (reporting bias)	Low risk	Quote: not applicable Judgement comment: reported all outcomes mentioned in the trial registration.
Other bias	Unclear risk	Quote: not applicable Judgement comment: enrolment had to be terminated early due to national shortage of EACA.

**Table:** Bradley 2019 knee

***Study characteristics***		
Methods	**Study design:** RCT **Intention‐to‐treat analysis**: yes **Duration of study:** 34 months + 3 months follow‐up **Power calculation reached:** no (study enrolment stopped early, therefore unable to reach target sample size) **Transfusion strategy:** patients received a transfusion based on the institutional protocol of Hb < 7.0 g/dL or Hb < 10.0 g/dL with associated symptoms **Was the trial stopped early:** no (enrolment stopped early ‐ but not trial)	
Participants	**Baseline characteristics** TXA *Age (years) (median IQR)*: 64.7 (60.7 to 69.6) *Ethnicity (N, %)*: Caucasian 57 (78.1), African American 15 (20.5), Other 1 (1.4) *Gender (males, females)*: 23/73 M (32%); 50/73 F (68%) *Length of surgery (minutes) (mean SD)*: not reported *Proportion of participants on anticoagulants prior to surgery (n/N, %)*: not reported *Incidence of preoperative anaemia (n/N, %)*: not reported *Co‐morbidities (n/N, %)*: not reported *ASA 1 (n/N, %) or ASA 2 (n/N, %)*: 34 (46.6) *ASA 2 (n/N, %)*: not reported *ASA 3 (n/N, %)*: not reported *ASA 4 (n/N, %)*: not reported *Number of participants randomised*: not reported *Number of participants receiving treatment*: not reported *Number of participants analysed*: 73 *Dropout rate*: not reported EACA *Age (years) (median IQR)*: 65.6 (58.8 to 69.4) *Ethnicity (N, %)*: Caucasian 52 (72.2), African American 18 (25.0), Other 2 (2.8) *Gender (males, females) (N)*: 30/72 M (42%); 42/72 F (58%) *Length of surgery (minutes) (mean SD)*: not reported *Proportion of participants on anticoagulants prior to surgery (n/N, %)*: not reported *Incidence of preoperative anaemia (n/N, %)*: not reported *Co‐morbidities (n/N, %)*: not reported *ASA 1 (n/N, %) or ASA 2 (n/N, %)*: 33 (45.8) *ASA 2 (n/N, %)*: not reported *ASA 3 (n/N, %)*: not reported *ASA 4 (n/N, %)*: not reported *Number of participants randomised*: not reported *Number of participants receiving treatment*: not reported *Number of participants analysed*: 72 *Dropout rate*: not reported Overall *Age (years) (median IQR)*: not reported *Ethnicity (N, %)*: not reported *Gender (males, females) (N)*: not reported *Length of surgery (minutes) (mean SD)*: 104 minutes (IQR 90 to 120) *Proportion of participants on anticoagulants prior to surgery (n/N, %)*: not reported *Incidence of preoperative anaemia (n/N, %)*: not reported *Co‐morbidities (n/N, %)*: not reported *ASA 1 (n/N, %) or ASA 2 (n/N, %)*: not reported *ASA 2 (n/N, %)*: not reported *ASA 3 (n/N, %)*: not reported *ASA 4 (n/N, %)*: not reported *Number of participants randomised*: 152 *Number of participants receiving treatment*: 145 *Number of participants analysed*: 145 *Dropout rate*: not reported **Inclusion criteria:** all patients aged 18 years and over who were to undergo primary THA or TKA were considered for inclusion in the study **Exclusion criteria:** 1) history of cardiac stents, 2) myocardial infarction (MI), 3) cerebrovascular accident or stroke, 4) deep venous thrombosis (DVT), 5) pulmonary embolism (PE), 6) late‐onset colour blindness, 7) any known hypercoagulable state **If TKR, is tourniquet used:** yes **Indication for surgery:** not reported **Type of anaesthetic:** for TKA, patients had an adductor canal block and sedation **Type of surgery:** primary TKR	
Interventions	**Intervention characteristics** TXA Antifibrinolytics were given twice perioperatively. The first dose was given immediately before making the incision and a second dose was given during wound closure. EACA was given as a 5 g dose in 250 mL of normal saline, while TXA was given as 1 g intravenously in 2 doses. TXA, IV, 1 g, intraop incision and TXA, IV, 1 g, intraop wound closure EACA EACA was given as a 5 g dose in 250 mL of normal saline EACA, IV, 1 g, intraop incision and EACA, IV, 1 g, intraop wound closure	
Outcomes	*Primary outcomes*: Change in haemoglobin level and blood volume Postoperative drainage Rate of transfusion *Secondary outcomes*: Postoperative complications Cost Length of stay (LOS)	
Notes	**Sponsorship source:** non‐pharmaceutical **Country:** USA **Setting**: single‐centre **Comments**: 1) enrolment onto study terminated early due to national lack of EACA **Author's name:** KE Bradley **Institution:** Duke University Medical Center **Email:** kendall.bradley@duke.edu **Address:** Duke University Medical Center, Durham North Carolina, United States **Native language of paper:** English **Reference type:** full text (1), trial registration (1) **Trial registration number:** NCT02030821 **Was it translated for this review:** no	
***Risk of bias***		
**Bias**	**Authors' judgement**	**Support for judgement**
Random sequence generation (selection bias)	Low risk	Quote: not applicable Judgement comment: after enrolment, patients were randomised using a random number table generated by the institutional Department of Biostatistics and Bioinformatics in a 1:1 ratio to one of two treatment arms: TXA or EACA.
Allocation concealment (selection bias)	Unclear risk	Quote: not applicable Judgement comment: insufficient information to permit judgement.
Blinding of participants and personnel (performance bias) Subjective outcomes	Unclear risk	Quote: not applicable Judgement comment: blinding of patients and personnel not mentioned.
Blinding of participants and personnel (performance bias) Objective outcomes	Low risk	Quote: not applicable Judgement comment: objective outcome for personnel, so low risk of bias regardless of quality of blinding.
Blinding of outcome assessment (detection bias) Subjective outcomes	High risk	Quote: not applicable Judgement comment: subjective outcome for outcome assessors and high risk of bias due to inadequate blinding.
Blinding of outcome assessment (detection bias) Objective outcomes	Low risk	Quote: Not applicable Judgement comment: objective outcome for outcome assessors, so low risk of bias regardless of quality of blinding.
Incomplete outcome data (attrition bias) All outcomes	Unclear risk	Quote: not applicable Judgement comment: insufficient reporting of attrition/exclusions to permit judgement of ‘low risk’ or ‘high risk’.
Selective reporting (reporting bias)	Low risk	Quote: not applicable Judgement comment: all outcomes planned in the protocol or prospective trial registration are reported.
Other bias	Unclear risk	Quote: not applicable Judgement comment: unable to reach sample size due to availability of EACA. Trial terminated due to this reason.

**Table:** Camarasa 2006

***Study characteristics***		
Methods	**Study design:** 3‐arm RCT, unclear whether single‐centre or multi‐centre **Intention‐to‐treat analysis:** yes **Duration of study:** 15 months **Power calculation reached:** yes **Transfusion strategy:** a haemoglobin level of less than 8 g/dL was considered a transfusion trigger except in patients who could have poor tolerance to these levels because of associated conditions such as myocardial ischaemia, chronic obstructive pulmonary disease (COPD), cerebral arterial insufficiency, or patients who presented signs, symptoms, or both, of hypoxia such as tachycardia, dyspnoea or syncope. The transfusion trigger was placed at less than 10 g/dL for these patients. **Was the trial stopped early:** no	
Participants	**Baseline characteristics** Placebo *Age (years) (median range)*: 72 (52 to 85) *Ethnicity*: not reported *Gender (males, females)*: 12/60 M (20%), 48/60 F (80%) *Length of surgery (minutes) (mean SD)*: 102 (19) *Proportion of participants on anticoagulants prior to surgery (n/N, %)*: not reported *Incidence of preoperative anaemia (n/N, %)*: not reported *Co‐morbidities (n/N, %)*: not reported *ASA 1 (n/N, %)*: 3/60 (5%) *ASA 2 (n/N, %)*: 44/60 (73.3%) *ASA 3 (n/N, %)*: 13/60 (21.7%) *ASA 4 (n/N, %)*: 0/60 (0%) *Number of participants randomised*: 60 *Number of participants receiving treatment*: 60 *Number of participants analysed*: 60 *Dropout rate (%)*: 0/60 (0%) TXA *Age (years) (median range)*: 73 (61 to 84) *Ethnicity*: not reported *Gender (males, females)*: 9/35 M (26%), 26/35 F (17%) *Length of surgery (minutes) (mean SD)*: 97 (22) *Proportion of participants on anticoagulants prior to surgery (n/N, %)*: not reported *Incidence of preoperative anaemia (n/N, %)*: not reported *Co‐morbidities (n/N, %)*: not reported *ASA 1 (n/N, %)*: 2/35 (5.7%) *ASA 2 (n/N, %)*: 24/35 (68.6%) *ASA 3 (n/N, %)*: 9/35 (25.7%) *ASA 4 (n/N, %)*: 0/60 (0%) *Number of participants randomised*: 35 *Number of participants receiving treatment*: 35 *Number of participants analysed*: 35 *Dropout rate (%)*: 0/35 (0%) EACA *Age (years) (median range)*: 73 (59 to 80) *Ethnicity*: not reported *Gender (males, females)*: 4/32 M (12.5%), 28/32 F (87.5%) *Length of surgery (minutes) (mean SD)*: 102 (20) *Proportion of participants on anticoagulants prior to surgery (n/N, %)*: not reported *Incidence of preoperative anaemia (n/N, %)*: not reported *Co‐morbidities (n/N, %)*: not reported *ASA 1 (n/N, %)*: 0/32 (0%) *ASA 2 (n/N, %)*: 28/32 (87.5%) *ASA 3 (n/N, %)*: 4/32 (12.5%) *ASA 4 (n/N, %)*: 0/60 (0%) *Number of participants randomised*: 33 *Number of participants receiving treatment*: 33 *Number of participants analysed*: 32 *Dropout rate (%)*: 1/32 (3.1%) Overall *Age (years) (median range)*: not reported *Ethnicity (N, %)*: not reported *Gender (males, females) (N)*: not reported *Length of surgery (minutes) (mean SD)*: not reported *Proportion of participants on anticoagulants prior to surgery (n/N, %)*: not reported *Incidence of preoperative anaemia (n/N, %)*: not reported *Co‐morbidities (n/N, %)*: not reported *ASA 1 (n/N, %) or ASA 2 (n/N, %)*: not reported *ASA 2 (n/N, %)*: not reported *ASA 3 (n/N, %)*: not reported *ASA 4 (n/N, %)*: not reported *Number of participants randomised*: not reported *Number of participants receiving treatment*: not reported *Number of participants analysed*: not reported *Dropout rate*: not reported **Inclusion criteria:** 1) all patients who needed unilateral, bicompartmental, primary, cemented TKR because of osteoarthritis or rheumatoid arthritis, 2) anaesthetic risk groups ASA I–III **Exclusion criteria:** 1) history of coagulopathy or thrombosis, embolism, or both, 2) had received acenocoumarol, aspirin or platelet antiaggregant treatment in the week before surgery, or nonsteroidal anti‐inflammatory agents in the 2 days before surgery, 3) preoperative plasma creatinine greater than 130 mmol/L, 4) history of myocardial infarction or chronic arteriopathy, 5) unstable angina in the previous 12 months, 6) mental states prevented them from understanding the study proposal **If TKR, is tourniquet used:** yes **Indication for surgery:** osteoarthritis or rheumatoid arthritis **Type of anaesthetic:** spinal anaesthetic **Type of surgery:** primary TKR	
Interventions	**Intervention characteristics** Placebo Major bleeding in this type of surgery occurs in the first hours, so a regimen of 2 doses, 3 h apart, was chosen for the effect to last over the first 6 h. With regard to EACA, the dose chosen (100 mg kg1) was mid‐way between doses used in other studies and the dose recommended by the laboratory (Fides Ecopharma) and was followed by a perfusion of 1 g h1 for 3 h, similar to doses used in the studies mentioned. The first dose of antifibrinolytics was administered over 30 min immediately before releasing the tourniquet, which prevented the agent from reaching the lower limb. Placebo, IV, intraop bolus (before tourniquet deflation) AND placebo, IV, intraop infusion AND placebo, IV, 3 hours later bolus TXA Patients in the study group received tranexamic acid 10 mg kg1 IV just before the tourniquet was deflated and 3 h later, or epsilon‐aminocaproicacid 100 mg kg1 before tourniquet deflation followed by continuous perfusion (1 g h1) during 3 h TXA, IV, 10 mg/kg, intraop bolus (before tourniquet deflation) AND placebo, IV, intraop infusion AND TXA, IV, 10 mg/kg 3 hours later bolus EACA Major bleeding in this type of surgery occurs in the first hours so a regimen of 2 doses, 3 h apart, was chosen for the effect to last over the first 6 h. With regard to EACA, the dose chosen (100 mg kg1) was mid‐way between doses used in other studies and the dose recommended by the laboratory (Fides Ecopharma) and was followed by a perfusion of 1 g h1 for 3 h, similar to doses used in the studies mentioned. The first dose of antifibrinolytics was administered over 30 min immediately before releasing the tourniquet which prevented the agent from reaching the lower limb. EACA, IV, 100 mg/kg, intraop bolus (before tourniquet deflation) AND EACA, IV, 100 mg/kg intraop infusion AND placebo, IV, 3 hours later bolus	
Outcomes	*Primary outcome:* Reduction in perioperative bleeding	
Notes	**Sponsorship source:** non‐pharmaceutical **Country:** Spain **Setting:** not reported **Comments**: none **Author's name:** MA Camarasa **Institution:** Hospital de Mataro **Email:** 12517mcg@comb.es **Address:** C/Marıa Vidal 202 A, 3‐1a, 08340 Vilassar de Mar, Barcelona, Spain **Native language of paper:** English **Reference type:** full text (1) **Trial registration number:** not applicable, but when checked, trial registration number not found **Was it translated for this review:** no	
***Risk of bias***		
**Bias**	**Authors' judgement**	**Support for judgement**
Random sequence generation (selection bias)	Low risk	Quote: not applicable Judgement comment: computer‐generated randomisation.
Allocation concealment (selection bias)	Unclear risk	Quote: "The randomized assignment was sealed in an opaque, numbered envelope which was opened only by the nurse who prepared the endovenous solutions." Judgement comment: does not mention 'sequential' or 'consecutively numbered.'
Blinding of participants and personnel (performance bias) Subjective outcomes	Low risk	Quote: "This nurse was the only person who knew the patients’ study groups and did not participate in any other phase of the trial. Masking was ensured by the administration of three apparently identical saline drips to each patient: the first, 50 ml; the second, 250 ml for 3 h continuous perfusion, and the third, 50 ml. Medication or placebo was added to the saline drip according to the protocol, as shown in Figure 1. Neither the patient nor the anaesthetist who assessed the results knew the patient’s study group." Judgement comment: blinding of participants and key study personnel ensured, and unlikely that the blinding could have been broken.
Blinding of participants and personnel (performance bias) Objective outcomes	Low risk	Quote: "This nurse was the only person who knew the patients’ study groups and did not participate in any other phase of the trial." Judgement comment: objective outcome for participants and personnel, so low risk despite quality of blinding.
Blinding of outcome assessment (detection bias) Subjective outcomes	Low risk	Quote: "Neither the patient nor the anaesthetist who assessed the results knew the patient’s study group." Judgement comment: blinding of participants and key study personnel ensured, and unlikely that the blinding could have been broken.
Blinding of outcome assessment (detection bias) Objective outcomes	Low risk	Quote: not applicable Judgement comment: objective outcome for outcome assessor so low risk of bias regardless of blinding.
Incomplete outcome data (attrition bias) All outcomes	High risk	Quote: "Venous echo‐Doppler was performed on four of these patients and the results were negative in all cases. In the telephone survey 3 months after the operation, six patients were not located, one had died from aggravation of a prior condition of pulmonary fibrosis, and five suspected episodes of thrombosis were resolved by examining clinical records." Quote: "There were no postoperative complications and follow‐up of possible thromboembolisms revealed eight patients with suspected deep vein thrombosis attributable to an increase in circumference and major oedema" Judgement comment: numbers reported do not account for all patients who may have had a thromboembolic event. Reason for missing outcome data may be related to the true outcome.
Selective reporting (reporting bias)	Unclear risk	Quote: not applicable Judgement comment: paper reports no thromboembolic events but unclear as to how many patients in each arm had a suspected thrombosis. No protocol to check pre‐specified outcomes reported.
Other bias	Low risk	Quote: not applicable Judgement comment: appears to be free of other bias.

**Table:** Cao 2018

***Study characteristics***		
Methods	**Study design:** RCT **Intention‐to‐treat analysis:** yes **Duration of study:** 8 months **Power calculation reached:** yes **Transfusion strategy:** transfusions were applied if the Hb level was <70 or 70 to 100 g/L with symptoms of anaemia (defined as bad mental status, palpitation or shortness of breath not due to other causes), according to the guidelines by the National Ministry of Health **Was the trial stopped early:** no	
Participants	**Baseline characteristics** TXA + placebo repeated dose *Age (years) (mean SD)*: 64.6 (12.8) *Ethnicity*: not reported *Gender (males, females)*: 27/51 M (53%); 24/51 F (47%) *Length of surgery (minutes) (mean SD)*: not reported *Proportion of participants on anticoagulants prior to surgery (n/N, %)*: not reported *Incidence of preoperative anaemia (n/N, %)*: not reported *Co‐morbidities (n/N, %)*: not reported *ASA 1 (n/N, %)*: not reported *ASA 2 (n/N, %)*: not reported *ASA 3 (n/N, %)*: not reported *ASA 4 (n/N, %)*: not reported *Number of participants randomised*: 51 *Number of participants receiving treatment*: 51 *Number of participants analysed*: 51 *Dropout rate*: 0/51, (0%) TXA repeated dose + placebo repeated dose *Age (years) (mean SD)*: 64.2 (10.1) *Ethnicity*: not reported *Gender (males, females)*: 20/51 M (39%), 31/51 F (61%) *Length of surgery (minutes) (mean SD)*: not reported *Proportion of participants on anticoagulants prior to surgery (n/N, %)*: not reported *Incidence of preoperative anaemia (n/N, %)*: not reported *Co‐morbidities (n/N, %):* not reported *ASA 1 (n/N, %)*: not reported *ASA 2 (n/N, %)*: not reported *ASA 3 (n/N, %)*: not reported *ASA 4 (n/N, %)*: not reported *Number of participants randomised*: 51 *Number of participants receiving treatment*: 51 *Number of participants analysed*: 51 *Dropout rate*: 0/51, (0%) TXA repeated dose *Age (years) (mean SD)*: 64.9 (11.9) *Ethnicity*: not reported *Gender (males, females)*: 22/50 M (44%), 28/50 F (56%) *Length of surgery (minutes) (mean SD)*: not reported *Proportion of participants on anticoagulants prior to surgery (n/N, %)*: not reported *Incidence of preoperative anaemia (n/N, %)*: not reported *Co‐morbidities (n/N, %)*: not reported *ASA 1 (n/N, %)*: not reported *ASA 2 (n/N, %)*: not reported *ASA 3 (n/N, %)*: not reported *ASA 4 (n/N, %)*: not reported *Number of participants randomised*: 50 *Number of participants receiving treatment*: 50 *Number of participants analysed*: 50 *Dropout rate*: 0/50, (0%) Overall *Age (years) (mean SD)*: not reported *Ethnicity*: not reported *Gender (males, females)*: not reported *Length of surgery (minutes) (mean SD)*: not reported *Proportion of participants on anticoagulants prior to surgery (n/N, %)*: not reported *Incidence of preoperative anaemia (n/N, %)*: not reported *Co‐morbidities (n/N, %)*: not reported *ASA 1 (n/N, %)*: not reported *ASA 2 (n/N, %)*: not reported *ASA 3 (n/N, %)*: not reported *ASA 4 (n/N, %)*: not reported *Number of participants randomised*: not reported *Number of participants receiving treatment*: not reported *Number of participants analysed*: not reported *Dropout rate*: not reported **Inclusion criteria:** all patients undergoing primary unilateral THA for osteoarthritis, osteonecrosis of the femoral head and developmental dysplasia of hip (Crowe I/II) **Exclusion criteria:** 1) patients with anaemia (< 120 g/L for female, < 130 g/L for male), 2) history of deep venous thrombosis (DVT) or pulmonary embolism (PE), 3) cardiovascular problems, 4) congenital or acquired clotting disorders, 5) known allergy to TXA, 6) renal insufficiency **If TKR, is tourniquet used:** not applicable **Indication for surgery:** osteoarthritis, osteonecrosis of the femoral head and developmental dysplasia of hip **Type of anaesthetic:** general **Type of surgery:** primary THR	
Interventions	**Intervention characteristics** TXA + placebo repeated dose Group A: patients received 2 g of oral TXA using 4 tablets of 500 mg 2 hours pre‐operatively and 4 placebo tablets, identical in appearance with no active ingredient, 4, 10 and 16 hours postoperatively TXA, 2 g, oral, 2 hours preop AND placebo, oral, 4, 10, 16 hours postop TXA repeated dose + placebo repeated dose Group B: patients received 2 g of oral TXA 2 hours pre‐operatively and 4 hours postoperatively along with 4 placebo tablets 10 and 16 hours postoperatively TXA 2 g, oral, 2 hours preop + 4 hours postop AND placebo, oral, 10 and 16 hours postop TXA repeated dose Group C: patients received 2 g of oral TXA 2 hours pre‐operatively and then 3 more doses 4, 10 and 16 hours postoperatively TXA, 2 g, oral, 2 hours preop + 4, 10, 16 hours postop	
Outcomes	*Primary outcomes:* Total blood loss (TBL) Hidden blood loss (HBL) Transfusion rate *Secondary outcomes:* Haemoglobin (Hb) and haematocrit (Hct) drop Level of fibrinolysis parameters (fibrin degradation products, D‐dimer) Complications (thrombotic diseases, stroke, cardiac infarction and infection)	
Notes	**Sponsorship source:** non‐pharmaceutical **Country:** China **Setting:** single‐centre **Comments**: none **Author's name:** G Cao **Institution:** West China Hospital, West China Medical School, Sichuan University **Email:** scucao@126.com **Address:** Department of Orthopaedic Surgery, West China Hospital, West China Medical School, Sichuan University, 37# Wainan Guoxue Road, Chengdu, Sichuan Province, People’s Republic of China **Native language of paper:** English **Reference type:** full text **Trial registration number:** ChiCTR‐IPR‐17012266 **Was it translated for this review:** no	
***Risk of bias***		
**Bias**	**Authors' judgement**	**Support for judgement**
Random sequence generation (selection bias)	Unclear risk	Quote: "Enrolled patients were randomly assigned into three study groups." Judgement comment: not enough information to permit judgement.
Allocation concealment (selection bias)	Unclear risk	Quote: "Randomization was blind and performed with the use of sealed envelopes opened just prior to surgery" Judgement comment: does not mention if envelopes were opaque or sequentially numbered.
Blinding of participants and personnel (performance bias) Subjective outcomes	Low risk	Quote: "A nurse not involved in the trial implemented peri‐operative protocol. The patients, surgeons, data collector, and analyst were blinded." Judgement comment: blinding of key study personnel and participants ensured and likely not to have been broken.
Blinding of participants and personnel (performance bias) Objective outcomes	Low risk	Quote: "A nurse not involved in the trial implemented peri‐operative protocol. The patients, surgeons, data collector, and analyst were blinded." Judgement comment: objective outcome for personnel, so low risk of bias regardless of quality of blinding.
Blinding of outcome assessment (detection bias) Subjective outcomes	Low risk	Quote: "A nurse not involved in the trial implemented peri‐operative protocol. The patients, surgeons, data collector, and analyst were blinded." Judgement comment: blinding of outcome assessors ensured and likely not to have been broken.
Blinding of outcome assessment (detection bias) Objective outcomes	Low risk	Quote: "A nurse not involved in the trial implemented peri‐operative protocol. The patients, surgeons, data collector, and analyst were blinded." Judgement comment: objective outcome for personnel, so low risk of bias regardless of quality of blinding
Incomplete outcome data (attrition bias) All outcomes	Low risk	Quote: not applicable Judgement comment: no missing outcome data.
Selective reporting (reporting bias)	Unclear risk	Quote: not applicable Judgement comment: paper mentions length of hospital stay had been measured, but then does not report data for it.
Other bias	Low risk	Quote: not applicable Judgement comment: the study appears to be free of other sources of bias.

**Table:** Chang 2022

***Study characteristics***		
Methods	**Study design:** RCT **Intention‐to‐treat analysis:** yes **Duration of study:** July 2017 to March 2019 (20 months) + 6 weeks follow‐up = 21.5 months **Power calculation reached:** yes **Transfusion strategy:** a restrictive transfusion trigger (Hb < 7.0 g/dL) was used. When the Hb was > 7.0 g/dL but < 8 g/dL, transfusion was administered only when the patient showed symptoms of anaemia such as severe fatigue, palpitations or dyspnoea **Was the trial stopped early:** no	
Participants	**Baseline characteristics** TXA, IV *Age (years) (mean SD)*: 71.0 (5.6) *Ethnicity*: not reported *Gender (males, females)*: 4/48 (8.3%) M, 44/48 (91.7%) F *Length of surgery (minutes) (mean SD)*: not reported *Proportion of participants on anticoagulants prior to surgery (n/N, %)*: excluded *Incidence of preoperative anaemia (n/N, %)*: not reported *Co‐morbidities (n/N, %)*: not reported *ASA 1 (n/N, %)*: 5/48, 10.4% *ASA 2 (n/N, %)*: 42/48, 87.5% *ASA 3 (n/N, %)*: 1/48, 2.1% *ASA 4 (n/N, %)*: excluded *Number of participants randomised*: 48 *Number of participants receiving treatment*: 48 *Number of participants analysed*: for radiographic DVT outcome 47; all other outcomes 48 *Dropout rate*: 0/48, 0% TXA, IV + TXA, oral days 1 and 2 *Age (years) (mean SD)*: 70.1 (6.3) *Ethnicity*: not reported *Gender (males, females)*: 3/46 (6.5%) M, 43/46 (93.5%) F *Length of surgery (minutes) (mean SD)*: not reported *Proportion of participants on anticoagulants prior to surgery (n/N, %)*: excluded *Incidence of preoperative anaemia (n/N, %)*: not reported *Co‐morbidities (n/N, %)*: not reported *ASA 1 (n/N, %)*: 8/46, 17.4% *ASA 2 (n/N, %)*: 38/46, 82.6% *ASA 3 (n/N, %)*: 0/46, 0% *ASA 4 (n/N, %)*: excluded *Number of participants randomised*: 46 *Number of participants receiving treatment*: 46 *Number of participants analysed*: for radiographic DVT outcome 45; all other outcomes 46 *Dropout rate*: 0/46, 0% TXA, IV + TXA, oral days 1 to 5 *Age (years) (mean SD)*: 69.6 (6.6) *Ethnicity*: not reported *Gender (males, females)*: 5/47 (10.6%) M, 42/47 (89.4%) F *Length of surgery (minutes) (mean SD)*: not reported *Proportion of participants on anticoagulants prior to surgery (n/N, %)*: excluded *Incidence of preoperative anaemia (n/N, %)*: not reported *Co‐morbidities (n/N, %)*: not reported *ASA 1 (n/N, %)*: 6/47, 12.8% *ASA 2 (n/N, %)*: 40/47, 85.1% *ASA 3 (n/N, %)*: 1/47, 2.1% *ASA 4 (n/N, %)*: excluded *Number of participants randomised*: 47 *Number of participants receiving treatment*: 47 *Number of participants analysed*: for radiographic DVT outcome 45; all other outcomes 47 *Drop out rate*: 0/47, 0% Overall *Age (years) (mean SD)*: not reported *Ethnicity*: not reported *Gender (males, females)*: 12/141, 8.5% M; 129/141, 91.5% *Length of surgery (minutes) (mean SD)*: not reported *Proportion of participants on anticoagulants prior to surgery (n/N, %)*: excluded *Incidence of preoperative anaemia (n/N, %)*: not reported *Co‐morbidities (n/N, %)*: not reported *ASA 1 (n/N, %)*: 19/141, 13.5% *ASA 2 (n/N, %)*: 120/141, 85.1% *ASA 3 (n/N, %)*: 2/141, 1.4% *ASA 4 (n/N, %)*: excluded *Number of participants randomised*: 141 *Number of participants receiving treatment*: 141 *Number of participants analysed*: 141 *Dropout rate*: 0/141, 0% **Inclusion criteria:** patients with knee osteoarthritis (Kellgren‐Lawrence grades III and IV) ‐ primary unilateral TKA at our institution ‐ when they were ≥ 18 years of age **Exclusion criteria:** 1) patients with an allergy to TXA, 2) secondary or inflammatory arthritis, 3) history of thromboembolism (including DVT, PE, cerebral infarction, transitory ischaemic attacks, myocardial infarction or angina pectoris), 4) congenital or acquired blood coagulation disease, 5) renal disease that precluded the use of contrast agents, 6) cancer, 7) ASA grade 4 systemic disease, 8) a preoperative prothrombin time (PT) international normalised ratio (INR) of > 1.4, 9) smokers, 10) premenopausal female patients, 11) patients who were administered anticoagulants other than aspirin (including adenosine diphosphate receptor inhibitors, vitamin K antagonists, factor‐Xa inhibitors and direct thrombin inhibitors), 12) patients who were administered aspirin within 5 days preoperatively **If TKR, is tourniquet used:** yes **Indication for surgery:** end‐stage knee osteoarthritis **Type of anaesthetic:** spinal **Type of surgery:** Uunilateral primary TKA	
Interventions	**Intervention characteristics** TXA, IV All patients received IV TXA (10 mg/kg) TXA, IV, 10 mg/kg, intraop TXA, IV + TXA, oral days 1 and 2 All patients received IV TXA (10 mg/kg). Oral administration consisted of 1.5 g of oral TXA (250 mg/capsule; 2 capsules thrice daily) on postoperative days (PODs) 1 and 2 for group 2D TXA, IV, 10 mg/kg, intraop + TXA, oral, 250 mg, postop, multiple dose, postop day 1, 2 TXA, IV + TXA, oral days 1 to 5 All patients received IV TXA (10 mg/kg). Oral administration consisted of 1.5 g of oral TXA (250 mg/capsule; 2 capsules thrice daily) on each day from POD 1 to 5 in group 5D TXA, IV, 10 mg/kg, intraop + TXA, oral, 250 mg, postop, multiple dose, postop day 1 to 5	
Outcomes	*Primary outcome:* Maximal Hb drop *Secondary outcomes:* Hb drops Estimated blood loss Transfusion rate Complications	
Notes	**Sponsorship source:** non‐pharmaceutical **Country:** South Korea **Setting:** single‐centre **Comments**: we took the symptomatic DVT numbers for our outcome, but radiographic evidence of DVT was also presented as an outcome in the paper. Time point for DVT and all other outcomes in this paper is 6 weeks **Author's name:** MJ Chang **Institution:** SMG‐SNU Boramae Medical Center **Email:** corresponding author: ossbkang@gmail.com **Address:** Department of Orthopedic Surgery, Seoul National University College of Medicine, SMG‐SNU Boramae Medical Center, Seoul, South Korea **Native language of paper:** English **Reference type:** full text (1) **Trial registration number:** NCT03109652 **Was it translated for this review:** no	
***Risk of bias***		
**Bias**	**Authors' judgement**	**Support for judgement**
Random sequence generation (selection bias)	Low risk	Quote: "The randomization of the participants was performed via the block randomization method by a third party using a computer program (R software, version 3.4.0; The R Foundation for Statistical Computing) at a ratio of 1:1:1" Judgement comment: adequate method of sequence generation with computer‐generated random numbers.
Allocation concealment (selection bias)	Low risk	Quote: "Random group assignment was generated at the time of the patient’s consent to participate. Each patient was issued a unique identification number. The randomization table was managed by a clinical investigator who did not participate in the collection of the study data or the data analysis." Judgement comment: adequate method of allocation concealment using independent study investigator who carried out the allocation concealment.
Blinding of participants and personnel (performance bias) Subjective outcomes	Unclear risk	Quote: "Patient allocation was unblinded after surgery as no placebo oral medication was used" Judgement comment: unblinded study with no placebo used for oral medication.
Blinding of participants and personnel (performance bias) Objective outcomes	Low risk	Quote: not applicable Judgement comment: objective outcome for personnel, so low risk of bias regardless of quality of blinding.
Blinding of outcome assessment (detection bias) Subjective outcomes	Unclear risk	Quote: not applicable Judgement comment: study is described as "single blind (outcomes assessor)" in trial registration, but no clear description of this in the text.
Blinding of outcome assessment (detection bias) Objective outcomes	Low risk	Quote: not applicable Judgement comment: objective outcome for outcome assessor, so low risk of bias regardless of quality of blinding.
Incomplete outcome data (attrition bias) All outcomes	Low risk	Quote: not applicable Judgement comment: no obvious outcome data missing. All randomised participants were included in the final analysis for all outcomes except radiographic evidence of DVT (1 participant excluded from each group. In Group IV, one participant had PE; in the other groups 1 participant each refused CT angiography).
Selective reporting (reporting bias)	Low risk	Quote: not applicable Judgement comment: all outcomes planned in the protocol or prospective trial registration are reported.
Other bias	Low risk	Quote: not applicable Judgement comment: premenopausal women were excluded.

**Table:** Chin 2020

***Study characteristics***		
Methods	**Study design:** RCT **Intention‐to‐treat analysis:** yes **Duration of study:** 6 years 11 months (from trial registration) **Power calculation reached:** no **Transfusion strategy:** a blood transfusion threshold of Hb < 80 g/dL was instated. However, patients with symptomatic anaemia with Hb in excess of 80 g/dL were also transfused on clinical reasoning **Was the trial stopped early:** probably ‐ target 200 and 88 achieved, long length of duration of study	
Participants	**Baseline characteristics** Placebo *Age (years) (mean SD)*: not reported *Ethnicity*: not reported *Gender (males, females)*: not reported *Length of surgery (minutes) (mean SD)*: not reported *Proportion of participants on anticoagulants prior to surgery (n/N, %)*: these patients were excluded *Incidence of preoperative anaemia (n/N, %)*: not reported *Co‐morbidities (n/N, %)*: not reported *ASA 1 (n/N, %)*: not reported *ASA 2 (n/N, %)*: not reported *ASA 3 (n/N, %)*: not reported *ASA 4 (n/N, %)*: not reported *Number of participants randomised*: not reported *Number of participants receiving treatment*: not reported *Number of participants analysed*: not reported *Dropout rate*: not reported TXA, 1g, IV *Age (years) (mean SD)*: not reported *Ethnicity*: not reported *Gender (males, females)*: not reported *Length of surgery (minutes) (mean SD)*: not reported *Proportion of participants on anticoagulants prior to surgery (n/N, %)*: these patients were excluded *Incidence of preoperative anaemia (n/N, %)*: not reported *Co‐morbidities (n/N, %)*: not reported *ASA 1 (n/N, %)*: not reported *ASA 2 (n/N, %)*: not reported *ASA 3 (n/N, %)*: not reported *ASA 4 (n/N, %)*: not reported *Number of participants randomised*: not reported *Number of participants receiving treatment*: not reported *Number of participants analysed*: not reported *Dropout rate*: not reported Overall *Age (years) (mean SD)*: not reported *Ethnicity*: not reported *Gender (males, females)*: not reported *Length of surgery (minutes) (mean SD)*: not reported *Proportion of participants on anticoagulants prior to surgery (n/N, %)*: these patients were excluded *Incidence of preoperative anaemia (n/N, %)*: not reported *Co‐morbidities (n/N, %)*: not reported *ASA 1 (n/N, %)*: not reported *ASA 2 (n/N, %)*: not reported *ASA 3 (n/N, %)*: not reported *ASA 4 (n/N, %)*: not reported *Number of participants randomised*: 181 *Number of participants receiving treatment*: 181 *Number of participants analysed*: 88 *Dropout rate*: not reported **Inclusion criteria:** patients undergoing elective THA for osteoarthritis or osteonecrosis. **Exclusion criteria:** patients were excluded if they had contraindications for TXA (history or risk of thrombosis, active thromboembolic disease and acquired disturbance of colour vision) or were on clopidogrel, ticagrelor, warfarin, dabigatran or any other anticoagulant. Patients with renal failure or bleeding disorders were also excluded. Those on aspirin were asked to stop 1 week prior to surgery. This was recommenced on day 1 postoperation. Those patients that did not receive a single‐dose spinal anaesthetic were excluded. **If TKR, is tourniquet used:** not applicable **Indication for surgery:** osteoarthritis or osteonecrosis **Type of anaesthetic:** this RCT only included those patients that received a spinal anaesthetic (± general anaesthetic) as part of their procedure. **Type of surgery:** elective THA	
Interventions	**Intervention characteristics** Placebo Placebo of IV saline prior to skin incision Placebo, IV, 1 g, preop TXA, 1g, IV 1 g IV TXA prior to skin incision TXA, IV, 1g, preop	
Outcomes	*Primary outcomes:* Intraoperative blood loss Transfusion rate *Secondary outcomes:* Time to discharge Functional outcome scores (Oxford Hip Score, Western Ontario and McMaster Universities Arthritis Index (WOMAC) and the High Activity Arthroplasty Score (HAAS)) Thromboembolic complications	
Notes	**Sponsorship source:** non‐pharmaceutical (this research was supported by New Zealand Orthopaedic Association (grant no. NZOA JB1) **Country:** New Zealand **Setting:** single‐centre **Comments**: 1) lots of demographic data (age, gender etc) not reported, as well as denominator information being unclear particularly for outcomes **Author's name:** J Chin **Institution:** University of Otago **Email:** kieserdavid@gmail.com **Address:** Department of Orthopaedic Surgery and Musculoskeletal Medicine, University of Otago, Christchurch, New Zealand **Native language of paper:** English **Reference type:** full text (1) **Trial registration number:** ACTRN12610001065088 **Was it translated for this review:** no	
***Risk of bias***		
**Bias**	**Authors' judgement**	**Support for judgement**
Random sequence generation (selection bias)	Low risk	Quote: "Patients were randomised based on a computer‐generated" Judgement comment: none
Allocation concealment (selection bias)	Low risk	Quote: "unblinded hospital pharmacist who dispensed TXA or placebo in a covered and blinded syringe to the anaesthetist just prior to skin incision." Judgement comment: none
Blinding of participants and personnel (performance bias) Subjective outcomes	Low risk	Quote: not applicable Judgement comment: blinded syringe to anaesthetist prior to skin incision.
Blinding of participants and personnel (performance bias) Objective outcomes	Low risk	Quote: not applicable Judgement comment: objective outcome for personnel, so low risk of bias regardless of quality of blinding.
Blinding of outcome assessment (detection bias) Subjective outcomes	Unclear risk	Quote: not applicable Judgement comment: insufficient information to permit judgement.
Blinding of outcome assessment (detection bias) Objective outcomes	Low risk	Quote: not applicable Judgement comment: objective outcome for personnel, so low risk of bias regardless of quality of blinding.
Incomplete outcome data (attrition bias) All outcomes	High risk	Quote: "Most patients were excluded following randomisation and ad‐ ministration of intervention/placebo due to progression to general anaesthetic without a spinal anaesthetic or having incomplete data collection" Judgement comment: none
Selective reporting (reporting bias)	Low risk	Quote: not applicable Judgement comment: all outcomes planned in the protocol or prospective trial registration are reported.
Other bias	Unclear risk	Judgement comment: unclear on basic patient demographics, significant amount of important information not reported.

**Table:** Claeys 2007

***Study characteristics***		
Methods	**Study design:** RCT **Intention‐to‐treat analysis:** yes **Duration of study:** not reported **Power calculation reached:** not reported **Transfusion strategy:** the indication for blood transfusion of packed cells was set at Hb < 8.5 g/dL or Hct < 27% **Was the trial stopped early:** no	
Participants	**Baseline characteristics** Placebo *Age (years) (mean SD)*: 68 (11) *Ethnicity*: not reported *Gender (males, females)*: 7/20 M (35%); 13/20 F (65%) *Length of surgery (minutes) (mean SD)*: 94 (15) *Proportion of participants on anticoagulants prior to surgery (n/N, %)*: 0/20 (0%) *Incidence of preoperative anaemia (n/N, %)*: not reported *Co‐morbidities (n/N, %)*: not reported *ASA 1 (n/N, %)*: not reported *ASA 2 (n/N, %)*: not reported *ASA 3 (n/N, %)*: not reported *ASA 4 (n/N, %)*: not reported *Number of participants randomised*: 20 *Number of participants receiving treatment*: 20 *Number of participants analysed*: 20 *Dropout rate*: 0/20 (0%) TXA *Age (years) (mean SD)*: 73 (8) *Ethnicity*: not reported *Gender (males, females)*: 5/20 M (25%), 15/20 F (75%) *Length of surgery (minutes) (mean SD)*: 98 (18) *Proportion of participants on anticoagulants prior to surgery (n/N, %)*: 0/20 (0%) *Incidence of preoperative anaemia (n/N, %)*: not reported *Co‐morbidities (n/N, %)*: not reported *ASA 1 (n/N, %)*: not reported *ASA 2 (n/N, %)*: not reported *ASA 3 (n/N, %)*: not reported *ASA 4 (n/N, %)*: not reported *Number of participants randomised*: 20 *Number of participants receiving treatment*: 20 *Number of participants analysed*: 20 *Dropout rate*: 0/20 (0%) Overall *Age (years) (mean SD)*: not reported *Ethnicity*: not reported *Gender (males, females)*: not reported *Length of surgery (minutes) (mean SD)*: not reported *Proportion of participants on anticoagulants prior to surgery (n/N, %)*: not reported *Incidence of preoperative anaemia (n/N, %)*: not reported *Co‐morbidities (n/N, %)*: not reported *ASA 1 (n/N, %)*: not reported *ASA 2 (n/N, %)*: not reported *ASA 3 (n/N%)*: not reported *ASA 4 (n/N, %)*: not reported *Number of participants randomised*: not reported *Number of participants receiving treatment*: not reported *Number of participants analysed*: not reported *Dropout rate*: not reported **Inclusion criteria:** 1) scheduled for unilateral elective total hip replacement, 2) ASA I‐II **Exclusion criteria:** 1) allergy to tranexamic acid, 2) preoperative renal or hepatic dysfunction, 3) known bleeding disorders or preoperative coagulation anomalies, 4) anticoagulant or aspirin‐like medication and long‐acting NSAID medication **If TKR, is tourniquet used:** not applicable **Indication for surgery:** degenerative osteoarthrosis **Type of anaesthetic:** spinal anaesthesia **Type of surgery:** primary THR	
Interventions	**Intervention characteristics** Placebo The patients were randomly allocated into 2 groups in a double‐blind fashion and received either tranexamic acid (15 mg/kg) or an equal volume of saline in a slow infusion 15 minutes before surgery. Placebo, IV, preop (15 mins before surgery) TXA The patients were randomly allocated into 2 groups in a double‐blind fashion and received either tranexamic acid (15 mg/kg) or an equal volume of saline in a slow infusion 15 minutes before surgery. TXA, IV, 15 mg/kg, preop (15 mins before surgery)	
Outcomes	*Primary outcome* Blood loss *Secondary outcomes:* Need for blood transfusion Incidence of DVT	
Notes	**Sponsorship source:** not reported **Country:** Belgium **Setting:** not reported **Comments:** unable to work out SD for mean transfusions from text **Author's name:** MA Claeys **Institution:** Universitair Ziekenhuis Brussel **Email:** MarieAnne.Claeys@uzbrussel.be **Address:** Department of Anaesthesiology, Universitair Ziekenhuis, Brussel, Laarbeeklaan 101B‐1090 Brussels, Belgium **Native language of paper:** English **Reference type:** full text (1) **Trial registration number:** not applicable, but when checked, trial registration number not found **Was it translated for this review:** no	
***Risk of bias***		
**Bias**	**Authors' judgement**	**Support for judgement**
Random sequence generation (selection bias)	Unclear risk	Quote: "The patients were randomly allocated into two groups" Judgement comment: insufficient information to permit judgement.
Allocation concealment (selection bias)	Unclear risk	Quote: not applicable Judgement comment: insufficient information to permit judgement.
Blinding of participants and personnel (performance bias) Subjective outcomes	Unclear risk	Quote: not applicable Judgement comment: no description of personnel blinding given.
Blinding of participants and personnel (performance bias) Objective outcomes	Low risk	Quote: not applicable Judgement comment: objective outcome for personnel, so low risk of bias regardless of quality of blinding.
Blinding of outcome assessment (detection bias) Subjective outcomes	Unclear risk	Quote: not applicable Judgement comment: no description of outcome assessor blinding given.
Blinding of outcome assessment (detection bias) Objective outcomes	Low risk	Quote: not applicable Judgement comment: objective outcome for outcome assessor, so low risk of bias regardless of quality of blinding.
Incomplete outcome data (attrition bias) All outcomes	Low risk	Quote: "Compression ultrasonography on the 10 th postoperative day was positive in 3 patients in the TA group (17 patients investigated) and negative in all patients in the placebo group (18 patients investigated). Post‐ operative doppler examination was not carried out in 5 of the 40 patients (12.5%), because these 5 patients refused the scheduled compression ultrasonography of their lower extremities." Judgement comment: missing outcome data appropriately accounted for.
Selective reporting (reporting bias)	Unclear risk	Quote: not applicable Judgement comment: no protocol to check pre‐specified outcomes were reported.
Other bias	Unclear risk	Quote: not applicable Judgement comment: no funding source declared.

**Table:** Clave 2019

***Study characteristics***		
Methods	**Study design:** RCT **Intention‐to‐treat analysis:** patients were analysed by both ITT and per protocol **Duration of study:** 22 months (19 months + 3 months (FU)) **Power calculation reached:** yes **Transfusion strategy:** transfusions were undertaken in accordance with the French guidelines (Société Française d’Anesthésie Réanimation): 15 autologous packed red blood cell (PRBC) transfusions were given when the level of Hb fell to < 7 g/dL in patients with no relevant history; to between 8 g/dL and 9 g/dL in those with a cardiovascular history; and to < 10 g/dL in those clinically intolerant of lower Hb levels, or with coronary heart disease or proven cardiac insufficiency. **Was the trial stopped early:** no	
Participants	**Baseline characteristics** Placebo *Age (years) (mean SD)*: 64.4 (11.64) *Ethnicity*: not reported *Gender (males, females)*: 33/75 M (44%), 42/75 F (56%) *Length of surgery (minutes) (mean SD)*: 78.9 (19.80) *Proportion of participants on anticoagulants prior to surgery (n/N, %)*: 3 (4.1) *Incidence of preoperative anaemia (n/N, %)*: not reported *Co‐morbidities (n/N, %)*: not reported *ASA 1 (n/N, %)*: not reported *ASA 2 (n/N, %)*: not reported *ASA 3 (n/N, %)*: not reported *ASA 4 (n/N, %)*: not reported *Number of participants randomised*: 75 *Number of participants receiving treatment*: 75 *Number of participants analysed*: 70 *Dropout rate*: 5/75, 6.67% Short TXA, IV *Age (years) (mean SD)*: 65.0 (11.99) *Ethnicity*: not reported *Gender (males, females)*: 31/76 M (41%); 45/76 F (59%) *Length of surgery (minutes) (mean SD)*: 78.3 (20.23) *Proportion of participants on anticoagulants prior to surgery (n/N, %)*: 7 (9.5) *Incidence of preoperative anaemia (n/N, %)*: not reported *Co‐morbidities (n/N, %)*: not reported *ASA 1 (n/N, %)*: not reported *ASA 2 (n/N, %)*: not reported *ASA 3 (n/N, %)*: not reported *ASA 4 (n/N, %)*: not reported *Number of participants randomised*: 76 *Number of participants receiving treatment*: 76 *Number of participants analysed*: 74 *Dropout rate*: 2/76, 2.63% Long TXA, IV *Age (years) (mean SD)*: 67.1 (10.59) *Ethnicity*: not reported *Gender (males, females)*: 34/78 M (44%); 44/78 F (56%) *Length of surgery (minutes) (mean SD)*: 75.7 (18.83) *Proportion of participants on anticoagulants prior to surgery (n/N, %)*: 4 (5.3) *Incidence of preoperative anaemia (n/N, %)*: not reported *Co‐morbidities (n/N, %)*: not reported *ASA 1 (n/N, %)*: not reported *ASA 2 (n/N, %)*: not reported *ASA 3 (n/N, %)*: not reported *ASA 4 (n/N, %)*: not reported *Number of participants randomised*: 78 *Number of participants receiving treatment*: 78 *Number of participants analysed*: 74 *Dropout rate*: 4/78, 5.13% Overall *Age (years) (mean SD)*: not reported *Ethnicity*: not reported *Gender (males, females)*: not reported *Length of surgery (minutes) (mean SD)*: not reported *Proportion of participants on anticoagulants prior to surgery (n/N, %)*: not reported *Incidence of preoperative anaemia (n/N, %)*: not reported *Co‐morbidities (n/N, %)*: not reported *ASA 1 (n/N, %)*: not reported *ASA 2 (n/N, %)*: not reported *ASA 3 (n/N%)*: not reported *ASA 4 (n/N, %)*: not reported *Number of participants randomised*: 229 *Number of participants receiving treatment*: not reported *Number of participants analysed*: not reported *Drop out rate*: not reported **Inclusion criteria:** 1) over 18 years of age; 2) awaiting primary elective THA; 3) scheduled for antithrombotic prophylaxis with rivaroxaban; 4) provided informed consent; and 5) registered in the national social security system **Exclusion criteria:** 1) rapidly destructive osteoarthritis of the hip; 2) previous ipsilateral hip surgery; 3) major contraindications for treatment with TXA, such as epilepsy and renal failure (renal clearance < 30 mL/min); 4) patients already receiving antiplatelet agents (aspirin > 160 mg/h) or anticoagulants; 5) ischaemic arterial disease (myocardial infarction, stroke); 6) previous venous thromboembolism (VTE); 7) contraindication to treatment with rivaroxaban and 8) Child B‐stage cirrhosis with coagulopathy **If TKR, is tourniquet used:** not applicable **Indication for surgery:** not reported **Type of anaesthetic:** general or spinal **Type of surgery:** primary THR	
Interventions	**Intervention characteristics** Placebo 1 g placebo IV at hour 0 followed by 1 g IV at postoperative hours 3, 7 and 11 Placebo, IV, pre‐incision, postop 3, 7 and 11 hours Short TXA, IV Short‐TXA group: 1 g TXA IV at hour 0 followed by 1 g IV at postoperative hour 3, and 1 g of placebo IV at postoperative hours 7 and 11 TXA, IV, 1g, pre‐incision, TXA, IV, 1g postop 3 hours Long TXA, IV Long‐TXA group: 1 g TXA IV at hour 0 (the time of incision), followed by 1 g IV at postoperative hours 3, 7 and 11 TXA, IV, 1 g, pre‐incision, TXA, IV, 1 g postop 3, 7 and 11 hours	
Outcomes	*Primary outcome:* Difference in perioperative real blood loss (RBL) between the baseline level and the level on day 3 (D3) postoperatively *Secondary outcomes:* Haemostatic effects of TXA on the levels of Hb and Hct Need for transfusion Major bleeding was defined as clinically overt bleeding accompanied by one or more of the following: a decrease in the Hb level of > 2 g/dL over a 24‐hour period, transfusion of 2 or more units of PRBCs, bleeding at a critical site (intracranial, intraspinal, intraocular, pericardial, intra‐articular, intramuscular with compartment syndrome or retroperitoneal) or fatal bleeding. Symptomatic thromboembolic events Adjudicated cardiovascular mortality Need for revision surgery All‐cause mortality	
Notes	**Sponsorship source:** pharmaceutical **Country:** France **Setting:** multi‐centre **Comments:** 1) 4 medical centres; 2) reoperation reported but not clear if it was for bleeding **Author's name:** A Clave **Institution:** Brest University Hospital **Email:** arnaud.clave@orange.fr **Address:** Department of Orthopedics and Traumatology, Brest University Hospital, Brest, France **Native language of paper:** English **Reference type:** full text (1), trial registration (1) **Trial registration number:** NCT02403596 **Was it translated for this review:** no	
***Risk of bias***		
**Bias**	**Authors' judgement**	**Support for judgement**
Random sequence generation (selection bias)	Low risk	Quote: "The patients were randomly assigned into three groups of 1:1:1 (using SAS software 9.4; SAS Institute, Cary, North Carolina) by the Centre d’Investigation Clinique de Brest (CIC)." Judgement comment: computer‐generated random number sequence.
Allocation concealment (selection bias)	Low risk	Quote: "Patients were anonymized and given an ID number (centre number/patient index number)." Judgement comment: none
Blinding of participants and personnel (performance bias) Subjective outcomes	Low risk	Quote: "Randomization was blinded and stratified by site." Judgement comment: blinding of personnel considered.
Blinding of participants and personnel (performance bias) Objective outcomes	Low risk	Quote: not applicable Judgement comment: objective outcome for personnel, so low risk of bias regardless of quality of blinding.
Blinding of outcome assessment (detection bias) Subjective outcomes	Low risk	Quote: "The patients, surgeons, and data collectors were blinded to the allocated postoperative protocol" Judgement comment: blinding of surgeons and data collection described.
Blinding of outcome assessment (detection bias) Objective outcomes	Low risk	Quote: "The patients, surgeons, and data collectors were blinded to the allocated postoperative protocol" Judgement comment: objective outcome for outcome assessors, so low risk of bias regardless of quality of blinding.
Incomplete outcome data (attrition bias) All outcomes	Low risk	Quote: not applicable Judgement comment: no obvious outcome data missing.
Selective reporting (reporting bias)	High risk	Quote: not applicable Judgement comment: outcome planned in protocol or prospective trial registry but not reported in the results ‐ all‐cause mortality, transfusion volume in mL, local infection.
Other bias	Unclear risk	Quote: not applicable Judgement comment: trial registration says 231 patients were 'actually' enrolled while paper says 229. Some group differences noted.

**Table:** Colwell 2007

***Study characteristics***		
Methods	**Study design:** RCT **Intention‐to‐treat analysis:** no **Duration of study:** not reported **Power calculation reached:** no **Transfusion strategy:** the only transfusion criteria specified by the study was a haematocrit less than 18% at any time after randomisation when the patient was given whole blood or packed red blood cells (RBCs). No other transfusion criteria were specified; however, even when the haematocrit was greater than 18%, whole blood or packed RBCs were given if deemed necessary based on the patient’s clinical condition and the judgement of the investigator. **Was the trial stopped early:** no	
Participants	**Baseline characteristics** Placebo *Age (at enrolment) (years) (mean SD)*: 64.4 (12.7) *Ethnicity (white) (n/N, %)*: 170/177 (96%) *Gender (males, females) (%)*: 81/177 M (46%); 96/177 F (54%) *Length of surgery (minutes) (mean SD)*: not reported *Proportion of participants on anticoagulants prior to surgery (n/N, %)*: 0/177 (0%) *Incidence of preoperative anaemia (n/N, %)*: 29/177, (16%) *Co‐morbidities (n/N, %)*: not reported *ASA 1 (n/N, %)*: 6/177 (3.39%) *ASA 2 (n/N, %)*: 62/177 (35.02%) *ASA 3 (n/N, %)*: 31/177 (17.51%) *ASA 4 (n/N, %)*: 2/177 (1.13%) *Number of participants randomised*: 179 *Number of participants receiving treatment*: 177 *Number of participants analysed*: 175 *Dropout rate*: 4/179 (2.23%) Aprotinin *Age (at enrolment) (years) (mean SD)*: 63.4 (12.1) *Ethnicity (white) (n/N, %)*: 164/175 (94%) *Gender (males, females) (%)*: 91/175 M (52%); 84/175 F (48%) *Length of surgery (minutes) (mean SD)*: not reported *Proportion of participants on anticoagulants prior to surgery (n/N, %)*: 0/175 (0%) *Incidence of preoperative anaemia (n/N, %)*: not reported *Co‐morbidities (n/N, %)*: not reported *ASA 1 (n/N, %)*: 10/175 (5.71%) *ASA 2 (n/N, %)*: 56/175 (32%) *ASA 3 (n/N, %)*: 33/175 (18.86%) *ASA 4 (n/N, %)*: 1/175 (0.57%) *Number of participants randomised*: 180 *Number of participants receiving treatment*: 176 *Number of participants analysed*: 175 *Dropout rate*: 4/180 (2.22%) Overall *Age (at enrolment) (years) (mean SD)*: not reported *Ethnicity (white) (n/N, %)*: not reported *Gender (males, females) (%)*: not reported *Length of surgery (minutes) (mean SD)*: not reported *Proportion of participants on anticoagulants prior to surgery (n/N, %)*: not reported *Incidence of preoperative anaemia (n/N, %)*: not reported *Co‐morbidities (n/N, %)*: not reported *ASA 1 (n/N, %)*: issue ‐ placebo numbers do not add up to 100% *ASA 2 (n/N, %)*: not reported *ASA 3 (n/N, %)*: not reported *ASA 4 (n/N, %)*: not reported *Number of participants randomised*: not reported *Number of participants receiving treatment*: not reported *Number of participants analysed*: not reported *Dropout rate*: not reported **Inclusion criteria:** 1) at least 18 years of age, 2) elective, unilateral, primary THR **Exclusion criteria:** 1) both hips replaced during the same operation, 2) undergone cardiac surgery using aprotinin within the previous 6 months, 3) known or suspected allergy to aprotinin, 4) impaired renal function (serum creatinine greater than 3.5 mg/dL or 309 mol/L), 5) a history of bleeding diathesis, 6) a known coagulation factor deficiency, 7) a history of deep vein thrombosis (DVT) or pulmonary embolism (PE), 8) a known major organ system failure including renal, or any active noteworthy medical illness that in the opinion of the investigator was likely to affect the patient’s ability to complete the study, 9) refused to receive allogeneic blood products or had low preoperative red blood cell volume (haematocrit less than 22% or haemoglobin values less than 8 g/dL), 10) concomitant chronic treatment with warfarin, 11) planned use of other antifibrinolytic agents such as tranexamic acid, 12) patients chronically treated with clopidogrel were included if treatment was discontinued at least 7 days before surgery, 13) pregnant or breastfeeding women, 14) participation in an investigational drug study within the past 30 days **If TKR, is tourniquet used:** not applicable **Indication for surgery:** not reported **Type of anaesthetic:** mixed (epidural or general) **Type of surgery:** primary THR	
Interventions	**Intervention characteristics** Placebo Treatment with either aprotinin (loading dose of 2 million KIU followed by 0.5 million KIU per hour until the end of surgery) or matching placebo. All patients received a 1 mL (10,000 KIU) test dose of the study drug or placebo to assess the potential forallergic reactions before full study drug administration. Placebo, IV, intraop, loading dose + infusion until end of surgery Aprotinin Treatment with either aprotinin (loading dose of 2 million KIU followed by 0.5 million KIU per hour until the end of surgery) or matching placebo. All patients received a 1 mL (10,000 KIU) test dose of the study drug or placebo to assess the potential for allergic reactions before full study drug administration. Aprotinin, IV, intraop, 2 million KIU loading dose + 0.5 million KIU infusion until end of surgery	
Outcomes	*Primary outcome:* Percentage of patients requiring a blood transfusion (whole blood or packed RBCs, autologous or allogeneic) any time during the intraoperative or postoperative period up to the earlier of 7 days or discharge. *Secondary outcomes:* Percentage of patients receiving an allogeneic transfusion of blood or packed RBCs during surgery and up to the earlier of day 7 or discharge The number of units of whole blood or packed RBCs transfused The number of units of blood or packed RBCs transfused per patient requiring transfusion Estimated blood loss during surgery, drainage from the operative site during the first 6 hours postoperatively, and total drainage until removal of drains	
Notes	**Sponsorship source:** pharmaceutical **Country:** USA **Setting:** multi‐centre (8 centres in Canada and 20 centres in the United States) **Comments**: none **Author's name:** CW Clifford **Institution:** Scripps Clinic **Email:** colwell@scripps.edu; hardwick.mary@scrippshealth.org **Address:** Shiley Center for Orthopaedic Research and Education at Scripps Clinic, 11025 North Torrey Pines Road, Suite 140, La Jolla, CA 92037 **Native language of paper:** English **Reference type:** full text (1) abstract (2) **Trial registration number:** not applicable, but when checked, trial registration number not found **Was it translated for this review:** no	
***Risk of bias***		
**Bias**	**Authors' judgement**	**Support for judgement**
Random sequence generation (selection bias)	Low risk	Quote: "Patients were randomly assigned on the day of surgery to a treatment group in a 1:1 ratio from a computer‐generated list managed by an interactive voice response system." Judgement comment: computer‐generated randomisation.
Allocation concealment (selection bias)	Low risk	Quote: not applicable Judgement comment: aprotinin and placebo were provided to the pharmacy in the same packaging and were dispensed by the randomisation assignment, blinding the patient and staff to the actual treatment group.
Blinding of participants and personnel (performance bias) Subjective outcomes	Low risk	Quote: "blinding the patient and staff to the actual treatment group." Judgement comment: blinding of key personnel described. Identical packaging issued by pharmacy.
Blinding of participants and personnel (performance bias) Objective outcomes	Low risk	Quote: not applicable Judgement comment: objective outcome for personnel, so low risk of bias regardless of quality of blinding.
Blinding of outcome assessment (detection bias) Subjective outcomes	Unclear risk	Quote: not applicable Judgement comment: blinding of outcome assessor not described.
Blinding of outcome assessment (detection bias) Objective outcomes	Low risk	Quote: not applicable Judgement comment: blinding of outcome assessor not described.
Incomplete outcome data (attrition bias) All outcomes	Unclear risk	Quote: not applicable Judgement comment: reason for withdrawal by investigator not explained.
Selective reporting (reporting bias)	High risk	Quote: not applicable Judgement comment: one or more outcome measures are reported incompletely such that they cannot be entered into the meta‐analysis.
Other bias	High risk	Quote: not applicable Judgement comment: appears from the consort diagram per protocol analysis undertaken.

**Table:** Compostella 1997

***Study characteristics***		
Methods	**Study design:** RCT **Intention‐to‐treat analysis:** unclear **Duration of study:** not reported **Power calculation reached:** not reported **Transfusion strategy:** the patients were transfused if the Hct value was less than 27% postoperatively **Was the trial stopped early: n**o	
Participants	**Baseline characteristics** Placebo *Age (years) (median)*: 67 *Ethnicity*: not reported *Gender (males)*: not reported *Length of surgery (minutes) (mean SD)*: not reported *Proportion of participants on anticoagulants prior to surgery (n/N, %)*: not reported *Incidence of preoperative anaemia (n/N, %)*: not reported *Co‐morbidities (n/N, %)*: not reported *ASA 1 (n/N, %)*: not reported *ASA 2 (n/N, %)*: not reported *ASA 3 (n/N, %)*: not reported *ASA 4 (n/N, %)*: not reported *Number of participants randomised*: 50 *Number of participants receiving treatment*: not reported *Number of participants analysed*: not reported *Dropout rate*: not reported Aprotinin *Age (years) (median)*: 68 *Ethnicity*: not reported *Gender (males)*: not reported *Length of surgery (minutes) (mean SD)*: not reported *Proportion of participants on anticoagulants prior to surgery (n/N, %)*: not reported *Incidence of preoperative anaemia (n/N, %): n*ot reported *Co‐morbidities (n/N, %)*: not reported *ASA 1 (n/N, %)*: not reported *ASA 2 (n/N, %)*: not reported *ASA 3 (n/N%)*: not reported *ASA 4 (n/N, %)*: not reported *Number of participants randomised*: 50 *Number of participants receiving treatment*: not reported *Number of participants analysed*: not reported *Dropout rate*: not reported Overall *Age (years) (median)*: not reported *Ethnicity*: not reported *Gender (males)*: not reported *Length of surgery (minutes) (mean SD)*: not reported *Proportion of participants on anticoagulants prior to surgery (n/N, %)*: not reported *Incidence of preoperative anaemia (n/N, %)*: not reported *Co‐morbidities (n/N, %)*: not reported *ASA 1 (n/N, %)*: not reported *ASA 2 (n/N, %)*: not reported *ASA 3 (n/N, %)*: not reported *ASA 4 (n/N, %)*: not reported *Number of participants randomised*: not reported *Number of participants receiving treatment*: not reported *Number of participants analysed*: not reported *Dropout rate*: not reported **Inclusion criteria:** 1) patients undergoing elective cementless THR **Exclusion criteria:** 1) severely impaired coagulation and renal function **If TKR, is tourniquet used:** not applicable **Indication for surgery:** not reported **Type of anaesthetic:** spinal anaesthetic **Type of surgery:** primary THR	
Interventions	**Intervention characteristics** Placebo Saline group (infusion of saline) Placebo, IV, infusion Aprotinin Aprotinin group (loading dose of 2,000,000 KIU followed by 500,000 KIU/h) Aprotinin, IV, 2^6 KIU loading dose + 5^6 KIU infusion	
Outcomes	*Primary outcomes:* Blood loss Need for transfusion	
Notes	**Sponsorship source:** not reported **Country:** Italy **Setting:** not reported **Comments**: none **Author's name:** FA Compostella **Institution:** S. Camillo de Lellis Hospital **Email:** not reported **Address:** Department of Anaesthesia, S. Camillo de Lellis Hospital, Schio, Italy **Native language of paper:** English **Reference type:** abstract (1) **Trial registration number:** not applicable, but when checked, trial registration number not found **Was it translated for this review:** no	
***Risk of bias***		
**Bias**	**Authors' judgement**	**Support for judgement**
Random sequence generation (selection bias)	Unclear risk	Quote: "randomly allocated" Judgement comment: insufficient information to permit judgement.
Allocation concealment (selection bias)	Unclear risk	Quote: not applicable Judgement comment: insufficient information to permit judgement.
Blinding of participants and personnel (performance bias) Subjective outcomes	High risk	Quote: not applicable Judgement comment: intervention arms not identical (loading dose and infusion) therefore unlikely personnel were blinded.
Blinding of participants and personnel (performance bias) Objective outcomes	Low risk	Quote: not applicable Judgement comment: objective outcome for personnel, so low risk of bias regardless of quality of blinding.
Blinding of outcome assessment (detection bias) Subjective outcomes	Unclear risk	Quote: Not applicable Judgement comment: insufficient information to permit judgement.
Blinding of outcome assessment (detection bias) Objective outcomes	Low risk	Quote: not applicable Judgement comment: objective outcome for outcome assessor, so low risk of bias regardless of quality of blinding.
Incomplete outcome data (attrition bias) All outcomes	Unclear risk	Quote: not applicable Judgement comment: patients (receiving treatment and analysed) unclear. Patients' demographic data not reported. No reporting of whether any data are missing.
Selective reporting (reporting bias)	Unclear risk	Quote: not applicable Judgement comment: no protocol to check pre‐specified outcomes reported.
Other bias	Unclear risk	Quote: not applicable Judgement comment: unclear as to how many patients received treatment and were analysed. Insufficient evidence to permit judgement.

**Table:** Cui 2019

***Study characteristics***		
Methods	**Study design:** RCT **Intention‐to‐treat analysis:** unclear **Duration of study:** 3 months (December 2017 and March 2018) + 3 months follow‐up = 6 months **Power calculation reached?:** not reported **Transfusion strategy:** patients received blood transfusion if they had haemoglobin < 70 g/L, or haemoglobin 70 ~ 100 g/L, but with symptoms of anaemia, such as dizziness, palpitation and fatigue **Was the trial stopped early:** no	
Participants	**Baseline characteristics** TXA, IV 20 mg/kg *Age (years) (range)*: 25 to 75 years old, average age: 56.4 *Ethnicity*: not reported *Gender (males, females)*: 16/36 M (44.4%), 20/36 F (55.5%) *Length of surgery (minutes) (mean SD)*: not reported *Proportion of participants on anticoagulants prior to surgery (n/N, %)*: not reported *Incidence of preoperative anaemia (n/N, %)*: patients excluded *Co‐morbidities (n/N, %)*: hypertension 12/36 (33.3%), diabetes 2/36 (5.6%) *ASA 1 (n/N, %)*: not reported *ASA 2 (n/N, %)*: not reported *ASA 3 (n/N, %)*: not reported *ASA 4 (n/N, %)*: not reported *Number of participants randomised*: 36 *Number of participants receiving treatment*: not reported *Number of participants analysed*: not reported *Dropout rate*: not reported TXA, IV 40 mg/kg *Age (years) (range)*: 28 to 79 years old, average age: 53.9 *Ethnicity*: not reported *Gender (males, females)*: 19/36 M (52.7%), 17/36 F (47.2%) *Length of surgery (minutes) (mean SD)*: not reported *Proportion of participants on anticoagulants prior to surgery (n/N, %)*: not reported *Incidence of preoperative anaemia (n/N, %)*: patients excluded *Co‐morbidities (n/N, %)*: hypertension 9/36 (25%), diabetes 3/36 (8.3%) *ASA 1 (n/N, %)*: not reported *ASA 2 (n/N, %)*: not reported *ASA 3 (n/N, %)*: not reported *ASA 4 (n/N, %)*: not reported *Number of participants randomised*: not reported *Number of participants receiving treatment*: not reported *Number of participants analysed*: not reported *Dropout rate*: not reported Overall *Age (years) (mean SD)*: not reported *Ethnicity*: not reported *Gender (males, females)*: not reported *Length of surgery (minutes) (mean SD)*: not reported *Proportion of participants on anticoagulants prior to surgery (n/N, %)*: not reported *Incidence of preoperative anaemia (n/N, %)*: patients excluded *Co‐morbidities (n/N, %)*: hypertension 21/36 (58.3%), diabetes 5/36 (13.8%) *ASA 1 (n/N, %)*: not reported *ASA 2 (n/N, %)*: not reported *ASA 3 (n/N, %)*: not reported *ASA 4 (n/N, %)*: not reported *Number of participants randomised*: not reported *Number of participants receiving treatment*: not reported *Number of participants analysed*: not reported *Dropout rate*: not reported **Inclusion criteria:** 1) patients anticipating unilateral THA surgery due to terminal‐stage hip joint disease, 2) patients who were willing to participate and provided written informed consent **Exclusion criteria:** 1) patients who had inflammatory joint diseases such as rheumatoid arthritis or ankylosing spondylitis that involved hip joint, 2) patients who had anaemia before surgery, 3) patients with coagulopathy, 4) patients at high risk of thrombosis, including atrial fibrillation and status post cardiac stent or pacemaker insertion, 5) patients who were allergic to TXA **If TKR, is tourniquet used:** "No tourniquet was used during surgery" **Indication for surgery:** terminal‐stage hip joint disease **Type of anaesthetic:** general **Type of surgery:** primary unilateral THA	
Interventions	**Intervention characteristics** TXA, IV 20 mg/kg A single dose of 20 mg/kg TXA was administered intravenously before 5 to 10 minutes of operation in group A. All patients received 5 doses of 1 g TXA at 3, 6, 12, 18 and 24 hours after the first dose. TXA, IV, 20 mg/kg, preop + 1 g, at 3, 6, 12, 18 and 24 hours after first dose TXA, IV 40 mg/kg A single dose of 40 mg/kg TXA was administered intravenously in group B at the same time point. All patients received 5 doses of 1 g TXA at 3, 6, 12, 18 and 24 hours after the first dose. TXA, IV, 40 mg/kg, preop + 1 g, at 3, 6, 12 and 18, 24 hours after first dose	
Outcomes	*Primary outcomes:* Total blood loss Haemoglobin level Fibrin(ogen) degradation products (FDP) and D‐dimer levels C‐reaction protein (CRP) and IL‐6 Venous thrombosis Blood transfusion Pulmonary embolism *Secondary outcomes:* Not reported	
Notes	**Sponsorship source:** non‐pharmaceutical (West China Hospital, Sichuan University) **Country:** China **Setting:** single‐centre **Comments:** none **Author's name:** D Cui **Institution:** West China Hospital, Sichuan University **Email:** corresponding author: F. Pei peifux@126.com **Address:** Department of Orthopedics, West China Hospital, Sichuan University, Chengdu Sichuan, 610041, P.R. China **Native language of paper:** Chinese **Reference type:** full text (1), trial registration (1) **Trial registration number:** ChiCTR‐IOR‐17013861 **Was it translated for this review:** yes	
***Risk of bias***		
**Bias**	**Authors' judgement**	**Support for judgement**
Random sequence generation (selection bias)	Low risk	Quote: not applicable Judgement comment: randomisation was achieved by employing a computer program.
Allocation concealment (selection bias)	Unclear risk	Quote: not applicable Judgement comment: method of allocation concealment not described.
Blinding of participants and personnel (performance bias) Subjective outcomes	Unclear risk	Quote: not applicable Judgement comment: subjective outcome for personnel and unclear risk of bias due to unclear adequacy of blinding. No description given of personnel blinding.
Blinding of participants and personnel (performance bias) Objective outcomes	Low risk	Quote: not applicable Judgement comment: objective outcome for personnel, so low risk of bias regardless of quality of blinding.
Blinding of outcome assessment (detection bias) Subjective outcomes	Unclear risk	Quote: not applicable Judgement comment: subjective outcome for personnel and unclear risk of bias due to unclear adequacy of blinding. No description given of personnel blinding.
Blinding of outcome assessment (detection bias) Objective outcomes	Low risk	Quote: not applicable Judgement comment: objective outcome for personnel, so low risk of bias regardless of quality of blinding.
Incomplete outcome data (attrition bias) All outcomes	Unclear risk	Quote: not applicable Judgement comment: insufficient reporting of attrition/exclusions to permit judgement of ‘low risk’ or ‘high risk’.
Selective reporting (reporting bias)	High risk	Quote: not applicable Judgement comment: not all outcomes reported in paper compared to trial registration: range of motion, length of hospital stay, swelling ratio.
Other bias	Unclear risk	Quote: not applicable Judgement comment: insufficient information to assess whether an important risk of bias exists.

**Table:** D'Ambrosio 1999

***Study characteristics***		
Methods	**Study design:** RCT **Intention‐to‐treat analysis:** yes **Duration of study:** not reported **Power calculation reached:** not reported **Transfusion strategy:** not reported **Was the trial stopped early:** no	
Participants	**Baseline characteristics** General anaesthesia + aprotinin *Age (years) (mean SD)*: 66.6 (9.2) *Ethnicity*: not reported *Gender (males, females)*: 7/15 M (47%); 8/15 F (53%) *Length of surgery (minutes) (mean SD)*: 130.6 (42.8) *Proportion of participants on anticoagulants prior to surgery (n/N, %)*: nil *Incidence of preoperative anaemia (n/N, %)*: not reported *Co‐morbidities (n/N, %)*: not reported *ASA 1 (n/N, %)*: not reported *ASA 2 (n/N, %)*: not reported *ASA 3 (n/N, %)*: not reported *ASA 4 (n/N, %)*: not reported *Number of participants randomised*: 15 *Number of participants receiving treatment*: 15 *Number of participants analysed*: 15 *Dropout rate*: 0 General anaesthesia + placebo *Age (years) (mean SD)*: 60.5 (12.9) *Ethnicity*: not reported *Gender (males, females)*: 7/15 M (47%); 8/15 F (53%) *Length of surgery (minutes) (mean SD)*: 139.3 (39.0) *Proportion of participants on anticoagulants prior to surgery (n/N, %)*: nil *Incidence of preoperative anaemia* 7/15 M (47%); 8/15 F (53%): not reported *Co‐morbidities* 7/15 M (47%); 8/15 F (53%): not reported *ASA 1 (n/N, %)*: not reported *ASA 2 (n/N, %)*: not reported *ASA 3 (n/N, %)*: not reported *ASA 4 (n/N, %)*: not reported *Number of participants randomised* : 15 *Number of participants receiving treatment*: 15 *Number of participants analysed* : 15 *Dropout rate*: 0 Overall *Age (years) (mean SD)*: not reported *Ethnicity*: not reported *Gender (males, females)*: not reported *Length of surgery (minutes) (mean SD)*: not reported *Proportion of participants on anticoagulants prior to surgery (n/N, %)*: not reported *Incidence of preoperative anaemia* 7/15 M (47%); 8/15 F (53%): not reported *Co‐morbidities* 7/15 M (47%); 8/15 F (53%): not reported *ASA 1 (n/N, %)*: not reported *ASA 2 (n/N, %)*: not reported *ASA 3 (n/N, %)*: not reported *ASA 4 (n/N, %)*: not reported *Number of participants randomised*: not reported *Number of participants receiving treatment*: not reported *Number of participants analysed*: not reported *Dropout rate*: not reported **Inclusion criteria:** 1) patients scheduled to undergo primary total hip arthroplasty by thesame surgical and anaesthesiological team, 2) ASA groups 1‐2. **Exclusion criteria:** 1) severe cardiorespiratory, 2) coagulation diseases, 3) therapy that could influence bleeding **If TKR, is tourniquet used:** not applicable **Indication for surgery:** not reported **Type of anaesthetic:** general **Type of surgery:** primary THR	
Interventions	**Intervention characteristics** General anaesthesia + aprotinin Aprotinin was administered to groups A and C: 500,000 KIU in bolus form before the surgical incision and 500,000 KIU/h in drip form until the skin was sutured Aprotinin, IV, 0.5^6 KIU, intraop bolus (before incision) + intraop infusion 0.5^6 until end of surgery General anaesthesia + placebo Groups B and D received a placebo (saline solution 0.9%) in the same way and amount as aprotinin Placebo, IV, intraop bolus (before incision) + intraop infusion until end of surgery	
Outcomes	*Primary outcome:* Intra and postoperative bleeding *Secondary outcome:* Not reported	
Notes	**Sponsorship source:** not reported **Country:** Italy **Setting:** single‐centre **Comments:** extracted data from groups C + D only (difference between C + D and A + B being spinal or general, not intervention type or regimen). Mean and SD worked out from paper numbers. **Author's name:** A D'Ambrosio **Institution:** Casa Sollievo Della Sofferenza Hospital **Email:** bborghi@ior.it **Address:** 1st Service of Anaesthesia and Intensive Care, I.R.C.C.S. "Casa Sollievo Della Sofferenza" Hospital, S. Giovanni Rotondo (Foggia) **Native language of paper:** English **Reference type:** full text (1) **Trial registration number:** not applicable, but when checked, trial registration number not found **Was it translated for this review:** no	
***Risk of bias***		
**Bias**	**Authors' judgement**	**Support for judgement**
Random sequence generation (selection bias)	Unclear risk	Quote: "The patients were randomly divided into four groups of 15'' Judgement comment: insufficient information to permit judgement.
Allocation concealment (selection bias)	Unclear risk	Quote: not applicable Judgement comment: no information on allocation concealment.
Blinding of participants and personnel (performance bias) Subjective outcomes	Unclear risk	Quote: "Sixty consecutive patients were included in this prospective and double‐blind study." Judgement comment: no description given of personnel blinding.
Blinding of participants and personnel (performance bias) Objective outcomes	Low risk	Quote: not applicable Judgement comment: objective outcome for personnel, so low risk of bias regardless of quality of blinding.
Blinding of outcome assessment (detection bias) Subjective outcomes	Unclear risk	Quote: not applicable Judgement comment: no description given of outcome assessor blinding.
Blinding of outcome assessment (detection bias) Objective outcomes	Low risk	Quote: not applicable Judgement comment: objective outcome for personnel, so low risk of bias regardless of quality of blinding.
Incomplete outcome data (attrition bias) All outcomes	Unclear risk	Quote: not applicable Judgement comment: insufficient information to permit judgement.
Selective reporting (reporting bias)	Unclear risk	Quote: "None of the patients in any of the four groups had noteworthy complications." Judgement comment: P values not reported, instead "not significant" reported. Complications not specified.
Other bias	Unclear risk	Quote: not applicable Judgement comment: source of funding unclear. Insufficient information to permit judgement.

**Table:** Dorji 2021

***Study characteristics***		
Methods	**Study design:** randomised controlled trial **Intention‐to‐treat analysis:** per protocol **Duration of study:** 15 months (June 2018 and September 2019) + 15 days (follow‐up) **Power calculation reached:** yes **Transfusion strategy:** apart from the routine postoperative care, transfusion of blood was given to patients whose haemoglobin levels were < 8.0 g/dL or to patients with haemoglobin level < 9.0 g/dL with the symptoms of anaemia like increase in heart rates (tachycardia), light headedness, shortness of breath **Was the trial stopped early:** no	
Participants	**Baseline s haracteristics** TXA, IV + TXA, IA *Age (years) (mean SD)*: 66.28 (5.67) *Ethnicity*: not reported *Gender (males, females)*: 11/14 M (78.57%), 3/14 F (21.43%) *Length of surgery (minutes) (mean SD)*: not reported *Proportion of participants on anticoagulants prior to surgery (n/N, %)*: not reported *Incidence of preoperative anaemia (n/N, %)*: not reported *Co‐morbidities (n/N, %)*: not reported *ASA 1 (n/N, %)*: not reported *ASA 2 (n/N, %)*: not reported *ASA 3 (n/N, %)*: not reported *ASA 4 (n/N, %)*: not reported *Number of participants randomised*: not reported *Number of participants receiving treatment*: 14 *Number of participants analysed*: 14 *Dropout rate*: not reported TXA, IA *Age (years) (mean SD)*: 66.4 (5.27) *Ethnicity*: not reported *Gender (males, females)*: 9/17 M (52.94%), 8/17 F (47.06%) *Length of surgery (minutes) (mean SD)*: not reported *Proportion of participants on anticoagulants prior to surgery (n/N, %)*: not reported *Incidence of preoperative anaemia (n/N, %)*: not reported *Co‐morbidities (n/N, %)*: not reported *ASA 1 (n/N, %)*: not reported *ASA 2 (n/N, %)*: not reported *ASA 3 (n/N, %)*: not reported *ASA 4 (n/N, %)*: not reported *Number of participants randomised*: 17 *Number of participants receiving treatment*: not reported *Number of participants analysed*: 16 *Dropout rate*: 1/17, 5.9% Overall *Age (years) (mean SD)*: not reported *Ethnicity*: not reported *Gender (males, females)*: not reported *Length of surgery (minutes) (mean SD)*: not reported *Proportion of participants on anticoagulants prior to surgery (n/N, %)*: not reported *Incidence of preoperative anaemia (n/N, %)*: not reported *Co‐morbidities (n/N, %)*: not reported *ASA 1 (n/N, %)*: not reported *ASA 2 (n/N, %)*: not reported *ASA 3 (n/N, %)*: not reported *ASA 4 (n/N, %)*: not reported *Number of participants randomised*: 32 *Number of participants receiving treatment*: 31 *Number of participants analysed*: 31 *Dropout rate: not reported* **Inclusion criteria:** participants undergoing unilateral primary total knee replacement at the tertiary care centre were invited to participate in this study **Exclusion criteria:** participants were then screened for exclusion criteria: allergic to tranexamic acid, known history of thromboembolic diseases, cardiovascular diseases (myocardial infarction or angina), cerebrovascular disease (stroke), pre‐operative significant renal dysfunction and pre‐operative haemoglobin less than 10 g/dL **If TKR, is tourniquet used:** yes (pneumatic tourniquet was used throughout the duration of the surgery in all patients) **Indication for surgery:** osteoarthritis, rheumatoid arthritis **Type of anaesthetic:** spinal **Type of surgery:** unilateral primary TKR	
Interventions	**Intervention characteristics** TXA, IV + TXA, IA Patients in Group A (intervention group) received both intravenous and intra‐articular tranexamic acid: tranexamic acid 1 g of IV injection 15 min before giving skin incision and tranexamic acid 1 g intra‐articular application after the closure of the joint capsule TXA, IV, 1 g, pre‐op + IA, 1 g, intraop TXA, IA Patients under Group B (control group) received only intra‐articular tranexamic acid: tranexamic acid 1 g intra‐articular after joint capsule closure TXA, IA, 1 g, intraop	
Outcomes	*Primary outcomes:* Fall in haemoglobin level Fall in haematocrit Drain output *Secondary outcomes:* Amount of blood loss Need for blood transfusion Complications	
Notes	**Sponsorship source:** none (this research did not receive any specific grant from any funding agencies in the public, commercial or non‐profit sectors) **Country:** India **Setting:** single‐centre **Comments:** none **Author's name:** Y. Dorji **Institution:** Maharashtra University of Health Sciences **Email:** dorjiyeshi5@gmail.com (Y. Dorji) **Address:** Department of Orthopaedics, Armed Forces Medical College, Maharashtra University of Health Sciences, Pune, Maharashtra, India **Native language of paper:** English **Reference type:** full text (1), trial registration (1) **Trial registration number:** CTRI/2018/05/014106 **Was it translated for this review:** no	
***Risk of bias***		
**Bias**	**Authors' judgement**	**Support for judgement**
Random sequence generation (selection bias)	Low risk	Quote: "The subjects were randomly divided into two groups using a lottery system." Judgement comment: adequate method of sequence generation with lottery system
Allocation concealment (selection bias)	Unclear risk	Quote: "This was a single‐blinded study with all subjects being blinded. The patient and the nursing staff during the process of obtaining consent for surgery were only told that tranexamic acid will be while the exact allocation was not revealed." Judgement comment: method of allocation concealment not mentioned in full text but trial registration says case record numbers.
Blinding of participants and personnel (performance bias) Subjective outcomes	High risk	Quote: not applicable Judgement comment: this was a single‐blinded study with all participants being blinded. The patient and the nursing staff during the process of obtaining consent for surgery were only told that tranexamic acid would be used while the exact allocation was not revealed.
Blinding of participants and personnel (performance bias) Objective outcomes	Low risk	Quote: not applicable Judgement comment: objective outcome for personnel, so low risk of bias regardless of quality of blinding.
Blinding of outcome assessment (detection bias) Subjective outcomes	High risk	Quote: not applicable Judgement comment: no blinding of personnel.
Blinding of outcome assessment (detection bias) Objective outcomes	Low risk	Quote: not applicable Judgement comment: objective outcome for outcome assessor, so low risk of bias regardless of quality of blinding.
Incomplete outcome data (attrition bias) All outcomes	Low risk	Quote: not applicable Judgement comment: no obvious outcome data missing.
Selective reporting (reporting bias)	Low risk	Quote: not applicable Judgement comment: all outcomes planned in the protocol or prospective trial registration are reported.
Other bias	High risk	Quote: not applicable Judgement comment: imbalance in study arms ‐ significantly higher proportion of females in the control group.

**Table:** Ekback 2000

***Study characteristics***		
Methods	**Study design:** RCT **Intention‐to‐treat analysis:** yes **Duration of study:** not reported **Power calculation reached:** not reported **Transfusion strategy?:** allogeneic SAGM‐PRBCs was transfused if Hct < 27% after transfusion of the autologous PRBCs **Was the trial stopped early:** no	
Participants	**Baseline characteristics** Placebo *Age (years) (mean SD)*: 65.6 (8.8) *Ethnicity*: not reported *Gender (males, females)*: 11/20 M (55%); 9/20 F (45%) *Length of surgery (minutes) (mean SD)*: not reported *Proportion of participants on anticoagulants prior to surgery (n/N, %)*: not reported *Incidence of preoperative anaemia (n/N, %)*: not reported *Co‐morbidities (n/N, %)*: not reported *ASA 1 (n/N, %)*: not reported *ASA 2 (n/N, %)*: not reported *ASA 3 (n/N, %)*: not reported *ASA 4 (n/N, %)*: not reported *Number of participants randomised*: 20 *Number of participants receiving treatment*: not reported *Number of participants analysed*: 20 *Dropout rate*: 0 TXA *Age (years) (mean SD)*: 66.4 (9.0) *Ethnicity*: not reported *Gender (males, females)*: 9/20 M (45%) 11/20 F (55%) *Length of surgery (minutes) (mean SD)*: not reported *Proportion of participants on anticoagulants prior to surgery (n/N, %)*: not reported *Incidence of preoperative anaemia (n/N, %)*: not reported *Co‐morbidities (n/N, %)*: not reported *ASA 1 (n/N, %)*: not reported *ASA 2 (n/N, %)*: not reported *ASA 3 (n/N, %)*: not reported *ASA 4 (n/N, %)*: not reported *Number of participants randomised*: 20 *Number of participants receiving treatment*: 20 *Number of participants analysed*: 20 *Dropout rate*: 0 Overall *Age (years) (mean SD)*: not reported *Ethnicity*: not reported *Gender (males, females)*: not reported *Length of surgery (minutes) (mean SD)*: not reported *Proportion of participants on anticoagulants prior to surgery? (n/N, %)*: not reported *Incidence of preoperative anaemia (n/N, %)*: not reported *Co‐morbidities (n/N, %)*: not reported *ASA 1 (n/N, %)*: not reported *ASA 2 (n/N, %)*: not reported *ASA 3 (n/N, %)*: not reported *ASA 4 (n/N, %)*: not reported *Number of participants randomised*: not reported *Number of participants receiving treatment*: not reported *Number of participants analysed*: not reported *Dropout rate*: not reported **Inclusion criteria:** 1) patients undergoing THR **Exclusion criteria:** not reported **If TKR, is tourniquet used:** not applicable **Indication for surgery:** not reported **Type of anaesthetic:** spinal‐epidural anaesthetic **Type of surgery:** primary THR	
Interventions	**Intervention characteristics** Placebo Control group got the same treatment but with a placebo drug (physiological saline) Placebo, IV, intraop (prior to surgical incision) AND placebo, IV, infusion (for 10 hours after 1st dose) AND placebo, IV, postop 3 hours after 1st dose TXA First bolus dose of 10 mg/kg of TXA before surgical incision. A continuous infusion of 1.0 mg/kg/h during 10 h was then started immediately after the first bolus dose. A second bolus dose of 10 mg/kg body weight was given 3 h later to counteract potential dilutive effects of IAT (intraoperative autotransfusion) on TXA concentrations in blood. TXA, IV, 10 mg/kg, intraop (prior to surgical incision) AND TXA, IV, 1 mg/kg infusion (for 10 hours after 1st dose) AND TXA, IV, 10 mg/kg, postop 3 hours after 1st dose	
Outcomes	*Primary outcomes:* Bleeding Postoperative drainage Deep vein thrombosis	
Notes	**Sponsorship source:** mixed, non‐pharmaceutical and pharmaceutical **Country:** Sweden **Setting:** not reported **Comments:** none **Author's name:** G Ekback **Institution:** Orebro Medical Center Hospital **Email:** not reported **Address:** Department of Anesthesiology and Intensive Care, Örebro Medical Center Hospital, S‐701 85 Örebro, Sweden **Native language of paper:** English **Reference type:** full text (1) **Trial registration number:** not applicable, but when checked, trial registration number not found **Was it translated for this review:** no	
***Risk of bias***		
**Bias**	**Authors' judgement**	**Support for judgement**
Random sequence generation (selection bias)	Unclear risk	Quote: not applicable Judgement comment: insufficient information to permit judgement.
Allocation concealment (selection bias)	Unclear risk	Quote: not applicable Judgement comment: insufficient information to permit judgement.
Blinding of participants and personnel (performance bias) Subjective outcomes	Unclear risk	Quote: not applicable Judgement comment: no description of blinding given.
Blinding of participants and personnel (performance bias) Objective outcomes	Low risk	Quote: not applicable Judgement comment: objective outcome for personnel, so low risk of bias regardless of quality of blinding.
Blinding of outcome assessment (detection bias) Subjective outcomes	Unclear risk	Quote: not applicable Judgement comment: no description of blinding given.
Blinding of outcome assessment (detection bias) Objective outcomes	Low risk	Quote: not applicable Judgement comment: objective outcome for personnel, so low risk of bias regardless of quality of blinding
Incomplete outcome data (attrition bias) All outcomes	Unclear risk	Quote: not applicable Judgement comment: insufficient information to permit judgement. Insufficient reporting of attrition.
Selective reporting (reporting bias)	Unclear risk	Quote: not applicable Judgement comment: no protocol to check pre‐specified outcomes reported. No n numbers reported in tables.
Other bias	Low risk	Quote: not applicable Judgement comment: paper appears to be free of other bias.

**Table:** Ellis 2001

***Study characteristics***		
Methods	**Study design:** RCT **Intention‐to‐treat analysis:** yes **Duration of study:** not reported (3‐month FU) **Power calculation reached:** not reported **Transfusion strategy:** throughout the postoperative period, a Hct < 27% constituted the postoperative transfusion trigger **Was the trial stopped early:** no	
Participants	**Baseline characteristics** TXA *Age (years) (mean SD)*: 71 (5) *Ethnicity*: not reported *Gender (males, females)*: 8/20 M (40%); 12/20 F (60%) *Length of surgery (minutes) (mean SD)*: 133 (13) *Proportion of participants on anticoagulants prior to surgery (n/N, %)*: 0/20, 0% *Incidence of preoperative anaemia (n/N, %)*: not reported C*o‐morbidities (n/N, %)*: not reported *ASA 1 (n/N, %)*: 4/20, (20%) *ASA 2 (n/N, %)*: 16/20, (80%) *ASA 3 (n/N, %)*: 0/20, (0%) *ASA 4 (n/N, %)*: 0/0, (0%) *Number of participants randomised*: 20 *Number of participants receiving treatment*: 20 *Number of participants analysed*: 20 *Dropout rate*: 0/20, (0%) Desmopressin *Age (years) (mean SD)*: 72 (5) *Ethnicity*: not reported *Gender (males, females)*: 3/20 M (15%), 17/20 F (85%) *Length of surgery (minutes) (mean SD)*: 128 (15) *Proportion of participants on anticoagulants prior to surgery (n/N, %)*: 0/20, 0% *Incidence of preoperative anaemia (n/N, %)*: not reported *Co‐morbidities (n/N, %)*: not reported *ASA 1 (n/N, %)*: 5/20, (25%) *ASA 2 (n/N, %)*: 14/20, (70%) *ASA 3 (n/N, %)*: 1/20, (5%) *ASA 4 (n/N, %)*: 0/0, (0%) *Number of participants randomised*: 20 *Number of participants receiving treatment*: 20 *Number of participants analysed*: 20 *Dropout rate*: 0/20, (0%) Overall *Age (years) (mean SD)*: not reported *Ethnicity*: not reported *Gender (males, females)*: not reported *Length of surgery (minutes) (mean SD)*: not reported *Proportion of participants on anticoagulants prior to surgery (n/N, %)*: not reported *Incidence of preoperative anaemia (n/N, %)*: not reported *Co‐morbidities (n/N, %)*: not reported *ASA 1 (n/N, %)*: not reported *ASA 2 (n/N, %)*: not reported *ASA 3 (n/N, %)*: not reported *ASA 4 (n/N, %)*: not reported *Number of participants randomised*: not reported *Number of participants receiving treatment*: not reported *Number of participants analysed*: not reported *Dropout rate*: not reported **Inclusion criteria:** 1) ASA physical status I‐II, 2) patients undergoing elective TKR **Exclusion criteria:** 1) history of severe ischaemic heart disease (New York Heart Association grade III, IV), 2) chronic renal failure, 3) liver cirrhosis, 4) bleeding disorders or those currently receiving anticoagulant therapy **If TKR, is tourniquet used:** yes **Indication for surgery:** osteoarthritis **Type of anaesthetic:** general anaesthetic **Type of surgery:** primary TKR	
Interventions	**Intervention characteristics** TXA In the TXA group, in the 30 minutes before deflation of the limb tourniquet, an IV bolus dose of 15 mg per kg of TXA was administered. Thereafter, a constant IV infusion of 10 mg per kg per hour was administered until 12 hours after final deflation of the limb tourniquet TXA, IV, 15 mg/kg, intraop (30 mins prior to tourniquet deflation) AND TXA, IV, 10 mg/kg, intraop, infusion for 12 hours after 1st dose Desmopressin In the desmopressin group, in the 30 minutes before deflation of the limb tourniquet, an IV bolus dose of desmopressin, 0.3 mg per kg, was infused. Thereafter, a constant IV infusion of saline was administered until 12 hours after final deflation of the limb tourniquet. Desmopressin, IV, 0.3 mg/kg, intraop (30 mins prior to tourniquet deflation) AND saline, IV, intraop, infusion for 12 hours after 1st dose	
Outcomes	*Primary outcome:* Procoagulant and fibrinolytic systems *Secondaryoutcome:* None reported	
Notes	**Sponsorship source:** not reported **Country:** Israel **Setting:** single‐centre **Comments:** none **Author's name:** E Zohar **Institution:** Meir Hospital **Email:** bdfgls@netvision.net.il **Address:** Department of Anesthesiology and Intensive Care,Meir Hospital, Kfar Saba 44281, Israel **Native language of paper:** English **Reference type:** full text (2) **Trial registration number:** not applicable, but when checked, trial registration number not found **Was it translated for this review:** no	
***Risk of bias***		
**Bias**	**Authors' judgement**	**Support for judgement**
Random sequence generation (selection bias)	Low risk	Quote: "According to a computer‐generated randomization table, patients were assigned to one of two treatment groups." Judgement comment: computer‐generated randomisation.
Allocation concealment (selection bias)	Unclear risk	Quote: not applicable Judgement comment: insufficient information to permit judgement.
Blinding of participants and personnel (performance bias) Subjective outcomes	Unclear risk	Quote: not applicable Judgement comment: insufficient information to permit judgement.
Blinding of participants and personnel (performance bias) Objective outcomes	Low risk	Quote: not applicable Judgement comment: objective outcome for personnel, so low risk of bias regardless of quality of blinding.
Blinding of outcome assessment (detection bias) Subjective outcomes	Low risk	Quote: "The decision to transfuse allogeneic blood was made by an independent observer (Martin Ellis), who was blinded as to the treatment modality." Judgement comment: blinding of outcome assessors described.
Blinding of outcome assessment (detection bias) Objective outcomes	Low risk	Quote: not applicable Judgement comment: objective outcome for outcome assessor, so low risk of bias regardless of quality of blinding.
Incomplete outcome data (attrition bias) All outcomes	Unclear risk	Quote: not applicable Judgement comment: it was mentioned that 3 patients were lost to follow‐up (40 down to 37) but it wasn't explained which arm/arms these 3 patients belonged to. This made outcome reporting difficult as there was no denominator.
Selective reporting (reporting bias)	High risk	Quote: "myocardial infarction, transient ischemic attack, and stroke was recorded." Judgement comment: no results for outcomes listed in methods section. One or more outcomes of interest in the review are reported incompletely such that they cannot be entered in a meta‐analysis.
Other bias	Unclear risk	Quote: not applicable Judgement comment: funding source was not reported.

**Table:** Engel 2001

***Study characteristics***		
Methods	**Study design:** RCT **Intention‐to‐treat analysis:** not reported **Duration of study:** not reported **Power calculation reached:** no **Transfusion strategy:** if the haemoglobin concentration decreased to < 10 g/dL, blood transfusions were administered **Was the trial stopped early:** no	
Participants	**Baseline characteristics** TXA *Age (years) (mean SD)*: 71 (9) *Ethnicity*: not reported *Gender (males, females)*: 4/12 M (33%); 8/12 F (67%) *Length of surgery (minutes) (mean SD)*: 107 (41) *Proportion of participants on anticoagulants prior to surgery (n/N, %)*: 0 *Incidence of preoperative anaemia (n/N, %) (< 130 g/L)*: 1/12 (8.3) *Co‐morbidities (n/N, %)*: not reported *ASA 1 (n/N, %)*: 0, 0 *ASA 2 (n/N, %)*: 7, 58.3 *ASA 3 (n/N, %)*: 5, 41.7 *ASA 4 (n/N, %)*: 0, 0 *Number of participants randomised*: 12 *Number of participants receiving treatment*: 12 *Number of participants analysed*: 12 *Dropout rate*: 0 Aprotinin *Age (years) (mean SD)*: 66 (11) *Ethnicity*: not reported *Gender (males, females)* : 3/12 M (25%); 9/12 F (75%) *Length of surgery (minutes) (mean SD)*: 101 (25) *Proportion of participants on anticoagulants prior to surgery (n/N, %)*: 0, 0 *Incidence of preoperative anaemia (n/N, %) (< 130 g/L)*: 4/12 (33.3) *Co‐morbidities (n/N, %)*: not reported *ASA 1 (n/N, %)*: 1, 8.3 *ASA 2 (n/N, %)*: 4, 33.3 *ASA 3 (n/N, %)*: 7, 58.3 *ASA 4 (n/N, %)*: 0, 0 *Number of participants randomised*: 12 *Number of participants receiving treatment*: 12 *Number of participants analysed*: 12 *Dropout rate*: 0 Overall *Age (years) (mean SD)*: not reported *Ethnicity*: not reported *Gender (males, females)*: not reported *Length of surgery (minutes)*: not reported *Proportion of participants on anticoagulants prior to surgery (n/N, %)*: not reported *Incidence of preoperative anaemia (n/N, %) (< 130 g/L)*: not reported *Co‐morbidities (n/N, %)*: not reported *ASA 1 (n/N, %)*: not reported *ASA 2 (n/N, %)*: not reported *ASA 3 (n/N, %)*: not reported *ASA 4 (n/N, %)*: not reported *Number of participants randomised*: not reported *Number of participants receiving treatment*: not reported *Number of participants analysed*: not reported *Dropout rate*: not reported **Inclusioncriteria:** patients undergoing total knee replacement **Exclusion criteria:** not reported **If TKR, is tourniquet used:** yes **Indication for surgery:** not reported **Type of anaesthetic:** spinal and epidural **Type of surgery:** primary TKR	
Interventions	**Intervention characteristics** TXA The second group received 15 mg/kg tranexamic acid, followed by a repeated dose of 10 mg/kg after 3 h TXA, IV, 15 mg/kg, intraop + 10 mg/kg 3 hours postop Aprotinin 1 million KIU aprotinin immediately before deflating the tourniquet followed by an infusion of 500,000 KIU per hour for4 h. Aprotinin, IV, 1 million KIU, intraop + 500,000 KIU infusion, 4 hours postop	
Outcomes	*Primary outcomes:* Haemoglobin concentration Platelet count Prothrombin time Thromboplastin time Thrombin time	
Notes	**Sponsorship source:** not reported **Country:** Germany **Setting:** not reported **Comments:** none **Author's name:** JM Engel **Institution:** Justus‐Liebig‐University, Giessen, Germany **Email:** Joerg.Engel@chiru.med.uni‐giessen.de **Address:** Department of Anaesthesiology and Intensive Care Medicine, Justus‐Liebig‐University, Rudolf‐Buchheim‐Str. 7, D‐35385 Giessen, Germany **Native language of paper:** English **Reference type:** full text **Trial registration number:** not applicable, but when checked, trial registration number not found **Was it translated for this review:** no	
***Risk of bias***		
**Bias**	**Authors' judgement**	**Support for judgement**
Random sequence generation (selection bias)	Unclear risk	Quote: not applicable Judgement comment: insufficient information to permit judgement.
Allocation concealment (selection bias)	Unclear risk	Quote: not applicable Judgement comment: insufficient information to permit judgement.
Blinding of participants and personnel (performance bias) Subjective outcomes	High risk	Quote: not applicable Judgement comment: interventions had different treatment timings and regimens, therefore personnel administering intervention would be aware of what the participant would be given.
Blinding of participants and personnel (performance bias) Objective outcomes	Low risk	Quote: not applicable Judgement comment: low risk of bias for objective outcomes.
Blinding of outcome assessment (detection bias) Subjective outcomes	High risk	Quote: not applicable Judgement comment: treatment regimen would have been known to the outcome assessors.
Blinding of outcome assessment (detection bias) Objective outcomes	Low risk	Quote: not applicable Judgement comment: objective outcome for assessor, so low risk of bias regardless of quality of blinding.
Incomplete outcome data (attrition bias) All outcomes	Low risk	Quote: not applicable Judgement comment: no missing outcome data.
Selective reporting (reporting bias)	Unclear risk	Quote: not applicable Judgement comment: insufficient information to permit judgement ‐ no protocol available to know whether all outcomes of interest were reported in a pre‐specified way.
Other bias	Unclear risk	Quote: not applicable Judgement Comment: Insufficient information to assess whether an important risk of bias exists; no reporting of funding or sponsorship.

**Table:** Flordal 1992

***Study characteristics***		
Methods	**Study design:** RCT **Intention‐to‐treat analysis:** yes **Duration of study:** not reported **Power calculation reached:** not reported **Transfusion strategy:** not reported **Was the trial stopped early:** no	
Participants	**Baseline characteristics** Placebo *Age (years) (mean SD)*: 68 (9) *Ethnicity*: not reported *Gender (males, females)*: 12/25 M (48%); 13/25 F (52%) *Length of surgery (minutes) (mean SD)*: 104 (20) *Proportion of participants on anticoagulants prior to surgery (n/N, %)*: not reported *Incidence of preoperative anaemia (n/N, %)*: not reported *Co‐morbidities (n/N, %)*: not reported *ASA 1 (n/N, %)*: not reported *ASA 2 (n/N, %)*: not reported *ASA 3 (n/N, %)*: not reported *ASA 4 (n/N, %)*: not reported *Number of participants randomised*: 25 *Number of participants receiving treatment*: 25 *Number of participants analysed*: 25 *Dropout rate*: 0/25, (0%) Desmopressin *Age (years) (mean SD)*: 64 (9) *Ethnicity*: not reported *Gender (males, females)*: 12/25 M (48%); 13/25 F (52%) *Length of surgery (minutes) (mean SD)*: 106 (23) *Proportion of participants on anticoagulants prior to surgery (n/N, %)*: not reported *Incidence of preoperative anaemia (n/N, %)*: not reported *Co‐morbidities (n/N, %)*: not reported *ASA 1 (n/N, %)*: not reported *ASA 2 (n/N, %)* : not reported *ASA 3 (n/N, %)*: not reported *ASA 4 (n/N, %)*: not reported *Number of participants randomised*: 25 *Number of participants receiving treatment*: 25 *Number of participants analysed*: 25 *Dropout rate*: 0/25, (0%) Overall *Age (years) (mean SD)*: not reported *Ethnicity*: not reported *Gender (males, females)*: not reported *Length of surgery (minutes) (mean SD)*: not reported *Proportion of participants on anticoagulants prior to surgery? (n/N, %)*: not reported *Incidence of preoperative anaemia (n/N, %)*: not reported *Co‐morbidities (n/N, %)*: not reported *ASA 1 (n/N, %)*: not reported *ASA 2 (n/N, %)*: not reported *ASA 3 (n/N, %)*: not reported *ASA 4 (n/N, %)*: not reported *Number of participants randomised*: not reported *Number of participants receiving treatment*: not reported *Number of participants analysed*: not reported *Dropout rate*: not reported **Inclusion criteria:** 1) scheduled for total hip replacement **Exclusion criteria:** 1) patients above the age of 80, 2) patients with severe vascular, hepatic or renal disease **If TKR, is tourniquet used:** not applicable **Indication for surgery:** primary and secondary arthrosis **Type of anaesthetic:** spinal anaesthetic **Type of surgery:** primary and revision THR	
Interventions	**Intervention characteristics** Placebo In a double‐blind fashion, each patient received an infusion of placebo or desmopressin (Minirin, Femng, Sweden) 0.3 μg/kg body weight diluted in 50 mL saline at the start of surgery. The same infusion was repeated 6 hours later, on both occasions at an infusion rate of 20 to 30 minutes Placebo, IV, intraop (start of surgery) + placebo, IV, 6 hours after 1st dose Desmopressin Infusion of placebo or desmopressin (Minirin, Femng, Sweden) 0.3 μg/kg body weight diluted in 50 mL saline at the start of surgery. The same infusion was repeated 6 hours later, on both occasions at an infusion rate of 20 to 30 minutes. Desmopressin, IV, 0.3 μg/kg, intraop (start of surgery) AND desmopressin, IV, 0.3 μg/kg 6 hours after 1st dose	
Outcomes	*Primary outcomes:* Blood loss Bleeding time Hb and platelet count Drainage loss Mean total blood loss	
Notes	**Sponsorship source:** non‐pharmaceutical, desmopressin supplied by Ferring AB free of charge **Country:** Sweden **Setting:** not reported **Comments:** none **Author's name:** PA Flordal **Institution:** Danderyd Hospital **Email:** not reported **Address:** Department of Surgery, Danderyd Hospital, S‐182 88 Danderyd, Sweden **Native language of paper:** English **Reference type:** full text (2) **Trial registration number:** not applicable, but when checked, trial registration number not found **Was it translated for this review:** no	
***Risk of bias***		
**Bias**	**Authors' judgement**	**Support for judgement**
Random sequence generation (selection bias)	Unclear risk	Quote: "Stratification was used in the randomization for primary/revision arthroplasties and for cemented/cementless procedures." Judgement comment: insufficient information to permit judgement.
Allocation concealment (selection bias)	Unclear risk	Quote: not applicable Judgement comment: insufficient information to permit judgement.
Blinding of participants and personnel (performance bias) Subjective outcomes	Unclear risk	Quote: not applicable Judgement comment: no description of blinding given.
Blinding of participants and personnel (performance bias) Objective outcomes	Low risk	Quote: not applicable Judgement comment: objective outcome for personnel, so low risk of bias regardless of quality of blinding.
Blinding of outcome assessment (detection bias) Subjective outcomes	Unclear risk	Quote: not applicable Judgement comment: no description of outcome assessor blinding
Blinding of outcome assessment (detection bias) Objective outcomes	Low risk	Quote: not applicable Judgement comment: objective outcome for outcome assessor, so low risk of bias regardless of quality of blinding.
Incomplete outcome data (attrition bias) All outcomes	Low risk	Quote: not applicable Judgement comment: all outcome data presented
Selective reporting (reporting bias)	Unclear risk	Quote: not applicable Judgement comment: no protocol available to assess whether pre‐specified outcomes reported.
Other bias	Unclear risk	Quote: not applicable Judgement comment: insufficient information to permit judgement. Only drug reaction data reported for desmopressin.

**Table:** Garcia Enguita 1998

***Study characteristics***		
Methods	**Study design:** RCT **Intention‐to‐treat analysis:** not reported **Duration of study:** not reported **Power calculation reached:** not reported **Transfusion strategy:** not reported **Was the trial stopped early:** no	
Participants	**Baseline characteristics** Placebo *Age (years) (mean SD)*: not reported *Ethnicity*: not reported *Gender (males, females)*: not reported *Length of surgery (minutes) (mean SD)*: not reported *Proportion of participants on anticoagulants prior to surgery (n/N, %)*: not reported *Incidence of preoperative anaemia (n/N, %)*: not reported *Co‐morbidities (n/N, %)*: not reported *ASA 1 (n/N, %)*: not reported *ASA 2 (n/N, %)*: not reported *ASA 3 (n/N, %)*: not reported *ASA 4 (n/N, %)*: not reported *Number of participants randomised*: 15 *Number of participants receiving treatment*: not reported *Number of participants analysed*: not reported *Dropout rate*: not reported Aprotinin *Age (years) (mean SD)*: not reported *Ethnicity*: not reported *Gender (males, females)*: not reported *Length of surgery (minutes) (mean SD)*: not reported *Proportion of participants on anticoagulants prior to surgery (n/N, %)*: not reported *Incidence of preoperative anaemia (n/N, %)*: not reported *Co‐morbidities (n/N, %)*: not reported *ASA 1 (n/N, %)*: not reported *ASA 2 (n/N, %)*: not reported *ASA 3 (n/N, %)*: not reported *ASA 4 (n/N, %)*: not reported *Number of participants randomised*: 15 *Number of participants receiving treatment*: not reported *Number of participants analysed*: not reported *Dropout rate*: not reported Overall *Age (years) (mean SD)*: not reported *Ethnicity*: not reported *Gender (males, females)*: not reported *Length of surgery (minutes) (mean SD)*: not reported *Proportion of participants on anticoagulants prior to surgery (n/N, %)*: not reported *Incidence of preoperative anaemia (n/N, %)*: not reported *Co‐morbidities (n/N, %)*: not reported *ASA 1 (n/N, %)*: not reported *ASA 2 (n/N, %)*: not reported *ASA 3 (n/N, %)*: not reported *ASA 4 (n/N, %)*: not reported *Number of participants randomised*: not reported *Number of participants receiving treatment*: not reported *Number of participants analysed*: not reported *Dropout rate*: not reported **Inclusion criteria:** patients undergoing elective cementless revision THR or bilateral THR **Exclusion criteria:** impaired coagulation and renal function **If TKR, is tourniquet used:** not applicable **Indication for surgery:** not reported **Type of anaesthetic:** combined epidural and light general anaesthesia **Type of surgery:** mixed, revision THR and bilateral primary THR	
Interventions	**Intervention characteristics** Placebo Before induction of anaesthesia, a test dose of 5 mL of the study drug was administered over 2 minutes followed by a continuous infusion (loading dose 2 million KIU of aprotinin or 200 mL of saline over 30 minutes followed by 0.5 million KIU/h of aprotinin or 50 mL/h of saline for the duration of surgery. Placebo, IV, 200 mL, intraop loading infusion over 30 mins + placebo, IV, 50 mL, intraop, infusion until end of surgery Aprotinin Before induction of anaesthesia, a test dose of 5 mL of the study drug was administered over 2 minutes followed by a continuous infusion (loading dose 2 million KIU of aprotinin or 200 mL of saline over 30 minutes followed by 0.5 million KIU/h of aprotinin or 50 mL/h of saline for the duration of surgery. Aprotinin, IV, 2^6 KIU, intraop, loading dose over 30 mins + aprotinin, IV, 0.5^6 KIU, intraop, infusion until end of surgery	
Outcomes	*Primary outcomes:* Blood loss Need for homologous transfusion *Secondary outcome:* Not reported	
Notes	**Sponsorship source:** not reported **Country:** Spain **Setting:** single‐centre **Comments:** none **Author's name:** MA Garcia‐Enguita **Institution:** Hospital Miguel Servet **Email:** not reported **Address:** Department of Anesthesiology, Hospital Miguel Servet, Zaragoza, Spain **Native language of paper:** English **Reference type:** abstract (1) **Trial registration number:** not applicable, but when checked, trial registration number not found **Was it translated for this review:** no	
***Risk of bias***		
**Bias**	**Authors' judgement**	**Support for judgement**
Random sequence generation (selection bias)	Unclear risk	Quote: "randomly allocated into 2 groups" Judgement comment: insufficient information to permit judgement.
Allocation concealment (selection bias)	Unclear risk	Quote: not applicable Judgement comment: insufficient information to permit judgement.
Blinding of participants and personnel (performance bias) Subjective outcomes	Unclear risk	Quote: not applicable Judgement comment: insufficient information to permit judgement.
Blinding of participants and personnel (performance bias) Objective outcomes	Low risk	Quote: not applicable Judgement comment: objective outcome for personnel, so low risk of bias regardless of quality of blinding.
Blinding of outcome assessment (detection bias) Subjective outcomes	Unclear risk	Quote: not applicable Judgement comment: insufficient information to permit judgement.
Blinding of outcome assessment (detection bias) Objective outcomes	Low risk	Quote: not applicable Judgement comment: objective outcome for outcome assessor, so low risk of bias regardless of quality of blinding.
Incomplete outcome data (attrition bias) All outcomes	Unclear risk	Quote: not applicable Judgement comment: not clear how many patients received treatment or were analysed.
Selective reporting (reporting bias)	High risk	Quote: "Demographic data and duration of surgery were similar in both groups" Judgement comment: the study fails to include results that should be reported.
Other bias	Unclear risk	Quote: not applicable Judgement comment: insufficient information to permit judgement. No source of funding declared. No protocol available to check whether pre‐specified outcomes reported.

**Table:** Garneti 2004

***Study characteristics***		
Methods	**Study design:** RCT **Intention‐to‐treat analysis:** yes **Duration of study:** not reported **Power calculation reached:** not reported **Transfusion strategy:** we established no defined criteria for administering blood transfusion in this trial, and this could be a source of bias. For patients who required transfusion, the median units of blood transfused were the same in the 2 groups. **Was the trial stopped early:** no	
Participants	**Baseline characteristics** Placebo *Age (years) (mean SD)*: 67.6 (11.4) *Ethnicity*: not reported *Gender (males, females)*: not reported *Length of surgery (minutes) (mean SD)*: not reported *Proportion of participants on anticoagulants prior to surgery (n/N, %)*: not reported *Incidence of preoperative anaemia (n/N, %)*: not reported *Co‐morbidities (n/N, %)*: not reported *ASA 1 (n/N, %)*: not reported *ASA 2 (n/N, %)*: not reported *ASA 3 (n/N, %)*: not reported *ASA 4 (n/N, %)*: not reported *Number of participants randomised*: 25 *Number of participants receiving treatment*: 25 *Number of participants analysed*: 25 *Dropout rate*: 0/25 (0%) TXA *Age (years) (mean SD)*: 69.6 (11.99) *Ethnicity*: not reported *Gender (males, females)*: not reported *Length of surgery (minutes) (mean SD)*: not reported *Proportion of participants on anticoagulants prior to surgery (n/N, %)*: not reported *Incidence of preoperative anaemia (n/N, %)*: not reported *Co‐morbidities (n/N, %)*: not reported *ASA 1 (n/N, %)*: not reported *ASA 2 (n/N, %)*: not reported *ASA 3 (n/N, %)*: not reported *ASA 4 (n/N, %)*: not reported *Number of participants randomised*: 25 *Number of participants receiving treatment*: 25 *Number of participants analysed*: 25 *Dropout rate*: 0/25 (0%) Overall *Age (years) (mean SD)*: not reported *Ethnicity*: not reported *Gender (males, females)*: not reported *Length of surgery (minutes)*: not reported *Proportion of participants on anticoagulants prior to surgery (n/N, %)*: not reported *Incidence of preoperative anaemia (n/N, %)*: not reported *Co‐morbidities (n/N, %)*: not reported *ASA 1 (n/N, %)*: not reported *ASA 2 (n/N, %)*: not reported *ASA 3 (n/N, %)*: not reported *ASA 4 (n/N, %)*: not reported *Number of participants randomised*: 50 *Number of participants receiving treatment*: 50 *Number of participants analysed*: 50 *Dropout rate*: 0/50 (0%) **Inclusion criteria:** 1) diagnosis of primary osteoarthritis of the hip necessitating THR **Exclusion criteria:** not reported **Pretreatment:** unclear **If TKR, is tourniquet used:** not applicable **Indication for surgery:** osteoarthritis **Type of anaesthetic:** spinal anaesthetic **Type of surgery:** primary THR	
Interventions	**Intervention characteristics** Placebo 10 mg/kg of intravenous tranexamic acid or a similar volume of normal saline (placebo) as a bolus at anaesthesia. A dose of 10 mg/kg was suggested by the Drug Information Department at Cheltenham General Hospital, after contacting Pharmacia. Placebo, IV, intraop (at anaesthesia) TXA Patients were randomised using a random number technique to receive either 10 mg/kg of intravenous tranexamic acid or a similar volume of normal saline (placebo) as a bolus at anaesthesia. TXA, IV, 10 mg/kg, intraop (at anaesthesia)	
Outcomes	*Primary outcome:* Bleeding from the femoral canal *Secondary outcome:* Not reported	
Notes	**Sponsorship source:** none **Country:** UK **Setting:** single‐centre **Comments:** mean and SD calculated from text **Author's name:** N Garneti **Institution:** Cheltenham General Hospital **Email:** not reported **Address:** 7 Woodlea Gate, Meanwood, Leeds LS6 4SR, United Kingdom **Native language of paper:** English **Reference type:** full text (1) **Trial registration number:** not applicable, but when checked, trial registration number not found **Was it translated for this review:** no	
***Risk of bias***		
**Bias**	**Authors' judgement**	**Support for judgement**
Random sequence generation (selection bias)	Low risk	Quote: "Patients were randomized using a random number technique" Judgement comment: use of random number table.
Allocation concealment (selection bias)	Unclear risk	Quote: not applicable Judgement comment: insufficient information to permit judgement.
Blinding of participants and personnel (performance bias) Subjective outcomes	Low risk	Quote: "The patient, anesthetist, and surgeon were all unaware of which solution was given." Judgement comment: description of blinding given and unlikely blinding could have been broken.
Blinding of participants and personnel (performance bias) Objective outcomes	Low risk	Quote: not applicable Judgement comment: objective outcome for personnel, so low risk of bias regardless of quality of blinding.
Blinding of outcome assessment (detection bias) Subjective outcomes	Unclear risk	Quote: not applicable Judgement comment: no description of outcome assessor blinding given.
Blinding of outcome assessment (detection bias) Objective outcomes	Low risk	Quote: not applicable Judgement comment: objective outcome for outcome assessor, so low risk of bias regardless of quality of blinding.
Incomplete outcome data (attrition bias) All outcomes	Unclear risk	Quote: not applicable Judgement comment: insufficient reporting of attrition to permit judgement. No n numbers in outcome tables.
Selective reporting (reporting bias)	Unclear risk	Quote: not applicable Judgement comment: no protocol to check pre‐specified outcome measures reported.
Other bias	Unclear risk	Quote: "Perhaps this was because of the different transfusion strategies of the anesthetists, one of whom transfused most patients unless they were young and healthy." Judgement comment: no reporting of key population baseline characteristics such as gender, which would be expected. No transfusion protocol and varying thresholds between outcome assessors.

**Table:** Georgiadis 2013

***Study characteristics***		
Methods	**Study design:** RCT **Intention‐to‐treat analysis:** yes **Duration of study:** 16.5 months **Power calculation reached:** not reported **Transfusion strategy:** patients were transfused for symptomatic anaemia (defined as light‐headedness, presyncope, fatigue precluding participation in therapy, palpitations or shortness of breath not due to other causes) with haemoglobin (Hb) of 8.0 g/dL or any Hb below 7.0 g/dL **Was the trial stopped early:** no	
Participants	**Baseline characteristics** Placebo *Age (years) (mean SD)*: 64.5 (8.2) *Ethnicity*: not reported *Gender (males, females)*: 12/51 M (24%), 39/51 F (76%) *Length of surgery (minutes)*: not reported *Proportion of participants on anticoagulants prior to surgery (n/N, %)*: 0/51 (0%) *Incidence of preoperative anaemia (n/N, %)*: not reported *Co‐morbidities (n/N, %)*: not reported *ASA 1 (n/N, %)*: not reported *ASA 2 (n/N, %)*: not reported *ASA 3 (n/N, %)*: not reported *ASA 4 (n/N, %)*: not reported *Number of participants randomised*: 51 *Number of participants receiving treatment*: 51 *Number of participants analysed*: 51 *Dropout rate*: 0/51 (0%) TXA *Age (years) (mean SD)*: 67.0 (9.0) *Ethnicity*: not reported *Gender (males, females)*: 19/50 M (38%), 31/50 F (62%) *Length of surgery (minutes) (mean SD)*: not reported *Proportion of participants on anticoagulants prior to surgery (n/N, %)*: 0/50 (0%) *Incidence of preoperative anaemia (n/N, %)*: not reported *Co‐morbidities (n/N, %)*: not reported *ASA 1 (n/N, %)*: not reported *ASA 2 (n/N, %)*: not reported *ASA 3 (n/N, %)*: not reported *ASA 4 (n/N, %)*: not reported *Number of participants randomised*: 50 *Number of participants receiving treatment*: 50 *Number of participants analysed*: 50 *Dropout rate*: 0/51 (0%) Overall *Age (years) (mean SD)*: not reported *Ethnicity*: not reported *Gender (males, females)*: not reported *Length of surgery (minutes) (mean SD)*: not reported *Proportion of participants on anticoagulants prior to surgery? (n/N, %)*: not reported *Incidence of preoperative anaemia (n/N, %)*: not reported *Co‐morbidities (n/N, %)*: not reported *ASA 1 (n/N, %)*: not reported *ASA 2 (n/N, %)*: not reported *ASA 3 (n/N, %)*: not reported *ASA 4 (n/N, %)*: not reported *Number of participants randomised*: not reported *Number of participants receiving treatment*: not reported *Number of participants analysed*: not reported *Dropout rate*: not reported **Inclusion criteria:** 1) all patients undergoing unilateral primary TKA **Exclusion criteria:** 1) religious objection to autologous blood transfusion, 2) preoperative use of anticoagulant medication seven days prior to surgery, 3) history of fibrinolytic disorder or blood dyscrasia, cerebrovascular accident (CVA), myocardial infarction (MI), New York Heart Association class III or IV heart failure (NYHA III‐IV), atrial fibrillation, 4) history of deep vein thrombosis (DVT) or pulmonary embolus (PE), 5) preoperative international normalised ratio (INR) N 1.4, activated partial thromboplastin time (aPTT) N 1.4 × normal, platelets b 140,000/mm^3^, 6) renal failure defined as creatinine N 1.1 mg/dL or glomerular filtration rate b 60 mL/min/1.73 m^2^ **If TKR, is tourniquet used:** yes **Indication for surgery:** not reported **Type of anaesthetic:** mixed spinal (77/101) and general (24/101) anaesthesia balanced across groups **Type of surgery:** primary TKR	
Interventions	**Intervention characteristics** Placebo Placebo solution (75 mL normal saline) (100 mL 0.9% normal saline, applied topically ‐ taken from NCT page) Placebo, IA, intraop (after component placing, prior to tourniquet deflation) TXA Tranexamic acid (2.0 g in 75 mL normal saline) or placebo solution (75 mL normal saline) was sterilely prepared by a non‐affiliated compounding pharmacy with no involvement in patient care and was delivered to the institution’s research pharmacy. 75 mL of TXA or placebo solution was sterilely opened on the back table and applied to the wound. TXA, IA, 2 g, intraop (after component placing, prior to tourniquet deflation)	
Outcomes	*Primary outcome:* Perioperative blood loss *Secondary outcome:* Not reported	
Notes	**Sponsorship source:** not reported **Country:** USA **Setting:** single‐centre **Comments:** mean RBC units and SD worked out from text **Author's name:** AG Georgiadis **Institution:** Rush University Medical Center **Email:** not reported **Address:** Department of Orthopaedic Surgery, Henry Ford Hospital, CFP‐642, 2799 W. Grand Blvd., Detroit, MI 48202 **Native language of paper:** English **Reference type:** full text (1), conference abstract (1), trial registration (1) **Trial registration number:** NCT01370460 **Was it translated for this review:** no	
***Risk of bias***		
**Bias**	**Authors' judgement**	**Support for judgement**
Random sequence generation (selection bias)	Low risk	Quote: "Consented patients were sequentially assigned to a computer‐generated randomization schedule known only to a research pharmacist who had no physician or patient contact." Judgement comment: computer‐generated randomisation.
Allocation concealment (selection bias)	Low risk	Quote: "Tranexamic acid (2.0 g in 75 mL normal saline) or placebo solution (75 mL normal saline) was sterilely prepared by a non‐affiliated compounding pharmacy with no involvement in patient care and was delivered to our institution’s research pharmacy." Judgement comment: centrally allocated by pharmacy.
Blinding of participants and personnel (performance bias) Subjective outcomes	Low risk	Quote: "Consented patients were sequentially assigned to a computer‐generated randomization schedule known only to a research pharmacist who had no physician or patient contact. All physicians, nurses, and operative assistants were blinded to the treatment arm for the duration of the clinical trial." Judgement comment: blinding of key personnel likely ensured.
Blinding of participants and personnel (performance bias) Objective outcomes	Low risk	Quote: not applicable Judgement comment: objective outcome for personnel, so low risk of bias regardless of quality of blinding.
Blinding of outcome assessment (detection bias) Subjective outcomes	Low risk	Quote: "All physicians, nurses, and operative assistants were blinded to the treatment arm for the duration of the clinical trial." Judgement comment: likely blinding of outcome assessors ensured.
Blinding of outcome assessment (detection bias) Objective outcomes	Low risk	Quote: not applicable Judgement comment: objective outcome for outcome assessor, so low risk of bias regardless of quality of blinding.
Incomplete outcome data (attrition bias) All outcomes	Low risk	Quote: not applicable Judgement comment: all outcome data were presented.
Selective reporting (reporting bias)	Low risk	Quote: not applicable Judgement comment: outcomes mentioned were measured and reported. They also match with those reported in the trial registration.
Other bias	Low risk	Quote: not applicable Judgement comment: this paper appears to be free of other bias.

**Table:** Gill 2009

***Study characteristics***		
Methods	**Study design:** RCT **Intention‐to‐treat analysis:** unclear **Duration of study:** 26 months **Power calculation reached:** yes **Transfusion strategy:** the decision to transfuse blood was based on a predetermined laboratory value demonstrating a haemoglobin level of less than 10 g/dL and haematocrit level of less than 30%. These are conservative values outlined by Walsh and McClelland in postsurgical participants. **Was the trial stopped early:** yes	
Participants	**Baseline characteristics** Placebo *Age (years) (mean range)*: 61.4 (36 to 73) *Ethnicity*: not reported *Gender (males, females)*: 2/5 M (40%); 3/5 F (60%) *Length of surgery (minutes)*: 243.4 *Proportion of participants on anticoagulants prior to surgery (n/N, %)*: not reported *Incidence of preoperative anaemia (n/N, %)*: not reported *Co‐morbidities (n/N, %)*: not reported *ASA 1 (n/N, %)*: not reported *ASA 2 (n/N, %)*: not reported *ASA 3 (n/N, %)*: not reported *ASA 4 (n/N, %)*: not reported *Number of participants randomised*: 5 *Number of participants receiving treatment*: 5 *Number of participants analysed*: 5 *Dropout rate*: 0, 0% TXA *Age (years) (mean range)*: 66.6 (53 to 83) *Ethnicity*: not reported *Gender (males, females)*: 1/5 M (20%); 4/5 F (80%) *Length of surgery (minutes)*: 201.4 *Proportion of participants on anticoagulants prior to surgery (n/N, %)*: not reported *Incidence of preoperative anaemia (n/N, %)*: not reported *Co‐morbidities (n/N, %)*: not reported *ASA 1 (n/N, %)*: not reported *ASA 2 (n/N, %)*: not reported *ASA 3 (n/N, %)*: not reported *ASA 4 (n/N, %)*: not reported *Number of participants randomised*: 5 *Number of participants receiving treatment*: 5 *Number of participants analysed*: 5 *Dropout rate*: 0, 0% Overall *Age (years) (mean range)*: not reported *Ethnicity*: not reported *Gender (males, females)*: not reported *Length of surgery (minutes)*: not reported *Proportion of participants on anticoagulants prior to surgery (n/N, %)*: not reported *Incidence of preoperative anaemia (n/N, %)*: not reported *Co‐morbidities (n/N, %)*: not reported *ASA 1 (n/N, %)*: not reported *ASA 2 (n/N, %)*: not reported *ASA 3 (n/N, %)*: not reported *ASA 4 (n/N, %)*: not reported *Number of participants randomised*: not reported *Number of participants receiving treatment*: not reported *Number of participants analysed*: not reported *Dropout rate*: not reported **Inclusion criteria:** 1) findings consistent with the need for revision total hip arthroplasty **Exclusion criteria:** 1) any patients in need of primary total hip arthoplasty or those with a known prosthetic infection, 2) a bleeding or coagulation disorder, 3) renal insufficiency (serum creatinine > 2 standard deviations for age), 4) history of deep venous thrombosis or pulmonary embolism were excluded from this study (theoretical concern of increased risk), 5) prisoners and pregnant patients also were excluded **If TKR, is tourniquet used:** not applicable **Indication for surgery:** not reported **Type of anaesthetic:** not reported **Type of surgery:** revision THR	
Interventions	**Intervention characteristics** Placebo A corresponding volume of normal saline was administered to patients in the placebo group in the same fashion as the tranexamic acid. Placebo, IV, intraop, bolus (at induction) AND placebo, IV, intraop infusion until wound closed TXA Tranexamic acid was administered in the operating room as a 10 mg/kg bolus before the induction of anaesthesia to ensure that there were no drug reactions. This was followed by 1 mg/kg/hr infusion at the start of the surgery until the surgical wound was closed. TXA, IV, 10 mg/kg, intraop, bolus (at induction) AND TXA, IV, 1 mg/kg/hr intraop infusion until wound closed	
Outcomes	*Primary outcome:* Total number of blood transfusions *Secondary outcome:* Incidence of deep venous thrombosis	
Notes	**Sponsorship source:** non‐pharmaeutical **Country:** USA **Setting:** single‐centre **Comments:** none **Author's name:** J Brian Gill **Institution:** Nebraska Foundation for Spinal Research **Email:** jbgill@nebraskaspinecenter.com **Address:** Nebraska Foundation for Spinal Research, 13616 California St, Suite 100, Omaha, NE 68154, USA **Native language of paper:** English **Reference type:** full text (1) **Trial registration number:** not applicable, but when checked, trial registration number not found **Was it translated for this review:** no	
***Risk of bias***		
**Bias**	**Authors' judgement**	**Support for judgement**
Random sequence generation (selection bias)	Low risk	Quote: "Upon being enrolled in the study, the patients were randomized into either the placebo group or the tranexamic acid group coordinated by our School of Pharmacy, using a computer software randomization program." Judgement comment: computer‐generated randomisation.
Allocation concealment (selection bias)	Low risk	Quote: "There were no identifying markers on the bag to whether tranexamic acid or placebo was being used." Quote: "coordinated by our School of Pharmacy," Judgement comment: centrally allocated treatment.
Blinding of participants and personnel (performance bias) Subjective outcomes	Low risk	Quote: "This study was blinded to the attending orthopaedic surgeon, the anesthesiologist and the patient, as only the School of Pharmacy knew whether tranexamic acid or placebo was administered." Judgement comment: blinding of participants and key study personnel ensured, and unlikely that the blinding could have been broken.
Blinding of participants and personnel (performance bias) Objective outcomes	Low risk	Quote: not applicable Judgement comment: objective outcome for personnel, so low risk of bias regardless of quality of blinding.
Blinding of outcome assessment (detection bias) Subjective outcomes	Low risk	Quote: "This study was blinded to the attending orthopaedic surgeon, the anesthe‐ siologist and the patient, as only the School of Pharmacy knew whether tranexamic acid or placebo was administered." Judgement comment: blinding of participants and key study personnel ensured, and unlikely that the blinding could have been broken.
Blinding of outcome assessment (detection bias) Objective outcomes	Low risk	Quote: not applicable Judgement comment: objective outcome for outcome assessor, so low risk of bias regardless of quality of blinding.
Incomplete outcome data (attrition bias) All outcomes	Low risk	Quote: not applicable Judgement comment: all data were presented.
Selective reporting (reporting bias)	Unclear risk	Quote: not applicable Judgement comment: no protocol to compare outcomes
Other bias	High risk	Quote: not applicable Judgement comment: 42% of eligible patients were enrolled, which raises a concern about potential selection bias. The initial power calculation needed 10 patients, however it seems that given the poor recruitment rate the power calculation was redone to allow for 5 patients per arm.

**Table:** Gomez Barrena 2014

***Study characteristics***		
Methods	**Study design:** RCT **Intention‐to‐treat analysis:** yes **Duration of study:** 10 months **Power calculation reached:** yes **Transfusion strategy:** blood transfusion was planned for patients with a haemoglobin level of < 8.0 g/dL who were asymptomatic and appeared healthy. Transfusion was planned for patients with a level of < 10.0 g/dL if they had 1) symptoms that were not well tolerated (including any organ dysfunction), were related to anaemia and were not attributable to another cause (myocardial ischaemia or hypoxaemia), or 2) ongoing blood loss. **Was the trial stopped early:** no	
Participants	**Baseline characteristics** TXA, IV *Age (years) (mean SD)*: 71.8 (10.3) *Ethnicity*: not reported *Gender (males, females)*: 14/39 M (36%); 25/39 F (64%) *Length of surgery (minutes) (mean SD)*: 75.1 (14.1) *Proportion of participants on anticoagulants prior to surgery (n/N, %)*: 0 (0%) *Incidence of preoperative anaemia (n/N, %)*: not reported *Co‐morbidities (n/N, %)*: not reported *ASA 1 (n/N, %)*: 1/39 (3%) *ASA 2 (n/N, %)*: 28/39 (72%) *ASA 3 (n/N, %)*: 10/39 (26%) *ASA 4 (n/N, %)*: 0/39 (0%) *Number of participants randomised*: 39 *Number of participants receiving treatment*: 39 *Number of participants analysed*: 39 *Dropout rate*: 0/39 (0%) TXA, IA *Age (years) (mean SD)*: 70.1 (9.1) *Ethnicity*: not reported *Gender (males, females)*: 13/39 M (33%); 26/39 F (67%) *Length of surgery (minutes) (mean SD)*: 76.4 (15.5) *Proportion of participants on anticoagulants prior to surgery (n/N, %)*: 0 (0%) *Incidence of preoperative anaemia (n/N, %)*: not reported *Co‐morbidities (n/N, %)*: not reported *ASA 1 (n/N, %)*: 3/39 (8%) *ASA 2 (n/N, %)*: 32/39 (82%) *ASA 3 (n/N, %)*: 4/39 (10%) *ASA 4 (n/N, %)*: 0/39 (0%) *Number of participants randomised*: 39 *Number of participants receiving treatment*: 39 *Number of participants analysed*: 39 *Dropout rate*: 0/39 (0%) Overall *Age (years) (mean SD)*: not reported *Ethnicity*: not reported *Gender (males, females)*: not reported *Length of surgery (minutes) (mean SD)*: not reported *Proportion of participants on anticoagulants prior to surgery (n/N, %)*: not reported *Incidence of preoperative anaemia (n/N, %)*: not reported *Co‐morbidities (n/N, %)*: not reported *ASA 1 (n/N, %)*: not reported *ASA 2 (n/N, %)*: not reported *ASA 3 (n/N, %)*: not reported *ASA 4 (n/N, %)*: not reported *Number of participants randomised*: 78 *Number of participants receiving treatment*: 78 *Number of participants analysed*: 78 *Dropout rate*: 0 **Inclusion criteria:** 1) all adult patients scheduled to undergo primary unilateral total knee replacement with cemented implant **Exclusion criteria:** 1) absence of written informed consent, 2) allergy to TXA, 3) major comorbidities (severe ischaemic cardiopathy, sleep apnoea syndrome, severe pulmonary disease, severe renal insufficiency, or hepatic failure), 4) coagulopathy (preoperative platelet count < 150,000/mm^3^, INR > 1.4 or prolonged partial thromboplastin time of > 1.4 times normal), 5) a history of arterial or venous thromboembolic disease (cerebrovascular accident, DVT or pulmonary thromboembolism), 6) a haematologic disorder (a haematopoietic, haemorrhagic or thrombogenic disease), 7) retinopathy (severe vision field limitation and/or colour distortion), 8) refusal of blood products, 9) pregnancy, 10) breastfeeding, 11) participation in another clinical trial during the last year **If TKR, is tourniquet used:** yes **Indication for surgery:** not reported **Type of anaesthetic:** spinal anaesthesia **Type of surgery:** primary TKR	
Interventions	**Intervention characteristics** TXA, IV Patients in the control group received a slow IV infusion of 100 mL of physiological saline solution containing a 15 mg/kg dose of TXA 15 to 20 minutes before tourniquet release and a second identical dose 3 hours after surgery, on the basis of previous efficacy studies. In addition, patients in this group received a topical intra‐articular placebo (100 mL of physiological saline solution). TXA, IV, 15 mg/kg, intraop (15 to 20 mins prior to tourniquet deflation) + placebo, IA, intraop (half on tissue before closure, half on injected after skin closure) + TXA, IV, 15 mg/kg, 3 hours postop TXA, IA The experimental group received a topical intra‐articular dose of 3 g of TXA (Amchafibrin; Rottapharm) in 100 mL of physiological saline solution (0.9% sodium chloride solution; Grifols) on the basis of previous studies that confirmed the high efficacy of this dosage. Half of the volume was administered by irrigation to achieve tissue impregnation before joint closure, and the other half was administered intra‐articularly after skin closure (through a 12 mm drain tube with the knee in a fully extended position after stapling and before tourniquet release). In addition, patients in this group received 100 mL of an IV placebo solution (physiological saline solution) 15 to 20 minutes before tourniquet release and 100 mL 3 hours later. Placebo, IV, intraop (15 to 20 mins prior to tourniquet deflation) + TXA, IA, 3 g, intraop (half on tissue before closure, half on injected after skin closure) + placebo, IV, 3 hours postop	
Outcomes	*Primary outcome*: Postoperative blood transfusion *Secondary outcomes*: Visible blood loss (as measured in the drain) Invisible blood loss (as estimated from the Nadler formula) Complications Severe adverse events Length of stay in the hospital Postoperative changes in active range of motion of the knee (which was measured with a standard clinical goniometer before and 30 days after the operation with the patient in a supine position)	
Notes	**Sponsorship source:** mixed non‐pharmaceutical and pharmaceutical **Country:** Spain **Setting:** single‐centre **Comments:** none **Author's name:** E Gomez‐Barrena **Institution:** La Paz University Hospital **Email:** egomezb@salud.madrid.org, egomezbarrena@gmail.com **Address:** Department of Orthopaedic Surgery and Traumatology, Hospital Universitario “La Paz”‐IdiPAZ‐UAM, Traumatologıa 1ª planta, Paseo de la Castellana 261, 28046 Madrid, Spain **Native language of paper:** English **Reference type:** full text (1) trial registration (1) **Trial registration number:** 2011‐003218‐17 **Was it translated for this review:** no	
***Risk of bias***		
**Bias**	**Authors' judgement**	**Support for judgement**
Random sequence generation (selection bias)	Unclear risk	Quote: "Recruited patients were randomly allocated" Judgement comment: insufficient information to permit judgement.
Allocation concealment (selection bias)	Low risk	Quote: "Patient assignments were prepared by a research statistician and were placed into sequentially numbered opaque sealed envelopes, which were kept by research personnel." Judgement comment: sequentially numbered, opaque, sealed envelopes.
Blinding of participants and personnel (performance bias) Subjective outcomes	Low risk	Quote: "Patients, surgeons, and health‐care personnel participating in treatment and evaluation were blinded to the group allocation throughout the study period." Judgement comment: blinding of key personnel ensured.
Blinding of participants and personnel (performance bias) Objective outcomes	Low risk	Quote: not applicable Judgement comment: objective outcome for personnel, so low risk of bias regardless of quality of blinding.
Blinding of outcome assessment (detection bias) Subjective outcomes	Low risk	Quote: "Patient assignments were prepared by a research statistician and were placed into sequentially numbered opaque sealed envelopes, which were kept by research personnel. An envelope was opened before each surgery, and the appropriate study medication and placebo were prepared under sterile conditions by uninvolved anesthesiologists under the supervision of a research pharmacist not involved in patient care. The study medication and placebo were identical in appearance. Patients, surgeons, and health‐care personnel participating in treatment and evaluation were blinded to the group allocation throughout the study period." Judgement comment: blinding of outcome assessor ensured.
Blinding of outcome assessment (detection bias) Objective outcomes	Low risk	Quote: not applicable Judgement comment: objective outcome for outcome assessors, so low risk of bias regardless of quality of blinding.
Incomplete outcome data (attrition bias) All outcomes	Low risk	Quote: not applicable Judgement comment: no missing data reported.
Selective reporting (reporting bias)	Low risk	Quote: not applicable Judgement comment: all outcomes in trial registrations reported.
Other bias	Low risk	Quote: not applicable Judgement comment: the paper appears to be free of other bias.

**Table:** Gonzalez Osuna 2021

***Study characteristics***		
Methods	**Study design:** randomised controlled trial **Intention‐to‐treat analysis:** no **Duration of study:** recruitment (19 September 2018 to 9 October 2018) + follow‐up (time period not stated) = 3 weeks **Power calculation reached:** not reported **Transfusion strategy:** not reported **Was the trial stopped early:** not reported	
Participants	**Baseline characteristics** TXA, IV *Age (years) (mean SD)*: 73.73 (6.19) *Ethnicity*: not reported *Gender (males, females)*: 2/12 M (16.7%); 10/12 F (83.3%) *Length of surgery (minutes) (mean SD)*: not reported *Proportion of participants on anticoagulants prior to surgery (n, %)*: not reported *Incidence of preoperative anaemia (n/N, %)*: not reported *Co‐morbidities (n/N, %)*: not reported *ASA 1 (n, %)*: not reported *ASA 2 (n, %)*: not reported *ASA 3 (n, %)*: not reported *ASA 4 (n, %)*: not reported *Number of participants randomised*: 13 *Number of participants receiving treatment*: 12 *Number of participants analysed*: 12 *Drop out rate*: 1/13, 7.7% TXA, IA *Age (years) (mean SD)*: 73.71 (5.17) *Ethnicity*: not reported *Gender (males, females)*: 2/12 M (16.7%); 10/12 F (83.3%) *Length of surgery (minutes) (mean SD)*: not reported *Proportion of participants on anticoagulants prior to surgery (n, %)*: not reported *Incidence of preoperative anaemia (n/N, %)*: not reported *Co‐morbidities (n/N, %)*: not reported *ASA 1 (n, %)*: not reported *ASA 2 (n, %)*: not reported *ASA 3 (n, %)*: not reported *ASA 4 (n, %)*: not reported *Number of participants randomised*: 15 *Number of participants receiving treatment*: 13 *Number of participants analysed*: 12 *Drop out rate*: 3/15, 20% Overall *Age (years) (mean SD)*: 73.72 (5.58) *Ethnicity*: not reported *Gender (males, females)*: 4/24 M (16.7%); 20/24 F (83.3%) *Length of surgery (minutes) (mean SD)*: not reported *Proportion of participants on anticoagulants prior to surgery (n, %)*: not reported *Incidence of preoperative anaemia (n/N, %)*: not reported *Co‐morbidities (n/N, %)*: not reported *ASA 1 (n, %)*: not reported *ASA 2 (n, %)*: not reported *ASA 3 (n, %)*: not reported *ASA 4 (n, %)*: not reported *Number of participants randomised*: 28 *Number of participants receiving treatment*: 25 *Number of participants analysed*: 24 *Drop out rate*: 4/28, 14.3% **Inclusion criteria:** the study included male and female patients aged ≥ 18 years who underwent primary unilateral TKR and who signed the informed consent form **Exclusion criteria:** exclusion criteria were allergy to TXA, history of thromboembolic disease, postoperative iron treatment, use of blood recovery systems during surgery, history of convulsions, severe renal disease and pharmacologic contraceptive treatment **If TKR, is tourniquet used:** yes **Indication for surgery:** not reported **Type of anaesthetic:** not reported **Type of surgery:** primary unilateral total knee replacement	
Interventions	a**Intervention characteristics** TXA, IV Intravenous TXA administered, according to the hospital protocol, as 2 x 30‐min infusions of 1 g each of TXA (Amchafbrin, Rottafarm SL, Valencia, Spain) diluted in 1000 mL of saline solution. The first dose was administered 15 to 30 min before the pneumatic tourniquet was inflated, and the second dose was administered at the end of surgery, when the pneumatic tourniquet was deflated (between 90 and 140 min). TXA, IV, 2 g, intraop TXA, IA Intra‐articular TXA: single dose of 1 g of TXA diluted in 10 mL of water for injection (concentration of 100 mg/mL) was administered to the surgical field using a syringe before wound closure. TXA was applied first to the intercondylar space with the knee in flexion and then extension, to avoid spilling fluid, then to the synovial membrane, capsule and subcutaneous tissues. TXA, IA, 1 g, intraop	
Outcomes	*Primary outcome:* Pharmacokinetics *Secondary outcomes:* Proportion of patients who need blood transfusion in the postoperative period Pre‐ and postoperative haemoglobin Number of blood transfusions Number of blood transfusion units administered Incidence of infection of the wound Days of hospital stay Adverse effects related to study drugs Concentration of TXA in the blood collected by the drainage	
Notes	**Sponsorship source:** non‐pharmaceutical (this study was supported by internal sources from the Institut de Recerca Hospital de la Santa Creu i Sant Pau, Barcelona, Spain) **Country:** Spain **Setting:** single‐centre (found from trial registration) **Comments:** 1) correct trial registration is in full body of text, trial registration in abstract is wrong, 2) unable to find this abstract online **Author's name:** A González Osuna **Institution:** Hospital de la Santa Creu i Sant Pau, Universitat Autònoma de Barcelona **Email:** agonzalezo@santpau.cat; aranzazu23@gmail.com **Address:** Orthopaedic Surgery and Traumatology Department, Hospital de la Santa Creu i Sant Pau, Universitat Autònoma de Barcelona, Barcelona, Spain **Native language of paper:** English **Reference type:** trial registration (1) full‐text (1) **Trial registration number:** Spanish Clinical Studies Registry Number: 2017‐004159‐22 **Was it translated for this review:** no	
***Risk of bias***		
**Bias**	**Authors' judgement**	**Support for judgement**
Random sequence generation (selection bias)	Low risk	Quote: "A computer‐generated randomization schedule was prepared before the study" Judgement comment: adequate method of sequence generation with computer‐generated code.
Allocation concealment (selection bias)	Low risk	Quote: "web‐based platform allocated each patient to one of the TXA administration routes." Judgement comment: adequate method of allocation concealment using computer‐generated code.
Blinding of participants and personnel (performance bias) Subjective outcomes	High risk	Quote: not applicable Judgement comment: blinding not possible due to differences in route of administration of TXA.
Blinding of participants and personnel (performance bias) Objective outcomes	Low risk	Quote: not applicable Judgement comment: objective outcome for personnel, so low risk of bias regardless of quality of blinding.
Blinding of outcome assessment (detection bias) Subjective outcomes	Unclear risk	Quote: not applicable Judgement comment: no information given on this.
Blinding of outcome assessment (detection bias) Objective outcomes	Low risk	Quote: not applicable Judgement comment: objective outcome for outcome assessor, so low risk of bias regardless of quality of blinding.
Incomplete outcome data (attrition bias) All outcomes	Low risk	Quote: "Three patients (one in the intravenous group and two in the intra‐articular group) dropped out before the drug was administered. Therefore, 25 patients formed the safety population. Another patient received the experimental administration (intra‐articular) but was not included in the PK analysis because their vein condition hampered the drawing of blood samples." Judgement comment: small numbers of participants dropped out. One participant was excluded from analysis of effectiveness outcomes because of inability to obtain blood samples.
Selective reporting (reporting bias)	Low risk	Quote: not applicable Judgement comment: all outcomes specified in the trial registration were reported.
Other bias	Low risk	Quote: not applicable Judgement comment: no other concerns such as early stopping or imbalanced study arms.

**Table:** Good 2003

***Study characteristics***		
Methods	**Study design:** RCT **Intention‐to‐treat analysis:** yes **Duration of study:** not reported **Power calculation reached:** yes **Transfusion strategy:** if Hb was less than 90 g/L, allogeneic leucodepleted red blood cell concentrate was given in 250 mL units containing about 150 mL cells and 10 ± 20 mL plasma. **Was the trial stopped early:** no	
Participants	**Baseline characteristics** Placebo *Age (years) (mean range)*: 72 (50 to 84) *Ethnicity*: not reported *Gender (males, females)*: 6/24 M (25%); 18/24 F (75%) *Length of surgery (minutes) (mean SD)*: not reported *Proportion of participants on anticoagulants prior to surgery (n, %)*: 0, 0% *Incidence of preoperative anaemia (n/N, %)*: not reported *Co‐morbidities (n/N, %)*: not reported *ASA 1 (n, %)*: 7, 29% *ASA 2 (n, %)*: 17, 71% *ASA 3 (n, %)*: 0, 0% *ASA 4 (n, %)*: 0, 0% *Number of participants randomised*: 27 *Number of participants receiving treatment*: not reported *Number of participants analysed*: 24 *Dropout rate*: 3/27 (11.1%) TXA *Age (years) (mean range)*: 72 (46 to 83) *Ethnicity*: not reported *Gender (males, females)*: 9/27 M (33%); 18/27 F (67%) *Length of surgery (minutes) (mean SD)*: not reported *Proportion of participants on anticoagulants prior to surgery (n, %)*: 0, 0% *Incidence of preoperative anaemia (n/N, %)*: not reported *Co‐morbidities (n/N, %)*: not reported *ASA 1 (n, %)*: 8, 30% *ASA 2 (n, %)*: 19, 70% *ASA 3 (n, %)*: 0, 0% *ASA 4 (n, %)*: 0, 0% *Number of participants randomised*: 28 *Number of participants receiving treatment*: not reported *Number of participants analysed*: 27 *Dropout rate*: 1/28 (3.7%) Overall *Age (years) (mean range)*: not reported *Ethnicity*: not reported *Gender (males, females)*: not reported *Length of surgery (minutes) (mean SD)*: not reported *Proportion of participants on anticoagulants prior to surgery (n, %)*: not reported *Incidence of preoperative anaemia (n/N, %)*: not reported *Co‐morbidities (n/N, %)*: not reported *ASA 1 (n, %)*: not reported *ASA 2 (n, %)*: not reported *ASA 3 (n, %)*: not reported *ASA 4 (n, %)*: not reported *Number of participants randomised*: not reported *Number of participants receiving treatment*: not reported *Number of participants analysed*: not reported *Dropout rate*: not reported **Inclusion criteria:** 1) elective total primary unilateral tricompartmental knee arthroplasty because of osteoarthrosis, 2) ASA I or II **Exclusion criteria:** 1) history of coagulopathy, an abnormally great prothrombin or activated partial thrombin time, 2) previous history of a thromboembolic event, 3) treatment with aspirin or non‐steroidal anti‐inflammatory agents (NSAID) in the previous week, 4) plasma creatinine greater than 115 mmol/L in men and 100 mmol/L in women, 5) acute infection (e.g. with leucocytosis or fever), 6) malignant disease, 7) patients with myocardial infarction in the preceding 12 months or those with unstable angina or coronary disease that would not allow haemodilution, 8) patients given plasma or other treatment affecting coagulation during the peri operative period **If TKR, is tourniquet used:** yes **Indication for surgery:** osteoarthrosis **Type of anaesthetic:** spinal **Type of surgery:** primary TKR	
Interventions	**Intervention characteristics** Placebo At the end of the surgical procedure, just before release of the tourniquet, tranexamic acid 10 mg kg ± 1 or placebo was infused IV (maximum dose 1000 mg). The dose was repeated after 3 h. Placebo, IV, intraop (just before tourniquet release) AND placebo, IV, postop (3 hours postop) TXA At the end of the surgical procedure, just before release of the tourniquet, tranexamic acid 10 mg kg ± 1 or placebo was infused IV (maximum dose 1000 mg). The dose was repeated after 3 h. TXA, IV, 10 mg/kg (MAX 1g), intraop (just before tourniquet release) AND TXA, IV, 10 mg/kg (MAX 1 g), postop (3 hours postop)	
Outcomes	*Primary outcome:* Total blood loss *Secondary outcome:* Not reported	
Notes	**Sponsorship source:** non‐pharmaceutical **Country:** Sweden **Setting:** not reported **Comments:** none **Author's name:** L Good **Institution:** University of Linkoping, S‐581 85, Linkoping, Sweden **Email:** bjorn.lisander@lio.se **Address:** Department of Anaesthesiology and Intensive Care, Faculty of Health Sciences, University of Linkoping, S‐581 85, Linkoping, Sweden **Native language of paper:** English **Reference type:** full text (1) **Trial registration number:** not applicable, but when checked, trial registration number not found **Was it translated for this review:** no	
***Risk of bias***		
**Bias**	**Authors' judgement**	**Support for judgement**
Random sequence generation (selection bias)	Low risk	Quote: "saline were prepared by Apoteksbolaget, Umeå Sweden. The contents of the ampoules were randomized in blocks of 10 (5 saline, 5 tranexamic acid) by computer‐generated numbers." Judgement comment: computer‐generated randomisation.
Allocation concealment (selection bias)	Low risk	Quote: "Coded ampoules containing either tranexamic acid 100 mg ml ±1 (Cyklokapron®, Pharmacia) or saline were prepared by Apoteksbolaget, Umeå Sweden." Judgement comment: central allocation.
Blinding of participants and personnel (performance bias) Subjective outcomes	Unclear risk	Quote: not applicable Judgement comment: insufficient information to permit judgement.
Blinding of participants and personnel (performance bias) Objective outcomes	Low risk	Quote: not applicable Judgement comment: objective outcome for personnel, so low risk of bias regardless of quality of blinding.
Blinding of outcome assessment (detection bias) Subjective outcomes	Unclear risk	Quote: not applicable Judgement comment: insufficient information to permit judgement.
Blinding of outcome assessment (detection bias) Objective outcomes	Low risk	Quote: not applicable Judgement comment: objective outcome for outcome assessor, so low risk of bias regardless of quality of blinding.
Incomplete outcome data (attrition bias) All outcomes	Low risk	Quote: not applicable Judgement comment: no missing data.
Selective reporting (reporting bias)	Unclear risk	Quote: not applicable Judgement comment: no protocol available to check pre‐specified outcomes reported.
Other bias	Unclear risk	Quote: not applicable Judgement comment: unclear number of participants receiving treatment.

**Table:** Goyal 2017

***Study characteristics***		
Methods	**Study design:** RCT **Intention‐to‐treat analysis:** yes **Duration of study:** 5 months + follow up **Power calculation reached:** yes **Transfusion strategy:** the triggers for transfusion were based on National Blood Authority Guidelines. These guidelines recommend red blood cell (RBC) transfusion with Hb levels of < 7 g/dL. A single unit of RBCs for Hb levels between 7 and 10 g/dL is recommended if there is associated acute myocardial or cerebrovascular ischaemia. Blood transfusion is not advised if the Hb level is > 10 g/dL and is considered inappropriate for postoperative Hb levels more than 8 g/dL in the absence of acute myocardial or cerebrovascular ischaemia. **Was the trial stopped early:** no	
Participants	**Baseline characteristics** TXA, IV *Age (years) (mean SD)*: 68.8 (7.4) *Ethnicity*: not reported *Gender (males, females)*: 40/87 M (46%); 47/87 F (54%) *Length of surgery (minutes) (mean SD)*: 96.7 (25.2) *Proportion of participants on anticoagulants prior to surgery (n, %)*: not reported *Incidence of preoperative anaemia (n/N, %)*: 0/0 (0%) *Co‐morbidities (n/N, %)*: not reported *ASA 1 (n/N, %)*: 8/85, (9%) *ASA 2 (n/N, %)*: 50/85, (59%) *ASA 3 (n/N, %)*: 27/85, (32%) *ASA 4 (n/N, %)*: 0/0, (0%) *Number of participants randomised*: 91 *Number of participants receiving treatment*: 91 *Number of participants analysed*: 85 *Dropout rate*: 6/91, (6.59%) TXA, IA *Age (years) (mean SD)*: 66.7 (8.9) *Ethnicity*: not reported *Gender (males, females)*: 38/81 M (47%); 43/81 F (53%) *Length of surgery (minutes) (mean SD)*: 95.7 (20.5) *Proportion of participants on anticoagulants prior to surgery (n, %)*: not reported *Incidence of preoperative anaemia (n/N, %)*: 0/0 (0%) *Co‐morbidities (n/N, %)*: not reported *ASA 1 (n/N, %)*: 10/83, (12%) *ASA 2 (n/N, %)*: 46/83, (55%) *ASA 3 (n/N, %)*: 27/83, (32%) *ASA 4 (n/N, %)* : 0/0, (0%) *Number of participants randomised*: 92 *Number of participants receiving treatment*: 92 *Number of participants analysed*: 83 *Dropout rate*: 9/92, (9.78%) Overall *Age (years) (mean SD)*: not reported *Ethnicity*: not reported *Gender (males, females)*: not reported *Length of surgery (minutes) (mean SD)*: not reported *Proportion of participants on anticoagulants prior to surgery (n, %)*: not reported *Incidence of preoperative anaemia (n/N, %)*: not reported *Co‐morbidities (n/N, %)*: not reported *ASA 1 (n/N, %)*: not reported *ASA 2 (n/N, %)*: not reported *ASA 3 (n/N, %)*: not reported *ASA 4 (n/N, %)*: not reported *Number of participants randomised*: not reported *Number of participants receiving treatment*: not reported *Number of participants analysed*: not reported *Dropout rate*: not reported **Inclusion criteria:** 1) patients undergoing primary unilateral TKA **Exclusion criteria:** 1) patients undergoing bilateral TKA, 2) history of thromboembolic events (deep vein thrombosis (DVT), pulmonary embolism or cerebrovascular accident), 3) renal dysfunction (plasma creatinine level > 130 mmol/L), 4) coagulopathy (INR > 1.4), 5) patients with preoperative anaemia (men with Hb < 13 g/dL; women with Hb < 12 g/dL) **If TKR, is tourniquet used:** 1 surgeon did not use a tourniquet, 2 surgeons used it for the operative exposure only, while 1 surgeon used for the whole procedure. **Indication for surgery:** not reported **Type of anaesthetic:** spinal combined with sedation or general anaesthesia **Type of surgery:** primary TKR	
Interventions	**Intervention characteristics** TXA, IV Group 2 (IV group) received 1000 mg (10 mL) of IV TXA 10 minutes before deflation of the tourniquet (if a tourniquet was used) or 10 minutes before incision (if a tourniquet was not used), 30 mL of IA saline to the knee joint after wound closure, and 2 more 1000 mg (10 mL) doses of IV TXA were given at 8‐hourly intervals postoperatively. The rationale behind the use of 2 postoperative doses of IV TXA is to provide equal amount of TXA in the 2 groups. It is also the approved dosing regimen recommended by the pharmaceutical company for therapeutic use. TXA, IV, 1 g, intraop (10 mins before tourniquet deflation if used or 10 mins before incision if tourniquet not used) AND placebo, IA, intraop, after wound closure AND TXA, IV, 1 g, postop 8 + 16 hours TXA, IA Group 1 (IA group) received 10 mL of saline IV 10 minutes before deflation of the tourniquet (if a tourniquet was used) or 10 minutes before incision (if a tourniquet was not used), 3000 mg (30 mL) of IA TXA to the knee joint after wound closure, and 2 more 10 mL doses of IV saline were given at 8‐hourly intervals postoperatively. The syringes used to inject TXA into the knee joint after wound closure were covered with an opaque dressing to keep the operating team blinded. Placebo, IV, intraop (10 mins before tourniquet deflation if used or 10 mins before incision if tourniquet not used) AND TXA, IA, 3 g, intraop, after wound closure AND placebo, IV, 1 g, postop 8 + 16 hours	
Outcomes	*Primary outcome:* Reduction in the Hb drop as measured by the difference in the preoperative and postoperative day 1 Hb level *Secondary outcomes*: Difference between Hb levels preoperatively and at day 2 postoperatively Transfusion requirements were measured as the proportion of patients requiring transfusion and the number of units transfused Length of stay Complications	
Notes	**Sponsorship source:** none **Country:** Australia **Setting:** single‐centre **Comments:** none **Author's name:** N Goyal **Institution:** St George Private Hospital **Email:** not reported **Address:** Sydney Knee Specialists, St George Private Hospital, Suite 8, 19 Kensington St, Kogarah, NewSouth Wales 2217, Australia **Native language of paper:** English **Reference type:** full text (1), abstract (3), trial registration (1) **Trial registration number:** ACTRN12614000582651 **Was it translated for this review:** no	
***Risk of bias***		
**Bias**	**Authors' judgement**	**Support for judgement**
Random sequence generation (selection bias)	Low risk	Quote: "Sealed opaque envelopes were made based upon random numbers generated by computer." Judgement comment: computer‐generated randomisation.
Allocation concealment (selection bias)	Unclear risk	Quote: "Sealed opaque envelopes were made based upon random numbers generated by computer. These envelopes were only accessible to the anesthesiologist who provided the IV and the IA injection material for each participant. The operating team, patients, and the data collection team were blinded to the group to which the patients were randomized. Only the anesthesiologist who prepared the treatment solution was aware of the allocation and was not involved in data collection or data analysis." Judgement comment: envelopes were sealed and opaque but does not mention if they were sequentially numbered.
Blinding of participants and personnel (performance bias) Subjective outcomes	High risk	Quote: "These envelopes were only accessible to the anesthesiologist who provided the IV and the IA injection material for each participant." Judgement comment: blinding of key personnel not ensured.
Blinding of participants and personnel (performance bias) Objective outcomes	Low risk	Quote: not applicable Judgement comment: objective outcome for personnel, so low risk of bias regardless of quality of blinding.
Blinding of outcome assessment (detection bias) Subjective outcomes	Low risk	Quote: "The operating team, patients, and the data collection team were blinded to the group to which the patients were randomized. Only the anesthesiologist who prepared the treatment solution was aware of the allocation and was not involved in data collection or data analysis." Judgement comment: blinding of outcome assessors likely ensured.
Blinding of outcome assessment (detection bias) Objective outcomes	Low risk	Quote: not applicable Judgement comment: objective outcome for outcome assessor, so low risk of bias regardless of quality of blinding.
Incomplete outcome data (attrition bias) All outcomes	Unclear risk	Quote: "These 15 patients did not satisfy the inclusion criteria for preoperative Hb (14 patients) or creatinine (1 patient). This was a human error as they were included although they did not satisfy the inclusion criteria and, therefore, were subsequently taken off from the final patient analysis." Judgement comment: human error reported, however clear discrepancy from inclusion criteria and represents a significant proportion of patients in the analysis.
Selective reporting (reporting bias)	High risk	Quote: not applicable Judgement comment: not all outcomes mentioned in the trial registration were reported in the paper. Trial registration outcomes including secondary outcomes knee joint swelling, pain assessment, passive knee motion.
Other bias	Unclear risk	Quote: "One or more of the authors of this paper have disclosed potential or pertinent conflicts of interest, which may include receipt of payment, either direct or indirect, institutional support, or association with an entity in the biomedical field which may be perceived to have potential conflict of interest with this work." Judgement comment: insufficient information to permit judgement. Reported conflicts of interest ‐ unclear source of bias. Tourniquet use not consistent amongst the treatment arms.

**Table:** Harley 2002

***Study characteristics***		
Methods	**Study design:** RCT **Intention‐to‐treat analysis:** no **Duration of study:** 19 months **Power calculation reached:** yes **Transfusion strategy:** packed red blood cells were transfused according to standardised guidelines that exist for orthopaedic patients at the study institute: haemoglobin level less than 80 g/L or a haematocrit less than 0.24; patients having symptoms from their anaemia (including tachycardia, dyspnoea, chest pain or recurrent syncope); or medical conditions that render the patient unable to compensate for diminished oxygen carrying capacity **Was the trial stopped early:** no	
Participants	**Baseline characteristics** Placebo: N *Age (years) (mean SD)*: 69 (10) *Ethnicity*: not reported *Gender (males, females)*: 11/29 M (38%); 18/29 F (62%) *Length of surgery (minutes) (mean SD)*: 80 (20) *Proportion of participants on anticoagulants prior to surgery (n/N, %)*: 15/29 (52%) *Incidence of preoperative anaemia (n/N, %)*: not reported *Co‐morbidities (n/N, %)*: not reported *ASA 1 (n/N, %)*: not reported *ASA 2 (n/N, %)*: not reported *ASA 3 (n/N, %)*: not reported *ASA 4 (n/N, %)*: not reported *Number of participants randomised*: 29 *Number of participants receiving treatment*: 29 *Number of participants analysed*: 24 *Dropout rate*: 5/29 (17.24%) EACA *Age (years) (mean SD)*: 69 (11) *Ethnicity*: not reported *Gender (males, females)*: 10/26 M (38%); 16/26 F (62%) *Length of surgery (minutes) (mean SD)*: 77 (17) *Proportion of participants on anticoagulants prior to surgery (n/N, %)*: 21/26 (81%) *Incidence of preoperative anaemia (n/N, %)*: not reported *Co‐morbidities (n/N, %)*: not reported *ASA 1 (n/N, %)*: not reported *ASA 2 (n/N, %)*: not reported *ASA 3 (n/N, %)*: not reported *ASA 4 (n/N, %)*: not reported *Number of participants randomised*: 26 *Number of participants receiving treatment*: 26 *Number of participants analysed*: 22 *Dropout rate*: 4/26 (15.38%) Overall *Age (years) (mean SD)*: not reported *Ethnicity*: not reported *Gender (males, females)*: not reported *Length of surgery (minutes) (mean SD)*: not reported *Proportion of participants on anticoagulants prior to surgery (n/N, %)*: not reported *Incidence of preoperative anaemia (n/N, %)*: not reported *Co‐morbidities (n/N, %)*: not reported *ASA 1 (n/N, %)*: not reported *ASA 2 (n/N, %)*: not reported *ASA 3 (n/N, %)*: not reported *ASA 4 (n/N, %)*: not reported *Number of participants randomised*: not reported *Number of participants receiving treatment*: not reported *Number of participants analysed*: not reported *Dropout rate*: not reported **Inclusion criteria:** 1) all patients scheduled to undergo a primary THA by 1 of 4 experienced surgeons **Exclusion criteria:** 1) known allergy to EACA, 2) a history of renal or hepatic failure, 3) coagulopathy, 4) uncontrolled hypertension, 5) symptomatic cardiac or pulmonary failure, 6) known upper urinary tract bleeding **If TKR, is tourniquet used:** NA **Indication for surgery:** primary and secondary osteoarthritis, rheumatoid arthritis **Type of anaesthetic:** mixed (at the discretion of the anaesthetist) **Type of surgery:** primary THR	
Interventions	**Intervention characteristics** Placebo An EACA loading dose of 150 mg/kg, or the equivalent dose of placebo, was administered as a bolus load over 20 minutes on the patient’s arrival in the operating room. An hourly EACA infusion of 12.5 mg/kg, or equivalent placebo, was subsequently administered for an additional 5 hours. Placebo, IV, intraop (arrival of patient into theatre), 20 min loading infusion + placebo, IV, intraop, 5‐hour infusion EACA An EACA loading dose of 150 mg/kg, or the equivalent dose of placebo, was administered as a bolus load over 20 minutes on the patient’s arrival in the operating room. An hourly EACA infusion of 12.5 mg/kg, or equivalent placebo, was subsequently administered for an additional 5 hours. EACA, IV, 150 mg/kg, intraop (arrival of patient into theatre), 20 min loading infusion + EACA, IV, 12.5 mg/kg, intraop, 5‐hour infusion	
Outcomes	*Primary outcome*: Reduction in blood loss *Secondary outcomes*: Haemoglobin levels Coagulation profiles Number of transfusions required	
Notes	**Sponsorship source:** not reported **Country:** Canada **Setting:** single‐centre **Comments:** none **Author's name:** BJ Harley **Institution:** University of Alberta Hospital and Royal Alexandra Hospital **Email:** not reported **Address:** Office of Orthopaedic Research, University of Alberta Hospital, 1F1.52 Walter Mackenzie Centre, 8440–112th Street, Edmonton AB **Native language of paper:** English **Reference type:** full text (1) abstract (1) **Trial registration number:** not applicable, but when checked, trial registration number not found **Was it translated for this review:** no	
***Risk of bias***		
**Bias**	**Authors' judgement**	**Support for judgement**
Random sequence generation (selection bias)	Unclear risk	Quote: not applicable Judgement comment: insufficient information to permit judgement.
Allocation concealment (selection bias)	Unclear risk	Quote: "Patients were randomly assigned to receive either EACA or saline placebo administered from uniformly blinded bottles." Judgement comment: insufficient information to permit judgement.
Blinding of participants and personnel (performance bias) Subjective outcomes	Low risk	Quote: "Patients were randomly assigned to receive either EACA or saline placebo administered from uniformly blinded bottles. Patients, anesthesiologists, surgeons and clinical evaluators were blinded to the patient allocation." Judgement comment: key personnel probably blinded.
Blinding of participants and personnel (performance bias) Objective outcomes	Low risk	Quote: not applicable Judgement comment: objective outcome for personnel, so low risk of bias regardless of quality of blinding.
Blinding of outcome assessment (detection bias) Subjective outcomes	Low risk	Quote: "Patients were randomly assigned to receive either EACA or saline placebo administered from uniformly blinded bottles. Patients, anesthesiologists, surgeons and clinical evaluators were blinded to the patient allocation." Judgement comment: blinding of outcome assessors probably blinded to intervention arm.
Blinding of outcome assessment (detection bias) Objective outcomes	Low risk	Quote: not applicable Judgement comment: objective outcome for outcome assessor, so low risk of bias regardless of quality of blinding.
Incomplete outcome data (attrition bias) All outcomes	Low risk	Quote: not applicable Judgement comment: no missing outcome data.
Selective reporting (reporting bias)	Unclear risk	Quote: not applicable Judgement comment: no protocol available to check pre‐specified outcomes reported.
Other bias	High risk	Quote: "Although 55 patients were enrolled, 4 patients in the EACA group and 5 patients in the placebo group were excluded postoperatively due to breaches in the anesthetic protocol or operating room instructions: use of a cell‐saver system (2 patients), colloid use (2 patients), incorrect administration of the drug bolus (2 patients), errors in measurement of intraoperative blood loss (2 patients) and packed red blood cell transfusion for an incorrectly reported postoperative hemoglobin level (1 patient)." Judgement comment: appears that per protocol analysis undertaken.

**Table:** Hayes 1996

***Study characteristics***		
Methods	**Study design:** RCT **Intention‐to‐treat analysis:** yes **Duration of study:** not reported **Power calculation reached:** not reported **Transfusion strategy:** if the intraoperative blood loss was greater than the calculated maximum allowable blood loss, then transfusion with blood took place. **Was the trial stopped early:** no	
Participants	**Baseline characteristics** Placebo *Age (years) (mean SD)*: 72.9 (10.3) *Ethnicity*: not reported *Gender (males, females) ASSUMED*: 7/22 M (32%); 15/22 F (68%) *Length of surgery (minutes) (mean SD)*: 99.6 (21.6) *Proportion of participants on anticoagulants prior to surgery (n/N, %)*: not reported *Incidence of preoperative anaemia (n/N, %)*: not reported *Co‐morbidities (n/N, %)*: not reported *ASA 1 (n/N, %)*: not reported *ASA 2 (n/N, %)*: not reported *ASA 3 (n/N, %)*: not reported *ASA 4 (n/N, %)*: not reported *Number of participants randomised*: 20 *Number of participants receiving treatment*: 20 *Number of participants analysed*: 20 *Dropout rate*: 0/20 (0%) Aprotinin *Age (years) (mean SD)*: 70 (7.9) *Ethnicity*: not reported *Gender (males/females) ASSUMED*: 8/20 M (40%), 12/20 F (60%) *Length of surgery (minutes) (mean SD)*: 93.46 (24.6) *Proportion of participants on anticoagulants prior to surgery (n/N, %)*: not reported *Incidence of preoperative anaemia (n/N, %)*: not reported *Co‐morbidities (n/N, %)*: not reported *ASA 1 (n/N, %)*: not reported *ASA 2 (n/N, %)*: not reported *ASA 3 (n/N, %)*: not reported *ASA 4 (n/N, %)*: not reported *Number of participants randomised*: 20 *Number of participants receiving treatment*: 20 *Number of participants analysed*: 20 *Dropout rate*: 0/20 (0%) Overall *Age (years) (mean SD)*: not reported *Ethnicity*: not reported *Gender (males, females) ASSUMED*: not reported *Length of surgery (minutes) (mean SD)*: not reported *Proportion of participants on anticoagulants prior to surgery (n/N, %)*: not reported *Incidence of preoperative anaemia (n/N, %)*: not reported *Co‐morbidities (n/N, %)*: not reported *ASA 1 (n/N, %)*: not reported *ASA 2 (n/N, %)*: not reported *ASA 3 (n/N, %)*: not reported *ASA 4 (n/N, %)*: not reported *Number of participants randomised*: not reported *Number of participants receiving treatment*: not reported *Number of participants analysed*: not reported *Dropout rate*: not reported **Inclusion criteria:** 1) patients scheduled for elective total hip replacement surgery **Exclusion criteria:** 1) known hypersensitivity to aprotinin, 2) preoperative renal, cardiac, hepatic and pulmonary failure, 3) uncontrolled hypertension, 4) pre‐existing coagulation abnormalities, 5) a recent history of thromboembolic events, 6) patients with previous exposure to aprotinin **If TKR, is tourniquet used:** not applicable **Indication for surgery:** not reported **Type of anaesthetic:** spinal anaesthesia **Type of surgery:** primary THR	
Interventions	**Intervention characteristics** Placebo Patients were randomly allocated to one of 2 groups: those who received aprotinin 2 million KIU intravenously (IV) (Group A) and those who received placebo (Group C). Patients in Group C served as controls and were given an equal volume infusion consisting of 0.9% normal saline. Placebo, IV, preop, bolus + preop infusion (20 mins prior to incision) Aprotinin Group A received aprotinin 50,000 KIU (5 mL) IV over a 10‐minute period. If there were no adverse sequelae, the remaining dose of 2 million KIU of aprotinin was given over 20 minutes prior to surgical incision. Aprotinin, IV, 0.5 x 10^6 KIU, preop, bolus + 2 x 10^6 preop infusion (20 mins prior to incision)	
Outcomes	*Primary outcomes:* Blood loss Transfusion requirements *Secondary outcome:* Not reported	
Notes	**Sponsorship source:** part non‐pharmaceutical **Country:** Ireland **Setting:** not reported **Comments:** none **Author's name:** A Hayes **Institution:** University Hospital Wilton **Email:** not reported **Address:** Department of Clinical Pharmacology and Therapeutics, Cork University Hospital, Wilton, Cork, Ireland **Native language of paper:** English **Reference type:** full text (1) **Trial registration number:** not applicable, but when checked, trial registration number not found **Was it translated for this review:** no	
***Risk of bias***		
**Bias**	**Authors' judgement**	**Support for judgement**
Random sequence generation (selection bias)	Unclear risk	Quote: "Patients were randomly allocated to one of two groups: those who received aprotinin 2 million KIU intravenously (Iv) (Group A) and those who received placebo (Group C)." Judgement comment: insufficient information to permit judgement.
Allocation concealment (selection bias)	Unclear risk	Quote: "Patients were randomly allocated to one of two groups: those who received aprotinin 2 million KIU intravenously (Iv) (Group A) and those who received placebo (Group C)." Judgement comment: insufficient information to permit judgement.
Blinding of participants and personnel (performance bias) Subjective outcomes	Unclear risk	Quote: not applicable Judgement comment: insufficient information to permit judgement.
Blinding of participants and personnel (performance bias) Objective outcomes	Low risk	Quote: not applicable Judgement comment: objective outcome for personnel, so low risk of bias regardless of quality of blinding.
Blinding of outcome assessment (detection bias) Subjective outcomes	Unclear risk	Quote: not applicable Judgement comment: insufficient information to permit judgement.
Blinding of outcome assessment (detection bias) Objective outcomes	Low risk	Quote: not applicable Judgement comment: objective outcome for outcome assessor, so low risk of bias regardless of quality of blinding.
Incomplete outcome data (attrition bias) All outcomes	Low risk	Quote: not applicable Judgement comment: no missing data.
Selective reporting (reporting bias)	Unclear risk	Quote: not applicable Judgement comment: no protocol to check pre‐specified outcomes reported.
Other bias	Unclear risk	Quote: not applicable Judgement comment: funding source not completely declared.

**Table:** Hiippala 1995

***Study characteristics***		
Methods	**Study design:** RCT **Intention‐to‐treat analysis:** unclear **Duration of study:** 6 months **Power calculation reached:** not reported **Transfusion strategy:** if the haemoglobin concentration was less than 100 g/L, concentrated red cells were given **Was the trial stopped early:** no	
Participants	**Baseline characteristics** Placebo *Age (years) (mean range)*: 70 (63 to 78) *Ethnicity*: not reported *Gender (males, females)*: 3/13 M (23%); 10/13 F (77%) *Length of surgery (minutes) (mean SD)*: 106 (17) *Proportion of participants on anticoagulants prior to surgery (n/N, %)*: not reported *Incidence of preoperative anaemia (n/N, %)*: not reported *Co‐morbidities (n/N, %)*: not reported *ASA 1 (n/N, %)*: not reported *ASA 2 (n/N, %)*: not reported *ASA 3 (n/N, %)*: not reported *ASA 4 (n/N, %)*: not reported *Number of participants randomised*: 14 *Number of participants receiving treatment*: not reported *Number of participants analysed*: 13 *Dropout rate*: 1/14 (7.1%) TXA *Age (years) (mean range)*: 70 (56 to 82) *Ethnicity*: not reported *Gender (males, females)*: 2/15 M (13%); 13/15 F (87%) *Length of surgery (minutes) (mean SD)*: 115 (18) *Proportion of participants on anticoagulants prior to surgery (n/N, %)*: not reported *Incidence of preoperative anaemia (n/N, %)*: not reported *Co‐morbidities (n/N, %)*: not reported *ASA 1 (n/N, %)*: not reported *ASA 2 (n/N, %)*: not reported *ASA 3 (n/N, %)*: not reported *ASA 4 (n/N, %)*: not reported *Number of participants randomised*: 15 *Number of participants receiving treatment*: not reported *Number of participants analysed*: 15 *Dropout rate*: 0/15 (0%) Overall *Age (years) (mean range)*: not reported *Ethnicity*: not reported *Gender (males, females)*: not reported *Length of surgery (minutes) (mean SD)*: not reported *Proportion of participants on anticoagulants prior to surgery (n/N, %)*: not reported *Incidence of preoperative anaemia (n/N, %)*: not reported *Co‐morbidities (n/N, %)*: not reported *ASA 1 (n/N, %)*: not reported *ASA 2 (n/N, %)*: not reported *ASA 3 (n/N, %)*: not reported *ASA 4 (n/N, %)*: not reported *Number of participants randomised*: 29 *Number of participants receiving treatment*: not reported *Number of participants analysed*: 28 *Dropout rate*: not reported **Inclusion criteria:** 1) patients undergoing total knee arthroplasty **Exclusion criteria:** not reported **If TKR, is tourniquet used:** yes **Indication for surgery:** osteoarthrosis, rheumatoid arthritis **Type of anaesthetic:** spinal **Type of surgery:** primary TKR	
Interventions	**Intervention characteristics** Placebo 2 to 5 minutes before deflating the tourniquet, the patients were given either a bolus of tranexamic acid (Cyklokapron, Pharmacia, Sweden) 15 mg kg‐1 or an equal volume of placebo (0.9% sodium chloride solution) IV within 1 min. Placebo, IV, intraop (2 to 5 mins prior to tourniquet deflation) TXA 2 to 5 minutes before deflating the tourniquet, the patients were given either a bolus of tranexamic acid (Cyklokapron, Pharmacia, Sweden) 15 mg kg‐1 or an equal volume of placebo (0.9% sodium chloride solution) IV within 1 min. TXA, IV, 15 mg/kg, intraop (2 to 5 mins prior to tourniquet deflation)	
Outcomes	*Primary outcome:* Blood loss *Secondary outcome*: Not reported	
Notes	**Sponsorship source:** not reported **Country:** Finland **Setting:** not reported **Comments:** none **Author's name:** S Hiippala **Institution:** South Carelian Central Hospital **Email:** not reported **Address:** Department of Orthopaedic Surgery; South Carelian Central Hospital, Kakelankatu 1, FIN‐ 53130 Lappeenranta, Finland **Native language of paper:** English **Reference type:** full text (1) **Trial registration number:** not applicable, but when checked, trial registration number not found **Was it translated for this review:** no	
***Risk of bias***		
**Bias**	**Authors' judgement**	**Support for judgement**
Random sequence generation (selection bias)	Unclear risk	Quote: "Randomization was carried out by a person not involved in the operation using a ticket drawn from an envelope containing an equal number of tranexamic acid and placebo tickets." Judgement comment: insufficient information to permit judgement.
Allocation concealment (selection bias)	Unclear risk	Quote: "involved in the operation using a ticket drawn from an envelope containing an equal number of tranexamic acid and placebo tickets." Judgement comment: insufficient information to permit judgement.
Blinding of participants and personnel (performance bias) Subjective outcomes	Unclear risk	Quote: "The operating team was unaware of the contents of the solution administered." Judgement comment: insufficient information to permit judgement.
Blinding of participants and personnel (performance bias) Objective outcomes	Low risk	Quote: not applicable Judgement comment: objective outcome for personnel, so low risk of bias regardless of quality of blinding.
Blinding of outcome assessment (detection bias) Subjective outcomes	Unclear risk	Quote: not applicable Judgement comment: no description of outcome assessor blinding given.
Blinding of outcome assessment (detection bias) Objective outcomes	Low risk	Quote: not applicable Judgement comment: objective outcome for outcome assessor, so low risk of bias regardless of quality of blinding.
Incomplete outcome data (attrition bias) All outcomes	Low risk	Quote: not applicable Judgement comment: no missing data reported.
Selective reporting (reporting bias)	High risk	Quote: not applicable Judgement comment: one or more outcomes of interest are reported incompletely such that they cannot be entered into a meta‐analysis.
Other bias	Unclear risk	Quote: not applicable Judgement comment: no funding source declared.

**Table:** Hiippala 1997

***Study characteristics***		
Methods	**Study design:** RCT **Intention‐to‐treat analysis:** no **Duration of study:** 19 months **Power calculation reached:** unclear **Transfusion strategy:** a transfusion of 1 or 2 units of red cells was ordered if the haemoglobin concentration decreased to less than 10 g/dL **Was the trial stopped early:** no	
Participants	**Baseline characteristics** Placebo *Age (years) (mean SD)*: 69 (5) *Ethnicity*: not reported *Gender (males, females)*: 8/38 M (21%); 30/38 F (79%) *Length of surgery (minutes) (mean SD)*: 96 (18) *Proportion of participants on anticoagulants prior to surgery (n/N, %)*: not reported *Incidence of preoperative anaemia (n/N, %)*: not reported *Co‐morbidities (n/N, %)*: not reported *ASA 1 (n/N, %)*: not reported *ASA 2 (n/N, %)*: not reported *ASA 3 (n/N, %)*: not reported *ASA 4 (n/N, %)*: not reported *Number of participants randomised*: unclear *Number of participants receiving treatment*: unclear *Number of participants analysed*: 38 *Dropout rate*: unclear TXA *Age (years) (mean SD)*: 70 (7) *Ethnicity*: not reported *Gender (males, females)*: 4/39 M (10%); 35/39 F (90%) *Length of surgery (minutes) (mean SD)*: 101 (21) *Proportion of participants on anticoagulants prior to surgery (n/N, %)*: not reported *Incidence of preoperative anaemia (n/N, %)*: not reported *Co‐morbidities (n/N, %)*: not reported *ASA 1 (n/N, %)*: not reported *ASA 2 (n/N, %)*: not reported *ASA 3 (n/N, %)*: not reported *ASA 4 (n/N, %)*: not reported *Number of participants randomised*: unclear *Number of participants receiving treatment*: unclear *Number of participants analysed*: 39 *Drop out rate*: unclear Overall *Age (years) (mean SD)*: not reported *Ethnicity*: not reported *Gender (males, females)*: not reported *Length of surgery (minutes) (mean SD)*: not reported *Proportion of participants on anticoagulants prior to surgery (n/N, %)*: not reported *Incidence of preoperative anaemia (n/N, %)*: not reported *Co‐morbidities (n/N, %)*: not reported *ASA 1 (n/N, %)*: not reported *ASA 2 (n/N, %)*: not reported *ASA 3 (n/N, %)*: not reported *ASA 4 (n/N, %)*: not reported *Number of participants randomised*: 77 *Number of participants receiving treatment*: not reported *Number of participants analysed*: not reported *Drop out rate*: not reported **Inclusion criteria:** 1) unilateral TKR patients **Exclusion criteria:** not reported **If TKR, is tourniquet used:** yes **Indication for surgery:** osteoarthrosis, rheumatoid arthritis **Type of anaesthetic:** spinal anaesthesia **Type of surgery:** primary TKR	
Interventions	**Intervention characteristics** Placebo Equal volume of normal saline was given to the patients in the placebo group Placebo, IV, intraop (at tourniquet deflation) AND placebo, IV, 3 to 4 hours post initial dose + 6 to 7 hours post TXA Patients in the treatment group (TXA) were given 15 mg/kg of tranexamic acid (Cyklokapron; Pharmacia, Uppsala, Sweden) intravenously just before the tourniquet was deflated. Two additional doses of 10 mg/kg were given during the operation day, the first in the recovery room 3 to 4 h after the initial dose and the second 6 to 7 h later on the surgical ward. TXA, IV, 15 mg/kg, intraop (at tourniquet deflation) AND TXA, IV, 10 mg/kg, 3 to 4 hours post initial dose + 6 to 7 hours post	
Outcomes	*Primary outcomes:* Blood loss Transfusion requirements *Secondary outcome:* Not reported	
Notes	**Sponsorship source:** not reported **Country:** Finland **Setting:** single‐centre **Comments:** counted number of patients rather than number of operations **Author's name:** S Hiippala **Institution:** South Carelian Central Hospital **Email:** not reported **Address:** Department of Anesthesia, Helsinki University Central Hospital, Haartmaninkatu 4, FIN‐00290 Helsinki, Finland **Native language of paper:** English **Reference type:** full text (1) **Trial registration number:** not applicable, but when checked, trial registration number not found **Was it translated for this review:** no	
***Risk of bias***		
**Bias**	**Authors' judgement**	**Support for judgement**
Random sequence generation (selection bias)	Unclear risk	Quote: "The patients were randomized into two groups" Judgement comment: insufficient information to permit judgement.
Allocation concealment (selection bias)	Unclear risk	Quote: "A ticket indicating the group was drawn and enclosed in an envelope." Judgement comment: insufficient information to permit judgement.
Blinding of participants and personnel (performance bias) Subjective outcomes	Low risk	Quote: "the blinded injection syringes were prepared by a person outside the surgical team." Judgement comment: likely blinding of participants and key personnel ensured.
Blinding of participants and personnel (performance bias) Objective outcomes	Low risk	Quote: not applicable Judgement comment: objective outcome for personnel, so low risk of bias regardless of quality of blinding.
Blinding of outcome assessment (detection bias) Subjective outcomes	Unclear risk	Quote: "The envelopes were opened after the study was completed and the exclusion criteria were checked. The operation team was unaware of the contents of the delivered syringes." Judgement comment: insufficient information to permit judgement.
Blinding of outcome assessment (detection bias) Objective outcomes	Low risk	Quote: not applicable Judgement comment: objective outcome for outcome assessor, so low risk of bias regardless of quality of blinding.
Incomplete outcome data (attrition bias) All outcomes	Low risk	Quote: not applicable Judgement comment: no missing data reported.
Selective reporting (reporting bias)	High risk	Quote: "Four weeks after the operation a second patient was treated in the hospital for 11 days due to cardiac problems." Judgement comment: one or more outcomes of interest are reported incompletely, such that they cannot be entered into a meta‐analysis.
Other bias	Unclear risk	Quote: not applicable Judgement comment: funding source not declared.

**Table:** Husted 2003

***Study characteristics***		
Methods	**Study design:** RCT **Intention‐to‐treat analysis:** yes **Duration of study:** not reported **Power calculation reached:** yes **Transfusion strategy:** if a patient had a reduction in haemoglobin exceeding 25% of the starting level and had clinical symptoms, a blood transfusion(s) was given. **Was the trial stopped early:** no	
Participants	**Baseline characteristics** Placebo *Age (years) (mean)*: 67 *Ethnicity*: not reported *Gender (males, females)*: 6/20 M (30%); 14/20 F (70%) *Length of surgery (mean, minutes)*: 76 *Proportion of participants on anticoagulants prior to surgery (n/N, %)*: not reported *Incidence of preoperative anaemia (n/N, %)*: not reported *Co‐morbidities (n/N, %)*: not reported *ASA 1 (n/N, %)*: not reported *ASA 2 (n/N, %)*: not reported *ASA 3 (n/N, %)*: not reported *ASA 4 (n/N, %)*: not reported *Number of participants randomised*: 20 *Number of participants receiving treatment*: 20 *Number of participants analysed*: 20 *Dropout rate*: 0/20 (0%) TXA *Age (years) (mean)*: 65 *Ethnicity*: not reported *Gender (males, females)*: not reported *Length of surgery (mean, minutes)*: 76 *Proportion of participants on anticoagulants prior to surgery (n/N, %)*: not reported *Incidence of preoperative anaemia (n/N, %)*: not reported *Co‐morbidities (n/N, %)*: not reported *ASA 1 (n/N, %)*: not reported *ASA 2 (n/N, %)*: not reported *ASA 3 (n/N, %)*: not reported *ASA 4 (n/N, %)*: not reported *Number of participants randomised*: 20 *Number of participants receiving treatment*: 20 *Number of participants analysed*: 20 *Dropout rate*: 0/20 (0%) Overall *Age (years) (mean)*: not reported *Ethnicity*: not reported *Gender (males, females)*: not reported *Length of surgery (mean, minutes)*: not reported *Proportion of participants on anticoagulants prior to surgery (n/N, %)*: not reported *Incidence of preoperative anaemia (n/N, %)*: not reported *Co‐morbidities (n/N, %)*: not reported *ASA 1 (n/N, %)*: not reported *ASA 2 (n/N, %)*: not reported *ASA 3 (n/N, %)*: not reported *ASA 4 (n/N, %)*: not reported *Number of participants randomised*: not reported *Number of participants receiving treatment*: not reported *Number of participants analysed*: not reported *Dropout rate*: not reported **Inclusion criteria:** 1) patients scheduled for primary total hip arthroplasty due to arthrosis or osteonecrosis of the femoral head **Exclusion criteria:** 1) rheumatoid arthritis, 2) malignancy, 3) previous thromboembolic episodes, 4) ischaemic heart disease, 5) previous subarachnoidal bleeding, 6) haematuria, 7) body weight > 100 kg **If TKR, is tourniquet used:** not applicable **Indication for surgery:** arthrosis or osteonecrosis of the femoral head **Type of anaesthetic:** spinal anaesthetic **Type of surgery:** primary THR	
Interventions	**Intervention characteristics** Placebo Patients randomised to receiving placebo (saline) were given a bolus intravenous injection of 20 mL about 15 minutes before the operation followed by a continuous infusion of 1 L of saline during 10 hours. Placebo, IV, preop (15 mins before incision), bolus + intraop infusion for 10 hours TXA Patients randomised to receive tranexamic acid were given a bolus intravenous injection of 10 mg/kg (maximum 1 g) during 10 minutes about 15 minutes before the incision, followed by a continuous infusion of 1 mg/kg/hour dissolved in 1 L of saline for 10 hours (maximum 1 g/10 hours). TXA, IV, 10 mg/kg, preop (15 mins before incision) bolus + 1 mg/kg/hr, intraop, infusion for 10 hours (max 1 g)	
Outcomes	*Primary outcomes*: Pre‐ and postoperative bleeding Number of blood transfusions *Secondary outcome*: None reported	
Notes	**Sponsorship source:** not reported **Country:** Denmark **Setting:** not reported **Comments:** mean worked out from text; unable to work out SD from text **Author's name:** H Husted **Institution:** Hvidovre University Hospital **Email:** henrikhusted@dadlnet.dk **Address:** Departments of Orthopaedics, Hvidovre University Hospital and Amager University Hospital, Copenhagen Hospital Corporation, Denmark **Native language of paper:** English **Reference type:** full text (1) **Trial registration number:** not applicable, but when checked, trial registration number not found **Was it translated for this review:** no	
***Risk of bias***		
**Bias**	**Authors' judgement**	**Support for judgement**
Random sequence generation (selection bias)	Low risk	Quote: "Randomization was done by a computer (Medstat, no block randomization)." Judgement comment: computer‐generated randomisation.
Allocation concealment (selection bias)	Unclear risk	Quote: "The drugs (tranexamic acid, 100 mg/mL, 4 ampoules of 5 mL or saline, 20 mL) were packed in numbered envelopes by a person not connected with the surgical procedure and handled by the anesthetist." Judgement comment: unclear whether sequentially numbered, sealed, opaque envelopes.
Blinding of participants and personnel (performance bias) Subjective outcomes	Unclear risk	Quote: "packed in numbered envelopes by a person not connected with the surgical procedure and handled by the anesthetist." Judgement comment: insufficient information to permit judgement.
Blinding of participants and personnel (performance bias) Objective outcomes	Low risk	Quote: not applicable Judgement comment: objective outcome for personnel, so low risk of bias regardless of quality of blinding.
Blinding of outcome assessment (detection bias) Subjective outcomes	Unclear risk	Quote: not applicable Judgement comment: no description of outcome assessor blinding.
Blinding of outcome assessment (detection bias) Objective outcomes	Low risk	Quote: not applicable Judgement Comment: Objective outcome for outcome assessors, so low risk of bias regardless of quality of blinding.
Incomplete outcome data (attrition bias) All outcomes	Low risk	Quote: not applicable Judgement comment: no missing data.
Selective reporting (reporting bias)	Unclear risk	Quote: not applicable Judgement comment: no protocol to check pre‐specified outcomes reported.
Other bias	Unclear risk	Quote: not applicable Judgement comment: no funding source declared.

**Table:** Jansen 1999

***Study characteristics***		
Methods	**Study design:** RCT **Intention‐to‐treat analysis:** yes **Duration of study:** not reported **Power calculation reached:** not reported **Transfusion strategy:** the indication for postoperative transfusion was set at a packed cell volume (PCV) of less than 26% in any of the postoperative measures. **Was the trial stopped early:** no	
Participants	**Baseline characteristics** Placebo *Age (years) (mean, range)*: 71.0 (64 to 84) *Ethnicity*: not reported *Gender (males, females)*: 3/21 M (14%); 18/21 F (86%) *Length of surgery (minutes) (mean SD)*: 94.4 (27.2) *Proportion of participants on anticoagulants prior to surgery (n/N, %)*: 0/21, (0%) *Incidence of preoperative anaemia (n/N, %)*: not reported *Co‐morbidities (n/N, %)*: not reported *ASA 1 (n/N, %)*: not reported *ASA 2 (n/N, %)*: not reported *ASA 3 (n/N, %)*: not reported *ASA 4 (n/N, %)*: 0/0 (0%) *Number of participants randomised*: 21 *Number of participants receiving treatment*: 21 *Number of participants analysed*: 21 *Dropout rate*: 0/21, (0%) TXA *Age (years) (mean, range)*: 70.7 (62 to 80) *Ethnicity*: not reported *Gender (males, females)*: 5/21 M (24%); 16/21 F (76%) *Length of surgery (minutes) (mean SD)*: 84.7 (15.9) *Proportion of participants on anticoagulants prior to surgery (n/N, %)*: 0/21, (0%) *Incidence of preoperative anaemia (n/N, %)*: not reported *Co‐morbidities (n/N, %)*: not reported *ASA 1 (n/N, %)*: not reported *ASA 2 (n/N, %)*: not reported *ASA 3 (n/N, %)*: not reported *ASA 4 (n/N, %)*: 0/0 (0%) *Number of participants randomised*: 21 *Number of participants receiving treatment*: 21 *Number of participants analysed*: 21 *Dropout rate*: 0/21, (0%) Overall *Age (years) (mean, range)*: not reported *Ethnicity*: not reported *Gender (males, females)*: not reported *Length of surgery (minutes) (mean SD)*: not reported *Proportion of participants on anticoagulants prior to surgery (n/N, %)*: not reported *Incidence of preoperative anaemia (n/N, %)*: not reported *Co‐morbidities (n/N, %)*: not reported *ASA 1 (n/N, %)*: not reported *ASA 2 (n/N, %)*: not reported *ASA 3 (n/N, %)*: not reported *ASA 4 (n/N, %)*: not reported *Number of participants randomised*: not reported *Number of participants receiving treatment*: not reported *Number of participants analysed*: not reported *Dropout rate*: not reported **Inclusion criteria:** 1) ASA I–III patients, 2) diagnosed with osteoarthrosis, 3) undergoing unilateral bicondylar cemented total knee arthroplasty **Exclusion criteria:** 1) known allergy to tranexamic acid, 2) preoperative hepatic or renal dysfunction, 3) serious cardiac or respiratory disease, 4) congenital or acquired coagulopathy, 5) a history of, or evolving, thromboembolic disease **If TKR, is tourniquet used:** yes **Indication for surgery:** osteoarthrosis **Type of anaesthetic:** general anaesthetic **Type of surgery:** primary TKR	
Interventions	**Intervention characteristics** Placebo Tranexamic acid 15 mg kg–1 (Exacyl, Bournonville Pharma, Belgium) or an equal volume of saline, in a double‐blind manner, 30 min before inflation of the tourniquet and surgery, and repeated subsequently every 8 h for 3 days. Placebo, IV, intraop (30 mins prior to tourniquet inflation AND placebo, IV, post 1st dose every 8 hours for 3 days TXA Patients were allocated randomly to receive tranexamic acid 15 mg kg–1 (Exacyl, Bournonville Pharma, Belgium) or an equal volume of saline, in a double‐blind manner, 30 min before inflation of the tourniquet and surgery, and repeated subsequently every 8 h for 3 days. TXA, IV, 15 mg/kg, intraop (30 mins prior to tourniquet inflation AND TXA, IV, 15 mg/kg, post 1st dose every 8 hours for 3 days	
Outcomes	*Primary outcomes*: Blood loss Blood transfusion requirements Blood coagulation Thromboembolic complications *Secondary outcome:* Not reported	
Notes	**Sponsorship source:** partly funded by pharmaceutical company **Country:** Belgium **Setting:** not reported **Comments:** none **Author's name:** AJ Jansen **Institution:** Vrije Universiteit Brussel **Email:** not reported **Address:** Department of Anaesthesiology, Academisch Ziekenhuis, Vrije Universiteit Brussel, Laarbeeklaan 101, B‐1090 Brussels, Belgium **Native language of paper:** English **Reference type:** abstract (1) full text (1) **Trial registration number:** not applicable, but when checked, trial registration number not found **Was it translated for this review:** no	
***Risk of bias***		
**Bias**	**Authors' judgement**	**Support for judgement**
Random sequence generation (selection bias)	Low risk	Quote: "Randomization was performed using a computer‐ generated random number list." Judgement comment: computer‐generated randomisation.
Allocation concealment (selection bias)	Unclear risk	Quote: not applicable Judgement comment: insufficient information to permit judgement.
Blinding of participants and personnel (performance bias) Subjective outcomes	Low risk	Quote: "Both the surgeon and anaesthetist were blinded to the treatment regimen." Judgement comment: blinding of key personnel described.
Blinding of participants and personnel (performance bias) Objective outcomes	Low risk	Quote: not applicable Judgement comment: objective outcome for personnel, so low risk of bias regardless of quality of blinding.
Blinding of outcome assessment (detection bias) Subjective outcomes	Unclear risk	Quote: not applicable Judgement comment: no description of outcome assessor blinding.
Blinding of outcome assessment (detection bias) Objective outcomes	Low risk	Quote: not applicable Judgement comment: objective outcome for outcome assessor, so low risk of bias regardless of quality of blinding.
Incomplete outcome data (attrition bias) All outcomes	Low risk	Quote: not applicable Judgement comment: all outcome data reported.
Selective reporting (reporting bias)	High risk	Quote: not applicable Judgement comment: one or more outcomes of interest were reported incompletely so that they cannot be entered into a meta‐analysis.
Other bias	Unclear risk	Quote: not applicable Judgement comment: insufficient information to permit judgement.

**Table:** Janssens 1994

***Study characteristics***		
Methods	**Study design:** RCT **Intention‐to‐treat analysis:** not reported **Duration of study:** not reported **Power calculation reached:** not reported **Transfusion strategy:** packed red blood cells were transfused to maintain a haematocrit of 30% **Was the trial stopped early:** no	
Participants	**Baseline characteristics** Placebo *Age (years) (mean SD)*: 65.3 (15.3) *Ethnicity*: not reported *Gender (males, females)*: 6/20 M (30%); 14/20 F (70%) *Length of surgery (minutes) (mean SD)*: 176 (32) *Proportion of participants on anticoagulants prior to surgery (n/N, %)*: not reported *Incidence of preoperative anaemia (n/N, %)*: not reported *Co‐morbidities (n/N, %)*: not reported *ASA 1 (n/N, %)*: not reported *ASA 2 (n/N, %)*: not reported *ASA 3 (n/N, %)*: not reported *ASA 4 (n/N, %)*: not reported *Number of participants randomised*: 20 *Number of participants receiving treatment*: 20 *Number of participants analysed*: 20 *Dropout rate*: 0 Aprotinin *Age (years) (mean SD)*: 64.9 (13.2) *Ethnicity*: not reported *Gender (males, females)*: 10/20 M (50%); 10/20 F (50%) *Length of surgery (minutes) (mean SD)*: 169 (27) *Proportion of participants on anticoagulants prior to surgery (n/N, %)*: not reported *Incidence of preoperative anaemia (n/N, %)*: not reported *Co‐morbidities (n/N, %)*: not reported *ASA 1 (n/N, %)*: not reported *ASA 2 (n/N, %)*: not reported *ASA 3 (n/N, %)*: not reported *ASA 4 (n/N, %)*: not reported *Number of participants randomised*: 20 *Number of participants receiving treatment*: 20 *Number of participants analysed*: 20 *Dropout rate*: 0 Overall *Age (years) (mean SD)*: not reported *Ethnicity*: not reported *Gender (males, females)*: not reported *Length of surgery (minutes)*: not reported *Proportion of participants on anticoagulants prior to surgery (n/N, %)*: not reported *Incidence of preoperative anaemia (n/N, %)*: not reported *Co‐morbidities (n/N, %)*: not reported *ASA 1 (n/N, %)*: not reported *ASA 2 (n/N, %)*: not reported *ASA 3 (n/N, %)*: not reported *ASA 4 (n/N, %)*: not reported *Number of participants randomised*: 40 *Number of participants receiving treatment*: not reported *Number of participants analysed*: not reported *Dropout rate*: not reported **Inclusion criteria:** patients scheduled for primary elective total hip replacement **Exclusion criteria:** 1) known allergy to aprotinin, 2) preoperative renal or hepatic failure, 3) uncontrolled hypertension (i.e. diastolic blood pressure equal or greater than 100 mmHg), 4) clinical cardiac or pulmonary failure, 5) preoperative coagulopathy suspected by clinical history or preoperative blood coagulation tests (platelet count, prothrombin time and activated partial thromboplastin time (aPTT)) **If TKR, is tourniquet used:** not applicable **Indication for surgery:** NR **Type of anaesthetic:** general **Type of surgery:** primary THR	
Interventions	**Intervention characteristics** Placebo Saline (10,000 kallikrein inactivator units (KIU)/mL) given as a bolus injection of 2 x 10(6) KIU over 30 minutes followed by an infusion of 5 x 10(5) KIU/h until the end of surgery, with a maximum dose of 3.5 x 10(6) KIU Placebo, IV, mL, intraop, bolus + infusion until the end of surgery Aprotinin Preservative free aprotinin (10,000 kallikrein inactivator units (KIU)/mL) given as a bolus injection of 2 x 10^6 KIU over 30 min followed by an infusion of 5 x 10^5 KIU/h until the end of surgery with a maximum dose of 3.5 x 10 ^6 KIU Aprotinin, IV, 2 x 10^6 KIU, intraop bolus + 5 x 10^5 KIU/h infusion (MAX 3.5 x 10 ^6 KIU total) until the end of surgery	
Outcomes	*Primary outcomes:* Blood loss Transfusion requirements *Secondary outcome:* Not reported	
Notes	**Sponsorship source:** not reported **Country:** Belgium **Setting:** single‐centre **Comments:** none **Author's name:** M Janssens **Institution:** University Hospital of Liege **Email:** not reported **Address:** Department of Anesthesiology, University Hospital of Liege, Domaine du Sart Tilman, B 4000 Liege, Belgium **Native language of paper:** English **Reference type:** full text (1) **Trial registration number:** not applicable, but when checked, trial registration number not found **Was it translated for this review:** no	
***Risk of bias***		
**Bias**	**Authors' judgement**	**Support for judgement**
Random sequence generation (selection bias)	Unclear risk	Quote: "randomly" Judgement comment: insufficient information to permit judgement.
Allocation concealment (selection bias)	Unclear risk	Quote: not applicable Judgement comment: insufficient information to permit judgement.
Blinding of participants and personnel (performance bias) Subjective outcomes	Unclear risk	Quote: Not applicable Judgement Comment: Insufficient information to permit judgement about blinding of participants.
Blinding of participants and personnel (performance bias) Objective outcomes	Low risk	Quote: not applicable Judgement comment: objective outcome for personnel, so low risk of bias regardless of quality of blinding.
Blinding of outcome assessment (detection bias) Subjective outcomes	Unclear risk	Quote: not applicable Judgement comment: insufficient information to permit judgement about blinding of participants.
Blinding of outcome assessment (detection bias) Objective outcomes	Low risk	Quote: not applicable Judgement comment: objective outcome for assessor, so low risk of bias regardless of quality of blinding
Incomplete outcome data (attrition bias) All outcomes	Low risk	Quote: nt applicable Judgement comment: no missing outcome data.
Selective reporting (reporting bias)	High risk	Quote: not applicable Judgement comment: one or more outcomes of interest in the review are reported incompletely so that they cannot be entered into a meta‐analysis, e.g. proportion of people transfused in the placebo group reported but not in the aprotinin group.
Other bias	Unclear risk	Quote: not applicable Judgement comment: insufficient information to assess whether an important risk of bias exists ‐ no funding or sponsorship declared.

**Table:** Jeserschek 2003

***Study characteristics***		
Methods	**Study design:** RCT **Intention‐to‐treat analysis:** yes **Duration of study:** not reported **Power calculation reached:** yes **Transfusion strategy:** the intraoperative transfusion of red blood cells (RBC) depended on the estimation of the blood loss by the anaesthetist. **Was the trial stopped early:** no	
Participants	**Baseline characteristics** Placebo *Age (years) (mean SD)*: 74 (6.7) *Ethnicity*: not reported *Gender (males, females)*: 5/8 M (62.5%); 3/8 F (37.5%) *Length of surgery (minutes)*: not reported *Proportion of participants on anticoagulants prior to surgery (n/N, %)*: not reported *Incidence of preoperative anaemia (n/N, %)*: not reported *Co‐morbidities (n/N, %)*: not reported *ASA 1 (n/N, %)*: not reported *ASA 2 (n/N, %)*: not reported *ASA 3 (n/N, %)*: not reported *ASA 4 (n/N, %)*: not reported *Number of participants randomised*: 8 *Number of participants receiving treatment*: 8 *Number of participants analysed*: 8 *Dropout rate*: 0/8, (0%) Aprotinin *Age (years) (mean SD)*: 67 (12.0) *Ethnicity*: not reported *Gender (males, females)*: 2/8 M (25%); 6/8 F (75%) *Length of surgery (minutes)*: not reported *Proportion of participants on anticoagulants prior to surgery (n/N, %)*: not reported *Incidence of preoperative anaemia (n/N, %)*: not reported *Co‐morbidities (n/N, %)*: not reported *ASA 1 (n/N, %)*: not reported *ASA 2 (n/N, %)*: not reported *ASA 3 (n/N, %)*: not reported *ASA 4 (n/N, %)*: not reported *Number of participants randomised*: 8 *Number of participants receiving treatment*: 8 *Number of participants analysed*: 8 *Dropout rate*: 0/8 (0%) Overall *Age (years) (mean SD)*: not reported *Ethnicity*: not reported *Gender (males, females)*: not reported *Length of surgery (minutes)*: not reported *Proportion of participants on anticoagulants prior to surgery (n/N, %)*: not reported *Incidence of preoperative anaemia (n/N, %)*: not reported *Co‐morbidities (n/N, %)*: not reported *ASA 1 (n/N, %)*: not reported *ASA 2 (n/N, %)*: not reported *ASA 3 (n/N, %)*: not reported *ASA 4 (n/N, %)*: not reported *Number of participants randomised*: 16 *Number of participants receiving treatment*: 16 *Number of participants analysed*: 16 *Dropout rate*: 0 **Inclusion criteria:** aseptic or septic failure of a hip or knee prosthesis or undergoing resection of a soft tissue sarcoma **Exclusion criteria:** 1) known or suspected allergy to aprotinin or previous treatment with the drug **If TKR, is tourniquet used:** not applicable **Indication for surgery:** aseptic or septic failure of a hip or knee prosthesis or undergoing resection of a soft tissue sarcoma **Type of anaesthetic:** not reported **Type of surgery:** revision TKR, revision THR	
Interventions	**Intervention characteristics** Placebo At the beginning of the operation, the patients received either 1 x 10^6 KIU of aprotinin as a loading dose followed by continuous infusion of 500,000 KIU/hour, or the same volume of normal saline. Placebo, IV, intraop, bolus AND placebo, IV, intraop, infusion Aprotinin At the beginning of the operation, the patients received either 1 x 10^6 KIU of aprotinin as a loading dose followed by continuous infusion of 500,000 KIU/hour, or the same volume of normal saline. Aprotinin, IV, 1 x 10^6 KIU intraop, bolus AND aprotinin, IV, 0.5 x 10^6, intraop, infusion	
Outcomes	*Primary outcomes:* Blood loss Transfusion requirements *Secondary outcome:* Not reported	
Notes	**Sponsorship source:** none **Country:** Austria **Setting:** not reported **Comments:** calculated mean age and SD rTHR patients only. Length of surgery and hospital stay includes resection for cancer surgery and therefore not included in our analysis. **Author's name:** R Jeserschek **Institution:** Karl‐Franzens University School of Medicine **Email:** not reported **Address:** University School of Medicine, Auenbruggerplatz 29, AT‐8036 Graz, Austria **Native language of paper:** English **Reference type:** full text (1) **Trial registration number:** not applicable, but when checked, trial registration number not found **Was it translated for this review:** no	
***Risk of bias***		
**Bias**	**Authors' judgement**	**Support for judgement**
Random sequence generation (selection bias)	Unclear risk	Quote: "Randomisation was performed by the head of the pharmacy department (BP)." Judgement comment: insufficient information to permit judgement.
Allocation concealment (selection bias)	Unclear risk	Quote: not applicable Judgement comment: insufficient information to permit judgement.
Blinding of participants and personnel (performance bias) Subjective outcomes	Low risk	Quote: "The surgeons and anaesthetists were blinded as to whether the patients were receiving aprotinin or the placebo." Judgement comment: blinding likely ensured.
Blinding of participants and personnel (performance bias) Objective outcomes	Low risk	Quote: not applicable Judgement comment: objective outcome for personnel and low risk of bias due to adequate personnel blinding.
Blinding of outcome assessment (detection bias) Subjective outcomes	Unclear risk	Quote: "The surgeons and anaesthetists were blinded as to whether the patients were receiving aprotinin or the placebo." Judgement comment: insufficient information to permit judgement.
Blinding of outcome assessment (detection bias) Objective outcomes	Low risk	Quote: not applicable Judgement comment: objective outcome for outcome assessors, so low risk of bias regardless of quality of blinding.
Incomplete outcome data (attrition bias) All outcomes	Low risk	Quote: not applicable Judgement comment: all outcome data presented.
Selective reporting (reporting bias)	Unclear risk	Quote: not applicable Judgement comment: no study protocol available to check pre‐specified outcomes.
Other bias	Unclear risk	Quote: not applicable Judgement comment: no funding declared. Insufficient information to permit judgement.

**Table:** Johansson 2005

***Study characteristics***		
Methods	**Study design:** RCT **Intention‐to‐treat analysis:** per protocol analysis **Duration of study:** 17 **Power calculation reached:** yes **Transfusion strategy:** the guideline threshold for blood transfusion was a Hb level of 90 g/L, with consideration of clinical well‐being **Was the trial stopped early:** no	
Participants	**Baseline characteristics** Placebo *Age (years) (mean SD)*: 68 (8) *Ethnicity*: not reported *Gender (males, females)*: 28/53 M (53%); 25/53 F (47%) *Length of surgery (minutes) (mean SD)*: 89 (20) *Proportion of participants on anticoagulants prior to surgery (n, %)*: 0 *Incidence of preoperative anaemia (n/N, %)*: not reported *Co‐morbidities (n/N, %)*: not reported *ASA 1 (n/N, %)*: not reported *ASA 2 (n/N, %)*: not reported *ASA 3 (n/N, %)*: not reported *ASA 4 (n/N, %)*: not reported *Number of participants randomised*: not reported *Number of participants receiving treatment*: not reported *Number of participants analysed*: 53 *Dropout rate*: not reported TXA *Age (years) (mean SD)*: 69 (7) *Ethnicity*: not reported *Gender (males, females)*: 25/47 M (53%); 22/47 F (47%) *Length of surgery (minutes) (mean SD)*: 84 (22) *Proportion of participants on anticoagulants prior to surgery (n, %)*: 0 *Incidence of preoperative anaemia (n/N, %)*: not reported *Co‐morbidities (n/N, %)*: not reported *ASA 1 (n/N, %)*: not reported *ASA 2 (n/N, %)*: not reported *ASA 3 (n/N, %)*: not reported *ASA 4 (n/N, %)*: not reported *Number of participants randomised*: not reported *Number of participants receiving treatment*: not reported *Number of participants analysed*: 47 *Dropout rate*: not reported Overall *Age (years) (mean SD)*: not reported *Ethnicity*: not reported *Gender (males, females)*: 47/100 M (47%); 53/100 F (53%) *Length of surgery (minutes) (mean SD)*: not reported *Proportion of participants on anticoagulants prior to surgery (n, %)*: 0 *Incidence of preoperative anaemia (n/N, %)*: not reported *Co‐morbidities (n/N, %)*: not reported *ASA 1 (n/N, %)*: not reported *ASA 2 (n/N, %)*: not reported *ASA 3 (n/N, %)*: not reported *ASA 4 (n/N, %)*: not reported *Number of participants randomised*: 119 *Number of participants receiving treatment*: 119 *Number of participants analysed*: 100 *Dropout rate*: 19/119, 16.0% **Inclusion criteria:** 1) planned THA due to osteoarthrosis, 2) no history or laboratory signs of bleeding disorders (activated partial thrombin, prothrombin time and thrombocyte count within standard limits), 3) absence of malignancy and rheumatic joint disease, 4) no consumption of aspirin or NSAIDs within a week before surgery, 5) no history of coagulopathy or thromboembolic events, 6) plasma creatinine levels below 115 µmol/L in men and 100 µmol/L in women **Exclusion criteria:** not reported **If TKR, is tourniquet used:** not applicable **Indication for surgery:** osteoarthrosis **Type of anaesthetic:** spinal **Type of surgery:** primary THR	
Interventions	**Intervention characteristics** Placebo Immediately before the start of the operation, the patients received a bolus infusion of TXA (15 mg/kg) mixed in 100 mL normal saline, or the same volume of placebo. Placebo, IV, 100 mL, preop, single dose TXA Immediately before the start of the operation, the patients received a bolus infusion of TXA (15 mg/kg) mixed in 100 mL normal saline, or the same volume of placebo. TXA, IV, 15 mg/kg, preop, single dose	
Outcomes	*Primary outcomes:* Blood loss Need for blood transfusion Postoperative complications (wound complications and thromboembolic events) *Secondary outcome:* Not reported	
Notes	**Sponsorship source:** non‐pharmaceutical **Country:** Sweden **Setting:** multi‐centre **Comments:** Health Research Council in the South‐East of Sweden **Author's name:** Torsten Johansson **Institution:** University of Linköping **Email:** Torsten.Johansson@lio.se **Address:** Division of Orthopaedics and Sports Medicine, Department of Neuroscience and Locomotion, Faculty of Health Sciences, University of Linköping, Sweden **Native language of paper:** English **Reference type:** full text (1) **Trial registration number:** not applicable, but when checked, trial registration number not found **Was it translated for this review:** no	
***Risk of bias***		
**Bias**	**Authors' judgement**	**Support for judgement**
Random sequence generation (selection bias)	Low risk	Quote: "The contents of the ampoules were randomized by a computer in blocks of 10 (5 TA, 5 saline)." Judgement comment: none
Allocation concealment (selection bias)	Low risk	Quote: "Coded ampoules containing either tranexamic acid (TA) (100 mg/mL Cyklokapron; Pharmacia) or saline as placebo were prepared by Apoteksbolaget, Umeå, Sweden.'' Judgement comment: central allocation
Blinding of participants and personnel (performance bias) Subjective outcomes	Low risk	Quote: "All personnel and patients were blinded as to the treatment until the randomization code was broken, which took place after all patients had been evaluated." Judgement comment: blinding of participants and key study personnel ensured, and unlikely that the blinding could have been broken
Blinding of participants and personnel (performance bias) Objective outcomes	Low risk	Quote: "All personnel and patients were blinded as to the treatment until the randomization code was broken, which took place after all patients had been evaluated." Judgement comment: blinding of participants and key study personnel ensured, and unlikely that the blinding could have been broken
Blinding of outcome assessment (detection bias) Subjective outcomes	Low risk	Quote: "All personnel and patients were blinded as to the treatment until the randomization code was broken, which took place after all patients had been evaluated." Judgement comment: blinding of participants and key study personnel ensured, and unlikely that the blinding could have been broken
Blinding of outcome assessment (detection bias) Objective outcomes	Low risk	Quote: "All personnel and patients were blinded as to the treatment until the randomization code was broken, which took place after all patients had been evaluated." Judgement comment: blinding of participants and key study personnel ensured, and unlikely that the blinding could have been broken
Incomplete outcome data (attrition bias) All outcomes	High risk	Quote: "Before the randomization code was broken, 19 patients were excluded due to violation of the study plan: 11 patients had not received drains or the drains were removed too early, 6 had been given drugs that could have influenced the blood loss, important data was missing for one patient, and 1 patient had been operated bilaterally." Judgement comment: per protocol analysis
Selective reporting (reporting bias)	Unclear risk	Quote: not applicable Judgement comment: the study protocol is not available and therefore unable to assess whether all the pre‐specified outcomes of interest in the review were reported.
Other bias	Unclear risk	Quote: not applicable Judgement comment: insufficient information to assess whether an important risk of bias exists.

**Table:** Jules‐Elysee 2019

***Study characteristics***		
Methods	**Study design:** RCT **Intention‐to‐treat analysis:** yes **Duration of study:** 12 months **Power calculation reached:** yes **Transfusion strategy:** not reported **Was the trial stopped early:** no	
Participants	**Baseline characteristics** TXA, IV *Age (years) (mean SD)*: 65.6 (8.4) *Ethnicity*: Hispanic or Latino: 0/31, 0%; not Hispanic or Latino 31/31, 100%; unknown or not reported 0/31, 0% *Gender (males, females)*: 11/31 M (35.5%); 20/31 F (64.5%) *Length of surgery (minutes) (mean SD)*: not reported *Proportion of participants on anticoagulants prior to surgery (n/N, %)*: not reported *Incidence of preoperative anaemia (n/N, %)*: not reported *Co‐morbidities (n/N, %)*: not reported *ASA 1 (n/N, %)*: not reported *ASA 2 (n/N, %)*: 28/31, 90.3% *ASA 3 (n/N, %)*: 3/31, 9.7% *ASA 4 (n/N, %)*: 0/31, 0% *Number of participants randomised*: 33 *Number of participants receiving treatment*: 32 *Number of participants analysed*: 31 *Dropout rate*: 2/33, 6.1% TXA, topical *Age (years) (mean SD)*: 65.0 (6.9) *Ethnicity*: Hispanic or Latino: 4/32, 12.5%; not Hispanic or Latino 28/32, 87.5%; unknown or not reported 0/32, 0% *Gender (males, females)*: 20/32 M (62.5%); 12/32 F (37.5%) *Length of surgery (minutes) (mean SD)*: not reported *Proportion of participants on anticoagulants prior to surgery (n, %)*: not reported *Incidence of preoperative anaemia (n/N, %)*: not reported *Co‐morbidities (n/N, %)*: not reported *ASA 1 (n/N, %)*: 0/32, 0% *ASA 2 (n/N, %)*: 32/32, 100% *ASA 3 (n/N, %)*: 0/32, 0% *ASA 4 (n/N, %)*: 0/32, 0% *Number of participants randomised*: 33 *Number of participants receiving treatment*: 32 *Number of participants analysed*: 32 *Dropout rate*: 1/33, 3.03% Overall *Age (years) (mean SD)*: 65.3 (7.6) *Ethnicity*: Hispanic or Latino: 4/63, 6.3%; not Hispanic or Latino 59/63, 93.7%; unknown or not reported 0/63, 0% *Gender (males, females)*: 31/63 M (49.2%); 32/63 F (50.8%) *Length of surgery (minutes) (mean SD)*: not reported *Proportion of participants on anticoagulants prior to surgery (n, %)*: not reported *Incidence of preoperative anaemia (n/N, %)*: not reported *Co‐morbidities (n/N, %)*: not reported *ASA 1 (n/N, %)*: 0/63, 0% *ASA 2 (n/N, %)*: 60/63, 95.2% *ASA 3 (n/N, %)*: 3/63, 4.8% *ASA 4 (n/N, %)*: 0/63, 0% *Number of participants randomised*: 66 *Number of participants receiving treatment*: 64 *Number of participants analysed*: 63 *Dropout rate*: 3/66, 4.5% **Inclusion criteria:** 1) patients undergoing primary unilateral total knee replacement with a participating surgeon, 2) patients aged 18 to 80 **Exclusion criteria:** 1) all patients on steroid therapy regardless of dose, duration or treatment or those requiring stress‐dose steroids preoperatively, 2) patients who will require postoperative use of Coumadin, Xarelto or Plavix, 3) use of non‐steroidal anti‐inflammatory drugs (NSAIDs) within 1 week of surgery, 4) hypersensitivity to tranexamic acid, 5) renal dysfunction (creatinine clearance < 40 mL/min), 6) hepatic dysfunction (AST or ALT 2x upper limit of normal), 7) cardiac exclusions: coronary stent, history of myocardial infarction, positive stress test, atrial fibrillation, advanced coronary artery disease, 8) advanced chronic obstructive pulmonary disease or advanced interstitial lung disease, 9) history of venous thromboembolism, 10) hypercoagulability (e.g. antiphospholipid syndrome, genetic hypercoagulability with or without prior venous thromboembolism), 11) history of stroke or transient ischaemic attack **If TKR, is tourniquet used:** yes **Indication for surgery:** osteoarthritis **Type of anaesthetic:** spinal‐epidural anaesthesia **Type of surgery:** primary TKA	
Interventions	**Intervention characteristics** TXA, IV Prior to tourniquet inflation, the patients in the IV group received 1.0 g of IV TXA and the topical group received 100 mL of an IV placebo (saline solution). Three hours after the first administration of the IV TXA or placebo, an identical preparation was administered in the post‐anaesthesia care unit (PACU). Five minutes prior to final tourniquet release, the topical group received 3.0 g of TXA in 75 mL of saline solution and the IV group received 75 mL of saline solution directly on the wound. TXA, IV, 1 g, intraop + 1 g, postop, placebo, topical, 75 cc, postop TXA, Topical Patients will receive 3 g tranexamic acid in 75 mL solution topically in the operating room, approximately 5 minutes before the tourniquet is released. It will sit for 5 minutes before the solution is suctioned off by the surgeon. They will also receive 2 intravenous saline solutions: one in the operating room before inflation of the tourniquet, and one in the post‐anaesthesia care unit 3 hours after the first solution was given. TXA, topical, 3 g, intraop, + placebo, IV, intraop, postop	
Outcomes	*Primary outcome:* Level of PAP complex in plasma of peripheral blood 4 hours after tourniquet release *Secondary outcomes*: PF1.2 IL‐6 TXA levels in collected peripheral and wound blood Total amount of drainage Total amount of estimated blood loss over the course of the hospital stay Total hidden amount of blood loss over the course of the hospital stay The time to discharge from physical therapy (PT) Length of the hospital stay Haemoglobin and haematocrit levels Units of blood transfused were measured until hospital discharge Prevalence of thrombosis was also assessed.	
Notes	**Sponsorship source:** non‐pharmaceutical (Hospital for Special Surgery, New York) **Country:** USA **Setting:** single‐centre **Comments:** 1) data given for DVT/ PE, not split individually for DVT and PE, so data not extracted **Author's name:** K Jules‐Elysee **Institution:** Hospital for Special Surgery **Email:** JulesElyseeK@HSS.EDU **Address:** Department of Anesthesiology, Critical Care & Pain Management, Hospital for Special Surgery, New York, NY **Native language of paper:** English **Reference type:** full text (1), trial registration (1) **Trial registration number:** NCT02540226 **Was it translated for this review:** no	
***Risk of bias***		
**Bias**	**Authors' judgement**	**Support for judgement**
Random sequence generation (selection bias)	Low risk	Quote: ''Patients were randomized in a 1:1 ratio in size‐4 and 6 blocks, via a computer‐generated randomization schedule, to receive IV or topical TXA.'' Judgement comment: adequate method of sequence generation with computer‐generated random numbers.
Allocation concealment (selection bias)	Unclear risk	Quote: not applicable Judgement comment: method of allocation concealment not described.
Blinding of participants and personnel (performance bias) Subjective outcomes	Low risk	Quote: "Patients, surgeons, anesthesiologists, nurses, and research assistants collecting data were blinded to group allocation." Judgement comment: trial registration says that participant, care provider and investigator were "masked".
Blinding of participants and personnel (performance bias) Objective outcomes	Low risk	Quote: "Patients, surgeons, anesthesiologists, nurses, and research assistants collecting data were blinded to group allocation." Judgement comment: trial registration says that participant, care provider and investigator were "masked". The paper also confirms this. Objective outcome for personnel, so low risk of bias regardless of quality of blinding.
Blinding of outcome assessment (detection bias) Subjective outcomes	Low risk	Quote: not applicable Judgement comment: trial registration says that outcome assessors were "masked". Research assistants collecting data were blinded. Subjective outcome for personnel and low risk of bias due to adequate personnel blinding.
Blinding of outcome assessment (detection bias) Objective outcomes	Low risk	Quote: not applicable Judgement comment: trial registration says that outcome assessors were "masked". Research assistants collecting data were blinded. Objective outcome for personnel, so low risk of bias regardless of quality of blinding.
Incomplete outcome data (attrition bias) All outcomes	Low risk	Quote: not applicable Judgement comment: no obvious outcome data missing.
Selective reporting (reporting bias)	Low risk	Quote: not applicable Judgement comment: all outcomes planned in the protocol or prospective trial registration are reported.
Other bias	Low risk	Quote: not applicable Judgement comment: no other concerns such as early stopping or imbalanced study arms.

**Table:** Kakar 2009 Bilateral TKR

***Study characteristics***		
Methods	**Study design:** RCT **Intention‐to‐treat analysis:** not reported **Duration of study:** not reported **Power calculation reached:** not reported **Transfusion strategy:** before the surgery, Hb transfusion trigger point was determined for each patient according to the following criteria: for patients over 60 yr and associated cardiopulmonary disease the transfusion trigger was 10 g dL−1, whereas for other patients, the transfusion trigger was 8 g dL–1. **Was the trial stopped early:** no	
Participants	**Baseline characteristics** Saline Uni. (CU) *Age (years) (mean SD)*: 66.2 (4.8) *Ethnicity*: not reported *Gender (males, females)*: 4/12 M (33%); 8/12 F (67%) *Length of surgery (minutes) (mean SD)*: 92.1 (10.8) *Proportion of participants on anticoagulants prior to surgery (n/N, %)*: 0 *Incidence of preoperative anaemia (n/N, %)*: not reported *Co‐morbidities (n/N, %)*: not reported *ASA 1 (n/N, %)*: not reported *ASA 2 (n/N, %)*: not reported *ASA 3 (n/N, %)*: not reported *ASA 4 (n/N, %)*: not reported *Number of participants randomised*: 12 *Number of participants receiving treatment*: 12 *Number of participants analysed*: 12 *Dropout rate*: 0 Saline Bi. (CB) *Age (years) (mean SD)*: 67.15 (6.9) *Ethnicity*: not reported *Gender (males, females)*: 3/13 M (23%); 10/13 F (77%) *Length of surgery (minutes) (mean SD)*: 152 (17.3) *Proportion of participants on anticoagulants prior to surgery (n/N, %)*: not reported *Incidence of preoperative anaemia (n/N, %)*: not reported *Co‐morbidities (n/N, %)*: not reported *ASA 1 (n/N, %)*: not reported *ASA 2 (n/N, %)*: not reported *ASA 3 (n/N, %)*: not reported *ASA 4 (n/N, %)*: not reported *Number of participants randomised*: 13 *Number of participants receiving treatment*: 13 *Number of participants analysed*: 13 *Dropout rate*: 0 TXA Uni. (TU) *Age (years) (mean SD)*: 62.4 (9.4) *Ethnicity*: not reported *Gender (males, females)*: 3/12 M (25%); 9/12 F (75%) *Length of surgery (minutes) (mean SD)*: 96.8 (17.7) *Proportion of participants on anticoagulants prior to surgery (n/N, %)*: not reported *Incidence of preoperative anaemia (n/N, %)*: not reported *Co‐morbidities (n/N, %)*: not reported *ASA 1 (n/N, %)*: not reported *ASA 2 (n/N, %)*: not reported *ASA 3 (n/N, %)*: not reported *ASA 4 (n/N, %)*: not reported *Number of participants randomised*: 12 *Number of participants receiving treatment*: 12 *Number of participants analysed*: 12 *Dropout rate*: 0 TXA Bi. (TB) *Age (years) (mean SD)*: 63.13 (16.8) *Ethnicity*: not reported *Gender (males, females)*: 4/13 M (31%); 9/13 F (69%) *Length of surgery (minutes) (mean SD)*: 154 (11.5) *Proportion of participants on anticoagulants prior to surgery (n/N, %)*: not reported *Incidence of preoperative anaemia (n/N, %)*: not reported *Co‐morbidities (n/N, %)*: not reported *ASA 1 (n/N, %)*: not reported *ASA 2 (n/N, %)*: not reported *ASA 3 (n/N, %)*: not reported *ASA 4 (n/N, %)*: not reported *Number of participants randomised*: 13 *Number of participants receiving treatment*: 13 *Number of participants analysed*: 13 *Dropout rate*: 0 Overall *Age (years) (mean SD)*: not reported *Ethnicity*: not reported *Gender (males, females)*: not reported *Length of surgery (minutes)*: not reported *Proportion of participants on anticoagulants prior to surgery (n/N, %)*: not reported *Incidence of preoperative anaemia (n/N, %)*: not reported *Co‐morbidities (n/N, %)*: not reported *ASA 1 (n/N, %)*: not reported *ASA 2 (n/N, %)*: not reported *ASA 3 (n/N, %)*: not reported *ASA 4 (n/N, %)*: not reported *Number of participants randomised*: not reported *Number of participants receiving treatment*: not reported *Number of participants analysed*: not reported *Dropout rate*: not reported **Inclusion criteria:** primary cemented total knee arthoplasties (both unilateral (U/L) and bilateral (B/L)) **Exclusion criteria:** patients were excluded if they had one of the following criteria: 1) known or suspected allergy to medications used (tax, local anaesthetics, midazolam, pethidine, propofol), 2) inherited or acquired haemostatic diseases, 3) abnormal coagulation screening tests (platelet count, prothrombin time, activated partial thromboplastin time), 4) ingestion of aspirin or other nonsteroidal anti‐inflammatory drugs within seven days of surgery, 5) renal or hepatic insufficiency, 6) pregnancy, 7) history of deep venous thrombosis (DVT) or pulmonary embolism or history of ocular pathology or ophthalmological procedure other than corrective lenses **If TKR, is tourniquet used:** yes **Indication for surgery:** not reported **Type of anaesthetic:** spinal and epidural **Type of surgery:** primary unilateral TKR and bilateral TKR	
Interventions	**Intervention characteristics** Saline Uni. (CU) Patients in Group CU and CB received an equivalent volume of physiologic saline Placebo, IV, intraop, infusion (unilateral) Saline Bi. (CB) Patients in Group CU and CB received an equivalent volume of physiologic saline Placebo, IV, intraop, infusion (bilateral) TXA Uni. (TU) In Group TU and TB, tranexamic acid was given immediately before inflation of the tourniquet. After a test dose of 1 mL, patients received a dose of 10 mg/kg–1 IV followed by an infusion of 1 mg/kg–1, 1 hr–1 until skin closure. TXA, IV, 10 mg/kg intraop, 1 mg/kg intraop infusion (unilateral) TXA Bi. (TB) In Group TU and TB, tranexamic acid was given immediately before inflation of the tourniquet. After a test dose of 1 mL, patients received a dose of 10 mg/kg–1 IV followed by an infusion of 1 mg/kg–1, 1 hr–1 until skin closure. TXA, IV, 10 mg/kg intraop, 1 mg/kg intraop infusion (bilateral)	
Outcomes	*Primary outcomes:* Blood loss Postoperative blood transfusions Incidence of deep vein thrombosis Coagulation abnormality *Secondary outcome:* Not reported	
Notes	**Sponsorship source:** not reported **Country:** India **Setting:** not reported **Comments:** none **Author's name:** PN Kakar **Institution:** Fortis Hospital **Email:** pn_kakar@hotmail.com **Address:** Head of the Department of Anesthesia, Fortis Hospital, Shalimar Bagh, New Delhi **Native language of paper:** English **Reference type:** full text (1) **Trial registration number:** not applicable, but when checked, trial registration number not found **Was it translated for this review?:** no	
***Risk of bias***		
**Bias**	**Authors' judgement**	**Support for judgement**
Random sequence generation (selection bias)	Unclear risk	Quote: not applicable Judgement comment: insufficient information to assess sequence generation.
Allocation concealment (selection bias)	Unclear risk	Quote: not applicable Judgement comment: insufficient information about the allocation concealment to permit judgement.
Blinding of participants and personnel (performance bias) Subjective outcomes	Low risk	Quote: not applicable Judgement comment: the anaesthetist, surgeon and the observer were blinded to the study drug and mention of double‐blinding in abstract. Blinding probably performed.
Blinding of participants and personnel (performance bias) Objective outcomes	Low risk	Quote: not applicable Judgement comment: objective outcome for personnel, so low risk of bias regardless of quality of blinding.
Blinding of outcome assessment (detection bias) Subjective outcomes	Low risk	Quote: "The anaesthetist, surgeon and the observer were blinded to the study drug" Judgement comment: assessor probably blinded.
Blinding of outcome assessment (detection bias) Objective outcomes	Low risk	Quote: not applicable Judgement comment: objective outcome for personnel, so low risk of bias regardless of quality of blinding.
Incomplete outcome data (attrition bias) All outcomes	Low risk	Quote: not applicable Judgement comment: no missing data.
Selective reporting (reporting bias)	Unclear risk	Quote: not applicable Judgement comment: no study protocol available; not clear if all the study's pre‐specified outcomes reported.
Other bias	Unclear risk	Quote: not applicable Judgement comment: insufficient information to assess whether an important risk of bias exists ‐ no funding or sponsorship declared.

**Table:** Kakar 2009 Unilateral TKR

***Study characteristics***		
Methods	**Study design:** RCT **Intention‐to‐treat analysis:** not reported **Duration of study:** not reported **Power calculation reached:** not reported **Transfusion strategy:** before the surgery, Hb transfusion trigger point was determined for each patient according to the following criteria: for patients over 60 yr and associated cardiopulmonary disease the transfusion trigger was 10 g dL−1 whereas for other patients, the transfusion trigger was 8 g dL–1. **Was the trial stopped early:** no	
Participants	**Baseline characteristics** Saline Uni. (CU) *Age (years) (mean SD)*: 66.2 (4.8) *Ethnicity*: not reported *Gender (males, females)*: 4/12 M (33%); 8/12 F (67%) *Length of surgery (minutes) (mean SD)*: 92.1 (10.8) *Proportion of participants on anticoagulants prior to surgery (n/N, %)*: 0 *Incidence of preoperative anaemia (n/N, %)*: not reported *Co‐morbidities (n/N, %)*: not reported *ASA 1 (n/N, %)*: not reported *ASA 2 (n/N, %)*: not reported *ASA 3 (n/N, %)*: not reported *ASA 4 (n/N, %)*: not reported *Number of participants randomised*: 12 *Number of participants receiving treatment*: 12 *Number of participants analysed*: 12 *Dropout rate*: 0 Saline Bi. (CB) *Age (years) (mean SD)*: 67.15 (6.9) *Ethnicity*: not reported *Gender (males, females)*: 3/13 M (23%); 10/13 F (77%) *Length of surgery (minutes) (mean SD)*: 152 (17.3) *Proportion of participants on anticoagulants prior to surgery (n/N, %)*: not reported *Incidence of preoperative anaemia (n/N, %)*: not reported *Co‐morbidities (n/N, %)*: not reported *ASA 1 (n/N, %)*: not reported *ASA 2 (n/N, %)*: not reported *ASA 3 (n/N, %)*: not reported *ASA 4 (n/N, %)*: not reported *Number of participants randomised*: 13 *Number of participants receiving treatment*: 13 *Number of participants analysed*: 13 *Dropout rate*: 0 TXA Uni. (TU) *Age (years) (mean SD)*: 62.4 (9.4) *Ethnicity*: not reported *Gender (males, females)*: 3/12 M (25%); 9/12 F (75%) *Length of surgery (minutes) (mean SD)*: 96.8 (17.7) *Proportion of participants on anticoagulants prior to surgery (n/N, %)*: not reported *Incidence of preoperative anaemia (n/N, %)*: not reported *Co‐morbidities (n/N, %)/*: not reported *ASA 1 (n/N, %)*: not reported *ASA 2 (n/N, %)*: not reported *ASA 3 (n/N, %)*: not reported *ASA 4 (n/N, %)*: not reported *Number of participants randomised*: 12 *Number of participants receiving treatment*: 12 *Number of participants analysed*: 12 *Dropout rate*: 0 TXA Bi. (TB) *Age (years) (mean SD)*: 63.13 (16.8) *Ethnicity*: not reported *Gender (males, females)*: 4/13 M (31%); 9/13 F (69%) *Length of surgery (minutes) (mean SD)*: 154 (11.5) *Proportion of participants on anticoagulants prior to surgery (n/N, %)*: not reported *Incidence of preoperative anaemia (n/N, %)*: not reported *Co‐morbidities (n/N, %)*: not reported *ASA 1 (n/N, %)*: not reported *ASA 2 (n/N, %)*: not reported *ASA 3 (n/N, %)*: not reported *ASA 4 (n/N, %)*: not reported *Number of participants randomised*: 13 *Number of participants receiving treatment*: 13 *Number of participants analysed*: 13 *Drop out rate*: 0 Overall *Age (years) (mean SD)*: not reported *Ethnicity*: not reported *Gender (males, females)*: not reported *Length of surgery (minutes)*: not reported *Proportion of participants on anticoagulants prior to surgery (n/N, %)*: not reported *Incidence of preoperative anaemia (n/N, %)*: not reported *Co‐morbidities (n/N, %)*: not reported *ASA 1 (n/N, %)*: not reported *ASA 2 (n/N, %)*: not reported *ASA 3 (n/N, %)*: not reported *ASA 4 (n/N, %)*: not reported *Number of participants randomised*: not reported *Number of participants receiving treatment*: not reported *Number of participants analysed*: not reported *Dropout rate*: not reported **Inclusion criteria:** primary cemented total knee arthoplasties (both unilateral (U/L) and bilateral (B/L)) **Exclusion criteria:** patients were excluded if they had one of the following criteria: 1) known or suspected allergy to medications used (tax, local anaesthetics, midazolam, pethidine, propofol), 2) inherited or acquired haemostatic diseases, 3) abnormal coagulation screening tests (platelet count, prothrombin time, activated partial thromboplastin time), 4) ingestion of aspirin or other nonsteroidal anti‐inflammatory drugs within seven days of surgery, 5) renal or hepatic insufficiency, 6) pregnancy, 7) history of deep venous thrombosis (DVT) or pulmonary embolism or history of ocular pathology or ophthalmological procedure other than corrective lenses **If TKR, is tourniquet used:** yes **Indication for surgery:** not reported **Type of anaesthetic:** spinal and epidural **Type of surgery:** primary unilateral TKR and bilateral TKR	
Interventions	**Intervention characteristics** Saline Uni. (CU) Patients in Group CU and CB received an equivalent volume of physiologic saline Placebo, IV, intraop, infusion (unilateral) Saline Bi. (CB) Patients in Group CU and CB received an equivalent volume of physiologic saline Placebo, IV, intraop, infusion (bilateral) TXA Uni. (TU) In Group TU and TB, tranexamic acid was given immediately before inflation of the tourniquet. After a test dose of 1 mL, patients received a dose of 10 mg/kg–1 IV followed by an infusion of 1 mg/kg–1, 1 hr–1 until skin closure. TXA, IV, 10 mg/kg intraop, 1 mg/kg intraop infusion (unilateral) TXA Bi. (TB) In Group TU and TB, tranexamic acid was given immediately before inflation of the tourniquet. After a test dose of 1 mL, patients received a dose of 10 mg/kg–1 IV followed by an infusion of 1 mg/kg–1, 1 hr–1 until skin closure. TXA, IV, 10 mg/kg intraop, 1 mg/kg intraop infusion (bilateral)	
Outcomes	*Primary outcomes:* Blood loss Postoperative blood transfusions Incidence of deep vein thrombosis Coagulation abnormality *Secondary outcome:* Not reported	
Notes	**Sponsorship source:** not reported **Country:** India **Setting:** not reported **Comments:** none **Author's name:** PN Kakar **Institution:** Fortis Hospital **Email:** pn_kakar@hotmail.com **Address:** Head of the Department of Anesthesia, Fortis Hospital, Shalimar Bagh, New Delhi **Native language of paper:** English **Reference type:** full text (1) **Trial registration number:** not applicable, but when checked, trial registration number not found **Was it translated for this review:** no	
***Risk of bias***		
**Bias**	**Authors' judgement**	**Support for judgement**
Random sequence generation (selection bias)	Unclear risk	Quote: not applicable Judgement comment: insufficient information to assess sequence generation.
Allocation concealment (selection bias)	Unclear risk	Quote: not applicable Judgement comment: insufficient information about the allocation concealment to permit judgement.
Blinding of participants and personnel (performance bias) Subjective outcomes	Low risk	Quote: not applicable Judgement comment: the anaesthetist, surgeon and the observer were blinded to the study drug and mention of double‐blinding in abstract. Blinding probably performed.
Blinding of participants and personnel (performance bias) Objective outcomes	Low risk	Quote: not applicable Judgement comment: objective outcome for personnel, so low risk of bias regardless of quality of blinding
Blinding of outcome assessment (detection bias) Subjective outcomes	Low risk	Quote: "The anaesthetist, surgeon and the observer were blinded to the study drug" Judgement comment: assessor probably blinded.
Blinding of outcome assessment (detection bias) Objective outcomes	Low risk	Quote: not applicable Judgement comment: objective outcome for personnel, so low risk of bias regardless of quality of blinding.
Incomplete outcome data (attrition bias) All outcomes	Low risk	Quote: not applicable Judgement comment: no missing data.
Selective reporting (reporting bias)	Unclear risk	Quote: not applicable Judgement comment: no study protocol available; not clear if all the study's pre‐specified outcomes reported.
Other bias	Unclear risk	Quote: not applicable Judgement comment: insufficient information to assess whether an important risk of bias exists ‐ no funding or sponsorship declared.

**Table:** Kang 2021a

***Study characteristics***		
Methods	**Study design:** RCT **Intention‐to‐treat analysis:** yes **Duration of study:** 8 months (September 2019 and May 2020) + FU (2 weeks) = 8.5 months **Power calculation reached:** no **Transfusion strategy:** blood transfusions were administered to patients with postoperative Hb level of less than 70 g/L or any organ dysfunction related to anemia regardless of Hb level **Was the trial stopped early:** no	
Participants	**Baseline characteristics** TXA, IV 1g pre‐incision, IA 1.5 g intraop, IV 1 g 3 hours postop *Age (years) (mean SD)*: 66.4 (5.9) *Ethnicity*: not reported *Gender (males, females)*: 4/48 M (8.3%); 44/48 F (91.6%) *Length of surgery (minutes) (mean SD)*: not reported *Proportion of participants on anticoagulants prior to surgery (n/N, %)*: 0/48, 0% *Incidence of preoperative anaemia (n/N, %)*: 0/48, 0% *Co‐morbidities (n/N, %)*: rheumatoid arthritis *ASA 1 (n/N, %)*: not reported *ASA 2 (n/N, %)*: not reported *ASA 3 (n/N, %)*: not reported *ASA 4 (n/N, %)*: not reported *Number of participants randomised*: 52 *Number of participants receiving treatment*: 52 *Number of participants analysed*: 48 *Drop out rate*: 4/52, 7.7% TXA, IV 1 g pre incision, IA 1.5 g intraop, IV 1 g 3, 6 and 12 hours postop *Age (years) (mean SD)*: 66.5 (5.5) *Ethnicity*: not reported *Gender (males, females)*: 6/49 M (12.24%); 43/49 F (87.8%) *Length of surgery (minutes) (mean SD)*: not reported *Proportion of participants on anticoagulants prior to surgery (n/N, %)*: not reported *Incidence of preoperative anaemia (n/N, %)*: not reported *Co‐morbidities (n/N, %)*: rheumatoid arthritis *ASA 1 (n/N, %)*: not reported *ASA 2 (n/N, %)*: not reported *ASA 3 (n/N, %)*: not reported *ASA 4 (n/N, %)*: not reported *Number of participants randomised*: 52 *Number of participants receiving treatment*: 52 *Number of participants analysed*: 49 *Drop out rate*: 3/52, 5.8% Overall *Age (years) (mean SD)*: not reported *Ethnicity*: not reported *Gender (males, females)*: not reported *Length of surgery (minutes) (mean SD)*: not reported *Proportion of participants on anticoagulants prior to surgery (n/N, %)*: not reported *Incidence of preoperative anaemia (n/N, %)*: patients with preoperative Hb level < 12 g/dL were administered erythropoietin 10,000 U orally once a day; the Hb level was checked regularly, and the administration was stopped when Hb level reached > 15 g/dL *Co‐morbidities (n/N, %)*: not reported *ASA 1 (n/N, %)*: not reported *ASA 2 (n/N, %)*: not reported *ASA 3 (n/N, %)*: not reported *ASA 4 (n/N, %)*: not reported *Number of participants randomised*: 104 *Number of participants receiving treatment*: 104 *Number of participants analysed*: 97 *Drop out rate*: 7/104, 6.7% **Inclusion criteria:** 1) patients aged 50 to 75 years who underwent primary unilateral TKA for RA **Exclusion criteria:** 1) a diagnosis of other types of arthritis (i.e. not RA), 2) renal dysfunction, 3) severe cardiovascular or cerebrovascular diseases, 4) patients who reported prolonged use of oral anticoagulant drug,s 5) acquired colour vision disorder, 6) active intravascular coagulation patients, 7) history of seizures **If TKR, is tourniquet used:** yes **Indication for surgery:** rheumatoid arthritis **Type of anaesthetic:** general **Type of surgery:** primary TKR	
Interventions	**Intervention characteristics** TXA, IV 1g pre‐incision, IA 1.5 g intraop, IV 1g 3 hours postop IV TXA (1 g) was administered 10 min before skin incision by an anaesthesiologist, and tranexamic acid (1.5 g dissolved in 10 mL of normal saline) was administered via articular injection by a surgeon after cavity suture during the surgery. Patients in group A received one dose of IV TXA (1 g) 3 h postoperatively, while those in group B received three doses of IV TXA (1 g) 3, 6 and 12 h postoperatively. TXA, IV 1 g pre incision, IA 1.5g intraop, IV 1g 3 hours postop TXA, IV 1g pre‐incision, IA 1.5 g intraop, IV 1 g 3, 6 and 12 hours postop IV TXA (1 g) was administered 10 min before skin incision by an anaesthesiologist, and tranexamic acid (1.5 g dissolved in 10 mL of normal saline) was administered via articular injection by a surgeon after cavity suture during the surgery. Group B received 3 doses of IV TXA (1 g) 3, 6 and 12 h postoperatively TXA, IV 1g pre‐incision, IA 1.5 g intraop, IV 1 g 3, 6 and 12 hours postop	
Outcomes	*Primary outcomes:* Perioperative haematocrit (Hct) and Hb levels Coagulation index Renal function Blood loss *Secondary outcome:* Adverse events (e.g. DVT, PE, wound complications, infection and acute renal failure) Transfusion rate Adverse events	
Notes	**Sponsorship source:** non‐pharmaceutical (this study was supported by the Foundation of Health and Family planning Commission of Shanghai, China (Grant No. ZY (2018–2020)‐FWTX‐6023)) **Country:** China **Setting:** single‐centre **Comments:** none **Author's name:** B‐X Kang **Institution:** Shanghai Guanghua Hospital of Integrated Traditional Chinese and Western Medicine **Email:** Lian‐bo Xiao: xiao_lianbo@163.com; Xiao‐jun Gao: 13501735927@139.com **Address:** Shanghai University of Traditional Chinese Medicine, Shanghai Guanghua Hospital of Integrated Traditional Chinese and Western Medicine, Shanghai 200052, CN, China **Native language of paper:** English **Reference type:** full text (1), study protocol (1), trial registration (1) **Trial registration number:** ChiCTR1900025013 **Was it translated for this review:** no	
***Risk of bias***		
**Bias**	**Authors' judgement**	**Support for judgement**
Random sequence generation (selection bias)	Low risk	Quote: "All eligible patients were randomized into two groups using computer‐generated randomization by a statistician who was not involved in the trial." Judgement comment: adequate method of sequence generation with computer‐generated random numbers.
Allocation concealment (selection bias)	Low risk	Quote: "The allocation was concealed in consecutively numbered, sealed, opaque" Judgement comment: envelopes described as sealed, opaque and sequentially numbered.
Blinding of participants and personnel (performance bias) Subjective outcomes	High risk	Quote: "The surgeon, anesthesiologist, and statistician were blinded to the trial." Judgement comment: participants and nurses not blinded to intervention.
Blinding of participants and personnel (performance bias) Objective outcomes	Low risk	Quote: not applicable Judgement comment: objective outcome for personnel, so low risk of bias regardless of quality of blinding.
Blinding of outcome assessment (detection bias) Subjective outcomes	Low risk	Quote: "The surgeon, anesthesiologist, and statistician were blinded to the trial." Judgement comment: none
Blinding of outcome assessment (detection bias) Objective outcomes	Low risk	Quote: not applicable Judgement comment: objective outcome measure, so low risk regardless of blinding.
Incomplete outcome data (attrition bias) All outcomes	Low risk	Quote: not applicable Judgement comment: no obvious outcome data missing.
Selective reporting (reporting bias)	Low risk	Quote: not applicable Judgement comment: all outcomes planned in the protocol or prospective trial registration are reported.
Other bias	Low risk	Quote: not applicable Judgement comment: no other concerns such as early stopping or imbalanced study arms

**Table:** Kang 2021b

***Study characteristics***		
Methods	**Study design:** RCT **Intention‐to‐treat analysis:** yes **Duration of study:** 12 months (May 2019 to May 2020) + 2 weeks (follow‐up) = 12.5 months **Power calculation reached:** not reported **Transfusion strategy:** the criterion for transfusion was a postoperative Hb value of less than 70 g/L in asymptomatic patients or between 70 g/L and 10 g/L in symptomatic patients **Was the trial stopped early:** no	
Participants	**Baseline characteristics** TXA, IV 1 dose postop *Age (years) (mean SD)*: 71.4 (4.0) *Ethnicity*: not reported *Gender (males, females)*: 19/100 M (19%); 81/100 F (81%) *Length of surgery (minutes) (mean SD)*: not reported *Proportion of participants on anticoagulants prior to surgery (n/N, %)*: not reported *Incidence of preoperative anaemia (n/N, %)*: these patients were excluded *Co‐morbidities (n/N, %)*: not reported *ASA 1 (n/N, %)*: not reported *ASA 2 (n/N, %)*: not reported *ASA 3 (n/N, %)*: not reported *ASA 4 (n/N, %)*: not reported *Number of participants randomised*: 100 *Number of participants receiving treatment*: 100 *Number of participants analysed*: 100 *Dropout rate*: 0/100, 0% TXA, IV 2 doses postop *Age (years) (mean SD)*: 71.8 (4.1) *Ethnicity*: not reported *Gender (males, females)*: 20/100 M (20%); 80/100 F (80%) *Length of surgery (minutes) (mean SD)*: not reported *Proportion of participants on anticoagulants prior to surgery (n/N, %)*: not reported *Incidence of preoperative anaemia (n/N, %)*: these patients were excluded *Co‐morbidities (n/N, %)*: not reported *ASA 1 (n/N, %)*: not reported *ASA 2 (n/N, %)*: not reported *ASA 3 (n/N, %)*: not reported *ASA 4 (n/N, %)*: not reported *Number of participants randomised*: 100 *Number of participants receiving treatment*: 100 *Number of participants analysed*: 100 *Dropout rate*: 0/100, 0% TXA, IV 3 doses postop *Age (years) (mean SD)*: 71.3 (4.7) *Ethnicity*: not reported *Gender (males, females)*: 17/100 M (17%); 83/100 F (83%) *Length of surgery (minutes) (mean SD)*: not reported *Proportion of participants on anticoagulants prior to surgery (n/N, %)*: not reported *Incidence of preoperative anaemia (n/N, %)*: these patients were excluded *Co‐morbidities (n/N, %)*: not reported *ASA 1 (n/N, %)*: not reported *ASA 2 (n/N, %)*: not reported *ASA 3 (n/N, %)*: not reported *ASA 4 (n/N, %)*: not reported *Number of participants randomised*: 100 *Number of participants receiving treatment*: 100 *Number of participants analysed*: 100 *Dropout rate*: 0/100, 0% Overall *Age (years) (mean SD)*: not reported *Ethnicity*: not reported *Gender (males, females)*: not reported *Length of surgery (minutes) (mean SD)*: not reported *Proportion of participants on anticoagulants prior to surgery (n/N, %)*: not reported *Incidence of preoperative anaemia (n/N, %)*: these patients were excluded *Co‐morbidities (n/N, %)*: not reported *ASA 1 (n/N, %)*: not reported *ASA 2 (n/N, %)*: not reported *ASA 3 (n/N, %)*: not reported *ASA 4 (n/N, %)*: not reported *Number of participants randomised*: 300 *Number of participants receiving treatment*: 300 *Number of participants analysed*: 300 *Dropout rate*: 0/300, 0% **Inclusion criteria:** 1) patients must have been diagnosed with stage III or IV knee osteoarthritis according to the Kellgren‐Lawrence classification18; 2) patients had undergone unilateral primary TKA by the same surgery team; 3) patients were aged 60 to 80 years **Exclusion criteria:** 1) patients with other types of arthritis; 2) flexion deformity ≥ 30, patients with varus/valgus deformity ≥ 30; 3) patients who underwent bilateral TKA; 4) patients with preoperative anemia (haemoglobin (Hb) < 120 g/L for women, < 130 g/L for men), renal dysfunction or severe cardiovascular or cerebrovascular diseases, and patients with prolonged use of oral anticoagulant drugs **If TKR, is tourniquet used:** yes ‐ tourniquet time: Group A 75.1 ± 7.4 Group B 75.5 ± 7.3 Group C 75.1 ± 7.3 (minutes) **Indication for surgery:** osteoarthritis **Type of anaesthetic:** general **Type of surgery:** unilateral primary TKA	
Interventions	**Intervention characteristics** TXA, IV 1 dose postop One dose (1 g) of IV TXA before skin incision combined with one dose (1.5 g) of intra‐articular tranexamic acid (IA TXA) followed by a single dose of IV TXA (1 g) for 3 h TXA, IV, 1 g, pre‐op + TXA, IA, 1.5 g, pre‐op + TXA, IV, 1 g, postop TXA, IV 2 dose postop One dose (1 g) of IV TXA before skin incision combined with one dose (1.5 g) of intra‐articular tranexamic acid (IA TXA) followed by 2 doses of IV TXA (1 g) for 3 and 6 h TXA, IV, 1 g, pre‐op + TXA, IA, 1.5 g, pre‐op + TXA, IV, 1 g, repeated dose, 3 h and 6 h TXA, IV 3 dose postop One dose (1 g) of IV TXA before skin incision combined with one dose (1.5 g) of intra‐articular tranexamic acid (IA TXA) followed by 3 doses of IV TXA (1 g) for 3, 6 and 12 h TXA, IV, 1 g, pre‐op + TXA, IA, 1.5 g, pre‐op + TXA, IV, 1 g, repeated dose, 3 h, 6 h and 12 h	
Outcomes	*Primary outcomes:* Total blood loss Hidden blood loss Maximum haemoglobin drop Need for blood transfusion *Secondary outcomes:* Mean level of postoperative CRP D‐dimer Complications Adverse events	
Notes	**Sponsorship source:** non‐pharmaceutical (this study was supported by the Foundation of Health and Family Planning Commission of Shanghai (grant no. ZY (2018‐2020)‐FWTX‐6023) **Country:** China **Setting:** single‐centre **Comments:** none **Author's name:** B‐X Kang **Institution:** Shanghai Guanghua Hospital of Integrated Traditional Chinese and Western Medicine **Email:** Lian‐bo Xiao: xiao_lianbo@163.com; Xiao‐xue Hu: ghyyxiaoxue@163.com **Address:** Department of Orthopaedics, Guanghua Hospital Shanghai University of Traditional Chinese Medicine, No. 540 Xinhua Road, Changning District, Shanghai **Native language of paper:** English **Reference type:** full text (1), study protocol (1), trial registration (1) **Trial registration number:** ChiCTR1900022737 **Was it translated for this review:** no	
***Risk of bias***		
**Bias**	**Authors' judgement**	**Support for judgement**
Random sequence generation (selection bias)	Low risk	Quote: "randomized block design" Judgement comment: the statistician generated a random sequence consisting of 300 random numbers in SPSS software to generate a sequence of random numbers, and input the new numbers after grouping, and marked them as group 1, group 2, and group 3.
Allocation concealment (selection bias)	Unclear risk	Quote: not applicable Judgement comment: method of allocation concealment not described.
Blinding of participants and personnel (performance bias) Subjective outcomes	Unclear risk	Quote: not applicable Judgement comment: no description given of personnel blinding.
Blinding of participants and personnel (performance bias) Objective outcomes	Low risk	Quote: not applicable Judgement comment: objective outcome for personnel, so low risk of bias regardless of quality of blinding.
Blinding of outcome assessment (detection bias) Subjective outcomes	Unclear risk	Quote: not applicable Judgement comment: no description given of outcome assessor blinding.
Blinding of outcome assessment (detection bias) Objective outcomes	Low risk	Quote: Not applicable Judgement Comment: Objective outcome for personnel, so low risk of bias regardless of quality of blinding.
Incomplete outcome data (attrition bias) All outcomes	Low risk	Quote: not applicable Judgement comment: no obvious outcome data missing.
Selective reporting (reporting bias)	High risk	Quote: not applicable Judgement comment: more outcomes are reported in the full text than are mentioned in the trial registration (e.g. DVT, PE, transfusions).
Other bias	Low risk	Quote: not applicable Judgement comment: no other concerns such as early stopping or imbalanced study arms.

**Table:** Karnezis 1994 hip

***Study characteristics***		
Methods	**Study design:** RCT **Intention‐to‐treat analysis:** unclear **Duration of study:** 18 months **Power calculation reached:** not reported **Transfusion strategy?:** homologous blood was used when reinfusion of autologous blood was completed and when haematocrit levels were less than 22% to 24%. **Was the trial stopped early:** no	
Participants	**Baseline characteristics** Placebo *Age (years) (mean SD)*: 67 (6.7) *Ethnicity*: not reported *Gender (males, females)*: 14/30 M (47%); 16/30 (53%) *Length of surgery (minutes)*: not reported *Proportion of participants on anticoagulants prior to surgery (n/N, %)*: not reported *Incidence of preoperative anaemia (n/N, %)*: not reported *Co‐morbidities (n/N, %)*: mean ASA (SD): 1.9 (0.5) *ASA 1 (n/N, %)*: not reported *ASA 2 (n/N, %)*: not reported *ASA 3 (n/N, %)*: not reported *ASA 4 (n/N, %)*: not reported *Number of participants randomised*: 30 *Number of participants receiving treatment*: 30 *Number of participants analysed*: 30 *Dropout rate*: 0/30 (0%) Desmopressin *Age (years) (mean SD)*: 65 (7.8) *Ethnicity*: not reported *Gender (males, females)*: 12/26 M (46%); 14/26 F (54%) *Length of surgery (minutes)*: not reported *Proportion of participants on anticoagulants prior to surgery (n/N, %)*: not reported *Incidence of preoperative anaemia (n/N, %)*: not reported *Co‐morbidities (n/N, %)*: mean ASA (SD): 1.9 (0.3) *ASA 1 (n/N, %)*: not reported *ASA 2 (n/N, %)*: not reported *ASA 3 (n/N, %)*: not reported *ASA 4 (n/N, %)*: not reported *Number of participants randomised*: 26 *Number of participants receiving treatment*: 26 *Number of participants analysed*: 26 *Dropout rate*: 0/26 (0%) Overall *Age (years) (mean SD)*: not reported *Ethnicity*: not reported *Gender (males, females)*: not reported *Length of surgery (minutes)*: not reported *Proportion of participants on anticoagulants prior to surgery (n/N, %)*: not reported *Incidence of preoperative anaemia (n/N, %)*: not reported *Co‐morbidities (n/N, %)*: not reported *ASA 1 (n/N, %)*: not reported *ASA 2 (n/N, %)*: not reported *ASA 3 (n/N, %)*: not reported *ASA 4 (n/N, %)*: not reported *Number of participants randomised*: not reported *Number of participants receiving treatment*: not reported *Number of participants analysed*: not reported *Dropout rate*: not reported **Inclusion criteria:** 1) scheduled for a primary total hip or total knee arthroplasty **Exclusion criteria:** 1) history of operative intervention involving the hip or knee, 2) coagulation disorder, 3) coronary artery disease, 4) patients who had received Coumadin (warfarin) or heparin within 7 days before the operation, 5) patients scheduled to have a bilateral total hip or knee arthroplasty or a revision operation **If TKR, is tourniquet used:** tourniquet was used for the TKA group in this study (see Karnezis 1994 knee for details) **Indication for surgery:** end‐stage degenerative osteoarthrosis or rheumatoid arthritis **Type of anaesthetic:** general anaesthetic **Type of surgery:** primary THR and primary TKR. Results for hip surgery are given here. See Karnezis 1994 knee for details of the TKA group in this study.	
Interventions	**Intervention characteristics** Placebo Approximately 30 minutes before complete closure of the wound, a single dose of 0.3 μg per kg of body mass of desmopressin (Roren Pharmaceutical, Fort Washington, Pennsylvania) in 50 mL of physiological saline solution was administered intravenously over a 20‐minute period to the patients in the desmopressin group. The control group received a saline solution placebo in the identical manner. Placebo, IV, intraop (30 mins before wound closure) Desmopressin Approximately 30 minutes before complete closure of the wound, a single dose of 0.3 μg per kg of body mass of desmopressin (Roren Pharmaceutical, Fort Washington, Pennsylvania) in 50 mL of physiological saline solution was administered intravenously over a 20‐minute period to the patients in the desmopressin group. The control group received a saline solution placebo in the identical manner. Desmopressin, IV, 0.3 μg/kg, intraop (30 mins before wound closure)	
Outcomes	*Primary outcomes:* Postoperative bleeding Postoperative transfusion requirements *Secondary outcome:* Not reported	
Notes	**Sponsorship source:** none **Country:** USA **Setting:** single‐centre **Comments:** none **Author's name:** TA Karnezis **Institution:** University of Chicago **Email:** not reported **Address:** Department of Orthopaedic Surgery, Northwestern University 303 East Chicago Avenue, Room 9‐037, Chicago, Illinois 60611‐3008 **Native language of paper:** English **Reference type:** full text (1) **Trial registration number:** not applicable, but when checked, trial registration number not found **Was it translated for this review:** no	
***Risk of bias***		
**Bias**	**Authors' judgement**	**Support for judgement**
Random sequence generation (selection bias)	Low risk	Quote: "After approval by the institutional review board had been obtained and the patients had given informed consent, ninety‐two patients who were scheduled for a primary total hip or total knee arthroplasty were randomly allocated to a control group or to a desmopressin group with use of a randomization table." Judgement comment: reference to random number table.
Allocation concealment (selection bias)	Unclear risk	Quote: not applicable Judgement comment: insufficient information to permit judgement.
Blinding of participants and personnel (performance bias) Subjective outcomes	Low risk	Quote: "All patients, treating physicians. and investigators collecting the data were blinded to the assigned treatment. Only the pharmacist was aware of the treatment group." Judgement comment: blinding of key personnel described.
Blinding of participants and personnel (performance bias) Objective outcomes	Low risk	Quote: not applicable Judgement comment: objective outcome for personnel, so low risk of bias regardless of quality of blinding.
Blinding of outcome assessment (detection bias) Subjective outcomes	Low risk	Quote: "All patients, treating physicians. and investigators collecting the data were blinded to the assigned treatment. Only the pharmacist was aware of the treatment group." Judgement comment: blinding of outcome assessor described.
Blinding of outcome assessment (detection bias) Objective outcomes	Low risk	Quote: not applicable Judgement comment: objective outcome for outcome assessor, so low risk of bias regardless of quality of blinding.
Incomplete outcome data (attrition bias) All outcomes	Low risk	Quote: not applicable Judgement comment: no missing outcome data reported.
Selective reporting (reporting bias)	High risk	Quote: not applicable Judgement comment: one or more outcomes of interest are reported incompletely such that they cannot be entered into the meta‐analysis.
Other bias	Low risk	Quote: not applicable Judgement comment: paper appears to be free of other bias.

**Table:** Karnezis 1994 knee

***Study characteristics***		
Methods	**Study design:** RCT **Intention‐to‐treat analysis:** unclear **Duration of study:** 18+ months **Power calculation reached:** not reported **Transfusion strategy:** homologous blood was used when reinfusion of autologous blood was completed and when haematocrit levels were less than 22% to 24%. **Was the trial stopped early:** no	
Participants	**Baseline characteristics** Placebo *Age (years) (mean SD)*: 66 (9.3) *Ethnicity*: not reported *Gender (males, females)*: 9/19 M (47%); 10/19 F (53%) *Length of surgery (minutes)*: not reported *Proportion of participants on anticoagulants prior to surgery (n/N, %)*: not reported *Incidence of preoperative anaemia (n/N, %)*: not reported *Co‐morbidities (n/N, %)*: mean ASA (SD): 2.1 (0.2) *ASA 1 (n/N, %)*: not reported *ASA 2 (n/N, %)*: not reported *ASA 3 (n/N, %)*: not reported *ASA 4 (n/N, %)*: not reported *Number of participants randomised*: 19 *Number of participants receiving treatment*: 19 *Number of participants analysed*: 19 *Dropout rate*: 0/19 (0%) Desmopressin *Age (years) (mean SD)*: 65 (5.3) *Ethnicity*: not reported *Gender (males, females)*: 7/17 M (41%); 10/17 F (59%) *Length of surgery (minutes)*: not reported *Proportion of participants on anticoagulants prior to surgery (n/N, %)*: not reported *Incidence of preoperative anaemia (n/N, %)*: not reported *Co‐morbidities (n/N, %)*: mean ASA (SD): 2.1 (0.5) *ASA 1 (n/N, %)*: not reported *ASA 2 (n/N, %)*: not reported *ASA 3 (n/N, %)*: not reported *ASA 4 (n/N, %)*: not reported *Number of participants randomised*: 17 *Number of participants receiving treatment*: 17 *Number of participants analysed*: 17 *Dropout rate*: 0/17 (0%) Overall *Age (years) (mean SD)*: not reported *Ethnicity*: not reported *Gender (males, females)*: not reported *Length of surgery (minutes)*: not reported *Proportion of participants on anticoagulants prior to surgery (n/N, %)*: not reported *Incidence of preoperative anaemia (n/N, %)*: not reported *Co‐morbidities (n/N, %)*: not reported *ASA 1 (n/N, %)*: not reported *ASA 2 (n/N, %)*: not reported *ASA 3 (n/N, %)*: not reported *ASA 4 (n/N, %)*: not reported *Number of participants randomised*: not reported *Number of participants receiving treatment*: not reported *Number of participants analysed*: not reported *Dropout rate*: not reported **Inclusion criteria:** 1) scheduled for a primary total hip or total knee arthroplasty **Exclusion criteria:** 1) history of operative intervention involving the hip or knee, 2) coagulation disorder, 3) coronary artery disease, 4) patients who had received Coumadin (warfarin) or heparin within 7 days before the operation, 5) patients scheduled to have a bilateral total hip or knee arthroplasty or a revision operation **If TKR, is tourniquet used:** yes **Indication for surgery:** end‐stage degenerative osteoarthrosis or rheumatoid arthritis **Type of anaesthetic:** general anaesthesia **Type of surgery:** primary THR and primary TKR. Results for knee surgery are given here. See Karnezis 1994 hip for details of the THA group in this study.	
Interventions	**Intervention characteristics** Placebo Approximately 30 minutes before complete closure of the wound, a single dose of 0.3 μg per kg of body mass of desmopressin (Roren Pharmaceutical, Fort Washington, Pennsylvania) in 50 mL of physiological saline solution was administered intravenously over a 20‐minute period to the patients in the desmopressin group. The control group received a saline‐solution placebo in the identical manner. Placebo, IV, intraop (30 mins before wound closure) Desmopressin Approximately 30 minutes before complete closure of the wound, a single dose of 0.3 μg per kg of body mass of desmopressin (Roren Pharmaceutical, Fort Washington, Pennsylvania) in 50 mL of physiological saline solution was administered intravenously over a 20‐minute period to the patients in the desmopressin group. The control group received a saline solution placebo in the identical manner. Desmopressin, IV, 0.3 μg/kg, intraop (30 mins before wound closure)	
Outcomes	*Primary outcomes:* Postoperative bleeding Postoperative transfusion requirements *Secondary outcome:* Not reported	
Notes	**Sponsorship source:** none **Country:** USA **Setting:** single‐centre **Comments:** none **Author's name:** TA Karnezis **Institution:** University of Chicago **Email:** not reported **Address:** Department of Orthopaedic Surgery, Northwestern University 303 East Chicago Avenue, Room 9‐037, Chicago, Illinois 60611‐3008 **Native language of paper:** English **Reference type:** full text (1) **Trial registration number:** not applicable, but when checked, trial registration number not found **Was it translated for this review:** no	
***Risk of bias***		
**Bias**	**Authors' judgement**	**Support for judgement**
Random sequence generation (selection bias)	Low risk	Quote: "After approval by the institutional review board had been obtained and the patients had given informed consent, ninety‐two patients who were scheduled for a primary total hip or total knee arthroplasty were randomly allocated to a control group or to a desmopressin group with use of a randomization table." Judgement comment: use of random number table.
Allocation concealment (selection bias)	Unclear risk	Quote: not applicable Judgement comment: insufficient information to permit judgement.
Blinding of participants and personnel (performance bias) Subjective outcomes	Low risk	Quote: "All patients, treating physicians and investigators collecting the data were blinded to the assigned treatment." Judgement comment: blinding of personnel described.
Blinding of participants and personnel (performance bias) Objective outcomes	Low risk	Quote: not applicable Judgement comment: objective outcome for personnel, so low risk of bias regardless of quality of blinding.
Blinding of outcome assessment (detection bias) Subjective outcomes	Low risk	Quote: "All patients, treating physicians and investigators collecting the data were blinded to the assigned treatment. Only the pharmacist was aware of the treatment group." Judgement comment: blinding of outcome assessor described.
Blinding of outcome assessment (detection bias) Objective outcomes	Low risk	Quote: not applicable Judgement comment: objective outcome for outcome assessor, so low risk of bias regardless of quality of blinding.
Incomplete outcome data (attrition bias) All outcomes	Low risk	Quote: not applicable Judgement comment: no missing data reported.
Selective reporting (reporting bias)	High risk	Quote: not applicable Judgement comment: one or more outcomes of interest were reported incompletely such that they cannot be entered into a meta‐analysis.
Other bias	Low risk	Quote: not applicable Judgement comment: paper appears to be free of other bias.

**Table:** Kayupov 2017a

***Study characteristics***		
Methods	**Study design:** RCT **Intention‐to‐treat analysis:** per protocol **Duration of study:** 6 months **Power calculation reached:** yes **Transfusion strategy:** the transfusion protocol for the study participants was planned for a haemoglobin level of less than 7.0 g/dL. If a transfusion was to be given at a haemoglobin level higher than 7.0 g/dL, it would only be performed secondary to a specific patient history (i.e. cardiac disease) under the request of an internal medicine physician. **Was the trial stopped early:** no	
Participants	**Baseline characteristics** TXA, IV *Age (years) (mean SD)*: 63 (10) *Ethnicity*: not reported *Gender (males, females)*: 11/37 M (30%); 26/37 F (70%) *Length of surgery (minutes) (mean SD)*: 90 (19) *Proportion of participants on anticoagulants prior to surgery (n/N, %)*: not reported *Incidence of preoperative anaemia (n/N, %)*: not reported *Co‐morbidities (n/N, %)*: not reported *ASA 1 (n/N, %)*: 1/37 (2.7%) *ASA 2 (n/N, %)*: 27/37 (73.0%) *ASA 3 (n/N, %)*: 9/37 (24.3%) *ASA 4 (n/N, %)*: 0/37 (0%) *Number of participants randomised*: 38 *Number of participants receiving treatment*: 37 *Number of participants analysed*: 37 *Dropout rate*: 1/37 (2.7%) TXA, oral *Age (years) (mean SD)*: 62 (11) *Ethnicity*: n*ot* reported *Gender (males, females)*: 13/34 M (38%); 21/34 F (62%) *Length of surgery (minutes) (mean SD)*: 82 (14) *Proportion of participants on anticoagulants prior to surgery? (n/N, %)*: not reported *Incidence of preoperative anaemia (n/N, %)*: not reported *Co‐morbidities (n/N, %)*: not reported *ASA 1 (n/N, %)*: 1/34 (2.9%) *ASA 2 (n/N, %)*: 25/32 (78.1%) *ASA 3 (n/N, %)*: 8/34 (23.5%) *ASA 4 (n/N, %)*: 0/34 (0%) *Number of participants randomised*: 40 *Number of participants receiving treatment*: 34 *Number of participants analysed*: 34 *Dropout rate*: 6/40 (15%) Overall *Age (years) (mean SD)*: not reported *Ethnicity*: not reported *Gender (males, female)*: not reported *Length of surgery (minutes) (mean SD)*: not reported *Proportion of participants on anticoagulants prior to surgery (n/N, %)*: not reported *Incidence of preoperative anaemia (n/N, %)*: not reported *Co‐morbidities (n/N, %)*: not reported *ASA 1 (n/N, %)*: not reported *ASA 2 (n/N, %)*: not reported *ASA 3 (n/N, %)*: not reported *ASA 4 (n/N, %)*: not reported *Number of participants randomised*: not reported *Number of participants receiving treatment*: not reported *Number of participants analysed*: not reported *Dropout rate*: not reported **Inclusion criteria:** 1) Patients scheduled to undergo unilateral primary TKA **Exclusion criteria:** 1) known allergy to TXA, 2) history of renal failure or kidney transplant, 3) a history of arterial thromboembolic event (e.g. myocardial infarction, stroke) within the past year, 4) placement of an arterial stent within the past year, 5) history of thromboembolic event, or refusal to receive blood products **If TKR, is tourniquet used:** yes **Indication for surgery:** not reported **Type of anaesthetic:** mixed spinal and general anaesthesia **Type of surgery:** primary TKR	
Interventions	**Intervention characteristics** TXA, IV Patients in the intravenous tranexamic acid group received the standard dosing for our institution of 1 g of tranexamic acid in 10 mL of normal saline solution given as an intravenous bolus immediately prior to incision along with a total dose of 750 mg of ascorbic acid in the form of 3 x 250 mg tablets as a placebo 2 hours prior to the incision TXA, IV, 1 g, intraop (prior to skin incision) AND placebo, oral, preop (2 hours prior to incision) TXA, oral Patients randomised to the oral tranexamic acid group were administered a total oral dose of 1950 mg of tranexamic acid using 3 tablets of 650 mg approximately 2 hours prior to incision and a placebo dose of 10 mL of normal saline solution immediately prior to incision Placebo, IV, intraop (prior to skin incision) AND TXA, 1.95 mg, oral, preop (2 hours prior to incision)	
Outcomes	*Primary outcomes:* Reduction of haemoglobin concentration *Secondary outcomes:* Postoperative calculated blood loss Postoperative calculated haemoglobin loss Transfusion rate Number of blood units transfused Length of hospital stay Incidence of thromboembolic events	
Notes	**Sponsorship source:** non‐pharmaceutical **Country:** USA **Setting:** single‐centre **Comments:** none **Author's name:** YA Fillingham **Institution:** Rush University Medical Center **Email:** not reported **Address:** Department of Orthopaedic Surgery, Rush University Medical Center, Chicago, Illinois **Native language of paper:** English **Reference type:** full text (1) trial registration (1) **Trial registration number:** NCT02233101 **Was it translated for this review:** no	
***Risk of bias***		
**Bias**	**Authors' judgement**	**Support for judgement**
Random sequence generation (selection bias)	Low risk	Quote: "Enrolled patients were randomly allocated between the 2 treatment groups of oral and IV TXA using a random number algorithm to provide a binary output to assign the patient's treatment." Judgement comment: use of a random number table.
Allocation concealment (selection bias)	Unclear risk	Quote: "Assignments of the study participants were prepared by a research assistant and were kept blinded from the study participants and clinical staff involved with decisions regarding study outcomes (ie, ordering transfusion of blood)." Judgement comment: insufficient information to permit judgement.
Blinding of participants and personnel (performance bias) Subjective outcomes	Low risk	Quote: "The patient, surgeon, and clinical staff involved in making decisions with regard to study outcomes such as ordering transfusions were blinded to the route of tranexamic acid administration. This information was linked to a confidential and secure database used for collection and analysis by an independent research coordinator and statistician." Judgement comment: blinding of key personnel ensured.
Blinding of participants and personnel (performance bias) Objective outcomes	Low risk	Quote: not applicable Judgement comment: objective outcome for personnel, so low risk of bias regardless of quality of blinding.
Blinding of outcome assessment (detection bias) Subjective outcomes	Low risk	Quote: "The patient, surgeon, and clinical staff involved in making decisions with regard to study outcomes such as ordering transfusions were blinded to the route of tranexamic acid administration." Judgement comment: blinding of outcome assessors described.
Blinding of outcome assessment (detection bias) Objective outcomes	Low risk	Quote: not applicable Judgement comment: objective outcome for outcome assessor, so low risk of bias regardless of quality of blinding.
Incomplete outcome data (attrition bias) All outcomes	Low risk	Quote: not applicable Judgement comment: no missing data from those analysed reported.
Selective reporting (reporting bias)	High risk	Quote: not applicable Judgement comment: not all pre‐specified outcome measures reported from trial registration: return to the operating room within 30 days, re‐admission within 30 days, superficial infection, deep infection, periprosthetic fracture, cerebrovascular accident or transient ischaemic attack, dislocation.
Other bias	High risk	Quote: not applicable Judgement comment: per protocol analysis undertaken. No funding source clear from reported text.

**Table:** Kayupov 2017b

***Study characteristics***		
Methods	**Study design:** RCT **Intention‐to‐treat analysis:** per protocol **Duration of study:** 10 **Power calculation reached:** yes **Transfusion strategy:** transfusions were administered if the haemoglobin level was < 7.0 g/dL or if it was deemed necessary by an internal medicine physician secondary to patient‐specific medical comorbidities or symptoms. **Was the trial stopped early:** no	
Participants	**Baseline characteristics** TXA, IV *Age (years) (mean SD)*: 55 (12) *Ethnicity*: not reported *Gender (males, females)*: 22/43 M (51%); 21/43 F (49%) *Length of surgery (minutes) (mean SD)*: 67 (12) *Proportion of participants on anticoagulants prior to surgery (n/N, %)*: not reported *Incidence of preoperative anaemia (n/N, %)*: not reported *Co‐morbidities (n/N, %)*: not reported *ASA 1 (n/N, %)*: 4/43 (9.3%) *ASA 2 (n/N, %)*: 32/43 (74.2%) *ASA 3 (n/N, %)*: 7/43 (16.3) *ASA 4 (n/N, %)*: 0/43 (0%) *Number of participants randomised*: 43 *Number of participants receiving treatment*: 43 *Number of participants analysed*: 43 *Dropout rate*: 0/43 (0%) TXA, oral *Age (years) (mean SD)*: 60 (10) *Ethnicity*: not reported *Gender (males, females)*: 20/40 M (50%); 20/40 F (50%) *Length of surgery (minutes) (mean SD)*: 70 (16) *Proportion of participants on anticoagulants prior to surgery (n/N, %)*: not reported *Incidence of preoperative anaemia (n/N, %)*: not reported *Co‐morbidities (n/N, %)*: not reported *ASA 1 (n/N, %)*: 2/40 (5%) *ASA 2 (n/N, %)*: 31/40 (77.5%) *ASA 3 (n/N, %)*: 7/40 (17.5%) *ASA 4 (n/N, %)*: 0/40 (0%) *Number of participants randomised*: 46 *Number of participants receiving treatment*: 40 *Number of participants analysed*: 40 *Dropout rate*: 6/46 (13%) Overall *Age (years) (mean SD)*: not reported *Ethnicity*: not reported *Gender (males, females)*: not reported *Length of surgery (minutes) (mean SD)*: not reported *Proportion of participants on anticoagulants prior to surgery (n, %)*: not reported *Incidence of preoperative anaemia*: not reported *Co‐morbidities*: not reported *ASA 1 (n/N, %)*: not reported *ASA 2 (n/N, %)*: not reported *ASA 3 (n/N, %)*: not reported *ASA 4 (n/N, %)*: not reported *Number of participants randomised*: not reported *Number of participants receiving treatment*: not reported *Number of participants analysed*: not reported *Dropout rate*: not reported **Inclusion criteria:** 1) patients scheduled to undergo cementless primary unilateral total hip replacement **Exclusion criteria:** 1) history of renal failure, 2) kidney transplant, 3) history of an arterial thromboembolic event such as myocardial infarction or stroke within the past year, 4) placement of an arterial stent within the past year, 5) any history of deep venous thrombosis or pulmonary embolism, 6) declined to participate or to consent to receive blood products **If TKR, is tourniquet used:** not applicable **Indication for surgery:** not reported **Type of anaesthetic:** combined spinal‐epidural anaesthesia **Type of surgery:** primary THR	
Interventions	**Intervention characteristics** TXA, IV Patients in the intravenous tranexamic acid group received the standard dosing for our institution of 1 g of tranexamic acid in 10 mL of normal saline solution given as an intravenous bolus immediately prior to incision along with a total dose of 750 mg of ascorbic acid in the form of 3 x 250 mg tablets as a placebo 2 hours prior to the incision TXA, IV, 1 g, intraop (prior to skin incision) AND placebo, oral, preop (2 hours prior to incision) TXA, oral Patients randomised to the oral tranexamic acid group were administered a total oral dose of 1950 mg of tranexamic acid using 3 tablets of 650 mg approximately 2 hours prior to incision and a placebo dose of 10 mL of normal saline solution immediately prior to incision Placebo, IV, intraop (prior to skin incision) AND TXA, 1.95 mg, oral, preop (2 hours prior to incision)	
Outcomes	*Primary outcomes:* Reduction of haemoglobin concentration *Secondary outcomes:* Postoperative calculated blood loss Postoperative calculated haemoglobin loss Transfusion rate Number of blood units transfused Length of hospital stay Incidence of thromboembolic events	
Notes	**Sponsorship source:** none **Country:** USA **Setting:** not reported **Comments:** mean units and SD calculated from paper **Author's name:** E Kayupov **Institution:** Rush University Medical Center **Email:** craigdv@rushortho.com **Address:** Rush University Medical Center, Chicago, Illinois **Native language of paper:** English **Reference type:** full text (1) trial registration (1) **Trial registration number:** NCT02233101 **Was it translated for this review:** no	
***Risk of bias***		
**Bias**	**Authors' judgement**	**Support for judgement**
Random sequence generation (selection bias)	Low risk	Quote: "Randomization between the two treatment groups was done using a random number algorithm to provide a binary output indicating the patient’s assigned treatment." Judgement comment: use of random number table.
Allocation concealment (selection bias)	Unclear risk	Quote: "Enrolled subjects provided informed consent and were randomly assigned by a research assistant to the oral tranexamic acid group or the intravenous tranexamic acid group via sealed, opaque envelope." Judgement comment: not clear whether these were sequentially numbered.
Blinding of participants and personnel (performance bias) Subjective outcomes	Low risk	Quote: "The patient, surgeon, and clinical staff involved in making decisions with regard to study outcomes such as ordering transfusions were blinded to the route of tranexamic acid administration. This information was linked to a confidential and secure database used for collection and analysis by an independent research coordinator and statistician." Judgement comment: blinding of key personnel ensured.
Blinding of participants and personnel (performance bias) Objective outcomes	Low risk	Quote: not applicable Judgement comment: objective outcome for personnel, so low risk of bias regardless of quality of blinding.
Blinding of outcome assessment (detection bias) Subjective outcomes	Low risk	Quote: "The patient, surgeon, and clinical staff involved in making decisions with regard to study outcomes such as ordering transfusions were blinded to the route of tranexamic acid administration." Judgement comment: blinding of outcome assessors described.
Blinding of outcome assessment (detection bias) Objective outcomes	Low risk	Quote: not applicable Judgement comment: objective outcome for outcome assessor, so low risk of bias regardless of quality of blinding.
Incomplete outcome data (attrition bias) All outcomes	Low risk	Quote: not applicable Judgement comment: all mentioned outcomes were measured and reported.
Selective reporting (reporting bias)	High risk	Quote: not applicable Judgement comment: secondary outcomes mentioned in the protocol not reported in the paper: return to the operating room within 30 days, re‐admission within 30 days, superficial infection, deep infection, periprosthetic fracture, cerebrovascular accident or transient ischaemic attack, dislocation.
Other bias	High risk	Quote: not applicable Judgement comment: per protocol analysis performed instead of intention‐to‐treat analysis.

**Table:** King 2019

***Study characteristics***		
Methods	**Study design:** RCT **Intention‐to‐treat analysis:** per protocol **Duration of study:** 13 months (August 2017 to September 2018) + 6 weeks (follow‐up) = 14.5 months **Power calculation reached:** yes **Transfusion strategy:** blood transfusion was performed on all patients with a haemoglobin below 80 g/L or at higher haemoglobin levels dependent on patient specific indications as outlined by the National Blood Authority of Australia **Was the trial stopped early:** no	
Participants	**Baseline characteristics** TXA, oral *Age (years) (mean SD)*: 65.4 (8.8) *Ethnicity*: not reported *Gender (males, females)*: 14/25 M (56.0%); 11/25 F (44%) *Length of surgery (minutes)*: 138.2 (20.8) *Proportion of participants on anticoagulants prior to surgery (n/N, %)*: use of anticoagulants or antithrombotics within 7 days of surgery *Incidence of preoperative anaemia (n/N, %)*: not reported *Co‐morbidities (n/N, %)*: BMI 30.1 (4.8) *ASA 1 (n/N, %)*: not reported *ASA 2 (n/N, %)*: not reported *ASA 3 (n/N, %)*: not reported *ASA 4 (n/N, %)*: not reported *Number of participants randomised*: 31 *Number of participants receiving treatment*: 28 *Number of participants analysed*: 25 *Dropout rate*: 6/31, 19.4% TXA, combined topical/IV/oral *Age (years) (mean SD)*: 63.8 (9.7) *Ethnicity*: not reported *Gender (males, females)*: 12/28 M (42.9%); 16/28 F (57.1%) *Length of surgery (minutes)*: 140.5 (21.4) *Proportion of participants on anticoagulants prior to surgery (n/N, %)*: use of anticoagulants or antithrombotics within 7 days of surgery *Incidence of preoperative anaemia (n/N, %)*: not reported *Co‐morbidities (n/N, %)*: BMI 30.2 (4.6) *ASA 1 (n/N, %)*: not reported *ASA 2 (n/N, %)*: not reported *ASA 3 (n/N, %)*: not reported *ASA 4 (n/N, %)*: not reported *Number of participants randomised*: 33 *Number of participants receiving treatment*: 28 *Number of participants analysed*: 28 *Dropout rate*: 5/33, 15.2% Overall *Age (years) (mean SD)*: not reported *Ethnicity*: not reported *Gender (males, females)*: 26 M, 27 F *Length of surgery (minutes)*: not reported *Proportion of participants on anticoagulants prior to surgery (n/N, %)*: use of anticoagulants or antithrombotics within 7 days of surgery *Incidence of preoperative anaemia (n/N, %)*: not reported *Co‐morbidities (n/N, %)*: not reported *ASA 1 (n/N, %)*: not reported *ASA 2 (n/N, %)*: not reported *ASA 3 (n/N, %)*: not reported *ASA 4 (n/N, %)*: not reported *Number of participants randomised*: 64 *Number of participants receiving treatment*: 56 *Number of participants analysed*: 53 *Drop out rate*: 11/64, 17.2% **Inclusion criteria:** patients scheduled for TKA by the collaborating orthopaedic surgeon during the trial period. All participants were required to be over the age of 18 and able to provide informed consent prior to surgery. **Exclusion criteria:** bilateral TKA, allergy to TXA, history of bleeding disorders, use of anticoagulants or antithrombotics within 7 days of surgery, inability to take apixaban as post‐surgical venous thromboembolism (VTE) prophylaxis, and the presence of contraindications to the use of TXA **If TKR, is tourniquet used:** no **Indication for surgery:** not reported **Type of anaesthetic:** not reported **Type of surgery:** primary TKA	
Interventions	**Intervention characteristics** TXA, oral The study group received an oral 1 g dose 2 hours prior to the commencement of surgery, a second 1 g oral dose 2 hours post‐surgery and a final 1 g oral dose 6 hours post‐surgery TXA, oral, 1 g, preop + 1 g, 2 h postop + 1 g, 6 h postop TXA, combined topical/IV/oral The control group received a 3 g topical application of TXA perioperatively, a 1 g IV dose TXA dose at 2 hours post‐surgery and a final 1 g oral dose 6 hours post‐surgery. TXA, IA, 3 g, intraop + TXA, IV, 1 g, 2 h, postop + TXA, oral, 1 g, 6 h postop	
Outcomes	*Primary outcomes:* Hb levels Blood loss Incidence of DVT *Secondary outcomes:* Adverse events Blood transfusion	
Notes	**Sponsorship source:** non‐pharmaceutical (funding for this study was provided by John Flynn Private Hospital and the Quality Use of Medicines Network, Griffith University) **Country:** Australia **Setting:** not reported **Comments:** none **Author's name:** L King **Institution:** John Flynn Hospital **Email:** n.bernaitis@griffith.edu.au **Address:** School of Pharmacy & Pharmacology, Gold Coast Campus, Griffith University, QLD 4222, Australia **Native language of paper:** English **Reference type:** full text (1) **Trial registration number:** ACTRN12617000617369 **Was it translated for this review:** no	
***Risk of bias***		
**Bias**	**Authors' judgement**	**Support for judgement**
Random sequence generation (selection bias)	Low risk	Quote: "Participants were assigned to one of two groups determined by randomisation using a blocking method." Judgement comment: adequate method of sequence generation with blocking method.
Allocation concealment (selection bias)	Unclear risk	Quote: not applicable Judgement comment: method of allocation concealment not described.
Blinding of participants and personnel (performance bias) Subjective outcomes	High risk	Quote: not applicable Judgement comment: trial registration says that no blinding/masking was done. Subjective outcome for personnel and high risk of bias due to inadequate personnel blinding.
Blinding of participants and personnel (performance bias) Objective outcomes	Low risk	Quote: not applicable Judgement comment: objective outcome for personnel, so low risk of bias regardless of quality of blinding.
Blinding of outcome assessment (detection bias) Subjective outcomes	High risk	Quote: not applicable Judgement comment: trial registration says that no blinding/masking was done. Subjective outcome for personnel and high risk of bias due to inadequate personnel blinding.
Blinding of outcome assessment (detection bias) Objective outcomes	Low risk	Quote: not applicable Judgement comment: objective outcome for personnel, so low risk of bias regardless of quality of blinding.
Incomplete outcome data (attrition bias) All outcomes	Low risk	Quote: not applicable Judgement comment: no obvious outcome data missing.
Selective reporting (reporting bias)	Low risk	Quote: not applicable Judgement comment: all outcomes planned in the protocol or prospective trial registration are reported.
Other bias	Low risk	Quote: not applicable Judgement comment: no other concerns such as early stopping or imbalanced study arms.

**Table:** Langdown 2000

***Study characteristics***		
Methods	**Study design:** RCT **Intention‐to‐treat analysis:** not reported **Duration of study:** not reported **Power calculation reached:** not reported **Transfusion strategy:** not reported **Was the trial stopped early:** no	
Participants	**Baseline characteristics** Placebo *Age (years) (mean SD)*: 72.8 (8.8) *Ethnicity*: not reported *Gender (males, females)*: not reported *Length of surgery (minutes)*: not reported *Proportion of participants on anticoagulants prior to surgery (n/N, %)*: not reported *Incidence of preoperative anaemia (n/N, %)*: not reported *Co‐morbidities (n/N, %)*: not reported *ASA 1 (n/N, %)*: not reported *ASA 2 (n/N, %)*: not reported *ASA 3 (n/N, %)*: not reported *ASA 4 (n/N, %)*: not reported *Number of participants randomised*: not reported *Number of participants receiving treatment*: not reported *Number of participants analysed*: not reported *Dropout rate*: not reported Aprotinin *Age (years) (mean SD)*: 70.0 (9.7) *Ethnicity*: not reported *Gender (males, females)*: not reported *Length of surgery (minutes)*: not reported *Proportion of participants on anticoagulants prior to surgery (n/N, %)*: not reported *Incidence of preoperative anaemia (n/N, %)*: not reported *Co‐morbidities (n/N, %)*: not reported *ASA 1 (n/N, %)*: not reported *ASA 2 (n/N, %)*: not reported *ASA 3 (n/N, %)*: not reported *ASA 4 (n/N, %)*: not reported *Number of participants randomised*: not reported *Number of participants receiving treatment*: not reported *Number of participants analysed*: not reported *Dropout rate*: not reported Overall *Age (years) (mean SD)*: not reported *Ethnicity*: not reported *Gender (males, females)*: not reported *Length of surgery (minutes)*: not reported *Proportion of participants on anticoagulants prior to surgery? (n/N, %)*: not reported *Incidence of preoperative anaemia*: not reported *Co‐morbidities*: not reported *ASA 1 (n/N, %)*: not reported *ASA 2 (n/N, %)*: not reported *ASA 3 (n/N, %)*: not reported *ASA 4 (n/N, %)*: not reported *Number of participants randomised*: 60 *Number of participants receiving treatment*: not reported *Number of participants analysed*: not reported *Drop out rate*: not reported **Inclusion criteria:** patients with a diagnosis of primary osteoarthritis of the hip necessitating THA **Exclusion criteria:** not reported **If TKR, is tourniquet used:** not applicable **Indication for surgery:** primary osteoarthritis of the hip **Type of anaesthetic:** spinal **Type of surgery:** primary THR	
Interventions	**Intervention characteristics** Placebo 1.5 x 10^6 KIU (Kallikrein inactivation units) of intravenous aprotinin or a similar volume of normal saline as a placebo as a bolus at the time of anaesthesia Placebo saline mL, IV, preop Aprotinin 1.5 x 10^6 KIU (Kallikrein inactivation units) of intravenous aprotinin or a similar volume of normal saline as a placebo as a bolus at the time of anaesthesia Aprotinin, IV, 1.5 x 10^6 KIU, preop	
Outcomes	*Primary outcomes:* Total blood loss Postoperative haemoglobin Transfusion requirement *Secondary outcome:* Not reported	
Notes	**Sponsorship source:** none **Country:** UK **Setting:** not reported **Comments:** none **Author's name:** AJ Langdown **Institution:** John Radcliffe Hospital **Email:** not reported **Address:** 83 Naunton Crescent, Leckhampton, Cheltenham, GL53 7BE UK **Native language of paper:** English **Reference type:** full text (1) **Trial registration number:** not applicable, but when checked, trial registration number not found **Was it translated for this review:** no	
***Risk of bias***		
**Bias**	**Authors' judgement**	**Support for judgement**
Random sequence generation (selection bias)	Low risk	Quote: not applicable Judgement comment: the patients were randomised using a random number technique.
Allocation concealment (selection bias)	Unclear risk	Quote: not applicable Judgement comment: insufficient information to permit judgement.
Blinding of participants and personnel (performance bias) Subjective outcomes	Low risk	Quote: "The patient, anesthetist, and surgeon were unaware of which solution was given." Judgement comment: blinding of participants and key study personnel ensured, and unlikely that the blinding could have been broken.
Blinding of participants and personnel (performance bias) Objective outcomes	Low risk	Quote: not applicable Judgement comment: objective outcome for personnel, so low risk of bias regardless of quality of blinding.
Blinding of outcome assessment (detection bias) Subjective outcomes	Unclear risk	Quote: not applicable Judgement comment: insufficient information to permit judgement
Blinding of outcome assessment (detection bias) Objective outcomes	Low risk	Quote: not applicable Judgement comment: objective outcome for outcome assessor, so low risk of bias regardless of quality of blinding.
Incomplete outcome data (attrition bias) All outcomes	Unclear risk	Quote: not applicable Judgement comment: insufficient information to permit judgement. Missing information not reported.
Selective reporting (reporting bias)	High risk	Quote: "Postoperative hemoglobin and transfusion requirements were similar between the 2 groups." Judgement comment: outcomes of interest are reported incompletely so cannot be entered into a meta‐analysis. Data on transfusions given is missing, despite being mentioned in the abstract.
Other bias	Unclear risk	Quote: not applicable Judgement comment: lack of patient characteristic data (e.g. gender) and methodological data (number randomised to each arm, number receiving treatment and dropout rate).

**Table:** Lei 2017

***Study characteristics***		
Methods	**Study design:** RCT **Intention‐to‐treat analysis:** yes **Duration of study:** 4.5 months (4 months + 2 weeks FU) **Power calculation reached:** yes **Transfusion strategy:** according to the guidelines by the National Ministry of Health, patients were transfused only when the postoperative haemoglobin (Hb) level was less than 70 g/L or 70 to 100 g/L with symptoms related to anaemia developing, such as lightheadedness, palpitation, tachypnoea or decreased exercise tolerance. **Was the trial stopped early:** no	
Participants	**Baseline characteristics** TXA, IV pre‐op + TXA, IV 3 and 6 h postop *Age (years) (mean SD)*: 63.6 (7.6) *Ethnicity*: not reported *Gender (males, females)*: 10/53 M (19%); 43/53 F (81%) *Length of surgery (minutes)*: not reported *Proportion of participants on anticoagulants prior to surgery (n/N, %)*: not reported *Incidence of preoperative anaemia (n/N, %)*: 0/53 (0%) *Co‐morbidities (n/N, %)*: hypertension 32/53 (60.3%), diabetes 6/53 (11.3%) *ASA 1 (n/N, %)*: not reported *ASA 2 (n/N, %)*: not reported *ASA 3 (n/N, %)*: not reported *ASA 4 (n/N, %)*: not reported *Number of participants randomised*: 53 *Number of participants receiving treatment*: 53 *Number of participants analysed*: 53 *Dropout rate*: 0/53 (0%) TXA, IV pre‐op + TXA, IV 3, 6 and 9 h postop *Age (years) (mean SD)*: 66.4 (8.4) *Ethnicity*: not reported *Gender (males, females)*: 7/49 M (14%); 42/49 F (86%) *Length of surgery (minutes)*: not reported *Proportion of participants on anticoagulants prior to surgery (n/N, %)*: not reported *Incidence of preoperative anaemia (n/N, %)*: 0/49 (0%) *Co‐morbidities (n/N, %)*: hypertension 29/49 (59.1%), diabetes 6/49 (12.2%) *ASA 1 (n/N, %)*: not reported *ASA 2 (n/N, %)*: not reported *ASA 3 (n/N, %)*: not reported *ASA 4 (n/N, %)*: not reported *Number of participants randomised*: 49 *Number of participants receiving treatment*: 49 *Number of participants analysed*: 49 *Dropout rate*: 0/49 (0%) TXA, IV pre‐op + TXA, IV 9 and 12 h postop *Age (years) (mean SD)*: 65.6 (8.4) *Ethnicity*: not reported *Gender (males, females)*: 10/57 M (18%); 47/57 F (82%) *Length of surgery (minutes)*: not reported *Proportion of participants on anticoagulants prior to surgery (n/N, %)*: not reported *Incidence of preoperative anaemia (n/N, %),* : 0/57 (0%) *Co‐morbidities (n/N, %)*: hypertension 36/57 (63.2%), diabetes 12/49 (24.4%) *ASA 1 (n/N, %)*: not reported *ASA 2 (n/N, %)*: not reported *ASA 3 (n/N, %)*: not reported *ASA 4 (n/N, %)*: not reported *Number of participants randomised*: 57 *Number of participants receiving treatment*: 57 *Number of participants analysed*: 57 *Dropout rate*: 0/57 (0%) Overall *Age (years) (mean SD)*: not reported *Ethnicity*: not reported *Gender (males, females)*: not reported *Length of surgery (minutes)*: not reported *Proportion of participants on anticoagulants prior to surgery (n/N, %)*: not reported *Incidence of preoperative anaemia (n/N, %)*: not reported *Co‐morbidities (n/N, %)*: not reported *ASA 1 (n/N, %)*: not reported *ASA 2 (n/N, %)*: not reported *ASA 3 (n/N, %)*: not reported *ASA 4 (n/N, %)*: not reported *Number of participants randomised*: 159 *Number of participants receiving treatment*: 159 *Number of participants analysed*: 159 *Dropout rate*: 0/159 (0%) **Inclusion criteria:** 1) scheduled to have primary unilateral TKA for osteoarthritis or rheumatoid arthritis, 2) aged 18 years or older **Exclusion criteria:** 1) revisions, 2) bilateral procedures, 3) flexion deformity ≥ 30°, varus/valgus deformity ≥ 30°, 4) patients with anaemia (< 120 g/L for females, < 130 g/L for males), 5) pre‐operative hepatic or renal dysfunction, 6) serious cardiac or cerebrovascular problems, 7) previous history of deep venous thrombosis or pulmonary embolism, 8) congenital or acquired clotting disorders, 9) contraindications for the use of TXA **If TKR, is tourniquet used:** no **Indication for surgery:** osteoarthritis or rheumatoid arthritis **Type of anaesthetic:** general anaesthesia **Type of surgery:** primary TKR	
Interventions	**Intervention characteristics** TXA, IV pre‐op + TXA, IV 3 and 6 h postop Group A: the patients received one bolus of 20 mg/kg IV TXA prior to skin incision, 2 boluses of 10 mg/kg IV TXA 3 and 6 hours later, and 10 mg/kg of normal saline was intravenously injected 9 and 12 hours later TXA, IV, 20 mg/kg, intraop (prior to skin incision) AND TXA, IV, 10 mg/kg, intra/postop 3 + 6 hours after 1st dose + placebo, IV, postop 9 + 12 hours after 1st dose TXA, IV pre‐op + TXA, IV 3, 6 and 9 postop Group B: a single bolus of 20 mg/kg IV TXA was administered before skin incision, 10 mg/kg of TXA was administered 3, 6 and 9 hours later, and another 10 mg/kg of normal saline was administered 12 hours later TXA, IV, 20 mg/kg, intraop (prior to skin incision) AND TXA, IV, 10 mg/kg, intra/postop 3 + 6 + 9 hours after 1st dose + placebo, IV, postop 12 hours after 1st dose TXA, IV pre‐op + TXA, IV 9 and 12 h postop Group C: a dosage of 20 mg/kg IV TXA was administered before skin incision, and 10 mg/kg of TXA was administered 3, 6, 9 and 12 hours later TXA, IV, 20 mg/kg, intraop (prior to skin incision) AND TXA, IV, 10 mg/kg, intra/postop 3 + 6 + 9 + 12 hours after 1st dose	
Outcomes	*Primary outcome:* Hidden blood loss *Secondary outcomes:* Hb decline Fibrinolysis parameters (fibrinogen degradation products (FDP), D‐dimer) Inflammatory factors (interleukin‐6 (IL‐6)) Knee range of motion Length of hospital stay Complications	
Notes	**Sponsorship source:** non‐pharmaceutical **Country:** China **Setting:** single‐centre **Comments:** none **Author's name:** Y Lei **Institution:** Sichuan University **Email:** peifux@126.com **Address:** Department of Orthopaedics, West China Hospital, Sichuan University, 37# Wainan Guoxue Road, Chengdu 610041, People’s Republic of China **Native language of paper:** English **Reference type:** full text (1) trial registration (1) **Trial registration number:** ChiCTR‐INR‐16009288 **Was it translated for this review?:** no	
***Risk of bias***		
**Bias**	**Authors' judgement**	**Support for judgement**
Random sequence generation (selection bias)	Unclear risk	Quote: "A random allocation sequence" Judgement comment: insufficient information to permit judgement.
Allocation concealment (selection bias)	Unclear risk	Quote: "A random allocation sequence concealed in opaque sealed envelopes only opened before surgery." Judgement comment: unclear whether consecutively numbered.
Blinding of participants and personnel (performance bias) Subjective outcomes	High risk	Quote: "The analgesists and nurses were not involved in this trial. The patients, surgeons, data controller, and analyst were blinded." Judgement comment: anaesthetists were not blinded to the treatment regimen.
Blinding of participants and personnel (performance bias) Objective outcomes	Low risk	Quote: not applicable Judgement comment: objective outcome for personnel, so low risk of bias regardless of quality of blinding.
Blinding of outcome assessment (detection bias) Subjective outcomes	Low risk	Quote: "The analgesists and nurses were not involved in this trial. The patients, surgeons, data controller, and analyst were blinded." Judgement comment: blinding of outcome assessor described.
Blinding of outcome assessment (detection bias) Objective outcomes	Low risk	Quote: not applicable Judgement comment: objective outcome for outcome assessor, so low risk of bias regardless of quality of blinding.
Incomplete outcome data (attrition bias) All outcomes	Low risk	Quote: not applicable Judgement comment: no missing data.
Selective reporting (reporting bias)	High risk	Quote: not applicable Judgement comment: not all outcome measures are reported as per the trial registration specifications, e.g. VAS score and swelling ratio.
Other bias	Low risk	Quote: not applicable Judgement comment: appears to be free of other bias.

**Table:** Lei 2018

***Study characteristics***		
Methods	**Study design:** RCT **Intention‐to‐treat analysis:** yes **Duration of study:** 8 months (5 + 3 months FU) **Power calculation reached:** yes **Transfusion strategy:** patients were transfused according to the guidelines by the National Ministry of Health, and either of the following leads to consideration of transfusion: patient has a haemoglobin (Hb) level of 70 g/L or less or patient has a Hb level between 70 g/L and 100 g/L with symptoms related to anaemia developing, such as dizziness, cardiopalmus, tachypnoea or decreased exercise tolerance. **Was the trial stopped early:** no	
Participants	**Baseline characteristics** TXA, IV, pre‐op + 2 doses TXA, IV postop *Age (years) (mean SD)*: 54.3 (12.6) *Ethnicity*: not reported *Gender (males, females)*: 19/50 M (38%); 31/50 F (62%) *Length of surgery (minutes)*: not reported *Proportion of participants on anticoagulants prior to surgery (n/N, %)*: 0/50 (0%) *Incidence of preoperative anaemia (n/N, %)*: not reported *Co‐morbidities (n/N, %)*: hypertension 15/50 (30%) *ASA 1 (n/N, %)*: not reported *ASA 2 (n/N, %)*: not reported *ASA 3 (n/N, %)*: not reported *ASA 4 (n/N, %)*: not reported *Number of participants randomised*: 50 *Number of participants receiving treatment*: 50 *Number of participants analysed*: 50 *Dropout rate*: 0/50, (0%) TXA, IV, pre‐op + 3 doses TXA, IV postop *Age (years) (mean SD)*: 50.0 (12.2) *Ethnicity*: not reported *Gender (males, females)*: 22/50 M (44%); 28/50 F (56%) *Length of surgery (minutes)*: not reported *Proportion of participants on anticoagulants prior to surgery (n/N, %)*: 0/50 (0%) *Incidence of preoperative anaemia (n/N, %)*: not reported *Co‐morbidities (n/N, %)*: hypertension 9/50 (18%) *ASA 1 (n/N, %)*: not reported *ASA 2 (n/N, %)*: not reported *ASA 3 (n/N, %)*: not reported *ASA 4 (n/N, %)*: not reported *Number of participants randomised*: 50 *Number of participants receiving treatment*: 50 *Number of participants analysed*: 50 *Dropout rate*: 0/50 (0%) TXA, IV, pre‐op + 4 doses TXA, IV postop *Age (years) (mean SD)*: 53.2 (13.0) *Ethnicity*: not reported *Gender (males, females)*: 20/50 M (40%); 30/50 F (60%) *Length of surgery (minutes)*: not reported *Proportion of participants on anticoagulants prior to surgery (n/N, %)*: 0/50 (0%) *Incidence of preoperative anaemia (n/N, %)*: not reported *Co‐morbidities (n/N, %)*: hypertension 9/50 (24%) *ASA 1 (n/N, %)*: not reported *ASA 2 (n/N, %)*: not reported *ASA 3 (n/N, %)*: not reported *ASA 4 (n/N, %)*: not reported *Number of participants randomised*: 50 *Number of participants receiving treatment*: 50 *Number of participants analysed*: 50 *Dropout rate*: 0/50 (0%) Overall *Age (years) (mean SD)*: not reported *Ethnicity*: not reported *Gender (males, females)*: not reported *Length of surgery (minutes)*: not reported *Proportion of participants on anticoagulants prior to surgery (n/N, %)*: not reported *Incidence of preoperative anaemia (n/N, %)*: not reported *Co‐morbidities (n/N, %)*: not reported *ASA 1 (n/N, %)*: not reported *ASA 2 (n/N, %)*: not reported *ASA 3 (n/N, %)*: not reported *ASA 4 (n/N, %)*: not reported *Number of participants randomised*: 150 *Number of participants receiving treatment*: not reported *Number of participants analysed*: 150 *Dropout rate*: 0/150 (0%) **Inclusion criteria:** 1) patients undergoing primary unilateral THA **Exclusion criteria:** 1) known allergies to TXA, 2) administration of any anti‐inflammatory or antiplatelet drugs 7 days before operation, 3) previous history of deep venous thrombosis or pulmonary embolism, clotting disorders, cardiac or cerebrovascular problem (history of myocardial infarction, angina, atrial fibrillation or previous stroke), 4) kidney or liver failure **If TKR, is tourniquet used:** not applicable **Indication for surgery:** osteoarthritis secondary to developmental dysplasia of the hip, osteonecrosis of the femoral head, osteoarthritis **Type of anaesthetic:** general anaesthesia **Type of surgery:** primary THR	
Interventions	**Intervention characteristics** TXA, IV, pre‐op + 2 doses TXA, IV postop To each patient of all groups, one dose of 20 mg/kg intravenous (IV) TXA was given prior to skin incision, with 2 doses of 1 g (100 mL) IV TXA given 3 and 6 hours later. Group A served as the control group and received another 2 doses of 100 mL normal saline 9 and 12 hours after the first dose; Group B received one 1 g dose of IV TXA 9 hours later and one 100 mL dose of normal saline 12 hours later; and group C received another 2 doses of 1 g IV TXA 9 and 12 hours after the first dose. The patients, attending surgeons, data controller and analyst were blinded to interventions. TXA, IV, 20 mg/kg preop AND TXA, IV, 1 g, postop 3 + 6 hours after 1st dose AND placebo, IV, postop 9 + 12 hours TXA, IV, pre‐op + 3 doses TXA, IV postop Group B received one 1 g dose of IV TXA 9 hours later and one 100 mL dose of normal saline 12 hours later TXA, IV, 20 mg/kg preop AND TXA, IV, 1 g, post op 3 + 6 + 9 hours after 1st dose AND placebo, IV, postop 12 hours TXA, IV, pre‐op + 4 doses TXA, IV postop Group C received another 2 doses of 1 g IV TXA 9 and 12 hours after the first dose TXA, IV, 20 mg/kg preop AND TXA, IV, 1 g, postop 3 + 6 + 9 + 12 hours after 1st dose	
Outcomes	*Primary outcome:* Hidden blood loss *Secondary outcomes:* Total blood loss Maximum haemoglobin (Hb) drop Transfusion rate Length of hospital stay Complications	
Notes	**Sponsorship source:** not reported **Country:** China **Setting:** single‐centre **Comments:** none **Author's name:** Y Lei **Institution:** Sichuan University **Email:** not reported **Address:** Department of Orthopaedics, West China Hospital, Sichuan University, 37# WainanGuoxue Road, Chengdu 610041, People's Republic of China **Native language of paper:** English **Reference type:** full text (1) trial registration (1) **Trial registration number:** ChiCTR‐INR‐16009288 **Was it translated for this review:** no	
***Risk of bias***		
**Bias**	**Authors' judgement**	**Support for judgement**
Random sequence generation (selection bias)	Low risk	Quote: "In total, 150 patients were randomized, with a computer‐ generated list" Judgement comment: computer‐generated randomisation.
Allocation concealment (selection bias)	Unclear risk	Quote: "The clinical research assistant use the computer to generate the random number. sealed envelopes" Judgement comment: insufficient information to permit judgement.
Blinding of participants and personnel (performance bias) Subjective outcomes	Low risk	Quote: "The patients, attending surgeons, data controller, and analyst were blinded to interventions." Judgement comment: blinding of key personnel described.
Blinding of participants and personnel (performance bias) Objective outcomes	Low risk	Quote: not applicable Judgement comment: objective outcome for personnel, so low risk of bias regardless of quality of blinding.
Blinding of outcome assessment (detection bias) Subjective outcomes	Low risk	Quote: "The patients, attending surgeons, data controller, and analyst were blinded to interventions." Judgement comment: blinding of outcome assessor described.
Blinding of outcome assessment (detection bias) Objective outcomes	Low risk	Quote: not applicable Judgement comment: objective outcome for outcome assessor, so low risk of bias regardless of quality of blinding.
Incomplete outcome data (attrition bias) All outcomes	Low risk	Quote: not applicable Judgement comment: no missing data.
Selective reporting (reporting bias)	High risk	Quote: not applicable Judgement comment: not all outcomes of interest reported on trial registry reported in paper: range of motion, VAS score, swelling ratio.
Other bias	High risk	Quote: not applicable Judgement comment: the same trial registration was also quoted for a different paper: "Multiple boluses of intravenous tranexamic acid to reduce hidden blood loss and the inflammatory response following enhancedrecovery primary total hip arthroplasty" published in the Bone & Joint Journal. For this paper, the interventions reported in the trial registration do not match the interventions given to the patients.

**Table:** Lei 2020

***Study characteristics***		
Methods	**Study design:** RCT **Intention‐to‐treat analysis:** yes **Duration of study:** 8 months (May 2017 to January 2018) + 3 months (follow‐up) = 11 months **Power calculation reached:** yes **Transfusion strategy:** decisions to transfuse were made based on the guidelines by the National Ministry of Health: 1) patient has a haemoglobin (Hb) level of 70 g/L or less; 2) patients whose Hb level was between 70 g/L and 100 g/L but who had symptoms related to anaemia develop, such as dizziness, increased heart rates, tachypnoea or decreased exercise tolerance **Was the trial stopped early:** no	
Participants	**Baseline characteristics** TXA, IV 20 mg/kg + 3 x TXA, IV 1 g *Age (years) (mean SD)*: 66.2 (8.3) *Ethnicity*: not reported *Gender (males, females)*: 7/50 M (14%), 43/50 F (86%) *Length of surgery (minutes)*: 72.4 (11.1) *Proportion of participants on anticoagulants prior to surgery (n/N, %)*: not reported *Incidence of preoperative anaemia (n/N, %)*: not reported *Co‐morbidities (n/N, %)*: hypertension: 30/50 *ASA 1 (n/N, %)*: not reported *ASA 2 (n/N, %)*: not reported *ASA 3 (n/N, %)*: not reported *ASA 4 (n/N, %)*: not reported *Number of participants randomised*: 50 *Number of participants receiving treatment*: not reported *Number of participants analysed*: 50 *Drop out rate*: 0/50, 0% TXA, IV 20 mg/kg + 4 x TXA, IV 1 g *Age (years) (mean SD)*: 66.9 (7.9) *Ethnicity*: not reported *Gender (males, females)*: 9/50 M (18%), 41/50 F (82%) *Length of surgery (minutes)*: 71.8 (10.4) *Proportion of participants on anticoagulants prior to surgery (n/N, %)*: not reported *Incidence of preoperative anaemia (n/N, %)*: not reported *Co‐morbidities (n/N, %)*: hypertension: 29/50 *ASA 1 (n/N, %)*: not reported *ASA 2 (n/N, %)*: not reported *ASA 3 (n/N, %)*: not reported *ASA 4 (n/N, %)*: not reported *Number of participants randomised*: 50 *Number of participants receiving treatment*: not reported *Number of participants analysed*: 50 *Dropout rate*: 0/50, 0% TXA, IV 20 mg/kg + 5 x TXA, IV 1 g *Age (years) (mean SD)*: 66.2 (7.7) *Ethnicity*: not reported *Gender (males, females)*: 10/50 M (20%), 40/50 F (80%) *Length of surgery (minutes)*: 72.2 (11.1) *Proportion of participants on anticoagulants prior to surgery (n/N, %)*: not reported *Incidence of preoperative anaemia (n/N, %)*: not reported *Co‐morbidities (n/N, %)*: hypertension: 31/50 *ASA 1 (n/N, %)*: not reported *ASA 2 (n/N, %)*: not reported *ASA 3 (n/N, %)*: not reported *ASA 4 (n/N, %)*: not reported *Number of participants randomised*: 50 *Number of participants receiving treatment*: not reported *Number of participants analysed*: 50 *Dropout rate*: 0/50, 0% Overall *Age (years) (mean SD)*: not reported *Ethnicity*: not reported *Gender (males, females)*: not reported *Length of surgery (minutes)*: not reported *Proportion of participants on anticoagulants prior to surgery (n/N, %)*: not reported *Incidence of preoperative anaemia (n/N, %)*: not reported *Co‐morbidities (n/N, %)*: not reported *ASA 1 (n/N, %)*: not reported *ASA 2 (n/N, %)*: not reported *ASA 3 (n/N, %)*: not reported *ASA 4 (n/N, %)*: not reported *Number of participants randomised*: 202 *Number of participants receiving treatment*: not reported *Number of participants analysed*: 200 *Drop out rate*: 0/200, 0% **Inclusion criteria:** patients undergoing TKA **Exclusion criteria:** cardiovascular problems or cerebrovascular conditions (history of myocardial infarction, angina, atrial fibrillation or previous stroke), kidney or liver failure, previous history of deep venous thrombosis or pulmonary embolism, allergies to drugs used **If TKR, is tourniquet used:** tourniquet not used **Indication for surgery:** osteoarthrosis or rheumatoid arthritis **Type of anaesthetic:** general **Type of surgery:** unilateral TKA	
Interventions	**Intervention characteristics** TXA, IV 20 mg/kg + 3 x TXA, IV 1 g Group B: the patients received one dose of 20 mg/kg IV TXA 5 to 15 min before the skin incision, and 3 doses of 1 g (100 mL) IV TXA 3, 6 and 12 h later TXA, IV, 20 mg/kg, preop + TXA, IV, 1 g, 3, 6 and 12 h postop TXA, IV 20 mg/kg + 4 x TXA, IV 1 g Group C: the patients received one dose of 20 mg/kg IV TXA 5 to 15 min before the skin incision, and 4 doses of 1 g (100 mL) IV TXA 3, 6, 12 and 18 h later TXA, IV, 20 mg/kg, preop + TXA, IV, 1g, 3, 6, 12 and 18 h postop TXA, IV 20 mg/kg + 5 x TXA, IV 1 g Group D: the patients received one dose of 20 mg/kg IV TXA 5 to 15 min before the skin incision, and 5 doses of 1 g (100 mL) IV TXA 3, 6, 12, 18 and 24 h later TXA, IV, 20 mg/kg, preop + TXA, IV, 1 g, 3, 6, 12, 18 and 24 h postop	
Outcomes	*Primary outcome*: Hidden blood loss *Secondary outcomes:* Allogeneic transfusion Level of inflammatory markers (C‐reactive protein (CRP) and interleukin‐6 (IL‐6)) and fibrinolysis parameter Length of hospital stay Transfusion rate Complications	
Notes	**Sponsorship source:** non‐pharmaceutical (this study was funded by the National Health and Family Planning Commission of the People’s Republic of China (CN) program (201302007)) **Country:** China **Setting:** single‐centre **Comments:** 1) it is unclear as to what Group A (control) were treated with, therefore this group was not used **Author's name:** Y Lei **Institution:** The First Affiliated Hospital of Chongqing Medical University **Email:** Wei Huang: huangw511@163.com **Address:** Department of Orthopedics, The First Affiliated Hospital of Chongqing Medical University, Chongqing 400016, People’s Republic of China **Native language of paper:** English **Reference type:** full text (1), trial registration (1) **Trial registration number:** ChiCTR‐INR‐16009288 **Was it translated for this review:** no	
***Risk of bias***		
**Bias**	**Authors' judgement**	**Support for judgement**
Random sequence generation (selection bias)	Unclear risk	Quote: not applicable Judgement comment: method of sequence generation for randomisation not described.
Allocation concealment (selection bias)	Unclear risk	Quote: not applicable Judgement comment: method of allocation concealment not described.
Blinding of participants and personnel (performance bias) Subjective outcomes	Low risk	Quote: "The patients, attending surgeons, data controller, and analyst were blinded to interventions." Judgement comment: subjective outcome for personnel and low risk of bias due to adequate personnel blinding.
Blinding of participants and personnel (performance bias) Objective outcomes	Low risk	Quote: not applicable Judgement comment: objective outcome for personnel, so low risk of bias regardless of quality of blinding.
Blinding of outcome assessment (detection bias) Subjective outcomes	Low risk	Quote: not applicable Judgement comment: subjective outcome for personnel and low risk of bias due to adequate personnel blinding.
Blinding of outcome assessment (detection bias) Objective outcomes	Low risk	Quote: not applicable Judgement comment: objective outcome for personnel, so low risk of bias regardless of quality of blinding.
Incomplete outcome data (attrition bias) All outcomes	Unclear risk	Quote: not applicable Judgement comment: insufficient reporting of attrition/exclusions to permit judgement of ‘low risk’ or ‘high risk’.
Selective reporting (reporting bias)	Low risk	Quote: not applicable Judgement comment: all outcomes planned in the protocol or prospective trial registration are reported.
Other bias	Unclear risk	Quote: not applicable Judgement comment: it is unclear what the 'control' arm received.

**Table:** Lemay 2004

***Study characteristics***		
Methods	**Study design:** RCT **Intention‐to‐treat analysis:** yes **Duration of study:** not reported **Power calculation reached:** no **Transfusion strategy:** before the surgery, a Hb transfusion trigger point was determined for each patient according to the following criteria: for men over 60 yr, women over 65 yr, and patients with a history of atherosclerotic disease, left ventricular dysfunction (ejection fraction < 35%), severe pulmonary obstructive disease (forced expiratory volume in one second < 1.5 L/min–1), or ingestion of calcium channel blockers, the transfusion trigger was 90 g/L–1. For all other patients, the transfusion trigger was 70 g/L–1, but they could be reclassified to the higher trigger by the attending physician (anaesthesiologist or physician in charge of the postoperative period) if they had signs of haemodynamic instability (heart rate > 120 beats/min–1 or a systolic blood pressure decrease by > 20% of preoperative value) despite adequate volume replacement. **Was the trial stopped early:** no	
Participants	**Baseline characteristics** Placebo *Age (years) (mean SD)*: 53.6 (12.8) *Ethnicity*: not reported *Gender (males, females)*: 13/19 M (68%); 6/19 F (32%) *Length of surgery (minutes) (mean SD)*: 101.4 (22.4) *Proportion of participants on anticoagulants prior to surgery (n/N, %)*: not reported *Incidence of preoperative anaemia (n/N, %)*: 0/19 (0%) *Co‐morbidities (n/N, %)*: not reported *ASA 1 (n/N, %)*: 9/19 (47.4%) *ASA 2 (n/N, %)*: 10/19 (52.6%) *ASA 3 (n/N, %)*: 0/19 (0%) *ASA 4 (n/N, %)*: 0/19 (0%) *Number of participants randomised*: 20 *Number of participants receiving treatment*: not reported *Number of participants analysed*: 19 *Dropout rate*: 1/20 (5%) TXA *Age (years) (mean SD)*: 59.7 (10.3) *Ethnicity*: not reported *Gender (males, females)*: 12/20 M (60%); 8/20 F (40%) *Length of surgery (minutes) (mean SD)*: 106.6 (26.4) *Proportion of participants on anticoagulants prior to surgery (n/N, %)*: not reported *Incidence of preoperative anaemia (n/N, %)*: 0/20 (0%) *Co‐morbidities (n/N, %)*: not reported *ASA 1 (n/N, %)*: 6/20 (30%) *ASA 2 (n/N, %)*: 12/20 (60%) *ASA 3 (n/N, %)*: 2/20 (10%) *ASA 4 (n/N, %)*: 0/20 (0%) *Number of participants randomised*: 20 *Number of participants receiving treatment*: 20 *Number of participants analysed*: 20 *Dropout rate*: 0/20 (0%) Overall *Age (years) (mean SD)*: not reported *Ethnicity*: not reported *Gender (males, females)*: not reported *Length of surgery (minutes) (mean SD)*: not reported *Proportion of participants on anticoagulants prior to surgery (n/N, %)*: not reported *Incidence of preoperative anaemia (n/N, %)*: not reported *Co‐morbidities (n/N, %)*: not reported *ASA 1 (n/N, %)*: not reported *ASA 2 (n/N, %)*: not reported *ASA 3 (n/N, %)*: not reported *ASA 4 (n/N, %)*: not reported *Number of participants randomised*: not reported *Number of participants receiving treatment*: not reported *Number of participants analysed*: not reported *Dropout rate*: not reported **Inclusion criteria:** 1) patients were eligible for this study if they were ASA class I to III, 2) undergoing primary THR **Exclusion criteria:** 1) history of previous ipsilateral hip surgery, 2) known or suspected allergy to medications used (TXA, local anaesthetics, midazolam, fentanyl, propofol, or dalteparin), 3) anaemia (haemoglobin (Hb) < 115 g/L–1 for women, Hb < 130 g/L–1 for men), 4) inherited or acquired haemostatic diseases, 5) abnormal coagulation screening tests (platelet count, prothrombin time, activated partial thromboplastin time), 6) ingestion of aspirin or other nonsteroidal anti‐inflammatory drugs within 7 days of surgery, 7) renal (serum creatinine > 2 standard deviation for age) or hepatic insufficiency, 8) pregnancy, 9) history of deep venous thrombosis (DVT) or pulmonary embolism, 10) history of ocular pathology or ophthalmological procedure other than corrective lenses **If TKR, is tourniquet used:** not applicable **Indication for surgery:** not reported **Type of anaesthetic:** spinal anaesthetic **Type of surgery:** primary THR	
Interventions	**Intervention characteristics** Placebo Patients in Group P received an equivalent volume of physiologic saline. Placebo, IV, preop (immediately before surgery) AND placebo, IV, intraop infusion until skin closure TXA In Group TXA, TXA was given immediately before the surgery. After a test dose of 1 mL, patients received a dose of 10 mg/kg–1 IV followed by an infusion of 1 mg/kg–1/hr–1 until skin closure. Patients in Group P received an equivalent volume of physiologic saline. TXA, IV, 10 mg/kg, preop (immediately before surgery) AND TXA, IV, 10 mg/kg, intraop infusion until skin closure	
Outcomes	*Primary outcomes:* Intraoperative and total blood losses Transfusion requirements *Secondary outcome*: Not reported	
Notes	**Sponsorship source:** non‐pharmaceutical **Country:** Canada **Setting:** not reported **Comments:** number of units transfused reported includes autologous pre‐donated blood transfusion units. As such, no results extracted. **Author's name:** J Guay **Institution:** Hôpital Maisonneuve‐Rosemont **Email:** joanne.guay@umontreal.ca **Address:** Département d’anesthésie‐réanimation, Hôpital Maisonneuve‐Rosemont, 5415, boul. l’Assomption, Montréal, Québec H1T 2M4, Canada **Native language of paper:** English **Reference type:** full text (1) **Trial registration number:** not applicable, but when checked, trial registration number not found **Was it translated for this review:** no	
***Risk of bias***		
**Bias**	**Authors' judgement**	**Support for judgement**
Random sequence generation (selection bias)	Low risk	Quote: "Patients were randomly allocated, with randomization in blocks of four, to receive either TA (Group TA) or a placebo (Group P)." Judgement comment: block randomisation.
Allocation concealment (selection bias)	Low risk	Quote: "Both solutions were prepared by the pharmacist." Judgement comment: central allocation by pharmacist.
Blinding of participants and personnel (performance bias) Subjective outcomes	Low risk	Quote: "Patient caregivers (nurses, residents, staff physicians), and the investigator collecting the data were blinded to the solution used." Judgement comment: likely blinding maintained.
Blinding of participants and personnel (performance bias) Objective outcomes	Low risk	Quote: not applicable Judgement comment: objective outcome for personnel, so low risk of bias regardless of quality of blinding.
Blinding of outcome assessment (detection bias) Subjective outcomes	Low risk	Quote: "Patient caregivers (nurses, residents, staff physicians), and the investigator collecting the data were blinded to the solution used." Judgement comment: likely blinding of outcome assessors maintained.
Blinding of outcome assessment (detection bias) Objective outcomes	Low risk	Quote: not applicable Judgement comment: objective outcome for outcome assessor, so low risk of bias regardless of quality of blinding.
Incomplete outcome data (attrition bias) All outcomes	Low risk	Quote: not applicable Judgement comment: all missing data for outcomes were explained.
Selective reporting (reporting bias)	High risk	Quote: not applicable Judgement comment: one or more outcomes of interest are reported incompletely so that they cannot be entered into a meta‐analysis. No protocol available to confirm pre‐specified outcomes reported.
Other bias	Unclear risk	Quote: not applicable Judgement comment: number receiving treatment unclear.

**Table:** Levine 2014

***Study characteristics***		
Methods	**Study design:** RCT **Intention‐to‐treat analysis:** yes **Duration of study:** not reported (routine clinical follow‐up occurred at 2, 6 and 12 weeks after surgery with radiographic analysis being performed at 6 weeks) **Power calculation reached:** no **Transfusion strategy:** not reported **Was the trial stopped early:** no	
Participants	**Baseline characteristics** TXA, IV, uniform dose *Age (years) (mean SD)*: 62.26 (7.31) *Ethnicity*: not reported *Gender (males, females)*: 7/20 M (35%); 13/20 F (65%) *Length of surgery (minutes)*: not reported *Proportion of participants on anticoagulants prior to surgery (n/N, %)*: not reported *Incidence of preoperative anaemia (n/N, %)*: not reported *Co‐morbidities (n/N, %)*: not reported *ASA 1 (n/N, %)*: not reported *ASA 2 (n/N, %)*: not reported *ASA 3 (n/N, %)*: not reported *ASA 4 (n/N, %)*: not reported *Number of participants randomised*: 20 *Number of participants receiving treatment*: 20 *Number of participants analysed*: 20 *Dropout rate*: 0, 0% TXA, IV, weighted dose *Age (years) (mean SD)*: 66.50 (9.65) *Ethnicity*: not reported *Gender (males, females)*: 8/20 M (40%); 12/20 F (60%) *Length of surgery (minutes)*: not reported *Proportion of participants on anticoagulants prior to surgery (n/N, %)*: not reported *Incidence of preoperative anaemia (n/N, %)*: not reported *Co‐morbidities (n/N, %)*: not reported *ASA 1 (n/N, %)*: not reported *ASA 2 (n/N, %)*: not reported *ASA 3 (n/N, %)*: not reported *ASA 4 (n/N, %)*: not reported *Number of participants randomised*: 20 *Number of participants receiving treatment*: 20 *Number of participants analysed*: 20 *Dropout rate*: 0, 0% Overall *Age (years) (mean SD)*: not reported *Ethnicity*: not reported *Gender (males, females)*: not reported *Length of surgery (minutes)*: not reported *Proportion of participants on anticoagulants prior to surgery (n/N, %)*: not reported *Incidence of preoperative anaemia*: not reported *Co‐morbidities*: not reported *ASA 1 (n/N, %)*: not reported *ASA 2 (n/N, %)*: mean ASA only reported *ASA 3 (n/N, %)*: not reported *ASA 4 (n/N, %)*: not reported *Number of participants randomised*: not reported *Number of participants receiving treatment*: not reported *Number of participants analysed*: not reported *Drop out rate*: not reported **Inclusion criteria:** 1) consecutive patients that were candidates for TKA **Exclusion criteria:** 1) age less than 18, age greater than 85, 2) prior history of an open surgical procedure on the operative knee, 3) significant cardiac disease, 4) history of a previous venothromboembolic (VTE) event **If TKR, is tourniquet used:** yes **Indication for surgery:** osteoarthritis **Type of anaesthetic:** not reported **Type of surgery:** primary TKR	
Interventions	**Intervention characteristics** TXA, IV, uniform dose We therefore elected to compare two of the more common regimens in the literature, a uniform 1 g dose versus a weighted, 20 mg/kg dose. For both groups, the TXA was administered as an infusion just prior to tourniquet release TXA, IV, 1 g, intraop (10 mins prior to tourniquet deflation) TXA, IV, weighted dose We therefore elected to compare two of the more common regimens in the literature, a uniform 1 g dose versus a weighted, 20 mg/kg dose. For both groups, the TXA was administered as an infusion just prior to tourniquet release TXA, IV, 20 mg/kg, intraop (10 mins prior to tourniquet deflation)	
Outcomes	*Primary outcomes:* Intraoperative blood loss Postoperative drain output Tourniquet time Surgery time Complications Transfusion requirements Preoperative and postoperative haemoglobin and haematocrit values	
Notes	**Sponsorship source:** non‐pharmaceutical **Country:** USA **Setting:** single‐centre **Comments:** decision made to not extract from control arm as historical cohort with no placebo **Author's name:** BR Levine **Institution:** Rush University Medical Center, Chicago, Illinois **Email:** not reported **Address:** Rush University Medical Center, 1611 W. Harrison St, Ste 300 Chicago, Illinois 60612. **Native language of paper:** English **Reference type:** full text (1) trial registration (1) **Trial registration number:** NCT01651806 **Was it translated for this review:** no	
***Risk of bias***		
**Bias**	**Authors' judgement**	**Support for judgement**
Random sequence generation (selection bias)	Unclear risk	Quote: "Enrolled subjects were randomly assigned to the uniform dose group or weighted dose group at the time of the surgery via the opening of a randomly selected, sealed envelope." Judgement comment: insufficient information to permit judgement.
Allocation concealment (selection bias)	Unclear risk	Quote: "Enrolled subjects were randomly assigned to the uniform dose group or weighted dose group at the time of the surgery via the opening of a randomly selected, sealed envelope." Judgement comment: does not mention if envelopes were opaque or sequentially numbered.
Blinding of participants and personnel (performance bias) Subjective outcomes	Low risk	Quote: "The patient and the independent reviewer were blinded as to the dose of TA utilized during the surgery." Judgement comment: blinding of participants likely maintained.
Blinding of participants and personnel (performance bias) Objective outcomes	Low risk	Quote: not applicable Judgement comment: objective outcome for personnel, so low risk of bias regardless of quality of blinding.
Blinding of outcome assessment (detection bias) Subjective outcomes	Low risk	Quote: "An independent observer who was blinded to the intra‐ operative dosing regimen that the patient received reviewed all charts." Judgement comment: likely blinding of outcome assessor maintained.
Blinding of outcome assessment (detection bias) Objective outcomes	Low risk	Quote: not applicable Judgement comment: objective outcome for assessor, therefore low risk of bias regardless of blinding.
Incomplete outcome data (attrition bias) All outcomes	Low risk	Quote: not applicable Judgement comment: no missing data.
Selective reporting (reporting bias)	Unclear risk	Quote: not applicable Judgement comment: all pre‐specified outcomes reported, but timings of outcomes unclear. Discrepancies between time points in trial registration and main paper.
Other bias	Low risk	Quote: not applicable Judgement comment: no other sources of bias.

**Table:** Llau 1998

***Study characteristics***		
Methods	**Study design:** RCT **Intention‐to‐treat analysis:** not reported **Duration of study:** not reported **Power calculation reached:** not reported **Transfusion strategy:** the patients were transfused 1 packed red cells (PRC) when Hb < 9 g%. Hb was determined 2 hours after each PRC transfusion. **Was the trial stopped early:** no	
Participants	**Baseline characteristics** Placebo *Age (years) (mean SD)*: 67 (7) *Ethnicity*: not reported *Gender (males, females)*: not reported *Length of surgery (minutes)*: not reported *Proportion of participants on anticoagulants prior to surgery (n/N, %)*: not reported *Incidence of preoperative anaemia (n/N, %)*: not reported *Co‐morbidities (n/N, %)*: not reported *ASA 1 (n/N, %)*: not reported *ASA 2 (n/N, %)*: not reported *ASA 3 (n/N, %)*: not reported *ASA 4 (n/N, %)*: not reported *Number of participants randomised*: 10 *Number of participants receiving treatment*: not reported *Number of participants analysed*: not reported *Dropout rate*: not reported Aprotinin *Age (years) (mean SD)*: 68 (8) *Ethnicity*: not reported *Gender (males, females)*: not reported *Length of surgery (minutes)*: not reported *Proportion of participants on anticoagulants prior to surgery (n/N, %)*: not reported *Incidence of preoperative anaemia (n/N, %)*: not reported *Co‐morbidities (n/N, %)*: not reported *ASA 1 (n/N, %)*: not reported *ASA 2 (n/N, %)*: not reported *ASA 3 (n/N, %)*: not reported *ASA 4 (n/N, %)*: not reported *Number of participants randomised*: 10 *Number of participants receiving treatment*: not reported *Number of participants analysed*: not reported *Dropout rate*: not reported Overall *Age (years) (mean SD)*: not reported *Ethnicity*: not reported *Gender (males, females)*: not reported *Length of surgery (minutes)*: not reported *Proportion of participants on anticoagulants prior to surgery (n/N, %)*: not reported *Incidence of preoperative anaemia (n/N, %)*: not reported *Co‐morbidities (n/N, %)*: not reported *ASA 1 (n/N, %)*: not reported *ASA 2 (n/N, %)*: not reported *ASA 3 (n/N, %)*: not reported *ASA 4 (n/N, %)*: not reported *Number of participants randomised*: not reported *Number of participants receiving treatment*: not reported *Number of participants analysed*: not reported *Dropout rate*: not reported **Inclusion criteria:** primary THR, ASA 1/2 **Exclusion criteria:** not reported **If TKR, is tourniquet used:** not applicable **Indication for surgery:** not reported **Type of anaesthetic:** general anaesthetic **Type of surgery:** primary THR	
Interventions	**Intervention characteristics** Placebo 2 x 10^6 KIU aprotinin during 30 minutes or normal saline in the same volume and time immediately after anaesthesia induction Placebo, IV, intraop (immediately after induction) Aprotinin 2 x 10^6 KIU aprotinin during 30 minutes or normal saline in the same volume and time immediately after anaesthesia induction Aprotinin, IV, 2 x 10^6 KIU, intraop (immediately after induction)	
Outcomes	*Primary outcomes:* Reduction of bleeding Transfusion requirements Incidence of deep vein thrombosis *Secondary outcome:* Not reported	
Notes	**Sponsorship source:** not reported **Country:** Spain **Setting:** single‐centre **Comments:** none **Author's name:** JV Llau **Institution:** Hospital Clinico Universitario **Email:** not reported **Address:** Anaesthesia and Critical Care Dept, Hospital Clinico Universitario Valencia, Spain **Native language of paper:** English **Reference type:** abstract (1) **Trial registration number:** not applicable, but when checked, trial registration number not found **Was it translated for this review:** no	
***Risk of bias***		
**Bias**	**Authors' judgement**	**Support for judgement**
Random sequence generation (selection bias)	Unclear risk	Quote: not applicable Judgement comment: not enough information to permit judgement.
Allocation concealment (selection bias)	Unclear risk	Quote: not applicable Judgement comment: insufficient information to permit judgement.
Blinding of participants and personnel (performance bias) Subjective outcomes	Unclear risk	Quote: not applicable Judgement comment: insufficient information to permit judgement.
Blinding of participants and personnel (performance bias) Objective outcomes	Low risk	Quote: not applicable Judgement comment: objective outcome for personnel, so low risk of bias regardless of quality of blinding.
Blinding of outcome assessment (detection bias) Subjective outcomes	Unclear risk	Quote: not applicable Judgement comment: insufficient information to permit judgement.
Blinding of outcome assessment (detection bias) Objective outcomes	Low risk	Quote: not applicable Judgement comment: objective outcome for outcome assessor, so low risk of bias regardless of quality of blinding.
Incomplete outcome data (attrition bias) All outcomes	Unclear risk	Quote: not applicable Judgement comment: the number of patients receiving treatment and those analysed is missing.
Selective reporting (reporting bias)	High risk	Quote: not applicable Judgement comment: demographic information not presented and these results would be expected to be reported.
Other bias	Unclear risk	Quote: not applicable Judgement comment: insufficient information to permit judgement. No funding source declared. Due to this being an abstract, some important information has not been included (maybe due to space restrictions).

**Table:** Lopez Picado 2017

***Study characteristics***		
Methods	**Study design:** RCT **Intention‐to‐treat analysis: n**ot reported **Duration of study:** 24 months **Power calculation reached:** yes **Transfusion strategy:** before starting the surgery, transfusion trigger levels were set at 8.5 g/dL of Hb under normovolemic conditions and at 9 g/dL in cases of moderate cardiac or respiratory diseases or symptoms of acute anaemia (angina, hypotension, dyspnoea). **Was the trial stopped early:** no	
Participants	**Baseline characteristics** Placebo *Age (years) (mean SD)*: 67.5 (12.1) *Ethnicity*: not reported *Gender (males, females)*: 21/37 M (57%); 16/37 F (43%) *Length of surgery (minutes) (mean SD)*: 65 (18) *Proportion of participants on anticoagulants prior to surgery (n/N, %)*: not reported *Incidence of preoperative anaemia (n/N, %)*: not reported *Co‐morbidities (n/N, %)*: not reported *ASA 1 (n/N, %)*: 12/37 (32.4) *ASA 2 (n/N, %)*: 22/37 (59.5) *ASA 3 (n/N, %)*: 3/37 (8.1) *ASA 4 (n/N, %)*: 0/0 (0) *Number of participants randomised*: 40 *Number of participants receiving treatment*: 39 *Number of participants analysed*: 37 *Dropout rate*: 3/40 (7.5%) TXA, 1 dose *Age (years) (mean SD)*: 69.2 (10.2) *Ethnicity*: not reported *Gender (males, females)*: 16/35 M (46%); 19/35 F (54%) *Length of surgery (minutes) (mean SD)*: 67 (19) *Proportion of participants on anticoagulants prior to surgery (n/N, %)*: not reported *Incidence of preoperative anaemia (n/N, %)*: not reported *Co‐morbidities (n/N, %)*: not reported *ASA 1 (n/N, %)*: 13/35 (37.2) *ASA 2 (n/N, %)*: 20/35 (57.1) *ASA 3 (n/N, %)*: 2/35 (5.7) *ASA 4 (n/N, %)*: 0/0 (0) *Number of participants randomised*: 38 *Number of participants receiving treatment*: 35 *Number of participants analysed*: 35 *Dropout rate*: 3/38 (7.89%) TXA, 2 doses *Age (years) (mean SD)*: 62.5 (13.0) *Ethnicity*: not reported *Gender (males, females)*: 20/36 M (56%); 16/36 F (44%) *Length of surgery (minutes) (mean SD)*: 68 (16) *Proportion of participants on anticoagulants prior to surgery (n/N, %)*: not reported *Incidence of preoperative anaemia (n/N, %)*: not reported *Co‐morbidities (n/N, %)*: not reported *ASA 1 (n/N, %)*: 12/36 (33.3) *ASA 2 (n/N, %)*: 24/36 (66.7) *ASA 3 (n/N, %)*: 0/36 (0) *ASA 4 (n/N, %)*: 0/0 (0) *Number of participants randomised*: 38 *Number of participants receiving treatment*: 37 *Number of participants analysed*: 36 *Dropout rate*: 2/38 (5.26%) Overall *Age (years) (mean SD)*: 66.4 (12.1) *Ethnicity*: not reported *Gender (males, females)*: 57, 51 *Length of surgery (minutes) (mean SD)*: 66 (17) *Proportion of participants on anticoagulants prior to surgery (n/N, %)*: not reported *Incidence of preoperative anaemia (n/N, %)*: not reported *Co‐morbidities (n/N, %)*: not reported *ASA 1 (n/N, %)*: 37/108 (34.3) *ASA 2 (n/N, %)*: 66/108 (61.6) *ASA 3 (n/N, %)*: 5/108 (4.6) *ASA 4 (n/N, %)*: 0/0 (0) *Number of participants randomised*: 113 *Number of participants receiving treatment*: 111 *Number of participants analysed*: 108 *Dropout rate*: 7/113 (6.2%) **Inclusion criteria:** all ASA physical status I to III patients older than 18 years with no known allergy to TXA were invited to participate. **Exclusion criteria:** 1) pregnancy or breastfeeding, 2) severe vascular ischaemia, 3) history of venous thrombosis, pulmonary embolism or diseases causing embolism, 4) known coagulopathies, 5) long‐term treatment with acetylsalicylic acid or nonsteroidal anti‐inflammatory drugs not discontinued before surgery, 6) a haemoglobin (Hb) concentration < 10 mg/dL, 7) moderate renal impairment, 8) liver cirrhosis, 9) any contraindications to prophylaxis with enoxaparin **If TKR, is tourniquet used:** not applicable **Indication for surgery:** arthrosis **Type of anaesthetic:** spinal **Type of surgery:** primary THR	
Interventions	**Intervention characteristics** Placebo Patients assigned to control group (CG) received an IV infusion of 100 mL of 0.9% saline over a 10‐minute period after instituting regional anaesthesia and before starting surgery. Three hours later, they received a further 100 mL of 0.9% saline over 10 minutes. Placebo, 100 mL, IV, preop AND placebo, 100 mL, IV, 3 hours postop TXA, 1 dose Patients assigned to single‐dose TXA group (SDG) received an IV infusion of 15 mg/kg TXA (Amchafibrin®, Rottapharm, Barcelona, Spain) in 100 mL of 0.9% saline over a 10‐minute period after the institution of regional anaesthesia and before the start of surgery. Three hours after the first infusion, they received a second infusion over 10 minutes, but this time with 100 mL of 0.9% saline alone. TXA, 15 mg/kg, IV, preop AND placebo, 100 mL, IV, 3 hours, postop TXA, 2 doses Patients assigned to this 2‐dose TXA group (TDG) received 10 mg/kg TXA (Amchafibrin®, Rottapharm, Barcelona, Spain). As in the single‐dose group, this was diluted in 100 mL of 0.9% saline and infused IV (intravenous) over 10 minutes, after instituting regional anaesthesia and before starting surgery, and 3 hours later after the start of surgery, they received a second infusion at the same dose and rate as the first. TXA 15 mg/kg, IV, preop AND TXA, 15 mg/kg, IV, 3 hours postop	
Outcomes	*Primary outcomes:* Total blood loss at 48 hours Blood transfusions given *Secondary outcomes:* Blood loss up to 1 hour and 6 hours after surgery Intra (in the operating theatre) and postoperative blood loss Rates of thrombosis Complications	
Notes	**Sponsorship source:** none **Country:** Spain **Setting:** multi‐centre **Comments:** none **Author's name:** B Barrachina **Institution:** Araba University Hospital **Email:** borjabarra@gmail.com **Address:** Servicio de Anestesiología y Reanimación, Hospital Universitario de Araba‐ Sede Txagorritxu, c/ Jose Atxotegui s/n, 01009 Vitoria‐Gasteiz, Alava, Spain **Native language of paper:** English **Reference type:** full text (2), trial registration (1) **Trial registration number:** NCT01199627 **Was it translated for this review:** no	
***Risk of bias***		
**Bias**	**Authors' judgement**	**Support for judgement**
Random sequence generation (selection bias)	Low risk	Quote: "Patients were randomly allocated to 1 of 3 groups using a computer‐generated random number list held in the Araba Research Unit, hidden from participating clinicians." Judgement comment: computer‐generated random number list.
Allocation concealment (selection bias)	Low risk	Quote: "To keep participating clinicians blind to patient allocation, study medication was prepared by the pharmacies of the participating hospitals in compliance with current regulations. At the time of surgery, the pharmacy delivered 100‐mL bags labeled with patient codes; depending on their allocation, bags contained only saline or saline with 1 of 2 doses of TXA but given that TXA solution is colorless, and the different bags were indistinguishable." Judgement comment: pharmacy‐controlled randomisation.
Blinding of participants and personnel (performance bias) Subjective outcomes	Low risk	Quote: "At the time of surgery, the pharmacy delivered 100‐mL bags labeled with patient codes; depending on their allocation, bags contained only saline or saline with 1 of 2 doses of TXA but given that TXA solution is colorless, and the different bags were indistinguishable." Quote: "To keep participating clinicians blind to patient allocation, study medication was prepared by the pharmacies of the participating hospitals in compliance with current regulations." Judgement comment: blinding or participants and key study personnel ensured, and unlikely that the blinding could have been broken.
Blinding of participants and personnel (performance bias) Objective outcomes	Low risk	Quote: not applicable Judgement comment: objective outcome for personnel, so low risk of bias regardless of quality of blinding.
Blinding of outcome assessment (detection bias) Subjective outcomes	Low risk	Quote: "To keep participating clinicians blind to patient allocation, study medication was prepared by the pharmacies of the participating hospitals in compliance with current regulations." Judgement comment: likely blinding of outcome assessor obtained.
Blinding of outcome assessment (detection bias) Objective outcomes	Low risk	Quote: "To keep participating clinicians blind to patient allocation, study medication was prepared by the pharmacies of the participating hospitals in compliance with current regulations." Judgement comment: objective outcome for outcome assessor, so low risk of bias regardless of quality of blinding.
Incomplete outcome data (attrition bias) All outcomes	Unclear risk	Quote: not applicable Judgement comment: no clear explanation why 38 patients randomised to receive single TXA, however only 35 received assigned intervention. Also, figure 1 states 113 were randomised but 38 + 38+ 40 = 116 not 113.
Selective reporting (reporting bias)	High risk	Quote: not applicable Judgement comment: not all of the study's pre‐specified primary outcomes have been reported. No secondary outcomes specified in trial registration.
Other bias	Unclear risk	Quote: not applicable Judgement comment: insufficient information to permit judgement.

**Table:** Luo 2022

***Study characteristics***		
Methods	**Study design:** RCT, 3‐arm parallel trial (only 2 arms included within the review as the 3rd arms had an additional intervention that was not included in this review) **Intention‐to‐treat analysis:** yes (no dropouts) **Duration of study:** January to April 2019 + 2 months follow‐up = 5 months **Power calculation reached:** yes **Transfusion strategy:** according to the guidelines for perioperative blood transfusion provided by the ministry of health of China, blood transfusion is required when haemoglobin concentration < 70 g/L or the patient presents any anaemic organ dysfunction, such as mental state changes or heart palpitations (regardless of haemoglobin concentration). **Was the trial stopped early:** no	
Participants	**Baseline characteristics** TXA, IV *Age (years) (mean SD)*: 59.40 (11.52) *Ethnicity*: not reported *Gender (males, females)*: 23/50 (46%) M, 27/50 (54%) F *Length of surgery (minutes)*: 59.47 (11.40) *Proportion of participants on anticoagulants prior to surgery (n/N, %)*: not reported *Incidence of preoperative anaemia (n/N, %)*: excluded *Co‐morbidities (n/N, %)*: not reported *ASA 1 (n/N, %)*: 7/50 (14%) *ASA 2 (n/N, %)*: 30/50 (60%) *ASA 3 (n/N, %)*: 13/50 (26%) *ASA 4 (n/N, %)*: 0/50 (0%) *Number of participants randomised*: 50 *Number of participants receiving treatment*: 50 *Number of participants analysed*: 50 *Dropout rate*: 0/50, 0% Placebo *Age (years) (mean SD)*: 59.26 (10.71) *Ethnicity*: not reported *Gender (males, females)*: 25/50 (50%) M, 25/50 (50%) F *Length of surgery (minutes)*: 61.70 (9.09) *Proportion of participants on anticoagulants prior to surgery (n/N, %)*: not reported *Incidence of preoperative anaemia (n/N, %)*: excluded *Co‐morbidities (n/N, %)*: not reported *ASA 1 (n/N, %)*: 4/50 (8%) *ASA 2 (n/N, %)*: 35/50 (70%) *ASA 3 (n/N, %)*: 11/50 (22%) *ASA 4 (n/N, %)*: 0/50 (0%) *Number of participants randomised*: 50 *Number of participants receiving treatment*: 50 *Number of participants analysed*: 50 *Dropout rate*: 0/50 (0%) Overall *Age (years) (mean SD)*: not reported *Ethnicity*: not reported *Gender (males, females)*: 48/100 (48%) M, 52/100 (52%) F *Length of surgery (minutes)*: not reported *Proportion of participants on anticoagulants prior to surgery (n/N, %)*: not reported *Incidence of preoperative anaemia (n/N, %)*: excluded *Co‐morbidities (n/N, %)*: not reported *ASA 1 (n/N, %)*: 11/100, (11%) *ASA 2 (n/N, %)*: 65/100, (65%) *ASA 3 (n/N, %)*: 24/100, (24%) *ASA 4 (n/N, %)*: 0/100, (0%) *Number of participants randomised*: 100 *Number of participants receiving treatment*: 100 *Number of participants analysed*: 100 *Dropout rate*: 0/100 (0%) **Inclusion criteria:** all patients diagnosed with hip osteoarthritis or femoral head necrosis (Ficat III or IV) scheduled for unilateral primary total hip arthroplasty using direct anterior approach (DAA) between January 2019 and April 2019 were eligible for this trial **Exclusion criteria:** patients with a history of renal failure, renal transplantation, arterial thromboembolism (e.g. myocardial infarction or stroke), arterial stenting or deep vein thrombosis or pulmonary embolism, or acetabular posterior wall defect, Crowe type 3 or 4 dysplasia osteoarthritis and low haemoglobin levels (< 110 g/L) were excluded. We also excluded people with allergies to TXA, CSS, anaesthetic drugs, and patients who refused to participate in or agree to receive blood products. We also excluded patients with a body mass index (BMI) greater than 30 kg/m^2^. **If TKR, is tourniquet used:** not applicable **Indication for surgery:** hip osteoarthritis or femoral head necrosis (Ficat III or IV) **Type of anaesthetic:** general **Type of surgery:** unilateral primary total hip arthroplasty using direct anterior approach (DAA)	
Interventions	**Intervention characteristics** TXA, IV 1 g of TXA was given intravenously 5 minutes before the skin incision, and 60 mL of mixed fluid (containing 40 mg placebo (glucose powder) + 60 mL saline) was injected around the joint capsule before the joint capsule was closed, the same as group A TXA, IV, 1 g, intraop + 60 mL mixed fluid, topical, intraop Placebo The same dose (100 mL, 0.9% normal saline) of saline solution was injected intravenously 5 minutes before the skin incision, and 60 mL mixed fluid (containing 40 mg placebo + 60 mL normal saline) was topically administered. Placebo, IV, 1 g, intraop, + 60 mL mixed fluid, topical, intraop A third arm (TXA plus topical carbazochrome sodium sulfonate was included in the trial but was excluded from the review).	
Outcomes	*Primary outcome:* Total blood loss *Secondary outcomes:* Hidden blood loss Intraoperative blood loss Reduction in haemoglobin concentration Coagulation parameters Inflammatory marker levels Perioperative visual analogue scale (VAS) pain score Transfusion rates Number of blood transfusion units Postoperative hospital stay Incidence of thromboembolic events Other complications	
Notes	**Sponsorship source:** non‐pharmaceutical (science and technology program of Sichuan Province. Grant ID: 2019YFS0123) **Country:** China **Setting:** not reported **Comments:** 1) only extracted groups B and C2. The types of hip joint diseases included in this study have selection bias. Other hip joint diseases such as rheumatoid arthritis and traumatic arthritis were excluded. Therefore, these results do not apply to all hip joint diseases. **Author's name:** Y Luo **Institution:** Sichuan University **Email:** corresponding author: P. Kang ‐ kangpengde69@126.com **Address:** Department of Orthopaedics, West China Hospital, Sichuan University, 37# Wainan Guoxue Road, 610041 Chengdu, People’s Republic of China **Native language of paper:** English **Reference type:** full text (1) **Trial registration number:** ChiCTR1900020498 **Was it translated for this review:** no	
***Risk of bias***		
**Bias**	**Authors' judgement**	**Support for judgement**
Random sequence generation (selection bias)	Low risk	Quote: "Patients included in the study were randomized into three groups, using a computer‐generated randomized table ..." Judgement comment: adequate method of sequence generation with computer‐generated random numbers.
Allocation concealment (selection bias)	Unclear risk	Quote: "A research assistant, who was not involved in the data collection, hid the computer‐generated randomly assigned sequence in a serially‐numbered sealed envelope" Judgement comment: envelopes not described as sealed, opaque and sequentially numbered.
Blinding of participants and personnel (performance bias) Subjective outcomes	Unclear risk	Quote: "The patients, the anesthesiologists, the attending physicians, the pharmacists, the medical providers, and all outcome assessors were blind to randomization." Judgement comment: adequate methods of masking and blinding were used.
Blinding of participants and personnel (performance bias) Objective outcomes	Low risk	Quote: not applicable Judgement comment: objective outcome for personnel, so low risk of bias regardless of quality of blinding.
Blinding of outcome assessment (detection bias) Subjective outcomes	Unclear risk	Quote: "The patients, the anesthesiologists, the attending physicians, the pharmacists, the medical providers, and all outcome assessors were blind to randomization." Judgement comment: outcome assessors were blinded.
Blinding of outcome assessment (detection bias) Objective outcomes	Low risk	Quote: not applicable Judgement comment: objective outcome for outcome assessor, so low risk of bias regardless of quality of blinding.
Incomplete outcome data (attrition bias) All outcomes	Low risk	Quote: not applicable Judgement comment: no obvious outcome data missing.
Selective reporting (reporting bias)	Low risk	Quote: not applicable Judgement comment: all outcomes planned in the protocol or prospective trial registration are reported.
Other bias	Unclear risk	Quote: not applicable Judgement comment: some hip diseases excluded.

**Table:** Molloy 2007

***Study characteristics***		
Methods	**Study design:** RCT **Intention‐to‐treat analysis:** yes **Duration of study:** 10 months + FU **Power calculation reached:** yes **Transfusion strategy:** the postoperative haematocrit measured at 8 hours determined if a transfusion was indicated from a protocol with a threshold of 0.25. **Was the trial stopped early:** no	
Participants	**Baseline characteristics** TXA *Age (years) (mean SD)*: not reported *Ethnicity*: not reported *Gender (males, females)*: not reported *Length of surgery (minutes)*: not reported *Proportion of participants on anticoagulants prior to surgery (n/N, %)*: 50/50, 100% *Incidence of preoperative anaemia (n/N, %)*: not reported *Co‐morbidities (n/N, %)*: not reported *ASA 1 (n/N, %)*: not reported *ASA 2 (n/N, %)*: not reported *ASA 3 (n/N, %)*: not reported *ASA 4 (n/N, %)*: not reported *Number of participants randomised*: 50 *Number of participants receiving treatment*: 50 *Number of participants analysed*: 50 *Dropout rate*: 0/50, (0%) Fibrin spray *Age (years) (mean SD)*: not reported *Ethnicity*: not reported *Gender (males, females)*: not reported *Length of surgery (minutes)*: not reported *Proportion of participants on anticoagulants prior to surgery (n/N, %)*: 50/50, 100% *Incidence of preoperative anaemia (n/N, %)*: not reported *Co‐morbidities (n/N, %)*: not reported *ASA 1 (n/N, %)*: not reported *ASA 2 (n/N, %)*: not reported *ASA 3 (n/N, %)*: not reported *ASA 4 (n/N, %)*: not reported *Number of participants randomised*: 50 *Number of participants receiving treatment*: 50 *Number of participants analysed*: 50 *Dropout rate*: 0/50, (0%) Overall *Age (years) (mean SD)*: not reported *Ethnicity*: not reported *Gender (males, females)*: not reported *Length of surgery (minutes)*: not reported *Proportion of participants on anticoagulants prior to surgery (n/N, %)*: not reported *Incidence of preoperative anaemia (n/N, %)*: not reported *Co‐morbidities (n/N, %)*: not reported *ASA 1 (n/N, %)*: not reported *ASA 2 (n/N, %)*: not reported *ASA 3 (n/N, %)*: not reported *ASA 4 (n/N, %)*: not reported *Number of participants randomised*: not reported *Number of participants receiving treatment*: not reported *Number of participants analysed*: not reported *Dropout rate*: not reported **Inclusion criteria:** 1) patients scheduled to undergo a primary TKR, 2) pre‐operative haemoglobin (Hb) level of 13.0 g/dL **Exclusion criteria:** 1) previous surgery to the knee, with the exception of meniscectomy, 2) bleeding disorders, platelet or bone marrow disorders, 3) a level of creatinine > 250 μmol/L since this is a contraindication to the administration of tranexamic acid, 4) a history of thromboembolism **If TKR, is tourniquet used:** yes **Indication for surgery:** NR **Type of anaesthetic:** spinal **Type of surgery:** primary TKR	
Interventions	**Intervention characteristics** TXA Those randomised to the tranexamic group received 500 mg of tranexamic acid intravenously 5 minutes before deflation of the tourniquet and a repeat dose 3 hours later TXA, IV, 0.5 mg, intraop (5 mins prior to tourniquet deflation) AND TXA, IV, 0.5 mg, postop 3 hours after 1st dose Fibrin spray Those patients randomised to the topical fibrin group received 10 mL of the reconstituted product intraoperatively, with 6 mL sprayed on to the posterior capsule and surrounding soft tissues before the prosthesis was inserted, and the remaining 4 mL sprayed on to the bone, which was exposed after placement of the prosthesis and on the soft tissues after closure of the capsule. Fibrin, IA, 10 mL, intraop ‐ 6 mL before prosthesis inserted, 4 mL to exposed bone after prosthesis placement and soft tissue	
Outcomes	*Primary outcome:* Calculated total blood loss *Secondary outcomes:* Transfusion requirements Postoperative leg swelling Pain scores Length of stay in hospital Rate of complications Rate of proximal deep venous thrombosis (DVT)	
Notes	**Sponsorship source:** pharmaceutical **Country:** UK **Setting:** not reported **Comments:** none **Author's name:** D Molloy **Institution:** Musgrave Park Hospital **Email:** dennis.molloy@greenpark.n‐i.nhs.uk **Address:** Orthopaedic Outcomes Department, Musgrave Park Hospital, Stockmans Lane, Belfast, BT9 7JB, Northern Ireland **Native language of paper:** English **Reference type:** abstract (1) full text (1) **Trial registration number:** not applicable, but when checked, trial registration number not found **Was it translated for this review:** no	
***Risk of bias***		
**Bias**	**Authors' judgement**	**Support for judgement**
Random sequence generation (selection bias)	Low risk	Quote: "The patients were randomised using a block‐design technique with permutated blocks of five, which was concealed until interventions" Judgement comment: block randomisation table.
Allocation concealment (selection bias)	Unclear risk	Quote: "which was concealed until interventions were assigned." Judgement comment: insufficient information to permit judgement
Blinding of participants and personnel (performance bias) Subjective outcomes	High risk	Quote: not applicable Judgement comment: different treatment regimens, therefore key study personnel not blinded to study intervention.
Blinding of participants and personnel (performance bias) Objective outcomes	Low risk	Quote: not applicable Judgement comment: objective outcome for personnel, so low risk of bias regardless of quality of blinding.
Blinding of outcome assessment (detection bias) Subjective outcomes	Low risk	Quote: "All the patients and staff, except those directly administering the topical fibrin spray or tranexamic acid, were blinded to the treatment, including those assessing and collecting the post‐ operative measurements, investigations and outcomes." Judgement comment: outcome assessor blinding described.
Blinding of outcome assessment (detection bias) Objective outcomes	Low risk	Quote: not applicable Judgement comment: objective outcome for outcome assessor, so low risk of bias regardless of quality of blinding.
Incomplete outcome data (attrition bias) All outcomes	Unclear risk	Quote: not applicable Judgement comment: insufficient information to permit judgement. No n numbers for the table of results.
Selective reporting (reporting bias)	Unclear risk	Quote: not applicable Judgement comment: no protocol available to check pre‐specified outcome measures reported.
Other bias	High risk	Quote: not applicable Judgement comment: basic information is missing such as average mean age of patients and gender split per arm or ASA. No n numbers in tables reported.

**Table:** Morales‐Avalos 2021

***Study characteristics***		
Methods	**Study design:** RCT **Intention‐to‐treat analysis:** yes **Duration of study:** 4 months (February 2020 to June 2020) + 90 days (follow‐up) = 7 months **Power calculation reached:** yes **Transfusion strategy:** blood transfusion was performed in case a patient had either a Hb level less than 7 g/dL or evolved with any anaemia‐related organ dysfunction (palpitation, shortness of breath or bad mental status not due to other causes) with a Hb level between 7 and 10 g/dL **Was the trial stopped early:** no	
Participants	f**Baseline characteristics** TXA, oral, 1.3 g, 2 h pre‐incision, 6 and 12 h postop *Age (years) (mean SD)*: 66.2 (11.23) *Ethnicity*: not reported *Gender (males, females)*: 24/51 M (47.05%); 27/51 F (52.95%) *Length of surgery (minutes) (mean, SD)*: 68.44 (10.23) *Proportion of participants on anticoagulants prior to surgery (n/N, %)*: 0/51, 0% *Incidence of preoperative anaemia (n/N, %)*: 0/51, 0% *Co‐morbidities (n/N, %)*: diabetes mellitus 16/51 (31.37%); hypertension 19/51 (37.25%); rheumatoid arthritis 5/51 (9.80%); history of contralateral total hip replacement 7/51 (13.73%); dyslipidaemia 2/51 (3.92%); another systemic disorder (asthma, COPD, cancer, chronic renal failure, chronic liver disease, ischaemic stroke or haemorrhagic stroke, osteoporosis, etc.) 13/51 (25.49%) *ASA 1 (n/N, %)*: 3/51 (5.88%) *ASA 2 (n/N, %)*: 47/51 (92.16%) *ASA 3 (n/N, %)*: 1/51 (1.96%) *ASA 4 (n/N, %)*: 0/51, (0%) *Number of participants randomised*: 51 *Number of participants receiving treatment*: 51 *Number of participants analysed*: 51 *Dropout rate*: 0/51, 0% EACA, oral, 2 g, 2 h pre‐incision, 6 and 12 h postop *Age (years) (mean SD)*: 64.5 (9.18) *Ethnicity*: not reported *Gender (males, females)*: 26/51 M (50.98%); 25/51 F (49.02%) *Length of surgery (minutes)*: 65.34 (11.34) *Proportion of participants on anticoagulants prior to surgery (n/N, %)*: not reported *Incidence of preoperative anaemia (n/N, %)*: not reported *Co‐morbidities (n/N, %)*: diabetes mellitus (n/%) 15/51 (29.41%); hypertension 17/51 (33.33%); rheumatoid arthritis 7/51 (13.73%); history of contralateral total hip replacement 9/51 (17.64%); dyslipidaemia 4/51 (7.84%); another systemic disorder (asthma, COPD, cancer, chronic renal failure, chronic liver disease, ischaemic stroke or hemorrhagic stroke, osteoporosis, etc.) 11/51 (21.57%) *ASA 1 (n/N, %)*: 5/51 9.80% *ASA 2 (n/N, %)*: 44/51 86.28% *ASA 3 (n/N, %)*: 2/51 3.92% *ASA 4 (n/N, %)*: 0/51, 0% *Number of participants randomised*: 51 *Number of participants receiving treatment*: 51 *Number of participants analysed*: 51 *Dropout rate*: 0/51, 0% Overall *Age (years) (mean SD)*: not reported *Ethnicity*: not reported *Gender (males, females)*: 50 M, 52 F *Length of surgery (minutes)*: not reported *Proportion of participants on anticoagulants prior to surgery (n/N, %)*: not reported *Incidence of preoperative anaemia (n/N, %)*: not reported *Co‐morbidities (n/N, %)*: not reported *ASA 1 (n/N, %)*: not reported *ASA 2 (n/N, %)*: not reported *ASA 3 (n/N, %)*: not reported *ASA 4 (n/N, %)*: not reported *Number of participants randomised*: 102 *Number of participants receiving treatment*: 102 *Number of participants analysed*: 102 *Dropout rate*: 0/102, 0% **Inclusion criteria:** all patients over 18 years; undergoing cementless primary unilateral THA for osteoarthritis, osteonecrosis of the femoral head (Ficat III or IV), or developmental dysplasia of hip (Crowe I/II); ASA I–III **Exclusion criteria:** patients with anaemia (< 120 g/L for female, < 130 g/L for male); patients with bilateral arthroplasty; planned revision surgery; developmental dysplasia of the hip (Crowe III/IV); prosthetic surgery for a fracture of the femoral head or neck or acetabulum; infected patients; pregnancy; lactating patients; use of oral contraceptives; a BMI more than 35 kg/m^2^; history of an arterial thrombotic event such as stroke or myocardial infarction in the last year; placement of an arterial stent within the last year; history of deep vein thrombosis (DVT) or pulmonary embolism; history of congenital or acquired clotting disorder; family history of thrombotic conditions; medication history of anticoagulant or antiplatelet prophylaxis in the perioperative period; complicated primary THAs with osteotomy; an existing implant removal or bone grafting; kidney failure; kidney transplant; anaphylaxis to either EACA or TXA; declined to consent receiving blood products; unable to ingest or receive the medication orally **If TKR, is tourniquet used:** not applicable **Indication for surgery:** osteoarthritis, osteonecrosis of the femoral head, developmental dysplasia of hip **Type of anaesthetic:** mixed general and spinal (for TXA spinal (n/%) 47 (92.16); for EACA 48 (94.12); for TXA general (n/%) 4 (7.84); for EACA general 2 (3.92) 0.555) **Type of surgery:** primary THA	
Interventions	**Intervention characteristics** TXA, oral, 1.3 g, 2 h pre incision, 6 and 12 h postop The oral TXA group received 1.3 g of TXA (2 tablets of 650 mg) (Lysteda, Pierre Fabre, Mexico City, Mexico) approximately 2 h before the incision, and the same dose was repeated 6 and 12 h postoperatively. TXA, oral, 1.3 g, 2 h pre‐incision, 6 and 12 h postop EACA, oral, 2 g, 2 h pre‐incision, 6 and 12 h postop The oral EACA group received 2 g of EACA (2 tablets of 1000 mg) (Amikar, Wyeth, Quebec, Canada) at the same times previously described (approximately 2 h before the incision, and the same dose was repeated 6 and 12 h postoperatively). The oral EACA group received 2 g of EACA (2 tablets of 1000 mg) (Amikar, Wyeth, Quebec, Canada) at the same times previously described.	
Outcomes	*Primary outcomes*: Total blood loss Hidden blood loss External blood loss Transfusion rate *Secondary outcomes:* Transfusion rate IBL Hb and Hct drop Drain output Readmission Mortality Pain indicated by the patients using a visual analogue scale (VAS) Functionality using the Harris hip score (HHS) Range of motion (these last 3 as indirect indicators of functional deficit and pain secondary to the presence of intra‐articular bleeding) Adverse effects Complications	
Notes	**Sponsorship source:** not reported (there is no funding source) **Country:** Mexico **Setting:** single‐centre **Comments:** none **Author's name:** R Morales‐Avalos **Institution:** Universidad Autonoma de Nuevo Leon (U.A.N.L.), Monterrey **Email:** rodolfot59@hotmail.com **Address:** Hip Unit, Department of Orthopedic Surgery and Traumatology, 4th Floor of the Central Building, School of Medicine and University Hospital ‘Dr Jose Eleuterio Gonzaez’, Universidad Autonoma de Nuevo Leon (U.A.N.L.), Av. Francisco I. Madero and Dr Edua **Native language of paper:** English **Reference type:** full text (1), trial registration (1) **Trial registration number:** NCT04187014 **Was it translated for this review:** no	
***Risk of bias***		
**Bias**	**Authors' judgement**	**Support for judgement**
Random sequence generation (selection bias)	Low risk	Quote: "To get a stratified randomized schedule we used a computer to develop random number tables" Judgement comment: adequate method of sequence generation with computer‐generated random numbers.
Allocation concealment (selection bias)	Unclear risk	Quote: "Randomization was done blind and with help of closed envelopes at a ratio of 1: 1 that were eventually opened before the surgical procedure." Judgement comment: envelopes not described as sealed, opaque and sequentially numbered.
Blinding of participants and personnel (performance bias) Subjective outcomes	Low risk	Quote: "For the development of this study, researchers and patients who worked and gathered the clinical data and information were blinded to patient allocation until the data was analyzed. We prepared the medication used in the study in generic equal bottles and gave them numbered in a randomized schedule for each patient. Nurses were in charge to manage and give the medication and were not involved in this trail." Judgement comment: patients and personnel were blinded.
Blinding of participants and personnel (performance bias) Objective outcomes	Low risk	Quote: "For the development of this study, researchers and patients who worked and gathered the clinical data and information were blinded to patient allocation until the data was analyzed." Judgement comment: patients and personnel were blinded.
Blinding of outcome assessment (detection bias) Subjective outcomes	Low risk	Quote: "For the development of this study, researchers and patients who worked and gathered the clinical data and information were blinded to patient allocation until the data was analyzed. We prepared the medication used in the study in generic equal bottles and gave them numbered in a randomized schedule for each patient. Nurses were in charge to manage and give the medication and were not involved in this trail." Judgement comment: none
Blinding of outcome assessment (detection bias) Objective outcomes	Low risk	Quote: not applicable Judgement comment: objective outcome for personnel, so low risk of bias regardless of quality of blinding.
Incomplete outcome data (attrition bias) All outcomes	Low risk	Quote: not applicable Judgement comment: no obvious outcome data missing.
Selective reporting (reporting bias)	Low risk	Quote: not applicable Judgement comment: all outcomes planned in the protocol or prospective trial registration are reported.
Other bias	Low risk	Quote: not applicable Judgement comment: no other concerns such as early stopping or imbalanced study arms.

**Table:** Murkin 1995

***Study characteristics***		
Methods	**Study design:** RCT **Intention‐to‐treat analysis:** yes **Duration of study:** 17 months **Power calculation reached:** no **Transfusion strategy:** transfusion criteria for packed red blood cells (PRBC) were defined prospectively. PRBC transfusion was permitted for intraoperative blood loss exceeding 15% of blood volume, where blood volume was estimated as kilograms of body weight (BW) x 70 mL for patients younger than 60 yr, and BW x 65 mL for males and BW x 60 mL for females 60 yr or older, or for postoperative hemoglobin concentration less than 8.0 g/dL. **Was the trial stopped early: n**o	
Participants	**Baseline characteristics** Placebo *Age (years) (mean SEM)*: 65.5 (3.4) *Ethnicity*: not reported *Gender (males, females)*: 11/24 M (46%); 13/24 F (54%) *Length of surgery (minutes) (mean SEM)*: 194 (11.0) *Proportion of participants on anticoagulants prior to surgery (n/N, %)*: 4/24 (16.7%) *Incidence of preoperative anaemia (n/N, %)*: not reported *Co‐morbidities (n/N, %)*: diabetes mellitus 0/24 (0%), preoperative hypertension 1/24 (4.2%) *ASA 1 (n/N, %)*: not reported *ASA 2 (n/N, %)*: not reported *ASA 3 (n/N, %)*: not reported *ASA 4 (n/N, %)*: not reported *Number of participants randomised*: 24 *Number of participants receiving treatment*: 24 *Number of participants analysed*: 24 *Dropout rate*: 0/24 (0%) Aprotinin *Age (years) (mean SEM)*: 66.9 (2.8) *Ethnicity*: not reported *Gender (males, females)*: 9/29 M (31%); 20/29 F (69%) *Length of surgery (minutes) (mean SEM)*: 180 (7.5) *Proportion of participants on anticoagulants prior to surgery (n/N, %)*: 7/29 (24.1%) *Incidence of preoperative anaemia (n/N, %)*: not reported *Co‐morbidities (n/N, %)*: diabetes mellitus 6/29 (20.7%), preoperative hypertension 2/29 (6.9%) *ASA 1 (n/N, %)*: not reported *ASA 2 (n/N, %)*: not reported *ASA 3 (n/N, %)*: not reported *ASA 4 (n/N, %)*: not reported *Number of participants randomised*: 29 *Number of participants receiving treatment*: 29 *Number of participants analysed*: 29 *Dropout rate*: 0/29 (0%) Overall *Age (years) (mean SEM)*: not reported *Ethnicity*: not reported *Gender (males, females)*: not reported *Length of surgery (minutes) (mean SEM)*: not reported *Proportion of participants on anticoagulants prior to surgery (n/N, %)*: not reported *Incidence of preoperative anaemia (n/N, %)*: not reported *Co‐morbidities (n/N, %)*: not reported *ASA 1 (n/N, %)*: not reported *ASA 2 (n/N, %)*: not reported *ASA 3 (n/N, %)*: not reported *ASA 4 (n/N, %)*: not reported *Number of participants randomised*: 53 *Number of participants receiving treatment*: not reported *Number of participants analysed*: not reported *Dropout rate*: not reported **Inclusion criteria:** 1) patients undergoing revision THA or primary bilateral THA **Exclusion criteria:** 1) previously known allergy or exposure to aprotinin, 2) history of pancreatitis, 3) impaired renal function defined as increased serum creatinine > 150 pmol/L for females or > 170 pmol/L for males, 4) biochemical evidence of hepatic dysfunction, 5) age < 18 yr **If TKR, is tourniquet used:** not applicable **Indication for surgery:** not reported **Type of anaesthetic:** general anaesthesia **Type of surgery:** revision THR and bilateral THR	
Interventions	**Intervention characteristics** Placebo Placebo‐treated patients received an equivalent volume of 0.9% saline. Test dose in both arms: placebo, IV, intraop, bolus AND placebo, IV, infusion until 1 hour post surgery Aprotinin Prior to induction of anaesthesia, a test dose of 5 mL of study drug was administered over 5 min followed by a continuous infusion for the duration of surgery and for 1 h postoperatively. Patients weighing between 60 and 80 kg, and randomised to receive aprotinin, were administered a loading dose of 2 million kallikrein inactivation units (KIU) (200 mL) over 15 min followed by an infusion of 0.5 million KIU (50 mL) per hour; those weighing less than 60 kg or more than 80 kg received a loading dose of 2.8 mL/kg (10,000 KIU/mL) and an infusion of 0.7 mL • kg^‐1^• h^‐1^. Test dose in both arms: for 60 to 80 kg people: aprotinin, IV, 2 x 10^6 KIU, intraop, bolus AND aprotinin, IV, 0.5 x 10^6 KIU infusion until 1 hour post surgery OR for people 60 to 80 kg: aprotinin, IV, 2.8 mL/kg (10,000 KIU/mL), intraop AND aprotinin, IV, 0.7 mL/kg, infusion until 1 hour postop	
Outcomes	*Primary outcomes:* Intraoperative blood loss Amount of blood products transfused Incidence of deep venous thrombosis Total blood loss Biochemical markers of hepatic and renal function *Secondary outcome:* Not reported	
Notes	**Sponsorship source:** unclear (searched for 'Miles Canada Inc' ‐ but no relevant hits) **Country:** Canada **Setting:** not reported **Comments:** none **Author's name:** JM Murkin **Institution:** University of Western Ontario **Email:** not reported **Address:** Department of Anesthesia, University Hospital, University of Western Ontario, 339 Windermere Road, London, Ontario, Canada N6A 5A5 **Native language of paper:** English **Reference type:** full text (1) **Trial registration number:** not applicable, but when checked, trial registration number not found **Was it translated for this review:** no	
***Risk of bias***		
**Bias**	**Authors' judgement**	**Support for judgement**
Random sequence generation (selection bias)	Low risk	Quote: "Using a computer‐generated random code," Judgement comment: computer‐generated randomisation code.
Allocation concealment (selection bias)	Unclear risk	Quote: "patients were assigned to receive either aprotinin or the equivalent volume of saline placebo administered from uniformly blinded bottles." Judgement comment: insufficient information to permit judgement.
Blinding of participants and personnel (performance bias) Subjective outcomes	Low risk	Quote: "Using a computer‐generated random code, patients were assigned to receive either aprotinin or the equivalent volume of saline placebo administered from uniformly blinded bottles. Prior to induction of anesthesia, a test dose of 5 mL" Judgement comment: likely blinding of key personnel and participants.
Blinding of participants and personnel (performance bias) Objective outcomes	Low risk	Quote: not applicable Judgement comment: objective outcome for personnel, so low risk of bias regardless of quality of blinding.
Blinding of outcome assessment (detection bias) Subjective outcomes	Unclear risk	Quote: not applicable Judgement comment: no description of outcome assessor blinding.
Blinding of outcome assessment (detection bias) Objective outcomes	Low risk	Quote: not applicable Judgement comment: objective outcome for outcome assessor, so low risk of bias regardless of quality of blinding.
Incomplete outcome data (attrition bias) All outcomes	Low risk	Quote: not applicable Judgement comment: all outcome data presented.
Selective reporting (reporting bias)	Unclear risk	Quote: not applicable Judgement comment: no protocol to check pre‐specified outcome measures reported.
Other bias	Unclear risk	Quote: not applicable Judgement comment: insufficient information to permit judgement.

**Table:** Murkin 2000

***Study characteristics***		
Methods	**Study design:** RCT **Intention‐to‐treat analysis:** no **Duration of study:** not reported **Power calculation reached:** no **Transfusion strategy:** patients had transfusion of whole blood or packed red blood cells if the postoperative haematocrit was 18% or less or if clinically warranted. **Was the trial stopped early:** no	
Participants	**Baseline characteristics** Placebo *Mean age (years) (mean)*: 63.2 *Ethnicity*: White: 64 (94%); Black: 2 (3%); Hispanic: 2 (3%) *Gender (males, females)*: 32/68 M (47%); 36/68 F (53%) *Mean operating time (minutes) (mean)*: 69 *Proportion of participants on anticoagulants prior to surgery (n/N, %)*: not reported *Incidence of preoperative anaemia (n/N, %)*: 0/73 (0%) *Co‐morbidities (n/N, %)*: not reported *ASA 1 (n/N, %)*: not reported *ASA 2 (n/N, %)*: not reported *ASA 3 (n/N, %)*: not reported *ASA 4 (n/N, %)*: not reported *Number of participants randomised*: 73 *Number of participants receiving treatment*: 68 *Number of participants analysed*: 68 *Dropout rate*: 5/73 (6.9%) Aprotinin 500,000 KIU (low dose) *Mean age (years) (mean)*: 63.7 *Ethnicity* : White: 66(96%); Black: 2 (3%); Hispanic: 1 (1%) *Gender (males, females)*: 34/69 M (49%); 35/69 F (51%) *Mean operating time (minutes) (mean)*: 67 *Proportion of participants on anticoagulants prior to surgery (n/N, %)*: not reported *Incidence of preoperative anaemia (n/N, %)*: 0/76 (0%) *Co‐morbidities (n/N, %)*: not reported *ASA 1 (n/N, %)*: not reported *ASA 2 (n/N, %)*: not reported *ASA 3 (n/N, %)*: not reported *ASA 4 (n/N, %)*: not reported *Number of participants randomised*: 76 *Number of participants receiving treatment*: 69 *Number of participants analysed*: 69 *Dropout rate*: 7/76 (9.2%) Aprotinin 250,000 KIU per hour (medium dose) *Mean age (years) (mean)*: 65.5 *Ethnicity* : White: 68 (100%); Black: 0 (0%); Hispanic: 0 (0%) *Gender (males, females)*: 27/68 M (40%); 41/68 F (60%) *Mean operating time (minutes)*: 67 *Proportion of participants on anticoagulants prior to surgery (n/N, %)*: not reported *Incidence of preoperative anaemia (n/N, %)*: 0/75 (0%) *Co‐morbidities (n/N, %)*: not reported *ASA 1 (n/N, %)*: not reported *ASA 2 (n/N, %)*: not reported *ASA 3 (n/N, %)*: not reported *ASA 4 (n/N, %)*: not reported *Number of participants randomised*: 75 *Number of participants receiving treatment*: 68 *Number of participants analysed*: 68 *Dropout rate*: 7/75 (9.3%) Aprotinin 500,000 KIU per hour (high dose) *Mean age (years) (mean)*: 63.4 *Ethnicity*: White: 72 (96%); Black: 2 (3%); Hispanic: 1 (1%) *Gender (males, females)*: 46/75 M (61%); 29/75 F (39%) *Mean operating time (minutes)*: 68 *Proportion of participants on anticoagulants prior to surgery (n/N, %)*: not reported *Incidence of preoperative anaemia (n/N, %)*: 0/77 (0%) *Co‐morbidities (n/N, %)*: not reported *ASA 1 (n/N, %)*: nR *ASA 2 (n/N, %)*: nR *ASA 3 (n/N, %)*: nR *ASA 4 (n/N, %)*: nR *Number of participants randomised*: 77 *Number of participants receiving treatment*: 75 *Number of participants analysed*: 75 *Dropout rate*: 2/77 (2.6%) Overall *Mean age (years) (mean)*: not reported *Ethnicity*: not reported *Gender (males, females)*: not reported *Mean operating time (minutes)*: not reported *Proportion of participants on anticoagulants prior to surgery (n/N, %)*: not reported *Incidence of preoperative anaemia (n/N, %)*: not reported *Co‐morbidities (n/N, %)*: not reported *ASA 1 (n/N, %)*: not reported *ASA 2 (n/N, %)*: not reported *ASA 3 (n/N, %)*: not reported *ASA 4 (n/N, %)*: not reported *Number of participants randomised*: not reported *Number of participants receiving treatment*: not reported *Number of participants analysed*: not reported *Dropout rate*: not reported **Inclusion criteria:** 1) patients at least 18 years old, 2) scheduled for an elective unilateral primary total hip replacement **Exclusion criteria:** 1) patients who had received aprotinin previously or who had a known or suspected allergy to aprotinin or warfarin were excluded, 2) pregnant women and those of child‐bearing age, 3) patients who had participated in an investigational drug study within the previous 30 days, 4) those who refused donor‐blood products, 5) patients with a history of deep vein thrombosis, pulmonary embolism, diabetes mellitus, 6) serum creatinine level above the upper limit of normal (1.5 mg/dL (133 μmol/L)), 7) impaired renal function (a serum creatinine level of greater than 2.5 mg/dL (221 μmol/L)), 8) failure of a major organ system, or an important medical illness (for example, decompensated congestive heart failure or notably elevated levels of hepatic enzymes) **If TKR, is tourniquet used:** not applicable **Indication for surgery:** not reported **Type of anaesthetic:** regional or general anaesthetic **Type of surgery:** primary THR	
Interventions	**Intervention characteristics** Placebo After transfer to the operating room and initiation of monitoring, all patients received a 1 mL test dose intravenously (taken from the loading dose) before or after the induction of anaesthesia. If there was no evidence of hypersensitivity to the drug after at least 10 minutes, the full loading dose was administered after the induction of anaesthesia, either regional or general. The contents of 4 x 50 mL vials of study medication were drawn up, injected into a sterile infusion bag and continuously infused at a rate of 50 mL per hour until the procedure was completed and the skin was closed. 73 patients received the placebo. Placebo, IV, intraop, ?loading/infusion or both Aprotinin 500,000 KIU (low dose) 76 patients received a loading dose of 500,000 KIU of aprotinin but no subsequent continuous infusion (low dose) Aprotinin, IV, 0.5 x 10^6 KIU intraop, loading dose only Aprotinin 250,000 KIU per hour (medium dose) 75 patients received a 1,000,000‐KIU loading dose followed by continuous infusion of 250,000 KIU per hour (medium dose) Aprotinin, IV, 1 x 10^6, intraop, loading dose AND aprotinin, IV, 0.25 x 10^6 KIU/hr infusion (from induction to skin closure) Aprotinin 500,000 KIU per hour (high dose) 77 patients received a loading dose of 2,000,000 KIU followed by continuous infusion of 500,000 KIU per hour (high dose) Aprotinin, IV, 2 x 10^6, intraop, loading dose AND aprotinin, IV, 0.5 x 10 ^6 KIU/hr infusion (from induction to skin closure)	
Outcomes	*Primary outcome:* Percentage of patients who required allogeneic or autologous blood or packed red blood cells through the 7th postoperative day *Secondary outcomes:* Number of units transfused Prevalence of deep venous thrombosis	
Notes	**Sponsorship source:** pharmaceutical **Country:** Canada and USA **Setting:** multi‐centre **Comments:** red cell transfusions ‐ unsure whether these include allogeneic or autologous units **Author's name:** JM Murkin **Institution:** University of Western Ontario **Email:** not reported **Address:** Department of Anaesthesia, London Health Sciences Center UC, University of Western Ontario, 339 Windermere Road, London, Ontario N6A 5A5, Canada **Native language of paper:** English **Reference type:** full text (1) **Trial registration number:** not applicable, but when checked, trial registration number not found **Was it translated for this review:** no	
***Risk of bias***		
**Bias**	**Authors' judgement**	**Support for judgement**
Random sequence generation (selection bias)	Unclear risk	Quote: "Three hundred and one patients were randomized into four groups and were stratified on the basis of whether or not preoperative autologous blood donations had been made." Judgement comment: insufficient information to permit judgement.
Allocation concealment (selection bias)	Low risk	Quote: "The study medication for intravenous infusion was supplied in sterile, sealed, fifty‐milliliter vials that appeared identical for all doses of aprotinin (Trasylol) and placebo. The labels on all vials were the same except for the consecutive patient number. The medication for each patient was supplied in a box that was affixed with a two‐part label. Part one of the label had a sealed, blinded area that concealed the identity and lot number of the contents. After transfer to the operating room and initiation of monitoring, all patients received a one‐ milliliter test dose intravenously (taken from the loading dose) before or after the induction of anesthesia. If there was no evidence of hypersensitivity to the drug after at least ten minutes, the full loading dose was administered after the induction of anesthesia, either regional or general." Judgement comment: sealed, consecutively numbered and in a box (opaque).
Blinding of participants and personnel (performance bias) Subjective outcomes	High risk	Quote: not applicable Judgement comment: treatment arms have different treatment regimens reported in the study, which would make blinding impossible.
Blinding of participants and personnel (performance bias) Objective outcomes	Low risk	Quote: not applicable Judgement comment: objective outcome for personnel, so low risk of bias regardless of quality of blinding.
Blinding of outcome assessment (detection bias) Subjective outcomes	Unclear risk	Quote: not applicable Judgement comment: insufficient information to permit judgement.
Blinding of outcome assessment (detection bias) Objective outcomes	Low risk	Quote: not applicable Judgement comment: objective outcome for outcome assessor, so low risk of bias regardless of quality of blinding.
Incomplete outcome data (attrition bias) All outcomes	Unclear risk	Quote: not applicable Judgement comment: reasons for missing data not explained.
Selective reporting (reporting bias)	High risk	Quote: not applicable Judgement comment: one or more outcomes of interest reported incompletely such that they cannot be entered into the meta‐analysis. No protocol to check pre‐specified outcomes reported.
Other bias	High risk	Quote: not applicable Judgement comment: analysed and reported adverse events collectively with patients who received the full treatment and those who received only a test dose (1 mL) of the treatment. In addition, other interventions were used (desmopressin and/or EACA) if indicated and not reported who received these interventions.

**Table:** NCT02922582

***Study characteristics***		
Methods	**Study design:** RCT **Intention‐to‐treat analysis:** yes **Duration of study:** 12 months (participants were recruited between 28 October 2016 and 12 October 2017 at 4 US sites. The study was terminated on 27 November 2017 due to low enrolment). Follow‐up length not reported. **Power calculation reached:** no **Transfusion strategy:** transfusion requirements consistent with each institution’s transfusion policy **Was the trial stopped early:** yes ‐ slow to recruit	
Participants	**Baseline characteristics** DepoTXA 400 mg *Age (years) (mean SD)*: 65 (7.12) *Ethnicity*: Hispanic or Latino 0/4, 0%; not Latino/Hispanic 4/4, 100%; unknown or not reported 0/4, 0% *Gender (males, females)*: 1/4 M (25%); 3/4 F (75%) *Length of surgery (minutes) (mean, SD)*: not reported *Proportion of participants on anticoagulants prior to surgery (n/N, %)*: not reported *Incidence of preoperative anaemia (n/N, %)*: not reported *Co‐morbidities (n/N, %)*: not reported *ASA 1 (n/N, %)*: not reported *ASA 2 (n/N, %)*: not reported *ASA 3 (n/N, %)*: not reported *ASA 4 (n/N, %)*: not reported *Number of participants randomised*: 4 *Number of participants receiving treatment*: 4 *Number of participants analysed*: 4 *Dropout rate*: 0/4, 0% DepoTXA 800 mg *Age (years) (mean SD)*: 66 (1.73) *Ethnicity*: Hispanic or Latino 0/3, 0%; not Latino/Hispanic 3/3, 100%; unknown or not reported 0/3, 0% *Gender (males, females)*: 1/3 M (33.3%); 2/3 F (66.7%) *Length of surgery (minutes)*: not reported *Proportion of participants on anticoagulants prior to surgery (n/N, %)*: not reported *Incidence of preoperative anaemia (n/N, %)*: not reported *Co‐morbidities (n/N, %)*: not reported *ASA 1 (n/N, %)*: not reported *ASA 2 (n/N, %)*: not reported *ASA 3 (n/N, %)*: not reported *ASA 4 (n/N, %)*: not reported *Number of participants randomised*: 4 *Number of participants receiving treatment*: 3 *Number of participants analysed*: 3 *Dropout rate*: 0/4, 0% DepoTXA 1200 mg *Age (years) (mean SD)*: 59.3 (5.38) *Ethnicity*: Hispanic or Latino 0/4, 0%; not Latino/Hispanic 4/4, 100%; unknown or not reported 0/4, 0% *Gender (males, females)*: 2/4 M (50%); 2/4 F (50%) *Length of surgery (minutes)*: not reported *Proportion of participants on anticoagulants prior to surgery (n/N, %)*: not reported *Incidence of preoperative anaemia (n/N, %)*: not reported *Co‐morbidities (n/N, %)*: not reported *ASA 1 (n/N, %)*: not reported *ASA 2 (n/N, %)*: not reported *ASA 3 (n/N, %)*: not reported *ASA 4 (n/N, %)*: not reported *Number of participants randomised*: 4 *Number of participants receiving treatment*: 4 *Number of participants analysed*: 4 *Dropout rate*: 0/4, 0% TXA, IV *Age (years) (mean SD)*: 58.0 (6.16) *Ethnicity*: Hispanic or Latino 0/4, 0%; not Latino/Hispanic 4/4, 100%; unknown or not reported 0/4, 0% *Gender (males, females)*: 2/4 M (50%); 2/4 F (50%) *Length of surgery (minutes)*: not reported *Proportion of participants on anticoagulants prior to surgery (n/N, %)*: not reported *Incidence of preoperative anaemia (n/N, %)*: not reported *Co‐morbidities (n/N, %)*: not reported *ASA 1 (n/N, %)*: not reported *ASA 2 (n/N, %)*: not reported *ASA 3 (n/N, %)*: not reported *ASA 4 (n/N, %)*: not reported *Number of participants randomised*: 4 *Number of participants receiving treatment*: 4 *Number of participants analysed*: 4 *Dropout rate*: 0/4, 0% Overall *Age (years) (mean SD)*: 61.8 (6.19) *Ethnicity*: Hispanic or Latino 0/15, 0%; not Latino/Hispanic 15/15, 100%; unknown or not reported 0/4, 0% *Gender (males, females)*: 6/15 M (40%); 9/15 F (60%) *Length of surgery (minutes)*: not reported *Proportion of participants on anticoagulants prior to surgery (n/N, %)*: not reported *Incidence of preoperative anaemia (n/N, %)*: not reported *Co‐morbidities (n/N, %)*: not reported *ASA 1 (n/N, %)*: not reported *ASA 2 (n/N, %)*: not reported *ASA 3 (n/N, %)*: not reported *ASA 4 (n/N, %)*: not reported *Number of participants randomised*: 16 *Number of participants receiving treatment*: 15 *Number of participants analysed*: 15 *Dropout rate*: 0/16, 0% **Inclusion criteria:** male or female, ≥ 18 years of age at screening. Scheduled to undergo elective unilateral open TKA under general, spinal, or regional anaesthesia. ASA physical status 1, 2 or 3. Female participants must be surgically sterile; or at least 2 years postmenopausal; or have a monogamous partner who is surgically sterile; or practising double‐barrier contraception; or practising abstinence (must agree to use double‐barrier contraception in the event of sexual activity); or using an insertable, injectable or transdermal contraceptive approved by the FDA for greater than 2 months prior to screening and commit to the use of an acceptable form of birth control for the duration of the study and for 30 days after completion of the study. Able to provide informed consent, adhere to the study visit schedule and complete all study assessments. **Exclusion criteria:** currently pregnant, nursing or planning to become pregnant during the study or within 1 month after study drug administration. Planned concurrent surgical procedure (e.g. bilateral TKA). Prior open knee surgery on ipsilateral knee. Prior arthroscopy is permitted. Subjects taking a medication with a known procoagulant effect (e.g. combination hormonal contraceptives, Factor IX complex concentrates or anti‐inhibitor coagulant concentrates, or all‐trans retinoic acid). Contraindication or hypersensitivity to TXA. History of thrombosis or prior venous thromboembolism (VTE). Known coagulopathy or active intravascular clotting. Prior myocardial infarction. Prior cardiovascular accident (stroke) or subarachnoid haemorrhage. History of epilepsy. Presence of an intravascular stent. History of impaired kidney function, chronic respiratory disease, rheumatoid arthritis, coagulopathy or loss of sensation in extremities. Renal insufficiency (serum creatinine level > 2 mg/dL). Anaemia (Hb level < 10 g/dL). Uncontrolled anxiety, psychiatric or neurological disorder that might interfere with study assessments. Acquired defective colour vision. Malignancy in the last 2 years, with the exception of non‐metastatic basal cell or squamous cell carcinoma of the skin or localised carcinoma in situ of the cervix. Suspected or known history of drug or alcohol abuse within the previous year. Body weight < 50 kg (110 pounds) or a body mass index > 44 kg/m^2^. Administration of an investigational drug within 30 days or 5 elimination half‐lives of such investigational drug, whichever is longer, prior to study drug administration, or planned administration of another investigational product or procedure during the participation in this study. **If TKR, is tourniquet used:** yes **Indication for surgery:** unclear **Type of anaesthetic:** general, spinal or regional anesthesia **Type of surgery:** unilateral open TKA	
Interventions	**Intervention characteristics** DepoTXA 400 mg Single injection of DepoTXA 400 mg into the joint space via catheter prior to capsular closure TXA, intracapsular, 400 mg, intraop DepoTXA 800 mg Single injection of DepoTXA 800 mg into the joint space via catheter prior to capsular closure TXA, intracapsular, 800 mg, intraop DepoTXA 1200 mg Single injection of DepoTXA 1200 mg into the joint space via catheter prior to capsular closure TXA, intracapsular, 1200mg, intraop TXA, IV 1g of IV TXA at the end of surgery TXA, IV, 1 g, intraop	
Outcomes	*Primary outcomes:* Area under the plasma concentration‐versus‐time curve from time 0 extrapolated to infinity after drug administration Area under the plasma concentration‐versus‐time curve from time 0 to the last collection time after drug administration Maximum plasma concentration Time to maximum plasma concentration Apparent terminal elimination rate constant Apparent terminal elimination half‐life *Secondary outcomes:* Summary of neurological assessments Incidence of reoperation due to haematoma or wound dehiscence Incidence of transfusion Number of participants with 90˚ passive and active knee flexion Time to Complete Timed Up‐and‐Go (TUG) Test Change in knee and thigh measurements Area under the curve (AUC) of NRS	
Notes	**Sponsorship source:** pharmaceutical (Pacira Pharmaceuticals) **Country:** USA **Setting:** multi‐centre **Comments:** 1) all information extracted here has come solely from the trial's NCT page, 2) reoperation data given but not for bleeding (which is what we want) but for haematoma and wound dehiscence **Author's name:** Dr Hassan Danesi (Study Director) **Institution:** Pacira Pharmaceuticals, Inc., 5 Sylvan Way, Parsippany, NJ 07054, (973) 254‐3560 **Email:** jim.jones@pacira.com **Address:** Pacira Pharmaceuticals, Inc., 5 Sylvan Way, Parsippany, NJ 07054, (973) 254‐3560 **Native language of paper:** trial registration only ‐ English **Reference type:** trial registration (1) **Trial registration number:** NCT02922582 **Was it translated for this review:** no	
***Risk of bias***		
**Bias**	**Authors' judgement**	**Support for judgement**
Random sequence generation (selection bias)	Low risk	Quote: "The randomization code will be generated by a centralized randomization system" Judgement comment: none
Allocation concealment (selection bias)	Unclear risk	Quote: not applicable Judgement comment: insufficient information to permit judgement.
Blinding of participants and personnel (performance bias) Subjective outcomes	High risk	Quote: not applicable Judgement comment: "single blinded study", personnel not blinded.
Blinding of participants and personnel (performance bias) Objective outcomes	Low risk	Quote: not applicable Judgement comment: objective outcome for personnel, so low risk of bias regardless of quality of blinding.
Blinding of outcome assessment (detection bias) Subjective outcomes	High risk	Quote: not applicable Judgement comment: 'single blinded', participant only blinded
Blinding of outcome assessment (detection bias) Objective outcomes	Low risk	Quote: not applicable Judgement comment: objective outcome for outcome assessor, so low risk of bias regardless of quality of blinding.
Incomplete outcome data (attrition bias) All outcomes	Low risk	Quote: not applicable Judgement comment: no missing data from participants included.
Selective reporting (reporting bias)	Low risk	Quote: not applicable Judgement comment: all outcomes reported.
Other bias	High risk	Quote: not applicable Judgement comment: study terminated as slow to recruit.

**Table:** Niskanen 2005

***Study characteristics***		
Methods	**Study design:** RCT **Intention‐to‐treat analysis:** per protocol (mentions 'on‐treatment' analysis being done) **Duration of study:** unclear **Power calculation reached:** yes **Transfusion strategy:** even though they had an indicative 0.28 to 0.30 level of haematocrit for blood transfusions, it was the clinical situation that determined the need for blood transfusions **Was the trial stopped early:** no	
Participants	**Baseline characteristics** Placebo *Age (years) (mean SD)*: 65 (8.2) *Ethnicity*: not reported *Gender (males, females)*: 7/20 M (35%); 13/20 (65%) *Length of surgery (minutes) (mean SD)*: 96 (12) *Proportion of participants on anticoagulants prior to surgery (n/N, %)*: 0/20 (0%) *Incidence of preoperative anaemia (n/N, %)*: not reported *Co‐morbidities (n/N, %)*: not reported *ASA 1 (n/N, %)*: not reported *ASA 2 (n/N, %)*: not reported *ASA 3 (n/N, %)*: not reported *ASA 4 (n/N, %)*: not reported *Number of participants randomised*: 20 *Number of participants receiving treatment*: 20 *Number of participants analysed*: 20 *Dropout rate*: 0/20 (0%) TXA *Age (years) (mean SD)*: 66 (9.1) *Ethnicity*: not reported *Gender (males, females)*: 6/19 M (32%); 13/19 F (68%) *Length of surgery (minutes) (mean SD)*: 93 (12) *Proportion of participants on anticoagulants prior to surgery (n/N, %)*: 0/19 (0%) *Incidence of preoperative anaemia (n/N, %)*: not reported *Co‐morbidities (n/N, %)*: not reported *ASA 1 (n/N, %)*: not reported *ASA 2 (n/N, %)*: not reported *ASA 3 (n/N, %)*: not reported *ASA 4 (n/N, %)*: not reported *Number of participants randomised*: 20 *Number of participants receiving treatment*: 20 *Number of participants analysed*: 19 *Dropout rate*: 1/19 (5.3%) Overall *Age (years) (mean SD)*: not reported *Ethnicity*: not reported *Gender (males, females)*: not reported *Length of surgery (minutes) (mean SD)*: not reported *Proportion of participants on anticoagulants prior to surgery (n/N, %)*: not reported *Incidence of preoperative anaemia (n/N, %)*: not reported *Co‐morbidities (n/N, %)*: not reported *ASA 1 (n/N, %)*: not reported *ASA 2 (n/N, %)*: not reported *ASA 3 (n/N, %)*: not reported *ASA 4 (n/N, %)*: not reported *Number of participants randomised*: not reported *Number of participants receiving treatment*: not reported *Number of participants analysed*: not reported *Dropout rate*: not reported **Inclusion criteria:** 1) patients scheduled for a cemented hip arthroplasty for osteoarthritis **Exclusion criteria:** 1) patients with rheumatoid arthritis and osteonecrosis, 2) patients with known coagulation disturbances including thromboembolic events, 3) patients using warfarin‐related preparations, 4) patients with allergy to tranexamic acid, 5) patients with signs of renal insufficiency **If TKR, is tourniquet used:** not applicable **Indication for surgery:** osteoarthritis **Type of anaesthetic:** spinal (or general) (1 patient had general) **Type of surgery:** primary THR	
Interventions	**Intervention characteristics** Placebo Half of the patients received a corresponding dose of saline. The first injection was given intravenously over 5 to 10 min, immediately before the operation. The next 2 doses of tranexamic acid or placebo were given 8 h and 16 h after the first injection. Placebo, IV, preop (5 to 10 mins prior) AND placebo, IV, postop 8 + 16 hours postop TXA Half of the patients received 3 doses of tranexamic acid (100 mg/mL, Cyklokapron, Pharmacia, later Pfizer) 10 mg/kg of body weight mixed in 100 mL saline. Half of the patients received a corresponding dose of saline. The first injection was given intravenously over 5 to 10 min, immediately before the operation. The next 2 doses of tranexamic acid or placebo were given 8 h and 16 h after the first injection. TXA, IV, 10 mg/kg, preop (5 to 10mins prior) AND TXA, IV, 10 mg/kg postop 8 + 16 hours postop	
Outcomes	*Primary outcomes*: Blood loss during the operation Amount of drainage after the operation *Secondary outcomes*: Amount of transfused units of red cells Wound leakage postoperatively Swelling Ecchymoses of the thigh Haematocrit Possible complications	
Notes	**Sponsorship source:** pharmaceutical **Country:** Finland **Setting:** single‐centre **Comments:** none **Author's name:** Raimo O Niskanen **Institution:** Päijät‐Häme Central Hospital Lahti **Email:** raimo.niskanen@fimnet.fi **Address:** Department of Orthopedics, Päijät‐Häme Central Hospital Lahti, Rheumatism Foundation Hospital, Heinola, Finland **Native language of paper:** English **Reference type:** full text (1) **Trial registration number:** not applicable, but when checked, trial registration number not found **Was it translated for this review:** no	
***Risk of bias***		
**Bias**	**Authors' judgement**	**Support for judgement**
Random sequence generation (selection bias)	Unclear risk	Quote: "The patients were randomized into two groups by an envelope method in a double‐blind manner." Judgement comment: insufficient information to permit judgement.
Allocation concealment (selection bias)	Unclear risk	Quote: "The randomization and preparation of the drug were done in the absence of other personnel by 2 anesthesia nurses not engaged in the study. The code was broken after the last patient had been treated." Judgement comment: insufficient information to permit judgement.
Blinding of participants and personnel (performance bias) Subjective outcomes	Unclear risk	Quote: "The randomization and preparation of the drug were done in the absence of other personnel by 2 anesthesia nurses not engaged in the study." Judgement comment: insufficient information to permit judgement.
Blinding of participants and personnel (performance bias) Objective outcomes	Low risk	Quote: not applicable Judgement comment: objective outcome for personnel, so low risk of bias regardless of quality of blinding.
Blinding of outcome assessment (detection bias) Subjective outcomes	Unclear risk	Quote: not applicable Judgement comment: no description of outcome assessor blinding.
Blinding of outcome assessment (detection bias) Objective outcomes	Low risk	Quote: not applicable Judgement comment: objective outcome for outcome assessor, so low risk of bias regardless of quality of blinding.
Incomplete outcome data (attrition bias) All outcomes	Low risk	Quote: not applicable Judgement comment: no missing data.
Selective reporting (reporting bias)	High risk	Quote: not applicable Judgement comment: one of the outcomes of interest in the review are reported incompletely so that they cannot be entered in a meta‐analysis.
Other bias	High risk	Quote: not applicable Judgement comment: on treatment analysis study.

**Table:** North 2016

***Study characteristics***		
Methods	**Study design:** RCT **Intention‐to‐treat analysis:** yes **Duration of study (months):** 17 **Power calculation reached:** no **Transfusion strategy:** patients were transfused at Hb < 7 g/dL in all cases and in cases of symptomatic anaemia when Hb < 8 g/dL **Was the trial stopped early:** no	
Participants	**Baseline characteristics** TXA, IV *Age (years) (mean, SD)*: 64.1 ± 12.0 *Ethnicity*: not reported *Gender (male, female)*: 38/70 M (54%); 32/70 F (46%) *Length of surgery*: not reported *Proportion of participants on anticoagulants prior to surgery (n/N, %)*: not reported *Incidence of preoperative anaemia (n/N, %)*: not reported *Co‐morbidities (n/N, %)*: not reported *ASA 1 (n, %)*: 0/70, (0%) *ASA 2 (n, %)*: 16/70, (23%) *ASA 3 (n, %)*: 52/70, (75%) *ASA 4 (n, %)*: 0/70, (0%) *Number of participants randomised*: 70 *Number of participants receiving treatment*: 70 *Number of participants analysed*: 70 *Dropout rate*: 0 TXA, IA *Age (years) (mean, SD)*: 65.7 ± 10.6 *Ethnicity*: not reported *Gender (male, female)*: 39/69 M (57%); 30/69 F (43%) *Length of surgery*: not reported *Proportion of participants on anticoagulants prior to surgery (n/N, %)*: not reported *Incidence of preoperative anaemia (n/N, %)*: not reported *Co‐morbidities (n/N, %)*: not reported *ASA 1 (n, %)*: 1/69, 1% *ASA 2 (n, %)*: 20/69, 29% *ASA 3 (n, %)*: 47/69, 68% *ASA 4 (n, %)*: 1/69, 1% *Number of participants randomised*: 69 *Number of participants receiving treatment*: 69 *Number of participants analysed*: 69 *Dropout rate*: 0 Overall *Age (years) (mean, SD)*: 64.9 ± 11.3 *Ethnicity*: not reported *Gender (male, female) (n, %)*: 77 M (55%); 62 F (45%) *Length of surgery*: not reported *Proportion of participants on anticoagulants prior to surgery (n/N, %)*: not reported *Incidence of preoperative anaemia (n/N, %)*: not reported *Co‐morbidities (n/N, %)*: not reported *ASA 1 (n, %)*: 1/139, (1%) *ASA 2 (n, %)*: 36/139, (26%) *ASA 3 (n, %)*: 99/139, (72%) *ASA 4 (n, %)*: 1/139, (1%) *Number of participants randomised*: 139 *Number of participants receiving treatment*: 139 *Number of participants analysed*: 139 *Dropout rate*: 0 **Inclusion criteria:** all patients scheduled for primary, unilateral THA were flagged for study inclusion. **Exclusion criteria:** 1) cemented femoral or acetabular component, 2) current medical management of DVT or PE, 3) previous embolic stroke or SAH, 4) active liver disease with abnormal coagulation profile, 5) alteration to colour vision, 6) epilepsy, 7) previous surgery on the planned operative hip, 8) current treatment with OCP or HRT **Type of surgery:** primary THR **Indication for surgery:** any **If TKR, is tourniquet used:** not applicable **Type of anaesthetic:** both (evenly distributed)	
Interventions	**Intervention characteristics** TXA, IV The patient was randomised to receive 2.0 g of either topical or IV TXA in 100 mL of 0.9% normal saline solution. Two solutions labelled “IV” and “Topical” accompanied the patient to the operating room (one solution contained the 2.0 g of TXA and the other contained saline placebo). The IV solution was administered by anaesthesia in two 50 mL doses, each over 20 minutes using a pump to ensure the correct volume was administered. One administration was started 10 minutes before incision, and the second during the fascial closure. The topical solution was applied to the wound by the surgical team after component placement and allowed to sit undisturbed for 5 minutes at which point it was removed by suction. TXA, IV, 2 g, preop TXA, IA The patient was randomised to receive 2.0 g of either topical or IV TXA in 100 mL of 0.9% normal saline solution. Two solutions labelled “IV” and “Topical” accompanied the patient to the operating room (one solution contained the 2.0 g of TXA and the other contained saline placebo). The IV solution was administered by anaesthesia in two 50 mL doses, each over 20 minutes using a pump to ensure the correct volume was administered. One administration was started 10 minutes before incision, and the second during the fascial closure. The topical solution was applied to the wound by the surgical team after component placement and allowed to sit undisturbed for 5 minutes at which point it was removed by suction. TXA, IA, 2 g, intraop	
Outcomes	*Primary outcomes:* Assessment of blood and haemoglobin loss Transfusion rates *Secondary outcomes:* Transfusion cost analysis relative to historic controls Assessment of thromboembolic events	
Notes	**Sponsorship source:** non‐pharmaceutical **Country:** USA **Setting:** single‐centre **Comments:** none **Author's name:** W North **Institution:** Henry Ford Hospital **Email:** not reported **Address:** not reported **Native language of paper:** English **Reference type:** full text (1) **Trial registration number:** NCT01683955 **Was it translated for this review:** no	
***Risk of bias***		
**Bias**	**Authors' judgement**	**Support for judgement**
Random sequence generation (selection bias)	Unclear risk	Quote: not applicable Judgement comment: the randomisation algorithm was created by a blinded biostatistician, and patients were allocated in blocks of 4 by a blinded research pharmacist.
Allocation concealment (selection bias)	Low risk	Quote: not applicable Judgement comment: patients were allocated in blocks of 4 by a blinded research pharmacist. The randomisation was not broken until study completion.
Blinding of participants and personnel (performance bias) Subjective outcomes	Low risk	Quote: not applicable Judgement comment: blinding of participants and key study personnel ensured and unlikely that blinding could have been broken.
Blinding of participants and personnel (performance bias) Objective outcomes	Low risk	Quote: not applicable Judgement comment: randomisation was not broken until study completion.
Blinding of outcome assessment (detection bias) Subjective outcomes	Low risk	Quote: "The randomization algorithm was created by a blinded biostatistician, and patients were allocated in blocks of 4 by a blinded research pharmacist. The randomization was not broken until study completion" Judgement comment: although not explicitly stated, study personnel were all blinded.
Blinding of outcome assessment (detection bias) Objective outcomes	Low risk	Quote: not applicable Judgement comment: low risk of bias as outcome is mortality.
Incomplete outcome data (attrition bias) All outcomes	Low risk	Quote: not applicable Judgement comment: no missing data.
Selective reporting (reporting bias)	Unclear risk	Quote: not applicable Judgement comment: not reported time frame for outcomes (appears different to protocol).
Other bias	Low risk	Quote: not applicable Judgement comment: the study appears to be free of other sources of bias.

**Table:** Orpen 2006

***Study characteristics***		
Methods	**Study design:** RCT **Intention‐to‐treat analysis:** not reported **Duration of study:** not reported **Power calculation reached:** no **Transfusion strategy:** blood transfusion was given to patients whose postoperative haemoglobin (Hb) was less than 9 g/dL, with 1 unit given for each g/dL (or part thereof) that the Hb was below 10 g/dL, with a minimum of 2 units being administered in the event of a transfusion being indicated. **Was the trial stopped early:** no	
Participants	**Baseline characteristics** Placebo *Age (years) (mean range)*: 69 (63 to 74) *Ethnicity*: not reported *Gender (males, females)*: 3/14 M (21%); 11/14 F (79%) *Length of surgery (minutes)*: not reported *Proportion of participants on anticoagulants prior to surgery (n/N, %)*: not reported *Incidence of preoperative anaemia (n/N, %)*: not reported *Co‐morbidities (n/N, %)*: not reported *ASA 1 (n/N, %)*: not reported *ASA 2 (n/N, %)*: not reported *ASA 3 (n/N, %)*: not reported *ASA 4 (n/N, %)*: not reported *Number of participants randomised*: 15 *Number of participants receiving treatment*: 15 *Number of participants analysed*: 14 *Dropout rate*: 1/15 (6.7%) TXA *Age (years) (mean range)*: 73 (70 to 78) *Ethnicity*: not reported *Gender (males, females)*: 8/15 M (53%); 7/15 F (47%) *Length of surgery (minutes)*: not reported *Proportion of participants on anticoagulants prior to surgery (n/N, %)*: not reported *Incidence of preoperative anaemia (n/N, %)*: not reported *Co‐morbidities (n/N, %)*: not reported *ASA 1 (n/N, %)*: not reported *ASA 2 (n/N, %)*: not reported *ASA 3 (n/N, %)*: not reported *ASA 4 (n/N, %)*: not reported *Number of participants randomised*: 15 *Number of participants receiving treatment*: 15 *Number of participants analysed*: 15 *Dropout rate*: 0/15 (0%) Overall *Age (years) (mean range)*: not reported *Ethnicity*: not reported *Gender (males, females)*: not reported *Length of surgery (minutes)*: not reported *Proportion of participants on anticoagulants prior to surgery (n/N, %)*: not reported *Incidence of preoperative anaemia (n/N, %)*: not reported *Co‐morbidities (n/N, %)*: not reported *ASA 1 (n/N, %)*: not reported *ASA 2 (n/N, %)*: not reported *ASA 3 (n/N, %)*: not reported *ASA 4 (n/N, %)*: not reported *Number of participants randomised*: 30 *Number of participants receiving treatment*: 30 *Number of participants analysed*: 29 *Dropout rate*: 1/30 (3.3%) **Inclusion criteria:** all patients fit for a total knee replacement were considered eligible **Exclusion criteria:** 1) history of thromboembolic disease, 2) cerebrovascular disease, 3) recent myocardial infarction or unstable angina, 4) a coagulation defect, 5) those with an allergy to TA, 6) not fit to undergo surgery under general anaesthetic **If TKR, is tourniquet used:** yes **Indication for surgery:** osteoarthritis, inflammatory arthritis **Type of anaesthetic:** general **Type of surgery:** primary, unilateral TKR	
Interventions	**Intervention characteristics** Placebo 15 mg/kg of TA, or an equivalent volume of normal saline, given intravenously at the time that cement mixing commenced. This time is reasonably constant in relation to the tourniquet release, as this is governed by the time taken for the cement to cure (10 to 12 min). Placebo saline mL, IV, intraop, single TXA 15 mg/kg of TA, or an equivalent volume of normal saline, given intravenously at the time that cement mixing commenced. This time is reasonably constant in relation to the tourniquet release, as this is governed by the time taken for the cement to cure (10 to 12 min). TXA, IV, 15 mg/kg, intraop, single	
Outcomes	*Primary outcomes:* Reductions in blood loss Clinical and sub‐clinical DVT *Secondary outcome:* Not reported	
Notes	**Sponsorship source:** not reported **Country:** UK **Setting:** single‐centre **Comments:** none **Author's name:** EJP Crawfurd **Institution:** Northampton General Hospital **Email:** edward.crawfurd@ngh.nhs.uk **Address:** Department of Orthopaedics and Department of Anaesthetics, Northampton General Hospital, Northampton NN1 5BD, UK **Native language of paper:** English **Reference type:** full text (1) **Trial registration number:** not applicable, but when checked, trial registration number not found **Was it translated for this review:** no	
***Risk of bias***		
**Bias**	**Authors' judgement**	**Support for judgement**
Random sequence generation (selection bias)	Unclear risk	Quote: "A pharmacist not involved with the study carried out randomisation in the pharmacy by a sealed envelope method" Judgement comment: insufficient information to permit judgement.
Allocation concealment (selection bias)	Unclear risk	Quote: "sealed envelope method" Judgement comment: no mention of whether the envelopes were sequentially numbered or opaque.
Blinding of participants and personnel (performance bias) Subjective outcomes	Low risk	Quote: "A pharmacist not involved with the study carried out randomisation in the pharmacy by a sealed envelope method and prepared the contents of the administered solution. The operating team was blinded to the contents of the administered solution for every patient although allowance was made for the code to be broken should an adverse drug reaction occur." Judgement comment: blinding of key personnel and participants ensured, unlikely blinding could have been broken.
Blinding of participants and personnel (performance bias) Objective outcomes	Low risk	Quote: not applicable Judgement comment: objective outcome for personnel, so low risk of bias regardless of quality of blinding.
Blinding of outcome assessment (detection bias) Subjective outcomes	Low risk	Quote: "A pharmacist not involved with the study carried out randomisation in the pharmacy by a sealed envelope method and prepared the contents of the administered solution. The operating team was blinded to the contents of the administered solution for every patient although allowance was made for the code to be broken should an adverse drug reaction occur." Judgement comment: a pharmacist not involved with the study carried out randomisation and prepared the contents of the administered solution.
Blinding of outcome assessment (detection bias) Objective outcomes	Low risk	Quote: not applicable Judgement comment: objective outcome for outcome assessor, so low risk of bias regardless of quality of blinding.
Incomplete outcome data (attrition bias) All outcomes	Low risk	Quote: "1 patient in the placebo group was excluded from the original total of 15 as the drains had fallen out in the immediate postoperative period thereby making data collection impossible" Judgement comment: reason for missing outcome data explained due to significant protocol deviation.
Selective reporting (reporting bias)	Unclear risk	Quote: not applicable Judgement comment: the study protocol is not available and therefore unable to assess whether all the pre‐specified outcomes of interest in the review were reported.
Other bias	Unclear risk	Quote: not applicable Judgement comment: insufficient information to permit judgement.

**Table:** Painter 2018

***Study characteristics***		
Methods	**Study design:** RCT **Intention‐to‐treat analysis:** yes **Duration of study:** 37 months + 6 months = 43 months **Power calculation reached:** yes **Transfusion strategy:** no red blood cell transfusion trigger was imposed, but clinicians were encouraged to adopt a haemoglobin (Hb) concentration threshold of 80 g/L before considering transfusion. **Was the trial stopped early:** no	
Participants	**Baseline characteristics** Placebo *Age (years) (mean SD)*: 68 (8.6) *Ethnicity*: not reported *Gender (males, females)*: 37/69 M (54%); 32/69 F (46%) *Length of surgery (minutes)*: not reported *Proportion of participants on anticoagulants prior to surgery (n/N, %)*: 8/69 (11.6%) *Incidence of preoperative anaemia (n/N, %)*: not reported *Co‐morbidities (n/N, %)*: hypertension 47/69 (68.1%), diabetes 17/69 (24.6%), hypercholesterolaemia 25/69 (36.2%), prior myocardial infarction 7/69 (10.1%) *ASA 1 (n/N, %)*: not reported *ASA 2 (n/N, %)*: not reported *ASA 3 (n/N, %)*: not reported *ASA 4 (n/N, %)*: not reported *Number of participants randomised*: 69 *Number of participants receiving treatment*: 69 *Number of participants analysed*: 69 *Dropout rate*: 0/69, (0)% TXA *Age (years) (mean SD)*: 69 (9.1) *Ethnicity*: not reported *Gender (males, females)*: 28/71 M (39%); 43/71 F (61%) *Length of surgery (minutes)*: not reported *Proportion of participants on anticoagulants prior to surgery (n/N, %)*: 6/71 (8.5%) *Incidence of preoperative anaemia (n/N, %)*: not reported *Co‐morbidities (n/N, %)*: hypertension 36/71 (50.7%), diabetes 11/71 (15.5%), hypercholesterolaemia 23/71 (32.4%), prior myocardial infarction 6/71 (8.5%) *ASA 1 (n/N, %)*: not reported *ASA 2 (n/N, %)*: not reported *ASA 3 (n/N, %)*: not reported *ASA 4 (n/N, %)*: not reported *Number of participants randomised*: 71 *Number of participants receiving treatment*: 71 *Number of participants analysed*: 71 *Dropout rate*: 0/71, (0)% Overall *Age (years) (mean SD)*: not reported *Ethnicity*: not reported *Gender (males, females)*: not reported *Length of surgery (minutes)*: not reported *Proportion of participants on anticoagulants prior to surgery (n/N, %)*: not reported *Incidence of preoperative anaemia (n/N, %)*: not reported *Co‐morbidities (n/N, %)*: not reported *ASA 1 (n/N, %)*: not reported *ASA 2 (n/N, %)*: not reported *ASA 3 (n/N, %)*: not reported *ASA 4 (n/N, %)*: not reported *Number of participants randomised*: 140 *Number of participants receiving treatment*: 140 *Number of participants analysed*: 140 *Dropout rate*: 0 **Inclusion criteria:** 1) aged 45 years or older 2) undergoing primary or revision hip or knee joint replacement **Exclusion criteria:** 1) contraindications to the administration of TXA including active thromboembolic disease or a history of venous (spontaneous or provoked) or arterial thromboembolic disease **If TKR, is tourniquet used:** yes **Indication for surgery:** not reported **Type of anaesthetic:** mixed (at discretion of treating clinician) **Type of surgery:** mixed ‐ primary and revision THR and TKR	
Interventions	**Intervention characteristics** Placebo No information given about placebo, except that saline was used Placebo, IV, intraop (at incision for THR, at tourniquet deflation for TKR) AND placebo, IV, 8 + 16 hours postop TXA TXA was prepared and administered per the product information: 15mg/kg at skin incision (hip joint replacement) or at tourniquet deflation (knee joint replacement) with repeat dosing at 8 and 16 hours post‐initial dosing. TXA, IV, 15 mg/kg, intraop (at incision for THR, at tourniquet deflation for TKR) AND TXA, IV, 15 mg/kg, 8 + 16 hours postop	
Outcomes	*Primary outcomes:* Proportion of patients receiving allogenic blood transfusion Feasibility of extending the trial methodology to a large trial of TXA and placebo in this population *Secondary outcomes:* Change in Hb concentration and PCV Incidence of adverse clinical events (mortality, myocardial infarction, MINS, non‐fatal cardiac arrest, transient ischaemic attack/stroke, deep venous thrombosis, pulmonary embolism, acute kidney injury, atrial fibrillation, infection/sepsis, bleeding, congestive heart failure, peripheral arterial thrombosis or coronary intervention) Incidence of surgical complications (need for reoperation, haemarthrosis, haematoma, infection of wound or joint or wound ooze) Length of hospital stay Change in quality of life (EQ‐5D) Quality of recovery (QoR‐15) Osteoarthritis severity (Western Ontario McMaster Osteoarthritis (WOMAC®) Index, under licence) Joint specific questionnaires (Oxford Hip or Knee score)	
Notes	**Sponsorship source:** non‐pharmaceutical **Country:** Australia **Setting:** multi‐centre **Comments:** none **Author's name:** TW Painter **Institution:** University of Adelaide **Email:** thomas.painter@adelaide.edu.au **Address:** Senior Staff Anaesthetist, Department of Anaesthesia, Royal Adelaide Hospital; Clinical Senior Lecturer, Discipline of Acute Care Medicine, Adelaide Medical School, University of Adelaide; Adelaide, South Australia **Native language of paper:** English **Reference type:** full text (1), trial registration (1) **Trial registration number:** ACTRN12613000323729 **Was it translated for this review:** no	
***Risk of bias***		
**Bias**	**Authors' judgement**	**Support for judgement**
Random sequence generation (selection bias)	Low risk	Quote: "Patients were randomly assigned to receive IV TA or a placebo (normal saline) by a computer‐generated randomisation sequence (random permuted blocks of four stratified by hospital, knee or hip joint replacement and primary or revision surgery) placed in consecutively numbered sealed..." Judgement comment: computer‐generated randomisation.
Allocation concealment (selection bias)	Low risk	Quote: "... placed in consecutively numbered sealed and opaque envelopes." Judgement comment: consecutively numbers sealed and opaque envelopes.
Blinding of participants and personnel (performance bias) Subjective outcomes	Low risk	Quote: "The study drug was prepared by an unblinded investigator, who took no further part in study procedures or patient follow‐up." Judgement comment: blinding of personnel and participants ensured.
Blinding of participants and personnel (performance bias) Objective outcomes	Low risk	Quote: not applicable Judgement comment: objective outcome for personnel, so low risk of bias regardless of quality of blinding.
Blinding of outcome assessment (detection bias) Subjective outcomes	Low risk	Quote: "The study drug was prepared by an unblinded investigator, who took no further part in study procedures or patient follow‐up." Judgement comment: blinding of outcome assessors ensured. Blinding of outcome assessor also described in trial registration.
Blinding of outcome assessment (detection bias) Objective outcomes	Low risk	Quote: not applicable Judgement comment: objective outcome for outcome assessors, so low risk of bias regardless of quality of blinding.
Incomplete outcome data (attrition bias) All outcomes	Low risk	Quote: not applicable Judgement comment: missing data adequately explained.
Selective reporting (reporting bias)	Low risk	Quote: not applicable Judgement comment: all pre‐specified outcomes reported from protocol in full text.
Other bias	Low risk	Quote: not applicable Judgement comment: the study appears to be free of other bias.

**Table:** Peng 2021

***Study characteristics***		
Methods	**Study design:** RCT **Intention‐to‐treat analysis:** yes **Duration of study:** 18 months (January 2017 to July 2018) + 3 months follow‐up = 21 months **Power calculation reached:** yes **Transfusion strategy:** the criteria for the transfusion of blood products included a haemoglobin level of < 8 g/dL or a haemoglobin level of < 10 g/dL in a patient with symptomatic anaemia, or a patient deemed at high risk because of notable underlying cardiac comorbidities. **Was the trial stopped early:** no	
Participants	**Baseline characteristics** TXA, IV *Age (years) (mean SD)*: 68.13 ± 8.12 *Ethnicity*: not reported *Gender (males, females)*: 6/47 M (13%); 41/47 F (87%) *Length of surgery (minutes)*: 105.36 ± 4.54 *Proportion of participants on anticoagulants prior to surgery (n/N, %)*: 0/47, 0% *Incidence of preoperative anaemia (n/N, %)*: 0/47, 0% *Co‐morbidities (n/N, %)*: not reported *ASA 1 (n/N, %)*: 5/47, 10.6% *ASA 2 (n/N, %)*: 21/47, 44.7% *ASA 3 (n/N, %)*: 21/47, 44.7% *ASA 4 (n/N, %)*: 0/47, 0% *Number of participants randomised*: 49 *Number of participants receiving treatment*: 47 *Number of participants analysed*: 47 *Dropout rate*: 2/49, 4.1% TXA, peri‐articular (PA) *Age (years) (mean SD)*: 68.65 ± 9.54 *Ethnicity*: not reported *Gender (males, females)*: 7/46 M (15%); 39/46 F (85%) *Length of surgery (minutes)*: 104.30 ± 3.52 *Proportion of participants on anticoagulants prior to surgery (n/N, %)*: 0/46, 0% *Incidence of preoperative anaemia (n/N, %)*: 0/46, 0% *Co‐morbidities (n/N, %)*: not reported *ASA 1 (n/N, %)*: 7/46, 15.2% *ASA 2 (n/N, %)*: 27/46, 58.7% *ASA 3 (n/N, %)*: 12/46, 26.1% *ASA 4 (n/N, %)*: 0/46, 0% *Number of participants randomised*: 49 *Number of participants receiving treatment*: 46 *Number of participants analysed*: 46 *Dropout rate*: 3/49, 6.1% Overall *Age (years) (mean SD)*: 68.2 years (range 48 to 80 years) *Ethnicity*: not reported *Gender (males, females)*: 13 M, 80 F *Length of surgery (minutes)*: not reported *Proportion of participants on anticoagulants prior to surgery (n/N, %)*: not reported *Incidence of preoperative anaemia (n/N, %)*: not reported *Co‐morbidities (n/N, %)*: not reported *ASA 1 (n/N, %)*: 12/93, 12.9% *ASA 2 (n/N, %)*: 48/93, 51.6% *ASA 3 (n/N, %)*: 33/93, 35.5% *ASA 4 (n/N, %)*: 0/93, 0% *Number of participants randomised*: 98 *Number of participants receiving treatment*: 93 *Number of participants analysed*: 93 *Dropout rate*: 5/98, 5.1% **Inclusion criteria:** patients undergoing elective unilateral, primary TKA for end‐stage osteoarthritis or rheumatoid arthritis **Exclusion criteria:** patients were then excluded for any of the following reasons: 1) an allergy to TXA; 2) preoperative hepatic or renal dysfunction; 3) serious cardiac or respiratory disease, including coronary artery stent placement; 4) congenital or acquired coagulopathy, as evidenced by an international normalised ratio (INR) of > 1.4 or a partial thromboplastin time (PTT) of > 1.4 times normal; 5) thrombocytopenia, as identified by a preoperative platelet count of < 150,000/mm^3^; 6) a history of a pro‐thrombotic condition; 7) pregnancy; 8) breastfeeding; 9) donated preoperative autologous blood; 10) an age of < 18 years or > 80 years; and/or 11) a preoperative hemoglobin level of < 10 g/dL **If TKR, is tourniquet used:** yes time: TXA IV, 82.36 ± 4.54, TXA PA 82.93 ± 3.21 **Indication for surgery:** end‐stage osteoarthritis or rheumatoid arthritis **Type of anaesthetic:** not reported **Type of surgery:** primary unilateral TKA	
Interventions	**Intervention characteristics** TXA, IV In the IV group, a 60 mL multimodal cocktail periarticular injection (MCPI) with ropivacaine, 200 mg/20 mL (AstraZeneca AB, Sweden); morphine, 10 mg/1 mL (domestic company); flurbiprofen axetil injection, 50 mg/5 mL (Beijing Tide Pharmaceutical Co., Ltd., China); adrenaline, 0.25 mg (1:1000); betamethasone, 7 mg/1 mL (Schering‐Plow Labo NV, Belgium); and 34 mL of normal saline solution was prepared in 3 x 20 mL syringes. Ten minutes before skin incision, patients received 1000 mg of IV TXA (110 mL total volume) IV administration. TXA, IV, 1 g, preop TXA, Peri‐articular In the PA group, the 60 mL MCPI was the same as that in the IV TXA group, except that TXA 1000 mg/10 mL (Guangzhou Baiyunshan Pharmaceutical Co., Ltd., China) was added. Before incision, patients received 110 mL of saline IV administration as a placebo. TXA, PA, 1 g, preop	
Outcomes	*Primary outcomes:* Total blood loss Hidden blood loss Haemoglobin drift Haematocrit drift Need for a blood transfusion *Secondary outcomes:* Deep vein thrombosis D‐dimer levels Complications (including local soft tissue complications, skin necrosis, peroneal nerve palsies, superficial and deep surgical site infections, symptomatic VTEs, cerebrovascular accident and myocardial infarction)	
Notes	**Sponsorship source:** non‐pharmaceutical (2016 Perioperative Orthopeadic Blood Management Research Project from the China International Medical Exchange Foundation) **Country:** China **Setting:** single‐centre **Comments:** none **Author's name:** H‐M Peng **Institution:** Chinese Academy of Medical Sciences & Peking Union Medical College **Email:** penghuiming@139.com, or corresponding author: Jin Lin ‐ lin_chaos@sina.com **Address:** Department of Orthopaedic Surgery, Peking Union Medical College Hospital, Chinese Academy of Medical Sciences & Peking Union Medical College, No.1, Shuaifuyuan Wangfujing, Dongcheng District, Beijing 100730, China **Native language of paper:** English **Reference type:** full text (1), trial registration (1) **Trial registration number:** ChiCTR‐INR‐16010270 **Was it translated for this review:** no	
***Risk of bias***		
**Bias**	**Authors' judgement**	**Support for judgement**
Random sequence generation (selection bias)	Low risk	Quote: "Patients were then randomized to 1 of 2 groups: IV administration of TXA or PAI TXA, in accordance with the random number table." Judgement comment: adequate method of sequence generation with random numbers table.
Allocation concealment (selection bias)	Unclear risk	Quote: "The randomization was concealed by sealed, opaque envelopes and was only accessible to the nurse in the operating room who provided the IV and the injection material for each patient." Judgement comment: envelopes not described as sealed, opaque and sequentially numbered.
Blinding of participants and personnel (performance bias) Subjective outcomes	Low risk	Quote: "The surgeons, patients, anesthesiologist, and the data collection team were blinded to randomization." Judgement comment: subjective outcome for personnel and low risk of bias due to adequate personnel blinding.
Blinding of participants and personnel (performance bias) Objective outcomes	Low risk	Quote: not applicable Judgement comment: objective outcome so low risk of bias regardless of blinding.
Blinding of outcome assessment (detection bias) Subjective outcomes	Low risk	Quote: not applicable Judgement comment: data collection team were blinded.
Blinding of outcome assessment (detection bias) Objective outcomes	Low risk	Quote: not applicable Judgement comment: objective outcome so low risk of bias regardless of blinding.
Incomplete outcome data (attrition bias) All outcomes	Low risk	Quote: not applicable Judgement comment: no obvious outcome data missing.
Selective reporting (reporting bias)	High risk	Quote: not applicable Judgement comment: outcome planned in protocol or prospective trial registry but not reported in the results. The paper has data about how many patients received a transfusion – this is not mentioned in the trial registration. Conversely, the trial registration says it looked at 1) postoperative days required to straight leg elevation, 2) postoperative days required to stand: neither of these are in the full text paper.
Other bias	Low risk	Quote: not applicable Judgement comment: no other concerns such as early stopping or imbalanced study arms.

**Table:** Petsatodis 2006

***Study characteristics***		
Methods	**Study design:** RCT **Intention‐to‐treat analysis:** yes **Duration of study:** not reported **Power calculation reached:** not reported **Transfusion strategy:** not reported **Was the trial stopped early:** no	
Participants	**Baseline characteristics** Placebo *Age (years) (mean SD)*: 59.6 (10.9) *Ethnicity*: not reported *Gender (males, females)*: not reported *Length of surgery (minutes)*: not reported *Proportion of participants on anticoagulants prior to surgery (n/N, %)*: not reported *Incidence of preoperative anaemia (n/N, %): n*ot reported *Co‐morbidities (n/N, %)*: not reported *ASA 1 (n/N, %)*: not reported *ASA 2 (n/N, %)*: not reported *ASA 3 (n/N, %)*: not reported *ASA 4 (n/N, %)*: not reported *Number of participants randomised*: 25 *Number of participants receiving treatment*: 25 *Number of participants analysed*: 25 *Dropout rate*: 0/25 (0%) Aprotinin *Age (years) (mean SD)*: 58.4 (12.5) *Ethnicity*: not reported *Gender (males, females)*: not reported *Length of surgery (minutes)*: not reported *Proportion of participants on anticoagulants prior to surgery (n/N, %)*: not reported *Incidence of preoperative anaemia (n/N, %)*: not reported *Co‐morbidities (n/N, %)*: not reported *ASA 1 (n/N, %)*: not reported *ASA 2 (n/N, %)*: not reported *ASA 3 (n/N, %)*: not reported *ASA 4 (n/N, %)*: not reported *Number of participants randomised*: 25 *Number of participants receiving treatment*: 25 *Number of participants analysed*: 25 *Dropout rate*: 0/25 (0%) Overall *Age (years) (mean SD)*: not reported *Ethnicity*: not reported *Gender (males, females)*: not reported *Length of surgery (minutes)*: not reported *Proportion of participants on anticoagulants prior to surgery (n/N, %)*: not reported *Incidence of preoperative anaemia (n/N, %)*: not reported *Co‐morbidities (n/N, %)*: not reported *ASA 1 (n/N, %)*: not reported *ASA 2 (n/N, %)*: not reported *ASA 3 (n/N, %)*: not reported *ASA 4 (n/N, %)*: not reported *Number of participants randomised*: 50 *Number of participants receiving treatment*: 50 *Number of participants analysed*: 50 *Dropout rate*: 0 **Inclusion criteria:** 1) patients with hip osteoarthritis necessitating THA **Exclusion criteria:** 1) suspected allergy to aprotinin or previous treatment with the drug **If TKR, is tourniquet used:** not applicable **Indication for surgery:** hip osteoarthritis **Type of anaesthetic:** not reported **Type of surgery:** primary THR	
Interventions	**Intervention characteristics** Placebo Same volume of normal saline Placebo, IV, intraop bolus at induction + placebo, IV, infusion Aprotinin Bolus of 20,000 kIU/kg at the time of anaesthesia followed by an infusion of 50,000 KIU/hr Aprotinin, IV, 20,000 KIU/kg, intraop bolus at induction AND aprotinin, IV, 50,000 KIU/hr, infusion	
Outcomes	*Primary outcomes:* Intra and postoperative blood loss Amount of transfusion units *Secondary outcomes:* Clinical signs of DVT	
Notes	**Sponsorship source:** not reported **Country:** Greece **Setting:** not reported **Comments:** none **Author's name:** G Petsatodis **Institution:** Aristotle University **Email:** not reported **Address:** 1st Orthopaedic Department, G. Papanikolaou Hospital, Aristotle University, Thessaloniki, Greece **Native language of paper:** English **Reference type:** full text (1) **Trial registration number:** not applicable, but when checked, trial registration number not found **Was it translated for this review:** no	
***Risk of bias***		
**Bias**	**Authors' judgement**	**Support for judgement**
Random sequence generation (selection bias)	Unclear risk	Quote: "Patients were randomized using the envelope technique" Judgement comment: insufficient information to permit judgement.
Allocation concealment (selection bias)	Unclear risk	Quote: "Patients were randomized using the envelope technique" Judgement comment: insufficient information to permit judgement.
Blinding of participants and personnel (performance bias) Subjective outcomes	Unclear risk	Quote: not applicable Judgement comment: no description given of personnel blinding.
Blinding of participants and personnel (performance bias) Objective outcomes	Low risk	Quote: not applicable Judgement comment: objective outcome for personnel, so low risk of bias regardless of quality of blinding.
Blinding of outcome assessment (detection bias) Subjective outcomes	Unclear risk	Quote: not applicable Judgement comment: no description given of outcome assessor blinding.
Blinding of outcome assessment (detection bias) Objective outcomes	Low risk	Quote: not applicable Judgement comment: objective outcome for outcome assessor, so low risk of bias regardless of quality of blinding
Incomplete outcome data (attrition bias) All outcomes	Low risk	Quote: not applicable Judgement comment: no missing outcome data.
Selective reporting (reporting bias)	Unclear risk	Quote: not applicable Judgement comment: no reporting of gender information. No protocol available for review.
Other bias	Unclear risk	Quote: not applicable Judgement comment: no funding information declared.

**Table:** Ray 2005

***Study characteristics***		
Methods	**Study design:** RCT **Intention‐to‐treat analysis:** yes **Duration of study:** not reported **Power calculation reached:** no **Transfusion strategy:** not reported **Was the trial stopped early:** yes	
Participants	**Baseline characteristics** Placebo *Age (years) (median IQR)*: 69 (58 to 74) *Ethnicity*: not reported *Gender (males, females)*: not reported *Length of surgery (minutes) (median IQR)*: 104 (87 to 116) *Proportion of participants on anticoagulants prior to surgery (n/N, %)*: 3/15 (20%) *Incidence of preoperative anaemia (n/N, %)*: not reported *Co‐morbidities (n/N, %)*: not reported *ASA 1 (n/N, %)*: not reported *ASA 2 (n/N, %)*: not reported *ASA 3 (n/N, %)*: not reported *ASA 4 (n/N, %)*: not reported *Number of participants randomised*: 15 *Number of participants receiving treatment*: 15 *Number of participants analysed*: 15 *Dropout rate*: 0/15 (0%) EACA *Age (years) (median IQR)*: 72 (59 to 77) *Ethnicity*: not reported *Gender (males, females)*: not reported *Length of surgery (minutes) (median IQR)*: 111 (89 to 125) *Proportion of participants on anticoagulants prior to surgery (n/N, %)*: 2/15 (13.3%) *Incidence of preoperative anaemia (n/N, %)*: not reported *Co‐morbidities (n/N, %)*: not reported *ASA 1 (n/N, %)*: not reported *ASA 2 (n/N, %)*: not reported *ASA 3 (n/N, %)*: not reported *ASA 4 (n/N, %)*: not reported *Number of participants randomised*: 15 *Number of participants receiving treatment*: 15 *Number of participants analysed*: 15 *Dropout rate*: 0/15 (0%) Aprotinin *Age (years) (median IQR)*: 72 (65 to 81) *Ethnicity*: not reported *Gender (males, females)*: not reported *Length of surgery (minutes) (median IQR)*: 100 (75 to 120) *Proportion of participants on anticoagulants prior to surgery (n/N, %)*: 2/15 (13.3%) *Incidence of preoperative anaemia (n/N, %)*: not reported *Co‐morbidities (n/N, %)*: not reported *ASA 1 (n/N, %)*: not reported *ASA 2 (n/N, %)*: not reported *ASA 3 (n/N, %)*: not reported *ASA 4 (n/N, %)*: not reported *Number of participants randomised*: 15 *Number of participants receiving treatment*: 15 *Number of participants analysed*: 15 *Dropout rate*: 0/15 (0%) Overall *Age (years) (median IQR)*: not reported *Ethnicity*: not reported *Gender (males, females)*: not reported *Length of surgery (minutes) (median IQR)*: not reported *Proportion of participants on anticoagulants prior to surgery (n/N, %)*: not reported *Incidence of preoperative anaemia (n/N, %)*: not reported *Co‐morbidities (n/N, %)*: not reported *ASA 1 (n/N, %)*: not reported *ASA 2 (n/N, %)*: not reported *ASA 3 (n/N, %)*: not reported *ASA 4 (n/N, %)*: not reported *Number of participants randomised*: not reported *Number of participants receiving treatment*: not reported *Number of participants analysed*: not reported *Dropout rate*: not reported **Inclusion criteria:** 1) undergoing primary, unilateral cemented THA under general anaesthesia, 2) preoperative haemoglobin of < 150 g/L‐1 for males and < 140 g/L‐1 for females **Exclusion criteria:** 1) previous exposure to aprotinin (to avoid the increased likelihood of allergic response entailed with such exposure), 2) abnormal preoperative coagulation profile (with the exception of platelet function as measured by whole blood platelet aggregometry employing collagen agonist at 1 μg/mL), 3) previous history of DVT, pulmonary embolus, stroke or transient ischaemic attacks, 4) a strong family history of thrombosis, 5) malignancy, 6) renal failure (creatinine > 0.15 mmol/L), 7) patients who would not consent to receive blood transfusion if required **If TKR, is tourniquet used:** not applicable **Indication for surgery:** not reported **Type of anaesthetic:** general anaesthetic **Type of surgery:** primary THR	
Interventions	**Intervention characteristics** Placebo Patients in the placebo group received saline only in the same way. Placebo, IV, intraop bolus over 30 mins AND placebo, IV intraop infusion for 3 hours EACA Patients in the EACA group received 10 g of EACA in 250 mL of IV saline given over 30 min after the induction of anaesthesia followed by 5 g in 250 mL of IV saline over 3 h EACA, IV, 10 g, intraop bolus over 30 mins AND EACA, IV, 5 g, intraop infusion for 3 hours Aprotinin Patients assigned to the aprotinin group received a 10,000 kallikrein inhibitor units (KIU) test dose of aprotinin (preservative free Trasylol, Bayer AG, Leverkusen, Germany) to test for any potential allergic reaction. Ten minutes later, aprotinin was administered as a bolus dose of 2 x 10^6 KIU given over 30 min after induction of anaesthesia followed by 0.5 x 10^6 KIU h for 3 h Aprotinin, IV, 10,000 KIU, preop test dose AND aprotinin, IV, 2 x 10^6, intraop bolus over 30 mins AND aprotinin, IV, 0.5 x 10^6 KIU, intraop infusion for 3 hours	
Outcomes	*Primary outcomes:* Blood loss Transfusion requirements Complications Adverse events *Secondary outcome:* Not reported	
Notes	**Sponsorship source:** non‐pharmaceutical **Country:** Australia **Setting:** single‐centre **Comments:** none **Author's name:** R Crawford **Institution:** The Prince Charles Hospital **Email:** r.crawford@ qut.edu.au **Address:** Level 5, Clinical Science Building, The Prince Charles Hospital, Brisbane, Queensland 4032, Australia **Native language of paper:** English **Reference type:** abstract (1) full text (1) **Trial registration number:** not applicable, but when checked, trial registration number not found **Was it translated for this review:** no	
***Risk of bias***		
**Bias**	**Authors' judgement**	**Support for judgement**
Random sequence generation (selection bias)	Unclear risk	Quote: "patients were randomly allocated to receive aprotinin, EACA or placebo." Judgement comment: insufficient information to permit judgement.
Allocation concealment (selection bias)	Unclear risk	Quote: not applicable Judgement comment: insufficient information to permit judgement.
Blinding of participants and personnel (performance bias) Subjective outcomes	Unclear risk	Quote: not applicable Judgement comment: no description of participant or personnel blinding given.
Blinding of participants and personnel (performance bias) Objective outcomes	Low risk	Quote: not applicable Judgement comment: objective outcome for personnel, so low risk of bias regardless of quality of blinding.
Blinding of outcome assessment (detection bias) Subjective outcomes	Unclear risk	Quote: not applicable Judgement comment: no description of outcome assessor blinding given.
Blinding of outcome assessment (detection bias) Objective outcomes	Low risk	Quote: not applicable Judgement comment: objective outcome for outcome assessor, so low risk of bias regardless of quality of blinding.
Incomplete outcome data (attrition bias) All outcomes	Unclear risk	Quote: not applicable Judgement comment: insufficient information to permit judgement. Also, the gender split between the arms was not reported. No n numbers in outcome tables.
Selective reporting (reporting bias)	High risk	Quote: "Six patients experienced adverse cardiac events postoperatively; two non‐ST elevation myocardial infarction, two atrial fibrillation and two with both. This did not constitute a significant increase in the treatment arms (P ¼ 0.08)." Judgement comment: outcomes reported incompletely such that they cannot be entered into the the meta‐analysis.
Other bias	High risk	Quote: not applicable Judgement Comment: Trial stopped early. Group baseline differences.

**Table:** Schott 1995

***Study characteristics***		
Methods	**Study design:** RCT **Intention‐to‐treat analysis:** yes **Duration of study:** not reported **Power calculation reached:** no **Transfusion strategy:** erythrocyte volume fraction (EVF) heterologous erythrocyte concentrate was transfused to correct EVF > 27%. **Was the trial stopped early:** no	
Participants	**Baseline characteristics** Placebo *Age (years) (mean SD)*: 68 (7) *Ethnicity*: not reported *Gender (males, females)*: 15/40 M (38%); 25/40 F (62%) *Length of surgery (minutes)*: not reported *Proportion of participants on anticoagulants prior to surgery (n/N, %)*: 0/40 (0%) *Incidence of preoperative anaemia (n/N, %)*: 0/40 (0%) *Co‐morbidities (n/N, %)*: not reported *ASA 1 (n/N, %)*: not reported *ASA 2 (n/N, %)*: not reported *ASA 3 (n/N, %)*: not reported *ASA 4 (n/N, %)*: not reported *Number of participants randomised*: 40 *Number of participants receiving treatment*: 40 *Number of participants analysed*: 40 *Dropout rate*: 0/40 (0%) Desmopressin *Age (years) (mean SD)*: 71 (9) *Ethnicity*: not reported *Gender (males, females)*: 20/39 M (51%); 19/39 F (49%) *Length of surgery (minutes)*: not reported *Proportion of participants on anticoagulants prior to surgery (n/N, %)*: 0, 0% *Incidence of preoperative anaemia (n/N, %)*: 0/39 (0%) *Co‐morbidities (n/N, %)*: not reported *ASA 1 (n/N, %)*: not reported *ASA 2 (n/N, %)*: not reported *ASA 3 (n/N, %)*: not reported *ASA 4 (n/N, %)*: not reported *Number of participants randomised*: 40 *Number of participants receiving treatment*: 39 *Number of participants analysed*: 39 *Dropout rate*: 1/40 (2.5%) Overall *Age (years) (mean SD)*: not reported *Ethnicity*: not reported *Gender (males, females)*: not reported *Length of surgery (minutes)*: not reported *Proportion of participants on anticoagulants prior to surgery (n/N, %)*: not reported *Incidence of preoperative anaemia (n/N, %)*: not reported *Co‐morbidities (n/N, %)*: not reported *ASA 1 (n/N, %)*: not reported *ASA 2 (n/N, %)*: not reported *ASA 3 (n/N, %)*: not reported *ASA 4 (n/N, %)*: not reported *Number of participants randomised*: 80 *Number of participants receiving treatment*: 79 *Number of participants analysed*: 79 *Dropout rate*: 1/79 **Inclusion criteria:** 1) normally haemostatic patients, scheduled for elective total hip replacement surgery for primary coxarthrosis **Exclusion criteria:** 1) patients with secondary coxarthrosis were excluded, 2) patients receiving acetylsalicylic acid or any other platelet inhibiting medication within 10 days prior to surgery, 3) patients suffering from anaemia, diabetes mellitus, rheumatic disease or any disorder requiring steroid therapy, 4) abnormal preoperative coagulation status and an abnormal bleeding time > 580 s (Simplate II test, which was performed by the same person in all patients) **If TKR, is tourniquet used:** not applicable **Indication for surgery:** primary coxarthrosis **Type of anaesthetic:** spinal anaesthetic **Type of surgery:** primary THR	
Interventions	**Intervention characteristics** Placebo Immediately after induction of spinal anaesthesia, the patient received in a double‐blind fashion placebo or desmopressin, 0.3 μg/kg BW diluted in 50 ml NaCI and infused over 15 minutes. Six hours after the first dose (i.e. post‐surgery, a second infusion was administered in an identical way). Placebo, IV, intraop (immediately after spinal) AND placebo, IV, 6 hours post 1st dose Desmopressin Immediately after induction of spinal anaesthesia, the patient received in a double‐blind fashion placebo or desmopressin, 0.3 pg/kg B\V diluted in 50 mL NaCI and infused over 15 minutes. Six hours after the first dose (i.e. post‐surgery), a second infusion was administered in an identical way. Desmopressin, IV, 0.3 μg/kg, intraop (immediately after spinal) AND desmopressin, IV, 0.3 μg/kg, 6 hours post 1st dose	
Outcomes	*Primary outcomes:* Blood loss Transfusions and dextran infusions Haemostasis	
Notes	**Sponsorship source:** pharmaceutical **Country:** Sweden **Setting:** not reported **Comments:** none **Author's name:** U Schott **Institution:** Orebro Medical Center Hospital **Email:** not reported **Address:** Departments of Anesthesiology and Orthopedics, Regional Blood Centre, Orebro Medical Center Hospital, Orebro, Sweden **Native language of paper:** English **Reference type:** full text (1) **Trial registration number:** not applicable, but when checked, trial registration number not found **Was it translated for this review:** no	
***Risk of bias***		
**Bias**	**Authors' judgement**	**Support for judgement**
Random sequence generation (selection bias)	Unclear risk	Quote: "The study was conducted as a prospective, randomized, double‐blind and placebo‐controlled study in 80 (at a level of 5% and 80% power) normally haemostatic patients, scheduled for elective total hip replacement (THR) surgery for primary coxarthrosis." Judgement comment: insufficient information to permit judgement.
Allocation concealment (selection bias)	Unclear risk	Quote: "Immediately after induction of spinal anaesthesia, the patient received in a double‐blind fashion placebo or desmopressin,0.3 ug/kg BW, diluted in 50 ml NaCI and infused over 15 minutes" Judgement comment: insufficient information to permit judgement.
Blinding of participants and personnel (performance bias) Subjective outcomes	Unclear risk	Quote: "By infusing desmopressin immediately after the administration of spinal anaesthesia, blindness was achieved with regard to the vasodilatory effect of both desmopressin and spinal anaesthesia." Judgement comment: insufficient information to permit judgement.
Blinding of participants and personnel (performance bias) Objective outcomes	Low risk	Quote: not applicable Judgement comment: objective outcome for personnel, so low risk of bias regardless of quality of blinding.
Blinding of outcome assessment (detection bias) Subjective outcomes	Unclear risk	Quote: not applicable Judgement comment: no description given of personnel blinding. Insufficient information to permit judgement.
Blinding of outcome assessment (detection bias) Objective outcomes	Low risk	Quote: not applicable Judgement comment: objective outcome for outcome assessor, so low risk of bias regardless of quality of blinding.
Incomplete outcome data (attrition bias) All outcomes	Low risk	Quote: not applicable Judgement comment: all outcome data presented.
Selective reporting (reporting bias)	Unclear risk	Quote: not applicable Judgement comment: no protocol available to check pre‐specified outcomes reported. Insufficient information to permit judgement.
Other bias	Low risk	Quote: not applicable Judgement comment: paper appears to be free of other bias.

**Table:** Sershon 2020

***Study characteristics***		
Methods	**Study design:** RCT **Intention‐to‐treat analysis:** no, per protocol analysis done ‐ report both ITT and per protocol **Duration of study:** 37 months (July 2016 to August 2019) + follow‐up (follow‐up period not reported) **Power calculation reached:** yes **Transfusion strategy:** haemoglobin < 7.0 g/dL or symptomatic anaemia not responsive to IV fluid hydration, history of cardiac disease and haemoglobin < 8.0 g/dL or symptomatic anaemia not responsive to IV fluid hydration. Active arterial thromboembolic event and haemoglobin < 10.0 g/dL, sepsis with haemoglobin < 10.0 g/dL, or symptomatic anaemia not responsive to IV fluid hydration. **Was the trial stopped early:** no	
Participants	**Baseline characteristics** Single‐dose IV group *Age (years) (mean SD)*: 66.8 (12.0) *Ethnicity*: not reported *Gender (males, females)*: 23/43 M (53%), 20/43 F (47%) *Length of surgery (minutes) (mean SD)*: 134 (57) *Proportion of participants on anticoagulants prior to surgery (n/N, %)*: aspirin: 35/43 (81.4%); warfarin: 4/43 (9.3%); apixaban: 0/43 (0%); enoxaparin: 4/43 (9.3%); rivaroxaban: 0/43 (0%) *Incidence of preoperative anaemia (n/N, %)*: not reported *Co‐morbidities (n/N, %)*: not reported *ASA 1 (n/N, %)*: mean only: 2.5 ± 0.6 *ASA 2 (n/N, %)*: not reported *ASA 3 (n/N, %)*: not reported *ASA 4 (n/N, %)*: not reported *Number of participants randomised*: 47 *Number of participants receiving treatment*: 43 *Number of participants analysed*: 43 *Dropout rate*: 4/47 (8.5%) Double‐dose IV group *Age (years) (mean SD)*: 67.8 (13.2 ) *Ethnicity*: not reported *Gender (males, females)*: 18/40 M (45%), 22/40 F (55%) *Length of surgery (minutes) (mean SD)*: 152 (66) *Proportion of participants on anticoagulants prior to surgery (n/N, %)*: aspirin: 31/40 (77.5%); warfarin: 4/40 (10%); apixaban: 0/40 (0%); enoxaparin: 3/40 (7.5%); rivaroxaban: 2/40 (5.0%) *Incidence of preoperative anaemia (n/N, %)*: not reported *Co‐morbidities (n/N, %)*: not reported *ASA 1 (n/N, %)*: mean only: 2.5 ± 0.6 *ASA 2 (n/N, %)*: not reported *ASA 3 (n/N, %)*: not reported *ASA 4 (n/N, %)*: not reported *Number of participants randomised*: 46 *Number of participants receiving treatment*: 40 *Number of participants analysed*: 40 *Dropout rate*: 6/46, 13% Combined IV and topical group *Age (years) (mean SD)*: 66.5 (14.4) *Ethnicity*: not reported *Gender (males, females)*: 19/46 M (41%), 27/46 F (59%) *Length of surgery (minutes) (mean SD)*: 155 ± 57 *Proportion of participants on anticoagulants prior to surgery (n/N, %)*: aspirin: 37/46 (80.4%); warfarin: 3/46 (6.5%); apixaban: 1/46 (2.2%); enoxaparin: 3/46 (6.5%); rivaroxaban: 2/46 (4.4%) *Incidence of preoperative anaemia (n/N, %)*: not reported *Co‐morbidities (n/N, %)*: not reported *ASA 1 (n/N, %)*: mean only: 2.5 ± 0.6 *ASA 2 (n/N, %)*: not reported *ASA 3 (n/N, %)*: not reported *ASA 4 (n/N, %)*: not reported *Number of participants randomised*: 54 *Number of participants receiving treatment*: 46 *Number of participants analysed*:*46* *Dropout rate*: 8/54, 14.8% Multi‐dose oral group *Age (years) (mean SD)*: 64.0 (12.3) *Ethnicity*: not reported *Gender (males, females)*: 26/46 M (56%), 20/46 F (44%) *Length of surgery (minutes) (mean SD)*: 148 (46) *Proportion of participants on anticoagulants prior to surgery (n/N, %)*: aspirin: 33/46 (71.7%); warfarin: 5/46 (10.9%); apixaban: 1/46 (2.2%); enoxaparin: 4/46 (8.7%); rivaroxaban: 3/46 (6.5%) *Incidence of preoperative anaemia (n/N, %)*: not reported *Co‐morbidities (n/N, %)*: not reported *ASA 1 (n/N, %)*: mean only: 2.4 ± 0.6 *ASA 2 (n/N, %)*: not reported *ASA 3 (n/N, %)*: not reported *ASA 4 (n/N, %)*: not reported *Number of participants randomised*: 54 *Number of participants receiving treatment*: 46 *Number of participants analysed*: 46 *Dropout rate*: 8/54, 14.8% Overall *Age (years) (mean SD)*: not reported *Ethnicity*: not reported *Gender (males, females)*: 86/175 M (49%), 89/175 F (51%) *Length of surgery (minutes) (mean SD)*: not reported *Proportion of participants on anticoagulants prior to surgery (n/N, %)*: not reported *Incidence of preoperative anaemia (n/N, %)*: not reported *Co‐morbidities (n/N, %)*: not reported *ASA 1 (n/N, %)*: not reported *ASA 2 (n/N, %)*: not reported *ASA 3 (n/N, %)*: not reported *ASA 4 (n/N, %)*: not reported *Number of participants randomised*: 251 *Number of participants receiving treatment*: 175 *Number of participants analysed*: 175 *Dropout rate*: 76/251, 30.3% **Inclusion criteria:** patients indicated for a revision total hip arthroplasty were approached to participate. Revision total hip arthroplasty was defined as a both‐component (femoral and acetabular) revision, femoral revision, acetabular revision, resection arthroplasty with antibiotic spacer insertion, or second‐stage reimplantation **Exclusion criteria:** individuals indicated for a modular head and polyethylene liner revision were excluded. Patients were also excluded for any of the following health conditions: prior thromboembolic event, anticoagulant medication (excluding aspirin) within 5 days prior to the surgical procedure, New York Heart Association Class‐III or IV heart failure, myocardial infarction or cardiac stenting within 6 months of the surgical procedure, hepatic failure, renal failure requiring dialysis, pulmonary disease requiring supplemental oxygen, documented allergy to TXA, acquired disturbances of colour vision, and the refusal to receive blood products **If TKR, is tourniquet used:** not applicable **Indication for surgery:** not reported **Type of anaesthetic:** general, general and nerve block, spinal and epidural, spinal, spinal and nerve block, general and epidural, epidural, spinal and general **Type of surgery:** revision THA	
Interventions	**Intervention characteristics** Single‐dose IV group The single‐dose IV group was administered a single dose of 1 g IV TXA prior to incision and during preparing and draping TXA, IV, 1g, preop Double‐dose IV group The double dose IV group was administered a double dose of IV TXA, in which the first dose of 1 g IV TXA was administered during preparing and draping with an additional 1 g IV TXA given at the time of skin closure TXA, IV, 1 g, preop + IV, 1 g, intraop Combined IV and topical group The combined IV and topical dose group, was administered a dose of 1 g IV TXA during preparing and draping and a dose of 1 g intra‐articular TXA was introduced immediately following arthrotomy closure TXA, IV, 1 g, preop + IA, 1 g, intraop Multi‐dose oral group The multidose oral group was administered a total of 1950 mg (3 tablets of 650 mg each) of oral TXA 2 hours prior to incision, 6 hours postoperatively and on the morning of postoperative day 1. TXA, oral, 1 x 650 g, preop + 1 x 650 g 6 h postop + 1 x 650 g day 1 postop am	
Outcomes	*Primary outcome:* Postoperative haemoglobin value *Secondary outcomes:* Deep vein thrombosis Venous thromboembolic complications Cerebrovascular events Transient ischaemic attacks Calculated blood loss Rate of transfusion Need for transfusion Length of stay	
Notes	**Sponsorship source:** not reported but this information was given... "Disclosure: The authors received partial financial support through a multipurpose resident grant from the Mid‐America Orthopaedic Association. On the Disclosure of Potential Conflicts of Interest forms, which are provided with the online version of the article, one or more of the authors checked “yes” to indicate that the author had a relevant financial relationship in the biomedical arena outside the submitted work and “yes” to indicate that the author had other relationships or activities that could be perceived to influence, or have the potential to influence, what was written in this work" **Country:** USA **Setting:** multi‐centre **Comments:** ASA given as an average, not individual grades (ASA I, ASA II, ASA III etc.) **Author's name:** RA Sershon **Institution:** Anderson Orthopaedic Research Institute **Email:** bobsershon@gmail.com **Address:** Department of Orthopedic Surgery, Anderson Orthopaedic Research Institute, Alexandria, Virginia, USA **Native language of paper:** English **Reference type:** full text (2), trial registration (1) **Trial registration number:** NCT02877381 **Was it translated for this review:** no	
***Risk of bias***		
**Bias**	**Authors' judgement**	**Support for judgement**
Random sequence generation (selection bias)	Low risk	Quote: "Randomization for each revision subtype was then performed using a random number generator, assigning patients to 1 of 4 TXA intervention groups." Judgement comment: adequate method of sequence generation with random number generator.
Allocation concealment (selection bias)	Unclear risk	Quote: not applicable Judgement comment: method of allocation concealment not described.
Blinding of participants and personnel (performance bias) Subjective outcomes	High risk	Quote: "Blinding for the study was not feasible because of the financial and organizational constraints of a multicenter randomized clinical trial." Judgement comment: subjective outcome for personnel and high risk of bias due to inadequate personnel blinding.
Blinding of participants and personnel (performance bias) Objective outcomes	Low risk	Quote: not applicable Judgement comment: objective outcome for personnel, so low risk of bias regardless of quality of blinding.
Blinding of outcome assessment (detection bias) Subjective outcomes	High risk	Quote: not applicable Judgement comment: subjective outcome for personnel and high risk of bias due to inadequate personnel blinding.
Blinding of outcome assessment (detection bias) Objective outcomes	Low risk	Quote: not applicable Judgement comment: objective outcome for personnel, so low risk of bias regardless of quality of blinding.
Incomplete outcome data (attrition bias) All outcomes	Low risk	Quote: not applicable Judgement comment: no obvious outcome data missing.
Selective reporting (reporting bias)	High risk	Quote: not applicable Judgement comment: some outcomes reported in trial registration are not reported in the full text (e.g. return to the operating room).
Other bias	Low risk	Quote: not applicable Judgement comment: no other concerns such as early stopping or imbalanced study arms.

**Table:** Staniforth 2017

***Study characteristics***		
Methods	**Study design:** RCT **Intention‐to‐treat analysis:** unclear **Duration of study:** 20 months (17 + 3 months follow‐up) **Power calculation reached:** not reported **Transfusion strategy:** not reported **Was the trial stopped early:** no	
Participants	**Baseline characteristics** Placebo *Age (years) (mean SD)*: not reported *Ethnicity*: not reported *Gender (males, females)*: not reported *Length of surgery (minutes)*: not reported *Proportion of participants on anticoagulants prior to surgery (n/N, %)*: 0/0 (0%) *Incidence of preoperative anaemia (n/N, %)*: not reported *Co‐morbidities (n/N, %)*: not reported *ASA 1 (n/N, %)*: not reported *ASA 2 (n/N, %)*: not reported *ASA 3 (n/N, %)*: not reported *ASA 4 (n/N, %)*: not reported *Number of participants randomised*: 70 *Number of participants receiving treatment*: not reported *Number of participants analysed*: not reported *Dropout rate*: not reported TXA, IA *Age (years) (mean SD)*: not reported *Ethnicity*: not reported *Gender (males, females)*: not reported *Length of surgery (minutes)*: not reported *Proportion of participants on anticoagulants prior to surgery (n/N, %)*: 0/0 (0%) *Incidence of preoperative anaemia (n/N, %)*: not reported *Co‐morbidities (n/N, %)*: not reported *ASA 1 (n/N, %)*: not reported *ASA 2 (n/N, %)*: not reported *ASA 3 (n/N, %)*: not reported *ASA 4 (n/N, %)*: not reported *Number of participants randomised*: 70 *Number of participants receiving treatment*: not reported *Number of participants analysed*: not reported *Dropout rate*: not reported Overall *Age (years) (mean SD)*: not reported *Ethnicity*: not reported *Gender (males, females)*: not reported *Length of surgery (minutes)*: not reported *Proportion of participants on anticoagulants prior to surgery (n/N, %)*: not reported *Incidence of preoperative anaemia (n/N, %)*: not reported *Co‐morbidities (n/N, %)*: not reported *ASA 1 (n/N, %)*: not reported *ASA 2 (n/N, %)*: not reported *ASA 3 (n/N, %)*: not reported *ASA 4 (n/N, %)*: not reported *Number of participants randomised*: 140 *Number of participants receiving treatment*: not reported *Number of participants analysed*: not reported *Dropout rate*: not reported **Inclusion criteria:** 1) 18 years and older, 2) osteoarthritis, 3) scheduled for elective primary unilateral THR or TKR, 4) provided informed consent, 5) can read, write and speak English **Exclusion criteria:** 1) history of arterial or venous thromboembolic disease (myocardial infarction, symptomatic ischaemic heart disease, atrial fibrillation, cerebrovascular accident, deep vein thrombosis, pulmonary embolus, or thrombogenic cardiac valvular disease or rhythm disease), 2) pre‐operative Hb of < 120 g/L, 3) known allergy to tranexamic acid, 4) coagulation disorder, 5) acquired disturbances of colour vision, 6) hepatic insufficiency, any history of liver disease, 7) renal insufficiency (on dialysis), 8) preoperative prophylactic use of antiplatelet or anticoagulant therapy such as clopidogrel, warfarin, dabigatran or rivaroxaban. This does not include low dose aspirin (81mg), 9) patients with a history of subarachnoid haemorrhage, 10) simultaneous bilateral THA or TKA, 11) any contra‐indication for spinal anaesthesia, 12) allergy to celecoxib, which will be the only nonsteroidal anti‐inflammatory drugs (NSAID) used in the multi‐modal analgesia regime, 13) retinal vein or retinal artery occlusion, 14) female on oral contraceptive pills and/or premenopausal, 15) concurrently taking hydrochlorothiazide, desmopressin, sulbactam‐ampicillin, carbazochrome, ranitidine and/or nitroglycerin for the duration of the surgery **If TKR, is tourniquet used:** not reported **Indication for surgery:** osteoarthritis **Type of anaesthetic:** spinal **Type of surgery:** primary THR and primary TKR	
Interventions	**Intervention characteristics** Placebo They were randomised to either receive 0.5 g of TXA in 50 mL NS or 50 mL of NS injected intra‐articularly after closure of the joint capsule during surgery Placebo, IA, intraop after closure through temporary catheter TXA, IA They were randomised to either receive 0.5 g of TXA in 50 mL NS or 50 mL of NS injected intra‐articularly after closure of the joint capsule during surgery TXA, IA, 0.5 g, intraop after closure through temporary catheter	
Outcomes	*Primary outcomes:* Blood loss Thromboembolic complications Blood transfusions Other postoperative complications	
Notes	**Sponsorship source:** non‐pharmaceutical **Country:** Canada **Setting:** single‐centre **Comments:** none **Author's name:** C. Staniforth **Institution:** University of Manitoba **Email:** not reported **Address:** Orthopaedic Innovation Centre Concordia Hip & Knee Institute Suite 320‐1155 Concordia Avenue Winnipeg, Manitoba, Canada R2K 2M9 **Native language of paper:** English **Reference type:** abstract (1) trial registration (1) **Trial registration number:** NCT02393963 **Was it translated for this review:** no	
***Risk of bias***		
**Bias**	**Authors' judgement**	**Support for judgement**
Random sequence generation (selection bias)	Unclear risk	Quote: not applicable Judgement comment: insufficient information to permit judgement.
Allocation concealment (selection bias)	Unclear risk	Quote: not applicable Judgement comment: insufficient information to permit judgement.
Blinding of participants and personnel (performance bias) Subjective outcomes	Low risk	Quote: not applicable Judgement comment: participant and care provider were masked (from trial registration).
Blinding of participants and personnel (performance bias) Objective outcomes	Low risk	Quote: not applicable Judgement comment: objective outcome for personnel, so low risk of bias regardless of quality of blinding.
Blinding of outcome assessment (detection bias) Subjective outcomes	Low risk	Quote: not applicable Judgement comment: investigators were masked (from trial registration)
Blinding of outcome assessment (detection bias) Objective outcomes	Low risk	Quote: not applicable Judgement comment: objective outcome for outcome assessor, so low risk of bias regardless of quality of blinding.
Incomplete outcome data (attrition bias) All outcomes	Unclear risk	Quote: not applicable Judgement comment: insufficient information to permit judgement.
Selective reporting (reporting bias)	High risk	Quote: not applicable Judgement comment: pre‐specified outcomes in trial registration not reported. One or more outcomes of interest in the review are reported incompletely, so they cannot be entered into the meta‐analysis.
Other bias	Unclear risk	Quote: not applicable Judgement comment: insufficient information to permit judgement. Need full‐text paper to permit judgement.

**Table:** Stowers 2017

***Study characteristics***		
Methods	**Study design:** RCT **Intention‐to‐treat analysis:** yes **Duration of study:** 16 months + follow‐up **Power calculation reached:** yes **Transfusion strategy:** the criteria for transfusion of blood products was Hb < 80 g/L, or Hb < 100 g/L in a patient with ischaemic heart disease or with symptomatic anaemia. Intravenous fluids in the perioperative period were also measured to account for any differences in haemodilution effect on Hb recordings between the 3 groups. **Was the trial stopped early:** no	
Participants	**Baseline characteristics** Placebo *Age (years) (mean SD)*: 70 (7.6) *Ethnicity*: not reported *Gender (males, females)*: 4/23 M (17%); 19/23 F (83%) *Length of surgery (minutes)*: not reported *Proportion of participants on anticoagulants prior to surgery (n/N, %)*: not reported *Incidence of preoperative anaemia (n/N, %)*: not reported *Co‐morbidities (n/N, %)*: type 2 diabetes ‐ 4/23, 17%, hypertension ‐ 12/23, 52%, cholesterol ‐ 4/23, 17%, chronic obstructive pulmonary disease ‐ 0/23, 0%, ischaemic heart disease ‐ 0/23, 0% *ASA 1 (n/N, %)*: 4/23, (17.39%) *ASA 2 (n/N, %)*: 15/23, (65.22%) *ASA 3 (n/N, %)*: 4/23, (17.39%) *ASA 4 (n/N, %)*: not reported *Number of participants randomised*: 30 *Number of participants receiving treatment*: not reported *Number of participants analysed*: 23 *Dropout rate*: 7/30, (23.33) TXA, IA *Age (years) (mean SD)*: 70 (8.5) *Ethnicity*: not reported *Gender (males, females)*: 28/60 M (47%); 32/60 F (53%) *Length of surgery (minutes)*: not reported *Proportion of participants on anticoagulants prior to surgery (n/N, %)*: not reported *Incidence of preoperative anaemia (n/N, %)*: not reported *Co‐morbidities (n/N, %)*: type 2 diabetes ‐ 14/60, 23%, hypertension ‐ 36/60, 60%, cholesterol ‐ 17/60, 28%, chronic obstructive pulmonary disease ‐ 6/60, 10%, ischaemic heart disease ‐ 4/60, 7% *ASA 1 (n/N, %)*: 4/60, (6.67%) *ASA 2 (n/N, %)*: 48/60, (80%) *ASA 3 (n/N, %)*: 8/60, (13.33%) *ASA 4 (n/N, %)*: not reported *Number of participants randomised*: 60 *Number of participants receiving treatment*: not reported *Number of participants analysed*: 60 *Dropout rate*: 0/60, (0%) TXA, IV *Age (years) (mean SD)*: 71 (8.6) *Ethnicity*: not reported *Gender (males, females)*: 27/51 M (53%); 24/51 F (47%) *Length of surgery (minutes)*: not reported *Proportion of participants on anticoagulants prior to surgery (n/N, %)*: not reported *Incidence of preoperative anaemia (n/N, %)*: not reported *Co‐morbidities (n/N, %)*: type 2 diabetes ‐ 5/51, 10%, hypertension ‐ 29/51, 57%, cholesterol ‐ 15/51, 29%, chronic obstructive pulmonary disease ‐ 7/51, 14%, ischaemic heart disease ‐ 4/51, 8% *ASA 1 (n/N, %)*: 10/51, (19.60%) *ASA 2 (n/N, %)*: 28/51, (54.90%) *ASA 3 (n/N, %)*: 13/51, (25.49%) *ASA 4 (n/N, %)*: not reported *Number of participants randomised*: 60 *Number of participants receiving treatment*: 60 *Number of participants analysed*: 51 *Dropout rate*: 9/60, (15%) Overall *Age (years) (mean SD)*: not reported *Ethnicity*: not reported *Gender (males, females)*: not reported *Length of surgery (minutes)*: not reported *Proportion of participants on anticoagulants prior to surgery (n/N, %)*: not reported *Incidence of preoperative anaemia (n/N, %)*: not reported *Co‐morbidities (n/N, %)*: not reported *ASA 1 (n/N, %)*: not reported *ASA 2 (n/N, %)*: not reported *ASA 3 (n/N, %)*: not reported *ASA 4 (n/N, %)*: not reported *Number of participants randomised*: 150 *Number of participants receiving treatment*: 150 *Number of participants analysed*: not reported *Dropout rate*: not reported **Inclusion criteria:** people older than 18 years undergoing primary unilateral TKA were eligible to participate in the study **Exclusion criteria:** 1) history or risk of thrombosis, 2) active thromboembolic disease, 3) refused blood products, 4) known hypersensitivity to TXA or any of its ingredients, 5) complex haematologic disorders requiring manipulation, 6) pregnant and lactating women, 7) taking anticoagulant therapy within 5 days of surgery (warfarin, dabigatran, heparin, rivaroxaban), 8) severe renal failure (estimated glomerular filtration rate < 29) **If TKR, is tourniquet used:** yes **Indication for surgery:** not reported **Type of anaesthetic:** spinal **Type of surgery:** primary TKR	
Interventions	**Intervention characteristics** Placebo Intra‐articular: 20 mL of normal saline intra‐articularly after implantation of prosthesis and closure of arthrotomy followed by standard closure. Intravenous: administration of 20 mL of normal saline intravenously at the same time before release of tourniquet Placebo, IA, intraop (after implantation) + placebo, IV, intraop (before tourniquet deflation) TXA, IA Intra‐articular: 1.5 g TXA in 20 mL intra‐articularly after implantation of prosthesis and closure of arthrotomy followed by standard closure. Intravenous: administration of 20 mL of normal saline (in a 20 mL syringe) intravenously at the same time before release of tourniquet TXA, IA, 1.5 g, intraop (after implantation) + placebo, IV, intraop (before tourniquet deflation) TXA, IV Intra‐articular: 20 mL of normal saline intra‐articularly after implantation of prosthesis and closure of arthrotomy followed by standard closure. Intravenous: administration of 1.5 g TXA intravenously at the same time before release of tourniquet Placebo, IA, intraop (after implantation) + TXA, IV, 1.5 g, intraop (before tourniquet deflation)	
Outcomes	*Primary outcome:* Estimated perioperative blood loss *Secondary outcomes:* Functional measurements using patient self‐reported questionnaires (Short‐Form 12 survey and Oxford knee scores) Transfusion rates Median length of stay 30‐day readmissions Complications (symptomatic deep vein thrombosis (DVT), pulmonary embolism (PE) and infection) Range of motion (both passive and active, was measured as a surrogate for postoperative swelling)	
Notes	**Sponsorship source:** none **Country:** New Zealand **Setting:** multi‐centre **Comments:** none **Author's name:** MDJ Stowers **Institution:** Middlemore Hospital, Counties Manukau District Health Board (DHB) **Email:** not reported **Address:** Marinus DJ Stowers, MBChB, Department of Orthopaedics, Middlemore Hospital, Counties Manukau District Health Board (DHB), Hospital Rd, Auckland 1064, New Zealand **Native language of paper:** English **Reference type:** full text (1), trial registration (1) **Trial registration number:** NCT02278263 **Was it translated for this review:** no	
***Risk of bias***		
**Bias**	**Authors' judgement**	**Support for judgement**
Random sequence generation (selection bias)	Unclear risk	Quote: "Sequence generation was performed by an independent biostatistician (IZ). The sequence was then assigned to pretemplated group instructions for placebo, intra‐articular, and systemic groups with a ratio of 1:2:2, respectively. These were then block randomized into 3 groups and distributed to the 5 different centers." Judgement comment: insufficient evidence to permit judgement.
Allocation concealment (selection bias)	Unclear risk	Quote: "Prepackaged A4 envelopes with consent forms, questionnaires, and a postoperative record template were kept in a box in the preoperative areas. In the same A4 envelope, another smaller (letter‐sized) envelope could be found with the patient's group allocation and corresponding instructions. Upon recruitment and before the patient entering the operative theater, the smaller envelope containing the group allocation would then be revealed to the theater nursing staff aware of the study protocol." Judgement comment: does not mention sequentially numbered, opaque envelopes.
Blinding of participants and personnel (performance bias) Subjective outcomes	Low risk	Quote: "After assignment to interventions, patients, surgeons, and outcome assessors remained blinded to group allocations." Judgement comment: likely blinded due to central allocation of interventions and identical intervention regimens.
Blinding of participants and personnel (performance bias) Objective outcomes	Low risk	Quote: "After assignment to interventions, patients, surgeons, and outcome assessors remained blinded to group allocations." Judgement comment: objective outcome for personnel, so low risk of bias regardless of quality of blinding
Blinding of outcome assessment (detection bias) Subjective outcomes	Low risk	Quote: "After assignment to interventions, patients, surgeons, and outcome assessors remained blinded to group allocations." Judgement comment: likely blinding maintained.
Blinding of outcome assessment (detection bias) Objective outcomes	Low risk	Quote: "After assignment to interventions, patients, surgeons, and outcome assessors remained blinded to group allocations." Judgement comment: objective outcome for outcome assessor, so low risk of bias regardless of quality of blinding
Incomplete outcome data (attrition bias) All outcomes	Low risk	Quote: not applicable Judgement comment: all outcome data presented.
Selective reporting (reporting bias)	High risk	Quote: not applicable Judgement comment: pain via the numerical rating scale (secondary outcome) not reported. Complications: myocardial infarction/cerebrovascular accident (MI/CVA), infection (deep and superficial), manipulation under anaesthesia (MUA), urinary tract infection (UTI) (secondary outcomes) not reported. Oxford hip score and SF12 mentioned as secondary outcomes in the paper but not reported in the trial registration.
Other bias	High risk	Quote: "One or more of the authors of this paper have disclosed potential or pertinent conflicts of interest, which may include receipt of payment, either direct or indirect, institutional support, or association with an entity in the biomedical field which may be perceived to have potential conflict of interest with this work." Judgement Comment: Outcome time point measurements between the trial registration and full text do not match. Baseline imbalance with more females than males in the placebo group compared to other intervention groups. Potential conflict of interest also noted.

**Table:** Tanaka 2001

***Study characteristics***		
Methods	**Study design:** RCT **Intention‐to‐treat analysis:** not reported **Duration of study:** not reported **Power calculation reached:** not reported **Transfusion strategy:** in 1988, the National Institutes of Health Consensus Conference on perioperative transfusion suggested appropriate criteria and guidelines, which formed the basis for transfusion in our study. **Was the trial stopped early:** no	
Participants	**Baseline characteristics** Placebo *Age (years) (mean range)*: 65 (58 to 70) *Ethnicity*: not reported *Gender (males, females)*: 9/26 M (35%); 17/26 F (65%) *Length of surgery (minutes) (mean range)*: 125 (90 to 160) *Proportion of participants on anticoagulants prior to surgery (n/N, %)*: not reported *Incidence of preoperative anaemia (n/N, %)*: not reported *Co‐morbidities (n/N, %)*: not reported *ASA 1 (n/N, %)*: not reported *ASA 2 (n/N, %)*: not reported *ASA 3 (n/N, %)*: not reported *ASA 4 (n/N, %)*: not reported *Number of participants randomised*: not reported *Number of participants receiving treatment*: 26 *Number of participants analysed*: 26 *Dropout rate*: not reported Pre‐op TXA *Age (years) (mean range)*: 65 (59 to 70) *Ethnicity*: not reported *Gender (males, females)*: 7/24 M (29%); 17/24 F (71%) *Length of surgery (minutes) (mean range)*: 110 (80 to 150) *Proportion of participants on anticoagulants prior to surgery (n/N, %)*: not reported *Incidence of preoperative anaemia (n/N, %)*: not reported *Co‐morbidities (n/N, %)*: not reported *ASA 1 (n/N, %)*: not reported *ASA 2 (n/N, %)*: not reported *ASA 3 (n/N, %)*: not reported *ASA 4 (n/N, %)*: not reported *Number of participants randomised*: not reported *Number of participants receiving treatment*: 24 *Number of participants analysed*: 24 *Dropout rate*: not reported Intraop TXA *Age (years) (mean range)*: 65 (60 to 71) *Ethnicity*: not reported *Gender (males, females)*: 7/22 M (32%); 15/22 F (68%) *Length of surgery (minutes) (mean range)*: 120 (85 to 155) *Proportion of participants on anticoagulants prior to surgery (n/N, %)*: not reported *Incidence of preoperative anaemia (n/N, %)*: not reported *Co‐morbidities (n/N, %)*: not reported *ASA 1 (n/N, %)*: not reported *ASA 2 (n/N, %)*: not reported *ASA 3 (n/N, %)*: not reported *ASA 4 (n/N, %)*: not reported *Number of participants randomised*: not reported *Number of participants receiving treatment*: 22 *Number of participants analysed*: 22 *Dropout rate*: not reported Pre and intraop TXA *Age (years) (mean range)*: 65 (59 to 69) *Ethnicity*: not reported *Gender (males, females)* : 8/27 M (30%); 19/27 F (70%) *Length of surgery (minutes) (mean range)*: 110 (70 to 150) *Proportion of participants on anticoagulants prior to surgery (n/N, %)*: not reported *Incidence of preoperative anaemia (n/N, %)*: not reported *Co‐morbidities (n/N, %)*: not reported *ASA 1 (n/N, %)*: not reported *ASA 2 (n/N, %)*: not reported *ASA 3 (n/N, %)*: not reported *ASA 4 (n/N, %)*: not reported *Number of participants randomised*: not reported *Number of participants receiving treatment*: 27 *Number of participants analysed*: 27 *Dropout rate*: not reported Overall *Age (years) (mean range)*: not reported *Ethnicity*: not reported *Gender (males, females)*: not reported *Length of surgery (minutes) (mean range)*: not reported *Proportion of participants on anticoagulants prior to surgery (n/N, %)*: not reported *Incidence of preoperative anaemia (n/N, %)*: not reported *Co‐morbidities (n/N, %)*: not reported *ASA 1 (n/N, %)*: not reported *ASA 2 (n/N, %)*: not reported *ASA 3 (n/N, %)*: not reported *ASA 4 (n/N, %)*: not reported *Number of participants randomised*: not reported *Number of participants receiving treatment*: 99 *Number of participants analysed*: 99 *Dropout rate*: not reported **Inclusion criteria:** rheumatoid arthritis or osteoarthritis patients who were to have a unilateral bicondylar cemented TKA **Exclusion criteria:** 1) known allergy to drug, 2) preoperative hepatic or renal dysfunction, 3) serious cardiac or respiratory disease, 4) congenital or acquired coagulopathy, 5) history of thromboembolic disease **If TKR, is tourniquet used:** yes **Indication for surgery:** rheumatoid arthritis or osteoarthritis **Type of anaesthetic:** not reported **Type of surgery:** primary TKR	
Interventions	**Intervention characteristics** Placebo Pairs of ampoules, each containing 20 mL of either TNA (Rikavarin 100 mg/ml; Asahi Chemical Industry Co Ltd, Japan) or a placebo (physiological saline) were numbered and placed in envelopes at random by a pharmacologist. A slow intravenous injection of the contents of each ampoule, the constituents of which were known only to the pharmacologist, was given by the anaesthetist. The patients were given either: 1) saline twice, 10 minutes before surgery and on deflation of the tourniquet (control group) Placebo, saline IV, 20 mL, pre‐op + intraop (at tourniquet deflation) Pre‐op TXA Pairs of ampoules, each containing 20 mL of either TNA (Rikavarin 100 mg/ml; Asahi Chemical Industry Co Ltd, Japan) or a placebo (physiological saline) were numbered and placed in envelopes at random by a pharmacologist. A slow intravenous injection of the contents of each ampoule, the constituents of which were known only to the pharmacologist, was given by the anaesthetist. The patients were given either: 2) 20 mg/kg of TXA 10 minutes before surgery and saline 10 minutes before deflation of the tourniquet (preop TXA group) TXA, IV, 20 mg/kg, preop + placebo, IV, 20 mL, intraop (at tourniquet deflation) Intraop TXA Pairs of ampoules, each containing 20 mL of either TNA (Rikavarin 100 mg/ml; Asahi Chemical Industry Co Ltd, Japan) or a placebo (physiological saline) were numbered and placed in envelopes at random by a pharmacologist. A slow intravenous injection of the contents of each ampoule, the constituents of which were known only to the pharmacologist, was given by the anaesthetist. The patients were given either: 3) saline 10 minutes before surgery and 20 mg/kg of TXA 10 minutes before deflation of the tourniquet (intraop TXA group) Placebo, saline IV, 20 mL, pre‐op + TXA, IV, 20 mg/kg, intraop (at tourniquet deflation) Pre and intraop TXA Pairs of ampoules, each containing 20 mL of either TNA (Rikavarin 100 mg/ml; Asahi Chemical Industry Co Ltd, Japan) or a placebo (physiological saline) were numbered and placed in envelopes at random by a pharmacologist. A slow intravenous injection of the contents of each ampoule, the constituents of which were known only to the pharmacologist, was given by the anaesthetist. The patients were given either: 4) 10 mg/kg of TXA 10 minutes before surgery and again 10 minutes before deflation of the tourniquet (pre‐ and intraop TXA group). TXA, IV, 10 mg/kg, preop + TXA, IV, 10 mg/kg, intraop (at tourniquet deflation)	
Outcomes	*Primary outcomes:* Blood loss Adverse events (thromboembolic and other complications) *Secondary outcome:* Not reported	
Notes	**Sponsorship source:** none **Country:** Japan **Setting:** single‐centre **Comments:** none **Author's name:** N Tanaka **Institution:** Sapporo Gorinbashi Orthopaedic Hospital **Email:** not reported **Address:** Sapporo Gorinbashi Orthopaedic Hospital, Gorinbashi Health Care Facilities and Hospitals, 2‐1 Kawazoe, Minami‐ku, Sapporo, Hokkaido 005‐0802, Japan **Native language of paper:** English **Reference type:** full text (1) **Trial registration number:** not applicable, but when checked, trial registration number not found **Was it translated for this review:** no	
***Risk of bias***		
**Bias**	**Authors' judgement**	**Support for judgement**
Random sequence generation (selection bias)	Unclear risk	Quote: "TNA (Rikavarin 100 mg/ml; Asahi Chemical Industry Co Ltd, Japan) or a placebo (physiological saline) were numbered and placed in envelopes at random by a pharmacologist." Judgement comment: insufficient information about the sequence generation process to permit judgement
Allocation concealment (selection bias)	Unclear risk	Quote: "Pairs of ampoules, each containing 20 ml of either TNA (Rikavarin 100 mg/ml; Asahi Chemical Industry Co Ltd, Japan) or a placebo (physiological saline) were numbered and placed in envelopes at random by a pharmacologist." Judgement comment: insufficient information about the sequence generation process and whether envelopes were sealed, opaque and sequentially numbered.
Blinding of participants and personnel (performance bias) Subjective outcomes	Low risk	Quote: "A slow intravenous injection of the contents of each ampoule, the constituents of which were known only to the pharmacologist, was given by the anaesthetist." Judgement comment: unlikely blinding could have been broken.
Blinding of participants and personnel (performance bias) Objective outcomes	Low risk	Quote: not applicable Judgement comment: objective outcome for personnel and participants, so low risk of bias regardless of quality of blinding.
Blinding of outcome assessment (detection bias) Subjective outcomes	Unclear risk	Quote: not applicable Judgement comment: insufficient information to permit judgement.
Blinding of outcome assessment (detection bias) Objective outcomes	Low risk	Quote: not applicable Judgement comment: objective outcome for outcome assessor, so low risk of bias regardless of quality of blinding.
Incomplete outcome data (attrition bias) All outcomes	Unclear risk	Quote: not applicable Judgement comment: insufficient reporting of attrition/exclusions to permit judgement.
Selective reporting (reporting bias)	Unclear risk	Quote: not applicable Judgement comment: insufficient information to permit judgement. No available prospective protocol or trial registration.
Other bias	Unclear risk	Quote: not applicable Judgement comment: lack of information about participants randomised and dropout rates. Unclear information on funding source.

**Table:** Tsukada 2019

***Study characteristics***		
Methods	**Study design:** RCT **Intention‐to‐treat analysis:** yes **Duration of study:** 23 months + 3 months follow‐up **Power calculation reached:** yes **Transfusion strategy:** planned additional allogenic blood transfusion for patients with a haemoglobin level of < 7.0 g/dL who were asymptomatic and those with a haemoglobin level of < 10.0 g/dL who had symptoms related to anaemia **Was the trial stopped early:** no	
Participants	**Baseline characteristics** TXA, IV + placebo, IA *Age (years) (mean SD)*: 77 (6) *Ethnicity*: not reported *Gender (males, females)*: 6/34 M (18%); 28/34 F (82%) *Length of surgery (minutes) (mean SD)*: not reported *Proportion of participants on anticoagulants prior to surgery (n, %)*: 5, 14.7% *Incidence of preoperative anaemia (n/N, %)*: not reported *Co‐morbidities (n/N)*: 6/28 *ASA 1 (n/N, %)*: not reported *ASA 2 (n/N, %)*: not reported *ASA 3 (n/N, %)*: not reported *ASA 4 (n/N, %)*: not reported *Number of participants randomised*: 34 *Number of participants receiving treatment*: 34 *Number of participants analysed*: 34 *Drop out rate*: 0/34, 0% TXA, IV + TXA, IA *Age (years) (mean SD)*: 75 (6) *Ethnicity*: not reported *Gender (males, females)*: 10/43 M (23%); 33/43 F (77%) *Length of surgery (minutes) (mean SD)*: not reported *Proportion of participants on anticoagulants prior to surgery (n/N, %)*: 8, 16.6% *Incidence of preoperative anaemia (n/N, %)*: not reported *Co‐morbidities (n/N)*: 9/34 *ASA 1 (n/N, %)*: not reported *ASA 2 (n/N, %)*: not reported *ASA 3 (n/N, %)*: not reported *ASA 4 (n/N, %)*: not reported *Number of participants randomised*: 43 *Number of participants receiving treatment*: 43 *Number of participants analysed*: 43 *Drop out rate*: 0/43, 0% Overall *Age (years) (mean SD)*: not reported *Ethnicity*: not reported *Gender (males, females)*: not reported *Length of surgery (minutes) (mean SD)*: not reported *Proportion of participants on anticoagulants prior to surgery (n/N, %)*: not reported *Incidence of preoperative anaemia (n/N, %)*: not reported *Co‐morbidities (n/N)*: not reported *ASA 1 (n/N, %)*: not reported *ASA 2 (n/N, %)*: not reported *ASA 3 (n/N, %)*: not reported *ASA 4 (n/N, %)*: not reported *Number of participants randomised*: 77 *Number of participants receiving treatment*: 77 *Number of participants analysed*: 77 *Drop out rate*: 0/77, 0% **Inclusion criteria:** 1) 20 years of age or older 2) medically fit for simultaneous bilateral TKA **Exclusion criteria:** 1) known allergic reaction to TXA, 2) patients with pre‐operative haemoglobin level under 11.0 g/dL, 3) patients who refused blood products, 4) patients who were enrolled in another interventional clinical trial within 6 months prior to surgery **If TKR, is tourniquet used:** no **Indication for surgery:** osteoarthritis **Type of anaesthetic:** general **Type of surgery:** simultaneous bilateral TKA	
Interventions	**Intervention characteristics** TXA, IV + placebo, IA In the intravenous TXA group, 1000 mg of TXA was similarly administered intravenously just before the skin incision in the first knee. After closing the capsule and retinaculum, 10 mL of normal saline was administered intra‐articularly into each knee. For the patients allocated to the intravenous TXA group, 1000 mg of TXA was administered in the operating theatre. Six hours later, another 1000 mg of TXA was administered intravenously TXA, IV, 1 g, pre‐incision and placebo, IA, intraop both knees and TXA, IV, 1 g, intraop and TXA, IV, 1 g, 6 hours postop TXA, IV + TXA, IA Combined TXA group, patients received 1000 mg of TXA intravenously (Transamin; Daiichi‐Sankyo,Tokyo, Japan) just before the skin incision in the first knee. After implantation of the prosthesis, we closed the capsule and retinaculum. Then, we injected 1000 mg of TXA (10 mL of 100 mg/mL TXA) intra‐articularly into each knee through the medial patellar retinaculum using a 23‐gauge needle. Thus, a total of 3000 mg of TXA was administered in the operating theatre for patients allocated to the combined TXA group. In the ward, another 1000 mg of TXA was given intravenously 6 h after the initial intravenous administration TXA, IV, 1 g, pre‐incision and TXA, IA, 1 g, intraop both knees and TXA, IV, 1 g, 6 hours postop	
Outcomes	*Primary outcomes:* Perioperative blood loss calculated Change in haemoglobin from preoperative to postoperative day 3 *Secondary outcomes:* Calculated blood loss at 7 days after TKA Number of patients requiring allogeneic blood transfusion Major bleeding Thrombotic events up to 3 months after TKA	
Notes	**Sponsorship source:** non‐pharmaceutical funding **Country:** Japan **Setting:** single‐centre **Comments:** paper says patients were allowed to continue anticoagulant tablets, but these data are not given **Author's name:** S Tsukada **Institution:** Hokusuikai Kinen Hospital **Email:** s8058@nms.ac.jp **Address:** Department of Orthopaedic Surgery, Hokusuikai Kinen Hospital, 3‐2‐1 Higashihara, Mito, Ibaraki 310‐0035, Japan **Native language of paper:** English **Reference type:** full text (1), trial registration (1) **Trial registration number:** UMIN000026137 **Was it translated for this review:** no	
***Risk of bias***		
**Bias**	**Authors' judgement**	**Support for judgement**
Random sequence generation (selection bias)	Low risk	Quote: "We generated a sequence of random numbers from 0 to 99 using computer software (R; The R Foundation for Statistical Computing, Vienna, Austria)." Judgement comment: computer‐generated randomisation.
Allocation concealment (selection bias)	Unclear risk	Quote: "We prepared sufficient number of opaque envelopes into which these randomized numbers were placed. A sealed envelope was selected just after starting TKA of the first knee by the allocating staff who were not otherwise involved in the trial." Judgement comment: use of sealed, opaque envelopes. Unclear whether these envelopes were sequentially numbered.
Blinding of participants and personnel (performance bias) Subjective outcomes	Low risk	Quote: "We maintained high adherence to the study protocol and successful blinding of the patients, surgical teams, and outcome assessors and successful randomization and patient allocation concealment" Judgement comment: none
Blinding of participants and personnel (performance bias) Objective outcomes	Low risk	Quote: not applicable Judgement comment: objective outcome for personnel, so low risk of bias regardless of quality of blinding.
Blinding of outcome assessment (detection bias) Subjective outcomes	Unclear risk	Quote: not applicable Judgement comment: insufficient information to permit judgement.
Blinding of outcome assessment (detection bias) Objective outcomes	Low risk	Quote: not applicable Judgement comment: objective outcome for outcome assessors, so low risk of bias regardless of quality of blinding.
Incomplete outcome data (attrition bias) All outcomes	Low risk	Quote: not applicable Judgement comment: no obvious outcome data missing.
Selective reporting (reporting bias)	Unclear risk	Quote: not applicable Judgement comment: combined and intravenous group regimen in the protocol different to that reported in the text. No pre‐specified outcomes reported in the trial registration, so unclear whether all outcomes reported.
Other bias	Low risk	Quote: not applicable Judgement comment: no other concerns such as early stopping or imbalanced study arms.

**Table:** Tsukada 2020

***Study characteristics***		
Methods	**Study design:** RCT **Intention‐to‐treat analysis:** yes **Duration of study:** 13 months + 3 months (follow‐up) **Power calculation reached:** yes **Transfusion strategy:** used a standardised allogeneic transfusion protocol whereby red blood cells were transfused if the haemoglobin concentration was < 7.0 g/dL or if it was < 10.0 g/dL and associated with symptoms related to anaemia **Was the trial stopped early:** no	
Participants	**Baseline characteristics** TXA, IV and IA + postop placebo *Age (years) (mean SD)*: 75 (6) *Ethnicity*: not reported *Gender (males, females)*: 14/54 M (26%); 40/54 F (74%) *Length of surgery (minutes) (mean SD)*: 96 (15) *Proportion of participants on anticoagulants prior to surgery (n/N, %)*: 12, 28% *Incidence of preoperative anaemia (n/N, %)*: not reported *Co‐morbidities (n/N, %)*: diabetes 10/44 *ASA 1 (n/N, %)*: not reported *ASA 2 (n/N, %)*: not reported *ASA 3 (n/N, %)*: not reported *ASA 4 (n/N, %)*: not reported *Number of participants randomised*: 54 *Number of participants receiving treatment*: 54 *Number of participants analysed*: 54 *Dropout rate*: 0/54, 0% TXA, IV and IA + postop TXA, IV *Age (years) (mean SD)*: 75 (7) *Ethnicity*: not reported *Gender (males, females)*: 9/46 M (20%); 37/46 F (80%) *Length of surgery (minutes) (mean SD)*: 102 (16) *Proportion of participants on anticoagulants prior to surgery (n/N, %)*: 9, 24% *Incidence of preoperative anaemia (n/N, %)*: not reported *Co‐morbidities (n/N, %)*: diabetes 7/39 *ASA 1 (n/N, %)*: not reported *ASA 2 (n/N, %)*: not reported *ASA 3 (n/N, %)*: not reported *ASA 4 (n/N, %)*: not reported *Number of participants randomised*: 46 *Number of participants receiving treatment*: 46 *Number of participants analysed*: 46 *Dropout rate*: 0/46, 0% Overall *Age (years) (mean SD)*: not reported *Ethnicity*: not reported *Gender (males, females)*: not reported *Length of surgery (minutes) (mean SD)*: not reported *Proportion of participants on anticoagulants prior to surgery (n/N, %)*: not reported *Incidence of preoperative anaemia (n/N, %)*: not reported *Co‐morbidities (n/N, %)*: not reported *ASA 1 (n/N, %)*: not reported *ASA 2 (n/N, %)*: not reported *ASA 3 (n/N, %)*: not reported *ASA 4 (n/N, %)*: not reported *Number of participants randomised*: not reported *Number of participants receiving treatment*: not reported *Number of participants analysed*: not reported *Dropout rate*: not reported **Inclusion criteria:** 1) > 20 years of age, 2) undergoing primary unilateral TKA, 3) medically fit for an operation, and suitable for TKA **Exclusion criteria:** 1) known allergic reaction to TXA **If TKR, is tourniquet used:** no **Indication for surgery:** osteoarthritis of the knee, rheumatoid arthritis, avascular necrosis **Type of anaesthetic:** general **Type of surgery:** primary TKR	
Interventions	**Intervention characteristics** TXA, IV and IA + postop placebo In the placebo group, intravenous normal saline solution (100 mL) was administered 6 hours after the first intravenous administration of TXA and at 8:00 AM and 8:00 PM 1 day after TKA. TXA, IV, 1 g, pre incision and TXA IA 1 g, intraop and placebo IV, 6 hours, 12 hours and 24 hours TXA, IV and IA + postop TXA, IV In both groups, patients received intravenous TXA (1000 mg/100 mL) (Transamin; Daiichi Sankyo) just before skin incision. After implantation of the prosthesis and arthrotomy closure, we injected 1000 mg of TXA into the knee joint with use of a 23‐gauge needle that penetrated the medial patellar retinaculum and knee capsule in the TXA group, patients received intravenous TXA (1000 mg/100 mL) 6 hours after the first intravenous administration of TXA and at 8:00 AM and 8:00 PM 1 day after TKA. TXA, IV, 1 g, pre‐incision and TXA IA 1 g, intraop and TXA IV 1 g, 6 hours, 12 hours and 24 hours	
Outcomes	*Primary outcomes:* Volume of perioperative blood loss Change in haemoglobin from preoperatively to 3 days postoperatively *Secondary outcomes:* Volume of perioperative blood loss at 7 days after TKA Range of knee motion Complications Major bleeding Thrombotic events Volume of perioperative blood loss at 1 day after TKA Number of patients requiring allogeneic blood transfusion	
Notes	**Sponsorship source:** no external funding **Country:** Japan **Setting:** single‐centre **Comments:** patients included taking anticoagulants **Author's name:** S Tsukada **Institution:** Hokusuikai Kinen Hospital **Email:** s8058@nms.ac.jp **Address:** Department of Orthopaedic Surgery, Hokusuikai Kinen Hospital, Mito, Japan **Native language of paper:** English **Reference type:** full text (1), trial registration (1) **Trial registration number:** UMIN000030237 **Was it translated for this review:** no	
***Risk of bias***		
**Bias**	**Authors' judgement**	**Support for judgement**
Random sequence generation (selection bias)	Low risk	Quote: "We employed simple random allocation in which treatment allocation was made without regard to prior allocation. A sequence of random digits from 0 to 99 was generated, allowing repeated numbers, with use of computer software (R; R Foundation for Statistical Computing)." Judgement comment: use of computer‐generated random number sequence.
Allocation concealment (selection bias)	Low risk	Quote: "The trial medication was packed and blinded by the Department of Pharmacology. The study drugs were identical in appearance because of the transparent nature of the TXA solution." Judgement comment: adequate allocation concealment as using central allocation (including telephone, web‐based and pharmacy‐controlled randomisation).
Blinding of participants and personnel (performance bias) Subjective outcomes	Low risk	Quote: "Patients remained blinded to treatment allocation until data analyses were completed." Judgement comment: low risk of bias due to adequate personnel blinding.
Blinding of participants and personnel (performance bias) Objective outcomes	Low risk	Quote: not applicable Judgement comment: objective outcome for personnel, so low risk of bias regardless of quality of blinding.
Blinding of outcome assessment (detection bias) Subjective outcomes	Unclear risk	Quote: not applicable Judgement comment: no information reported on blinding of anyone other than participants.
Blinding of outcome assessment (detection bias) Objective outcomes	Low risk	Quote: not applicable Judgement comment: objective outcome for outcome assessors, so low risk of bias regardless of quality of blinding.
Incomplete outcome data (attrition bias) All outcomes	Low risk	Quote: not applicable Judgement comment: n obvious outcome data missing.
Selective reporting (reporting bias)	Low risk	Quote: not applicable Judgement comment: all outcomes planned in the protocol or prospective trial registration are reported.
Other bias	Low risk	Quote: not applicable Judgement comment: no other concerns such as early stopping or imbalanced study arms.

**Table:** Utada 1997

***Study characteristics***		
Methods	**Study design:** RCT **Intention‐to‐treat analysis:** yes **Duration of study:** 18 months **Power calculation reached:** not reported **Transfusion strategy:** not reported **Was the trial stopped early:** no	
Participants	**Baseline characteristics** Placebo *Age (years) (mean SD)*: 64 (5) *Ethnicity*: not reported *Gender (males, females)*: 2/10 M (20%); 8/10 F (80%) *Length of surgery (minutes)*: 88 (29) *Proportion of participants on anticoagulants prior to surgery (n/N, %)*: not reported *Incidence of preoperative anaemia (n/N, %)*: not reported *Co‐morbidities (n/N, %)*: not reported *ASA 1 (n/N, %)*: not reported *ASA 2 (n/N, %)*: not reported *ASA 3 (n/N, %)*: not reported *ASA 4 (n/N, %)*: not reported *Number of participants randomised*: 10 *Number of participants receiving treatment*: 10 *Number of participants analysed*: 10 *Dropout rate*: 0/10 (0%) Aprotinin *Age (years) (mean SD)*: 63 (11) *Ethnicity*: not reported *Gender (males, females)*: 1/11 M (9%); 10/11 F (91%) *Length of surgery (minutes)*: 80 (28) *Proportion of participants on anticoagulants prior to surgery (n/N, %)*: not reported *Incidence of preoperative anaemia (n/N, %)*: not reported *Co‐morbidities (n/N, %)*: not reported *ASA 1 (n/N, %)*: not reported *ASA 2 (n/N, %)*: not reported *ASA 3 (n/N, %)*: not reported *ASA 4 (n/N, %)*: not reported *Number of participants randomised*: 11 *Number of participants receiving treatment*: 11 *Number of participants analysed*: 11 *Dropout rate*: 0/11 (0%) Overall *Age (years) (mean SD)*: not reported *Ethnicity*: not reported *Gender (males, females)*: not reported *Length of surgery (minutes)*: not reported *Proportion of participants on anticoagulants prior to surgery (n/N, %)*: not reported *Incidence of preoperative anaemia (n/N, %)*: not reported *Co‐morbidities (n/N, %)*: not reported *ASA 1 (n/N, %)*: not reported *ASA 2 (n/N, %)*: not reported *ASA 3 (n/N, %)*: not reported *ASA 4 (n/N, %)*: not reported *Number of participants randomised*: 21 *Number of participants receiving treatment*: not reported *Number of participants analysed*: not reported *Dropout rate*: not reported **Inclusion criteria:** 1) undergoing primary total hip replacement, 2) ASA classification I or II **Exclusion criteria:** 1) history of hip replacement surgery, 2) for whom bone cements were used **If TKR, is tourniquet used:** not applicable **Indication for surgery:** not reported **Type of anaesthetic:** combined spinal and general anaesthetic **Type of surgery:** primary THR	
Interventions	**Intervention characteristics** Placebo After inducing anaesthesia, we started administration of ready‐to‐use third‐party aprotinin or saline 200 mL. In the first 30 minutes, 100 mL was intravenously infused and, thereafter, the infusion speed was at 50 mL/hr until the end of surgery to complete administration of 200 mL in total (aprotinin 1 mL = 10,000 KIU). Placebo, IV, intraop, bolus, + fast intraop infusion (30 mins) + slow intraop infusion until surgery end Aprotinin After inducing anaesthesia, we started administration of ready‐to‐use third‐party aprotinin or saline 200 mL. In the first 30 minutes, 100 mL was intravenously infused and, thereafter, the infusion speed was at 50 mL/hr until the end of surgery to complete administration of 200 mL in total (aprotinin 1 mL = 10,000 KIU). Aprotinin, IV, 2 x 10^6 KIU, intraop, bolus, + fast intraop infusion (30 mins) 1 x 10^6 KIU + slow intraop infusion 0.5 x 10^6 KIU until surgery end	
Outcomes	*Primary outcome:* Blood loss *Secondary outcome:* Not reported	
Notes	**Sponsorship source:** not reported **Country:** Japan **Setting:** single‐centre **Comments:** results reported mean and SD of transfusions intraop and postop but not total, therefore calculated combined means as per Cochrane Handbook section 7.7.3.8 **Author's name:** K Utada **Institution:** Yamaguchi Prefectural Central Hospital **Email:** not reported **Address:** Department of Anesthesia, Yamaguchi Prefectural Central Hospital, Hofu, 747 **Native language of paper:** Japanese **Reference type:** full text (1) **Trial registration number:** not applicable, but when checked, trial registration number not found **Was it translated for this review:** yes	
***Risk of bias***		
**Bias**	**Authors' judgement**	**Support for judgement**
Random sequence generation (selection bias)	Unclear risk	Quote: "The 21 patients were randomly assigned to two groups" Judgement comment: insufficient information to permit judgement.
Allocation concealment (selection bias)	Unclear risk	Quote: "The 21 patients were randomly assigned to two groups" Judgement comment: insufficient information to permit judgement.
Blinding of participants and personnel (performance bias) Subjective outcomes	Unclear risk	Quote: not applicable Judgement comment: no description of personnel blinding given. Insufficient information to permit judgement.
Blinding of participants and personnel (performance bias) Objective outcomes	Low risk	Quote: not applicable Judgement Comment: Objective outcome for personnel, so low risk of bias regardless of quality of blinding.
Blinding of outcome assessment (detection bias) Subjective outcomes	Unclear risk	Quote: not applicable Judgement comment: no description of outcome assessor blinding given. Insufficient information to permit judgement.
Blinding of outcome assessment (detection bias) Objective outcomes	Low risk	Quote: not applicable Judgement comment: objective outcome for outcome assessor, so low risk of bias regardless of quality of blinding.
Incomplete outcome data (attrition bias) All outcomes	Low risk	Quote: not applicable Judgement comment: all outcome data presented.
Selective reporting (reporting bias)	Unclear risk	Quote: not applicable Judgement comment: no protocol available to check pre‐specified outcome measures reported.
Other bias	Unclear risk	Quote: not applicable Judgement comment: no funding source declared.

**Table:** Veien 2002

***Study characteristics***		
Methods	**Study design:** RCT **Intention‐to‐treat analysis:** yes **Duration of study:** not reported **Power calculation reached:** yes **Transfusion strategy:** blood transfusion was indicated when the haematocrit value was less than 28% **Was the trial stopped early:** no	
Participants	**Baseline characteristics** Placebo *Age (years) (mean SD)*: 69.5 (9.0) *Ethnicity*: not reported *Gender (males, females)*: 1/15 M (7%); 14/15 F (93%) *Length of surgery (minutes) (mean SD)*: 70.4 (21.8) *Proportion of participants on anticoagulants prior to surgery (n/N, %)*: 0/15, 0% *Incidence of preoperative anaemia (n/N, %)*: not reported *Co‐morbidities (n/N, %)*: not reported *ASA 1 (n/N, %)*: not reported *ASA 2 (n/N, %)*: not reported *ASA 3 (n/N, %)*: not reported *ASA 4 (n/N, %)*: not reported *Number of participants randomised*: 15 *Number of participants receiving treatment*: 15 *Number of participants analysed*: 15 *Dropout rate*: 0/15 (0%) TXA *Age (years) (mean SD)*: 70.5 (9.5) *Ethnicity*: not reported *Gender (males, females)*: 4/15 M (27%); 11/15 F (73%) *Length of surgery (minutes) (mean SD)*: 71.6 (10.4) *Proportion of participants on anticoagulants prior to surgery (n/N, %)*: 0/15, 0% *Incidence of preoperative anaemia (n/N, %)*: not reported *Co‐morbidities (n/N, %)*: not reported *ASA 1 (n/N, %)*: not reported *ASA 2 (n/N, %)*: not reported *ASA 3 (n/N, %)*: not reported *ASA 4 (n/N, %)*: not reported *Number of participants randomised*: 15 *Number of participants receiving treatment*: 15 *Number of participants analysed*: 15 *Dropout rate*: 0/15 (0%) Overall *Age (years) (mean SD)*: not reported *Ethnicity*: not reported *Gender (males, females)*: not reported *Length of surgery (minutes) (mean SD)*: not reported *Proportion of participants on anticoagulants prior to surgery (n/N, %)*: not reported *Incidence of preoperative anaemia (n/N, %)*: not reported *Co‐morbidities (n/N, %)*: not reported *ASA 1 (n/N, %)*: not reported *ASA 2 (n/N, %)*: not reported *ASA 3 (n/N, %)*: not reported *ASA 4 (n/N, %)*: not reported *Number of participants randomised*: 30 *Number of participants receiving treatment*: 30 *Number of participants analysed*: 30 *Dropout rate*: 0/0 (0%) **Inclusion criteria:** 1) patients scheduled for primary cemented TKR surgery **Exclusion criteria:** 1) patient aged less than 18 years, 2) recent myocardial infarction (< 6 months), 3) unstable angina, 4) severe aortic or mitral valve stenosis, 5) previous stroke, 6) unmedicated hypertension, 7) history of thromboembolic episodes, 8) bleeding disorders or warfarin medication **If TKR, is tourniquet used:** yes **Indication for surgery:** not reported **Type of anaesthetic:** spinal anaesthetic **Type of surgery:** primary TKR	
Interventions	**Intervention characteristics** Placebo Randomised according to a computer‐generated randomisation table for either TXA or not TXA (non‐TXA) (no other information given about this arm) ASSUMED not reported: placebo, IV, intraop prior to tourniquet release AND placebo, IV, 3 hours after 1st dose TXA The TXA dose of 10 mg/kg body weight was given just before the release of the tourniquet, and the same dose was again given 3 h later in the recovery room, although a maximum of 1 g was given each time. TXA, IV, 10 mg/kg, intraop prior to tourniquet release AND TXA, IV, 10 mg/kg, 3 hours after 1st dose (max 1g each time)	
Outcomes	*Primary outcomes:* Blood loss Blood transfusions Complications (intra‐articular haematoma, thromboembolic episodes) *Secondary outcome:* Not reported	
Notes	**Sponsorship source:** not reported **Country:** Denmark **Setting:** not reported **Comments:** none **Author's name:** M Veien **Institution:** Aarhus University Hospital **Email:** mveien@dadlnet.dk **Address:** Department of Anesthesiology, Aarhus Amtssygehus, Aarhus University Hospital, Aarhus, Denmark **Native language of paper:** English **Reference type:** full text (1) **Trial registration number:** not applicable, but when checked, trial registration number not found **Was it translated for this review:** no	
***Risk of bias***		
**Bias**	**Authors' judgement**	**Support for judgement**
Random sequence generation (selection bias)	Low risk	Quote: "randomized according to a computer‐ generated randomization table for either TXA or not TXA (non‐TXA)." Judgement comment: computer‐generated randomisation.
Allocation concealment (selection bias)	Unclear risk	Quote: not applicable Judgement comment: insufficient information to permit judgement.
Blinding of participants and personnel (performance bias) Subjective outcomes	Low risk	Quote: "Only the investigators were aware of the randomization and they administrated the drug. The patient and the nurses in the PACU and on the wards were unaware of the randomization and all registrations were performed by these nurses." Judgement comment: blinding of personnel likely ensured.
Blinding of participants and personnel (performance bias) Objective outcomes	Low risk	Quote: not applicable Judgement comment: objective outcome for personnel, so low risk of bias regardless of quality of blinding.
Blinding of outcome assessment (detection bias) Subjective outcomes	High risk	Quote: "Only the investigators were aware of the randomization and they administrated the drug. The patient and the nurses in the PACU and on the wards were unaware of the randomization and all registrations were performed by these nurses." Judgement comment: investigators not blinded.
Blinding of outcome assessment (detection bias) Objective outcomes	Low risk	Quote: not applicable Judgement comment: objective outcome for outcome assessor, so low risk of bias regardless of quality of blinding
Incomplete outcome data (attrition bias) All outcomes	Low risk	Quote: not applicable Judgement comment: all outcome data were presented.
Selective reporting (reporting bias)	Unclear risk	Quote: not applicable Judgement comment: no study protocol available to check pre‐specified outcomes reported.
Other bias	Unclear risk	Quote: not applicable Judgement comment: the placebo treatment arm regimen was not described.

**Table:** Veien 2005

***Study characteristics***		
Methods	**Study design:** RCT **Intention‐to‐treat analysis:** unclear **Duration of study:** not reported **Power calculation reached:** not reported **Transfusion strategy:** not reported **Was the trial stopped early:** no	
Participants	**Baseline characteristics** TXA, IV, repeated dose *Age (years) (mean SD)*: 70.1 (10) *Ethnicity*: not reported *Gender (males, females)*: 6/17 M (35%); 11/17 F (65%) *Length of surgery (minutes)*: 65 (17.3) *Proportion of participants on anticoagulants prior to surgery (n/N, %)*: 0/17, (0%) *Incidence of preoperative anaemia (n/N, %)*: not reported *Co‐morbidities (n/N, %)*: not reported *ASA 1 (n/N, %)*: not reported *ASA 2 (n/N, %)*: not reported *ASA 3 (n/N, %)*: not reported *ASA 4 (n/N, %)*: not reported *Number of participants randomised*: not reported *Number of participants receiving treatment*: not reported *Number of participants analysed*: 17 *Dropout rate*: not reported TXA, IV, single dose *Age (years) (mean SD)*: 67.8 (7.3) *Ethnicity*: not reported *Gender (males, females)*: 8/14 M (57%); 6/14 F (43%) *Length of surgery (minutes)*: 62.3 (11.7) *Proportion of participants on anticoagulants prior to surgery (n/N, %)*: 0/14, (0%) *Incidence of preoperative anaemia (n/N, %)*: not reported *Co‐morbidities (n/N, %)*: not reported *ASA 1 (n/N, %)*: not reported *ASA 2 (n/N, %)*: not reported *ASA 3 (n/N, %)*: not reported *ASA 4 (n/N, %)*: not reported *Number of participants randomised*: not reported *Number of participants receiving treatment*: not reported *Number of participants analysed*: 14 *Dropout rate*: not reported Overall *Age (years) (mean SD)*: not reported *Ethnicity*: not reported *Gender (males, females)*: not reported *Length of surgery (minutes)*: not reported *Proportion of participants on anticoagulants prior to surgery (n/N, %)*: not reported *Incidence of preoperative anaemia (n/N, %)*: not reported *Co‐morbidities (n/N, %)*: not reported *ASA 1 (n/N, %)*: not reported *ASA 2 (n/N, %)*: not reported *ASA 3 (n/N, %)*: not reported *ASA 4 (n/N, %)*: not reported *Number of participants randomised*: not reported *Number of participants receiving treatment*: not reported *Number of participants analysed*: not reported *Drop out rate*: not reported **Inclusion criteria:** 1) ≥ 18 years of age, 2) able to be anaesthetised regionally, according to local guidelines, 3) haematocrit value of > 0.3, 4) scheduled to have total knee replacement (TKR) surgery **Exclusion criteria:** 1) prior cardiac infarction (< 6 months), 2) unstable angina pectoris, 3) severe aortic‐ or mitral stenosis, 4) former vascular disease in the CNS (stroke, haemorrhage, TCI), 5) Untreated hypertension (diastolic pressure > 105 mmHg, systolic > 10 mmHg), 6) former thromboembolic episodes, 7) haematologic disease, 8) active anticoagulant treatment, 9) intake of acetylsalicylic acid or NSAID 2 weeks prior to surgery **If TKR, is tourniquet used:** no **Indication for surgery:** not reported **Type of anaesthetic:** spinal anaesthesia **Type of surgery:** primary TKR	
Interventions	**Intervention characteristics** TXA, IV, repeated dose Group A: two doses of TXA (10 mg/kg body weight) with 3‐hour intervals TXA, IV, 10 mg/kg, repeated dose (time point unclear) TXA, IV, single dose Group B: one dose (10 mg/kg body weight) TXA, IV, 10 mg/kg, (time point unclear)	
Outcomes	*Primary outcomes:* Blood loss Transfusions Episodes of nausea Intra‐articular haematomas Post‐surgery thromboembolic episodes	
Notes	**Sponsorship source:** not reported **Country:** Denmark **Setting:** not reported **Comments:** further information on treatment arms provided by author **Author's name:** M Veien **Institution:** Arhus Universitetshospital **Email:** mveien@dadlnet.dk **Address:** Anaestesiologisk, Arhus Universitetshospital Afdeling, Arhus Sygehus, DK‐8000 Arhus **Native language of paper:** Danish **Reference type:** full text (1) **Trial registration number:** not applicable, but when checked, trial registration number not found **Was it translated for this review:** yes	
***Risk of bias***		
**Bias**	**Authors' judgement**	**Support for judgement**
Random sequence generation (selection bias)	Low risk	Quote: not applicable Judgement comment: randomised through a computer program.
Allocation concealment (selection bias)	Unclear risk	Quote: not applicable Judgement comment: insufficient information to permit judgement.
Blinding of participants and personnel (performance bias) Subjective outcomes	High risk	Quote: "Only the physician who gave the medicine (TXA) to the patients knew about the randomisation process." Judgement comment: blinding of key personnel not ensured.
Blinding of participants and personnel (performance bias) Objective outcomes	Low risk	Quote: not applicable Judgement comment: objective outcome for personnel, so low risk of bias regardless of quality of blinding.
Blinding of outcome assessment (detection bias) Subjective outcomes	Unclear risk	Quote: not applicable Judgement comment: no information on outcome assessor blinding.
Blinding of outcome assessment (detection bias) Objective outcomes	Low risk	Quote: not applicable Judgement comment: objective outcome for outcome assessor, so low risk of bias regardless of quality of blinding.
Incomplete outcome data (attrition bias) All outcomes	Low risk	Quote: not applicable Judgement comment: no missing outcome data reported.
Selective reporting (reporting bias)	Unclear risk	Quote: not applicable Judgement comment: no protocol to check pre‐specified outcome measures reported.
Other bias	High risk	Quote: not applicable Judgement comment: unclear number randomised and how many received treatment. Appears to be a per protocol analysis.

**Table:** Vles 2020

***Study characteristics***		
Methods	**Study design:** RCT **Intention‐to‐treat analysis:** yes **Duration of study:** recruitment (July 2014 and May 2015) + follow‐up (6 weeks) = 11.5 months **Power calculation reached:** yes "Based on an effect size of 0.50, a significance level of 0.05, a power of 0.80 and an allocation ratio of 1, a sample size of 128 patients (two‐tailed independent t test) was calculated. This was rounded down to 60 patients per group." **Transfusion strategy:** transfusion with PRBCs was considered in patients with a postoperative Hb < 8.0 g/dL or < 8.5 g/dL with accompanying symptoms of anaemia resistant to fluid resuscitation and/or a cardiac past medical history. If deemed necessary, the number of units of PRBCs was estimated according to the Hb or the severity of symptoms with the intention to increase the Hb to ≥ 8.0 g/dL. **Was the trial stopped early:** no	
Participants	**Baseline characteristics** TXA, IV *Age (years) (mean SD)*: 61.5 (11.8) *Ethnicity*: not reported *Gender (males, females)*: 29/60 (48.3%) M; 31/60 (51.7%) F *Length of surgery (minutes) (mean SD)*: not reported *Proportion of participants on anticoagulants prior to surgery (n/N, %)*: not reported *Incidence of preoperative anaemia (n/N, %)*: not reported *Co‐morbidities (n/N, %)*: major comorbidities excluded *ASA 1 (n/N, %)*: not reported *ASA 2 (n/N, %)*: not reported *ASA 3 (n/N, %)*: not reported *ASA 4 (n/N, %)*: not reported *Number of participants randomised*: 60 *Number of participants receiving treatment*: 60 *Number of participants analysed*: 60 *Dropout rate*: 0/60, 0% TXA, IA *Age (years) (mean SD)*: 64.0 (13.4) *Ethnicity*: not reported *Gender (males, females)*: 22/60 (36.7%) M; 38/60 (63.3%) F *Length of surgery (minutes) (mean SD)*: not reported *Proportion of participants on anticoagulants prior to surgery (n/N, %)*: not reported *Incidence of preoperative anaemia (n/N, %)*: not reported *Co‐morbidities (n/N, %)*: major comorbidities excluded *ASA 1 (n/N, %)*: not reported *ASA 2 (n/N, %)*: not reported *ASA 3 (n/N, %)*: not reported *ASA 4 (n/N, %)*: not reported *Number of participants randomised*: 60 *Number of participants receiving treatment*: 60 *Number of participants analysed*: 60 *Dropout rate*: 0/60, 0% Overall *Age (years) (mean SD)*: not reported *Ethnicity*: not reported *Gender (males, females)*: 51/120 M (42.5%), 69/120 F (57.5%) *Length of surgery (minutes) (mean SD)*: not reported *Proportion of participants on anticoagulants prior to surgery (n/N, %)*: not reported *Incidence of preoperative anaemia (n/N, %)*: not reported *Co‐morbidities (n/N, %)*: major comorbidities excluded *ASA 1 (n/N, %)*: not reported *ASA 2 (n/N, %)*: not reported *ASA 3 (n/N, %)*: not reported *ASA 4 (n/N, %)*: not reported *Number of participants randomised*: 120 *Number of participants receiving treatment*: 120 *Number of participants analysed*: 120 *Dropout rate*: 0/120, 0% **Inclusion criteria:** all patients over the age of 18 years, who were scheduled for a primary unilateral THA via DAA for osteoarthritis (OA) or avascular necrosis (AVN) between July 2014 and May 2015, were eligible for inclusion in the study **Exclusion criteria:** 1) history of coagulopathy, 2) allergy to tranexamic acid, 3) preoperative anaemia, 4) fibrinolytic disorders, 5) history of arterial or venous thromboembolic disease, 6) disturbances of colour vision, 7) pregnancy, 8) breastfeeding, 9) major comorbidities, 10) participation in another clinical trial, 11) platelet count below 150,000/mm^3^, PT below 70% and INR above 1.5 **If TKR, is tourniquet used:** not applicable ‐ hips **Indication for surgery:** osteoarthritis (OA) or avascular necrosis (AVN) **Type of anaesthetic:** spinal **Type of surgery:** direct anterior primary unilateral total hip arthroplasty	
Interventions	**Intervention characteristics** TXA, IV IV solution of 1.5 g TXA in 100 mL 0.9% saline just before closure of the wound TXA, IV, 1.5 g, intraop TXA, IA The circulating nurse prepared a solution of 3.0 g TXA in 100 mL 0.9% saline, injected via the drain to the deep subfascial space TXA, IA, 3 g, intraop	
Outcomes	*Primary outcome:* Postoperative hidden blood loss *Secondary outcomes:* Amount of PRBCs transfused Length of hospital stay Development of VTEs (for which no active screening was performed)	
Notes	**Sponsorship source:** non‐pharmaceutical (this research received no specific grant from any funding agency in the public, commercial or not‐for‐profit sectors) **Country:** Belgium **Setting:** single‐centre **Comments:** none **Author's name:** GF Vles **Institution:** University Hospitals, Leuven **Email:** gfvles@gmail.com **Address:** Department of Orthopaedic Surgery, University Hospitals Leuven – Gasthuisberg, Herestraat 49, 3000 Leuven, Belgium **Native language of paper:** English **Reference type:** full text (1), abstract (1), trial registration (1) **Trial registration number:** NCT01940692 **Was it translated for this review:** no	
***Risk of bias***		
**Bias**	**Authors' judgement**	**Support for judgement**
Random sequence generation (selection bias)	Low risk	Quote: "allocated (1:1) to either group using a computer‐generated randomization table (www.randomizer.org)." Judgement comment: adequate method of sequence generation with computer‐generated code.
Allocation concealment (selection bias)	Unclear risk	Quote: not applicable Judgement comment: method of allocation concealment not described.
Blinding of participants and personnel (performance bias) Subjective outcomes	Low risk	Quote: "The administration was performed by an anaesthetist not involved in further follow‐up or care of the patient. The dose of 1.5 g was based on several benchmark studies. A circulating nurse also prepared a syringe with 100 ml saline and handed this over to the scrub nurse and surgeon not aware of the composition, who would then inject this via the drain to the deep subfascial space." Judgement comment: surgical team blinded to allocation.
Blinding of participants and personnel (performance bias) Objective outcomes	Low risk	Quote: not applicable Judgement comment: objective outcome for personnel, so low risk of bias regardless of quality of blinding.
Blinding of outcome assessment (detection bias) Subjective outcomes	Low risk	Quote: not applicable Judgement comment: double‐blind study.
Blinding of outcome assessment (detection bias) Objective outcomes	Low risk	Quote: not applicable Judgement comment: objective outcome for outcome assessor, so low risk of bias regardless of quality of blinding.
Incomplete outcome data (attrition bias) All outcomes	Low risk	Quote: not applicable Judgement comment: no obvious outcome data missing.
Selective reporting (reporting bias)	High risk	Quote: not applicable Judgement comment: one outcome specified in the trial registration was not reported: severity of pain at rest as determined with the use of a visual analogue scale.
Other bias	Unclear risk	Quote: not applicable Judgement comment: according to their power calculation, the target sample size should have been 128, but this was rounded down to 120. Reasons for stopping at 120 participants are not fully explained.

**Table:** Wang 2018

***Study characteristics***		
Methods	**Study design:** RCT **Intention‐to‐treat analysis:** yes **Duration of study:** 11 (4 + 7) months **Power calculation reached:** yes **Transfusion strategy:** according to the blood transfusion protocol, based on the guidelines of Chinese Ministry of Health, an allogeneic transfusion was given if the Hb level was < 7 g/dL in asymptomatic patients or between 7 g/dL and 10 g/dL in symptomatic patients. **Was the trial stopped early:** no	
Participants	**Baseline characteristics** TXA 2 g preop + placebo postop repeated dose *Age (years) (mean SD)*: 63.02 (14.14) *Ethnicity*: not reported *Gender (males, females)*: 15/50 M (30%); 35/50 F (70%) *Length of surgery (minutes)*: 66.21 (10.40) *Proportion of participants on anticoagulants prior to surgery (n/N, %)*: 0/50 (0%) *Incidence of preoperative anaemia (n/N, %)*: not reported *Co‐morbidities (n/N, %)*: not reported *ASA 1 (n/N, %)*: 11/50, (22%) *ASA 2 (n/N, %)*: 32/50, (64%) *ASA 3 (n/N, %)*: 7/50, (14%) *ASA 4 (n/N, %)*: not reported *Number of participants randomised*: 50 *Number of participants receiving treatment*: 50 *Number of participants analysed*: 50 *Dropout rate*: 0/50 (0%) TXA 2 g preop + 1 g postop + placebo repeated dose *Age (years) (mean SD)*: 65.60 (8.55) *Ethnicity*: not reported *Gender (males, females)*: 13/50 M (26%); 37/50 F (74%) *Length of surgery (minutes)*: 67.56 (12.21) *Proportion of participants on anticoagulants prior to surgery (n/N, %)*: 0/50 (0%) *Incidence of preoperative anaemia (n/N, %)*: not reported *Co‐morbidities (n/N, %)*: not reported *ASA 1 (n/N, %)*: 12/50, (24%) *ASA 2 (n/N, %)*: 30/50, (60%) *ASA 3 (n/N, %)*: 8/50, (16%) *ASA 4 (n/N, %)*: not reported *Number of participants randomised*: 50 *Number of participants receiving treatment*: 50 *Number of participants analysed*: 50 *Dropout rate*: 0/50 (0%) TXA 2 g preop + 1 g postop repeated dose + placebo *Age (years) (mean SD)*: 64.10 (11.36) *Ethnicity*: not reported *Gender (males, females)*: 17/50 M (34%); 33/50 F (66%) *Length of surgery (minutes)*: 67.58 (13.25) *Proportion of participants on anticoagulants prior to surgery (n/N, %)*: 0/50 (0%) *Incidence of preoperative anaemia (n/N, %)*: not reported *Co‐morbidities (n/N, %)*: not reported *ASA 1 (n/N, %)*: 10/50, (20%) *ASA 2 (n/N, %)*: 35/50, (70%) *ASA 3 (n/N, %)*: 5/50, (10%) *ASA 4 (n/N, %)*: not reported *Number of participants randomised*: 50 *Number of participants receiving treatment*: 50 *Number of participants analysed*: 50 *Dropout rate*: 0/50 (0%) TXA 2 g preop + 1 g postop repeated dose *Age (years) (mean SD)*: 63.42 (11.72) *Ethnicity*: not reported *Gender (males, females)*: 16/50 M (32%); 34/50 F (68%) *Length of surgery (minutes)*: 68.56 (12.45) *Proportion of participants on anticoagulants prior to surgery (n/N, %)*: 0/50 (0%) *Incidence of preoperative anaemia (n/N, %)*: not reported *Co‐morbidities (n/N, %)*: not reported *ASA 1 (n/N, %)*: 12/50, (24%) *ASA 2 (n/N, %)*: 31/50, (62%) *ASA 3 (n/N, %)*: 7/50, (14%) *ASA 4 (n/N, %)*: not reported *Number of participants randomised*: 50 *Number of participants receiving treatment*: 50 *Number of participants analysed*: 50 *Dropout rate*: 0/50 (0%) Overall *Age (years) (mean SD)*: not reported *Ethnicity*: not reported *Gender (males, females)*: not reported *Length of surgery (minutes)*: not reported *Proportion of participants on anticoagulants prior to surgery (n/N, %)*: not reported *Incidence of preoperative anaemia (n/N, %)*: not reported *Co‐morbidities (n/N, %)*: not reported *ASA 1 (n/N, %)*: not reported *ASA 2 (n/N, %)*: not reported *ASA 3 (n/N, %)*: not reported *ASA 4 (n/N, %)*: not reported *Number of participants randomised*: not reported *Number of participants receiving treatment*: 200 *Number of participants analysed*: 200 *Dropout rate*: not reported **Inclusion criteria:** all patients aged > 18 years who were scheduled for elective, unilateral, primary TKA **Exclusion criteria:** 1) diagnosis other than primary osteoarthritis (OA), absence of written informed consent, 2) the use of spinal anaesthesia, 3) simultaneous bilateral TKAs, 4) a history of medical comorbidity including deep vein thrombosis (DVT) or pulmonary embolism (PE), haematological disorders, 5) current anticoagulant therapy (warfarin or heparin) within 1 week, 6) a history of allergy to TXA **If TKR, is tourniquet used:** no **Indication for surgery:** primary osteoarthritis (OA) **Type of anaesthetic:** general anaesthetic **Type of surgery:** primary TKA	
Interventions	**Intervention characteristics** TXA 2 g preop + placebo postop repeated dose Those in group A were given an oral dose of 2 g of TXA (4 tablets of 500 mg) approximately 2 hours preoperatively and then an oral form of 1 g of placebo pills (2 tablets of 500 mg) identical in appearance, with no active ingredient, 3, 9 and 15 hours postoperatively. TXA, 2 g, oral, 2 hours preop AND TXA, 1.5 g, IA, intraop, AND placebo, oral, 3, 9, 15 hours postop TXA 2 g preop + 1 g postop + placebo repeated dose Those in group B were given 2 g of oral TXA 2 hours preoperatively, 1 g of oral TXA 3 hours postoperatively and 1 g of placebo 9 and 15 hours postoperatively. TXA, 2 g, oral, 2 hours preop + TXA, 1 g, oral, 3 hours postop AND TXA, 1.5, IA, intraop AND placebo, oral, 9 + 15 hours postop TXA 2 g preop + 1 g postop repeated dose + placebo Those in group C were given 2 g of oral TXA 2 hours preoperatively, 1 g of oral TXA 3 and 9 hours postoperatively, and 1 g of placebo 15 hours postoperatively. TXA, 2 g, 2 hours preop + TXA, 1 g, oral, 3 + 9 hours postop AND TXA, 1.5, IA, intraop AND placebo, oral, 15 hours postop TXA 2 g preop + 1 g postop repeated dose Those in group D were given 2 g oral TXA 2 hours preoperatively and 1 g of oral TXA 3, 9 and 15 hours postoperatively. All patients received an intra‐articular dose of 1.5 g of TXA on the basis of our previous study. TXA, 2 g, 2 hours preop + TXA, 1 g, oral, 3 + 9 + 15 hours postop AND TXA, 1.5, IA, intraop	
Outcomes	*Primary outcome:* Total blood loss *Secondary outcomes:* Hidden blood loss Reduction in the level of haemoglobin Rate of transfusion Adverse events	
Notes	**Sponsorship source:** non‐pharmaceutical **Country:** China **Setting:** single‐centre **Comments:** none **Author's name:** Z‐K Zhou **Institution:** Sichuan University **Email:** zongkehx@163.com **Address:** W‐N Zeng, MD, Associated Professor Center for Joint Surgery, Southwest Hospital, Third Military Medical University, Chongqing, China **Native language of paper:** English **Reference type:** full text (1) **Trial registration number:** ChiCTR‐IPR‐17012265 **Was it translated for this review:** no	
***Risk of bias***		
**Bias**	**Authors' judgement**	**Support for judgement**
Random sequence generation (selection bias)	Low risk	Quote: "computer‐generated randomization" Judgement comment: computer‐generated randomisation.
Allocation concealment (selection bias)	Low risk	Quote: "Allocation was concealed in consecutively numbered, sealed, opaque envelopes." Judgement comment: consecutively numbered, sealed, opaque envelopes.
Blinding of participants and personnel (performance bias) Subjective outcomes	Low risk	Quote: "The envelope containing the assignment to a group was opened on the morning of surgery by a dedicated study nurse not involved with patient care and evaluation, and the inclusion criteria were rechecked by a dedicated assistant. Under the supervision of a research pharmacist, the appropriate dose and placebo were prepared by the same senior nurse not involved with patient care and evaluation to ensure the identical appearance and blinding." Judgement comment: blinding of key personnel ensured and unlikely blinding could have been broken.
Blinding of participants and personnel (performance bias) Objective outcomes	Low risk	Quote: not applicable Judgement comment: objective outcome for personnel, so low risk of bias regardless of quality of blinding.
Blinding of outcome assessment (detection bias) Subjective outcomes	Low risk	Quote: "The envelope containing the assignment to a group was opened on the morning of surgery by a dedicated study nurse not involved with patient care and evaluation, and the inclusion criteria were rechecked by a dedicated assistant. Under the supervision of a research pharmacist, the appropriate dose and placebo were prepared by the same senior nurse not involved with patient care and evaluation to ensure the identical appearance and blinding. Patients, surgeons, anaesthetists, care providers, and data collectors were all blinded to allocation." Judgement comment: blinding of outcome assessors ensured and unlikely to have been broken.
Blinding of outcome assessment (detection bias) Objective outcomes	Low risk	Quote: not applicable Judgement comment: objective outcome for assessors, so low risk of bias regardless of quality of blinding.
Incomplete outcome data (attrition bias) All outcomes	Low risk	Quote: not applicable Judgement comment: all outcome data reported.
Selective reporting (reporting bias)	Low risk	Quote: not applicable Judgement comment: all trial registration outcomes reported in the full paper.
Other bias	Low risk	Quote: not applicable Judgement comment: appears to be free of other biases.

**Table:** Wang 2019a

***Study characteristics***		
Methods	**Study design:** RCT **Intention‐to‐treat analysis:** yes **Duration of study:** 7 months (September 2017 and April 2018) + 90 days follow‐up (3 months) = 10 months **Power calculation reached:** yes **Transfusion strategy:** based on the guidelines of the Chinese Ministry of Health, an allogeneic transfusion was given if Hb was < 70 g/L in asymptomatic patients or between 70 g/L and 100 g/L in symptomatic patients (i.e. fatigue, poor appetite, anaemia or myocardial ischaemia) in hospital **Was the trial stopped early:** no	
Participants	**Baseline characteristics** TXA, IV + TXA, oral *Age (years) (mean SD)*: 63.0 (13.9) *Ethnicity*: not reported *Gender (males, females)*: 15/60 M (25%), 45/60 F (75%) *Length of surgery (minutes)*: 62.9 (13.1) *Proportion of participants on anticoagulants prior to surgery (n/N, %)*: not reported *Incidence of preoperative anaemia (n/N, %)*: not reported *Co‐morbidities (n/N, %)*: not reported *ASA 1 (n/N, %)*: not reported separately: overall 2.1 ± 0.3 *ASA 2 (n/N, %)*: not reported *ASA 3 (n/N, %)*: not reported *ASA 4 (n/N, %)*: not reported *Number of participants randomised*: 60 *Number of participants receiving treatment*: 60 *Number of participants analysed*: 60 *Dropout rate*: 0/60, 0% TXA, IV + placebo, oral *Age (years) (mean SD)*: 64.1 (9.3) *Ethnicity*: not reported *Gender (males, females)*: 11/58 M (18.96%), 47/58 F (81.03%) *Length of surgery (minutes)*: 63.6 (12.4) *Proportion of participants on anticoagulants prior to surgery (n/N, %)*: not reported *Incidence of preoperative anaemia (n/N, %)*: not reported *Co‐morbidities (n/N, %)*: not reported *ASA 1 (n/N, %)*: not reported separately: overall 2.1 ± 0.4 *ASA 2 (n/N, %)*: not reported *ASA 3 (n/N, %)*: not reported *ASA 4 (n/N, %)*: not reported *Number of participants randomised*: 60 *Number of participants receiving treatment*: 58 *Number of participants analysed*: 58 *Dropout rate*: 2/60, 3.3% Overall *Age (years) (mean SD)*: not reported *Ethnicity*: not reported *Gender (males, females)*: 26 M, 92 F *Length of surgery (minutes)*: not reported *Proportion of participants on anticoagulants prior to surgery (n/N, %)*: not reported *Incidence of preoperative anaemia (n/N, %)*: not reported *Co‐morbidities (n/N, %)*: not reported *ASA 1 (n/N, %)*: not reported *ASA 2 (n/N, %)*: not reported *ASA 3 (n/N, %)*: not reported *ASA 4 (n/N, %)*: not reported *Number of participants randomised*: 120 *Number of participants receiving treatment*: 118 *Number of participants analysed*: 118 *Dropout rate*: 2/120, 1.6% **Inclusion criteria:** patients with primary osteoarthritis undergoing primary unilateral TKA **Exclusion criteria:** secondary osteoarthritis, allergy to this medicine, a history of coexisting diseases that cannot tolerate surgery or general anaesthesia and active cancer **If TKR, is tourniquet used:** a tourniquet was not used **Indication for surgery:** primary osteoarthritis **Type of anaesthetic:** general **Type of surgery:** primary TKA	
Interventions	**Intervention characteristics** TXA, IV + TXA, oral The patients in Group A were given TXA (20 mg/kg) intravenously 10 min before the surgery and 3 h after the operation, and then the patients received 1 g TXA orally from postoperative day (POD) 1 to POD 14. TXA, IV, 20 mg, preop and postop + TXA, oral, 1 g, postop day 1 to 14 TXA, IV + placebo, oral Patients in Group B received IV TXA (20 mg/kg) intravenously 10 min before the surgery and 3 h after the operation, and then the patients received placebo pills identical in quantity to oral TXA from POD 1 to POD 14. TXA, IV, 20 mg, preop and postop + placebo, oral, 1g, postop day 1 to 14	
Outcomes	*Primary outcomes:* Total blood loss Estimated blood loss *Secondary outcomes:* Subcutaneous ecchymosis morbidity Area of ecchymosis Patients requiring transfusion Units transfused Postoperative laboratory values (i.e. haemoglobin, haematocrit, FDP and D‐dimer) Postoperative knee function Knee circumference VAS pain score Length of hospital stay	
Notes	**Sponsorship source:** non‐pharmaceutical (this research was funded by the China Health Ministry Program (201302007), who is not involved in study design, data collection, analysis and interpretation or manuscript preparation) **Country:** China **Setting:** single‐centre **Comments:** none **Author's name:** H‐Y Wang **Institution:** Sichuan University **Email:** Dr Xin Tang: tangxin9388@163.com; Dr Zong‐Ke Zhou: zongkehx@163.com **Address:** Department of Orthopedics, West China Hospital/West China School of Medicine, Sichuan University, 37# Wuhou Guoxue Road, Chengdu 610041, People’s Republic of China **Native language of paper:** English **Reference type:** full text (1), trial registration (1) **Trial registration number:** ChiCTR‐IPR‐17012264 **Was it translated for this review:** no	
***Risk of bias***		
**Bias**	**Authors' judgement**	**Support for judgement**
Random sequence generation (selection bias)	Low risk	Quote: "All patients were randomized into two groups (Group A: IV and subsequent oral TXA; Group B: IV TXA only) based on a computer‐generated randomization list generated using Randomization.com." Judgement comment: adequate method of sequence generation with computer‐generated random numbers.
Allocation concealment (selection bias)	Low risk	Quote: "The randomization assignments were placed into sequentially numbered opaque sealed envelopes, which were kept by a certificated research pharmacist." Judgement comment: adequate method of central allocation concealment by pharmacy
Blinding of participants and personnel (performance bias) Subjective outcomes	Low risk	Quote: "The envelope was opened on the day of operation, and the corresponding drug and placebo were handled by a researcher who was not involved in patient care. The patients, trial participants, anesthesiologists, outcome assessors, and data collectors were blinded to allocation." Judgement comment: participants and personnel blinded.
Blinding of participants and personnel (performance bias) Objective outcomes	Low risk	Quote: not applicable Judgement comment: objective outcome for personnel, so low risk of bias regardless of quality of blinding.
Blinding of outcome assessment (detection bias) Subjective outcomes	Low risk	Quote: "The patients, trial participants, anesthesiologists, outcome assessors, and data collectors were blinded to allocation." Judgement comment: outcome assessors blinded.
Blinding of outcome assessment (detection bias) Objective outcomes	Low risk	Quote: not applicable Judgement comment: objective outcome for personnel, so low risk of bias regardless of quality of blinding.
Incomplete outcome data (attrition bias) All outcomes	Low risk	Quote: not applicable Judgement comment: no obvious outcome data missing.
Selective reporting (reporting bias)	Low risk	Quote: not applicable Judgement comment: all outcomes planned in the protocol or prospective trial registration are reported.
Other bias	Low risk	Quote: not applicable Judgement comment: no other concerns such as early stopping or imbalanced study arms.

**Table:** Wang 2019b

***Study characteristics***		
Methods	**Study design:** RCT **Intention‐to‐treat analysis:** yes **Duration of study:** 4 months (June 2017 to October 2017) + 3 months (follow‐up) = 7 months **Power calculation reached:** yes **Transfusion strategy:** a standardised blood transfusion protocol was followed for all patients (consistent with the perioperative transfusion guidelines of the Chinese Ministry of Health), whereby blood transfusion was indicated for a haemoglobin (Hb) level of < 7.0 g/dL in asymptomatic patients or a Hb level of < 10.0 g/dL in patients who developed any anaemia‐related organ dysfunction, intolerable symptoms of anaemia or ongoing blood loss **Was the trial stopped early:** no	
Participants	**Baseline characteristics** TXA, oral, 2 g, 2 h pre‐incision + placebo, oral, postop 3, 9 and 15 h + TXA, IA, 1 g *Age (years) (mean SD)*: 64.41 (13.94) *Ethnicity*: not reported *Gender (males, females)*: 13/50 M (26%); 37/50 F (74%) *Length of surgery (minutes) (mean SD)*: 69.67 ± 11.17 *Proportion of participants on anticoagulants prior to surgery (n/N, %)*: treatment with anticoagulant therapy (warfarin or heparin) within 1 week prior to surgery ‐ excluded *Incidence of preoperative anaemia (n/N, %)*: not reported *Co‐morbidities (n/N, %)*: not reported *ASA 1 (n/N, %)*: 13/50, 26% *ASA 2 (n/N, %)*: 30/50, 60% *ASA 3 (n/N, %)*: 7/50, 14% *ASA 4 (n/N, %)*: 0/50, 0% *Number of participants randomised*: 50 *Number of participants receiving treatment*: not reported *Number of participants analysed*: 50 *Dropout rate*: 0/50, 0% TXA, oral, 2 g, 2 h pre‐incision + TXA, oral, 1g, 3 h post + placebo, oral, 9, 15 h post + TXA, IA, 1 g *Age (years) (mean SD)*: 66.98 (9.71) *Ethnicity*: not reported *Gender (males, females)*: 15/50 M (30%); 35/50 F (70%) *Length of surgery (minutes) (mean SD)*: 70.54 ± 12.63 *Proportion of participants on anticoagulants prior to surgery (n/N, %)*: treatment with anticoagulant therapy (warfarin or heparin) within 1 week prior to surgery ‐ excluded *Incidence of preoperative anaemia (n/N, %)*: not reported *Co‐morbidities (n/N, %)*: not reported *ASA 1 (n/N, %)*: 11/50, 22% *ASA 2 (n/N, %)*: 34/50, 68% *ASA 3 (n/N, %)*: 5/50, 10% *ASA 4 (n/N, %)*: 0/50, 0% *Number of participants randomised*: 50 *Number of participants receiving treatment*: not reported *Number of participants analysed*: 50 *Dropout rate*: 0/50, 0% TXA, oral, 2 g, 2 h pre‐incision + TXA, oral, 1 g, 3, 9 h post + placebo, oral, 15 h postop + TXA, IA, 1 g *Age (years) (mean SD)*: 66.76 (9.87) *Ethnicity*: not reported *Gender (males, females)*: 17/50 M (34%); 33/50 F (66%) *Length of surgery (minutes) (mean SD)*: 67.48 ± 12.67 *Proportion of participants on anticoagulants prior to surgery (n/N, %)*: treatment with anticoagulant therapy (warfarin or heparin) within 1 week prior to surgery ‐ excluded *Incidence of preoperative anaemia (n/N, %)*: not reported *Co‐morbidities (n/N, %)*: not reported *ASA 1 (n/N, %)*: 15/50, 30% *ASA 2 (n/N, %)*: 28/50, 56% *ASA 3 (n/N, %)*: 7/50, 14% *ASA 4 (n/N, %)*: 0/50, 0% *Number of participants randomised*: 50 *Number of participants receiving treatment*: not reported *Number of participants analysed*: 50 *Dropout rate*: 0/50, 0% TXA, oral, 2 g, 2 hours pre‐incision + TXA, oral, 1 g, 3, 9, 15 h post + TXA, IA, 1 g *Age (years) (mean SD)*: 63.94 ± 12.37 *Ethnicity*: not reported *Gender (males, females)*: 14/50 M (28%); 36/50 F (72%) *Length of surgery (minutes) (mean SD)*: 67.07 ± 11.64 *Proportion of participants on anticoagulants prior to surgery (n/N, %)*: treatment with anticoagulant therapy (warfarin or heparin) within 1 week prior to surgery ‐ excluded *Incidence of preoperative anaemia (n/N, %)*: not reported *Co‐morbidities (n/N, %)*: not reported *ASA 1 (n/N, %)*: 11/50, 22% *ASA 2 (n/N, %)*: 30/50, 60% *ASA 3 (n/N, %)*: 9/50, 18% *ASA 4 (n/N, %)*: 0/50, 0% *Number of participants randomised*: 50 *Number of participants receiving treatment*: not reported *Number of participants analysed*: 50 *Dropout rate*: 0/50, 0% Overall *Age (years) (mean SD)*: not reported *Ethnicity*: not reported *Gender (males, females)*: not reported *Length of surgery (minutes) (mean SD)*: not reported *Proportion of participants on anticoagulants prior to surgery (n/N, %)*: not reported *Incidence of preoperative anaemia (n/N, %)*: not reported *Co‐morbidities (n/N, %)*: not reported *ASA 1 (n/N, %)*: not reported *ASA 2 (n/N, %)*: not reported *ASA 3 (n/N, %)*: not reported *ASA 4 (n/N, %)*: not reported *Number of participants randomised*: 200 *Number of participants receiving treatment*: not reported *Number of participants analysed*: 200 *Dropout rate*: 0/200, 0% **Inclusion criteria:** consecutive adult patients (18 years of age and over) who were scheduled for primary unilateral total hip arthroplasty **Exclusion criteria:** exclusion criteria included a diagnosis other than osteoarthritis or osteonecrosis of the femoral head, a known allergy to TXA, use of spinal anaesthesia, a history of a haematopoietic or haemorrhagic disorder, a history of deep venous thrombosis (DVT) or pulmonary embolism (PE), treatment with anticoagulant therapy (warfarin or heparin) within 1 week prior to surgery, and refusal of participation or blood products. **If TKR, is tourniquet used:** not applicable **Indication for surgery:** osteoarthritis or osteonecrosis of the femoral head **Type of anaesthetic:** general (97% received this), assumed the rest had spinal **Type of surgery:** primary THA	
Interventions	**Intervention characteristics** TXA, oral, 2 g, 2 h pre‐incision + placebo, oral, postop 3, 9 and 15 h + TXA, IA, 1 g 2.0 g of oral TXA (4 tablets, 0.5 g each) at 2 hours preoperatively and 1.0 g of placebo (2 tablets, 0.5 g each) at 3, 9 and 15 hours postoperatively (Group A, control) TXA, oral, 2 g, 2 h pre‐incision + TXA, oral, 1 g, 3 h post + placebo, oral, 9, 15 h post + TXA, IA, 1 g 2.0 g of oral TXA at 2 hours preoperatively, 1.0 g of oral TXA at 3 hours postoperatively, and 1.0 g of placebo at 9 and 15 hours postoperatively TXA, oral, 2 g, 2 h pre‐incision + TXA, oral, 1 g, 3, 9 h post + placebo, oral, 15 h postop + TXA, IA, 1 g 2.0 g of oral TXA at 2 hours preoperatively, 1.0 g of oral TXA at 3 and 9 hours postoperatively, and 1.0 g of placebo at 15 hours postoperatively TXA, oral, 2 g, 2 hours pre‐incision + TXA, oral, 1 g, 3, 9, 15 h post + TXA, IA, 1 g 2.0 g of oral TXA at 2 hours preoperatively and 1.0 g of oral TXA at 3, 9 and 15 hours postoperatively	
Outcomes	*Primary outcome:* Total blood loss *Secondary outcomes:* Hb reduction Intraoperative blood loss Operative time Hospital stay Transfusion rate Thromboembolic complications Adverse events	
Notes	**Sponsorship source:** not reported (but no external funding was received in support of this study) **Country:** China **Setting:** single‐centre **Comments:** none **Author's name:** D Wang **Institution:** Sichan University **Email:** zongke@126.com; weinanzeng@163.com **Address:** Department of Orthopedics, West China Hospital/West China School of Medicine, Sichuan University, Chengdu, People’s Republic of China **Native language of paper:** English **Reference type:** full text (1), trial registration (1) **Trial registration number:** ChiCTR‐IPR‐17012266 **Was it translated for this review:** no	
***Risk of bias***		
**Bias**	**Authors' judgement**	**Support for judgement**
Random sequence generation (selection bias)	Low risk	Quote: "A random allocation sequence was computer‐generated" Judgement comment: adequate method of sequence generation with computer‐generated code.
Allocation concealment (selection bias)	Low risk	Quote: "concealed in consecutively numbered, opaque, sealed envelopes" Judgement comment: adequate method of central allocation concealment.
Blinding of participants and personnel (performance bias) Subjective outcomes	Low risk	Quote: "An envelope was opened prior to surgery, and the study medication and placebo were prepared by a dedicated study nurse not involved with patient care or evaluation to ensure identical protocol appearance and blinding." Judgement comment: participants and personnel blinded.
Blinding of participants and personnel (performance bias) Objective outcomes	Low risk	Quote: "Patients, surgeons, anesthesiologists, care providers, and data collectors were blinded to the allocation sequence" Judgement comment: patients and personnel blinded. Objective outcome for personnel, so low risk of bias regardless of quality of blinding.
Blinding of outcome assessment (detection bias) Subjective outcomes	Low risk	Quote: "Patients, surgeons, anesthesiologists, care providers, and data collectors were blinded to the allocation sequence." Judgement comment: data collectors were blinded.
Blinding of outcome assessment (detection bias) Objective outcomes	Low risk	Quote: not applicable Judgement comment: objective outcome for outcome assessors, so low risk of bias regardless of quality of blinding.
Incomplete outcome data (attrition bias) All outcomes	Low risk	Quote: not applicable Judgement comment: no obvious outcome data missing.
Selective reporting (reporting bias)	Low risk	Quote: not applicable Judgement comment: all outcomes planned in the protocol or prospective trial registration are reported.
Other bias	Low risk	Quote: not applicable Judgement comment: no other concerns such as early stopping or imbalanced study arms.

**Table:** Wang 2019c

***Study characteristics***		
Methods	**Study design:** RCT **Intention‐to‐treat analysis:** per protocol **Duration of study:** 6 months (October 2017 to April 2018 ‐ recruitment) + 3 months follow‐up = 9 months **Power calculation reached:** yes **Transfusion strategy:** allogeneic red cell transfusion was given if the Hb level was < 7 g/dL in patients who were asymptomatic or if a patient with intolerable symptoms of anaemia (dyspnoea, palpitation, pallor or tachycardia) or any organ dysfunction that was attributable to anaemia regardless of the Hb level. **Was the trial stopped early:** no	
Participants	**Baseline characteristics** TXA, oral, 2 g, 2 h pre‐incision + placebo, oral, 1 g postop 3, 7, 11, 15 h *Age (years) (mean SD)*: 68.7 (9.8) *Ethnicity*: not reported *Gender (males, females)*: 22/60 M (36.7%), 38/60 F (63.3%) *Length of surgery (minutes) (mean SD)*: 69.8 (14.1) *Proportion of participants on anticoagulants prior to surgery (n/N, %)*: patients excluded if full dose of warfarin or heparin consumed within 1 week of surgery *Incidence of preoperative anaemia (n/N, %)*: not reported *Co‐morbidities (n/N, %)*: not reported *ASA 1 (n/N, %)*: not reported *ASA 2 (n/N, %)*: ASA 1/2 ‐ 52 (86.7) *ASA 3 (n/N, %)*: ASA 3 and greater ‐ 8 (13.3) *ASA 4 (n/N, %)*: not reported *Number of participants randomised*: 60 *Number of participants receiving treatment*: 60 *Number of participants analysed*: 60 *Dropout rate*: 0/60, 0% TXA, oral, 2 g, 2 h pre‐incision + TXA, oral, 1 g postop 3 h + placebo, oral, 1 g postop 7, 11, 15 h *Age (years) (mean SD)*: 67.3 (8.3) *Ethnicity*: not reported *Gender (males, females)*: 23/60 M (38.3%); 37/60 F (61.7%) *Length of surgery (minutes) (mean SD)*: 70.3 (10.7) *Proportion of participants on anticoagulants prior to surgery (n/N, %)*: patients excluded if full dose of warfarin or heparin consumed within 1 week of surgery *Incidence of preoperative anaemia (n/N, %)*: not reported *Co‐morbidities (n/N, %)*: not reported *ASA 1 (n/N, %)*: not reported *ASA 2 (n/N, %)*: ASA 1/2 ‐ 50 (83.3) *ASA 3 (n/N, %)*: ASA 3 and greater ‐ 10 (16.7) *ASA 4 (n/N, %)*: not reported *Number of participants randomised*: 60 *Number of participants receiving treatment*: 60 *Number of participants analysed*: 60 *Dropout rate*: 0/60, 0% TXA, oral, 2 g, 2 h pre‐incision + TXA, oral, 1 g postop 3, 7 h + placebo, oral, 1 g postop 11, 15 h *Age (years) (mean SD)*: 68.9 (7.7) *Ethnicity*: not reported *Gender (males, females)*: 22/60 M (36.7%); 38/60 F (63.3%) *Length of surgery (minutes) (mean SD)*: 68.3 (9.8) *Proportion of participants on anticoagulants prior to surgery (n/N, %)*: patients excluded if full dose of warfarin or heparin consumed within 1 week of surgery *Incidence of preoperative anaemia (n/N, %)*: not reported *Co‐morbidities (n/N, %)*: not reported *ASA 1 (n/N, %)*: not reported *ASA 2 (n/N, %)*: ASA 1/2 ‐ 52 (86.7) *ASA 3 (n/N, %)*: ASA 3 and greater ‐ 8 (13.3) *ASA 4 (n/N, %)*: not reported *Number of participants randomised*: 60 *Number of participants receiving treatment*: 60 *Number of participants analysed*: 60 *Dropout rate*: 0/60, 0% TXA, oral, 2 g, 2 h pre‐incision + TXA, oral, 1 g postop 3, 7, 11 h + placebo, oral, 1 g postop 15 h *Age (years) (mean SD)*: 67.2 (9.6) *Ethnicity*: not reported *Gender (males, females)*: 24/60 M (38.7%); 36/60 F (60%) *Length of surgery (minutes) (mean SD)*: 69.2 (9.9) *Proportion of participants on anticoagulants prior to surgery (n/N, %)*: patients excluded if full dose of warfarin or heparin consumed within 1 week of surgery *Incidence of preoperative anaemia (n/N, %)*: not reported *Co‐morbidities (n/N, %)*: not reported *ASA 1 (n/N, %)*: not reported *ASA 2 (n/N, %)*: ASA 1/2 ‐ 51 (85.0) *ASA 3 (n/N, %)*: ASA 3 and greater ‐ 9 (15.0) *ASA 4 (n/N, %)*: not reported *Number of participants randomised*: 60 *Number of participants receiving treatment*: 60 *Number of participants analysed*: 60 *Dropout rate*: 0/60, 0% TXA, oral, 2 g, 2 h pre‐incision + TXA, oral, 1 g postop 3, 7, 11, 15 h *Age (years) (mean SD)*: 68.5 (9.5) *Ethnicity*: not reported *Gender (males, females)*: 21/60 M (35.1%); 39/60 F (65%) *Length of surgery (minutes) (mean SD)*: 71.5 (11.6) *Proportion of participants on anticoagulants prior to surgery (n/N, %)*: patients excluded if full dose of warfarin or heparin consumed within 1 week of surgery *Incidence of preoperative anaemia (n/N, %)*: not reported *Co‐morbidities (n/N, %)*: not reported *ASA 1 (n/N, %)*: not reported *ASA 2 (n/N, %)*: ASA 1/2 ‐ 53 (88.3) *ASA 3 (n/N, %)*: ASA 3 and greater ‐ 7 (11.7) *ASA 4 (n/N, %)*: not reported *Number of participants randomised*: 60 *Number of participants receiving treatment*: 60 *Number of participants analysed*: 60 *Dropout rate*: 0/60, 0% Overall *Age (years) (mean SD)*: not reported *Ethnicity*: not reported *Gender (males, females)*: 112/300 M (37.3%); 188/300 F (62.7%) *Length of surgery (minutes) (mean SD)*: not reported *Proportion of participants on anticoagulants prior to surgery (n/N, %)*: patients excluded if full dose of warfarin or heparin consumed within 1 week of surgery *Incidence of preoperative anaemia (n/N, %)*: not reported *Co‐morbidities (n/N, %)*: not reported *ASA 1 (n/N, %)*: not reported *ASA 2 (n/N, %)*: not reported *ASA 3 (n/N, %)*: not reported *ASA 4 (n/N, %)*: not reported *Number of participants randomised*: 300 *Number of participants receiving treatment*: 300 *Number of participants analysed*: 300 *Dropout rate*: 0/300, 0% **Inclusion criteria:** all patients (age > 18 years) with hip osteoarthritis or osteonecrosis of the femoral head, scheduled for elective, unilateral, primary THA **Exclusion criteria:** known allergy to TXA; a haemoglobin (Hb) level of < 11 g/dL; a history of arrhythmia, pulmonary embolism (PE), deep venous thrombosis (DVT) or severe ischaemic heart disease; an acquired or congenital coagulopathy; previous vascular or cardiac bypass surgery; a history of high‐risk medical co‐morbidities (severe renal insufficiency, hepatic failure or severe pulmonary disease); current full‐dose anticoagulant therapy (warfarin or heparin) within 1 week; refusal of blood products or participation; or participation in another clinical trial during the last year **If TKR, is tourniquet used:** not applicable **Indication for surgery:** patients with hip osteoarthritis or osteonecrosis of the femoral head **Type of anaesthetic:** general **Type of surgery:** elective, unilateral, primary THA	
Interventions	**Intervention characteristics** TXA, oral, 2 g, 2 h pre‐incision + placebo, oral, 1 g postop 3, 7, 11, 15 h Group A received an oral form of 2 g of matching placebo (4 tablets of 500 mg) approximately 2 hours pre‐operatively, followed by an oral administration of 1 g placebo 3, 7, 11 and 15 hours postoperatively. TXA, oral, 2 g, 2 h pre‐incision + TXA, oral, 1 g postop 3 h + placebo, oral, 1 g postop 7, 11, 15 h Group B received an oral dose of 2 g of TXA (4 tablets of 500 mg) approximately 2 hours pre‐operatively, 1 g oral TXA 3 hours postoperatively and 1 g placebo 7, 11 and 15 hours postoperatively. TXA, oral, 2 g, 2 h pre‐incision + TXA, oral, 1 g postop 3, 7 h + placebo, oral, 1 g postop 11, 15 h Group C received 2 g oral TXA 2 hours pre‐operatively, 1 g oral TXA 3 and 7 hours postoperatively and 1 g placebo 11 and 15 hours postoperatively TXA, oral, 2 g, 2 h pre‐incision + TXA, oral, 1 g postop 3, 7, 11 h + placebo, oral, 1 g postop 15 h Group D received 2 g oral TXA 2 hours pre‐operatively, 1 g oral TXA 3, 7 and 11 hours postoperatively and 1 g placebo 15 hours postoperatively TXA, oral, 2 g, 2 h pre‐incision + TXA, oral, 1 g postop 3, 7, 11, 15 h Group E received 2 g oralTXA 2 hours pre‐operatively and 1 ‐g oral TXA 3, 5, 11 and 15 hours postoperatively	
Outcomes	*Primary outcome:* Total blood loss *Secondary outcomes:* Hb drop Intraoperative blood loss Allogeneic red cell transfusion rates Number of blood units transfused Length of hospital stay Postoperative changes in joint function (range of motion) Severity of hip pain Thromboembolic and other complications	
Notes	**Sponsorship source:** not reported **Country:** China **Setting:** single‐centre **Comments:** none **Author's name:** D Wang **Institution:** Sichuan University **Email:** zongke@126.com **Address:** Department of Orthopedics, West China School of Medicine, West China Hospital, Sichuan University, Chengdu, China **Native language of paper:** English **Reference type:** full text (1), trial registration (1) **Trial registration number:** ChiCTR‐IPR‐17011669 **Was it translated for this review:** no	
***Risk of bias***		
**Bias**	**Authors' judgement**	**Support for judgement**
Random sequence generation (selection bias)	Low risk	Quote: "Randomization was computer‐generated using Randomization.com." Judgement comment: adequate method of sequence generation with computer‐generated code.
Allocation concealment (selection bias)	Low risk	Quote: "A random allocation sequence was placed into sequentially numbered, opaque, sealed envelopes" Judgement comment: considered as adequate allocation concealment as it is described as "sequentially numbered, opaque, sealed".
Blinding of participants and personnel (performance bias) Subjective outcomes	Low risk	Quote: not applicable Judgement comment: just prior to surgery, the envelope was opened randomly by a senior nurse (not otherwise involved with the collection of patient data and patient care) who also handled study drug preparations to ensure identical appearance and blinding.
Blinding of participants and personnel (performance bias) Objective outcomes	Low risk	Quote: not applicable Judgement comment: objective outcome for personnel, so low risk of bias regardless of quality of blinding.
Blinding of outcome assessment (detection bias) Subjective outcomes	Low risk	Quote: "data collectors were all blinded to the randomization." Judgement comment: outcome assessors/data collectors were blinded.
Blinding of outcome assessment (detection bias) Objective outcomes	Low risk	Quote: not applicable Judgement comment: outcome assessors/data collectors were blinded.
Incomplete outcome data (attrition bias) All outcomes	Low risk	Quote: not applicable Judgement comment: no obvious outcome data missing.
Selective reporting (reporting bias)	Low risk	Quote: not applicable Judgement comment: all outcomes planned in the protocol or prospective trial registration are reported.
Other bias	Low risk	Quote: not applicable Judgement comment: no other concerns such as early stopping or imbalanced study arms.

**Table:** Wu 2018

***Study characteristics***		
Methods	**Study design:** RCT **Intention‐to‐treat analysis:** yes **Duration of study:** 13 months (7 + 6) **Power calculation reached:** yes **Transfusion strategy:** blood transfusions were applied using the guidelines of the Chinese Ministry of Health if the Hb level was < 70 g/L or 70 to 100 g/L with symptoms of anaemia (e.g. change in mental status and palpitations). **Was the trial stopped early:** no	
Participants	**Baseline characteristics** TXA, IV *Age (years) (mean SD)*: 65.1 (63.8 to 66.5) *Ethnicity*: not reported *Gender (males, females)*: 30/50 M (60%); 20/50 F (40%) *Length of surgery (minutes)*: 66.6 (4.6) *Proportion of participants on anticoagulants prior to surgery (n/N, %)*: not reported *Incidence of preoperative anaemia (n/N, %)*: not reported *Co‐morbidities (n/N, %)*: hypertension 7/50 (14%), type 2 diabetes 4/50 (8%) *ASA 1 (n/N, %)*: 25/50 (50%) *ASA 2 (n/N, %)*: 17/50 (34%) *ASA 3 (n/N, %)*: 8/50 (16%) *ASA 4 (n/N, %)*: not reported *Number of participants randomised*: 50 *Number of participants receiving treatment*: 50 *Number of participants analysed*: 50 *Dropout rate*: 0/50 (0%) TXA, oral *Age (years) (mean SD)*: 66.5 (65.1 to 67.9) *Ethnicity*: not reported *Gender (males, females)*: 29/50 M (58%); 21/50 F (42%) *Length of surgery (minutes)*: 68.2 (4.5) *Proportion of participants on anticoagulants prior to surgery (n/N, %)*: not reported *Incidence of preoperative anaemia (n/N, %)*: not reported *Co‐morbidities (n/N, %)*: hypertension 5/50 (10%), type 2 diabetes 3/50 (6%) *ASA 1 (n/N, %)*: 23/50 (46%) *ASA 2 (n/N, %)*: 20/50 (40%) *ASA 3 (n/N, %)*: 7/50 (14%) *ASA 4 (n/N, %)*: not reported *Number of participants randomised*: 50 *Number of participants receiving treatment*: 50 *Number of participants analysed*: 50 *Dropout rate*: 0/50 (0%) Overall *Age (years) (mean SD)*: not reported *Ethnicity*: not reported *Gender (males, females)*: not reported *Length of surgery (minutes)*: not reported *Proportion of participants on anticoagulants prior to surgery (n/N, %)*: not reported *Incidence of preoperative anaemia*: not reported *Co‐morbidities*: not reported *ASA 1 (n/N, %)*: not reported *ASA 2 (n/N, %)*: not reported *ASA 3 (n/N, %)*: not reported *ASA 4 (n/N, %)*: not reported *Number of participants randomised*: 100 *Number of participants receiving treatment*: not reported *Number of participants analysed*: not reported *Dropout rate*: not reported **Inclusion criteria:** patients with a diagnosis of osteoarthritis (OA) or osteonecrosis of the femoral head (ONFH) undergoing unilateral THA **Exclusion criteria:** 1) developmental dysplasia of the hip, 2) rheumatoid arthritis, 3) revision surgery, 4) history of thrombosis, 5) patients with infection, 6) allergy to TXA, 7) uncontrolled hypertension, 8) body mass index (BMI) > 35 kg/m^2^, 9) preoperative anaemia (haemoglobin (Hb) level < 12 g/dL for women and < 13 g/dL for men) **If TKR, is tourniquet used:** NA **Indication for surgery:** osteoarthritis (OA) or osteonecrosis of the femoral head (ONFH) **Type of anaesthetic:** general **Type of surgery:** primary THR	
Interventions	**Intervention characteristics** TXA, IV In the IV TXA group, 1 g of TXA was administered 10 min before the incision, and the same dose was repeated 3 h and 6 h postoperatively. Patients in the IV TXA group were given 2 placebo tablets (with no active component) orally 2 h before the incision, and the same dose was repeated 3 h and 6 h postoperatively. TXA, IV, 1 g, intraop (10 min before incision) + TXA, IV, 3 + 6 hours postop + placebo, oral, 2 hours preop + placebo, oral, 3 + 6 hours postop TXA, oral In the oral TXA group, 1 g of TXA (2 tablets of 500 mg) was given 2 h before the incision, and the same dose was repeated 3 h and 6 h postoperatively. Additionally, to support this double‐blind study, patients in the oral TXA group were given 100 mL of normal saline solution intravenously 10 min before the incision, and the same dose was repeated 3 h and 6 h postoperatively. Placebo, IV, intraop (10 min before incision) + placebo, IV, 3 + 6 hours postop + TXA, oral, 1 g, 2 hours preop + TXA, oral, 1 g, 3 + 6 hours postop	
Outcomes	*Primary outcomes:* Total blood loss Maximum Hb drop TXA cost (¥, RMB) Transfusion rates Transfusion units *Secondary outcomes:* Hb level on postoperative days 1 and 3 Operating time Hospital length of stay DVT and/or PE Wound complications	
Notes	**Sponsorship source:** non‐pharmaceutical **Country:** China **Setting:** single‐centre **Comments:** none **Author's name:** B Shen **Institution:** Sichuan University **Email:** shenbin_1971@163.com **Address:** Department of Orthopaedics, West China hospital, Sichuan University, 37# Guoxue Road, Chengdu, 610041, China **Native language of paper:** English **Reference type:** full text (1), trial registration (1) **Trial registration number:** ChiCTR1800015265 **Was it translated for this review:** no	
***Risk of bias***		
**Bias**	**Authors' judgement**	**Support for judgement**
Random sequence generation (selection bias)	Low risk	Quote: "Computer‐generated random number tables were used to generate the stratified randomization schedule." Judgement comment: computer‐generated randomisation.
Allocation concealment (selection bias)	Unclear risk	Quote: "Patients were allocated randomly into oral TXA or IV TXA groups, and randomization was blinded and performed with the use of sealed envelopes at a ratio of 1:1 to be opened just prior to surgery." Judgement comment: unclear whether sequentially numbered, opaque envelopes used.
Blinding of participants and personnel (performance bias) Subjective outcomes	Low risk	Quote: "All drugs were administered by a nurse who was not involved in the study. The patients, investigators, and statisticians were blind during the study." Judgement comment: intervention regimen identical in both arms, unlikely blinding could have been broken.
Blinding of participants and personnel (performance bias) Objective outcomes	Low risk	Quote: "All drugs were administered by a nurse who was not involved in the study. The patients, investigators, and statisticians were blind during the study." Judgement comment: objective outcome for personnel, so low risk of bias regardless of quality of blinding.
Blinding of outcome assessment (detection bias) Subjective outcomes	Low risk	Quote: "All drugs were administered by a nurse who was not involved in the study. The patients, investigators, and statisticians were blind during the study." Judgement comment: blinding of outcome assessors ensured.
Blinding of outcome assessment (detection bias) Objective outcomes	Low risk	Quote: not applicable Judgement comment: objective outcome for outcome assessor, so low risk of bias regardless of quality of blinding.
Incomplete outcome data (attrition bias) All outcomes	Low risk	Quote: not applicable Judgement comment: all outcome data presented.
Selective reporting (reporting bias)	Low risk	Quote: not applicable Judgement comment: all pre‐specified outcomes reported in trial registration reported in full text.
Other bias	Low risk	Quote: not applicable Judgement comment: paper appears to be free of other biases.

**Table:** Xie 2016

***Study characteristics***		
Methods	**Study design:** RCT **Intention‐to‐treat analysis:** yes **Duration of study:** 8 months **Power calculation reached:** yes **Transfusion strategy:** the criterion for a blood transfusion was set as a haemoglobin level of < 70 g/L or 70 to 100 g/L with symptomatic anaemia (defined as light‐headedness, fatigue precluding participation in the therapy, palpitations or shortness of breath, not due to other causes) according to the guidelines by the National Ministry of Health. **Was the trial stopped early:** no	
Participants	**Baseline characteristics** TXA, IV pre‐op + placebo, IV, postop, repeated dose *Age (years) (mean SD)*: 66.2 (9.1) *Ethnicity*: not reported *Gender (males, females)*: 17/50 M (34%); 33/50 F (66%) *Length of surgery (minutes) (mean SD)*: 69.6 (10.1) *Proportion of participants on anticoagulants prior to surgery (n/N, %)*: not reported *Incidence of preoperative anaemia (n/N, %)*: not reported *Co‐morbidities (n/N, %)*: not reported *ASA 1 (n, %)*: 1.7 (0.6) *ASA 2 (n, %)*: not reported *ASA 3 (n, %)*: not reported *ASA 4 (n, %)*: not reported *Number of participants randomised*: 50 *Number of participants receiving treatment*: 50 *Number of participants analysed*: 50 *Dropout rate*: 0/50 (0%) TXA, IV pre‐op + TXA, IV, postop + placebo, IV, postop *Age (years) (mean SD)*: 65.3 (8.8) *Ethnicity*: not reported *Gender (males, females)*: 14/50 M (28%); 36/50 F (72%) *Length of surgery (minutes) (mean SD)*: 72.6 (12.1) *Proportion of participants on anticoagulants prior to surgery (n/N, %)*: not reported *Incidence of preoperative anaemia (n/N, %)*: not reported *Co‐morbidities (n/N, %)*: not reported *ASA 1 (n, %)*: 1.9 (0.4) *ASA 2 (n, %)*: not reported *ASA 3 (n, %)*: not reported *ASA 4 (n, %)*: not reported *Number of participants randomised*: 50 *Number of participants receiving treatment*: 50 *Number of participants analysed*: 50 *Dropout rate*: 0/50 (0%) TXA, IV pre‐op + TXA, IV, postop, repeated dose *Age (years) (mean SD)*: 61.5 (7.6) *Ethnicity*: not reported *Gender (males, females)*: 10/51 M (20%); 41/51 F (80%) *Length of surgery (minutes) (mean SD)*: 72.4 (8.4) *Proportion of participants on anticoagulants prior to surgery (n/N, %)*: not reported *Incidence of preoperative anaemia (n/N, %)*: not reported *Co‐morbidities (n/N, %)*: not reported *ASA 1 (n, %)*: 1.7 (0.5) *ASA 2 (n, %)*: not reported *ASA 3 (n, %)*: not reported *ASA 4 (n, %)*: not reported *Number of participants randomised*: 51 *Number of participants receiving treatment*: 51 *Number of participants analysed*: 51 *Dropout rate*: 0/51 (0%) Overall *Age (years) (mean SD)*: not reported *Ethnicity*: not reported *Gender (males, females)*: not reported *Length of surgery (minutes) (mean SD)*: not reported *Proportion of participants on anticoagulants prior to surgery (n/N, %)*: not reported *Incidence of preoperative anaemia (n/N, %)*: not reported *Co‐morbidities (n/N, %)*: not reported *ASA 1 (n, %)*: mean reported only *ASA 2 (n, %)*: not reported *ASA 3 (n, %)*: not reported *ASA 4 (n, %)*: not reported *Number of participants randomised*: 151 *Number of participants receiving treatment*: 151 *Number of participants analysed*: 151 *Dropout rate*: 0/151 (0%) **Inclusion criteria:** 1) aged 18 years or older, 2) scheduled to have primary unilateral TKA for osteoarthritis or rheumatoid arthritis **Exclusion criteria:** 1) patients with anaemia (< 120 g/L for female, < 130 g/L for male), 2) cardiovascular problems (history of myocardial infraction, angina, and atrial fibrillation), 3) cerebrovascular conditions (history of previous stroke), 4) thromboembolic disorders (history of deep vein thrombosis (DVT) or pulmonary embolism (PE)), 5) clotting disorders, 6) known allergy to TXA, 7) flexion deformity, varus and/or valgus deformity **If TKR, is tourniquet used:** no **Indication for surgery:** osteoarthritis or rheumatoid arthritis **Type of anaesthetic:** mixed general and spinal **Type of surgery:** primary TKR	
Interventions	**Intervention characteristics** TXA, IV pre‐op + placebo, IV, postop, repeated dose *TXA, IV, 2 g, preop:* group A: the patients received 1 bolus of 20 mg/kg IV TXA before skin incision, and 10 mg/kg of normal saline was intravenously injected 3 and 6 hours later *TXA, IA, 2 g, intraop:* TXA, IV, 20 mg/kg, intraop (5 to 10 mins prior to skin incision) AND TXA, IA, 1 g, intraop (irrigated after cementing) AND placebo, IV, postop (3 + 6 hours after 1st dose) TXA, IV pre‐op + TXA, IV, postop + placebo, IV, postop *TXA, IV, 2 g, preop:* group B: a dosage of 20 mg/kg IV TXA was administered 5 to 10 minutes before skin incision, and 10 mg/kg of TXA was administered 3 hours later; another 10 mg/kg of normal saline was administered 6 hours later *TXA, IA, 2 g, intraop:* TXA, IV, 20 mg/kg, intraop (5 to 10 mins prior to skin incision) AND TXA, IA, 1 g, intraop (irrigated after cementing) AND TXA, IV, 10 mg/kg, postop (3 hours 1st dose) AND placebo, IV, postop (6 hours after 1st dose) TXA, IV pre‐op + TXA, IV, postop, repeated dose *TXA, IV, 2 g, preop:* group C: a dosage of 20 mg/kg of IV TXA was administered 5 to 10 minutes before skin incision, and 10 mg/kg of TXA was administered 3 and 6 hours later *TXA, IA, 2 g, intraop:* TXA, IV, 20 mg/kg, intraop (5 to 10 mins prior to skin incision) AND TXA, IA, 1 g, intraop (irrigated after cementing) AND TXA, IV, 10 mg/kg, postop (3 + 6 hours after 1st dose)	
Outcomes	*sPrimary outcome:* Hidden blood loss Maximum haemoglobin drop *Secondary outcomes:* Total blood loss Transfusion rate Inflammation markers (C‐reactive protein, interleukin 6) Visual analogue scale pain score Limb swelling ratio Hospital for Surgery score Range of motion Length of hospital stay (LOH) Deep venous thrombosis	
Notes	**Sponsorship source:** non‐pharmaceutical **Country:** China **Setting:** single‐centre **Comments:** none **Author's name:** J Xie **Institution:** Schian University **Email:** not reported **Address:** Fuxing Pei, MD, Department of Orthopaedic Surgery, West China Hospital, Sichuan University, 37# Guoxue Road, Chengdu, Sichuan 610041, People's Republic of China **Native language of paper:** English **Reference type:** full text (1) trial registration (1) **Trial registration number:** ChiCTR‐INR‐16009288 **Was it translated for this review:** no	
***Risk of bias***		
**Bias**	**Authors' judgement**	**Support for judgement**
Random sequence generation (selection bias)	Unclear risk	Quote: "The recruited patients in this double‐blinded study were randomized into 1 of 3 groups" Judgement comment: insufficient information to permit judgement.
Allocation concealment (selection bias)	Unclear risk	Quote: "opaque sealed envelopes only opened before surgery." Judgement comment: not clear whether consecutively numbered.
Blinding of participants and personnel (performance bias) Subjective outcomes	Low risk	Quote: "The postoperative protocol was implemented by a nurse who was not involved in this trial. The patients, surgeons, data collector, and analyst were blinded." Judgement comment: blinding of key personnel ensured.
Blinding of participants and personnel (performance bias) Objective outcomes	Low risk	Quote: not applicable Judgement comment: objective outcome for personnel, so low risk of bias regardless of quality of blinding.
Blinding of outcome assessment (detection bias) Subjective outcomes	Low risk	Quote: "The postoperative protocol was implemented by a nurse who was not involved in this trial. The patients, surgeons, data collector, and analyst were blinded." Judgement comment: blinding of key personnel ensured.
Blinding of outcome assessment (detection bias) Objective outcomes	Low risk	Quote: not applicable Judgement comment: objective outcome for outcome assessor, so low risk of bias regardless of quality of blinding.
Incomplete outcome data (attrition bias) All outcomes	Low risk	Quote: not applicable Judgement comment: all outcome data reported.
Selective reporting (reporting bias)	Low risk	Quote: not applicable Judgement comment: all outcome measures reported from trial registration.
Other bias	Low risk	Quote: not applicable Judgement comment: the paper appears to be free of other bias.

**Table:** Xie 2017

***Study characteristics***		
Methods	**Study design:** RCT **Intention‐to‐treat analysis:** yes **Duration of study:** 5 + 3 months FU = 8 months **Power calculation reached:** yes **Transfusion strategy:** criteria for a blood transfusion were: Hb < 70 g/L or between 70g/L and 100 g/L in the presence of symptomatic anaemia, defined as light‐headedness, fatigue that precluded participation in treatment, palpitations or shortness of breath not due to other causes. **Was the trial stopped early:** no	
Participants	**Baseline characteristics** TXA, IV pre‐op + TXA, IA intraop *Age (years) (mean SD)*: 57.24 (13.05) *Ethnicity*: not reported *Gender (males, females)*: 22/50 M (44%); 28/50 F (56%) *Length of surgery (minutes) (mean SD)*: 66.72 (7.45) *Proportion of participants on anticoagulants prior to surgery (n/N, %)*: not reported *Incidence of preoperative anaemia (n/N, %)*: not reported *Co‐morbidities (n/N, %)*: not reported *ASA 1 (n/N, %)*: 33/50 (66%) *ASA 2 (n/N, %)*: 17/50 (34%) *ASA 3 (n/N, %)*: 0/50 (0%) *ASA 4 (n/N, %)*: 0/50 (0%) *Number of participants randomised*: 50 *Number of participants receiving treatment*: 50 *Number of participants analysed*: 50 *Dropout rate*: 0/50 (0%) TXA, IV pre‐op + TXA, IA intraop + TXA, IV, postop *Age (years) (mean SD)*: 54.96 (12.57) *Ethnicity*: not reported *Gender (males, females)*: 18/50 M (36%); 32/50 F (64%) *Length of surgery (minutes) (mean SD)*: 72.72 (13.59) *Proportion of participants on anticoagulants prior to surgery (n/N, %)*: not reported *Incidence of preoperative anaemia (n/N, %)*: not reported *Co‐morbidities (n/N, %)*: not reported *ASA 1 (n/N, %)*: 28/50 (56%) *ASA 2 (n/N, %)*: 22/50 (44%) *ASA 3 (n/N, %)*: 0/50 (0%) *ASA 4 (n/N, %)*: 0/50 (0%) *Number of participants randomised*: 50 *Number of participants receiving treatment*: 50 *Number of participants analysed*: 50 *Dropout rate*: 0/50 (0%) TXA, IV pre‐op + TXA, IA intraop + TXA, IV, postop, repeated dose *Age (years) (mean SD)*: 54.38 (11.80) *Ethnicity*: not reported *Gender (males, females)*: 20/50 M (40%); 30/50 F (60%) *Length of surgery (minutes) (mean SD)*: 69.99 (12.52) *Proportion of participants on anticoagulants prior to surgery (n/N, %)*: not reported *Incidence of preoperative anaemia (n/N, %)*: not reported *Co‐morbidities (n/N, %)*: not reported *ASA 1 (n/N, %)*: 30/50 (60%) *ASA 2 (n/N, %)*: 20/50 (40%) *ASA 3 (n/N, %)*: 0/50 (0%) *ASA 4 (n/N, %)*: 0/50 (0%) *Number of participants randomised*: 50 *Number of participants receiving treatment*: 50 *Number of participants analysed*: 50 *Drop out rate*: 0/50 (0%) Overall *Age (years) (mean SD)*: not reported *Ethnicity*: not reported *Gender (males, females)*: not reported *Length of surgery (minutes) (mean SD)*: not reported *Proportion of participants on anticoagulants prior to surgery (n/N, %)*: not reported *Incidence of preoperative anaemia (n/N, %)*: not reported *Co‐morbidities (n/N, %)*: not reported *ASA 1 (n/N, %)*: not reported *ASA 2 (n/N, %)*: not reported *ASA 3 (n/N, %)*: not reported *ASA 4 (n/N, %)*: not reported *Number of participants randomised*: 150 *Number of participants receiving treatment*: 150 *Number of participants analysed*: 150 *Drop out rate*: 0/150 (0%) **Inclusion criteria:** 1) aged > 18 years, 2) scheduled for primary unilateral THA for osteoarthritis or osteonecrosis of the femoral head **Exclusion criteria:** 1) cardiovascular disease (including myocardial infarction, angina or atrial fibrillation), 2) cerebrovascular pathology (a history of stroke), 3) thromboembolic disorders (a history of deep vein thrombosis (DVT) or pulmonary embolism (PE)), 4) an allergy to TXA **If TKR, is tourniquet used:** not applicable **Indication for surgery:** osteoarthritis or osteonecrosis of the femoral head **Type of anaesthetic:** general anaesthesia **Type of surgery:** primary THR	
Interventions	**Intervention characteristics** TXA, IV pre‐op + TXA, IA intraop Group A, the control group, received a bolus of 20 mg/kg intravenous (IV) TXA before the incision and 100 mL of normal saline was injected intravenously 3 and 6 hours later. All patients received 1.5 g topical TXA intraoperatively, based on previous work. TXA, IV, 20 mg/kg preop, AND TXA IA, 1.5 g intraop TXA, IV pre‐op + TXA, IA intraop + TXA, IV, postop Group B received 20 mg/kg IV TXA before the incision, 1 g/100 mL TXA 3 hours later and 100 mL of normal saline 6 hours later. All patients received 1.5 g topical TXA intraoperatively, based on previous work. TXA, IV, 20 mg/kg preop, AND TXA IA, 1.5 g, intraop AND TXA, IV, 1 g, 3 hours later AND placebo, IV, 6 hours later TXA, IV pre‐op + TXA, IA intraop + TXA, IV, postop, repeated dose Group C received 20 mg/kg IV TXA before the incision and 1 g/100 mL TXA 3 and 6 hours later. All patients received 1.5 g topical TXA intraoperatively, based on previous work. TXA, IV, 20 mg/kg preop, AND TXA IA, 1.5 g, intraop AND TXA, IV, 1 g, 3 + 6 hours later	
Outcomes	*Primary outcomes:* Hidden blood loss Level of haemoglobin (Hb) Levels of C‐reactive protein (CRP) and interleukin‐6 (IL‐6) *Secondary outcomes:* Length of stay in hospital Incidence of venous thromboembolism (VTE) Total blood loss Rate of transfusion Pain score using a visual analogue scale (VAS) from 1 to 10 Wound complications, including infection, persistent discharge, periprosthetic infection and other adverse events	
Notes	**Sponsorship source:** non‐pharmaceutical **Country:** China **Setting:** single‐centre **Comments:** none **Author's name:** J Xie **Institution:** Sichuan University **Email:** peifuxinghuaxi@126.com **Address:** West China Hospital, Sichuan University, Chengdu, China **Native language of paper:** English **Reference type:** full text (1) trial registration (1) **Trial registration number:** ChiCTR‐INR‐16009288 **Was it translated for this review:** no	
***Risk of bias***		
**Bias**	**Authors' judgement**	**Support for judgement**
Random sequence generation (selection bias)	Unclear risk	Quote: "Patients were allocated randomly into one of three groups that received different numbers of boluses of TXA." Judgement comment: insufficient information to permit judgement.
Allocation concealment (selection bias)	Unclear risk	Quote: "The allocation remained confidential and was revealed to the staff pre‐operatively using opaque, sealed envelopes." Judgement comment: does not include whether these were consecutively numbered.
Blinding of participants and personnel (performance bias) Subjective outcomes	High risk	Quote: "The allocation remained confidential and was revealed to the staff pre‐operatively using opaque, sealed envelopes." Judgement comment: blinding of key personnel not ensured.
Blinding of participants and personnel (performance bias) Objective outcomes	Low risk	Quote: not applicable Judgement comment: objective outcome for personnel, so low risk of bias regardless of quality of blinding.
Blinding of outcome assessment (detection bias) Subjective outcomes	Low risk	Quote: "The postoperative protocol was implemented by a nurse not involved in the trial" Judgement comment: blinding of outcome assessor described.
Blinding of outcome assessment (detection bias) Objective outcomes	Low risk	Quote: not applicable Judgement comment: objective outcome for outcome assessor, so low risk of bias regardless of quality of blinding.
Incomplete outcome data (attrition bias) All outcomes	Low risk	Quote: not applicable Judgement comment: all outcome data reported.
Selective reporting (reporting bias)	High risk	Quote: "The original protocol was registered on the Chinese Clinical Trial Registry (ChiCTR‐INR‐16009288) but the present study differs from that protocol in two respects. First, the number of intervention groups was reduced from four to three. The three groups were treated with between one and three boluses of TXA and there was no group treated with five boluses, as was originally planned. This change was made with the approval of the monitoring committee following analysis of a pilot study in which a five‐bolus regime was found to require waking patients at midnight. Since this would mean waking all groups at this time in order to administer placebo or TXA to preserve blinding of group allocation, it might have detrimental effects on the patients. The second deviation is that the protocol stipulated primary outcomes of HBL, inflammation, the decrease in the level of Hb and fibrinolysis. Based on a previous kinetics study, 15 in our pilot study involving 40 THAs (data not shown), D‐dimer and fibrin degradation products were only used to detect the trend and duration of hyper‐ fibrinolysis in THA. We found that hyper‐fibrinolysis peaked at six hours post‐operatively and lasted for at least 24 hours. Therefore, we collected primary outcomes data only on HBL, the level of Hb and inflammation." Judgement comment: despite justification for two protocol deviations, trial registration also listed in another paper published in the Journal of Arthroplasty by the same group. Inconsistent reporting. In addition, swelling not reported as an outcome listed in protocol.
Other bias	Low risk	Quote: not applicable Judgement comment: paper appears to be free of other bias.

**Table:** Xu 2023

***Study characteristics***		
Methods	**Study design:** RCT **Intention‐to‐treat analysis:** not reported ‐ 6/168 participants dropped out after randomisation **Duration of study:** September 2019 to August 2020 + 3 months follow‐up = 14 months **Power calculation reached:** yes **Transfusion strategy:** transfusion was performed if the patient’s condition met the criteria established by the Chinese National Ministry of Health: 1) if haemoglobin level was < 70 g/L; 2) or if haemoglobin level was between 70 and 100 g/L and the patient showed dizziness, palpitations, fatigue or other obvious symptoms of anaemia. **Was the trial stopped early:** no	
Participants	**Baseline characteristics** TXA IV 60 mg/kg, preop; TXA IV 1 g x 5 postop *Age (years) (mean SD)*: Group A: 69.42 (6.05); Group C: 67.59 (7.52) *Ethnicity*: not reported *Gender (males, females)*: 12/82 M (14.6%); 70/82 F (85.4%) *Length of surgery (minutes) (mean SD)*: Group A: 75.11 (8.18); Group C: 76.84 (10.41) *Proportion of participants on anticoagulants prior to surgery (n/N, %)*: excluded *Incidence of preoperative anaemia (n/N, %)*: not reported *Co‐morbidities (n/N, %)*: hypertension: 27/82 (32.9%); diabetes: 10/82 (12.2%); COPD: 1/82 (1.2%); hypothyroidism: 2/82 (2.4%); history of cardiac surgery: 2/82 (2.4%) *ASA 1 (n/N, %)*: not reported *ASA 2 (n/N, %)*: 53/82 (64.6%) *ASA 3 (n/N, %)*: 29/82 (35.4%) *ASA 4 (n/N, %)*: not reported *Number of participants randomised*: 84 *Number of participants receiving treatment*: 84 *Number of participants analysed*: 82 *Dropout rate*: 2/84 (2.4%) TXA IV 60 mg/kg, preop; TXA IV 1 g x 8 postop *Age (years) (mean SD)*: Group B: 68.40 (6.59); Group D: 67.03 (8.23) *Ethnicity*: not reported *Gender (males, females)*: 11/80 M (13.8%); 39/80 F (86.3%) (*11 + 39 does not equal 80; the author has been emailed for clarification but no response as yet 17 January 2023) *Length of surgery (minutes) (mean SD)*: Group B: 75.56 (8.34); Group D: 75.83 (8.67) *Proportion of participants on anticoagulants prior to surgery (n/N, %)*: excluded *Incidence of preoperative anaemia (n/N, %)*: not reported *Co‐morbidities (n/N, %)*: hypertension: 27/80 (33.8%); diabetes: 8/80 (10%); COPD: 5/80 (6.3%); Hypothyroidism: 1/80 (1.3%); History of cardiac surgery: 2/80 (2.5%) *ASA 1 (n/N, %)*: not reported *ASA 2 (n/N, %)*: 47/80 (58.8%) *ASA 3 (n/N, %)*: 33/80 (41.3%) *ASA 4 (n/N, %)*: not reported *Number of participants randomised*: 84 *Number of participants receiving treatment*: 84 *Number of participants analysed*: 80 *Dropout rate*: 4/84 (4.8%) Overall *Age (years) (mean SD)*: 68.11 (7.1) *Ethnicity*: not reported *Gender (males, females)*: 23/162 M (14.2%); 139/162 F (85.8%) *Length of surgery (minutes) (mean SD)*: 75.87 (9.0) *Proportion of participants on anticoagulants prior to surgery (n/N, %)*: excluded *Incidence of preoperative anaemia (n/N, %)*: not reported *Co‐morbidities (n/N, %)*: hypertension: 54/162 (33.3%); diabetes: 18/162 (11.1%); COPD: 6/162 3.7%; hypothyroidism: 3/162 (1.9%); history of cardiac surgery: 4/162 (2.5%) *ASA 1 (n/N, %)*: not reported *ASA 2 (n/N, %)*: 100/162 (61.7%) *ASA 3 (n/N, %)*: 62/162 (38.3%) *ASA 4 (n/N, %)*: not reported *Number of participants randomised*: 168 *Number of participants receiving treatment*: 168 *Number of participants analysed*: 162 *Dropout rate*: 6/168 (3.6%) **Inclusion criteria:** patients who underwent primary unilateral TKA for osteoarthritis between September 2019 and August 2020 were screened for enrolment. **Exclusion criteria:** patients were excluded from the study if their TKA was a revision or bilateral, if they were younger than 18 years or older than 80 years, if they were allergic to TXA or DXM, or if they were using anticoagulants, had a history of myocardial infarction, angina, stroke, severe renal or liver failure, deep vein thrombosis (DVT), pulmonary embolism (PE) or inflammatory disease. **If TKR, is tourniquet used:** no **Indication for surgery:** osteoarthritis **Type of anaesthetic:** general **Type of surgery:** primary unilateral TKA	
Interventions	**Intervention characteristics** TXA IV 60 mg/kg, preop; TXA IV 1 g x 5 postop (A + C) Group A (control group) received 60 mg/kg TXA intravenously at 5 to 10 minutes before skin incision, followed by 5 doses of 1 g TXA at 3, 6, 12, 18 and 24 hours after the initial dose. This group also received 20 mg DXM intravenously during induction of anaesthesia. Group C received the same treatment as group A, as well as 10 mg DXM at 08:00 to 09:00 hours on postoperative day (POD) 1 and 5 mg DXM at the same time on POD 2. TXA IV 60 mg/kg, preop; TXA IV 1 g x 5 postop TXA IV 60 mg/kg, preop; TXA IV 1 g x 8 postop (B + D) Group B received the same treatment as group A, as well as 3 doses of 1 g TXA at 36, 48 and 60 hours after the initial dose. AND Group D received all the treatments given to groups B and C. DXM was given at 08:00 to 09:00 hours to groups C to D, because giving the drug at the nadir of the patient’s secretion of endogenous adrenocortical hormone may inhibit adrenocortical function the next day. TXA IV 60 mg/kg, preop; TXA IV 1 g x 8 postop	
Outcomes	*Primary outcomes:* Fibrinolytic and inflammatory markers Knee function Postoperative pain levels Consumption of opioids *Secondary outcomes:* Blood loss Maximal drop in haemoglobin Coagulation Fasting blood glucose (FBG) Complications	
Notes	**Sponsorship source:** non‐pharmaceutical ‐ "The research was supported by Post‐Doctor Research Project, West China Hospital, Sichuan University (2018HXBH073); National Clinical Research Center for Geriatrics, West China Hospital, Sichuan University (Z2018B11)." **Setting:** single‐centre **Comments:** 1) interventions have been combined; we have extracted group A + group C vs group B + group D **Author's name:** H Xu **Institution:** West China Hospital, Sichuan University **Email:** corresponding author: Fuxing Pei ‐ peifuxingwestchina@163.com **Address:** Department of Orthopaedic Surgery, West China Hospital, Sichuan University, Chengdu 610041, China **Native language of paper:** English **Reference type:** full text (1) **Trial registration number:** ChiCTR1900026092 **Was it translated for this review:** no	
***Risk of bias***		
**Bias**	**Authors' judgement**	**Support for judgement**
Random sequence generation (selection bias)	Low risk	Quote: "Enrolled patients were randomized by computer into one of four groups." Judgement comment: adequate method of sequence generation with computer‐generated code.
Allocation concealment (selection bias)	Low risk	Quote: "Enrolled patients were randomized by computer into one of four groups" Judgement comment: adequate method of allocation concealment using computer‐generated code.
Blinding of participants and personnel (performance bias) Subjective outcomes	Unclear risk	Quote: "Patients, surgeons, data collectors, and analysts remained blinded to patient allocation throughout the study, whereas the attending physician was told of the allocation after surgery." Judgement comment: attending physician was unblinded. Unclear what effect this had on the study.
Blinding of participants and personnel (performance bias) Objective outcomes	Low risk	Quote: not applicable Judgement comment: objective outcome for personnel, so low risk of bias regardless of quality of blinding.
Blinding of outcome assessment (detection bias) Subjective outcomes	Low risk	Quote: "Patients, surgeons, data collectors, and analysts remained blinded to patient allocation throughout the study, whereas the attending physician was told of the allocation after surgery." Judgement comment: outcome assessors were blinded
Blinding of outcome assessment (detection bias) Objective outcomes	Low risk	Quote: not applicable Judgement comment: objective outcome for outcome assessor, so low risk of bias regardless of quality of blinding.
Incomplete outcome data (attrition bias) All outcomes	Unclear risk	Quote: not applicable Judgement comment: 6 participants dropped out after randomisation. Reasons for this are not described.
Selective reporting (reporting bias)	Low risk	Quote: not applicable Judgement comment: all outcomes specified in the trial registration were reported in the paper.
Other bias	Low risk	Quote: not applicable Judgement comment: no other sources of bias.

**Table:** Xue 2021

***Study characteristics***		
Methods	**Study design:** RCT **Intention‐to‐treat analysis:** yes **Duration of study:** March to August 2020 (5 months) + 4 weeks follow‐up = 6 months **Power calculation reached:** yes. Recruited slightly fewer than their target population but experienced fewer dropouts: " On the basis of the outcomes of the previous study and total Hb loss, 47 patients per group were needed to detect a difference of 1 g/dL of Hb, with a significance level of 0.05 and a power of 0.90. Considering unexpected protocol violations and a certain dropout rate,56 patients per group were officially registered in the study." **Transfusion strategy:** "Blood transfusions were performed according to the management guidelines published by the Ministry of Health of China, which guided perioperative transfusion. Based on these guidelines, when the hemoglobin was less than 7 g/dL, blood transfusion was required. It was still required if the patient manifested symptomatic anemia, such as light‐headedness, fatigue, shortness of breath, or palpitations with the hemoglobin within 7–9 g/dL" **Was the trial stopped early:** met > 90% target recruitment of 56 in each group, but unclear why the trial did not recruit the remaining participants	
Participants	**Baseline characteristics** TXA, IV 2 x 15 mg/kg postop *Age (years) (mean SD)*: 66.91 (3.33) *Ethnicity*: not reported *Gender (males, females)*: 13/53 (24.5%) M, 40/53 (75.5%) F *Length of surgery (minutes) (mean SD)*: 56.53 (9.19) *Proportion of participants on anticoagulants prior to surgery (n/N, %)*: excluded *Incidence of preoperative anaemia (n/N, %)*: excluded *Co‐morbidities (n/N, %)*: not reported *ASA 1 (n/N, %)*: 0/53, 0% *ASA 2 (n/N, %)*: 45/53, 84.9% *ASA 3 (n/N, %)*: 8/53, 15.1% *ASA 4 (n/N, %)*: 0/53, 0% *Number of participants randomised*: 53 *Number of participants receiving treatment*: 53 *Number of participants analysed*: 53 *Dropout rate*: 0/53, 0% TXA, IV 1 x 15 mg/kg postop *Age (years) (mean SD)*: 66.18 (3.52) *Ethnicity*: not reported *Gender (males, females)*: 11/50 (22%) M, 39/50 (78%) F *Length of surgery (minutes) (mean SD)*: 56.46 (8.67) *Proportion of participants on anticoagulants prior to surgery (n/N, %)*: excluded *Incidence of preoperative anaemia (n/N, %)*: excluded *Co‐morbidities (n/N, %)*: not reported *ASA 1 (n/N, %)*: 0/50, 0% *ASA 2 (n/N, %)*: 43/50, 86% *ASA 3 (n/N, %)*: 7/50, 14% *ASA 4 (n/N, %)*: 0/50, 0% *Number of participants randomised*: 50 *Number of participants receiving treatment*: 50 *Number of participants analysed*: 50 *Dropout rate*: 0/50, 0% Placebo *Age (years) (mean SD)*: 67.06 (3.05) *Ethnicity*: not reported *Gender (males, females)*: 14/53 (26.4%) M, 39/53 (73.6%) F *Length of surgery (minutes) (mean SD)*: 55.62 (8.99) *Proportion of participants on anticoagulants prior to surgery (n/N, %)*: excluded *Incidence of preoperative anaemia (n/N, %)*: excluded *Co‐morbidities (n/N, %)*: not reported *ASA 1 (n/N, %)*: 0/53, 0% *ASA 2 (n/N, %)*: 46/53, 86.8% *ASA 3 (n/N, %)*: 7/53, 13.2% *ASA 4 (n/N, %)*: 0/53, 0% *Number of participants randomised*: 53 *Number of participants receiving treatment*: 53 *Number of participants analysed*: 53 *Dropout rate*: 0/53, 0% Overall *Age (years) (mean SD)*: not reported *Ethnicity*: not reported *Gender (males, females)*: 38/156 (24%) M, 118/156 (76%) F *Length of surgery (minutes) (mean SD)*: data not reported overall *Proportion of participants on anticoagulants prior to surgery (n/N, %)*: excluded *Incidence of preoperative anaemia (n/N, %)*: excluded *Co‐morbidities (n/N, %)*: not reported *ASA 1 (n/N, %)*: 0/156, 0% *ASA 2 (n/N, %)*: 134/156, 86% *ASA 3 (n/N, %)*: 22/156, 14% *ASA 4 (n/N, %)*: 0/156, 0% *Number of participants randomised*: 156 *Number of participants receiving treatment*: 156 *Number of participants analysed*: 156 *Dropout rate*: 0/156, 0% **Inclusion criteria:** 1) patients who were diagnosed with knee arthritis, aged over 55; 2) patients planning to accept primary unilateral TKA; and 3) no obvious knee deformity; the pre‐operative knee deformity criteria were: i) valgus deformity less than 10 degrees and varus deformity less than 20 degrees and ii) flexion more than 90 degrees and lack of extension less than 10 degrees. Furthermore, the coagulation profile had to be normal. **Exclusion criteria:** 1) abnormal fibrinolytic system, coagulopathy or long‐term anticoagulant therapy; 2) history of thromboembolic disease or diagnosed with deep vein thrombosis (DVT); 3) patients with anaemia (< 12 g/dL for male, < 11 g/dL for female); 4) a known history of cerebral vascular disease and cardiovascular disease; 5) placement of a coronary or vascular stent within the past 12 months; 6) complaining of hepatic or renal dysfunction; and 7) significantly increased inflammatory markers or infection **If TKR, is tourniquet used:** yes **Indication for surgery:** knee arthritis **Type of anaesthetic:** general **Type of surgery:** primary unilateral TKA	
Interventions	**Intervention characteristics** TXA, IV 2 x 15 mg/kg postop Group A: a single dose of 15 mg/kg intravenous TXA in 250 mL normal saline 2 h after closing the incision, and a second dose of intravenous TXA in 250 mL normal saline 24 h after surgery TXA, IV, 15 mg/kg, intraop + TXA, IV, 15 mg/kg, postop TXA, IV 1 x 15 mg/kg postop Group B: a single dose of 15 mg/kg intravenous TXA in 250 mL normal saline 2 h after closing the incision, and a second dose of intravenous 250 mL normal saline without TXA 24 h after surgery TXA, IV, 15 mg/kg, intraop + placebo, IV, 250 mL, postop Placebo Group C: double dose of intravenous 250 mL normal saline without TXA 2 and 24 h after closing the incision Placebo, IV, 250 mL, postop (double dose)	
Outcomes	*Primary outcomes:* Hidden blood loss Total blood loss *Secondary outcomes:* Haematocrit DVT	
Notes	**Sponsorship source:** non‐pharmaceutical **Country:** China **Setting:** single‐centre **Comments:** none **Author's name:** C‐X Xue **Institution:** Second Affiliated Hospital of Anhui Medical University **Email:** corresponding author: J‐H Jing ‐ jhjpaper@sina.com **Address:** The Second Affiliated Hospital of Anhui Medical University in Hefei City, Anhui Province, China 230000 **Native language of paper:** English **Reference type:** full text (1) **Trial registration number:** ChiCTR2000039368 **Was it translated for this review:** no	
***Risk of bias***		
**Bias**	**Authors' judgement**	**Support for judgement**
Random sequence generation (selection bias)	Low risk	Quote: "Patients are enrolled by nurses who are not involved in the study using a computer‐generated randomised sequence on a scale of 1:1:1." Judgement comment: method of sequence generation not described.
Allocation concealment (selection bias)	Low risk	Quote: "Two nurses who were not involved in the study implemented the post‐operative protocol and dispensed the medications." Judgement comment: adequate method of allocation concealment, using independent personnel.
Blinding of participants and personnel (performance bias) Subjective outcomes	Unclear risk	Quote: "Two nurses who were not involved in the study implemented the post‐operative protocol and dispensed the medications. All of the surgeons, data collector and analyst, and patients were blinded" Judgement comment: personnel and participants were blinded.
Blinding of participants and personnel (performance bias) Objective outcomes	Low risk	Quote: not applicable Judgement comment: objective outcome for personnel, so low risk of bias regardless of quality of blinding.
Blinding of outcome assessment (detection bias) Subjective outcomes	Unclear risk	Quote: "Two nurses who were not involved in the study implemented the post‐operative protocol and dispensed the medications. All of the surgeons, data collector and analyst, and patients were blinded" Judgement comment: outcome assessors were blinded.
Blinding of outcome assessment (detection bias) Objective outcomes	Low risk	Quote: not applicable Judgement comment: objective outcome for outcome assessor, so low risk of bias regardless of quality of blinding.
Incomplete outcome data (attrition bias) All outcomes	Low risk	Quote: not applicable Judgement comment: no obvious outcome data missing.
Selective reporting (reporting bias)	Unclear risk	Quote: not applicable Judgement comment: measurement of haemoglobin and haematocrit are specified as outcome measures in the trial registration. However, in the paper the raw values are not presented but combined into a calculation of Hb drop and Hct change. Raw values cannot be recalculated without knowing individual participant's total blood volume.
Other bias	Low risk	Quote: not applicable Judgement comment: no other significant sources of bias.

**Table:** Yamasaki 2004

***Study characteristics***		
Methods	**Study design:** RCT **Intention‐to‐treat analysis:** yes **Duration of study:** 4 months (3 months + 1 months FU) **Power calculation reached:** not reported **Transfusion strategy:** not reported **Was the trial stopped early:** no	
Participants	**Baseline characteristics** Placebo *Age (years) (mean SD)*: 61.2 (6.9) *Ethnicity*: not reported *Gender (males, females)*: 18/20 M (90%); 2/20 F (10%) *Length of surgery (minutes)*: 70.0 (14.8) *Proportion of participants on anticoagulants prior to surgery (n/N, %)*: not reported *Incidence of preoperative anaemia (n/N, %)*: not reported *Co‐morbidities (n/N, %)*: not reported *ASA 1 (n/N, %)*: not reported *ASA 2 (n/N, %)*: not reported *ASA 3 (n/N, %)*: not reported *ASA 4 (n/N, %)*: not reported *Number of participants randomised*: 20 *Number of participants receiving treatment*: 20 *Number of participants analysed*: 20 *Dropout rate*: 0/20 (0%) TXA (whole body) *Age (years) (mean SD)*: 55.5 (14.2) *Ethnicity*: not reported *Gender (males, females)*: 19/20 M (95%); 1/20 F (5%) *Length of surgery (minutes)*: 68.8 (15.1) *Proportion of participants on anticoagulants prior to surgery (n/N, %)*: not reported *Incidence of preoperative anaemia (n/N, %)*: not reported *Co‐morbidities (n/N, %): n*ot reported *ASA 1 (n/N, %)*: not reported *ASA 2 (n/N, %)*: not reported *ASA 3 (n/N, %)*: not reported *ASA 4 (n/N, %)*: not reported *Number of participants randomised*: 20 *Number of participants receiving treatment*: 20 *Number of participants analysed*: 20 *Dropout rate*: 0/20 (0%) Overall *Age (years) (mean SD)*: not reported *Ethnicity*: not reported *Gender (males, females)*: not reported *Length of surgery (minutes)*: not reported *Proportion of participants on anticoagulants prior to surgery (n/N, %)*: not reported *Incidence of preoperative anaemia (n/N, %)*: not reported *Co‐morbidities (n/N, %)*: not reported *ASA 1 (n/N, %)*: not reported *ASA 2 (n/N, %)*: not reported *ASA 3 (n/N, %)*: not reported *ASA 4 (n/N, %)*: not reported *Number of participants randomised*: 40 *Number of participants receiving treatment*: not reported *Number of participants analysed*: not reported *Dropout rate*: not reported **Inclusion criteria:** 1) primary THA with osteoarthritis of the hip **Exclusion criteria:** 1) femoral head osteonecrosis, 2) rheumatoid arthritis **If TKR, is tourniquet used:** not applicable **Indication for surgery:** osteoarthritis **Type of anaesthetic:** spinal anaesthetic **Type of surgery:** primary THR	
Interventions	**Intervention characteristics** Placebo The other 20 patients served as a control group and were operated on without tranexamic acid ASSUMED, no text: placebo, IV, 5 mins preop TXA (whole body) In 20 patients, 1000 mg of whole‐body tranexamic acid (Transamin, Daiichi Pharmaceutical Co. Ltd., Japan) was administrated intravenously 5 min before the operation started. TXA, IV, 1 g, 5 mins preop	
Outcomes	*Primary outcomes:* Perioperative and postoperative bleeding Need for transfusion Thromboembolic complications *Secondary outcome:* Not reported	
Notes	**Sponsorship source:** not reported **Country:** Japan **Setting:** single‐centre **Comments:** none **Author's name:** S Yamasaki **Institution:** Osaka Kosei‐Nenkin Hospital **Email:** ys8483@okn.gr.jp **Address:** Department of Orthopaedic Surgery, Osaka Kosei‐Nenkin Hospital,4–2‐78, Fukushima, 553–0003 Fukushimaku, Osaka, Japan **Native language of paper:** English **Reference type:** full text (1) **Trial registration number:** not applicable, but when checked, trial registration number not found **Was it translated for this review:** no	
***Risk of bias***		
**Bias**	**Authors' judgement**	**Support for judgement**
Random sequence generation (selection bias)	Low risk	Quote: "Randomization was carried out by a person not involved in the operation using a ticket drawn from an envelope containing an equal number of tranexamic acid and placebo tickets." Judgement comment: refers to drawing lots.
Allocation concealment (selection bias)	Unclear risk	Quote: not applicable Judgement comment: insufficient information to permit judgement.
Blinding of participants and personnel (performance bias) Subjective outcomes	Unclear risk	Quote: "Randomization was carried out by a person not involved in the operation using a ticket drawn from an envelope containing an equal number of tranexamic acid and placebo tickets. The operating team was unaware of the contents of the solution administered." Judgement comment: insufficient information to permit judgement.
Blinding of participants and personnel (performance bias) Objective outcomes	Low risk	Quote: not applicable Judgement comment: objective outcome for personnel, so low risk of bias regardless of quality of blinding.
Blinding of outcome assessment (detection bias) Subjective outcomes	Unclear risk	Quote: not applicable Judgement comment: no description given of outcome assessor blinding.
Blinding of outcome assessment (detection bias) Objective outcomes	Low risk	Quote: not applicable Judgement comment: objective outcome for outcome assessor, so low risk of bias regardless of quality of blinding.
Incomplete outcome data (attrition bias) All outcomes	Low risk	Quote: not applicable Judgement comment: all outcome data presented.
Selective reporting (reporting bias)	Unclear risk	Quote: not applicable Judgement comment: no protocol available to check pre‐specified outcomes reported.
Other bias	Unclear risk	Quote: not applicable Judgement comment: no description of placebo treatment regimen. Mentions use of placebo once.

**Table:** Yang 2020

***Study characteristics***		
Methods	**Study design:** RCT **Intention‐to‐treat analysis:** yes **Duration of study:** 13 months + follow‐up (length of follow‐up not stated) **Power calculation reached:** no **Transfusion strategy:** allogeneic transfusion was performed when haemoglobin levels were < 7.0 g/dL or when haemoglobin was 7.0 to 9.0 g/dL combined with a poor mental status, palpitation or pallor. **Was the trial stopped early:** no	
Participants	**Baseline characteristics** Placebo *Age (years) (mean SD)*: 64.4 (5.8) *Ethnicity*: not reported *Gender (males, females)*: 13/47 M (28%); 34/47 F (72%) *Length of surgery (minutes)*: not reported *Proportion of participants on anticoagulants prior to surgery (n/N, %)*: not reported *Incidence of preoperative anaemia (n/N, %)*: not reported *Co‐morbidities (n/N, %)*: not reported *ASA 1 (n/N, %)*: not reported *ASA 2 (n/N, %)*: not reported *ASA 3 (n/N, %)*: not reported *ASA 4 (n/N, %)*: not reported *Number of participants randomised*: 48 *Number of participants receiving treatment*: 48 *Number of participants analysed*: 47 *Dropout rate*: 1/48, 2.08% TXA *Age (years) (mean SD)*: 65.2 (6.2) *Ethnicity*: not reported *Gender (males, females)*: 11/47 M (23%); 36/47 F (77%) *Length of surgery (minutes)*: not reported *Proportion of participants on anticoagulants prior to surgery (n/N, %)*: not reported *Incidence of preoperative anaemia (n/N, %)*: not reported *Co‐morbidities (n/N, %)*: not reported *ASA 1 (n/N, %)*: not reported *ASA 2 (n/N, %)*: not reported *ASA 3 (n/N, %)*: not reported *ASA 4 (n/N, %)*: not reported *Number of participants randomised*: 48 *Number of participants receiving treatment*: 48 *Number of participants analysed*: 47 *Dropout rate*: 1/48, 2.08% Overall *Age (years) (mean SD)*: not reported *Ethnicity*: not reported *Gender (males, females)*: not reported *Length of surgery (minutes)*: not reported *Proportion of participants on anticoagulants prior to surgery (n/N, %)*: not reported *Incidence of preoperative anaemia (n/N, %)*: not reported *Co‐morbidities (n/N, %)*: not reported *ASA 1 (n/N, %)*: not reported *ASA 2 (n/N, %)*: not reported *ASA 3 (n/N, %)*: not reported *ASA 4 (n/N, %)*: not reported *Number of participants randomised*: not reported *Number of participants receiving treatment*: not reported *Number of participants analysed*: not reported *Drop out rate*: not reported **Inclusion criteria:** 1) diagnosis of moderate or severe knee OA (grade 3 or 4, based on the degree of erosion); 2) ineffective conservative treatment before undergoing primary TKA **Exclusion criteria:** 1) < 55 years of age; 2) any haemorrhagic blood disease; 3) haemoglobin levels < 10 g/dL; 4) history of thromboembolic disease; 5) rheumatoid arthritis; 6) traumatic arthritis; 7) any disorder of the hips; 8) preoperative use of anticoagulants; or 9) TXA contraindications **If TKR, is tourniquet used:** yes **Indication for surgery:** osteoarthritis **Type of anaesthetic:** spinal anaesthetic **Type of surgery:** primary TKR	
Interventions	**Intervention characteristics** Placebo The patients in the control group had the same volume of physiological saline injected as a placebo, and the affected limb was fully extended. A high‐elasticity bandage was applied to compress the wound in all patients. Placebo, IA, intraop TXA (whole body) In the TXA group, 500 mg of TXA mixed with 50 mL of physiological saline was injected into the knee joint before tourniquet release and after the closure of surgical wounds. Then, the affected knee and hip were flexed at 45° using a folding hospital bed after transporting the patients to the recovery ward; the position was retained for 4 h TXA, IA, 0.5 g, intraop	
Outcomes	*Primary outcome:* Postoperative haemoglobin reduction *Secondary outcomes:* Calculated blood loss (CBL) at 3 days Transfusion rate Range of motion (ROM) VAS pain score Knee circumference increment	
Notes	**Sponsorship source:** not mentioned in full text, but trial registration says it was 'self‐financed' **Country:** China **Setting:** single‐centre **Comments:** none **Author's name:** J‐Q Yang **Institution:** The Third Affiliated Hospital of Southern Medical University **Email:** daozhangcai@163.com **Address:** Department of Orthopaedics, The Third Affiliated Hospital of Southern Medical University, 183 Zhongshan Avenue West, Tianhe District, Guangzhou, China 510630 **Native language of paper:** English **Reference type:** full text (1), trial registration (1) **Trial registration number:** ChiCTR‐INR‐16010287 **Was it translated for this review:** no	
***Risk of bias***		
**Bias**	**Authors' judgement**	**Support for judgement**
Random sequence generation (selection bias)	Unclear risk	Quote: "The patients were randomized 1:1 to the TXA and control groups equally by a third‐party junior doctor" Judgement comment: method of sequence generation for randomisation not described.
Allocation concealment (selection bias)	Low risk	Quote: "sequentially numbered sealed opaque envelopes that were initially prepared by an independent statistician." Judgement comment: adequate method of allocation concealment.
Blinding of participants and personnel (performance bias) Subjective outcomes	High risk	Quote: not applicable Judgement comment: none
Blinding of participants and personnel (performance bias) Objective outcomes	Low risk	Quote: not applicable Judgement comment: objective outcome for personnel, so low risk of bias regardless of quality of blinding.
Blinding of outcome assessment (detection bias) Subjective outcomes	Low risk	Quote: "The postoperative assessments were performed by a surgeon who was blinded to grouping." Judgement comment: subjective outcome for outcome assessors and low risk of bias due to adequate personnel blinding.
Blinding of outcome assessment (detection bias) Objective outcomes	Low risk	Quote: "The postoperative assessments were performed by a surgeon who was blinded to grouping." Judgement comment: objective outcome for outcome assessor, so low risk of bias regardless of quality of blinding.
Incomplete outcome data (attrition bias) All outcomes	Low risk	Quote: not applicable Judgement comment: no obvious outcome data missing.
Selective reporting (reporting bias)	Low risk	Quote: not applicable Judgement comment: all outcomes planned in the protocol or prospective trial registration are reported.
Other bias	Low risk	Quote: not applicable Judgement comment: no other concerns such as early stopping or imbalanced study arms.

**Table:** Yasli 2019

***Study characteristics***		
Methods	**Study design:** RCT **Intention‐to‐treat analysis:** unclear **Duration of study:** 4 months (taken from trial registration) + follow‐up (not reported) **Power calculation reached:** yes **Transfusion strategy:** a HgB level of less than 8 g/dL ‐1 was considered a transfusion trigger except in patients who could have a poor tolerance to these levels because of associated conditions such as chronic obstructive pulmonary disease (COPD), cerebral arterial insufficiency, or patients who presented signs, symptoms or both of hypoxia such as tachycardia, dyspnoea or syncope. The transfusion trigger was placed at less than 10 g/dL for these patients. **Was the trial stopped early:** no	
Participants	**Baseline characteristics** Placebo *Age (years) (mean SD)*: 54.6 ± 14.9 *Ethnicity*: not reported *Gender (males, females)*: 19/30 M (63%); 11/30 F (37%) *Length of surgery (minutes) (median, min‐max)*: 135 (60 to 210) *Proportion of participants on anticoagulants prior to surgery (n/N, %)*: 0/30, 0% *Incidence of preoperative anaemia (n/N, %)*: not reported *Co‐morbidities (n/N, %)*: not reported *ASA 1 (n/N, %)*: 17/30, 56.7% *ASA 2 (n/N, %)*: 12/30, 40% *ASA 3 (n/N, %)*: 1/30, 3.3% *ASA 4 (n/N, %)*: not reported *Number of participants randomised*: not reported *Number of participants receiving treatment*: 30 *Number of participants analysed*: 30 *Dropout rate*: 0/30, 0% TXA *Age (years) (mean SD)*: 53.5 ± 12.7 *Ethnicity*: not reported *Gender (males, females)*: 21/30 M (70%), 9/30 F (30%) *Length of surgery (minutes) (median, min‐max)*: 135 (60 to 225) *Proportion of participants on anticoagulants prior to surgery (n/N, %)*: 0/30, 0% *Incidence of preoperative anaemia (n/N, %)*: not reported *Co‐morbidities (n/N, %)*: not reported *ASA 1 (n/N, %)*: 10/30, 33.3% *ASA 2 (n/N, %)*: 18/30, 60% *ASA 3 (n/N, %)*: 2/30, 6.7% *ASA 4 (n/N, %)*: not reported *Number of participants randomised*: 30 *Number of participants receiving treatment*: 30 *Number of participants analysed*: 30 *Dropout rate*: 0/30, 0% Overall *Age (years) (mean SD)*: not reported *Ethnicity*: not reported *Gender (males, females)*: not reported *Length of surgery (minutes) (median, min‐max)*: not reported *Proportion of participants on anticoagulants prior to surgery (n/N, %)*: not reported *Incidence of preoperative anaemia (n/N, %)*: not reported *Co‐morbidities (n/N, %)*: not reported *ASA 1 (n/N, %)*: not reported *ASA 2 (n/N, %)*: not reported *ASA 3 (n/N, %)*: not reported *ASA 4 (n/N, %)*: not reported *Number of participants randomised*: not reported *Number of participants receiving treatment*: not reported *Number of participants analysed*: not reported *Dropout rate*: not reported **Inclusion criteria:** 1) ASA 1‐3, 2) age 18 to 75, 3) total hip arthroplasty surgery, 4) regional anaesthesia **Exclusion criteria:** 1) history of drug sensitivity, 2) liver and kidney failure, 3) DVT or embolism, 4) coagulopathy, 5) severe aortic or mitral valve stenosis, 6) neurologic or CVA disease, 7) aspirin or platelet antiaggregant in the week before surgery 8) or NSAIDs in the 2 days before surgery **If TKR, is tourniquet used:** not applicable **Indication for surgery:** primary OA (n = 51), femoral neck fracture (n = 4) and rheumatoid arthritis (n = 5) **Type of anaesthetic:** spinal‐epidural anaesthetic **Type of surgery:** primary THA	
Interventions	**Intervention characteristics** Placebo Group C received normal saline in place of TXA, in the same manner and same volume Placebo, IV, pre‐incision and intraop, infusion TXA The TXA group received a total dose of 50 mg/kg TXA mixed in normal saline, which started 15 minutes prior to the skin incision and took a total time of 30 minutes, meaning that it continued during part of the surgery. TXA, IV, 50 mg/kg, pre‐incision and intraop, infusion	
Outcomes	*Primary outcomes:* Amount of intraoperative bleeding Number of packed red blood cells given *Secondary outcomes:* Length of hospital stay Thromboembolic events	
Notes	**Sponsorship source:** non‐pharmaceutical **Country:** Turkey **Setting:** not reported **Comments:** none **Author's name:** SO Yasli **Institution:** Erciyes University **Email:** syasli@erciyes.edu.tr **Address:** Department of Oral and Maxillofacial Surgery, Faculty of Dentistry Erciyes, University Kayseri Turkey **Native language of paper:** English **Reference type:** full text (1), trial registration (1) **Trial registration number:** NCT02094066 **Was it translated for this review:** no	
***Risk of bias***		
**Bias**	**Authors' judgement**	**Support for judgement**
Random sequence generation (selection bias)	Low risk	Quote: "The patients were assigned as TA group and control (C) group using the coin toss method." Judgement comment: patients assigned to groups by coin toss method.
Allocation concealment (selection bias)	Unclear risk	Quote: not applicable Judgement comment: insufficient information to permit judgement.
Blinding of participants and personnel (performance bias) Subjective outcomes	Unclear risk	Quote: not applicable Judgement comment: insufficient information to permit judgement.
Blinding of participants and personnel (performance bias) Objective outcomes	Low risk	Quote: not applicable Judgement comment: objective outcome for personnel, so low risk of bias regardless of quality of blinding
Blinding of outcome assessment (detection bias) Subjective outcomes	Unclear risk	Quote: not applicable Judgement comment: insufficient information to permit judgement.
Blinding of outcome assessment (detection bias) Objective outcomes	Low risk	Quote: not applicable Judgement comment: objective outcome for personnel, so low risk of bias regardless of quality of blinding.
Incomplete outcome data (attrition bias) All outcomes	Unclear risk	Quote: not applicable Judgement comment: insufficient reporting of attrition/exclusions to permit judgement of ‘low risk’ or ‘high risk’.
Selective reporting (reporting bias)	Low risk	Quote: not applicable Judgement comment: all outcomes planned in the protocol or prospective trial registration are reported.
Other bias	Unclear risk	Quote: not applicable Judgement comment: inclusion and exclusion criteria in the protocol not the same as in the paper. No mention of numbers randomised or dropout rates for participants.

**Table:** Yen 2017

***Study characteristics***		
Methods	**Study design:** RCT **Intention‐to‐treat analysis:** yes **Duration of study:** 11 (8 months + 3 months follow‐up) **Power calculation reached:** yes **Transfusion strategy:** the trigger for allogenic transfusion of red blood cells was set at a Hb level of 8 g/dL in healthy patients, or between 8 and 9 g/dL in patients with clinical symptoms and signs of acute anaemia. In patients with cardiovascular disease, the transfusion threshold was set at a Hb level of 9 g/dL. **Was the trial stopped early:** no	
Participants	**Baseline characteristics** Placebo *Age (years) (mean SD)*: 70.87 (6.05) *Ethnicity*: not reported *Gender (males, females)*: 6/30 M (20%); 24/30 F (80%) *Length of surgery (minutes)*: not reported *Proportion of participants on anticoagulants prior to surgery (n/N, %)*: 0/30, (0%) *Incidence of preoperative anaemia (n/N, %)*: 0 *Co‐morbidities (n/N, %)*: not reported *ASA 1 (n/N, %)*: 1/30, (3.3%) *ASA 2 (n/N, %)*: 24/30, (80%) *ASA 3 (n/N, %)*: 4/30, (13.3%) *ASA 4 (n/N, %)*: not reported *Number of participants randomised*: 33 *Number of participants receiving treatment*: 30 *Number of participants analysed*: 30 *Dropout rate*: 3/33, (9.09%) TXA, IV *Age (years) (mean SD)*: 69.13 (7.94) *Ethnicity*: not reported *Gender (males, females)*: 4/31 M (13%); 27/31 F (87%) *Length of surgery (minutes)*: not reported *Proportion of participants on anticoagulants prior to surgery (n/N, %)*: 0/31, (0%) *Incidence of preoperative anaemia (n/N, %)*: 0 *Co‐morbidities (n/N, %)*: not reported *ASA 1 (n/N, %)*: 1/31, 3.2% *ASA 2 (n/N, %)*: 22/31, (71%) *ASA 3 (n/N, %)*: 8/31, (25.8%) *ASA 4 (n/N, %)*: not reported *Number of participants randomised*: 32 *Number of participants receiving treatment*: 31 *Number of participants analysed*: 31 *Dropout rate*: 1/32, (3.13%) TXA, IA *Age (years) (mean SD)*: 69.66 (5.53) *Ethnicity*: not reported *Gender (males, females)*: 13/32 M (41%); 19/32 F (59%) *Length of surgery (minutes)*: not reported *Proportion of participants on anticoagulants prior to surgery (n/N, %)*: 0/32, (0%) *Incidence of preoperative anaemia (n/N, %)*: 0 *Co‐morbidities (n/N, %)*: not reported *ASA 1 (n/N, %)*: 0/32, (0%) *ASA 2 (n/N, %)*: 19/32, (59.4%) *ASA 3 (n/N, %)*: 13/32, (40.6%) *ASA 4 (n/N, %)*: not reported *Number of participants randomised*: 33 *Number of participants receiving treatment*: 32 *Number of participants analysed*: 32 *Dropout rate*: 1/33, (3.03%) Overall *Age (years) (mean SD)*: (range available) *Ethnicity*: not reported *Gender (males, females)*: not reported *Length of surgery (minutes)*: not reported *Proportion of participants on anticoagulants prior to surgery (n/N, %)*: not reported *Incidence of preoperative anaemia (n/N, %)*: not reported *Co‐morbidities (n/N, %)*: not reported *ASA 1 (n/N, %)*: not reported *ASA 2 (n/N, %)*: not reported *ASA 3 (n/N, %)*: 1 patient missing *ASA 4 (n/N, %)*: not reported *Number of participants randomised*: 98 *Number of participants receiving treatment*: not reported *Number of participants analysed*: not reported *Dropout rate*: not reported **Inclusion criteria:** 1) aged 40 years or more 2) underwent unilateral primary minimally invasive TKA **Exclusion criteria:** 1) patients with a documented history of thromboembolic disease, cardiovascular disease (myocardiac infarction or angina), stroke, coagulopathy, 2) lifelong warfarin treatment for thromboembolic prophylaxis, 3) impaired hepatic or renal function (impaired hepatic function was defined as liver enzyme level, AST or ALT, which is more than twice normal range, history of liver cirrhosis, elevated total bilirubin level, or coagulopathy (INR < 1.3); and impaired renal function was defined as GFR < 55 mL/min/1.73 m^2^, which is relative contraindicated for chemical venous thromboembolism and venography), 4) patients with an allergy history to tranexamic acid, 5) concomitant use of protease inhibitors of human immunodeficiency virus, 6) fibrinolytic agent that contraindicated the use of rivaroxaban, 7) preoperative anaemia (a haemoglobin level of ≤ 10 g/dL) **If TKR, is tourniquet used:** yes **Indication for surgery:** not reported **Type of anaesthetic:** general anaesthetic **Type of surgery:** primary TKR	
Interventions	**Intervention characteristics** Placebo Patients in the placebo group received saline twice, 20 mL intravenously 10 minutes before skin closure and 160 mL intra‐articularly via the drain after capsule closure before deflation of the tourniquet. Placebo, IV, intraop (10 mins before closure) AND placebo, IA, intraop before tourniquet deflation TXA, IV Patients in the IV group received 1 g (20 mL) TXA intravenously 10 minutes before skin closure and 160 mL saline intra‐articularly via the drain after capsule closure. TXA, IV, 1 g, intraop (10 mins before closure) AND placebo, IA, intraop before tourniquet deflation TXA, IA Patients in the topical TXA group received 10 mL of saline intravenously 10 minutes before skin closure and 3 g (60 mL) TXA (Transamin 50 mg/mL) Placebo, IV, intraop (10 mins before closure) AND TXA, IA, 3 g, intraop before tourniquet deflation	
Outcomes	*Primary outcome:* Estimated total blood loss *Secondary outcomes:* Rate of perioperative blood transfusion Rate of deep vein thrombosis (DVT) Wound complications Visual analogue scale (VAS) on POD 1 Length of hospital stay Range of motion of the knee	
Notes	**Sponsorship source:** non‐pharmaceutical **Country:** Taiwan **Setting:** single‐centre **Comments:** none **Author's name:** J‐W Wang **Institution:** Chang Gung University **Email:** wangjw@adm.cgmh.org.tw **Address:** Department of Orthopaedic Surgery, Kaohsiung Chang Gung Memorial Hospital, Chang Gung University, College of Medicine, Taoyuan, Taiwan **Native language of paper:** English **Reference type:** abstract (1), full text (1), trial registration (1) **Trial registration number:** NCT02453802 **Was it translated for this review:** no	
***Risk of bias***		
**Bias**	**Authors' judgement**	**Support for judgement**
Random sequence generation (selection bias)	Low risk	Quote: "Patients were assigned randomly into 3 groups: a placebo group (n = 30), a topical TXA group (n = 32) and an IV TXA group undefined = 31) by an independent research assistant who was not involved in the study via a computer‐generated method." Judgement comment: computer‐generated randomisation.
Allocation concealment (selection bias)	Low risk	Quote: "The research assistant placed the study medications into sequentially numbered opaque sealed envelopes, which were kept in our Clinical Trial Pharmacy." Judgement comment: sequentially numbered, sealed, opaque envelopes.
Blinding of participants and personnel (performance bias) Subjective outcomes	Low risk	Quote: "On the day of operation, the research assistant sent the sequentially numbered envelopes taking from the research pharmacy to the operating room according to sequence of the surgery. The envelope was opened and the study medications were prepared by an anesthetist not involved in this study. The study medications were all identical." Judgement comment: blinding of key study personnel and participants ensured.
Blinding of participants and personnel (performance bias) Objective outcomes	Low risk	Quote: Not applicable Judgement comment: objective outcome for personnel, so low risk of bias regardless of quality of blinding.
Blinding of outcome assessment (detection bias) Subjective outcomes	Low risk	Quote: "The patients, surgeons, research assistant, and nurses in charge were all blind to the randomization until the complete data were all collected." Judgement comment: blinding of outcome assessors ensured.
Blinding of outcome assessment (detection bias) Objective outcomes	Low risk	Quote: not applicable Judgement comment: objective outcome for outcome assessor, so low risk of bias regardless of quality of blinding.
Incomplete outcome data (attrition bias) All outcomes	Low risk	Quote: not applicable Judgement comment: all outcome data reported.
Selective reporting (reporting bias)	Low risk	Quote: not applicable Judgement comment: all pre‐specified outcome measures reported.
Other bias	Low risk	Quote: not applicable Judgement comment: appears to be free of other bias.

**Table:** Yen 2021

***Study characteristics***		
Methods	**Study design:** RCT **Intention‐to‐treat analysis:** per protocol **Duration of study:** 11 months (January 2017 and December 2017) + 3 months follow‐up = 14 months **Power calculation reached:** yes **Transfusion strategy:** the trigger for allogenic transfusion of red blood cells was set at a Hb level of 8 g/dL in healthy patients and between 8 and 9 g/dL in patients with clinical symptoms and signs of anaemia. **Was the trial stopped early:** no	
Participants	**Baseline characteristics** TXA, IA *Age (years) (mean SD)*: 69.35 (7.11) *Ethnicity*: not reported *Gender (males, females)*: 6/34 M (17.6%); 28/34 F (82.4%) *Length of surgery (minutes)*: not reported *Proportion of participants on anticoagulants prior to surgery (n/N, %)*: 0/34, 0% *Incidence of preoperative anaemia (n/N, %)*: 0/34, 0% *Co‐morbidities (n/N, %)*: not reported *ASA 1 (n/N, %)*: 2/34 (5.9%) *ASA 2 (n/N, %)*: 23/34 (67.6%) *ASA 3 (n/N, %)*: 9/34 (26.5%) *ASA 4 (n/N, %)*: not reported *Number of participants randomised*: 35 *Number of participants receiving treatment*: not reported *Number of participants analysed*: 34 *Dropout rate*: 1/35, 2.9% Floseal, IA *Age (years) (mean SD)*: 69.71 (6.79) *Ethnicity*: not reported *Gender (males, females)*: 6/34 M (17.6%); 28/34 F (82.4%) *Length of surgery (minutes)*: not reported *Proportion of participants on anticoagulants prior to surgery (n/N, %)*: 0/34, 0% *Incidence of preoperative anaemia (n/N, %)*: 0/34, 0% *Co‐morbidities (n/N, %)*: not reported *ASA 1 (n/N, %)*: 1/34 (2.9%) *ASA 2 (n/N, %)*: 19/34 (55.9%) *ASA 3 (n/N, %)*: 14/34 (41.2%) *ASA 4 (n/N, %)*: not reported *Number of participants randomised*: 34 *Number of participants receiving treatment*: not reported *Number of participants analysed*: 34 *Dropout rate*: 0/34, 0% Placebo, IA *Age (years) (mean SD)*: 69.71 (5.94) *Ethnicity*: not reported *Gender (males, females)*: 3/35 M (8.6%); 32/35 F (91.4%) *Length of surgery (minutes)*: not reported *Proportion of participants on anticoagulants prior to surgery (n/N, %)*: 0/35, 0% *Incidence of preoperative anaemia (n/N, %)*: 0/35, 0% *Co‐morbidities (n/N, %)*: not reported *ASA 1 (n/N, %)*: 1/35 (2.9%) *ASA 2 (n/N, %)*: 23/35 (65.7%) *ASA 3 (n/N, %)*: 11/35 (31.4%) *ASA 4 (n/N, %)*: not reported *Number of participants randomised*: 35 *Number of participants receiving treatment*: not reported *Number of participants analysed*: 35 *Dropout rate*: 0/35, 0% Overall *Age (years) (mean SD)*: not reported *Ethnicity*: not reported *Gender (males, females)*: not reported *Length of surgery (minutes)*: not reported *Proportion of participants on anticoagulants prior to surgery (n/N, %)*: 0/103, 0% *Incidence of preoperative anaemia (n/N, %)*: not reported *Co‐morbidities (n/N, %)*: not reported *ASA 1 (n/N, %)*: not reported *ASA 2 (n/N, %)*: not reported *ASA 3 (n/N, %)*: not reported *ASA 4 (n/N, %)*: not reported *Number of participants randomised*: 104 *Number of participants receiving treatment*: not reported *Number of participants analysed*: 103 *Dropout rate*: 1/104, 0.96% **Inclusion criteria:** patients who were aged 50 years or older with a preoperative Hb level of ≥ 11 g/dL and a history of thromboembolic disease, cardiovascular disease (myocardial infarction or angina, stroke) or risk factors related to venous thromboembolism, such as old age (≥ 70 years), obesity (BMI ≥ 25), a history of cancer or varicose veins of the leg **Exclusion criteria:** patients who had preoperative anaemia (a Hb level of < 11 g/dL), a history of infection or intra‐articular fracture of the affected knee, or impaired hepatic or renal function. The impaired hepatic function was defined as liver enzyme levels of AST or ALT that were more than twice the normal range, or a history of liver cirrhosis, while the impaired renal function was defined as a glomerular filtration rate (GFR) < 30 mL/min/1:73 m^2^, which is contraindicated for chemical thromboprophylaxis. Patient use of lifelong anticoagulant therapy, allergy to tranexamic acid, Floseal, enoxaparin or rivaroxaban, or preoperative evaluation as being at high risk during surgery by a cardiologist or neurologist were also exclusion criteria in this study. **If TKR, is tourniquet used:** yes **Indication for surgery:** osteoarthritis **Type of anaesthetic:** general **Type of surgery:** unilateral primary TKR	
Interventions	**Intervention characteristics** TXA, IA Patients in the topical TXA group received 3 g (30 mL) intra‐articular TXA (Transamin 100 mg/mL; China Chemical and Pharmaceutical Co, Taiwan) in 100 mL of saline via the drain after capsule closure. TXA, IA, 3 g, intraop Floseal, IA Patients in the topical Floseal® group received intra‐articular 10 mL Floseal® (Baxter, Deerfield, IL, USA), which was applied to the exposed bone surfaces of the femoral and tibial condyles after cutting and soft tissue release, as well as to the bleeding points of the soft tissue and tibial pinholes after cementing of the implant and before insertion of the tibial polyethylene liner. Floseal, IA, 10 mL, intraop Placebo, IA Patients in the placebo group received a 130 mL saline injection intra‐articularly via the drain after capsule closure. Placebo, IA, 130 mL, intraop	
Outcomes	*Primary outcomes:* Calculated total blood loss Hb level *Secondary outcomes:* Rate of perioperative blood transfusion Rate of deep vein thrombosis (DVT) Pulmonary embolism (PE) Wound complications Length of hospital stay	
Notes	**Sponsorship source:** non‐pharmaceutical (supported by the Ministry of Science and Technology (NMRPG8F0191)) **Country:** Taiwan **Setting:** single‐centre **Comments:** none **Author's name:** S‐H Yen **Institution:** Kaohsiung Chang Gung Memorial Hospital and Chang Gung University **Email:** J‐W Wang: wangjw@adm.cgmh.org.tw **Address:** Department of Orthopaedic Surgery, Kaohsiung Chang Gung Memorial Hospital and Chang Gung University, College of Medicine, Taiwan **Native language of paper:** English **Reference type:** full text (1), trial registration (1) **Trial registration number:** NCT02865174 **Was it translated for this review:** no	
***Risk of bias***		
**Bias**	**Authors' judgement**	**Support for judgement**
Random sequence generation (selection bias)	Low risk	Quote: "Patients were randomly assigned into 3 groups by an independent research assistant who was not taking part in the study via a computer‐generated simple randomization method" Judgement comment: adequate method of sequence generation with computer–generated code.
Allocation concealment (selection bias)	Low risk	Quote: "The study medications were packed into sequentially numbered opaque sealed envelopes by the research assistant." Judgement comment: adequate method of allocation concealment.
Blinding of participants and personnel (performance bias) Subjective outcomes	High risk	Quote: "On the day of surgery, the sealed envelopes were sent to the operating theatre according to the planned sequence of operations by the research assistant. At the time of surgery, the envelope was opened by a circulating nurse, and the study medications were transferred to and prepared by a scrub nurse, neither of whom were involved in this study. The surgeon was responsible to the application of the topical experimental agents, and the blinding to surgeon was impossible." Judgement comment: none
Blinding of participants and personnel (performance bias) Objective outcomes	Low risk	Quote: not applicable Judgement comment: objective outcome for personnel, so low risk of bias regardless of quality of blinding.
Blinding of outcome assessment (detection bias) Subjective outcomes	Low risk	Quote: "the research assistant were blind to the randomization until all data had been collected." Judgement comment: none
Blinding of outcome assessment (detection bias) Objective outcomes	Low risk	Quote: not applicable Judgement comment: objective outcome for personnel, so low risk of bias regardless of quality of blinding.
Incomplete outcome data (attrition bias) All outcomes	Unclear risk	Quote: not applicable Judgement comment: insufficient reporting of attrition/exclusions to permit judgement of ‘low risk’ or ‘high risk’.
Selective reporting (reporting bias)	Low risk	Quote: not applicable Judgement comment: none
Other bias	Low risk	Quote: not applicable Judgement comment: no other concerns such as early stopping or imbalanced study arms.

**Table:** Zeng 2017

***Study characteristics***		
Methods	**Study design:** RCT **Intention‐to‐treat analysis:** yes **Duration of study:** 5 months and 3 weeks follow‐up **Power calculation reached:** not reported **Transfusion strategy:** a transfusion protocol based on the guidelines for perioperative transfusion by the Chinese Ministry of Health was utilised to standardise the application of blood transfusions. Blood transfusion was indicated when the haemoglobin concentration was < 7 g/dL; blood transfusion was indicated when the haemoglobin concentration was < 8 g/dL in a patient who tolerated anaemia poorly, and was indicated when the haemoglobin concentration was < 10 g/dL in a patient who developed any anaemia‐related organ dysfunction. **Was the trial stopped early:** no	
Participants	**Baseline characteristics** Placebo *Age (years) (mean SD)*: 56.1 (11.2) *Ethnicity*: not reported *Gender (males, females)*: 29/50 M (58%); 21/50 F (42%) *Length of surgery (minutes)*: not reported *Proportion of participants on anticoagulants prior to surgery (n/N, %)*: 0/50 (0%) *Incidence of preoperative anaemia (n/N, %)*: not reported *Co‐morbidities (n/N, %)*: not reported *ASA 1 (n/N, %)*: 0, 0% *ASA 2 (n/N, %)*: 35/50 (70%) *ASA 3 (n/N, %)*: 15/50 (30%) *ASA 4 (n/N, %)*: 0/0 (0%) *Number of participants randomised*: 50 *Number of participants receiving treatment*: 50 *Number of participants analysed*: 50 *Dropout rate*: 0/50, (0%) TXA *Age (years) (mean SD)*: 51.1 (14.9) *Ethnicity*: not reported *Gender (males, females)*: 31/50 M (62%); 19/50 F (38%) *Length of surgery (minutes)*: not reported *Proportion of participants on anticoagulants prior to surgery (n/N, %)*: 0/50 (0%) *Incidence of preoperative anaemia (n/N, %)*: not reported *Co‐morbidities (n/N, %)*: not reported *ASA 1 (n/N, %)*: 0/0 (0%) *ASA 2 (n/N, %)*: 38/50 (76%) *ASA 3 (n/N, %)*: 12/50 (24%) *ASA 4 (n/N, %)*: 0/0 (0%) *Number of participants randomised*: 50 *Number of participants receiving treatment*: 50 *Number of participants analysed*: 50 *Dropout rate*: 0/50, (0%) Overall *Age (years) (mean SD)*: not reported *Ethnicity*: not reported *Gender (males, females)*: not reported *Length of surgery (minutes)*: not reported *Proportion of participants on anticoagulants prior to surgery (n/N, %)*: not reported *Incidence of preoperative anaemia*: not reported *Co‐morbidities*: not reported *ASA 1 (n/N, %)*: not reported *ASA 2 (n/N, %)*: not reported *ASA 3 (n/N, %)*: not reported *ASA 4 (n/N, %)*: not reported *Number of participants randomised*: not reported *Number of participants receiving treatment*: not reported *Number of participants analysed*: not reported *Dropout rate*: not reported **Inclusion criteria:** 1) all adult patients (aged between 18 and 90 years), 2) undergoing primary unilateral THA, 3) end‐stage joint disease and the treating surgeon believed their pre‐morbid activity profile and general condition made them suitable for a THA **Exclusion criteria:** 1) allergy to TXA, 2) preoperative hepatic or renal dysfunction, 3) preoperative use of anticoagulant medication 7 days prior to surgery, 4) history of fibrinolytic disorder or blood dyscrasia, 5) history of cerebrovascular accident or myocardial infarction, 6) New York Heart Association class III or IV heart failure, 7) atrial fibrillation, 8) history of deep vein thrombosis or pulmonary embolus, 9) preoperative international normalised ratio (INR) > 1.4, activated partial thromboplastin time (aPTT) > 1.4 × normal **If TKR, is tourniquet used:** not applicable **Indication for surgery:** end‐stage joint disease (osteoarthritis, osteonecrosis of the femoral head, rheumatoid arthritis) **Type of anaesthetic:** general anaesthetic **Type of surgery:** primary THR	
Interventions	**Intervention characteristics** Placebo The TXA group received IV (15 mg/kg, 15 mg TXA) in 1.5 mL normal saline (NS) combined with topical administration (1000 mg TXA in 100 mL NS) of TXA during THA procedure, and the placebo group received the same dosage of NS (100 mL NS IV combined with 100 mL NS topical). For patients in the TXA group, 15 mg/kg TXA was applied IV 5 min before the skin incision. Placebo, 100 mL IV, intraop (5 mins before incision) AND placebo, 20 mL, IA, intraop to acetabular cup (bathed) + 20 mL to femoral canal + 60 mL to open joint surface TXA The TXA group received IV (15 mg/kg, 15 mg TXA) in 1.5 mL normal saline (NS) combined with topical administration (1000 mg TXA in 100 mL NS) of TXA during THA procedure, and the placebo group received the same dosage of NS (100 mL NS IV combined with 100 mL NS topical). For patients in the TXA group, 15 mg/kg TXA was applied IV 5 min before the skin incision. TXA, 15 mg/kg IV, intraop (5 mins before incision) AND TXA, 1 g, IA, intraop broken into 20 mL to acetabular cup (bathed) + 20 mL to femoral canal + 60 mL to open joint surface	
Outcomes	*Primary outcomes:* Total blood loss Haemoglobin Haematocrit Platelet concentration changes Amount of drainage Amount of intraoperative blood loss Frequency of transfusion Number of blood units transfused *Secondary outcomes:* Length of postoperative stay Range of hip motion Harris hip scores (HHS) Perioperative complications or events such as infection, DVT or PE	
Notes	**Sponsorship source:** non‐pharmaceutical **Country:** China **Setting:** single‐centre **Comments:** none **Author's name:** Y Zeng **Institution:** Sichuan University **Email:** zengyigd@126.com **Address:** Department of Orthopaedics, West China Hospital, Sichuan University, 37# Guoxue Road, Chengdu, China 610041 **Native language of paper:** English **Reference type:** full text (1) trial registration (1) **Trial registration number:** ChiCTR‐TRC‐14004474 **Was it translated for this review:** no	
***Risk of bias***		
**Bias**	**Authors' judgement**	**Support for judgement**
Random sequence generation (selection bias)	Low risk	Quote: "The patients were randomized into two groups by computer‐generated list number:" Judgement comment: computer‐generated randomisation.
Allocation concealment (selection bias)	Unclear risk	Quote: not applicable Judgement comment: insufficient information to permit judgement.
Blinding of participants and personnel (performance bias) Subjective outcomes	Unclear risk	Quote: not applicable Judgement comment: no description of blinding given.
Blinding of participants and personnel (performance bias) Objective outcomes	Low risk	Quote: not applicable Judgement comment: objective outcome for personnel, so low risk of bias regardless of quality of blinding.
Blinding of outcome assessment (detection bias) Subjective outcomes	Unclear risk	Quote: not applicable Judgement comment: no description given of outcome assessor blinding.
Blinding of outcome assessment (detection bias) Objective outcomes	Low risk	Quote: not applicable Judgement comment: objective outcome for outcome assessor, so low risk of bias regardless of quality of blinding.
Incomplete outcome data (attrition bias) All outcomes	Low risk	Quote: not applicable Judgement comment: all outcome data were accounted for.
Selective reporting (reporting bias)	Low risk	Quote: not applicable Judgement comment: all pre‐specified outcome measures reported in full text.
Other bias	Low risk	Quote: not applicable Judgement comment: appears to be free of other bias.

**Table:** Zeng 2018

***Study characteristics***		
Methods	**Study design:** RCT **Intention‐to‐treat analysis:** yes **Duration of study:** 11 months (8 months + 3 months follow‐up) **Power calculation reached:** not reported **Transfusion strategy:** the use of blood transfusions was standardised according to a protocol based on the guidelines for peri‐operative transfusion provided by the Chinese Ministry of Health. According to the protocol, a blood transfusion was indicated when the haemoglobin concentration was < 70 g/L or when a patient developed any anaemia‐related organ dysfunction, such as an alteration in mental status or palpitation. **Was the trial stopped early:** no	
Participants	**Baseline characteristics** Extension + TXA, IV + TXA, topical *Age (years) (mean SD)*: 69.68 (6.34) *Ethnicity*: not reported *Gender (males, females)*: 10/30 M (33%); 20/30 F (67%) *Length of surgery (minutes)*: not reported *Proportion of participants on anticoagulants prior to surgery (n/N, %)*: not reported *Incidence of preoperative anaemia (n/N, %)*: not reported *Co‐morbidities (n/N, %)*: not reported *ASA 1 (n/N, %)*: 1/30, (3.33%) *ASA 2 (n/N, %)*: 24/30, (80%) *ASA 3 (n/N, %)*: 5/30, (16.67%) *ASA 4 (n/N, %)*: not reported *Number of participants randomised*: 30 *Number of participants receiving treatment*: 30 *Number of participants analysed*: 30 *Dropout rate*: 0/30, (0%) Control + TXA, IV *Age (years) (mean SD)*: 72.15 (7.24) *Ethnicity*: not reported *Gender (males, females)*: 13/30 M (43%); 17/30 F (57%) *Length of surgery (minutes)*: not reported *Proportion of participants on anticoagulants prior to surgery (n/N, %)*: not reported *Incidence of preoperative anaemia (n/N, %)*: not reported *Co‐morbidities (n/N, %)*: not reported *ASA 1 (n/N%)*: 4/30, (13.33%) *ASA 2 (n/N, %)*: 17/30, (56.67%) *ASA 3 (n/N, %)*: 9/30, (30%) *ASA 4 (n/N, %)*: not reported *Number of participants randomised*: 30 *Number of participants receiving treatment*: 30 *Number of participants analysed*: 30 *Dropout rate*: 0/30, (0%) Overall *Age (years) (mean SD)*: not reported *Ethnicity*: not reported *Gender (males, females)*: not reported *Length of surgery (minutes)*: not reported *Proportion of participants on anticoagulants prior to surgery (n/N, %)*: not reported *Incidence of preoperative anaemia (n/N, %)*: not reported *Co‐morbidities (n/N, %)*: not reported *ASA 1 (n/N, %)*: not reported *ASA 2 (n/N, %)*: not reported *ASA 3 (n/N, %)*: not reported *ASA 4 (n/N, %)*: not reported *Number of participants randomised*: not reported *Number of participants receiving treatment*: not reported *Number of participants analysed*: not reported *Dropout rate*: not reported **Inclusion criteria:** 1) patients undergoing primary unilateral TKA **Exclusion criteria:** 1) allergic to TXA, 2) treatment with warfarin, heparin, or oestrogen before surgery, 3) a history of hypercoagulation, haemophilia, deep vein thrombosis (DVT), pulmonary embolism (PE), 4) previous surgery to the knee, 5) bleeding disorders, platelet of bone marrow disorders **If TKR, is tourniquet used:** yes **Indication for surgery:** not reported **Type of anaesthetic:** not reported **Type of surgery:** primary TKR	
Interventions	**Intervention characteristics** Extension + TXA, IV + TXA, topical The patients in the flexion group and extension group received 1 g (100 mL) of intravenous TXA 5 minutes before inflation of the tourniquet, and 1 g (100 mL) TXA was irrigated in the wound after implantation of the components. TXA, IV, 1 g, intraop (5 min before tourniquet) + TXA, IA, 1 g, intraop (after implantation) Control + TXA, IV For the control group, a single dose of 1 g (100 mL) TXA was applied intravenously before tourniquet inflation and knee was completely extended after surgery, combined with 100 mL normal saline applied topically without TXA. TXA, IV, 1 g, intraop (5 min before tourniquet) + placebo, IA, intraop (after implantation)	
Outcomes	*Primary outcomes:* Total blood loss Changes in haemoglobin and haematocrit Amount of drainage Transfusion frequency Number of blood units transfused *Secondary outcomes:* Knee flexion motion Rates of DVT and PE Length of the postoperative hospital stay Hospital and surgery costs	
Notes	**Sponsorship source:** non‐pharmaceutical **Country:** China **Setting:** single‐centre **Comments:** none **Author's name:** B Shen **Institution:** Sichuan University **Email:** shenbin_1971@163.com **Address:** Department of Orthopaedic Surgery, West China Hospital, West China Medical School, Sichuan University, 37# Guoxue Road, Chengdu 610041, People’s Republic of China **Native language of paper:** English **Reference type:** full text (1) **Trial registration number:** ChiCTR‐INR‐17010951 **Was it translated for this review:** no	
***Risk of bias***		
**Bias**	**Authors' judgement**	**Support for judgement**
Random sequence generation (selection bias)	Low risk	Quote: not applicable Judgement comment: in trial registration published, mentions computer‐generated randomisation method.
Allocation concealment (selection bias)	Unclear risk	Quote: "Randomized was blinded and performed with the use of sealed envelopes" Judgement comment: not clear whether these were opaque and sequentially numbered.
Blinding of participants and personnel (performance bias) Subjective outcomes	Unclear risk	Quote: "Randomized was blinded and performed with the use of sealed envelopes in a 1:1:1 ratio opened before surgery." Judgement comment: insufficient evidence to permit judgement.
Blinding of participants and personnel (performance bias) Objective outcomes	Low risk	Quote: not applicable Judgement comment: objective outcome for personnel, so low risk of bias regardless of quality of blinding.
Blinding of outcome assessment (detection bias) Subjective outcomes	Unclear risk	Quote: "Randomized was blinded and performed with the use of sealed envelopes in a 1:1:1 ratio opened before surgery." Judgement comment: insufficient evidence to permit judgement.
Blinding of outcome assessment (detection bias) Objective outcomes	Low risk	Quote: not applicable Judgement comment: objective outcome for outcome assessor, so low risk of bias regardless of quality of blinding.
Incomplete outcome data (attrition bias) All outcomes	Low risk	Quote: not applicable Judgement comment: author contacted to clarify the discrepancy between 30 patients per group or 50 (as reported in the paper). Author confirmed 30 patients per group. Therefore, there are no missing outcome data.
Selective reporting (reporting bias)	High risk	Quote: ''Our primary outcome measures were the total blood loss (calculated using the modified Gross formula [14, 15]) and changes in haemoglobin and haematocrit on the third post‐operative day; amount of drainage; transfusion frequency; and number of blood units transfused. Our secondary outcome measures were the knee flexion motion at the time of final follow‐up, the rates of DVT and PE, length of the post‐operative hospital stay, and the hospital and surgery costs.'' Judgement comment: protocol published prior to publication included 'hidden blood loss' and 'hip recovery function' as primary outcomes, which are not reported in the final paper.
Other bias	Low risk	Quote: not applicable Judgement comment: paper appears to be free of other bias.

**Table:** Zhang 2007

***Study characteristics***		
Methods	**Study design:** RCT **Intention‐to‐treat analysis:** unclear **Duration of study:** 24 months (12 months + 12 months follow‐up) **Power calculation reached:** not reported **Transfusion strategy:** blood transfusion was less than 10 g/dL **Was the trial stopped early:** no	
Participants	**Baseline characteristics** Placebo *Age (years) (mean range)*: not reported *Ethnicity*: not reported *Gender (males, females)*: not reported *Length of surgery (minutes)*: not reported *Proportion of participants on anticoagulants prior to surgery (n/N, %)*: not reported *Incidence of preoperative anaemia (n/N, %)*: not reported *Co‐morbidities (n/N, %)*: not reported *ASA 1 (n/N, %)*: not reported *ASA 2 (n/N, %)*: not reported *ASA 3 (n/N, %)*: not reported *ASA 4 (n/N, %)*: not reported *Number of participants randomised*: 51 *Number of participants receiving treatment*: 51 *Number of participants analysed*: 51 *Dropout rate*: 0/51 (0%) TXA *Age (years) (mean range)*: not reported *Ethnicity*: not reported *Gender (males, females)*: not reported *Length of surgery (minutes)*: not reported *Proportion of participants on anticoagulants prior to surgery (n/N, %)*: not reported *Incidence of preoperative anaemia (n/N, %)*: not reported *Co‐morbidities (n/N, %)*: not reported *ASA 1 (n/N, %)*: not reported *ASA 2 (n/N, %)*: not reported *ASA 3 (n/N, %)*: not reported *ASA 4 (n/N, %)*: not reported *Number of participants randomised*: 51 *Number of participants receiving treatment*: 51 *Number of participants analysed*: 51 *Dropout rate*: 0/51 (0%) Overall *Age (years) (mean range)*: 68 (59 to 77) *Ethnicity*: not reported *Gender (males, females)*: 43 M, 59 F *Length of surgery (minutes)*: not reported *Proportion of participants on anticoagulants prior to surgery (n/N, %)*: not reported *Incidence of preoperative anaemia (n/N, %)*: not reported *Co‐morbidities (n/N, %)*: not reported *ASA 1 (n/N, %)*: not reported *ASA 2 (n/N, %)*: not reported *ASA 3 (n/N, %)*: not reported *ASA 4 (n/N, %)*: not reported *Number of participants randomised*: 102 *Number of participants receiving treatment*: 102 *Number of participants analysed*: 102 *Dropout rate*: not reported **Inclusion criteria:** patients who underwent TKA **Exclusion criteria:** not reported **If TKR, is tourniquet used:** yes **Indication for surgery:** osteoarthritis, rheumatoid arthritis, traumatic arthritis **Type of anaesthetic:** not reported **Type of surgery:** primary TKR	
Interventions	**Intervention characteristics** Placebo In Group B, only 250 mL of normal saline was infused intravenously Placebo, IV, intraop (prior to tourniquet deflation) AND placebo, IV, 3 hours later TXA In Group A, 1 g of tranexamic acid dissolved in 250 mL of normal saline was intravenously infused before deflation of the tourniquet; another intravenous administration of the same drug of the same dosage was given 3 hours later. TXA, IV, 1 g, intraop (prior to tourniquet deflation) AND TXA, IV, 1 g, 3 hours later	
Outcomes	*Primary outcomes:* Blood loss Blood transfusion Venous thrombosis *Secondary outcome:* Not reported	
Notes	**Sponsorship source:** not reported **Country:** China **Setting:** not reported **Comments:** mean units calculated from mL in paper. Assumed 525 mL as 1 unit. **Author's name:** F Zhang **Institution:** Tianjin Hospital **Email:** zhang656696@yahoo.com.cn **Address:** Department of Joint Surgery, Tianjin Hospital, Tianjin Medical University,Tianjin300211, P.R. China **Native language of paper:** Chinese **Reference type:** full text (1) **Trial registration number:** not applicable, but when checked, trial registration number not found **Was it translated for this review:** yes	
***Risk of bias***		
**Bias**	**Authors' judgement**	**Support for judgement**
Random sequence generation (selection bias)	Unclear risk	Quote: not applicable Judgement comment: insufficient information to permit judgement.
Allocation concealment (selection bias)	Unclear risk	Quote: not applicable Judgement comment: insufficient information to permit judgement.
Blinding of participants and personnel (performance bias) Subjective outcomes	Unclear risk	Quote: not applicable Judgement comment: no description of participant or personnel blinding.
Blinding of participants and personnel (performance bias) Objective outcomes	Low risk	Quote: not applicable Judgement comment: objective outcome for personnel, so low risk of bias regardless of quality of blinding.
Blinding of outcome assessment (detection bias) Subjective outcomes	Unclear risk	Quote: not applicable Judgement comment: no description of outcome assessor blinding.
Blinding of outcome assessment (detection bias) Objective outcomes	Low risk	Quote: not applicable Judgement comment: objective outcome for assessor, so low risk of bias regardless of quality of blinding.
Incomplete outcome data (attrition bias) All outcomes	Low risk	Quote: not applicable Judgement comment: no missing data reported.
Selective reporting (reporting bias)	Unclear risk	Quote: not applicable Judgement comment: no protocol to check pre‐specified outcomes reported.
Other bias	Unclear risk	Quote: not applicable Judgement comment: insufficient information to permit judgement, for example no information regarding patient characteristics. Unclear funding source.

**Table:** Zhao 2018

***Study characteristics***		
Methods	**Study design:** RCT **Intention‐to‐treat analysis:** yes **Duration of study:** 10 **Power calculation reached:** not reported **Transfusion strategy:** perioperative blood transfusions were given based on guidelines of the Chinese Ministry of Health, which indicate transfusions when haemoglobin concentration is < 70 g/L or when symptoms of anaemia are present, such as altered mental state or palpitation (regardless of haemoglobin concentration). **Was the trial stopped early:** no	
Participants	**Baseline characteristics** Placebo *Age (years) (mean SD)*: 59.86 (10.68) *Ethnicity*: not reported *Gender (males, females)*: 25/40 M (63%); 15/40 F (37%) *Length of surgery (minutes) mean (SD)*: 66.3 (6.2) *Proportion of participants on anticoagulants prior to surgery (n/N, %)*: not reported *Incidence of preoperative anaemia (n/N, %)*: not reported *Co‐morbidities (n/N, %)*: not reported *ASA 1 (n/N, %)*: 19/40 (47.5%) *ASA 2 (n/N, %)*: 9/40 (22.5%) *ASA 3 (n/N, %)*: 2/40 (5%) *ASA 4 (n/N, %)*: 0/40 (0%) *Number of participants randomised*: 40 *Number of participants receiving treatment*: 40 *Number of participants analysed*: 40 *Dropout rate*: 0/40 (0%) TXA, IV *Age (years) (mean SD)*: 59.50 (11.42) *Ethnicity*: not reported *Gender (males, females)*: 23/40 M (58%); 17/40 F (42%) *Length of surgery (minutes) mean (SD)*: 62.1 (9.1) *Proportion of participants on anticoagulants prior to surgery (n/N, %)*: not reported *Incidence of preoperative anaemia (n/N, %)*: not reported *Co‐morbidities (n/N, %)*: not reported *ASA 1 (n/N, %)*: 18/40 (45%) *ASA 2 (n/N, %)*: 8/40 (20%) *ASA 3 (n/N, %)*: 4/40 (10%) *ASA 4 (n/N, %)*: 0/40 (0%) *Number of participants randomised*: 40 *Number of participants receiving treatment*: 40 *Number of participants analysed*: 40 *Dropout rate*: 0/40 (0%) TXA, oral *Age (years) (mean SD)*: 60.47 (10.35) *Ethnicity*: not reported *Gender (males, females)*: 22/40 M (55%); 18/40 F (45%) *Length of surgery (minutes) mean (SD)*: 64.9 (13.4) *Proportion of participants on anticoagulants prior to surgery (n/N, %)*: not reported *Incidence of preoperative anaemia (n/N, %)*: not reported *Co‐morbidities (n/N, %)*: not reported *ASA 1 (n/N, %)*: 16/40 (40%) *ASA 2 (n/N, %)*: 10/40 (25%) *ASA 3 (n/N, %)*: 4/40 (10%) *ASA 4 (n/N, %)*: 0/40 (0%) *Number of participants randomised*: 40 *Number of participants receiving treatment*: 40 *Number of participants analysed*: 40 *Dropout rate*: 0/40 (0%) Overall *Age (years) (mean SD)*: not reported *Ethnicity*: not reported *Gender (males, females)*: not reported *Length of surgery (minutes) mean (SD)*: not reported *Proportion of participants on anticoagulants prior to surgery (n/N, %)*: not reported *Incidence of preoperative anaemia (n/N, %)*: not reported *Co‐morbidities (n/N, %)*: not reported *ASA 1 (n/N, %)*: not reported *ASA 2 (n/N, %)*: not reported *ASA 3 (n/N, %)*: not reported *ASA 4 (n/N, %)*: not reported *Number of participants randomised*: 120 *Number of participants receiving treatment*: 120 *Number of participants analysed*: 120 *Dropout rate*: not reported **Inclusion criteria:** all patients had been diagnosed with hip osteoarthritis or femoral head necrosis (Ficat III or IV) **Exclusion criteria:** 1) body mass index (BMI) > 30 kg/m^2^, 2) Crowe type 3 or 4 dysplasia, 3) previous hardware, 4) prior hip surgery, 5) inability to tolerate general anaesthesia, 6) bilateral arthroplasty, 7) allergy to TXA, 8) history of renal failure, 9) kidney transplant, 10) recent arterial thromboembolic event such as myocardial infarction or stroke, hypercoagulation, haemophilia, deep vein thrombosis, or pulmonary embolism, 11) declined to participate or to receive blood products **If TKR, is tourniquet used:** not applicable **Indication for surgery:** hip osteoarthritis or femoral head necrosis (Ficat III or IV) **Type of anaesthetic:** general **Type of surgery:** primary THR	
Interventions	**Intervention characteristics** Placebo Patients in the control group received ascorbic acid tablets and intravenous injections of saline as in the other groups, but no TXA Placebo, IV + oral, 10 mins preop + 3 hours postop AND placebo, oral, 2 hours preop and 3 hours postop TXA, IV TXA in the intravenous group was administered at a dose of 15 mg/kg at 10 minutes before skin incision and again at 3 hours after THA. In order to support the double‐blind nature of the study, patients in this group also received 4 tablets of ascorbic acid (250 mg, so 1000 mg total) at 2 hours before and 3 hours after THA. TXA, 15 mg/kg, IV 10 mins preop, + 3 hours postop, AND placebo, oral 2 hours preop and 3 hours postop TXA, oral TXA in the oral group was administered at a dose of 20 mg/kg at 2 hours before and 3 hours after THA. In order to support the double‐blind nature of the study, patients in this group also received intravenous injections of saline 10 minutes before skin incision and again 3 hours after THA. TXA, 20 mg/kg, oral, 2 hours preop and 3 hours postop AND placebo, IV 10 mins preop + 3 hours postop	
Outcomes	*Primary outcomes:* Reduction in haemoglobin concentration (defined as pre‐operative haemoglobin minus lowest postoperative haemoglobin) Total blood loss Intraoperative blood loss Transfusion rates Number of blood units transfused *Secondary outcomes:* Thromboembolic events Wound complications	
Notes	**Sponsorship source:** none **Country:** China **Setting:** single‐centre **Comments:** none **Author's name:** P‐D Kang **Institution:** Sichuan University **Email:** kangpd@163.com **Address:** Department of Orthopedics, West China Hospital, West China School of Medicine, Sichuan University, 37# Wuhou Guoxue Road, Chengdu 610041, People’s Republic of China **Native language of paper:** English **Reference type:** full text (1) **Trial registration number:** ChiCTR‐INR17013110 **Was it translated for this review:** no	
***Risk of bias***		
**Bias**	**Authors' judgement**	**Support for judgement**
Random sequence generation (selection bias)	Low risk	Quote: "Patients recruited into the study were randomized into three groups using a computer‐generated randomization table at an allocation ratio of 1:1:1 with a maximum number of 40 per each group." Judgement comment: use of computer‐generated randomisation.
Allocation concealment (selection bias)	Unclear risk	Quote: not applicable Judgement comment: insufficient information to permit judgement.
Blinding of participants and personnel (performance bias) Subjective outcomes	Low risk	Quote: "All drugs were administered by a nurse and anesthetist who were not involved in the surgeries, care, or assessment of outcomes." Judgement comment: blinding of participants and key study personnel ensured and unlikely that the blinding could have been broken.
Blinding of participants and personnel (performance bias) Objective outcomes	Low risk	Quote: not applicable Judgement comment: objective outcome for personnel, so low risk of bias regardless of quality of blinding.
Blinding of outcome assessment (detection bias) Subjective outcomes	Low risk	Quote: "Patients and researchers who prospectively collected all clinical information were blinded to patient allocation until the final data analysis" Judgement comment: blinding of outcome assessors ensured and unlikely could have been broken.
Blinding of outcome assessment (detection bias) Objective outcomes	Low risk	Quote: "Patients and researchers who prospectively collected all clinical information were blinded to patient allocation until the final data analysis" Judgement comment: objective outcome for outcome assessor, so low risk of bias regardless of quality of blinding
Incomplete outcome data (attrition bias) All outcomes	Low risk	Quote: not applicable Judgement comment: all outcome data were reported.
Selective reporting (reporting bias)	High risk	Quote: not applicable Judgement comment: the study registration is available and not all pre‐specified outcomes have been reported (VAS score, ROM and swelling).
Other bias	Unclear risk	Quote: not applicable Judgement comment: insufficient information to permit judgement.

**Table:** Zohar 2004

***Study characteristics***		
Methods	**Study design:** RCT **Intention‐to‐treat analysis:** yes **Duration of study:** not reported **Power calculation reached:** yes **Transfusion strategy:** in all cases, a haematocrit of 28% constituted the postoperative transfusion trigger **Was the trial stopped early:** no	
Participants	**Baseline characteristics** TXA, IV long *Age (years) (mean SD)*: 73 (8) *Ethnicity*: not reported *Gender (males, females)*: 6/20 M (30%); 14/20 F (70%) *Length of surgery (minutes) mean (SD)*: 118 (19) *Proportion of participants on anticoagulants prior to surgery (n/N, %)*: 0/20 (0%) *Incidence of preoperative anaemia (n/N, %)*: not reported *Co‐morbidities (n/N, %)*: not reported *ASA 1 (n/N, %)*: 4/20 (20%) *ASA 2 (n/N, %)*: 16/20 (80%) *ASA 3 (n/N, %)*: 0/20 (0%) *ASA 4 (n/N, %)*: 0/20 (0%) *Number of participants randomised*: 20 *Number of participants receiving treatment*: 20 *Number of participants analysed*: 20 *Dropout rate*: 0/20 (0%) TXA, IV short *Age (years) (mean SD)*: 69 (7) *Ethnicity*: not reported *Gender (males, females)*: 4/20 M (20%); 16/20 F (80%) *Length of surgery (minutes) mean (SD)*: 124 (14) *Proportion of participants on anticoagulants prior to surgery (n/N, %)*: 0/20 (0%) *Incidence of preoperative anaemia (n/N, %)*: not reported *Co‐morbidities (n/N, %)*: not reported *ASA 1 (n/N, %)*: 2/20 (10%) *ASA 2 (n/N, %)*: 17/20 (85%) *ASA 3 (n/N, %)*: 1/20 (5%) *ASA 4 (n/N, %)*: 0/20 (0%) *Number of participants randomised*: 20 *Number of participants receiving treatment*: 20 *Number of participants analysed*: 20 *Dropout rate*: 0/20 (0%) TXA, oral *Age (years) (mean SD)*: 69 (10) *Ethnicity*: not reported *Gender (males, females)*: 8/20 M (40%); 12/20 F (60%) *Length of surgery (minutes) mean (SD)*: 122 (17) *Proportion of participants on anticoagulants prior to surgery (n/N, %)*: 0/20 (0%) *Incidence of preoperative anaemia (n/N, %)*: not reported *Co‐morbidities (n/N, %)*: not reported *ASA 1 (n/N, %)*: 5/20 (25%) *ASA 2 (n/N, %)*: 15/20 (75%) *ASA 3 (n/N, %)*: 0/20 (0%) *ASA 4 (n/N, %)*: 0/20 (0%) *Number of participants randomised*: 20 *Number of participants receiving treatment*: 20 *Number of participants analysed*: 20 *Dropout rate*: 0/20 (0%) Overall *Age (years) (mean SD)*: not reported *Ethnicity*: not reported *Gender (males, females)*: not reported *Length of surgery (minutes) mean (SD)*: not reported *Proportion of participants on anticoagulants prior to surgery (n/N, %)*: not reported *Incidence of preoperative anaemia (n/N, %)*: not reported *Co‐morbidities (n/N, %)*: not reported *ASA 1 (n/N, %)*: not reported *ASA 2 (n/N, %)*: not reported *ASA 3 (n/N, %)*: not reported *ASA 4 (n/N, %)*: not reported *Number of participants randomised*: not reported *Number of participants receiving treatment*: not reported *Number of participants analysed*: not reported *Dropout rate*: not reported **Inclusion criteria:** 1) ASA physical status I–III, 2) undergoing elective TKR **Exclusion criteria:** 1) patients with a history of severe ischaemic heart disease (New York Heart Association Class III and IV), 2) chronic renal failure 3) cirrhosis 4) bleeding disorders 5) current anticoagulant therapy **If TKR, is tourniquet used:** yes **Indication for surgery:** not reported **Type of anaesthetic:** general **Type of surgery:** primary TKR	
Interventions	TXA, IV long In group TXA‐long, 30 min before the limb tourniquet was deflated, an IV bolus dose of TXA (TEVA™; Biogal Pharmaceutical Works Ltd., Debrecen, Hungary) 15 mg/kg was administered over 30 min. Thereafter, a constant IV infusion of 10 mg/kg/h was administered until 12 h after final deflation of the limb tourniquet. TXA, IV, 15 mg/kg, intraop (30 mins prior to tourniquet deflation) infusion over 30 mins + 10 mg/kg, infusion for 12 h TXA, IV short In group TXA‐short, 30 min before deflation of the limb tourniquet, an IV bolus dose of TXA 15 mg/kg was administered over 30 min, followed by a constant IV infusion of 10 mg/kg/h until 2 h after final deflation of the limb tourniquet (time of discharge from the PACU). Thereafter, oral TXA 1 g was administered after 6 and 12 h. TXA, IV, 15 mg/kg, intraop (30 mins prior to tourniquet deflation) infusion over 30 mins + 10 mg/kg, infusion for 2 h AND TXA, oral, 1 g 6 + 12 hours after 2 h infusion TXA, oral In group TXA‐oral, 60 min before surgery, an oral dose of TXA 1 g was administered. After surgery, the same dose of TXA was administered every 6 h for the next 18 h. TXA, oral, 1 g, preop (1 h prior) + 1 g postop 6 and 18 h	
Outcomes	*Primary outcomes:* Allogeneic blood transfusion Blood accumulation in surgical drain Thromboembolic events	
Notes	**Sponsorship source:** not reported **Country:** Israel **Setting:** not reported **Comments:** mean and SD worked out from table in paper **Author's name:** E Zohar **Institution:** Tel Aviv University **Email:** Fredman.Brian@clalit.org.il **Address:** Department of Anesthesiology and Intensive Care, Meir Hospital, Kfar Saba 44281, The Sackler School of Medicine, Tel Aviv University, Tel Aviv, Israel **Native language of paper:** English **Reference type:** full text (1), abstract (1) **Trial registration number:** not applicable, but when checked, trial registration number not found **Was it translated for this review:** no	
***Risk of bias***		
**Bias**	**Authors' judgement**	**Support for judgement**
Random sequence generation (selection bias)	Low risk	Quote: "According to a computer‐generated randomization table" Judgement comment: computer‐generated randomisation.
Allocation concealment (selection bias)	Unclear risk	Quote: "patients were allocated to one of four treatment groups." Judgement comment: insufficient information to permit judgement.
Blinding of participants and personnel (performance bias) Subjective outcomes	High risk	Quote: not applicable Judgement comment: treatment regimens are different. Therefore, blinding of key personnel not ensured.
Blinding of participants and personnel (performance bias) Objective outcomes	Low risk	Quote: not applicable Judgement comment: objective outcome for personnel, so low risk of bias regardless of quality of blinding.
Blinding of outcome assessment (detection bias) Subjective outcomes	Unclear risk	Quote: "Allogeneic blood transfusion was verified by an independent observer (ME), who was blinded to the treatment modality." Judgement comment: insufficient information to permit judgement.
Blinding of outcome assessment (detection bias) Objective outcomes	Low risk	Quote: not applicable Judgement comment: objective outcome for outcome assessor, so low risk of bias regardless of quality of blinding.
Incomplete outcome data (attrition bias) All outcomes	Low risk	Quote: not applicable Judgement comment: no missing data.
Selective reporting (reporting bias)	High risk	Quote: "Three months after hospital discharge, all patients were interviewed by a blinded researcher. During this interview, the incidence of DVT, pulmonary embolus, myocardial infarction, transient ischemic attack, and stroke was recorded." Judgement comment: myocardial infarction, TIA and stroke rates not reported.
Other bias	Unclear risk	Quote: not applicable Judgement comment: no funding source declared.

ALT: alanine transaminase; ASA: American Society of Anesthesiologists; AST: aspartate transaminase; BMI: body mass index; BW: body weight; CNS: central nervous system; CRP: c‐reactive protein; CSS: carbazochrome sodium sulfonate; CVA: cerebrovascular accident; DAA: direct anterior approach; DVT: deep vein thrombosis; DXM: dexamethasone; EACA: epsilon aminocaproic acid; F: female; FDA: (US) Food and Drug Administration; FDP: fibrin(ogen) degradation products; FU: follow‐up; GFR: glomerular filtration rate; Hb: haemoglobin; Hct: haematocrit; hr/h: hour; HRT: hormone replacement therapy; IA: intra‐articular; IBL: intraoperative blood loss; IL‐6 interleukin 6; INR: international normalised ratio; intraop: intraoperative; IQR interquartile range; ITT: intention‐to‐treat; IV: intravenous; KIU: kallikrein inactivation units; M: male; MCPI: multimodal cocktail periarticular injection; MI: myocardial infarction; MINS: minutes; NRS: numerical rating scale; NS: normal saline; NSAID: non‐steroidal anti‐inflammatory drug; OA: osteoarthritis; OCP: oral contraceptive pill; PACU: post‐anaesthesia care unit; PCV: packed cell volume; PE: pulmonary embolism; PF1.2: prothrombin fragment 1.2; POD: postoperative day; postop: postoperative; PRBC: packed red blood cells; preop: preoperative; RA: rheumatoid arthritis; RBC: red blood cells; RCT: randomised controlled trial; ROM: range of motion; SAGM: standard additive solution for storage of red blood cells upon collection (S = sodium chloride, A = adenine, M = mannitol); SAH: subarachnoid haemorrhage; SD: standard deviation; SEM: standard error of the mean; TCI: transient cognitive impairment; THA: total hip arthroplasty; THR: total hip replacement; TIA: transient ischaemic attack; TKR: total knee replacement; TXA: tranexamic acid; VAS: visual analogue scale; VTE: venous thromboembolism; yr: year

### Characteristics of excluded studies [ordered by study ID]

**Table:**

Study	Reason for exclusion
Abdel 2018	Retrospectively registered trial
Abrisham 2018	Retrospectively registered trial
ACTRN12613001043729	Does not have ethical approval, withdrawn, unfunded
ACTRN12616000606482	Retrospectively registered trial
ACTRN12616000907448	Retrospectively registered trial
ACTRN12617000617369	Ineligible study design: not a prospective RCT. No details provided in the abstract as to the treatment group allocation. Trial registry information states that this is a "non‐randomised study".
Aguilera 2012	Ineligible study design: not an RCT. The full text was required to identify that patients were not randomised to the two study groups.
Aguilera 2013	Retrospectively registered trial
Aguilera 2015	Retrospectively registered trial
Ahmed 2018	Unregistered trial: author confirmed trial not registered
Akgul 2016	Inelgibile study design: not an RCT. The full text was required to identify that patients were not randomised to the 2 study groups.
Alshryda 2013	Retrospectively registered trial
Alshryda 2013a	Retrospectively registered trial
Amar 2003	Ineligible patient population: data provided for a mixed population only in the paper. Author contacted by email but was unable to provide data for just our population of interest.
Bali 2011	Ineligible study design: not an RCT. No abstract available and full text of paper required to determine eligibility on study design.
Benoni 1997	Ineligible study design: the study was designed as a laboratory study, which was not discovered until screening of the full text.
Bloomfield 2012	Retrospectively registered trial
Bouali 2011	Unregistered trial: author confirmed trial not registered
Cao 2015	Ineligible study design: the method of treatment allocation was "Non‐random grouping".
Cao 2018a	Retrospectively registered trial
Cao 2018b	Retrospectively registered trial
Capdevila 1998	Ineligible patient population (data provided for a mixed population. Author emailed to ask for access to individual patient populations, but we have had no response to our emails). Study excluded due to lack of available study data.
Chareancholvanich 2011a	Retrospectively registered trial
Chen 2014	Ineligible study design: not a prospective RCT, as randomisation was not done. Full text of the paper required in order to understand that this was not a randomised study.
Chen 2016c	Retrospectively registered trial
ChiCTR‐INR‐16008801	Retrospectively registered trial
ChiCTR‐INR‐16009770	Retrospectively registered trial
ChiCTR‐INR‐17010826	Retrospectively registered trial
ChiCTR‐IOR‐14005705	Retrospectively registered trial
ChiCTR‐IPR‐14005247	Retrospectively registered trial
ChiCTR‐IPR‐15005884	Retrospectively registered trial
ChiCTR‐IPR‐17011669	Retrospectively registered trial
ChiCTR‐IPR‐17011896	Retrospectively registered trial
ChiCTR‐TRC‐14004379	Ineligible patient population: population was patients with fractures. Full study details required to understand the population of interest in this study.
ChiCTR‐TRC‐14004440	Retrospectively registered trial
ChiCTR‐TRC‐14004671	Retrospectively registered trial
ChiCTR1800015839	Ineligible comparator: carbazochrome sodium sulfonate. Full text required to identify the comparator used in this study.
ChiCTR1800016494	Retrospectively registered trial
ChiCTR1800016640	Retrospectively registered trial
ChiCTR1800017563	Retrospectively registered trial
ChiCTR1800018049	Retrospectively registered trial
Choufani 2015	Ineligible comparator: standard care. Full text required to understand the nature of the comparator in this study.
Cornell 2017	Ineligible study design: not a prospective RCT, but a commentary. No abstract and title states "randomized controlled trial". Full text required to understand that this is not a report of an RCT but a commentary.
CTRI/2010/091/001260	Retrospectively registered trial
CTRI/2015/09/006185	Retrospectively registered trial
CTRI/2017/04/008401	Retrospectively registered trial
CTRI/2017/07/009076	Retrospectively registered trial
CTRI/2018/03/012728	Retrospectively registered trial
Cvachovec 2011	Ineligible comparator: standard care. The abstract does not mention the comparator: full text required to understand the nature of the comparator.
DeNapoli 2016	Unregistered trial: author confirmed trial not registered
DeSandesKimura 2016	Retrospectively registered trial
Dong 2017	Ineligible study design (not a prospective RCT, as randomisation was not done)
DRKS00007564	Study withdrawn
Drosos 2016	Unregistered trial: author confirmed trial not registered
EUCTR‐2009‐012043‐42‐UK	Study prematurely ended and no data available
EUCTR‐2015‐000107‐94	Retrospectively registered trial
Fernandez‐Collins 2017	Ineligible study design: the method of treatment allocation was described as "To ensure randomisation the hospital clinical file number was used, assigned in order of patient entry, with the result that even numbers were assigned to group A and odd numbers to group B", indicating that this was not a randomised controlled trial.
Fraval 2017	Retrospectively registered trial
Fraval 2019	Retrospectively registered trial
Freick 1983	Ineligible comparator: standard care. No abstract, full text required to understand the eligibility of the intervention and comparator in this study.
George 2018	Retrospectively registered trial
Gonzalez‐Osuna 2017	Retrospectively registered trial
Guerreiro 2017	Retrospectively registered trial
Haas 1984	Ineligible study design: not a prospective RCT, but pharmacokinetics. No abstract; full text required to determine that this was not an RCT.
Hegde 2013	Ineligible study design: not a prospective RCT, as randomisation was not done. Abstract available but limited in terms of description of study methods: full text required to determine study eligibility.
Heyse 2014	Retrospectively registered trial
Hill 2018	Retrospectively registered trial
Hou 2017	Retrospectively registered trial
Hourlier 2014	Unregistered trial: author confirmed trial not registered
Hourlier 2015	Unregistered trial: author confirmed trial not registered.
IRCT201009204780N1 Iranian Registry	Retrospectively registered trial
IRCT201012264784N2 Iranian Registry	Retrospectively registered trial
IRCT201509056280N8 Iranian Registry	Retrospectively registered trial
IRCT2016122730123N1 Iranian Registry	Retrospectively registered trial
IRCT20180411039272N1 Iranian Registry	Retrospectively registered trial
Iseki 2018	Ineligible study design: not an RCT. Full text required to confirm that this was not an RCT.
Ishida 2011	Ineligible study design: the method of treatment allocation was described as"Patients were alternately assigned to one of two groups. In the first, drain clamping was performed after injection of TXA (2,000 mg/20 ml) into the knee joint (TXA group). In the second, drain clamping was performed after injection of saline (20 ml) (control group)", indicating that this was not a randomised controlled trial.
ISRCTN43363116	Retrospectively registered trial
ISRCTN58790500	Retrospectively registered trial
ISRCTN59245192	Retrospectively registered trial
ISRCTN68578366	Retrospectively registered trial
Janatmakan 2021	Retrospectively registered trial
Joyce 2015	Retrospectively registered trial
Karampinas 2019	Unregistered trial: author confirmed trial not registered
KCT0003084	Retrospectively registered trial
Ketterl 1982	Ineligible study design: not a prospective RCT, but pharmacokinetics. No abstract; full text required to ascertain study type.
Kim 2012	Retrospectively registered trial
Kim 2018	Unregistered trial: author confirmed trial not registered
Kim 2021	Retrospectively registered trial
Kim 2021a	Retrospectively registered trial
Kim 2020	Retrospectively registered trial
Kluba 2012	Ineligible comparator group (standard care): no information given in the abstract on what the comparator treatment was. Full text required to understand that the comparator treatment was standard care.
Kraft 1999	Ineligible comparator: unclear what the comparator group received. Full text required to try and determine eligibility.
Kumar 2018	Retrospectively registered trial
Kyriakopoulos 2019	Unregistered trial: author confirmed trial was not prospectively registered
Lanoiselee 2018	Retrospectively registered trial
Laoruengthana 2019	Retrospectively registered trial
Laoruengthana 2019a	Unregistered trial: author confirmed trial was not prospectively registered
Lassen 2006	Ineligible patient population: data provided for a mixed population but not for a single population. Author emailed (on three separate occasions), but we have had no response to our emails.
Lee 2017b	Ineligible study design: not a prospective RCT, as randomisation was not done. No details of method of treatment group assignment in abstract; full text required to ascertain that this was not an RCT.
Leino 2010	Unregistered trial: author confirmed trial not registered
Li 2020	Unregistered trial: author confirmed trial not registered
Lin 2011	Ineligible study design: not a prospective RCT, as randomisation was not done. Abstract available but no details as to study group assignment. Full text revealed that this was not an RCT.
Lin 2012	Retrospectively registered trial
Lo 2020	Unregistered trial: author confirmed trial not registered
Lostak 2020	Unregistered trial: author confirmed trial not registered
Lostak 2020a	Unregistered trial: author confirmed trial not registered
Lum 2018	Retrospectively registered trial
Luo 2012	Ineligible study design: not a prospective RCT, as randomisation was not done. Translation of the full text was required to confirm this was not an RCT.
Luo 2018a	Retrospectively registered trial
Magill 2021	Retrospectively registered trial
Maniar 2017	Unregistered trial: author confirmed trial not registered
Martin 2014	Unregistered trial: author confirmed trial not registered
McConnell 2011	Retrospectively registered trial
McConnell 2012	Retrospectively registered trial
Mehta 2019a	Unregistered trial: author confirmed trial not registered
Melo 2017	Unregistered trial: author confirmed trial not registered
Mena 2002	Ineligible comparator: standard care. No abstract available; full text required to ascertain that the comparator was ineligible.
Mercuriali 2004	Ineligible study design: not a prospective RCT, as randomisation was not done. No abstract available; full text required to ascertain that this was not a randomised trial.
Meshram 2020	Retrospectively registered trial
Morales Santias 2020	Unregistered trial: author confirmed trial not registered
Morales‐Avalos 2021a	Retrospectively registered trial
Mortazavi 2020	Retrospectively registered trial
Motififard 2015	Retrospectively registered trial
Munoz Gomez 2013	Ineligible study design (not a prospective RCT, but an editorial). No abstract available; full text required to ascertain that this was not an randomised trial.
Najafi 2014	Retrospectively registered trial
NCT00161902	Retrospectively registered trial
NCT00167895	Retrospectively registered trial
NCT00375440	Study withdrawn
NCT00378872	Retrospectively registered trial
NCT00440921	Study withdrawn
NCT00668031	Retrospectively registered trial
NCT00985920	Retrospectively registered trial
NCT00990288	Retrospectively registered trial
NCT01027286	Retrospectively registered trial
NCT01285024	Retrospectively registered trial
NCT01410240	Ineligible comparator: standard care, but details of comparator not available at title and abstract screening; full text required to determine the comparator was ineligible.
NCT01449552	Retrospectively registered trial
NCT01472913	Retrospectively registered trial
NCT01656759	Retrospectively registered trial
NCT01816282	Retrospectively registered trial
NCT01850394	Retrospectively registered trial
NCT01866943	Retrospectively registered trial
NCT01881568	Retrospectively registered trial
NCT01891461	Retrospectively registered trial
NCT01937559	Retrospectively registered trial
NCT01940523	Retrospectively registered trial
NCT02152917	Retrospectively registered trial
NCT02252497	Retrospectively registered trial
NCT02312440	Retrospectively registered trial
NCT02323373	Retrospectively registered trial
NCT02327117	Retrospectively registered trial
NCT02374398	Retrospectively registered trial
NCT02393300	Retrospectively registered trial
NCT02427412	Retrospectively registered trial
NCT02458729	Retrospectively registered trial
NCT02504125	Retrospectively registered trial
NCT02553122	Study withdrawn
NCT02584725	Retrospectively registered trial
NCT02644473	Does not have ethical approval therefore not started; unfunded
NCT02650856	Retrospectively registered trial
NCT02687399	Retrospectively registered trial
NCT02829346	Retrospectively registered trial
NCT02860221	Retrospectively registered trial
NCT03019198	Retrospectively registered trial
NCT03044041	Retrospectively registered trial
NCT03074994	Retrospectively registered trial
NCT03183583	Retrospectively registered trial
NCT03328832	Retrospectively registered trial
NCT03359525	Retrospectively registered trial
NCT03365999	Retrospectively registered trial
NCT03690037	Retrospectively registered trial
Nielsen 2016	Retrospectively registered trial
NTR6464 Netherlands Trials Register	Ineligible comparator: drink supplement. No details on comparator in abstract; full text required to determine nature of the comparator.
Ollivier 2016	Ineligible study design: not a prospective RCT, but a commentary. No abstract available: full text required to ascertain that this was not an RCT.
Oremus 2014	Unregistered trial: author confirmed trial not registered
Pachauri 2014	Ineligible study design: not a prospective RCT, but quasi‐randomised. Abstract states that this was a "a randomized, prospective, observational, double‐blinded study". Full text was required to understand what study methodology had been used in this study.
Palija 2021	Unregistered study: author confirmed trial not registered
Pathan 2020	Retrospectively registered trial
Pavao 2019	Unregistered study: author confirmed trial not registered
Perez‐Jimeno 2018	Ineligible study design: the method of treatment allocation was "According to medical record number, consecutive THA patients fulfilling eligibility criteria were randomly assigned to receive topical TXA at the end of surgery (tranexamic group, even numbers) or not (control group, odd numbers)", indicating that this was not a randomised controlled trial.
Pinsornsak 2016	Retrospectively registered trial
Pinsornsak 2021	Retrospectively registered trial
Prakash 2016	Ineligible study design: not a prospective RCT, but a commentary. No abstract and full text required to determine that this was not an RCT.
Qin 2020	Retrospectively registered trial
Rajesparan 2009	Ineligible intervention: standard care. Not clear from the abstract whether there was a comparator arm and what the comparator arm was. Full text determined that the comparator arm was not eligible for this review.
Randelli 2010	Retrospectively registered trial
Randelli 2013	Retrospectively registered trial
Randelli 2014	Retrospectively registered trial
Roy 2012	Retrospectively registered trial
Ruiz‐Moyano 1997	Ineligible comparator: standard care. Conference abstract had to be translated from Spanish to determine eligibility. Following translation, it was determined that the comparator was standard care.
Sa‐Ngasoongsong 2011	Unregistered trial: author confirmed trial not registered
Sa‐Ngasoongsong 2013	Retrospectively registered trial
Sahin 2019	Unregistered trial: author confirmed trial not registered
Samama 2002	Ineligible patient population (data provided for a mixed population). Author contacted by email (on three separate occasions) to ask whether they would provide us with the data per patient group. Author did not respond to our emails.
Seol 2016	Ineligible study design: not a prospective RCT, but a prospective comparative study. Lack of clarity in study type in the methods meant that full text had to be examined to determine eligibility.
Seviciu 2016	Retrospectively registered trial
Shah 2006	Ineligible study design: not a prospective RCT, as randomisation was not done. No abstract available at title and abstract screening. Full text screening revealed that this was not an RCT.
Shihab 2021	Unregistered trial: author confirmed trial not registered
Skovgaard 2013	Retrospectively registered trial
SouzaNeto 2020	Retrospectively registered trial
Stutz 2004	Ineligible study design: not a prospective RCT, as randomisation was not done. Abstract implies patients were randomised to treatment groups, but on reading the full text it is clear that no method of randomisation was used to assign patients to treatment groups.
Suarez 2014	Ineligible comparator: standard care. No information on comparator was provided in the abstract. Full text revealed that the comparator was standard care.
Subramanyam 2018	Retrospectively registered trial
Tammachote 2019	Retrospectively registered trial
Tandogan 2021	Unregistered trial: author confirmed trial not registered
Tavares Sanchez‐Monge 2018	Unregistered trial: author confirmed trial not registered
TCTR20170503003 Thai Clinical Trials Registry	Retrospectively registered trial
TCTR20170618001 Thai Clinical Trials Registry	Retrospectively registered trial
TCTR20200511002	Retrospectively registered trial
Thipparampall 2017	Ineligible patient population: patients undergoing hip fracture surgery. The abstract states "patients scheduled for hip surgery". At full text, the nature of the surgery was stated: 'hip fracture', which is an ineligible patient population for this review.
Thorpe 1994	Ineligible comparator: standard care. No information was provided in the abstract on the comparator treatment. At full text, it was stated that the comparator group received 'standard care'.
Torkaman 2020	Retrospectively registered trial
Tripathy 2020	Unregistered trial: author confirmed trial not registered
Tzatzairis 2016	Retrospectively registered trial
U1111‐1172‐0797 Brazilian Clinical Trials Registry	Retrospectively registered trial
U1111‐1187‐8385 Brazilian Clinical Trials Registry	Retrospectively registered trial
U1111‐1207‐0408 Brazilian Clinical Trials Registry	Retrospectively registered trial
Van Elst 2013	Retrospectively registered trial
Vela 2012	Unregistered trial: author confirmed trial not registered
Wang 2001	Ineligible comparator: standard care. It was unclear from the abstract alone, what the comparator treatment arm was. Full text revealed that patients in the comparator treatment group received standard care.
Wang 2003	Ineligible comparator: standard care. It was unclear from the abstract alone, what the comparator treatment arm was. Full text revealed that patients in the comparator treatment group received standard care.
Wang 2017a	Retrospectively registered trial
Wang 2018a	Retrospectively registered trial
Wang 2018b	Retrospectively registered trial
Wang 2018c	Retrospectively registered trial
Wei 2018	Retrospectively registered trial
Wollinsky 1991	Ineligible comparator: standard care. No abstract available at title and abstract screening: full text revealed that the comparator was ineligible for inclusion in the review.
Wong 2010	Retrospectively registered trial
Xu 2015	Ineligible patient population: data provided for a mixed population (femoral neck fractures). Author emailed on three separate occasions to ask whether they could provide us with the data per individual patient group. Author did not respond to our emails.
Yi 2016	Retrospectively registered trial
Yuan 2017	Retrospectively registered trial
Yuan 2018	Retrospectively registered trial
Zecker 2016	Retrospectively registered trial
Zecker 2017	Retrospectively registered trial
Zeng 2018a	Retrospectively registered trial
Zhang 2020c	Retrospectively registered trial
Zhang 2021	Retrospectively registered trial
Zhang 2021a	Retrospectively registered trial
Zhou 2018	Retrospectively registered trial
Zufferey 2017	Retrospectively registered trial

RCT: randomised controlled trial TXA: tranexamic acid

### Characteristics of studies awaiting classification [ordered by study ID]

**Table:** Abdallah 2020

Methods	Randomised controlled trial
Participants	Patients at moderate‐to‐high risk of bleeding undergoing primary total knee arthroplasty
Interventions	**Intervention** TXA IA alone **Comparator** TXA IV vs combined group (IA + IV)
Outcomes	Drainage blood volume Total blood loss Hidden blood loss Intraoperative blood loss Allogenic transfusion rate Postoperative haemoglobin drop Amount of transfused blood units Thromboembolism Wound complications
Notes	We are unable to find a trial registration number for this study to ascertain whether the trial was prospectively registered.

**Table:** Adravanti 2018

Methods	Randomised controlled trial
Participants	Patients with primary knee osteoarthritis, post‐traumatic knee osteoarthritis or knee osteoarthritis secondary to rheumatoid arthritis undergoing total knee arthroplasty
Interventions	**Intervention** TXA IV alone **Comparator** Combined IV and topical IA TXA
Outcomes	Hb value Amount of drained blood (mL) in the first 24 hours after surgery Number of blood transfusion units Total postoperative blood loss Deep vein thrombosis (DVT) events Other postoperative complications
Notes	We are unable to find a trial registration number for this study to ascertain whether the trial was prospectively registered.

**Table:** Aggarwal 2016

Methods	Randomised controlled trial
Participants	Patients with knee arthritis undergoing simultaneous bilateral total knee arthroplasty
Interventions	**Intervention** TXA IV **Comparator** Topical TXA
Outcomes	Total blood loss Total drain output Number of blood units transfused Clinical and functional outcomes Visual analogue score Wound score
Notes	We are unable to find a trial registration number for this study to ascertain whether the trial was prospectively registered.

**Table:** Alipour 2013

Methods	Randomised controlled trial
Participants	Patients undergoing total knee arthroplasty
Interventions	**Intervention** Oral TXA **Comparator** Control
Outcomes	Decreasing blood loss Haematocrit Postoperative bleeding Bleeding rate Thrombotic complications
Notes	We are unable to find a trial registration number for this study to ascertain whether the trial was prospectively registered.

**Table:** Almeida 2018

Methods	Randomised controlled trial
Participants	Patients with primary knee osteoarthrosis undergoing total knee arthroplasty
Interventions	**Intervention** TXA **Comparator** Placebo
Outcomes	Haemoglobin Haematocrit Estimated blood loss Drain output
Notes	We are unable to find a trial registration number for this study to ascertain whether the trial was prospectively registered.

**Table:** Amin 2020

Methods	Randomised controlled trial
Participants	Patients with degenerative conditions like osteoarthritis and rheumatoid arthritis undergoing primary unilateral total knee arthroplasty
Interventions	**Intervention** TXA IA **Comparator** TXA IV
Outcomes	Mean postoperative haemoglobin Mean postoperative haematocrit Mean fall in haemoglobin Haematocrit
Notes	We are unable to find a trial registration number for this study to ascertain whether the trial was prospectively registered.

**Table:** Anon 2016

Methods	Randomised controlled trial
Participants	Patients undergoing primary unilateral total hip arthroplasty
Interventions	**Intervention 1** Topical group TXA **Intervention 2** TXA IV **Comparator** Combined group TXA (intravenous and intra‐articular)
Outcomes	Total blood loss Transfusion rate Maximum haemoglobin decrease Maximum haematocrit decrease Incidence of deep vein thrombosis
Notes	We are unable to find a trial registration number for this study to ascertain whether the trial was prospectively registered.

**Table:** Antinolfi 2014

Methods	Randomised controlled trial
Participants	Patients with primary knee osteoarthritis undergoing primary unilateral total knee arthroplasty
Interventions	**Intervention 1** Knee flexion **Intervention 2** Local administration of tranexamic acid **Comparator** Control
Outcomes	Blood loss volume Haemoglobin and haematocrit concentrations Blood transfusion needs
Notes	We are unable to find a trial registration number for this study to ascertain whether the trial was prospectively registered.

**Table:** Arora 2018

Methods	Randomised controlled trial
Participants	Patients with clinical and radiological features of end‐stage osteoarthritis of the knees undergoing simultaneous bilateral total knee replacement
Interventions	**Intervention** TXA IV **Comparator** TXA IA
Outcomes	Mean drop in postoperative haemoglobin Need for blood transfusion
Notes	We are unable to find a trial registration number for this study to ascertain whether the trial was prospectively registered.

**Table:** Arslan 2018

Methods	Randomised controlled trial
Participants	Patients undergoing total knee replacement surgery
Interventions	**Intervention 1** TXA + TNR (tourniquet not released) where there is no bleeding control and TXA is applied after wound closure without tourniquet release **Intervention 2** TXA ‐ TNR where placebo is applied after wound closure without tourniquet release **Intervention 3** TXA + TR (tourniquet released) where tourniquet is released first and TXA is applied after bleeding control and wound closure **Comparator** TXA ‐ TR where tourniquet release is followed by bleeding control and placebo application
Outcomes	Amount of haemorrhage Haemoglobin and haematocrit
Notes	We are unable to find a trial registration number for this study to ascertain whether the trial was prospectively registered.

**Table:** Bae 2014

Methods	Randomised controlled trial
Participants	Patients with degenerative osteoarthritis who underwent unilateral total knee arthroplasty
Interventions	**Intervention** Thrombin‐based haemostatic agent **Comparator** Control
Outcomes	Drain output Haemoglobin level Total red blood cell loss for 24 hours after surgery Transfusion rates Complications
Notes	We are unable to find a trial registration number for this study to ascertain whether the trial was prospectively registered.

**Table:** Balasubramanian 2016

Methods	Randomised controlled trial
Participants	Patients with bilateral osteoarthritis knee who underwent staged bilateral total knee arthroplasty
Interventions	**Intervention 1** TXA IV **Intervention 2** TXA IA **Comparator** Control
Outcomes	Postoperative blood loss Change in haemoglobin (Hb) level and haematocrit (PCV) Need for blood transfusion
Notes	We are unable to find a trial registration number for this study to ascertain whether the trial was prospectively registered.

**Table:** Bao 2019

Methods	Randomised controlled trial
Participants	Patients with unilateral knee osteoarthritis who underwent total knee arthroplasty
Interventions	**Intervention** IA infusion group **Comparator** IA infusion combined with peri‐articular injection group
Outcomes	24‐hour drainage Blood loss Coagulation function Visual analogue scale score Knee joint mobility Blood transfusion rate Incision infection rate Pulmonary embolism Deep vein thrombosis
Notes	We are unable to find a trial registration number for this study to ascertain whether the trial was prospectively registered.

**Table:** Bidolegui 2014

Methods	Randomised controlled trial
Participants	Patients with a diagnosis of osteoarthritis scheduled to have primary, unilateral total knee arthroplasty
Interventions	**Intervention** TXA IV **Comparator** Placebo
Outcomes	Transfusion rate Drain output Haemoglobin/haematocrit levels
Notes	Unable to find a trial registration and therefore cannot determine if the trial was prospectively registered.

**Table:** Borisov 2011

Methods	Randomised controlled trial
Participants	Patients undergoing primary cementless total hip arthroplasty
Interventions	**Intervention** Repeated IV bolus of TXA **Comparator** Single bolus of TXA
Outcomes	Postoperative drain blood loss within first 18 hrs Perioperative blood loss within first 48 hrs
Notes	Unable to find a trial registration and therefore cannot determine if the trial was prospectively registered.

**Table:** Bowman 2018

Methods	Randomised controlled trial
Participants	Patients undergoing elective primary total hip arthroplasty for osteoarthritis
Interventions	**Intervention** Thrombin‐collagen platelet‐rich plasma use **Comparator** Standard treatment
Outcomes	Estimated total blood loss Blood transfusions Operative blood loss Drain output Daily postoperative haematocrit Change in haematocrit Length of stay
Notes	Unable to find a trial registration and therefore cannot determine if the trial was prospectively registered.

**Table:** Bradshaw 2012

Methods	Randomised controlled trial
Participants	Patients undergoing unilateral total knee replacement arthroplasty for osteoarthritis
Interventions	**Intervention** Oral TXA **Comparator** Placebo
Outcomes	Haemoglobin Haematocrit Total blood loss Transfusion events Thromboembolic complications
Notes	Unable to find a trial registration and therefore cannot determine if the trial was prospectively registered.

**Table:** Canata 2012

Methods	Randomised controlled trial
Participants	Patients undergoing total knee replacements
Interventions	**Intervention 1** No infiltration was performed **Intervention 2** Infiltration of tranexamic acid **Comparator** Mixture of ropivacaine, clonidine, ketorolac and norepinephrine was infiltrated
Outcomes	Preoperative haemoglobin value Blood loss
Notes	Unable to find a trial registration and therefore cannot determine if the trial was prospectively registered.

**Table:** Cankaya 2017

Methods	Randomised controlled trial
Participants	Patients with knee osteoarthrosis who underwent primary total knee replacement
Interventions	**Intervention** Topical TXA group **Comparator** Combined (oral + topical) TXA group
Outcomes	Haemoglobin Haematocrit Drainage amounts Blood loss volume Transfusion rates
Notes	Unable to find a trial registration and therefore cannot determine if the trial was prospectively registered.

**Table:** Carvalho 2015

Methods	Randomised controlled trial
Participants	Patients who were scheduled for a primary total knee arthroplasty
Interventions	**Intervention 1** Topical TXA 1.5 g **Intervention 2** Topical TXA 3 g **Comparator** Placebo
Outcomes	Haematimetrics indices (haemoglobin, haematocrit, prothrombin time, activated partial thromboplastin time and international normalised ratio) Drain volume (mL) Allogenic blood transfusion Thromboembolic events Total calculated blood loss Acute postoperative infection
Notes	Unable to find a trial registration and therefore cannot determine if the trial was prospectively registered.

**Table:** Castro‐Menendez 2016

Methods	Randomised controlled trial
Participants	Patients with primary unilateral osteoarthritis of the knee and/or hip undergoing total hip and knee replacement
Interventions	**Intervention 1** 1 g of TXA intraoperative, followed by another postoperative **Intervention 2** 2 g preoperative **Comparator** Control
Outcomes	Postoperative blood loss Transfusion rate Thromboembolic complications
Notes	Unable to find a trial registration and therefore cannot determine if the trial was prospectively registered.

**Table:** Cavusoglu 2015

Methods	Randomised controlled trial
Participants	Patients undergoing primary total knee arthroplasty due to a diagnosis of primary osteoarthritis
Interventions	**Intervention 1** TXA IV **Intervention 2** TXA IA **Comparator** Control
Outcomes	Pre‐ and postoperative haemoglobin difference Volume of blood collected in drains Transfusion rate
Notes	Unable to find a trial registration and therefore cannot determine if the trial was prospectively registered.

**Table:** Chai 2015

Methods	Randomised controlled trial
Participants	Patients with knee osteoarthritis and rheumatoid arthritis who underwent unilateral total knee arthroplasty or bilateral unilateral replacement for the first time
Interventions	**Intervention** TXA IV **Comparator** Topical TXA
Outcomes	Drainage amount after replacement Haemoglobin and haematocrit on the next day after replacement Number of blood transfusion
Notes	Unable to find a trial registration and therefore cannot determine if the trial was prospectively registered.

**Table:** Chen 2016a

Methods	Randomised controlled trial
Participants	Patients who were diagnosed with osteoarthritis of the knee and scheduled for an elective primary total knee arthroplasty
Interventions	**Intervention** TXA IV **Comparator** TXA IA
Outcomes	Transfusion incidences Drain output Postoperative drop in serum haemoglobin level Perioperative blood loss Postoperative increment in lower limb girth measurements Duration of surgery Length of hospital stay Wound complications Thromboembolic events within 30 days of surgery
Notes	Unable to find a trial registration and therefore cannot determine if the trial was prospectively registered.

**Table:** Chen 2018

Methods	Randomised controlled trial
Participants	Patients with unilateral osteoarthritis undergoing primary cemented total knee arthroplasty
Interventions	**Intervention 1** TXA IV **Intervention 2** TXA IA **Comparator** Control
Outcomes	Hidden blood loss Blood transfusion rate Pulmonary embolism as well as lower extremity deep venous thrombosis
Notes	Unable to find a trial registration and therefore cannot determine if the trial was prospectively registered.

**Table:** ChiCTR‐INR‐16010188

Methods	Randomised controlled trial
Participants	Patients following primary total hip/knee arthroplasty due to osteoarthritis, hip dysplasia and ischaemic necrosis of the femoral head. Patients without preoperative platelet and coagulation function dysfunction, preoperative double lower limb venous colour Doppler ultrasound was not abnormal; voluntarily participated in clinical trials and signed informed consent, good compliance of patients.
Interventions	**Intervention** Intravenous remedial application of TXA after using TXA 3 times intravenously **Comparator** No more application of TXA after using TXA 3 times intravenously
Outcomes	Hidden blood loss Inflammatory index D‐dimer Fibrin degradation products Drop of haemoglobin Allogeneic blood transfusion rate Total blood loss VAS Limb swelling ratio Joint mobility Postoperative hospital stay Adverse event rate
Notes	Status on trial registry "not yet recruiting". RC emailed author for trial update on 3 separate occasions and has had no response. Trial status remains "not yet recruiting" as of 29 November 2022.

**Table:** ChiCTR‐INR‐16010270

Methods	Randomised controlled trial
Participants	Patients receiving total hip or total knee arthroplasty due to end‐stage hip and knee joint disease
Interventions	**Intervention** TXA IV **Comparator** Periarticular TXA
Outcomes	Blood loss Inflammatory index Drainage volume Complications Postoperative days required to straight leg elevation Thigh circumference Postoperative days required to stand Safety indexes
Notes	"Paper in submission". Although as of 29 November 2022, status of the trial is listed as "recruiting" on the trial registry, there has been no update to this page since 2016. RC emailed author for an update 13 July 2021 and author responded 5 August 2021, saying the paper is in submission. We have searched for a publication but have not found one so far and, from what data are available on the trial registry, we believe that this trial registration does not relate to any current included trial.

**Table:** ChiCTR‐INR‐17010951

Methods	Randomised controlled trial
Participants	Patients undergoing total knee arthroplasty for the treatment of osteoarthritis
Interventions	**Intervention 1** Knee flexion position after operation + TXA **Intervention 2** TXA **Comparator** Control
Outcomes	Total blood loss Hidden blood loss Joint function recovery Joint range of motion Blood transfusion rate Drainage volume Length of hospital stay
Notes	Status on trial registry "not yet recruiting". Trial registry page last updated March 2017. RC emailed author for update on the current status of the trial on 14 July 2021 (and on 3 occasions thereafter) but no response. Trial status remains "not yet recruiting" as of 29 November 2022.

**Table:** ChiCTR‐IOR‐15007198

Methods	Randomised controlled trial
Participants	Patients more than 18 years old, given preoperative blood routine test and blood coagulation at normal status, with a diagnosis of osteoarthritis, undergoing elective, unilateral, primary total knee arthroplasty
Interventions	**Intervention 1** IV + topical TXA **Intervention 2** TXA IV **Comparator** Topical TXA
Outcomes	Blood loss Volume and rate of postoperative autologous blood retransfusion Incidence of postoperative venous thromboembolism Degree of swelling on operated knee Knee Society Score Pain score (VAS) Operative time Length of hospital stay Patient satisfaction
Notes	Status on trial registry "not yet recruiting". Trial registry page last updated October 2015. RC emailed author for update on the current status of the trial on 13 July 2021 [and on 3 occasions thereafter) but no response. Trial status remains "not yet recruiting" as of 29 November 2022.

**Table:** ChiCTR‐IPR‐17011848

Methods	Randomised controlled trial
Participants	1) Patients undergoing initial total knee arthroplasty due to knee osteoarthritis; 2) platelet and blood coagulation functions were normal before surgery; 3) there was no abnormality in venous color Doppler ultrasound of both lower extremities before surgery; (4) voluntary participation in clinical trials and signed an informed consent form, patients with good compliance.
Interventions	**Intervention** 2 g po TXA before surgery, 2 g po TXA 3, 9 hours later **Comparator** 2 g po TXA before surgery, 2 g po TXA 3, 9 hours later, 2 g po with a 24‐hr interval to discharge, 1 g po TXA to POD14
Outcomes	Haemoglobin level Blood loss KSS Transfusion ESR CRP IL‐6 Range of motion Deep vein thrombosis Cost Swelling Nausea and vomiting Ecchymosis Length of hospital stay Pain
Notes	Status on trial registry "not yet recruiting". RC emailed author for update on the current status of the trial on 23 February 2022 (and on 3 occasions thereafter) but no response. Trial status remains "not yet recruiting" as of 29 November 2022.

**Table:** ChiCTR‐IPR‐17012265

Methods	Randomised controlled trial
Participants	1) Patients following primary total knee arthroplasty due to osteoarthritis; 2) patients with preoperative platelet and coagulation function dysfunction; 3) preoperative double lower limb venous colour Doppler ultrasound was not abnormal; 4) voluntarily participated in clinical trials and signed informed consent, good compliance of patients
Interventions	**Intervention 1** 2 g oral TXA 2 hrs preoperatively **Intervention 2** 2 g oral TXA 2 hrs preoperatively, and 1 g oral TXA 3 hrs postoperatively **Intervention 3** 2 g oral TXA 2 hrs preoperatively, and 1g oral TXA 3 hrs and 9 hrs postoperatively **Comparator** 2 g oral TXA 2 hrs preoperatively, and 1 g oral TXA 3 hrs, 9 hrs and 15 hrs postoperatively
Outcomes	Total blood loss Haemoglobin Haematocrit Allogeneic blood transfusion DVT PE Wound complications Adverse events Intraoperative blood loss Hidden blood loss
Notes	Status on trial registry "completed". Trial registry page last updated December 2017. RC emailed author for update on the current status of the trial on 1 March 2022 (and on 3 occasions thereafter) but no response. Trial status remains "completed" as of 29 November 2022.

**Table:** ChiCTR1800015834

Methods	Randomised controlled trial
Participants	Patients aged 18 years and above, with a BMI of 20 to 35 kg/m^2^, ASA grade I‐III, with osteoarthritis or osteonecrosis of the femoral head, undergoing total hip arthroplasty
Interventions	**Intervention 1** Tranexamic acid (IV injection) + carbazochrome sodium sulfonate (infiltration of the surrounding tissue of the capsule + IV injection) **Intervention 2** Tranexamic acid (IV injection) + carbazochrome sodium sulfonate (infiltration of the surrounding tissue of the capsule) **Intervention 3** Tranexamic acid (IV injection) + carbazochrome sodium sulfonate (IV injection) **Comparator** Tranexamic acid (IV injection)
Outcomes	Total blood loss Haemoglobin Haematocrit Reduction in haemoglobin concentration D‐dimer Intraoperative blood loss Transfusion rates Thromboelastographic Inflammatory cytokines Range of motion of the hip Length of postoperative hospital stay
Notes	"Complete but not yet analysed". June 2021, email sent to authors for an update. Authors replied 3 August 2021 saying the 'experiment' has finished, but data are yet to be analysed. RC last accessed page on 25 November 2022: status remains unchanged.

**Table:** ChiCTR1900026092

Methods	Randomised controlled trial
Participants	1) Patients with primary unilateral total knee arthroplasty due to terminal knee disease (osteoarthritis); 2) patients who voluntarily participate in clinical trials and sign informed consent; 3) no abnormalities were found in the preoperative colour doppler ultrasound of both lower extremities; 4) patients who have good compliance
Interventions	**Intervention 1** 1 g of TXA was given at POD1 21:00 AM, POD2 09:00 AM and 21:00 PM respectively **Intervention 2** POD1 and POD2 were given 10 mg and 5 mg dexamethasone at 09:00 AM respectively **Comparator** POD1 10 mg of dexamethasone was given at 09:00 AM and 1 g of TXA was given at 21:00 PM. POD2 5 mg dexamethasone, then 1 g TXA at 09:00 AM and 1 g TXA at 21:00 PM.
Outcomes	Readmission Reoperation Wound complications Gastrointestinal haemorrhage
Notes	Status on trial registry "not yet recruiting". RC last accessed trial page 29 November 2022: trial information was last updated in September 2019. Author emailed to check on current status of the trial but had no response.

**Table:** ChiCTR1900027416

Methods	Randomised controlled trial
Participants	Patients aged 18 years and over, diagnosed with osteoarthritis of the hip joint and necrosis of the femoral head with unilateral initial artificial total hip arthroplasty indication
Interventions	**Intervention 1** TXA IV **Intervention 2** TXA plus dexamethasone **Intervention 3** Dexamethasone IV **Comparator** Placebo
Outcomes	Plasma immunoglobulin (lgG, lgA, lgM, lgE) Complement (C3, C4), Lymphocyte subsets (CD3 lymphocyte, CD4 lymphocyte, CD8 lymphocyte and CD4/CD8 ratio) Inflammatory marker Routine blood
Notes	Status on trial registry "not yet recruiting". RC emailed author for update on current status of the trial on 23 February 2022 (and on 3 occasions thereafter), but has had no response. Trial registry last accessed on 29 November 2022 where trial status remains as "not yet recruiting".

**Table:** ChiCTR2000032271

Methods	Randomised controlled trial
Participants	Primary unilateral total hip arthroplasty Patients with end‐stage osteoarthritis or ONFH (osteonecrosis of the femoral head) Able and willing to provide signed informed consent
Interventions	**Intervention** TXA **Comparator** EACA
Outcomes	Blood loss Transfusion rate Transfusion units Maximum haemoglobin reduction Length of hospital stay Haemoglobin level postoperatively Complication Haematocrit ESR C‐reactive protein
Notes	Status on trial registry "not yet recruiting". RC emailed author for update on the current status of the trial on 1 March 2022 (and on 3 occasions thereafter) but no response. Trial status remains "not yet recruiting" as of 29 November 2022.

**Table:** CTRI/2018/02/012030

Methods	Randomised controlled trial
Participants	Patients aged 18 to 80 years undergoing primary, unilateral hip replacement
Interventions	**Intervention 1** Topical TXA **Intervention 2** TXA IV **Comparator** Placebo
Outcomes	Total blood loss Change in haematocrit Operation time Transfusion rates Deep venous thrombosis Pulmonary embolism rates
Notes	Status on trial registry "not yet recruiting". Trial registry page last updated February 2018. RC emailed author for update on the current status of the trial on 14 July 2022 (and on 3 occasions thereafter) but no response. Trial status remains "not yet recruiting" as of 29 November 2022.

**Table:** CTRI/2018/05/013588

Methods	Randomised controlled trial
Participants	Patients aged 18 to 80 years of age undergoing primary unilateral knee replacement
Interventions	**Intervention 1** Single bolus of 20 mg/kg IV TXA 20 mins before inflation of the tourniquet **Intervention 2** Two boluses of 10 mg/kg IV TXA 20 mins before inflation of the tourniquet and before deflation of the tourniquet **Intervention 3** Two boluses of 10 mg/kg IV TXA before deflation and three hours after 1st dose **Intervention 4** Two boluses of 10 mg/kg IV TXA before inflation and three hours after deflation **Comparator** Placebo (no treatment/control group)
Outcomes	Total blood loss Change in haematocrit Transfusion rates Deep venous thrombosis Pulmonary embolisation rates Operative times
Notes	Status on trial registry "completed". Trial registry page last updated September 2018. RC emailed author for update on the current status of the trial on 14 July 2021 (and on 3 occasions thereafter) but no response. Trial status remains "completed" as of 29 November 2022.

**Table:** CTRI/2018/08/015421

Methods	Randomised controlled trial
Participants	Adult patients, 18 to 65 years of either sex with ASA grade I and II undergoing primary elective unilateral hip joint arthroplasty by same surgeon
Interventions	**Intervention 1** Double dose of TXA (tranexamic acid at 15 mg/kg before skin incision and a second dose 6 hours after the first dose at a dose of 15 mg/kg) **Intervention 2** Single dose of TXA (tranexamic acid is administered at a decided dose as per protocol, which is 15 mg/kg) **Comparator** Control (no drug is administered)
Outcomes	Drop in Hct after 24 hours Blood transfusion requirement in the first 24 hours Decrease in inflammatory markers (IL6 and D‐dimer) after 12 hours compared to the baseline Complications, if any, during the stay in hospital
Notes	Status on trial registry "completed". Trial registry page last updated 17 August 2018. RC emailed author for update on the current status of the trial on 29 November 2022: author has applied for an extension, results to be published shortly.

**Table:** CTRI/2019/01/017105

Methods	Randomised controlled trial
Participants	Patients undergoing total knee replacement surgery
Interventions	**Intervention** TXA, IV 15 mg/kg **Comparator** TXA, IA 1.5g
Outcomes	Total blood loss Need for any blood transfusion Symptomatic deep venous thrombosis Wound complications Infection
Notes	Status on trial registry "not yet recruiting". Trial registry page last updated January 2019. RC emailed author for update on the current status of the trial on 23 February 2022 (and on 3 occasions thereafter) but no response. Trial status remains "not yet recruiting" as of 29 November 2022.

**Table:** CTRI/2019/09/021302

Methods	Randomised controlled trial
Participants	All consecutive patients requiring open reduction surgeries from orthopaedic ward, who had given informed consent
Interventions	**Intervention** 10 mg/kg TXA in 100 mL saline, 20 minutes before incision **Comparator** 100 mL normal saline
Outcomes	Decrease in blood loss Haemoglobin reduction Requirements of blood transfusion
Notes	Status on trial registry 'complete'. Trial registry page last updated September 2020. RC emailed author for update on the current status of the trial on 23 February 2022 (and on 3 occasions thereafter) but no response. Trial status remains 'complete' as of 29 November 2022. We have searched for a publication but have not found one so far and from what data are available on the trial registry, we believe that this trial registration does not relate to any current included trial.

**Table:** CTRI/2021/09/036855

Methods	Randomised controlled trial
Participants	Patients requiring major periarticular hip surgeries
Interventions	**Intervention** Tablets containing 1950 mg of TXA given 2 hours prior to incision for surgery and 2 g of injection tranexamic acid diluted in 20 mL normal saline given through postoperative drain **Comparator** Tablets containing calcium given orally 2 hours prior to incision; 20 mL normal saline given through drain postoperatively
Outcomes	Intra and postoperative blood loss Need for blood transfusion Thromboembolic events
Notes	Status on trial registry "not yet recruiting". Trial registry page last updated September 2021. RC emailed author for update on the current status of the trial on 29 November 2022 (and on 3 occasions thereafter) but no response. Trial status remains "not yet recruiting" as of 29 November 2022.

**Table:** DiFrancesco 2013

Methods	Randomised controlled trial
Participants	Patients undergoing unilateral total knee arthroplasty for osteoarthritis
Interventions	**Intervention** Haemostatic matrix as well as the standard method of haemostasis (haemostatic matrix group) **Comparator** Standard method of haemostasis only (control group)
Outcomes	Mean total postoperative blood loss Drainage volume Patient blood volume Haematocrit Red blood cell volume Drain output Transfusion requirements
Notes	Unable to find a trial registration and therefore cannot determine if the trial was prospectively registered.

**Table:** Digas 2015

Methods	Randomised controlled trial
Participants	Patients younger than 85 years with primary osteoarthritis who were awaiting total knee arthroplasty
Interventions	**Intervention 1** TXA IV **Intervention 2** TXA IA **Comparator** Control
Outcomes	Blood loss Need for transfusion
Notes	Unable to find a trial registration and therefore cannot determine if the trial was prospectively registered.

**Table:** EUCTR‐2013‐003169‐33‐DK

Methods	Randomised controlled trial
Participants	Those 18 years old and above undergoing surgery for unilateral total knee prothesis
Interventions	**Intervention** IV TXA at the start of surgery + injection of TXA into the joint (end surgery) **Comparator** IV TXA at the start of surgery + injection of equal volume of NaCl (placebo)
Outcomes	Total blood loss 24 hours after surgery Total bleeding 2 days after surgery Amount of blood transfusion Thromboembolic events within the first 90 days of surgery
Notes	Status on trial registry 'completed'. Trial registry page last updated March 2017. RC emailed author for update on the current status of the trial on 5 July 2021 (and on 3 occasions thereafter) but no response. Trial status remains 'completed' as of 25 November 2022. We have searched for a publication but have not found one so far and from what data is available on the trial registry, we believe that this trial registration does not relate to any current included trial.

**Table:** Falez 2013

Methods	Randomised controlled trial
Participants	Patients undergoing unilateral elective primary cementless total hip replacement
Interventions	**Intervention 1** Topical fibrin spray before closure **Intervention 2** Haemostasis with radiofrequency energy using a bipolar sealer **Comparator** Standard electrocautery
Outcomes	Peri‐operative blood loss Blood re‐infusion or transfusion Adverse events
Notes	Unable to find a trial registration and therefore cannot determine if the trial was prospectively registered.

**Table:** Fleischmann 2011

Methods	Randomised controlled trial
Participants	Patients undergoing elective implantation of cementless arthroplasty of the hip due to manifest coxarthrosis
Interventions	**Intervention** Aprotinin **Intervention 2** Placebo and suction drains **Comparator** External compression on the other
Outcomes	Blood loss Thrombosis Drug reaction Need for blood transfusion Erythrocytes Haemoglobin Infection rate Coagulation Platelet concentration Adverse events
Notes	Unable to find a trial registration and therefore cannot determine if the trial was prospectively registered.

**Table:** Gautam 2011

Methods	Randomised controlled trial
Participants	Patients undergoing elective primary unilateral total knee replacement for osteoarthritis
Interventions	**Intervention** TXA IV **Comparator** Placebo (saline)
Outcomes	Blood loss Blood transfusion details Change in haemoglobin levels
Notes	Unable to find a trial registration and therefore cannot determine if the trial was prospectively registered.

**Table:** Gautam 2013

Methods	Randomised controlled trial
Participants	Patients undergoing total knee replacement
Interventions	**Intervention** TXA IV **Comparator** Control (no drug)
Outcomes	Amount of blood loss Haemoglobin and haematocrit loss Number of transfusions
Notes	Unable to find a trial registration and therefore cannot determine if the trial was prospectively registered.

**Table:** Gomez Barbero 2019

Methods	Randomised controlled trial
Participants	Patients who underwent elective total hip arthroplasty due to coxarthrosis or avascular necrosis
Interventions	**Intervention** TXA IV **Comparator** TXA IA
Outcomes	Preoperative and postoperative haemoglobin and haematocrit Lowest haemoglobin recorded during the hospital stay Patient’s blood volume Total blood loss Hidden blood loss Drainage volume Number of blood units transfused Complications (the wound developing infection or necrosis, DVT, PTE or death)
Notes	Unable to find a trial registration and therefore cannot determine if the trial was prospectively registered.

**Table:** Gulabi 2019

Methods	Randomised controlled trial
Participants	Patients with primary osteoarthritis scheduled for elective unilateral primary total knee arthroplasty
Interventions	**Intervention** TXA IV **Comparator** TXA IV + topical TXA
Outcomes	Preoperative and postoperative haemoglobin levels Postoperative transfusion records and 90‐day joint‐related readmission rate Implant subsidence Dislocation Postoperative anemia Deep infection Haematoma Wound problem Postoperative periprosthetic fracture Complication rate
Notes	Unable to find a trial registration and therefore cannot determine if the trial was prospectively registered.

**Table:** Guzel 2016

Methods	Randomised controlled trial
Participants	Patients underwent primary unilateral total knee arthroplasty
Interventions	**Intervention 1** PAT (postoperative autologous transfusion) **Intervention 2** Topical TXA **Comparator** Routine drainage (control)
Outcomes	Pre‐ and postoperative haemoglobin level Total postoperative drainage volume Need for allogeneic blood transfusion
Notes	Unable to find a trial registration and therefore cannot determine if the trial was prospectively registered.

**Table:** Hongshun 2019

Methods	Randomised controlled trial
Participants	Patients undergoing primary unilateral total hip arthroplasty
Interventions	**Intervention** TXA IV **Comparator** Placebo (saline)
Outcomes	Intraoperative blood loss Postoperative drainage volume Total blood loss Hidden blood loss Blood transfusion volume Transfusion rate Haemoglobin Haematocrit Levels of C‐reactive protein Interleukin‐6 Complications (fever or lower extremity venous thrombosis)
Notes	Unable to find a trial registration and therefore cannot determine if the trial was prospectively registered.

**Table:** Hou 2015

Methods	Randomised controlled trial
Participants	Patients undergoing primary unilateral cemented total knee arthroplasty
Interventions	**Intervention 1** 0.9% NS 100 mL was given at the beginning of the operation, and 0.9% NS 10 mL was injected into the joint cavity after suture **Intervention 2** Tranexamic acid (10 mg/kg diluted in 0.9% NS 100 mL) was given at the beginning of the operation, and 0.9% NS 10 ml was injected into the joint cavity after suture **Comparator** 0.9% NS 100 mL was given at the beginning of the operation, and intra‐articular injection of tranexamic acid (500 mg in 0.9% NS 10 mL) was injected into the joint
Outcomes	Dominant blood loss Hidden blood loss Blood transfusion ratio and per capita Clinical symptoms of pulmonary embolism and lower limb deep vein thrombosis
Notes	Unable to find a trial registration and therefore cannot determine if the trial was prospectively registered.

**Table:** Hsu 2015

Methods	Randomised controlled trial
Participants	Patients with osteoarthritis of the hip secondary to degeneration, inflammatory arthritis, gouty arthritis, acetabular dysplasia or osteonecrosis of the femoral head undergoing primary unilateral minimally invasive total hip arthroplasty
Interventions	**Intervention** TXA IV **Comparator** Placebo
Outcomes	Length of hospital stay Operating time Number of red cell transfusions Levels of haemoglobin Intraoperative blood loss Total drainage Complications
Notes	Unable to find a trial registration and therefore cannot determine if the trial was prospectively registered.

**Table:** Hu 2018

Methods	Randomised controlled trial
Participants	Patients with unilateral knee osteoarthritis undergoing total knee arthroplasty
Interventions	**Intervention 1** Tranexamic acid was administered intravenously before and after wound closure **Intervention 2** Tranexamic acid in 20 mL of normal saline was injected into the articular cavity through the drainage after wound closure and the tube was clamped for 4 hours **Comparator** No tranexamic acid administration
Outcomes	Intraoperative blood loss Haemoglobin level at postoperative 24 and 48 hours Postoperative drainage volume Incidence of deep venous thrombosis
Notes	Unable to find a trial registration and therefore cannot determine if the trial was prospectively registered.

**Table:** Huang 2014

Methods	Randomised controlled trial
Participants	Patients undergoing unilateral total knee arthroplasty
Interventions	**Intervention** TXA IV **Comparator** TXA IV + topical TXA
Outcomes	Transfusion rate Blood loss Haemoglobin (Hb) Drainage volume Postoperative knee pain Knee swelling Length of hospital stay Short‐term satisfaction
Notes	Unable to find a trial registration and therefore cannot determine if the trial was prospectively registered.

**Table:** Imai 2012

Methods	Randomised controlled trial
Participants	Patients with osteoarthritis of the hip joint undergoing total hip arthroplasty
Interventions	**Intervention 1** Untreated control group (no drug was administered) **Intervention 2** 1 g of TXA administered 10 minutes before skin closure to avoid fibrinolytic inhibition during the phase of operation and to exploit the effect of TXA maximally in the postoperative phase (T1 group) **Intervention 3** 1 g of TXA administered 10 minutes before skin closure and again at 6 hours after the first administration **Intervention 4** 1 g of TXA administered 10 minutes before surgery (T3 group) **Comparator** 1 g of TXA administered 10 minutes before surgery and again at 6 hours after the first administration
Outcomes	Intraoperative blood loss Postoperative blood loss Haemoglobin
Notes	Unable to find a trial registration and therefore cannot determine if the trial was prospectively registered.

**Table:** IRCT20120910010800N3

Methods	Randomised controlled trial
Participants	Patients over 18 years of age, who had given consent to the study
Interventions	**Intervention** In the fibrinogen group (case), patients will receive 1 g of this drug in a volume of 50 mL of distilled water through an infusion pump within 10 minutes of intravenous injection **Comparator** In the control group, 50 mL of distilled water used in the same way as above
Outcomes	Bleeding Transfusion
Notes	Status on trial registry 'recruitment complete'. Trial registry page last updated April 2022. Trial status remains 'recruitment complete' as of 5 December 2022. We have searched for a publication but have not found one so far and, from what data is available on the trial registry, we believe that this trial registration does not relate to any current included trial.

**Table:** Jain 2016

Methods	Randomised controlled trial
Participants	Patients with diagnosis of primary osteoarthritis (OA) undergoing unilateral total knee arthroplasty
Interventions	**Intervention** TXA IV **Comparator** TXA IV + intra‐articular TXA
Outcomes	Total blood loss Allogeneic blood transfusion rate Postoperative Hb drop Incidences of symptomatic DVT and TE
Notes	Unable to find a trial registration and therefore cannot determine if the trial was prospectively registered.

**Table:** Jaszczyk 2015

Methods	Randomised controlled trial
Participants	Patients undergoing total cementless hip arthroplasty
Interventions	**Intervention** TXA IV **Comparator** Control (no drug)
Outcomes	Intraoperative blood loss Postoperative blood loss Total perioperative blood loss Number of patients requiring transfusion Number of thromboembolic complications in both groups
Notes	Unable to find a trial registration and therefore cannot determine if the trial was prospectively registered.

**Table:** Jia 2019

Methods	Randomised controlled trial
Participants	Patients with hip osteoarthritis or femoral head osteonecrosis undergoing elective unilateral primary total hip arthroplasty
Interventions	**Intervention 1** TXA IV **Intervention 2** Topical TXA **Comparator** Combined IV and IA TXA
Outcomes	Total blood loss Allogeneic blood transfusion rate Maximum haemoglobin drop Haematocrit change Incidence of DVT and PE Length of hospital stay Complications (wound leakage, haematoma, superficial infection and deep infection) Adverse events (cardiac infarction, stroke and acute renal failure)
Notes	Unable to find a trial registration and therefore cannot determine if the trial was prospectively registered.

**Table:** Karaaslan 2015

Methods	Randomised controlled trial
Participants	Patients with a diagnosis of osteoarthritis scheduled to have primary, simultaneous bilateral total knee arthroplasty
Interventions	**Intervention** TXA IV + TXA IA **Comparator** Control (no TXA)
Outcomes	Volume of drained blood Decrease in haemoglobin levels Amount of blood transfused Number of patients requiring allogenic blood transfusion
Notes	Unable to find a trial registration and therefore cannot determine if the trial was prospectively registered.

**Table:** Kazemi 2010

Methods	Randomised controlled trial
Participants	Patients who were candidates for cementless total hip arthroplasty
Interventions	**Intervention** TXA IV **Comparator** Saline
Outcomes	Duration of surgery Units of blood given Volume of blood given Volume of blood lost Mean blood loss Mean number of patients who needed a transfusion Haematocrit level Length of hospital stay Thromboembolic events
Notes	Unable to find a trial registration and therefore cannot determine if the trial was prospectively registered.

**Table:** Keyhani 2016

Methods	Randomised controlled trial
Participants	Patients with knee osteoarthritis scheduled to undergo unilateral total knee arthroplasty
Interventions	**Intervention 1** TXA IV **Intervention 2** Topical IA TXA **Comparator** Control
Outcomes	Volume of bleeding Level of Hb Frequency of transfusion Number of packed red blood cells transfused Complications
Notes	Unable to find a trial registration and therefore cannot determine if the trial was prospectively registered.

**Table:** Kim 2014

Methods	Randomised controlled trial
Participants	Patients undergoing unilateral or bilateral total knee arthroplasty
Interventions	**Intervention** TXA IV **Comparator** Control
Outcomes	Total blood loss Allogenic transfusion rate Rate of autologous transfusion with preoperative autologous blood donation Blood loss via the drain Postoperative Hb drop Proportions of patients with the Hb level below the three cut‐off values, namely 7.0, 8.0 and 9.0 g/dL Incidences of symptomatic DVT and PE Functional outcomes
Notes	Unable to find a trial registration and therefore cannot determine if the trial was prospectively registered.

**Table:** Kundu 2015

Methods	Randomised controlled trial
Participants	Patients scheduled for unilateral total knee replacement
Interventions	**Intervention** TXA IV **Comparator** Placebo
Outcomes	Duration of operation Total blood loss Need for transfusion Thromboembolic complications
Notes	Unable to find a trial registration and therefore cannot determine if the trial was prospectively registered.

**Table:** Kusuma 2013

Methods	Randomised controlled trial
Participants	Patients with a diagnosis of osteoarthritis preparing to undergo total knee arthroplasty
Interventions	**Intervention** IA bovine thrombin at the time of wound closure **Comparator** Control (identical closure and drain placement protocol without the thrombin infusion)
Outcomes	Haemoglobin Drain output Transfusion requirements Length of hospital stay Bleeding related complications Knee Society Score
Notes	Unable to find a trial registration and therefore cannot determine if the trial was prospectively registered.

**Table:** Lacko 2017

Methods	Randomised controlled trial
Participants	Patients with knee osteoarthritis undergoing a unilateral cemented total knee replacement
Interventions	**Intervention 1** TXA IV (dose 10 mg/kg) 20 minutes preoperatively and 3 hours after first dose **Intervention 2** TxA (dose 3 g) locally (intra‐articular) into surgical site **Comparator** No TXA
Outcomes	Perioperative blood loss Volume of drained blood Overall blood loss Decrease in haemoglobin and haematocrit levels Amount of blood transfusion
Notes	Unable to find a trial registration and therefore cannot determine if the trial was prospectively registered.

**Table:** Lee 2013

Methods	Randomised controlled trial
Participants	Patients undergoing primary unilateral cementless total hip replacement
Interventions	**Intervention** TXA IV **Comparator** Saline
Outcomes	Intraoperative blood loss Postoperative blood loss Duration of anaesthesia and surgery Controlled hypotension Hospital length of stay Quantity of infused fluids Transfused blood Amount of blood loss (intraoperative, postoperative and total) Frequency of transfusion (intraoperative, postoperative and total) Number of transfused PRBC units Haemoglobin and Hct values Prothrombin time (PT), activated partial thromboplastin time (aPTT) and platelet count Urine output Cognitive dysfunction Lower‐limb DVT
Notes	Unable to find a trial registration and therefore cannot determine if the trial was prospectively registered.

**Table:** Lee 2013a

Methods	Randomised controlled trial
Participants	Patients with primary osteoarthritis undergoing unilateral total knee arthroplasty
Interventions	**Intervention** TXA IV **Comparator** Placebo
Outcomes	Postoperative retransfusion volume Allogenic transfusion volumes Drain amount Level of haemoglobin Prothrombin time Activated partial thromboplastin time D‐dimer
Notes	Unable to find a trial registration and therefore cannot determine if the trial was prospectively registered.

**Table:** Lee 2017

Methods	Randomised controlled trial
Participants	Patients scheduled for elective unilateral primary total knee arthroplasty
Interventions	**Intervention 1** TXA IV only **Intervention 2** TXA IA only **Intervention 3** Low‐dose combined (IV + IA injection of 1 g) **Comparator** High‐dose combined (IV + IA injection of 2 g)
Outcomes	Calculated total blood loss Allogeneic transfusion rate Decrease in haemoglobin Frequency of symptomatic deep vein thrombosis and pulmonary embolism Wound complications Periprosthetic joint infection
Notes	Unable to find a trial registration and therefore cannot determine if the trial was prospectively registered.

**Table:** Lee 2017a

Methods	Randomised controlled trial
Participants	Patients undergoing primary total knee arthroplasty
Interventions	**Intervention** Oral TXA **Comparator** No TXA
Outcomes	Hb drop Intraoperative blood loss Drain output Total blood loss Hidden blood loss Transfusion requirement Thromboembolic complications Cerebrovascular or cardiovascular complications 30‐day mortality Proximal DVT
Notes	Unable to find a trial registration and therefore cannot determine if the trial was prospectively registered.

**Table:** Lei 2020a

Methods	Randomised controlled trial ‐ study protocol
Participants	Patients with rheumatoid arthritis undergoing total knee arthroplasty
Interventions	**Intervention** TXA 1.5 g twice daily starting from 3 days before the operation **Comparator** 100 mL normal saline twice daily starting from 3 days before the operation
Outcomes	Total blood loss Hidden blood loss Fibrinolysis parameters Inflammatory factors Visual analogue scale for postoperative pain Analgesia usage Coagulation parameters Transfusion Length of stay Total hospitalisation costs Incidence of thromboembolic events Other complications
Notes	Status on trial registry "not yet recruiting". Trial registry page last updated February 2020. Trial status remains "not yet recruiting" as of 29 November 2022.

**Table:** Li‐Qing 2018

Methods	Randomised controlled trial
Participants	Patients undergoing unilateral total knee arthroplasty
Interventions	**Intervention 1** The patients in group A received the intra‐articular injection normal saline (50 mL), drainage tube was placed postoperatively and closed for 4 hours **Intervention 2** Group B: the mixture of 50 mL of normal saline and 1.6 g of tranexamic acid was injected into the articular cavity, drainage tube was placed and closed for 4 hours **Comparator** Group C: the mixture of 50 mL of normal saline and 1.6 g tranexamic acid was injected into the articular cavity without drainage tube placed
Outcomes	Intraoperative blood loss Haemoglobin Drainage volume Blood transfusion volume Number of blood transfusion Total blood loss Hidden blood loss Number of deep vein thromboses of the lower extremity
Notes	Unable to find a trial registration and therefore cannot determine if the trial was prospectively registered.

**Table:** Liebelt 2013

Methods	Randomised controlled trial
Participants	Patients undergoing routine unilateral total knee arthroplasty
Interventions	**Intervention** Standard haemostatic therapy **Comparator** Standard haemostatic therapy + Floseal
Outcomes	Postoperative drain output Drop in haemoglobin and haematocrit levels Incidence of postoperative transfusions
Notes	Unable to find a trial registration and therefore cannot determine if the trial was prospectively registered.

**Table:** Lin 2015

Methods	Randomised controlled trial
Participants	Patients with a diagnosis of primary osteoarthritis undergoing unilateral total knee arthroplasty
Interventions	**Intervention 1** 1 g (100 mg/mL) of TXA in 20 mL normal saline using intra‐articular application intraoperatively after joint capsule closure (topical group) **Intervention 2** 1 g of TXA using IV injection 15 minutes before skin incision and 1 g of TXA using intra‐articular application intraoperatively after joint capsule closure (combined group) **Comparator** 20 mL of normal saline using intra‐articular application intraoperatively after joint capsule closure (control group)
Outcomes	Postoperative Hb levels Hb drop Total drain amount Total blood loss Transfusion rate Hb level Haematocrit Platelet count Prothrombin time Partial thromboplastin time Electrolyte Liver enzyme Renal function test
Notes	Unable to find a trial registration and therefore cannot determine if the trial was prospectively registered.

**Table:** Liu 2018

Methods	Randomised controlled trial
Participants	Patients undergoing total knee arthroplasty
Interventions	**Intervention 1** IV infusion of 10 mg/kg TXA **Intervention 2** IV infusion of 15 mg/kg TXA **Comparator** IV infusion of normal saline
Outcomes	Postoperative blood loss Occult blood loss Blood transfusion rate Blood transfusion volume Haemoglobin levels Fibrinogen levels Prothrombin time Activated partial thromboplastin time D‐dimer level Thrombin time VAS Circumference diameter of knee HSS Recovery of knee function
Notes	Unable to find a trial registration and therefore cannot determine if the trial was prospectively registered.

**Table:** Liu 2021

Methods	Randomised controlled trial
Participants	Patients undergoing primary unilateral total knee arthroplasty
Interventions	**Intervention 1** Group A were intravenously injected with TXA (1 g:100 mL) combined with periarticular multipoint infiltration injection of 50 mL normal saline before the implantation of prosthesis **Intervention 2** Group B were intravenously injected with TXA (1 g:100 mL) combined with periarticular multipoint infiltration injection of 50 mL cocktail (0.3 mL epinephrine, 10 mL ropivacaine, 0.5 mL morphine, 39.2 mL normal saline) before the implantation **Comparator** Group C were intravenously injected with TXA (1 g:100 mL) combined with periarticular multipoint infiltration injection of cocktail complex TXA (1 g:10 mL), a total of 50 mL, before the implantation
Outcomes	Total blood loss Drainage volume Hidden blood loss Maximum haemoglobin decline Transfusion rate Transfusion volume Visual analogue scale Incidence of deep vein thrombosis and other adverse events
Notes	Unable to find a trial registration and therefore cannot determine if the trial was prospectively registered.

**Table:** Lopez‐Hualda 2018

Methods	Randomised controlled trial
Participants	Patients undergoing primary total knee arthroplasty
Interventions	**Intervention 1** 1 g of topical TXA **Intervention 2** 1 g of TXA IV **Comparator** Control group (no drug was administered)
Outcomes	Blood loss Drain outputs
Notes	Unable to find a trial registration and therefore cannot determine if the trial was prospectively registered.

**Table:** Luo 2018

Methods	Randomised controlled trial
Participants	Patients scheduled for primary unilateral total hip arthroplasty for osteoarthritis or osteonecrosis of the femoral head
Interventions	**Intervention** 2 g TXA orally 2 hrs preoperatively, and 2 doses of 1 g TXA postoperatively (oral group) **Comparator** 3 g of TXA topical administration in the operating room (topical group)
Outcomes	Reduction in haemoglobin concentration Blood loss TXA‐related cost Length of hospital stay Complications such as pulmonary thromboembolism (PE), deep vein thrombosis (DVT), and infection Blood coagulation and fibrinolysis Hip function
Notes	Unable to find a trial registration and therefore cannot determine if the trial was prospectively registered.

**Table:** Ma 2014

Methods	Randomised controlled trial
Participants	Patients undergoing unilateral total knee arthroplasty
Interventions	**Intervention** IA TXA **Comparator** Saline
Outcomes	Amounts of intraoperative and postoperative blood loss Blood transfusion Postoperative drainage volume Preoperative and postoperative limb circumference
Notes	Unable to find a trial registration and therefore cannot determine if the trial was prospectively registered.

**Table:** MacGillivray 2011

Methods	Randomised controlled trial
Participants	Patients undergoing concurrent bilateral total knee arthroplasty
Interventions	**Intervention 1** 2 doses of IV TXA of 10 mg/kg **Intervention 2** 2 doses of IV TXA of 15 mg/kg **Comparator** 2 equal volumes of normal saline (the control group)
Outcomes	Volumes collected by the intraarticular drains Retransfusion of autologous blood Postoperative Hb level Haematocrit Platelets PI INR aPTT Thromboembolic complications Transfusion volumes
Notes	Unable to find a trial registration and therefore cannot determine if the trial was prospectively registered.

**Table:** Malhotra 2011

Methods	Randomised controlled trial
Participants	Patients undergoing unilateral, cementless total hip arthroplasty
Interventions	**Intervention** IV TXA **Comparator** IV saline placebo
Outcomes	Blood loss during the operation Amount of drainage after the operation Postoperative blood loss Amount of transfused units of red cells Drop in haemoglobin levels Rise in D‐dimer value Possible complications
Notes	Unable to find a trial registration and therefore cannot determine if the trial was prospectively registered.

**Table:** Maniar 2012

Methods	Randomised controlled trial
Participants	Patients with a diagnosis of osteoarthritis scheduled to have primary, unilateral total knee arthroplasty
Interventions	**Intervention 1** 10 mg/kg 15 minutes before deflation of the tourniquet as an intraoperative dose (IO group) **Intervention 2** 10 mg/kg 15 minutes before deflation of the tourniquet as an intraoperative dose and 10 mg/kg 3 hours after the first dose as a postoperative dose (IOPO group) **Intervention 3** 10 mg/kg at least 20 minutes before tourniquet inflation as a preoperative dose and 10 mg/kg 15 minutes before deflation of the tourniquet as an intraoperative dose (POIO group) **Intervention 4** 10 mg/kg 20 minutes before tourniquet application as a preoperative dose, 10 mg/kg 15 minutes before deflation of the tourniquet as an intraoperative dose, and 10 mg/kg 3 hours after the second dose as a postoperative dose (POIOPO group) **Comparator** 3 g diluted in 100 mL normal saline applied locally after cementing the implant and before tourniquet release (LA group)
Outcomes	Drain loss Total blood loss Number of transfusions Adverse events
Notes	Unable to find a trial registration and therefore cannot determine if the trial was prospectively registered.

**Table:** May 2016

Methods	Randomised controlled trial
Participants	Patients undergoing primary total knee arthroplasty
Interventions	**Intervention** Intracapsular TXA **Comparator** IV TXA
Outcomes	Total blood loss Total number of blood transfusions required
Notes	Unable to find a trial registration and therefore cannot determine if the trial was prospectively registered.

**Table:** McDonald 2017

Methods	Randomised controlled trial
Participants	Patients undertaking elective knee arthroplasty
Interventions	**Intervention** Oral TXA **Comparator** Placebo
Outcomes	Hb Haematocrit Leg circumference Knee flexion Wound appearance
Notes	Unable to find a trial registration and therefore cannot determine if the trial was prospectively registered.

**Table:** McDonald 2022

Methods	Randomised controlled trial
Participants	Patients scheduled for a primary total knee arthroplasty for severe symptomatic osteoarthritis
Interventions	**Intervention** Oral TXA group **Comparator** Oral placebo group
Outcomes	Total blood loss Hb drop
Notes	We are unable to find the registry entry for this trial to confirm prospective registration. Full paper is available. Author emailed and confirmed the trial registration number was that listed in the paper, but the number given does not relate to a published trial registration.

**Table:** Mehta 2019

Methods	Randomised controlled trial
Participants	Patients undergoing bilateral total knee arthroplasty
Interventions	**Intervention 1** TXA, IV 1 g **Intervention 2** TXA, IA 2.5 g **Comparator** Placebo
Outcomes	Total amount of blood loss (intraoperative blood loss and postoperative blood loss through suction drains of both knees) Pre‐ and post‐surgery haemoglobin levels Transfusion rate and transfusion quantity Complications such as superficial or deep infection, wound dehiscence, DVT, pulmonary embolism or any organ dysfunction
Notes	We are unable to find a trial registration number for this study. We have emailed the authors but had no response to multiple emails, therefore cannot confirm whether the trial was prospectively registered.

**Table:** Min 2015

Methods	Randomised controlled trial
Participants	Patients with primary osteoarthritis undergoing unilateral total knee arthroplasty
Interventions	**Intervention** IV TXA **Comparator** Placebo
Outcomes	Fibrinogen Prothrombin time Partial prothrombin time D‐dimer Haemoglobin Number of transfused patients Amount of blood transfusion Amount of blood loss
Notes	Unable to find a trial registration and therefore cannot determine if the trial was prospectively registered.

**Table:** Moo 2017

Methods	Randomised controlled trial
Participants	Patients diagnosed with primary osteoarthritis scheduled for an elective primary total knee arthroplasty
Interventions	**Intervention** Bone wax group received 2.5 g of bone wax **Comparator** Haemostasis achieved using electrocautery only
Outcomes	Hb Haematocrit Tourniquet time Total blood loss Blood transfusion Postoperative lower limb swelling DVT PE Complications
Notes	Unable to find a trial registration and therefore cannot determine if the trial was prospectively registered.

**Table:** Na 2016

Methods	Randomised controlled trial
Participants	Patients diagnosed with either avascular necrosis of the femoral head or degenerative arthritis of the hip who were scheduled to undergo primary total hip replacement
Interventions	**Intervention** IV TXA **Comparator** Placebo
Outcomes	Results of the ROTEM (rotational thromboelastometry) analyses Laboratory results (haemoglobin, haematocrit, platelets, PT‐INR, aPTT and fibrinogen) Input (infused volume of crystalloid and colloid) Output (intra‐ and postoperative blood loss and urine output) Transfusion of blood components Intraoperative blood loss (IBL) Postoperative blood loss (PBL) Total blood loss (TBL)
Notes	Unable to find a trial registration and therefore cannot determine if the trial was prospectively registered.

**Table:** Nambiar 2019

Methods	Randomised controlled trial
Participants	Patients undergoing total knee replacement
Interventions	**Intervention** IV TXA 10 mg/kg **Comparator** IV TXA 15 mg/kg
Outcomes	Haemodynamic parameters Haemoglobin levels Transfusions Thromboembolic complications Duration of hospital stay
Notes	Unable to find a trial registration and therefore cannot determine if the trial was prospectively registered.

**Table:** NCT00983112

Methods	Randomised controlled trial
Participants	Patients having total knee prosthesis surgery
Interventions	**Intervention** Evicel (human fibrinogen and human thrombin) **Comparator** Placebo
Outcomes	Blood loss Rate of cell saver transfusion Rate of red blood cell transfusion Haematoma size Site incision state Rest and movement pain Antalgic consumption Functional recovering index Major rate or clinically significative haemorrhage Infectious complications Rate of SUSARs Rate of thrombotic events
Notes	Status on trial registry 'unknown'. Trial registry page last updated April 2012. RC emailed author for update on the current status of the trial on 1 March 2021 (and on 3 occasions thereafter), but no response. Trial status remains 'unknown' as of 29 November 2022. We have searched for a publication but have not found one so far that relates to the study details provided in the trial registration.

**Table:** NCT01260818

Methods	Randomised controlled trial
Participants	Patients undergoing elective total primary hip arthroplasty (single‐side)
Interventions	**Intervention** Topical TXA **Comparator** Topical saline
Outcomes	Blood loss (change in haematocrit) Blood loss (intraoperative and postoperative) Haemoglobin levels Drainage volume Proportion of patients receiving transfusions Incidence of postoperative deep venous thrombosis
Notes	Status on trial registry 'unknown'. Trial registry page last updated December 2011. RC emailed author for update on the current status of the trial on 1 March 2021 (and on 3 occasions thereafter), but no response. Trial status remains 'unknown' as of 2 November 2022. We have searched for a publication but have not found one so far that relates to the study details provided in the trial registration.

**Table:** NCT01391182

Methods	Randomised controlled trial
Participants	Patients with a preoperative haemoglobin between 10.0 and 13.5, scheduled for a primary total hip arthroplasty
Interventions	**Intervention** Epsilon aminocaproic acid **Comparator** Placebo
Outcomes	Haemoglobin levels Transfusion rates
Notes	Status on trial registry 'complete'. Trial registry page last updated February 2015. No results posted, no author email address, so no contact possible. We have searched for a publication but have not found one so far that relates to the study details provided in the trial registration.

**Table:** NCT01527968

Methods	Randomised controlled trial
Participants	Patients scheduled to receive primary unilateral total knee arthroplasty
Interventions	**Intervention 1** TXA **Intervention 2** EACA **Comparator** Placebo
Outcomes	Blood loss
Notes	Status on trial registry 'unknown'. Trial registry page last updated July 2016. No author contact email. No results posted. We have searched for a publication but have not found one so far that relates to the study details given in the trial registration.

**Table:** NCT02056444

Methods	Randomised controlled trial
Participants	Patients undergoing primary elective total hip arthroplasty using the cementless total hip implant system
Interventions	**Intervention** Topical TXA **Comparator** IV TXA
Outcomes	Change in haemoglobin Calculated blood loss Venous thromboembolic event Acute coronary syndrome Cerebrovascular accident Acute kidney injury Pneumonia Other systemic illness/infection Number of units of packed red blood cells transfused Haematoma Length of stay in hospital Systemic serum tranexamic acid (TXA) levels
Notes	Status on trial registry 'complete'. Trial registry page last updated 2016. RC emailed author for update 6 July 2021. Author replied 3 August 2021, saying the trial was presented as a master's thesis and not written up for publication.

**Table:** NCT02117128

Methods	Randomised controlled trial
Participants	Adult patients who plan to undergo primary total knee arthroplasty on unilateral knee joint with a diagnosis of osteoarthritis or aseptic bone necrosis, but not of rheumatoid arthritis
Interventions	**Intervention 1** IA TXA **Intervention 2** IV TXA **Comparator** IA TXA + IV TXA
Outcomes	Average amounts of transfusion Calculated blood loss Thrombosis
Notes	Status on trial registry 'complete'. Trial registry page last updated March 2016. RC emailed author for update on the current status of the trial on 1 March 2022 (and on three occasions thereafter), but no response. Trial status remains 'complete' as of 29 November 2022. We have searched for a publication but have not found one so far that relates to the study details given in the trial registration.

**Table:** NCT02286973

Methods	Randomised controlled trial
Participants	Patients with diagnosis of primary osteoarthritis
Interventions	**Intervention 1** IV TXA **Intervention 2** IV TXA + topical TXA 1 g **Intervention 3** IV TXA + topical TXA 2 g **Comparator** Topical TXA
Outcomes	Change in haemoglobin Blood loss
Notes	Status on trial registry 'unknown'. Trial registry page last updated November 2014. RC emailed author for update on the current status of the trial on 19 July 2021 (and on 3 occasions thereafter), but no response. Trial status remains 'unknown' as of 29 November 2022. We have searched for a publication but have not found one so far that relates to the study details given in the trial registration.

**Table:** NCT02438566

Methods	Randomised controlled trial
Participants	Patients aged 18 years and over undergoing hip or bilateral knee replacement surgery
Interventions	**Intervention** Oral TXA **Comparator** IV TXA
Outcomes	Number of units of blood required for transfusion Incidence of patients requiring blood transfusion Blood loss Length of hospital stay
Notes	Status on trial registry 'unknown'. Trial registry page last updated March 2016. RC emailed author 7 July 2021. Kenneth Bauer (author) replied to email on 8 July 2021. He said "My name was assigned to this trial after PI retired back in 2014, but I don't think the data was ever analyzed." We have searched for a publication but have not found one so far that relates to the study details given in the trial registration.

**Table:** NCT02587845

Methods	Randomised controlled trial
Participants	Patients undergoing elective primary unilateral or revision THA, not for fractures
Interventions	**Intervention** IV TXA **Comparator** Topical TXA
Outcomes	Bleeding Allogeneic transfusion Operation time
Notes	Status on trial registry 'unknown'. Trial registry page last updated October 2015. RC emailed author for update on the current status of the trial on 14 July 2021 (and on 3 occasions thereafter), but no response. Trial status remains 'unknown' as of 29 November 2022. We have searched for a publication but have not found one so far that relates to the study details given in the trial registration.

**Table:** NCT02938962

Methods	Randomised controlled trial
Participants	Patients aged 18 and over at the time of surgery having revison hip arthroplasty for osteolysis, component failure, prosthetic joint infection, aseptic/septic loosening, periprosthetic fracture, recurrent instability/dislocation, polyethylene wear and abductor insufficiency
Interventions	**Intervention** IV TXA **Comparator** Topical TXA
Outcomes	Change in haemoglobin Allogeneic blood units transfused Length of stay Estimated intraoperative blood loss Postoperative complications
Notes	Status on trial registry "unknown". Trial registry page last updated October 2016. RC emailed author for update on the current status of the trial on 13 July 2021 (and on 3 occasions thereafter), but no response. Trial status remains "unknown" as of 29 November 2022. We have searched for a publication but have not found one so far that relates to the study details given in the trial registration.

**Table:** NCT03109652

Methods	Randomised controlled trial
Participants	Candidates for total knee replacement arthroplasty due to osteoarthritis of the knee
Interventions	**Intervention 1** IV TXA alone **Intervention 2** IV TXA and oral TXA 5 days **Comparator** IV TXA and oral TXA 2 days
Outcomes	Hb Transfusion rate and amount Complications Calculated blood loss
Notes	Status on trial registry "unknown". Trial registry page last updated January 2019. We have searched for a publication but have not found one so far that relates to the study details given in the trial registration.

**Table:** NCT03310060

Methods	Randomised controlled trial
Participants	Patients classified as low perioperative risk with advanced knee osteoarthritis and failure of medical treatment or rehabilitation, within age limit
Interventions	**Intervention** Tisseel® (fibrin sealant, 4 mL, Baxter) **Comparator** IV TXA
Outcomes	Total blood loss after operation Incidence of any thrombotic events Incidence of wound infection after surgery Blood transfusion requirement Calculated blood loss from drainage
Notes	Status on trial registry: trial status ‐ 'unknown' and recruitment status listed as "not yet recruiting". Trial registry page last updated October 2017. No contact name or email provided. Trial status remains "not yet recruiting" as of 29 November 2022. We have searched for a publication but have not found one so far that relates to the study details given in the trial registration.

**Table:** NCT03386656

Methods	Randomised controlled trial
Participants	Patients aged 18 to 80 years, diagnosed with confirmation of severe knee osteoarthritis according to Kellgren criteria (equal or greater than 2) and EVA greater than 7, who will be subjected to knee arthroplasty surgery
Interventions	**Intervention** Tranexamic acid **Comparator** Placebo
Outcomes	Blood loss Time post‐intervention of functional recovery Length of hospital stay
Notes	Status on trial registry "complete". Trial registry page last updated January 2020. No author contact information provided. Trial status remains "complete" as of 29 November 2022. We have searched for a publication but have not found one so far that relates to the study details given in the trial registration.

**Table:** NCT03822793

Methods	Randomised controlled trial
Participants	Patient requiring primary hip arthroplasty (less than 3 months) who have given consent (or consent has been gained from a family member or the support person)
Interventions	**Intervention 1** Perfusion of NaCl (sodium chloride 9%; placebo) IV over 10 minutes just before surgery **Intervention 2** Perfusion of 500 mg TXA IV over 10 minutes just before surgery **Intervention 3** Perfusion of 1000 mg TXA IV over 10 minutes just before surgery **Intervention 4** Perfusion of 1500mg TXA IV over 10 minutes just before surgery **Comparator** Perfusion of 3000 mg TXA IV over 10 minutes just before surgery.
Outcomes	Haemoglobin decrease in the perioperative period Evolution of the concentration of tranexamic acid Evolution of the concentration of D‐dimer Allogenic red blood cell transfusion Severe anaemia Incidence of symptomatic thrombotic events Death Occurrence of a seizure
Notes	Status on trial registry "not yet recruiting". Trial registry page last updated November 2021. Trial status remains "not yet recruiting" as of 5 December 2022.

**Table:** NCT0393407

Methods	Randomised controlled trial
Participants	Adults who have osteoarthritis and need a total knee replacement
Interventions	**Intervention** Visual field infiltration of TXA during the total knee replacement operation **Comparator** No visual field infiltration of TXA during the total knee replacement operation
Outcomes	Blood loss
Notes	Status on trial registry "unknown". Trial registry page last updated May 2019 RC emailed author for update on the current status of the trial but no response. Trial status remains "unknown" as of 29 November 2022. We have searched for a publication but have not found one so far that relates to the study details given in the trial registration.

**Table:** Ni 2016

Methods	Randomised controlled trial
Participants	Patients with osteonecrosis of the femoral head treated by total hip arthroplasty
Interventions	**Intervention** TXA IV **Comparator** Topical TXA
Outcomes	Intraoperative and postoperative bleeding Hb Haematocrit Total blood loss Hidden blood loss Dominant blood loss
Notes	Unable to find a trial registration and therefore cannot determine if the trial was prospectively registered.

**Table:** Notarnicola 2012

Methods	Randomised controlled trial
Participants	Patients affected by gonarthrosis scheduled to undergo a total knee arthroplasty
Interventions	**Intervention 1** Fibrin sealant 5 mL **Intervention 2** Fibrin sealant 10 mL **Comparator** Untreated control
Outcomes	Mean reduction of haemoglobin concentrations Mean number of blood transfusions Functional recovery Joint range of motion Mean blood loss Hospital stay Adverse events
Notes	Unable to find a trial registration and therefore cannot determine if the trial was prospectively registered.

**Table:** Obaidur 2014

Methods	Randomised controlled trial
Participants	Patients below 85 years of age undergoing unilateral or bilateral cemented total knee arthroplasty
Interventions	**Intervention** TXA IV **Comparator** Control
Outcomes	Postoperative blood loss Number of blood units transfused Change in haemoglobin level Adverse events
Notes	Unable to find a trial registration and therefore cannot determine if the trial was prospectively registered.

**Table:** Oztas 2015

Methods	Randomised controlled trial
Participants	Patients with degenerative knee osteoarthritis who did not respond to conservative treatment and underwent unilateral primary total knee replacement
Interventions	**Intervention 1** TXA IV **Intervention 2** Topical TXA **Comparator** No TXA used
Outcomes	Total blood loss and transfusion rate Hb Haematocrit
Notes	Unable to find a trial registration and therefore cannot determine if the trial was prospectively registered.

**Table:** Pan 2019

Methods	Randomised controlled trial
Participants	Patients with knee osteoarthritis who underwent unilateral total knee arthroplasty
Interventions	**Intervention 1** 7.5 mg/kg IV TXA **Intervention 2** 15 mg/kg IV TXA **Comparator** Saline
Outcomes	Intraoperative bleeding volume Postoperative drainage volume Recessive blood loss Total blood loss volume Blood transfusion Activated partial thromboplastin time Prothrombin time Prothrombin international standardised ratio Indexes of D‐dimer DVT PE
Notes	Unable to find a trial registration and therefore cannot determine if the trial was prospectively registered.

**Table:** Patel 2014

Methods	Randomised controlled trial
Participants	Patients undergoing elective unilateral primary total knee arthroplasty for osteoarthritis
Interventions	**Intervention** IV TXA **Comparator** Topical TXA
Outcomes	Perioperative change in haemoglobin level Preoperative haemoglobin level Lowest postoperative haemoglobin level Total drain output Need for transfusion Postoperative complications DVT PE Ecchymosis Wound infections Re‐operation
Notes	Unable to find a trial registration and therefore cannot determine if the trial was prospectively registered.

**Table:** Patni 2012

Methods	Randomised controlled trial
Participants	Patients who were scheduled to undergo a sequential primary bilateral total knee arthroplasty
Interventions	**Intervention** IV TXA **Comparator** Saline
Outcomes	Mean external blood loss Hb Haematocrit Wound complications Thromboembolic events
Notes	Unable to find a trial registration and therefore cannot determine if the trial was prospectively registered.

**Table:** Piolanti 2018

Methods	Randomised controlled trial
Participants	Patients who underwent primary unilateral minimally invasive total hip arthroplasty because of a hip osteoarthritic degeneration
Interventions	**Intervention** Two IV doses of 10 mg/kg of TXA **Comparator** Two IV doses of 20 mg/kg of TXA
Outcomes	Levels of Hb Levels of haematocrit Mean blood volume loss Adverse events Blood transfusion Length of hospital stay
Notes	Unable to find a trial registration and therefore cannot determine if the trial was prospectively registered.

**Table:** Prabhu 2015

Methods	Randomised controlled trial
Participants	Patients undergoing cemented total knee arthroplasty
Interventions	**Intervention** TXA **Comparator** Saline
Outcomes	Packed cell volume Plasminogen Bleeding time Fibrinogen degradation product Platelet count Prothrombin time apTT (activated partial thromboplastin time) D‐dimer concentrations Thromboembolic complications Blood loss Blood transfusion
Notes	Unable to find a trial registration and therefore cannot determine if the trial was prospectively registered.

**Table:** Prakash 2018

Methods	Randomised controlled trial
Participants	Patients with diagnosis of primary osteoarthritis of knee awaiting navigation‐assisted total knee arthroplasty surgery
Interventions	**Intervention 1** IV TXA **Intervention 2** IA TXA **Intervention 3** Combined (IV + IA) TXA **Comparator** Control
Outcomes	Transfusion incidence Drain output Postoperative haemoglobin Haematocrit drop Calculated perioperative blood loss Blood volume Incidence of symptomatic DVT and pulmonary embolism Duration of surgery Wound‐related complications including excessive oozing and skin ecchymosis, and skin blisters Functional scores (hospital for special surgery scores and Western Ontario and McMaster Universities Osteoarthritis Index) Range of motion Time to walk independently and time to flex knee to 90 degrees after surgery
Notes	Unable to find a trial registration and therefore cannot determine if the trial was prospectively registered.

**Table:** Raviraj 2012

Methods	Randomised controlled trial
Participants	Patients who underwent simultaneous bilateral total knee replacement were included in the study
Interventions	**Intervention** IV TXA **Comparator** Control (no TXA)
Outcomes	Hb Number of blood transfusions Blood loss Revision surgery
Notes	Unable to find a trial registration and therefore cannot determine if the trial was prospectively registered.

**Table:** Rizzo 2020

Methods	Randomised controlled trial
Participants	Patients undergoing uncemented primary total hip arthroplasty
Interventions	**Intervention 1** Topical TXA **Intervention 2** IV + topical TXA **Comparator** Controls
Outcomes	Pre‐ and postoperative haemoglobin (Hb) levels and haematocrit (Hct) Rate of blood transfusion
Notes	Unable to find a trial registration and therefore cannot determine if the trial was prospectively registered.

**Table:** Sarzaeem 2014

Methods	Randomised controlled trial
Participants	Patients who underwent unilateral total knee arthroplasty
Interventions	**Intervention 1** 500 mg of TXA in 100 cc of saline was administered intravenously after closing the wound immediately **Intervention 2** Knee joint cavity irrigated with 3 g of TXA in 100 cc of saline just before suturing for 5 min **Intervention 3** Immediately after wound closure, 1.5 g of TXA in 100 cc of saline was injected through the Portovac drain **Comparator** Control group patients did not undergo treatment with TXA
Outcomes	Haemoglobin drop Drainage (postoperative blood loss) Number of transfused units Frequency of transfusion Postoperative complications or events such as infection, skin necrosis, deep vein thrombosis (DVT) and pulmonary embolism
Notes	Unable to find a trial registration and therefore cannot determine if the trial was prospectively registered.

**Table:** Seo 2013

Methods	Randomised controlled trial
Participants	Patients who planned to undergo total knee arthroplasty due to degenerative arthritis in a knee joint
Interventions	**Intervention 1** IV TXA **Intervention 2** IA TXA **Comparator** Placebo
Outcomes	Amount of drainage Difference in haemoglobin levels Frequency of transfusion Number of blood units transfused Any perioperative complications or events such as infection, deep vein thrombosis (DVT) and pulmonary embolism
Notes	Unable to find a trial registration and therefore cannot determine if the trial was prospectively registered.

**Table:** Shen 2015

Methods	Randomised controlled trial
Participants	Patients with primary knee osteoarthritis undergoing unilateral total knee arthroplasty
Interventions	**Intervention** IV TXA **Comparator** Placebo (saline)
Outcomes	Intraoperative blood loss Postoperative drainage Total drainage amount Hidden blood loss Total blood loss Transfusion volumes Number of transfusions Postoperative haemoglobin D‐dimer Number of lower limb ecchymoses DVT
Notes	Unable to find a trial registration and therefore cannot determine if the trial was prospectively registered.

**Table:** Shinde 2015

Methods	Randomised controlled trial
Participants	Patients of Indian origin undergoing total knee arthroplasty for primary osteoarthritis of the knee joint
Interventions	**Intervention 1** IV TXA in patients having a unilateral operation **Intervention 2** Saline in patients having a unilateral operation **Intervention 3** IV TXA in patients having a bilateral operation **Comparator** Saline in patients having a bilateral operation
Outcomes	Volume of drained blood Postoperative blood loss Mean Hb Need for blood transfusion Complications
Notes	Unable to find a trial registration and therefore cannot determine if the trial was prospectively registered.

**Table:** Singh Sidhu 2021

Methods	Randomised controlled trial
Participants	Patients between 55 to 80 years undergoing bilateral total knee replacement
Interventions	**Intervention** IV TXA **Comparator** Topical TXA
Outcomes	Average haemoglobin loss Reduction in blood loss Blood transfusion rate WOMAC score
Notes	Unable to find a trial registration and therefore cannot determine if the trial was prospectively registered.

**Table:** Soni  2014

Methods	Randomised controlled trial
Participants	Patients who were planned to undergo total knee arthroplasty surgery due to degenerative arthritis of the knee joint
Interventions	**Intervention** IV TXA **Comparator** IA TXA
Outcomes	Hb fall Mean drained blood Need for blood transfusion DVT Thromboembolic complications
Notes	Unable to find a trial registration and therefore cannot determine if the trial was prospectively registered.

**Table:** Specchiulli 2011

Methods	Randomised controlled trial
Participants	Patients who underwent total hip and knee replacement
Interventions	**Intervention 1** IV TXA for patients who underwent total hip replacement **Intervention 2** Control (no pharmacological intervention) for patients who underwent total hip replacement **Intervention 3** IV TXA for patients who underwent total knee replacement **Comparator** Control (no pharmacological intervention) for patients who underwent total knee replacement
Outcomes	Total blood loss Number of units of blood transfused Complications, such as venous or pulmonary thrombosis
Notes	Unable to find a trial registration and therefore cannot determine if the trial was prospectively registered.

**Table:** Sun 2016

Methods	Randomised controlled trial
Participants	Patients undergoing total hip arthroplasty
Interventions	**Intervention** Combined (IV + IA TXA) **Comparator** IV TXA
Outcomes	Postoperative drainage Number of blood transfusions Postoperative blood loss Hb and haematocrit Postoperative coagulation function Thromboembolic events Septum thrombosis Deep thromboembolic events
Notes	Unable to find a trial registration and therefore cannot determine if the trial was prospectively registered.

**Table:** Sun 2017

Methods	Randomised controlled trial
Participants	Patients who were scheduled to undergo primary unilateral total knee arthroplasty
Interventions	**Intervention 1** IV TXA (30 mg/kg) **Intervention 2** IV TXA (15 mg/kg) **Intervention 3** IV TXA (10 mg/kg) **Comparator** Control (TXA not infused)
Outcomes	Blood loss (intraoperative, postoperative and total blood loss) Blood transfusion rate and volume Haemoglobin level Incidence of deep vein thrombosis
Notes	Unable to find a trial registration and therefore cannot determine if the trial was prospectively registered.

**Table:** Taheriazam 2018

Methods	Randomised controlled trial
Participants	Patients undergoing revision THA
Interventions	**Intervention** IV TXA **Comparator** Local TXA + diluted epinephrine
Outcomes	Pre‐ and postoperative level of haemoglobin (Hb) Number of transfused packed red blood cells Total blood loss Hidden blood loss Transfusion rate Risks of complications such as thromboembolic events.
Notes	Unable to find a trial registration and therefore cannot determine if the trial was prospectively registered.

**Table:** Taheriazam 2019

Methods	Randomised controlled trial
Participants	Patients undergoing revision total hip arthroplasty
Interventions	**Intervention** IV TXA **Comparator** Local TXA + diluted epinephrine
Outcomes	Level of Hb Rate of Hb drop Blood transfused Reduced total blood loss Hidden blood loss Transfusion rate Risks of haemodynamic complexity
Notes	Unable to find a trial registration and therefore cannot determine if the trial was prospectively registered.

**Table:** Tang 2019

Methods	Randomised controlled trial
Participants	Patients underwent primary unilateral total knee arthroplasty for osteoarthritis or rheumatoid arthritis
Interventions	**Intervention 1** 2 g of oral TXA 2 hrs preoperatively **Intervention 2** An additional dose of 2 g of oral TXA 4 hrs postoperatively **Comparator** Additional doses of 2 g of oral TXA at 4, 10 and 16 hrs postoperatively
Outcomes	Total blood loss Maximum drop in haemoglobin (Hb) and haematocrit (Hct) Level of inflammatory and fibrinolytic parameters Transfusion rate The incidence of complications
Notes	Unable to find a trial registration and therefore cannot determine if the trial was prospectively registered.

**Table:** Triyudanto 2016

Methods	Randomised controlled trial
Participants	Patients with severe osteoarthritis end‐stage of grade III and grade IV Kelgren Lawrence criteria who underwent total knee arthroplasty
Interventions	**Intervention 1** IA TXA ‐ intraoperative **Intervention 2** IV TXA ‐ preoperative **Comparator** Control group
Outcomes	Intraoperative bleeding Haemoglobin (Hb) level Total drain production Total blood transfusion needed Drain removal timing
Notes	Unable to find a trial registration and therefore cannot determine if the trial was prospectively registered.

**Table:** Tzatzairis 2019

Methods	Randomised controlled trial
Participants	Patients undergoing total knee arthroplasty for knee osteoarthritis
Interventions	**Intervention 1** Group A received 15 mg/kg of IV TXA given on induction **Intervention 2** Group B received an additional dose of IV TXA (15 mg/kg) 3 hrs after incision **Comparator** Group C received an additional (third) dose 3 hrs later (15 mg/kg)
Outcomes	Change in haemoglobin (Hb) Amount of blood transfusion given Functional and quality of life (QoL) Pain assessment
Notes	Unable to find a trial registration and therefore cannot determine if the trial was prospectively registered.

**Table:** Ugurlu 2017

Methods	Randomised controlled trial
Participants	Patients undergoing primary total knee arthroplasty for degenerative osteoarthritis
Interventions	**Intervention 1** IV TXA **Intervention 2** Topical TXA **Comparator** Control
Outcomes	Hb Length of hospital stay Drain output volumes Complications Preoperative co‐morbidities Number of transfusions
Notes	Unable to find a trial registration and therefore cannot determine if the trial was prospectively registered.

**Table:** UMIN000029797

Methods	Randomised controlled trial
Participants	Patients undergoing simultaneous bilateral total hip arthroplasty
Interventions	**Intervention** Intravenous + topical tranexamic acid **Comparator** Intravenous tranexamic acid
Outcomes	Perioperative blood loss Complications
Notes	Status on trial registry "recruitment status is completed, but results are still unpublished". Trial registry page last updated March 2019. RC emailed author for update on the current status of the trial on 13 July 2021 (and on 3 occasions thereafter), but no response. Trial status remains "recruitment status is completed, but results are still unpublished" as of 29 November 2022. We have searched for a publication but have not found one so far that relates to the study details given in the trial registration.

**Table:** Vandesande 2015

Methods	Randomised controlled trial
Participants	Patients undergoing elective total hip arthroplasty
Interventions	**Intervention** Topical TXA **Comparator** Control
Outcomes	Haemoglobin level (Hb, g/dL) Haematocrit value (Hct, %) Platelet count INR Partial thromboplastin time Postoperative blood loss Transfusion rate
Notes	Unable to find a trial registration and therefore cannot determine if the trial was prospectively registered.

**Table:** Volquind 2016

Methods	Randomised controlled trial
Participants	Patients undergoing primary total knee replacement due to osteoarthrosis or rheumatoid arthritis
Interventions	**Intervention** IV TXA **Comparator** Saline
Outcomes	Haemoglobin Haematocrit Blood loss DVT Number of blood transfusions Thromboembolic events Postoperative bleeding
Notes	Unable to find a trial registration and therefore cannot determine if the trial was prospectively registered.

**Table:** Wang 2015

Methods	Randomised controlled trial
Participants	Patients who received bilateral total knee arthroplasty
Interventions	**Intervention** IV TXA **Comparator** Control (no TXA)
Outcomes	Total blood loss Intraoperative blood loss Hidden blood loss Amount of postoperative drainage Ratio of blood transfusion Haemoglobin D‐dimer Prothrombin time Activated partial thromboplastin time
Notes	Unable to find a trial registration and therefore cannot determine if the trial was prospectively registered.

**Table:** Wang 2015a

Methods	Randomised controlled trial
Participants	Hospital‐admitted patients who underwent primary unilateral total knee arthroplasty
Interventions	**Intervention** IA TXA **Comparator** Control
Outcomes	Postoperative haemoglobin Blood coagulation index Total blood loss volume Drainage volume Blood transfusion rate Lower extremity DVT rate
Notes	Unable to find a trial registration and therefore cannot determine if the trial was prospectively registered.

**Table:** Wang 2015b

Methods	Randomised controlled trial
Participants	Patients treated with unilateral primary cement total knee arthroplasty
Interventions	**Intervention** IA TXA **Comparator** Saline
Outcomes	Mean blood loss Maximum Hb drop Reduction in red blood cell Haematocrit Transfusion rate Average amount transfused Coagulation markers D‐dimer Rate of symptomatic DVT, PE or wound healing problems
Notes	Unable to find a trial registration and therefore cannot determine if the trial was prospectively registered.

**Table:** Wang 2016

Methods	Randomised controlled trial
Participants	Patients diagnosed with osteoarthritis and scheduled to undergo primary unilateral total hip arthroplasty
Interventions	**Intervention 1** 10 mg/kg IV TXA **Intervention 2** 15 mg/kg IV TXA **Comparator** Control (placebo)
Outcomes	Blood transfusions DVT PE Blood loss Decrease in Hb and haematocrit Other complications
Notes	Unable to find a trial registration and therefore cannot determine if the trial was prospectively registered.

**Table:** Wang 2017

Methods	Randomised controlled trial
Participants	Patients who were scheduled for a primary unilateral total knee arthroplasty for end‐stage osteoarthritis
Interventions	**Intervention 1** IV TXA **Intervention 2** IA TXA **Comparator** Control (saline)
Outcomes	Amount of total and hidden blood loss Drainage Transfusion Changes in haemoglobin levels Complications
Notes	Unable to find a trial registration and therefore cannot determine if the trial was prospectively registered.

**Table:** Wei 2014

Methods	Randomised controlled trial
Participants	Patients undergoing total hip arthroplasty
Interventions	**Intervention 1** IV TXA **Intervention 2** IA TXA **Comparator** No TXA
Outcomes	Hct Maximum Hct drop Length of hospital stay Transfusion rates Wound complications Total blood loss DVT PE
Notes	Unable to find a trial registration and therefore cannot determine if the trial was prospectively registered.

**Table:** Wu 2016a

Methods	Randomised controlled trial
Participants	Patients undergoing revision total hip arthroplasty
Interventions	**Intervention** IV TXA + topical TXA **Comparator** IV TXA
Outcomes	Mean total blood loss Drainage volume Maximum Hb drop Hct drop Amount of blood transfusions Number of blood transfusions required DVT PE Calf muscular vein thrombosis (CMVT) Blood transfusion rate
Notes	Unable to find a trial registration and therefore cannot determine if the trial was prospectively registered.

**Table:** Wu 2018a

Methods	Randomised controlled trial
Participants	Patients suffering from unilateral osteoarthritis who underwent primary total knee arthroplasty
Interventions	**Intervention 1** IV TXA + 1 g IA TXA **Intervention 2** IV TXA + 2 g IA TXA **Comparator** IV TXA + 3 g IA TXA
Outcomes	Preoperative haemoglobin (Hb) concentration Platelet count Preoperative prothrombin time Activated partial thromboplastin time Postoperative wound blood drainage Transfusion rate Thromboembolic complications DVT PE
Notes	Unable to find a trial registration and therefore cannot determine if the trial was prospectively registered.

**Table:** Wu 2019

Methods	Randomised controlled trial
Participants	Patients who underwent unilateral total knee arthroplasty due to knee osteoarthritis
Interventions	**Intervention 1** IV TXA **Intervention 2** IA TXA **Comparator** IV TXA + IA TXA
Outcomes	Perioperative total blood loss Occult blood loss Number of patients receiving blood transfusion Volume of blood transfused
Notes	Unable to find a trial registration and therefore cannot determine if the trial was prospectively registered.

**Table:** Wu 2019a

Methods	Randomised controlled trial
Participants	Patients with osteoarthritis of the knee who were scheduled to undergo elective unicompartmental knee arthroplasty surgery
Interventions	**Intervention** IA TXA **Comparator** Saline
Outcomes	Blood volumes Total blood loss Postoperative drainage Hidden blood loss Blood transfusion rates Postoperative haemoglobin values Indicators of coagulation function Rates of wound complications
Notes	Unable to find a trial registration and therefore cannot determine if the trial was prospectively registered.

**Table:** Wu 2020

Methods	Controlled trial but unclear if patients were randomised
Participants	Patients undergoing minimally invasive unicompartmental knee arthroplasty
Interventions	TXA IA (dose not reported in abstract)
Outcomes	Wound healing Incidence of DVT Incidence of PE Postoperative blood loss Need for transfusion
Notes	Unable to find a trial registration and therefore cannot determine if the trial was prospectively registered. Unable to contact author to confirm study design.

**Table:** Wu 2020a

Methods	Randomised controlled trial
Participants	Patients with unilateral degenerative osteoarthritis who underwent primary total knee arthroplasty
Interventions	**Intervention 1** IV TXA + 0 g IA TXA **Intervention 2** IV TXA + 1 g IA TXA **Intervention 3** IV TXA + 2 g IA TXA **Comparator** IV TXA + 4 g IA TXA
Outcomes	Wound blood drainage Hb concentration Blood transfusion Wound complications DVT PE Postoperative blood drainage
Notes	Unable to find a trial registration and therefore cannot determine if the trial was prospectively registered.

**Table:** Xiaofei 2020

Methods	Randomised controlled trial
Participants	Patients with knee osteoarthritis undergoing primary knee arthroplasty
Interventions	1 g TXA IV intraoperatively followed by 1 g TXA IV at 3 hours postoperative vs placebo
Outcomes	Duration of surgery Intraoperative blood loss Drainage volume at 24 hours IL‐6 at 24 and 72 hours TNF‐alpha at 24 and 72 hours
Notes	Unable to find a trial registration and therefore cannot determine if the trial was prospectively registered.

**Table:** Xie 2016a

Methods	Randomised controlled trial
Participants	Patients with osteoarthritis or osteonecrosis of the femoral head undergoing unilateral total hip arthroplasty
Interventions	1.5 g TXA IV preoperatively vs 3 g TXA IA intraoperatively vs 1 g TXA IV plus 2 g TXA IA
Outcomes	Total blood loss Overt blood loss Hidden blood loss Maximum haemoglobin drop Transfusion rate Transfusion index Duration of surgery Duration of hospital stay
Notes	Unable to find a trial registration and therefore cannot determine if the trial was prospectively registered.

**Table:** Xu 2016

Methods	Randomised controlled trial
Participants	Patients with osteoarthritis undergoing unilateral primary total knee arthroplasty
Interventions	Control vs 2 g TXA IA intraoperatively vs TXA 1 g IV intraoperatively vs TXA 1 g IV and 2 g TXA IA, both intraoperatively
Outcomes	Blood loss Haemoglobin Need for transfusion Incidence of complications: DVT PE Wound trouble, e.g. necrosis, infection, haematoma
Notes	Unable to find a trial registration and therefore cannot determine if the trial was prospectively registered.

**Table:** Xu 2019b

Methods	Randomised controlled trial
Participants	Patients with osteoarthritis undergoing unilateral primary cementless total hip arthroplasty
Interventions	2 g TXA IA vs 20 mg/kg TXA IV vs placebo
Outcomes	Haematocrit Fibrinogen Prothrombin time Activated partial thromboplastin time Incidence of DVT Incidence of PE
Notes	Unable to find a trial registration and therefore cannot determine if the trial was prospectively registered.

**Table:** Yang 2015

Methods	Randomised controlled trial
Participants	Patients over 60 years of age diagnosed with osteoarthritis, traumatic arthritis or rheumatoid arthritis undergoing primary unilateral total knee arthroplasty
Interventions	500 mg TXA IA intraoperatively vs placebo
Outcomes	Haematocrit Haemoglobin D‐dimer Fibrinogen Prothrombin time Activated partial thromboplastin clotting time Amount of blood transfused
Notes	Unable to find a trial registration and therefore cannot determine if the trial was prospectively registered.

**Table:** Ye 2019

Methods	Randomised controlled trial
Participants	Patients with rheumatoid arthritis or advanced osteoarthritis undergoing total knee arthroplasty
Interventions	Placebo vs 10 mg/kg TXA IV intraoperatively vs 15 mg/kg TXA IV intraoperatively vs 20 mg/kg TXA IV intraoperatively vs 30 mg/kg TXA IV intraoperatively
Outcomes	TEG parameters postoperatively reaction time coagulation time maximum amplitude Coagulation parameters: Prothrombin time Activated partial thromboplastin time Fibrinogen D‐dimer and thrombin time Intraoperative blood loss Need for transfusion Incidence of DVT
Notes	Unable to find a trial registration and therefore cannot determine if the trial was prospectively registered.

**Table:** Yue 2014

Methods	Randomised controlled trial
Participants	Patients with osteoarthritis or osteonecrosis of the femoral head undergoing primary unilateral total hip arthroplasty
Interventions	3 g TXA IA x 3 intraoperatively vs placebo
Outcomes	Need for transfusion Incidence of DVT Incidence of PE Total blood loss Haemoglobin day 1 and 3 Haematocrit day 1 and 3 Incidence of infection Incidence of haematoma Incidence of wound secretion Duration of hospitalisation
Notes	Unable to find a trial registration and therefore cannot determine if the trial was prospectively registered.

**Table:** Zeng 2016

Methods	Randomised controlled trial
Participants	Patients with knee osteoarthritis undergoing unicompartmental knee arthroplasty at the Second Affiliated Hospital of Dalian Medical University between January 2014 and August 2015
Interventions	1 g TXA IA intraoperatively vs placebo
Outcomes	Haemoglobin 2 days and 1 month postoperatively Haematocrit 2 days and 1 month postoperatively Drainage volume Total blood loss Need for transfusion HSS score Incidence of DVT
Notes	Unable to find a trial registration and therefore cannot determine if the trial was prospectively registered.

**Table:** Zhang 2015

Methods	Randomised controlled trial
Participants	Patients undergoing primary total hip arthroplasty
Interventions	0.5 g TXA IV plus 0.5 g TXA IA intraoperatively vs placebo
Outcomes	Haemoglobin Haematocrit Total blood loss Hidden blood loss Need for transfusion Duration of hospital stay Pain score Incidence of DVT
Notes	Unable to find a trial registration and therefore cannot determine if the trial was prospectively registered.

**Table:** Zhang 2016

Methods	Randomised controlled trial
Participants	Patients with osteonecrosis of the femoral head undergoing total hip arthroplasty
Interventions	1 g TXA IV preoperatively vs 1 g TXA IA intraoperatively
Outcomes	Adverse events Intraoperative blood loss Postoperative drainage volume Total blood loss of red cells Recessive loss of red blood cells Calculated total blood loss during surgery Need for transfusion Clinical symptoms (swelling, pain, chest tightness, shortness of breath) postoperatively Postoperative complications
Notes	Unable to find a trial registration and therefore cannot determine if the trial was prospectively registered.

**Table:** Zhang 2018

Methods	Randomised controlled trial
Participants	Patients undergoing unicompartmental knee arthroplasty
Interventions	1.5 g TXA IA vs placebo
Outcomes	Total blood loss Total drainage volume Hidden blood loss Adverse events
Notes	Unable to find a trial registration and therefore cannot determine if the trial was prospectively registered.

**Table:** Zhang 2019

Methods	Randomised controlled trial
Participants	Patients 40 to 80 years undergoing total knee arthroplasty at Weifang People's Hospital between January 2015 and December 2016
Interventions	20 mg/kg TXA IV before the incision and 3 g TXA IA vs 20 mg/kg TXA IV alone
Outcomes	HSS score KSS score NASS score Range of motion Total blood loss Hidden blood loss Maximum haemoglobin drop FDP D‐dimer
Notes	Unable to find a trial registration and therefore cannot determine if the trial was prospectively registered.

**Table:** Zhang 2019a

Methods	Randomised controlled trial
Participants	Patients with knee osteoarthritis requiring unilateral TKA in Zaozhuang Municipal Hospital between October 2016 and September 2018
Interventions	1 g TXA IA vs 1 g periarticular TXA vs 1 g TXA IA plus 1 g periarticular TXA vs placebo All patients received 20 mg/kg TXA prior to tourniquet compression
Outcomes	Duration of operation Intraoperative blood loss Haemoglobin day 1 Haematocrit day 1 Transfusion requirements Drainage volume Total blood loss Hidden blood loss
Notes	Unable to find a trial registration and therefore cannot determine if the trial was prospectively registered.

**Table:** Zhang 2020

Methods	Randomised controlled trial
Participants	Patients with avascular necrosis of the femoral head undergoing total hip arthroplasty via direct anterior approach
Interventions	TXA vs saline
Outcomes	Blood loss Haemoglobin value Number of blood transfusion cases Time of first landing after operation Incidence of thrombosis and incision adverse events
Notes	Unable to find a trial registration and therefore cannot determine if the trial was prospectively registered. Cannot access full text. Request via British Library failed to find the paper. Cannot confirm whether it is an RCT and whether the study was prospectively registered.

**Table:** Zhang 2020a

Methods	Randomised controlled trial
Participants	Patients undergoing total hip arthroplasty in Beijing Chao‐Yang Hospital between March 2018 and March 2019
Interventions	2.0 g TXA in 10 mL normal saline IA vs 2 g TXA in 100 mL normal saline IV
Outcomes	Apparent blood loss Hidden blood loss Average volume of blood transfusion Incidence of DVT and PE
Notes	Unable to find a trial registration and therefore cannot determine if the trial was prospectively registered.

**Table:** Zhang 2020b

Methods	Randomised controlled trial
Participants	Patients with osteoarthritis or osteonecrosis of the femoral head undergoing primary unilateral cementless total hip arthroplasty
Interventions	IV TXA at 15 mg/kg of bodyweight 30 min before skin incision
Outcomes	Transfusion rate Volume of blood transfused Visible blood loss Hidden blood loss Drainage blood loss Calculated blood loss Total blood loss TEG variables (day 7) Conventional lab values (day 7)
Notes	Unable to find a trial registration and therefore cannot determine if the trial was prospectively registered.

**Table:** Zhaohui 2014

Methods	Randomised controlled trial
Participants	Patients with osteoarthritis of the knee undergoing bilateral total knee arthroplasty
Interventions	5 ml (25 mg/mL) tranexamic acid (TXA) and 5 mL analgesic containing epinephrine (3 μg/mL) topically
Outcomes	Intraoperative blood loss Total postoperative blood loss Need for allogeneic transfusion
Notes	Unable to find a trial registration and therefore cannot determine if the trial was prospectively registered.

aPTT: activated partial thromboplastin time; ASA: American Society of Anesthesiologists; BMI: body mass index; CRP: C‐reactive protein; DVT: deep vein thrombosis; EACA: epsilon aminocaproic acid; ESR: erythrocyte sedimentation rate; EVA: enlarged vestibular aqueduct; FDP: fibrin degradation products; Hb: haemoglobin; hr/hrs: hour/s; HSS: Hospital for Special Surgery score; IA: intra‐articular; IL6: interleukin 6; INR: international normalised ratio; IV: intravenous; KSS: Knee Society Score; NASS: North American Spinal Association score; NS: normal saline; PE: pulmonary embolism; PI: perfusion index; po: oral; POD: postoperative day; PRBC: packed red blood cells; SUSAR: suspected unexpected serious adverse reaction; PTE: pulmonary thromboembolism; TE: thromboembolism; TEG: thromboelastography; TNF: tumour necrosis factor; TXA: tranexamic acid; VAS: visual analogue scale; WOMAC: Western Ontario and McMaster Universities Osteoarthritis Index

### Characteristics of ongoing studies [ordered by study ID]

**Table:** ChiCTR‐INR‐16008762

Study name	'Is tourniquet really necessary when multiple uses of intravenous and topical tranexamic acid are applied in primary total knee arthroplasty? A prospective randomized controlled trial'
Methods	Randomised controlled trial China
Participants	**Inclusion criteria** Aged 18 years and older Scheduled for a primary total knee arthroplasty because of end‐stage osteoarthritis **Exclusion criteria** Revisions, bilateral procedures Previous knee surgery history Flexion deformity = 30 degree, varus/valgus deformity = 30 degree Anaemia (< 120 g/L for female, < 130 g/L for male) Contraindications for the use of TXA Coagulation disorders **Planned recruitment** 150 participants (1:1:1)
Interventions	**Intervention 1** Tourniquet + 20 mg/kg IV TXA was administered 5 to 10 minutes before skin incision and 10 mg/kg TXA was administered 3, 6, 12 and 24 hours later **Intervention 2** 20 mg/kg IV TXA was administered 5 to 10 minutes before skin incision and 10 mg/kg TXA was administered 3, 6, 12 and 24 hours later **Comparator** Only use a tourniquet during the surgery
Outcomes	Hidden blood loss, maximum Hb change, CRP, IL‐6, lower limb swelling ratio, VAS pain score, the length of hospital stays, transfusion rate, patient satisfaction, complications
Starting date	July 2016
Contact information	Zeyu Huang zey.huang@gmail.com
Notes	RC emailed author from 14 July 2021 and on 3 separate occasions, but no response. Trial page last accessed 29 November 2022 and trial status remains as 'recruiting' but this has not been updated since 2016.

**Table:** ChiCTR‐INR‐16010030

Study name	'Optimized cocktail therapy during operation in ERAS total hip and knee arthroplasty: a prospective randomized controlled trial'
Methods	Randomised controlled trial China
Participants	**Inclusion criteria** Aged 18 years or older Scheduled to have primary unilateral total hip arthroplasty/total knee arthroplasty for osteoarthritis or ANFH or DDH **Exclusion criteria** History of deep vein thrombosis (VTE, calf muscle vein thrombosis or superficial vein thrombosis, pulmonary embolism) Cardiovascular or cerebrovascular diseases (history of myocardial infraction, angina, atrial fibrillation or previous stroke) Clotting disorders Discontinuation of oral anticoagulants or aspirin less than 1 week Allergy to tranexamic acid (TXA) or ropivacaine or have contraindications Serious liver and kidney dysfunction Patients at high risk of thrombosis **Planned recruitment** 200 participants (1:1)
Interventions	A combined local injection of ropivacaine (200 mg) and TXA (80 mL) before closure vs local injection of ropivacaine (200 mg) and saline (80 mL) before closure
Outcomes	Hidden blood loss, inflammatory index (CRP IL‐6), fibrinolysis index (D‐dimer, FDP), VAS pain score, opioid consumption, Hb drop, total blood loss, transfusion, swelling ratio, range of motion, postoperative hospital stay
Starting date	November 2016
Contact information	Shaoyun Zhang 15102839590@163.com
Notes	RC emailed author from 29 November 2022 on 3 separate occasions, but no response. Trial page last accessed 29 November 2022 when trial status said "ongoing", but this had not been updated since 2016.

**Table:** ChiCTR‐INR‐17013711

Study name	'The clinical effect of multiple high‐dose intravenous tranexamic acid in peri‐operative period of total hip/knee arthroplasty under the management of enhanced recovery after surgery: a prospective, randomized, controlled study'
Methods	Randomised controlled trial China
Participants	**Inclusion criteria** Aged 18 years or older Scheduled to have primary unilateral total hip arthroplasty/total knee arthroplasty for osteoarthritis (OA) or ANFH **Exclusion criteria** Anaemic (< 120 g/L for female, < 130 g/L for male) Cardiovascular problems (history of myocardial infraction, angina and atrial fibrillation) Cerebrovascular conditions (history of previous stroke) Thromboembolic disorders (history of deep vein thrombosis (DVT) or pulmonary embolism (PE) Clotting disorders, known allergy to TXA **Planned recruitment** 100 participants (1:1)
Interventions	**Intervention** Tranexamic acid 20 mg/kg intravenous infusion 5 to 10 min before skin incision, 1 g tranexamic acid intravenous infusion 3, 6, 12, 18, 24 h after the first dose of tranexamic acid **Comparator** Tranexamic acid 20 mg/kg intravenously 5 to 10 min before skin incision, intraoperative tranexamic acid 20 mg/kg slow intravenous infusion, the first dose of tranexamic acid 3, 6, 12, 18, 24 h after use tranexamic acid 20 mg/kg intravenously
Outcomes	Inflammation indicators, fibrinolysis index, total blood loss, hidden blood loss, reduced haemoglobin, blood transfusion rate, joint range of motion, length of hospital stay, swelling ratio, incidence of thrombosis (DVT PE), wound complications
Starting date	December 2017
Contact information	Fuxing Pei peifux@126.com
Notes	RC emailed author for an update from 23 February 2022 on 3 separate occasions, but no response. Trial page last accessed 29 November 2022 and trial status remains as "recruiting", but this has not been updated since 2017.

**Table:** ChiCTR‐IPC‐14005579

Study name	'Intravenous versus intravenous combined with topical administration of tranexamic acid in primary total knee arthroplasty: a randomized controlled trial'
Methods	Randomised controlled trial China
Participants	**Inclusion criteria** 18 years and older Undergoing elective unilateral primary total knee arthroplasty for osteoarthritis **Exclusion criteria** Undergoing TKA for secondary osteoarthritis (rheumatoid arthritis, post‐traumatic arthritis, gouty arthritis) Simultaneous bilateral TKA Cardiovascular problems (history of myocardial infarction, atrial fibrillation, angina, heart failure ‐ class III or IV) Cerebrovascular conditions (history of previous stroke or peripheral vascular surgery) Clotting disorders or blood dyscrasia Thromboembolic disorders (history of DVT or PE) Preoperative haemoglobin more than 150 g/L Known allergy to TXA Pregnancy **Planned recruitment** 120 participants (1:1)
Interventions	**Intervention** Intravenous + topical tranexamic acid **Comparator** Intravenous tranexamic acid
Outcomes	Drainage amount, invisible blood loss, haemoglobin level, haematocrit, deep vein thrombosis, pulmonary embolism, clinical function evaluation, complication
Starting date	January 2015
Contact information	Yong Jiang Jiangyong2007@126.com
Notes	RC emailed author from 13 July 2021 on 3 separate occasions, but no response. Trial page last accessed 25 September 2022 and trial status remains as 'recruiting'; no results posted but the record has not been updated since 2016.

**Table:** ChiCTR‐IPR‐16008175

Study name	'The cocktail therapy control the blood loss and pain after total hip arthroplasty: a randomized controlled trial'
Methods	Randomised controlled trial China
Participants	**Inclusion criteria** aged 18 to 70 years old Having primary unilateral uncemented total hip arthroplasty for severe osteonecrosis of the femoral head **Exclusion criteria** More than 70 years old Made habitual use of opioids Had inflammatory joint disease Previous fracture of the affected hip Haemostatic drugs and pain relievers contraindicated Revision surgery, bilateral joint arthroplasty Known hypersensitivity to TXA or its ingredients Active intravascular clotting disorders Acute subarachnoid haemorrhage **Planned recruitment** 100 participants (1:1)
Interventions	**Intervention** Intravenous plus topical haemostatic drugs and topical pain relievers **Comparator** Topical haemostatic drugs only
Outcomes	Blood loss, pain score (VAS), Harris score, haemoglobin change
Starting date	April 2016
Contact information	Fuqiang Gao gaofuqiang@bjmu.edu.cn
Notes	RC emailed author 5 July 2021 and on 3 separate occasions, but no response. Trial page last accessed 29 November 2022 and trial status remains as 'recruiting'; no results posted but the record has not been updated since March 2016.

**Table:** ChiCTR‐IPR‐16008176

Study name	'The cocktail therapy control the blood loss and pain after total knee arthroplasty: a randomized controlled trial'
Methods	Randomised controlled trial
Participants	**Inclusion criteria** Aged 18 to 70 years old Requiring total knee arthroplasty due to severe osteoarthritis **Exclusion criteria** More than 70 years old Made habitual use of opioids Had inflammatory joint disease Previous fracture of the affected hip Haemostatic drugs and pain relievers contraindicated Revision surgery, bilateral joint arthroplasty Known hypersensitivity to TXA or its ingredients Active intravascular clotting disorders Acute subarachnoid haemorrhage **Planned recruitment** 100 participants (1:1)
Interventions	**Intervention** Intravenous + local infusion of haemostatic and analgesic drugs and local infiltration analgesics **Comparator** Local infusion of haemostatic drugs and local infiltration analgesics
Outcomes	Blood loss, pain score (VAS), HSS score, haemoglobin drop
Starting date	April 2016
Contact information	Fuqiang Gao gaofuqiang@bjmu.edu.cn
Notes	RC emailed author 5 July 2021 and on 3 separate occasions, but no response. Trial page last accessed 29 November 2022 and trial status remains as 'recruiting'; no results posted but the record has not been updated since March 2016.

**Table:** ChiCTR1800016960

Study name	'Multiple boluses of oral tranexamic acid to reduce hidden blood loss after primary medial unicompartmental knee arthroplasty without tourniquet: a randomized clinical trial'
Methods	Randomised controlled trial China
Participants	**Inclusion criteria** Adults (18 to 85 years) with primary medial unicompartmental knee arthroplasty **Exclusion criteria** Acute or chronic infection, a history of deep vein thrombosis (DVT) or pulmonary embolism (PE), haematologic diseases, cancer and a preoperative haemoglobin level below 100 g/L **Planned recruitment** 90 participants (1:1)
Interventions	**Intervention** Oral tranexamic acid 2 g + 1 g **Comparator** Oral tranexamic acid 2 g
Outcomes	Blood loss, range of motion, inflammation
Starting date	5 July 2018
Contact information	Yuangang Wu wuyuangang23@163.com
Notes	RC emailed author from 14 July 2021 and on 3 separate occasions, but no response. Trial page re‐accessed 25 November 2022 and trial status remains as 'recruiting', but this has not been updated since July 2018.

**Table:** ChiCTR1800017038

Study name	'Multiple boluses of oral tranexamic acid to reduce hidden blood loss after primary knee arthroplasty: a randomized clinical trial'
Methods	Randomised controlled trial China
Participants	**Inclusion criteria** Adults (18 to 85 years) with total knee arthroplasty **Exclusion criteria** Aged < 18 years and > 85 years, rheumatoid arthritis, allergy to TXA, a history of thrombosis, coagulation dysfunction, uncontrolled hypertension, patients with infection and body mass index (BMI) > 35 **Planned recruitment** 100 participants (1:1)
Interventions	**Intervention** Two hours before skin incision, 2 g of TXA, and 1 g at 3 h, 6 h and 9 h after operation **Comparator** Two hours before skin incision, 2 g of TXA and 1 g at 3 h, 6 h and 9 h after operation
Outcomes	Blood loss Range of motion Inflammatory index
Starting date	9 July 2018
Contact information	Yuangang Wu wuyuangang23@163.com
Notes	"Recruiting" RC emailed author for update 23 February 2022 to understand the status of this trial and how this trial, and trials ChiCTR1800017094, ChiCTR1800017095 and ChiCTR1800018100, relate to each other. No reply received. RC accessed trial page 29 November 2022 and trial information last updated July 2018 when status was 'recruiting'.

**Table:** ChiCTR1800017094

Study name	'Multiple boluses of oral and topical tranexamic acid to reduce hidden blood loss after primary hip arthroplasty: a randomized clinical trial'
Methods	Randomised controlled trial China
Participants	**Inclusion criteria** Adults (18 to 85 years) with osteoarthritis (OA) or osteonecrosis of the femoral head (ONFH) undergoing unilateral total hip arthroplasty **Exclusion criteria** Developmental dysplasia of the hip, rheumatoid arthritis, revision surgery, a history of thrombosis, patients with infection, allergy to TXA, uncontrolled hypertension, body mass index (BMI) > 35 kg/m^2^ and preoperative anaemia **Planned recruitment** 90 participants (1:1)
Interventions	**Intervention** 2 hours before skin incision and 1 g at 3 hours after operation **Comparator** Local TXA 3 g
Outcomes	Blood loss Range of motion Cost
Starting date	July 2018
Contact information	Yuangang Wu wuyuangang23@163.com
Notes	"Recruiting" RC emailed author for update 23 February 2022 to understand the status of this trial and how this trial, and trials ChiCTR1800017038, ChiCTR1800017095 and ChiCTR1800018100, relate to each other. RC accessed trial page 29 November 2022 and trial information last updated July 2018 when status was 'recruiting'.

**Table:** ChiCTR1800017095

Study name	ChiCTR1800017095
Methods	Randomised controlled trial China
Participants	**Inclusion criteria** Adults (18 to 85 years) with osteoarthritis (OA) or osteonecrosis of the femoral head (ONFH) undergoing unilateral THA **Exclusion criteria** Developmental dysplasia of the hip, rheumatoid arthritis, revision surgery, a history of thrombosis, patients with infection, allergy to TXA, uncontrolled hypertension, body mass index (BMI) > 35 kg/m^2^ and preoperative anaemia **Planned recruitment** 90 participants (1:1)
Interventions	**Intervention** 2 g TXA was orally taken 2 h before operation, and repeated 1 g at 3 h, 6 h and 9 h after operation **Comparator** 2 g TXA was given IV 2 h before operation, and repeated 1 g at 3 h, 6 h and 9 h after operation
Outcomes	Blood loss Range of motion Cost Inflammatory response
Starting date	July 2018
Contact information	Yuangang Wu wuyuangang23@163.com
Notes	"Recruiting" RC emailed author for update 23 February 2022 to understand the status of this trial and how this trial, and trials ChiCTR1800017038, ChiCTR1800017094 and ChiCTR1800018100, relate to each other. No reply received. RC accessed trial page 29 November 2022 and trial information last updated July 2018 when status was 'recruiting'.

**Table:** ChiCTR1800018100

Study name	'Multiple boluses of oral tranexamic Acid (TXA) to reduce hidden blood loss after primary total hip arthroplasty using a direct anterior approach: a randomized clinical trial'
Methods	Randomised controlled trial China
Participants	**Inclusion criteria** Adults with primary unilateral anterior total hip arthroplasty due to end‐stage osteoarthritis, femoral head necrosis (Ficat stage III‐IV) and type I‐II hip dysplasia **Exclusion criteria** Hip joint stiffness, history of previous hip surgery, anaemia (female < 120 g/L, male < 130 g/L), history of cardiovascular and cerebrovascular disease (myocardial ischaemia, atrial fibrillation, cerebral infarction, etc.), contraindications for TXA (6 months of thrombotic events), coagulopathy, allergic or intolerable drugs used in the experiment and abnormal liver function, renal dysfunction, language disorders, mental illness, dementia, age less than 18 or > 80 years old, American Society of Anesthesiologists (ASA) IV or V **Planned recruitment** 120 participants (1:1:1)
Interventions	**Intervention 1** Oral 2 g TXA 2 hours before surgery **Intervention 2** Oral 2 g TXA 2 hours before surgery and 2 g TXA 2 hours after surgery **Comparator** Oral 2 g TXA 2 hours before surgery, 2 g TXA orally after 2, 6 and 10 hours
Outcomes	Hidden blood loss Total blood loss Maximum haemoglobin decline Operative hip swelling rate Range of hip flexion and abduction Postoperative Harris Score Length of stay Postoperative compliance
Starting date	31 August 2018
Contact information	Guanglin Wang 37 Guoxue Lane, Wuhou District, Chengdu, Sichuan, China guanglwhx@gmail.com
Notes	"Recruiting" RC emailed author for update 23 February 2022 to understand the status of this trial and how this trial, and trials ChiCTR1800017038, ChiCTR1800017094 and ChiCTR1800017095, relate to each other. RC accessed trial page 25 November 2022 and trial information last updated July 2018 when status was 'recruiting'.

**Table:** ChiCTR1800018751

Study name	'Comparison of different combined regimens of tranexamic acid in primary total knee arthroplasty: a prospective study of efficacy and safety'
Methods	Randomised controlled trial China
Participants	**Inclusion criteria** Adults (60 to 90 years) with unilateral degenerative osteoarthritis of the knee according to diagnostic criteria for knee osteoarthritis (ICD‐10 M17.901) Normal preoperative haemoglobin (Hb) levels, normal coagulation function and renal function Normal deep veins in the legs on colour Doppler echography Stable concurrent chronic diseases, if any **Exclusion criteria** Anaemic Abnormal coagulation function Liver or kidney dysfunction History of bleeding disorders History of deep vein thrombosis (DVT) Hyper‐coagulability or risk factors of thrombosis (e.g. atrial fibrillation, coronary stenting, tumours or long‐term use of hormones) Known history of TXA hypersensitivity **Planned recruitment** 160 participants (1:1:1:1)
Interventions	**Intervention 1** TXA: IV 1 g+ IA 3 g **Intervention 2** TXA: IV 1 g+ IA 2 g **Intervention 3** TXA: IV 1 g+ IA 1 g **Comparator** TXA: IV 1 g+ IA 0 g
Outcomes	Drainage volume, haemoglobin, thromboembolic event, transfusion unit, healing of incision
Starting date	July 2018
Contact information	Jun Wu wujun2310@126.com
Notes	RC emailed author for update from 23 February 2022 and on 3 separate occasions, but no response. Trial page last accessed 25 November 2022 when trial status remains as "recruiting". Trial registered 7 October 2018 and there have been no updates since.

**Table:** ChiCTR1800019261

Study name	'The blood sparing, anti‐fibrinolytic and anti‐inflammatory effects of multiple doses of oral tranexamic acid in total knee arthroplasty: a randomized controlled trial'
Methods	Randomised controlled trial China
Participants	**Inclusion criteria** Primary unilateral total knee arthroplasty Normal platelet and coagulation function preoperatively Normal intravenous ultrasound in the lower extremities preoperatively Able and willing to provide signed informed consent **Exclusion criteria** Systemic infections such as pulmonary infection or urinary tract infection not controlled preoperatively Abnormal immune function or combined immune‐related disease preoperatively Abnormal inflammatory index preoperatively (C‐reactive protein > 20 mg/L) Abnormal fibrinolysis index preoperatively (FDP > 5 mg/L) Discontinuation of oral anticoagulants or aspirin less than 1 week Allergy to tranexamic acid (TXA) or dexamethasone or have contraindications At high risk of thrombosis History of deep vein thrombosis (VTE, calf muscle vein thrombosis or superficial vein thrombosis, pulmonary embolism) Serious liver and kidney dysfunction **Planned recruitment** 120 participants (1:1)
Interventions	**Intervention** 2 g oral TXA 2 h before surgery, 2 g oral TXA 3 h and 9 h postoperatively **Comparator** 2 g oral TXA 2 h before surgery, 1 g oral TXA 3 h and 9 h postoperatively
Outcomes	Blood loss, Hb, TEG, ESR, CRP, IL‐6, IL‐10, knee ROM, KSS, perioperative complications: deep vein thrombosis, swelling, pain, length of hospital stay, transfusion
Starting date	November 2018
Contact information	zongke@126.com
Notes	RC emailed author from Spring 2022 and on 3 separate occasions, but no response. Trial page last accessed 25 November 2022 and trial status remains as "recruiting', but the trial record has not been updated since 2 November 2018.

**Table:** ChiCTR1900020498

Study name	'Efficacy of tranexamic acid combined with carbazochrome sodium sulfonate on blood loss in primary total hip arthroplasty using a direct anterior approach: a prospective randomized controlled trial'
Methods	Randomised controlled trial China
Participants	**Inclusion criteria** Adults (18 to 85 years) with osteoarthritis or osteonecrosis of the femoral head (Ficat III or IV) undergoing THA BMI 20 to 35 kg/m^2^ American Society of Anesthesiologists grade I to III **Exclusion criteria** TXA, sodium carboxylic acid, anaesthesia drug allergy Rigid hip joint, severe femoral deformity Posterior wall of the acetabulum was removed, and there was a history of surgery in the posterior hip Osteoarthritis (Crowe type 3 or 4) of DDH History of thromboembolic disease (DVT), pulmonary embolism (PE), coagulopathy, recent arterial thromboembolic events (such as myocardial infarction or stroke) Hypercoagulability Haemophilia Severe cardiovascular and respiratory diseases Kidney and liver failure, kidney transplantation history Persons who refuse to participate in or refuse to accept blood products **Planned recruitment** 90 participants (1:1:1)
Interventions	**Intervention 1** Tranexamic acid (intravenous) + carbazochrome sodium sulfonate (infiltration around joint capsule) **Intervention 2** Tranexamic acid (intravenous) **Comparator** Placebo
Outcomes	**Primary outcomes** Total blood loss, HCT, Hb, reduction in haemoglobin concentration, transfusion rates **Secondary outcomes** Intraoperative blood loss, hospital stay, thromboembolism
Starting date	January 2019
Contact information	Kang Pengde kangpd@163.com
Notes	RC emailed author from November 2022 and on 3 separate occasions, but no response. Trial page last accessed on 29 November 2022 and trial status remains as 'recruiting', but this has not been updated since January 2019.

**Table:** ChiCTR2000035271

Study name	'Effects of low‐dose tranexamic acid intra‐articular injection on blood loss, pain, and functional recovery after total knee arthroplasty: a randomized controlled trial'
Methods	Randomised controlled trial China
Participants	**Inclusion criteria** Adults diagnosed with knee osteoarthritis requiring unilateral primary TKA surgery American Society of Anesthesiologists grade before operation: I‐III Over 18 years of age **Exclusion criteria** Allergic to tranexamic acid Operative knee deformity (buckling deformity > 20 degrees; varus and valgus deformity BBB 0 20 degrees) and stiffness of joints Revision partial or total knee replacement, suppurative arthritis or post‐traumatic arthritis Serious cardiovascular and respiratory diseases Kidney and liver failure, history of kidney transplantation Refusal to participate in or accept blood products **Planned recruitment** 120 participants (1:1)
Interventions	**Intervention** TXA, IA 0.5 g **Comparator** Placebo, IA 0.5 g
Outcomes	**Primary outcomes** Pain score, morphine consumption **Secondary outcomes** Total blood loss, invisible blood loss, intraoperative blood loss, volume of drainage, inflammatory markers, range of motion, blood transfusion rate, length of hospital stay
Starting date	August 2020
Contact information	Luo Yue 760455936@qq.com
Notes	RC emailed author for update from 1 March 2022 and on 3 separate occasions. Trial page last accessed 25 November 2022 and trial status remains as 'recruiting' but this has not been updated since May 2020.

**Table:** ChiCTR2000039368

Study name	'Efficacy and safety of postoperative intravenous tranexamic acid in total knee arthroplasty: a prospective randomized controlled study'
Methods	Randomised controlled trial China
Participants	**Inclusion criteria** Adults with knee osteoarthritis diagnosis and total knee arthroplasty No previous history of venous thrombosis Complete clinical data Aged 55 to 80 years **Exclusion criteria** Severe valgus deformity of knee joint Abnormal fibrinolytic system Receiving anticoagulant therapy for a long time DVT before operation Severe hepatic and renal insufficiency Significantly increased inflammatory indexes or infection **Planned recruitment** 168 participants (1:1:1)
Interventions	**Intervention** Tranexamic acid was applied a single time after surgery **Comparator** Tranexamic acid
Outcomes	**Primary outcomes** Hb, HCT, DVT
Starting date	October 2020
Contact information	Chenxi Xue 810987347@qq.com
Notes	RC emailed author from 29 November 2022 and on 3 separate occasions, but had no response. Trial page last accessed on 29 November 2022 where trial status remains as 'recruiting' but this has not been updated since January 2021.

**Table:** ChiCTR2100045474

Study name	'Effect of tranexamic acid on blood protection in patients undergoing total hip arthroplasty'
Methods	Randomised controlled trial China
Participants	**Inclusion criteria** At least 18 years old ASA classification II to IV Intend to undergo total hip replacement surgery under intraspinal anaesthesia **Exclusion criteria** Re‐operation of the hip joint Allergic to tranexamic acid ingredients Thrombosis **Planned recruitment** 120 participants (1:1:1:1)
Interventions	**Intervention 1** Tranexamic acid low‐dose group, 0.5 g tranexamic acid intravenous drip before skin incision + 0.5 g tranexamic acid intravenous drip after prosthesis installation **Intervention 2** Tranexamic acid pump group, 1 g tranexamic acid intravenous drip before skin incision + 0.5 g/h tranexamic acid continuous pump for 2 hours **Intervention 3** Tranexamic acid high‐dose group, 1 g tranexamic acid intravenous drip before skin incision + 1 g tranexamic acid intravenous drip after prosthesis installation **Comparator** Control group, 100 mL normal saline intravenous drip before skin incision + 100 mL normal saline intravenous drip after prosthesis installation
Outcomes	**Primary outcomes** Blood loss (intraoperative blood loss + drainage volume 48 hours after operation), the amount of blood transfusion, thromboelastogram, coagulation function, routine blood test **Secondary outcomes** Ultrasound examination of lower extremity vessels, renal dysfunction, thromboembolism, epilepsy, death
Starting date	April 2021
Contact information	Deng Jicai: 247545782@qq.com
Notes	Trial page accessed 5 December 2022. Trial record last updated November 2021 when recruitment status was 'recruiting'.

**Table:** CTRI/2022/03/041001

Study name	'Study on the effectiveness of tranexamic acid(drug) in reducing blood loss during hip surgeries'
Methods	Randomised controlled trial India
Participants	**Inclusion criteria** ASA grades I and II Aged 40 to 65 years Elective surgery Body mass index < 35 **Planned recruitment** 60 participants
Interventions	**Intervention** Tranexamic acid 1 g given in 100 mL NS as a bolus dose pre‐operatively **Comparator** 100 mL NS preoperatively
Outcomes	Intraoperative and postoperative blood loss, HR, BP, SPO_2_, urine output, amount of blood in suction drain
Starting date	March 2022
Contact information	Dr S Vigneshwaran: drsvigneshwaran@gmail.com
Notes	RC accessed trial page 29 November 2022 and trial status states 'recruiting'. Trial page last updated March 2022.

**Table:** EUCTR‐2008‐007110‐29‐FR

Study name	'An international randomised, double‐blind, placebo‐controlled study to assess the efficacy and safety of 2 dosing regimens of FIBRINOGENE T1 in the treatment of peri‐operative bleeding associated with revision total hip arthroplasty'
Methods	Randomised controlled trial France
Participants	**Inclusion criteria** Women of childbearing potential, aged > 18 years old, undergoing elective revision total hip arthroplasty including major acetabular and/or femoral reconstruction **Planned recruitment** 90 participants
Interventions	**Intervention 1** Fibrinogen **Intervention 2** Fibrinogen **Comparator** Placebo
Outcomes	Peri‐operative bleeding, transfusion needs, safety
Starting date	Not reported
Contact information	Not reported
Notes	Page last accessed 25 November 2022. Status still reported as 'ongoing'. Trial first entered into database March 2009. No publication details. No author contact information.

**Table:** EUCTR‐2016‐000071‐24‐ES

Study name	'Time of administration of tranexamic acid to prevent bleeding in total knee arthroplasty' 'Determinar el momento de administración de ácido tranexámico para prevenir el sangrado en cirugía de prótesis total de rodilla'
Methods	Randomised controlled trial Spain
Participants	**Inclusion criteria** Adult patients aged between 18 and 85 years, scheduled for primary and unilateral total knee arthroplasty with tourniquet **Planned recruitment** 192 participants
Interventions	Intravenous tranexamic acid vs intravenous placebo
Outcomes	Volume of postoperative bleeding, total postoperative bleeding, incidence of clinically significant thromboembolic events
Starting date	Not reported
Contact information	ebisbe@parcdesalutmar.cat
Notes	RC emailed author 14 July 2021 and on 3 separate occasions, but no response. Trial page last accessed 25 November 2022 and trial status remains as 'ongoing', but the record has not been updated since 2016.

**Table:** EUCTR‐2018‐003537‐15

Study name	'Different dosing of tranexamic acid in patients undergoing elective total hip or knee arthroplasty. A randomized, controlled, double‐blinded clinical trial'
Methods	Randomised controlled trial Austria
Participants	**Inclusion criteria** Undergoing elective total hip or knee arthroplasty under general anaesthesia Age ≥ 18 years Written informed consent given **Planned recruitment** 180 participants
Interventions	Different doses of TXA (specific doses not reported)
Outcomes	Total perioperative blood loss in mL, difference of haemoglobin levels before surgery and 24 h after surgery, total number of red blood cells transfused, complications (cardiovascular and neurological), plasma tranexamic acid concentrations at different time points, results of EMT – ClotPro at the same time points, results of ROTEM, thrombin generation, the correlation of plasma tranexamic acid concentrations and ClotPro/ROTEM results, correlation of plasma tranexamic acid concentrations and thrombin generation
Starting date	Not reported
Contact information	lukas.infanger@meduniwien.ac.at
Notes	RC emailed author 1 March 2022 on 3 separate occasions, but no response. Trial page last accessed 29 November 2022 and trial status remains as 'ongoing'; no results posted but the record has not been updated since March 2019.

**Table:** EUCTR‐2020‐003321‐32‐DK

Study name	'Evaluation of intra articular tranexamic acid for reduction of total blood loss in total hip arthroplasty' 'Evaluering af intraartikulær Tranexamsyre til reduktion af total blødning ved total hoftealloplastik'
Methods	Randomised controlled trial Denmark
Participants	**Inclusion criteria** 18 years and over, undergoing surgery for unilateral total hip arthroplasty and able to give consent **Planned recruitment** 100 participants
Interventions	IV TXA at the start of surgery + injection of TXA into the joint (end surgery) vs TXA at the start of surgery + injection of equal volume of NaCl (placebo)
Outcomes	Total blood loss, total bleeding 2 days after surgery, likewise the amount of blood transfusion and the thromboembolic events within the first 90 days of surgery
Starting date	Not reported
Contact information	lph821@alumni.ku.dk
Notes	RC emailed author for update from 4 January 2022 and on 3 separate occasions. Author responded that the trial is at a standstill due to COVID; not sure when elective surgeries will start again. Page re‐accessed on 7 July 2022 and trial remains 'ongoing'.

**Table:** EUCTR‐2020‐004167‐29‐BE

Study name	'Study of the noninferiority of an oral versus intravenous administration of tranexamic acid in total hip arthroplasty'
Methods	Randomised controlled trial
Participants	**Inclusion criteria** Adults > 18 years old Physical status score ASA I to III Scheduled for total hip arthroplasty under regional anaesthesia (supra‐inguinal fascia‐iliaca block) combined with general or spinal anaesthesia **Exclusion criteria** Kidney injury Pregnant women Diagnosed with thromboembolic disease in last 12 months Undergone a digestive surgery that could lead to malabsorption (sleeve, bypass, gastrectomy, intragastric balloon) Insulin‐dependent diabetic with gastroparesis **Planned recruitment** 256 participants
Interventions	Oral tranexamic acid vs intravenous tranexamic acid
Outcomes	Blood loss, Hb levels, renal insufficiency, hospital stay, complications
Starting date	Not reported
Contact information	mcarella@chuliege.be
Notes	RC accessed trial page 30 November 2022. Trial information last updated February 2021 when status was 'ongoing'.

**Table:** NCT03623789

Study name	'Blood‐saving effect of combined intravenous tranexamic acid with topical Floseal® application total hip arthroplasty'
Methods	Randomised controlled trial Taiwan
Participants	**Inclusion criteria** Osteoarthritis of the hip secondary to degeneration, inflammatory arthritis, gouty arthritis, acetabular dysplasia or osteonecrosis of the femoral head Undergoing primary unilateral minimally invasive total hip arthroplasty Age > 18 years and < 90 years Failure of medical treatment or rehabilitation Haemoglobin > 110 g/L No use of non‐steroid anti‐inflammatory agent 1 week before operation **Exclusion criteria** Preoperative haemoglobin ≤ 110 g/L History of infection or intra‐articular fracture of the affective hip Renal function deficiency (GFR < 30 mL/min/1.73 m^2^) Elevated liver enzyme (aspartate transaminase (AST)/alanine transaminase(ALT) level more than twice normal range), history of liver cirrhosis, impaired liver function (elevated total bilirubin level) and coagulopathy (including long‐term use of anticoagulant) History of deep vein thrombosis, ischaemic heart disease or stroke Contraindications of tranexamic acid, Floseal or rivaroxaban Allergy to tranexamic acid, Floseal, rivaroxaban or the excipients History of heparin‐induced thrombocytopenia (HIT) Coagulopathy or bleeding tendency caused by organ dysfunction, such as cirrhosis, bone marrow suppression etc. Active bleeding disorder, such as intracranial haemorrhage, upper gastrointestinal bleeding, haematuria Known allergies to materials of bovine origin **Planned recruitment** 90 participants
Interventions	**Intervention 1** TXA pre‐operatively and 2 boluses TXA postoperatively intravenously and Floseal topical application **Intervention 2** TXA pre‐operatively and 2 boluses TXA postoperatively intravenously (no Floseal) **Comparator** Normal saline (without TXA and Floseal)
Outcomes	Total blood loss, acute intraoperative blood loss, change in haemoglobin level, blood transfusion rate, thrombosis risk evaluation, incidence of wound complications
Starting date	15 August 2018
Contact information	Jun‐Wen Wang, MD 886‐7‐7317123 wangjw@adm.cgmh.org.tw
Notes	Page accessed 29 November 2022. Trial page says status is 'recruiting'. Trial status last updated January 2020.

**Table:** NCT03897621

Study name	'The effect of tranexamic acid on blood coagulation in total hip arthroplasty surgery'
Methods	Randomised controlled trial USA
Participants	**Inclusion criteria** Adults (age range of 18 to 85 years) ASA Physical Status (PS) I to III Undergoing unilateral, primary, total hip arthroplasty **Exclusion criteria** History of significant coagulopathy or on anticoagulation therapy Pregnant or breastfeeding Anaemic (Hb < 80 g/L) or received blood transfusion within 1 week before surgery Receiving subcutaneous heparin on the same day prior to surgery **Planned recruitment** 50 participants
Interventions	**Intervention** Tranexamic acid will be administered intravenously after induction of anaesthesia (a bolus of 10 mg/kg or maximal dose of 1 g) **Comparator** Normal saline (a bolus of sodium chloride 0.9%, 0.1 mL/kg or maximal dose of 10 mL
Outcomes	Fibrinolysis, blood loss, blood transfusion, pre and postoperative haemoglobin level, wound infection, haematoma, thrombotic events
Starting date	20 May 2019
Contact information	Uzung Yoon, MD: uzung.yoon@gmail.com
Notes	Page accessed 5 December 2022. The record was last updated on 27 September 2022 where the study was listed as 'recruiting'. Estimated enrolment of 200 participants.

**Table:** NCT04691362

Study name	'Noninferiority oral tranexamic acid vs intravenous administration in total hip arthroplasty'
Methods	Randomised controlled trial Belgium
Participants	**Inclusion criteria** ASA physical status I to III Scheduled for primary total hip arthroplasty **Exclusion criteria** Renal failure with serum creatinine level higher than 1.40 mg/dL Thromboembolic events in last 12 months before surgery Pregnancy Congenital or acquired coagulation diseases History of gastric surgery that could lead to malabsorption Diabetic gastroparesis **Planned recruitment** 230 participants
Interventions	**Intervention** Oral administration of 2 g tranexamic acid 2 hours before skin incision and 2 g oral tranexamic acid 4 hours after first administration **Comparator** Intravenous administration of 1 g tranexamic acid 30 minutes before skin incision and 1 g intravenous tranexamic acid 4 hours after first administration
Outcomes	Total blood loss, serum concentration of tranexamic acid, serum haemoglobin variation, incidence of blood transfusion, incidence of thromboembolic complications, length of hospital stay
Starting date	January 2021
Contact information	Jean François Brichant, University of Liege
Notes	Page accessed 5 December 2022 ‐ study completion date planned as September 2023. Estimated enrolment of 256 participants.

**Table:** NCT04992052

Study name	'Bone wax use for hemostasis during primary unilateral total knee arthroplasty'
Methods	Randomised controlled trial USA
Participants	**Inclusion criteria** Scheduled for primary unilateral total knee arthroplasty Preoperative haemoglobin > 110 g/L Preoperative platelet count of > 150 x 10^9^/L **Exclusion criteria** Unable to take aspirin or apixaban for VTE prophylaxis Allergy to any of the ingredients in bone wax (beeswax, paraffin or isopropyl palmitate) Taking clopidogrel (Plavix), ticagrelor (Brilinta) or prasugrel (Effient), or any other antiplatelet medication (except for aspirin 81 mg) Unable to receive IV tranexamic acid for any reason Requiring anticoagulant treatment prior to surgery **Planned recruitment** 100 participants
Interventions	**Intervention** Bone wax applied to the exposed cancellous surfaces of the bone **Comparator** Bone wax will not be used in this group (control)
Outcomes	Blood loss, Knee Society scoring system
Starting date	July 2021
Contact information	Nancy Dengler: ndengler@northwell.edu
Notes	Page accessed 5 December 2022. Estimated study completion date September 2023. Estimated enrolment of 100 participants.

**Table:** NCT05099276

Study name	Extended postoperative oral tranexamic acid in knee replacement
Methods	Randomised controlled trial USA
Participants	**Inclusion criteria** Primary total knee replacement in ambulatory setting Willing to participate Physical therapy on site at Campbell Clinic Wolf River **Exclusion criteria** Revision total knee replacement Preoperative use of anticoagulants other than 81 mg aspirin Previous history of thromboembolic event Previous history of cancer other than non‐melanoma skin cancers **Planned recruitment** 40 participants
Interventions	**Intervention** 1950 mg tranexamic acid (3 capsules) given in post‐anaesthesia room and 3 capsules for postoperative day 1 and 3 capsules for postoperative day 2 **Placebo** 3 capsules of cellulose given in post‐anaesthesia room and 3 capsules for postoperative day 1 and 3 capsules for postoperative day 2
Outcomes	Hb, pain score, range of motion, adverse events
Starting date	December 2021
Contact information	Dr Marcus C Ford, Campbell Clinic, USA
Notes	Trial page accessed 5 December 2022. Trial information last updated January 2022, when status was 'enrolling by invitation'. Study completion expected by December 2023.

**Table:** UMIN000047607

Study name	'Randomized control trial for suppression of perioperative bleeding byetween tranexamic acid and surge cell'
Methods	Randomised controlled trial Japan
Participants	**Inclusion criteria** Adult (18 to 85 years) Osteoarthritis of the knee or hip **Exclusion criteria** Other than osteoarthritis History of spine, hip, knee surgery Bilateral simultaneous THA or TKA Contralateral THA within 1 year Reoperation Stroke Neuromuscular disease paralysis **Planned recruitment** 100 participants
Interventions	**Intervention** Tranexamic acid 1 g to 2 g **Comparator** Surgicel less than 3 g
Outcomes	Estimated blood loss
Starting date	May 2022
Contact information	Kentaro Iwakiri: kenpiecekenpiece@yahoo.co.jp
Notes	RC accessed trial page 30 November 2022. Trial information last updated 7 May 2022 where status was 'ongoing'.

**Table:** Yang 2021

Study name	Prospective, randomised, controlled study on the efficacy and safety of different strategies of tranexamic acid with total blood loss, blood transfusion rate and thrombogenic biomarkers in total knee arthroplasty
Methods	Randomised controlled trial China
Participants	**Inclusion criteria** Adult > 18 and < 100 years Undergoing primary total knee arthroplasty **Exclusion criteria** Allergy to tranexamic acid Preoperative hepatic or renal dysfunction Serious cardiac or respiratory disease, including coronary artery stent placement or bypass Congenital or acquired coagulopathy, as evidenced by an international normalised ratio of > 1.4 or a partial thromboplastin time of > 1.4 times normal Preoperative platelet count of < 150 x 10^9^/L History of a prothrombotic condition Pregnancy or breastfeeding Diagnosis of inflammatory arthritis Preoperative haemoglobin level of < 100 g/L **Planned recruitment** 250 participants
Interventions	Placebo vs TXA, IV vs TXA, topical irrigation vs TXA, periarticular tissue injection vs TXA, drainage injection
Outcomes	Total blood loss, blood transfusion rate, drainage volume, plasma D‐dimer, PAI‐1, TAT and F1+2 levels, maximum haemoglobin drop, wound complications, venous thromboembolism, length of hospital stay
Starting date	—
Contact information	—
Notes	Trial registration accessed 29 November 2022. Trial status was "recruiting".

ANFH: avascular necrosis of the femoral head; ASA: American Society of Anaesthesiologists' (ASA) (score: classification of physical health); BMI: body mass index; BP: blood pressure; CRP: C‐reactive protein; DDH: developmental dysplasia of the hip; DVT: deep vein thrombosis; ESR: erythrocyte sedimentary rate; FDP: fibrin(ogen) degradation products; h: hours; GFR: glomerular filtration rate; Hb: haemoglobin; Hct: haematocrit; HR: heart rate; HSS: Hospital for Special Surgery score; IA: intra‐articular; IV: intravenous; KSS: Knee Society Score; OA: osteoarthritis; PE: pulmonary embolism; ROM: range of motion; SPO_2_: saturated oxygen; TEG: thromboelastography; THA: total hip arthroplasty; TKA: total knee arthroplasty; TXA: tranexamic acid; VTE: venous thromboembolism

## Differences between protocol and review

The protocol for the review was prospectively published in 2019 (Gibbs 2023). This review deviates from the protocol in the following ways:

**1. Definition of "Red cell transfusions up to 30 days post‐surgery (units)" outcome**

In hindsight, this definition was ambiguous. In the review, under this definition, we recorded the mean number of red blood cell transfusions.

**2. Definition of outcomes**

Following advice from the Cochrane Injuries editorial base, we have changed the primary outcome from "Proportion of people needing allogeneic blood transfusion" to "Risk of allogeneic blood transfusion", and we defined more clearly the secondary outcome of adverse events as the "risk of" an adverse event.

**2. No thromboembolic events**

Many of the included papers reported that in their study there were ‘no thromboembolic events’. We have taken that to mean that both pulmonary embolism and deep vein thrombosis events were zero.

**3. Intention‐to‐treat (ITT)**

In some papers, it was not explicitly stated whether ITT analysis was done or not. In these situations, we looked at the numbers in the study flowchart, as well as the data, to assess how the analyses were conducted.

**4. Definition of "mean number of transfusions"**

When considering the data for this outcome in preparation for the network meta‐analyses, we excluded any studies where the mean and/or standard deviation was 0. This was done following guidance from an experienced statistician (Prof N Welton). We were also advised to exclude the mean number of transfusions data from any studies where the median interquartile range was reported, as this denotes skewed data.

**5. Definition of ‘N’**

In the protocol, it was not clearly explained that for the "Mean number of transfusions" outcome, N was used to describe the number of patients randomised and not the number of patients transfused.

**6. Network meta‐analysis (NMA) software**

We used BUGSnet v1.1.0 (an R package for Bayesian network meta‐analysis) to perform the network meta‐analyses rather than Stata as we had stated in the protocol. This choice was made because we preferred a Bayesian approach.

**7. Rounding up of doses**

When inputting pairwise data into RevMan, we categorised study data by comparison, outcome and then dose. Where possible, we tried to divide studies into 1 g, 2 g, 3 g or more than 3 g options. Sometimes this categorisation did not work; in these situations, we rounded up doses. This was done consistently amongst the studies where this was a problem.

**8. Sensitivity and subgroup analysis**

In the protocol we stated that we would perform the main analysis only with trials at low risk of bias, but we felt that we should conduct the main analysis with all trials included and then perform a sensitivity with trials at low risk of bias, as this approach is more commonly used. In the event, however, we decided not to do this because it breaks the network in such a way that we cannot directly compare results, and also because the trials were generally of good quality, so it was not expected to make a difference to our conclusions.

Similarly, we have not produced any subgroup analyses as there were not enough data for a reliable analysis.
